# Hydroxyquinoline-functionalised aza-crown macrocycles for lanthanide coordination

**DOI:** 10.1039/d5dt03015c

**Published:** 2026-01-28

**Authors:** Christina Siakalli, Bradley E. Osborne, Ryan K. Brown, Claudia Rocco, Dominik Weiss, Enrique V. García-España, Pascal V. Grundler, Anzhelika N. Moiseeva, Zeynep Talip, Nicholas P. van der Meulen, Michelle T. Ma, Nicholas J. Long

**Affiliations:** a Department of Chemistry, Imperial College London, Molecular Sciences Research Hub White City Campus London W12 0BZ UK n.long@imperial.ac.uk; b School of Biomedical Engineering and Imaging Sciences, King's College London, St Thomas’ Hospital London SE1 7EH UK michelle.ma@kcl.ac.uk; c Department of Earth Science and Engineering, Imperial College London South Kensington Campus London SW7 2BP UK; d ICMol, Departament de Química Inorgànica, Universitat de València C/Catedrático José Beltrán 2 Paterna 46980 Spain; e Center for Radiopharmaceutical Sciences, PSI Center for Life Sciences 5232 Villigen-PSI Switzerland; f Laboratory of Radiochemistry, PSI Center for Nuclear Engineering and Science 5232 Villigen-PSI Switzerland

## Abstract

Emerging therapeutic radiolanthanides have utility for systemic molecular radiotherapy in nuclear medicine, provided that suitable chemical technology is available to incorporate them into receptor-targeted radiopharmaceuticals. In this work, *N*,*N*′-bis(8-hydroxyquinoline-2-ylmethyl)-4,13-diaza-18-crown-6 (H_2_KHQ) was synthesised, and its binding ability, thermodynamic stability and selectivity for Ln^3+^ ions (Ln^3+^ = La, Tb, and Lu) investigated. The design of H_2_KHQ involves pendant arms featuring 8-hydroxyquinoline units, known to possess metal-chelating properties and desirable activity in other therapeutic molecules. H_2_KHQ exhibited selectivity for the larger Ln^3+^ ions, confirmed by experimentally measured stability constants as well as DFT calculations. H_2_KHQ was able to bind the larger, non-radioactive La^3+^ and Tb^3+^ ions within 30 minutes at room temperature, forming a single, 2-fold symmetric species in solution. The structure of [La-HKHQ]^2+^, as determined by single crystal XRD, emphasized the need for high denticity chelators to satisfy the coordination sphere of the Ln^3+^, showing a 10-coordinate La^3+^ metal centre. H_2_KHQ was radiolabelled with [^161^Tb]TbCl_3_ under mild conditions in 92% radiochemical yield in promising proof-of-concept measurements.

## Abstract 2

Synthesis and evaluation of an 8-hydroxyquinoline-based macrocycle exhibiting selectivity for the larger lanthanide ions. Optical spectroscopy and radiolabelling with terbium-161 revealed the potential for dual-modal terbium(iii) theranostic probes.

## Graphical abstract

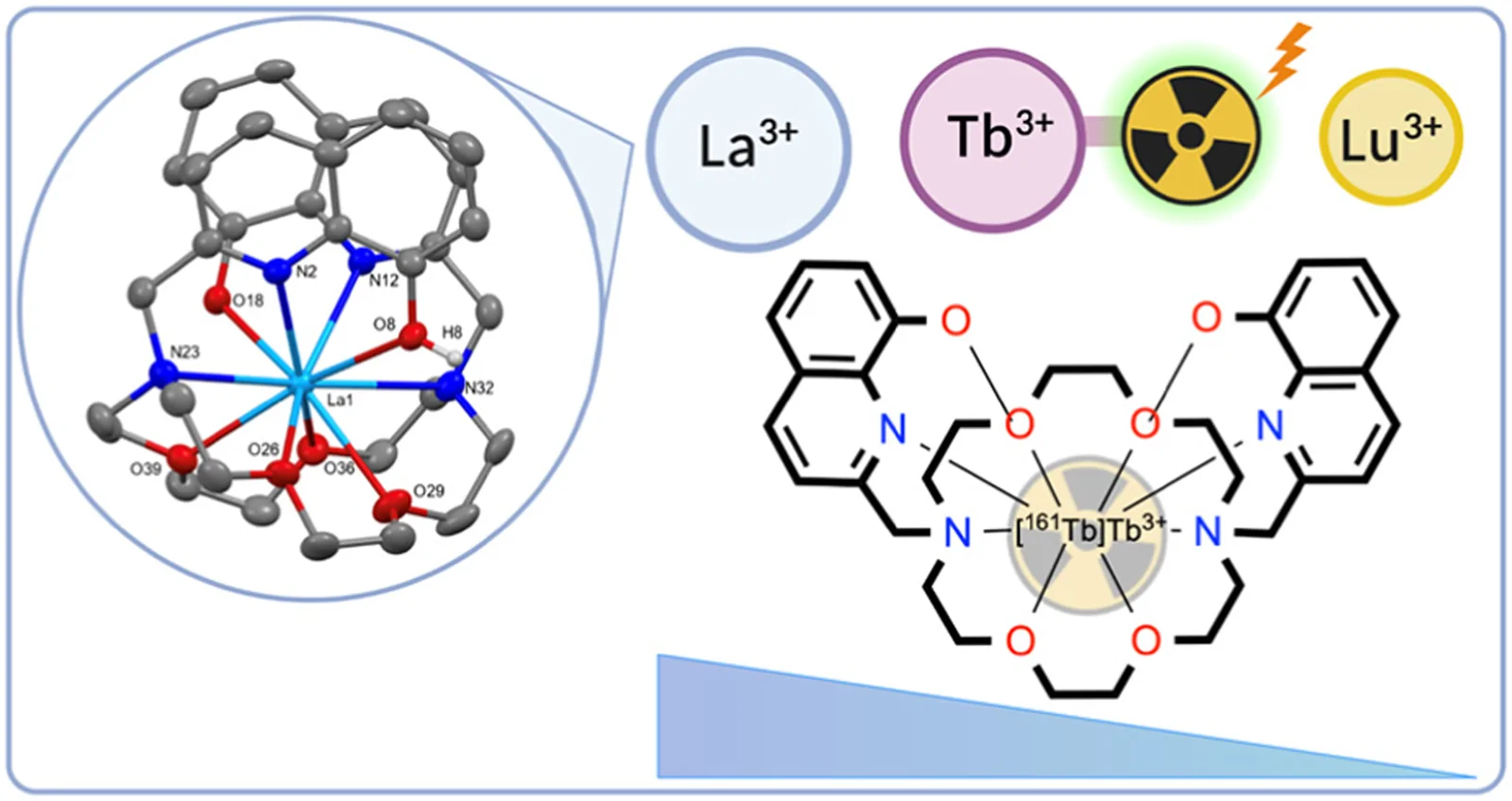

## Introduction

Radioactive nuclides of the lanthanides have significant utility in nuclear medicine for both diagnostic imaging and systemic radiotherapy. In particular, radiolanthanides that emit cytotoxic particles including beta (β^−^), alpha (α) and Auger Electrons (AE), have demonstrated efficacy in theranostic radiopharmaceuticals.^1–3^ Diagnostic and therapeutic (“theranostic”) pairs of radiopharmaceuticals typically utilise the same biologically active receptor-targeted vector to deliver either an imaging radionuclide or a cytotoxic therapeutic radionuclide to diseased tissue. This “look and treat” approach uses the radiotracer, in combination with either Positron Emission Tomography (PET) or Single Photon Emission Computed Tomography (SPECT) imaging to provide diagnostic information that guides decisions on suitability of the companion therapeutic radiotracer.^1,4^ For example, the PET radionuclide gallium-68 (^68^Ga, *t*_1/2_ = 68 min, β^+^) is commonly used in tandem with therapeutic lutetium-177 (^177^Lu, *t*_1/2_ = 6.64 days, β^−^) for the treatment of somatostatin receptor (SSTR)-positive neuroendocrine tumours, using an “octreotate” peptide attached to a macrocyclic chelator.^3,5,6^ While the use of the ^68^Ga/^177^Lu radionuclide pair is effective, “true theranostic” agents consisting of a pair of imaging and therapeutic radionuclides of the same element could provide significant advantages. In “true theranostic” pairs, the chemically identical imaging radiotracer and radiotherapeutic agent exhibit equivalent biodistributions: the imaging radiotracer can be used to determine accurate dosimetry of the radiotherapeutic agent. Examples include copper-64/copper-67 (^64^Cu, β^+^/^67^Cu, β^−^), terbium-155/terbium-161 (^155^Tb, γ/^161^Tb, β^−^, AE, γ) and scandium-44/scandium-47 (^44^Sc, β^+^/^47^Sc, β^−^).^3,7–9^ Terbium radioisotopes have potential for receptor-targeted theranostic radiopharmaceuticals. There are four clinically relevant radioisotopes: ^149^Tb (*t*_1/2_ = 4.12 hours, *E*_α_ = 3.97 MeV, *I*_α_ = 16.7%) for targeted α therapy, ^152^Tb (*t*_1/2_ = 17.5 hours, *E*_β+,av_ = 1.14 MeV, *I* = 20.3%) for PET and ^155^Tb (*t*_1/2_ = 5.3 days, *E*_γ_ = 86.6, 105.3 and 180.1 keV) for SPECT. Finally, ^161^Tb (*t*_1/2_ = 6.95 days) is a β^−^-emitter (154 keV) that undergoes low-energy internal conversion (IC), co-emitting high-energy AE (∼12.12 e^−^, <40 keV per decay) and γ-rays.^3,7,10^ In side-by-side preclinical studies, ^161^Tb has showcased superior *in vitro* and *in vivo* efficacy compared to ^177^Lu, alongside an ability to deliver higher absorbed doses.^10–14^ The co-emission of both long-range β^−^ and shorter-range AE emissions from ^161^Tb in comparison to ^177^Lu (β^−^ emitter only) is hypothesized to increase its efficacy and radiotherapeutic effect.^7,15,16^ Phase I/II clinical trials with ^161^Tb radiopharmaceuticals are currently evaluating the efficacy of ^161^Tb-based radiopharmaceuticals.^17^

**Fig. 1:**
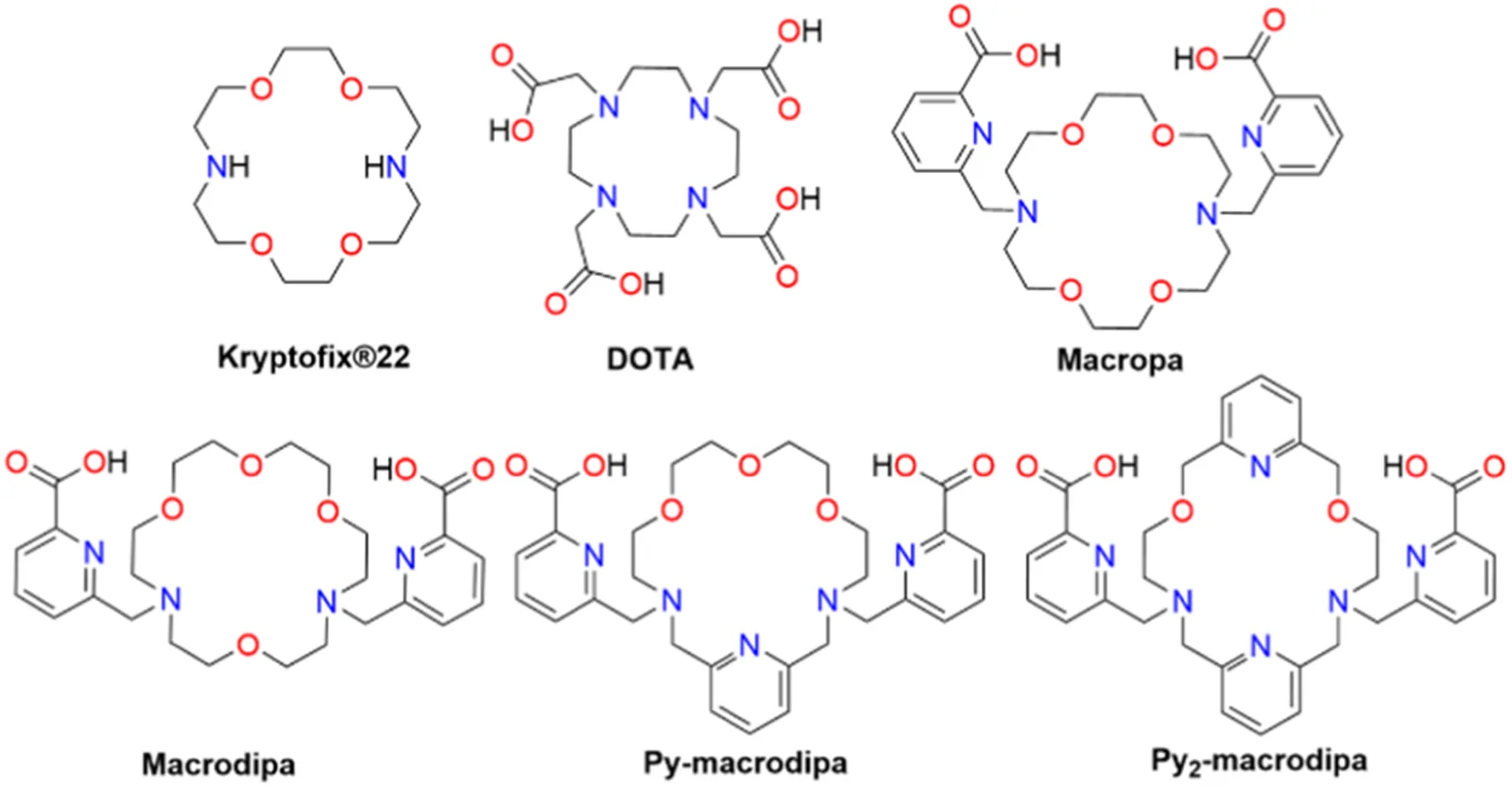
Examples of azamacrocyclic chelators.

A suitable chelating agent is required to coordinate radiolanthanides such as ^161^Tb and subsequently attach them to biologically active motifs that target surface receptors of diseased cells. Over the years, a series of aza-crown macrocyclic chelators have been developed, for coordination of rare-earth metals available for theranostic applications (Fig. 1). The coordination chemistry of macropa, macrodipa and next-generation analogues have been extensively studied with regards to their ability to bind clinically relevant radionuclides, including actinium-225 (^225^Ac) and lanthanum-135 (^135^La).^18–27^ Macropa displayed preferential binding with radionuclides of larger ionic radius, over smaller radionuclides whereas macrodipa, py-macrodipa and py_2_-macrodipa complexes with both large and small radionuclides demonstrated increased thermodynamic and kinetic stability.^21–23^ Blei *et al.* have further highlighted the abilities of macropa to bind radiometals with larger ionic radii (lead-212 (^212^Pb) and lanthanum-133 (^133^La)) in 100% radiochemical conversion (RCC), comparable to labelling with ^225^Ac.^27^ Macropa was also able to complex the smaller radionuclide, ^177^Lu, however, the complex's kinetic stability was lower and higher ligand concentrations were required to achieve satisfactory RCC.^27^ Still, macropa is one of the two state-of-the-art chelators for ^225^Ac, alongside 1,4,7,10-tetraazacyclododecane-1,4,7,10-tetraacetic acid (DOTA) and is currently being used in clinical trials.^28,29^

In this work, we explore a diaza-18-crown-6 derivative, H_2_KHQ, containing two 8-hydroxyquinoline pendant arms. This chelator is related to macropa, in that the picolinic acid groups of the latter are replaced by 8-hydroxyquinoline motifs. H_2_KHQ has highly similar topology to macropa, with increased rigidity in the pendant arms. H_2_KHQ has been previously studied for the complexation of divalent metals ions of zinc, barium, copper, as well as monovalent potassium and sodium.^30^*However, it has not been reported for the complexation of trivalent lanthanides or for use in nuclear medicine*. In general, 8-hydroxyquinoline units and derivatives are widely used in medicinal chemistry and drug discovery.^31–36^ They are well-known to possess metal chelating properties and have been assessed as ionophores,^37,38^ antiseptic,^39^ antioxidant,^40,41^ and anticancer agents.^42,43^ In addition, they exhibit photophysical properties enabling them to act as sensitizers for Ln^3+^ in a wide variety of applications including biological imaging and materials chemistry.^44–49^

## Results

### Ligand synthesis

**Scheme 1:**
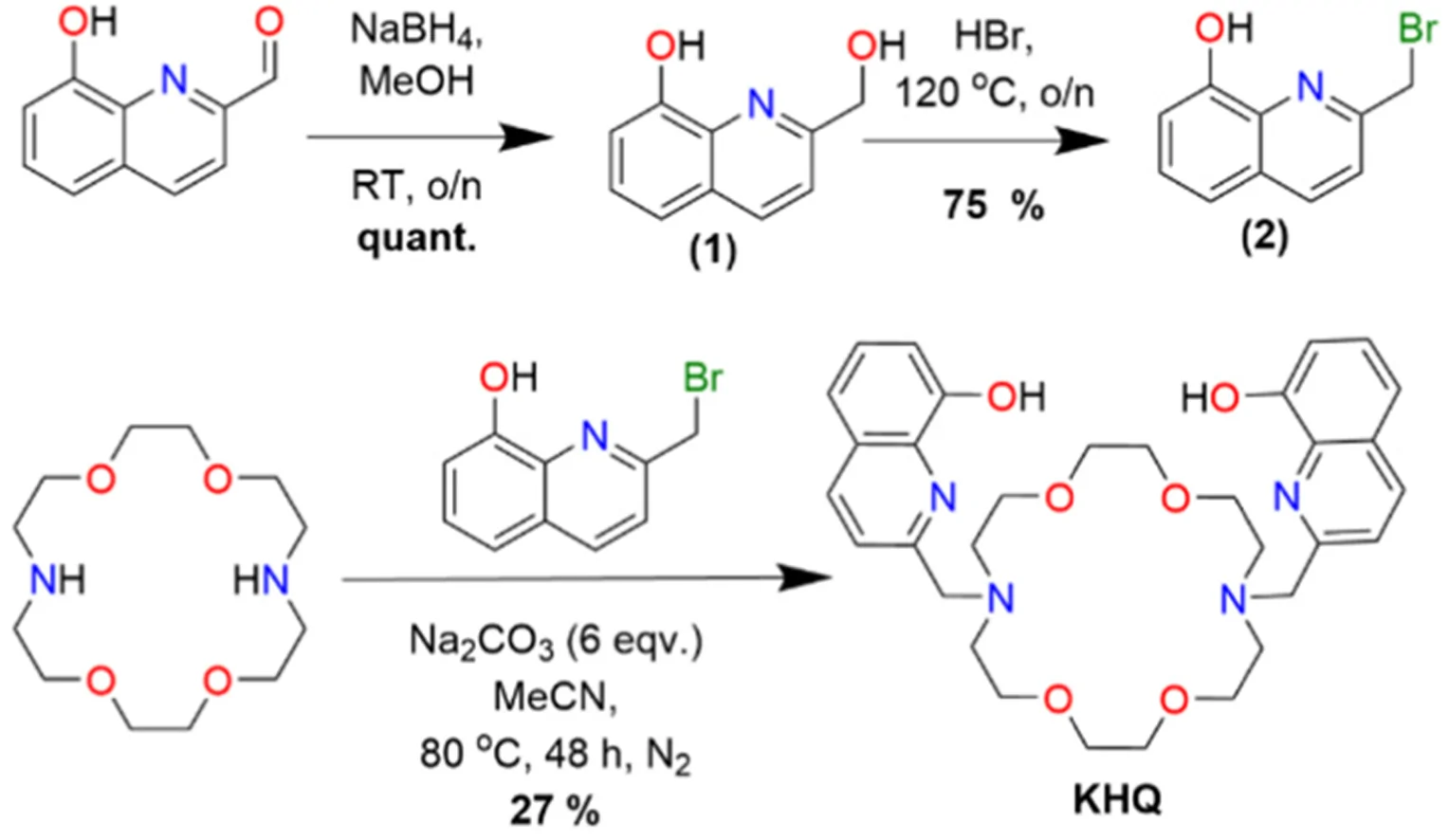
Synthesis of H_2_KHQ.

H_2_KHQ (*N*,*N*′-bis(8-hydroxyquinoline-2-ylmethyl)-4,13-diaza-18-crown-6) was synthesised in a three-step reaction as shown in Scheme 1. 8-Hydroxyquinoline-2-carbaldehyde was reduced and brominated following a previous procedure.^50^ Subsequent attachment of intermediate 2 onto a Kryptofix®22 backbone was carried out *via* nucleophilic substitution in the presence of sodium carbonate, adapted from previous procedures.^24,26^ The product was isolated as a pure, white solid in 27% yield post purification (Fig. S1–S6, SI).

### Characterisation of lanthanide complexes

**Fig. 2:**
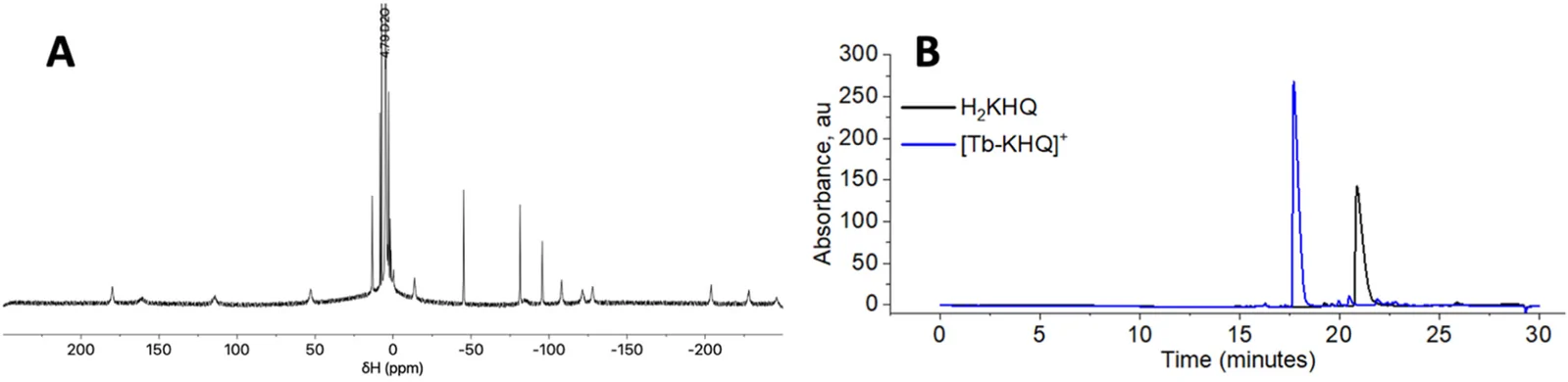
(A) ^1^H NMR spectrum of [Tb-KHQ]^+^ (D_2_O, 400 MHz, 298 K). (B) HPLC chromatogram of H_2_KHQ and [Tb-KHQ]^+^ (1 mM in NH_4_OAc, pH 6.5).

To investigate the metal chelating abilities of H_2_KHQ, the non-radioactive [Tb-KHQ]^+^ complex was synthesised by mixing H_2_KHQ and TbCl_3_·6H_2_O (1.2 eq.) in H_2_O (pH 4–5) at room temperature for 30 min. The pure complex was isolated by C18 reverse-phase chromatography as a yellow solid in 65% yield. The ^1^H NMR spectrum shows no evidence of unchelated H_2_KHQ ligand and contains characteristic paramagnetic NMR shifts of Tb^3+^, with signals observed between −200 and +250 ppm (Fig. 2A).^51^ LC-MS (Fig. S26) and HR-ESI-MS (Fig. S27) detected a molecular ion peak corresponding to the stoichiometry of [Tb-KHQ]^+^ at *m*/*z* 733.2076 (for [C_32_H_38_N_4_TbO_6_]^+^ calcd = 733.2039). HPLC confirmed the presence of a single [Tb-KHQ]^+^ species in solution, with a retention time distinct to that of unchelated H_2_KHQ ligand (Fig. 2B).

**Fig. 3:**
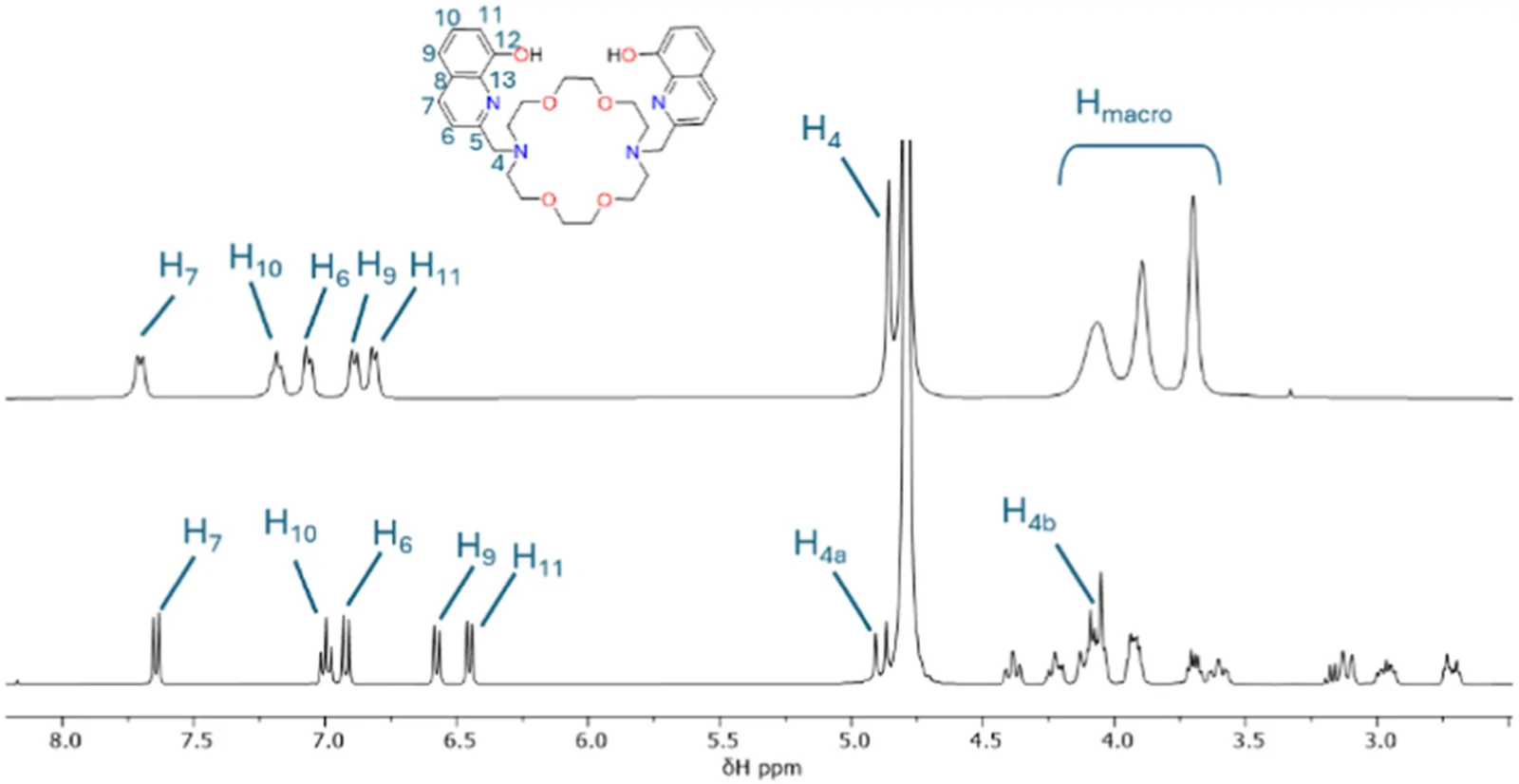
Expanded ^1^H NMR spectrum of H_2_KHQ (top) and [La-KHQ]^+^ (bottom). (D_2_O, pD 6.5, 400 MHz, 298 K). The macrocyclic protons are labelled as *H*_macro_.

The diamagnetic [La-KHQ]^+^ complex was also prepared: H_2_KHQ and LaCl_3_·7H_2_O (1.2 eq.) were mixed in D_2_O (pD 6.5) at room temperature for 30 min. The ^1^H NMR spectrum (Fig. 3) indicated a single symmetric species present: only five resonances in the aromatic region were observed, corresponding to the five distinct proton environments on the 8-hydroxyquinoline arms. Notably, large geminal ^1^H–^1^H coupling constants (^2^*J*_HH_ > 10 Hz) were observed for all macrocyclic CH_2_ protons. This is consistent with metal binding, leading to chemically inequivalent geminal proton environments. In the ^13^C{^1^H} NMR spectrum (Fig. S12), sixteen ^13^C signals were detected, further supporting the presence of a 2-fold symmetric species. Upon La^3+^ binding, an increase in the chemical shift of aromatic 8-hydroxyquinoline ^13^C resonances was observed, relative to the unchelated H_2_KHQ ligand. The chemical equivalence of ^1^H and ^13^C atoms of both 8-hydroxyquinoline motifs is similar to analagous complexes (La^3+^-macrodipa, La^3+^-macropa), in which 2-fold symmetry was also observed in solution by ^1^H and ^13^C NMR spectroscopy.^20,21,52^

To further probe the solution-state chemistry of [La-KHQ]^+^, a mixture of H_2_KHQ and LaCl_3_·7H_2_O (1.2 eq.) in D_2_O was monitored *via*^1^H NMR spectroscopy from pD 2–13 (Fig. S15). The ^1^H NMR spectrum of H_2_KHQ ligand did not change significantly with pD. Binding of La^3+^ to H_2_KHQ was not observed at low pD but was seen at pD 6.5, with only a single species noted. With increasing pD, the chemical shifts of [La-KHQ]^+^ did not change significantly.

Lastly, the formation of [Lu-KHQ]^+^ complex was also explored *via*^1^H NMR studies (Fig. S18–S20). H_2_KHQ and LuCl_3_·6H_2_O (1.2 eq.) were reacted in D_2_O, and ^1^H NMR spectra were obtained over pD 2–9. At low pD, no complex formation was detected whereas at pD 6, H_2_KHQ ligand and a [Lu-KHQ]^+^ species were observed in a 1 : 2 ratio. At pD 9, full conversion to a [Lu-KHQ]^+^ metal complex was observed. Similarly to [La-KHQ]^+^, the Lu^3+^ complex showed 2-fold symmetry, as evidenced by a single set of aromatic protons.

**Fig. 4:**
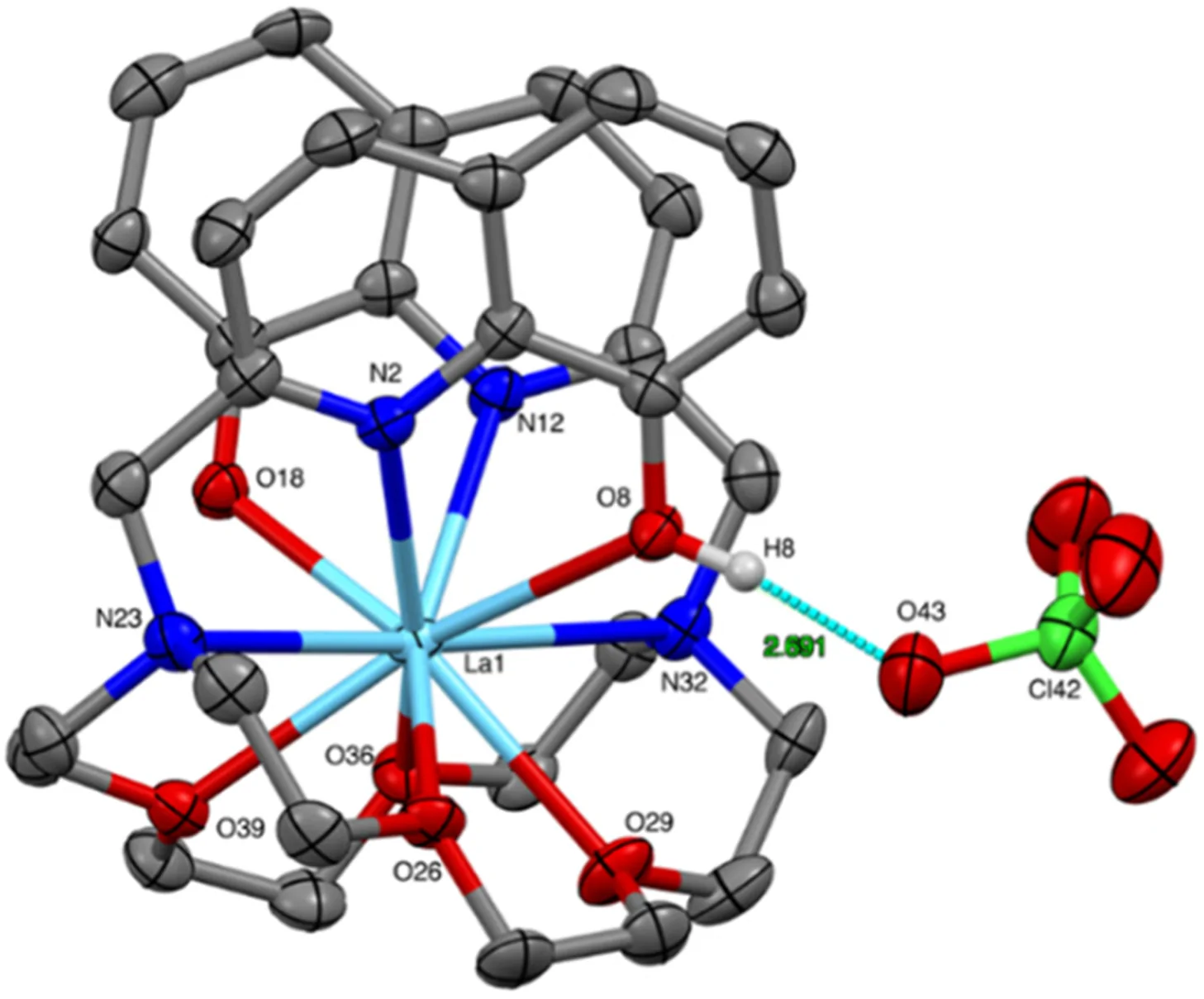
The structure of [La-HKHQ](ClO_4_)_2_ (50% probability ellipsoids). H atoms and second perchlorate anion omitted for clarity. H-bonding contacts in teal and interatomic distances in green.

To explore the solid-state structure of the [La-KHQ]^+^ complex, it was re-synthesised by mixing H_2_KHQ with La(ClO_4_)_3_ (1.2 eq.) in MeOH at room temperature for 30 min. Single crystals of [La-HKHQ](ClO_4_)_2_ were obtained by vapour diffusion of pentane into a solution of [La-HKHQ](ClO_4_)_2_ in EtOH. Single crystal X-ray diffraction analysis revealed the crystalline material to be [La-HKHQ](ClO_4_)_2_ (Fig. 4). The La^3+^ metal centre sits in a 10-coordinate environment, with all nitrogen and oxygen donor atoms bound to the metal centre. The complex adopted a *syn*-conformation, where the 8-hydroxyquinoline arms were both coordinated to the metal centre on the same face relative to the macrocyclic ring. The *syn*-conformation of the ligand implies the presence of two helices, one for the pendant arms (absolute configuration Δ or Λ) and one for the six five-membered chelate rings formed (absolute configuration δ or λ).^53,54^ The crystal structure reveals the most stable isomer to be Δ(δλλ)(δλδ), present as part of a racemate. One of the hydroxyl groups remained protonated, forming a hydrogen bond to a second perchlorate counter-anion, and a long La–O8 bond (2.673(3) Å). The non-protonated oxygen atom in the 8-hydroxyquinoline arms formed a strong La–O18 bond (2.365(3) Å). In [La-HKHQ]^2+^, La–N12 (2.658(4) Å), La–N2 (2.655(3) Å), La–N23 (2.847(4) Å) were all relatively shorter than the La–N bonds in [La(Hmacropa)(H_2_O)], suggesting that the 8-hydroxyquinoline arms provide a basis for stronger binding in comparison to the picolinate motif.^20^ The solid-state structure was consistent with the ^1^H NMR solution-state data.

### Lanthanide complexes thermodynamic stability

Potentiometric titrations were employed to obtain ligand protonation constants (*K*_a_) and complex stability constants (*K*_LnL_) (defined in eqn (S4) and (S5) respectively).

As shown in Table 1, the first and second H_2_KHQ protonation constants (log *K*_H1_ and log *K*_H2_) correspond to the hydroxyl groups of the two 8-hydroxyquinoline arms. In free 8-hydroxyquinoline, the hydroxyl groups have a p*K*_a_ of 9.9.^55^ The differences in protonation constants between the two 8-hydroxyquinoline substituents on H_2_KHQ is likely a result of complex intramolecular hydrogen bonding patterns in the unchelated ligand, such as those between the O–H and the macrocyclic nitrogen atoms (Fig. S9).^56^ The third and fourth protonation constants (log *K*_H3_ and log *K*_H4_) correspond to the macrocyclic tertiary amine atoms, while the fifth and sixth protonation constants (log *K*_H5_ and log *K*_H6_) correspond to the nitrogen atoms of the 8-hydroxyquinoline motifs.

**Table 1:** Protonation constants (log *K*_a_) and stability constants (log *K*_LnL_) obtained by potentiometric titrations

	H_2_KHQ^a^	Macropa^24,57^	Py_2_-macrodipa^23^	Py-macrodipa^22^	Macrodipa^21^
Log *K*_a1_	10.40(2)	7.41	7.58(4)	7.20	7.79
Log *K*_a2_	7.70(2)	6.85	6.48(1)	6.54	7.04
Log *K*_a3_	4.03(4)	3.32	3.52(3)	3.17	3.18
Log *K*_a4_	3.74(3)	2.36	2.60(5)	2.31	2.14
Log *K*_a5_	3.10(5)	1.69	2.10(11)	—	—
Log *K*_a6_	3.08(4)	—	—	—	—

Log *K*_LaL_	12.10(8)	14.99	16.68(8)	14.31(6)	12.19(2)
Log *K*_TbL_	11.05(2)	11.79	14.76(6)	11.95(3)	9.68(1)
Log *K*_LuL_	8.58(5)	8.25	11.90(3)	11.54(2)	10.64(4)
Log *K*_LaHL_	—	2.28	—	—	—
Log *K*_LuLH−1_	2.46(1)	—	—	—	—

*p*_La_	9.47	15.58	17.20	15.03	12.49
*p*_Tb_	8.54	12.38	15.28	12.62	9.98
*p*_Lu_	7.50	8.84	12.42	12.26	10.94

a This work: 0.1 M NaCl, 25 °C. Ligand concentration 0.018 mmol. pH range used 3–10.5. Three repeats. The values in the parentheses are one standard deviation of the last significant figure. pM is the negative logarithm of the free metal concentration in equilibrium with complexed and free ligand, at a fixed pH 7.4.

**Fig. 5:**
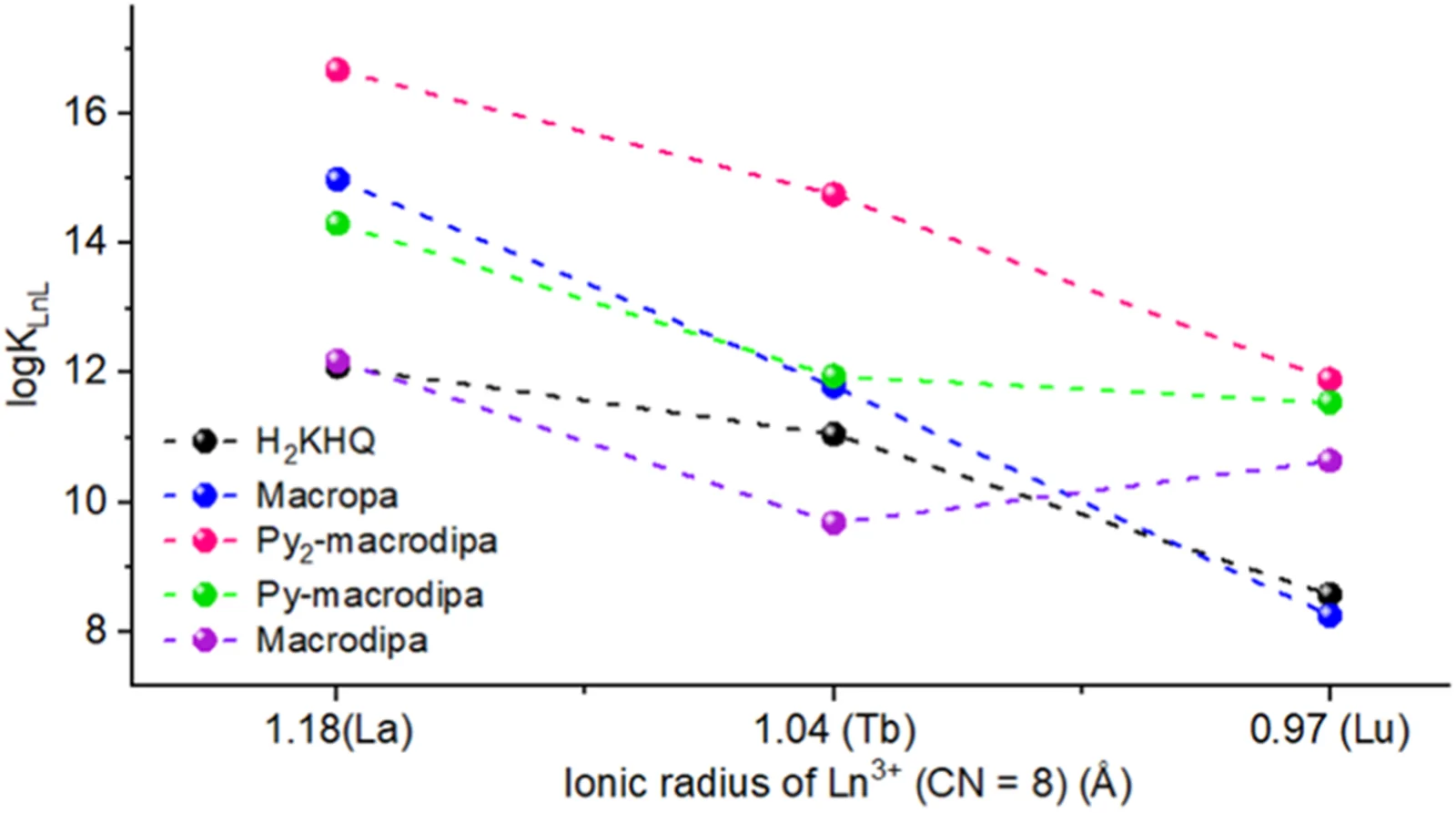
Stability constants of [Ln-KHQ]^+^ (Ln = La, Tb, Lu) plotted *versus* Ln^3+^ ionic radii.

The log *K*_LnL_ values for H_2_KHQ and similar macrocyclic chelators such as macropa and py_2_-macrodipa were plotted against the ionic radii of key Ln^3+^ ions (Fig. 5).^21–23,57,58^ The affinity of H_2_KHQ for trivalent cationic lanthanide metal ions decreases as the ionic radius decreases. Importantly, the log *K*_LnL_ (L = KHQ^2−^, Ln = Tb^3+^, Lu^3+^) values were comparable to those of macropa, highlighting that substituting picolinic acid groups for hydroxyquinoline groups does not have a marked effect on the binding affinity of this class of chelators. Indeed, the log *K*_LnL_ values for Ln^3+^ complexes of H_2_KHQ are lower than those of many newer chelators (such as py_2_-macrodipa).

However, to compare ligands with different basicities, the relevant parameter pM, is used as an indicator of affinity (Table 1). The pM value is the negative logarithm of the free metal concentration in equilibrium with complexed and free ligand, at a fixed pH 7.4. Analysis of the pM(Ln(iii)) values emphasizes that with H_2_KHQ at pH 7.4, more Ln(iii) ions are present in solution, in comparison to other chelators.

**Fig. 6:**
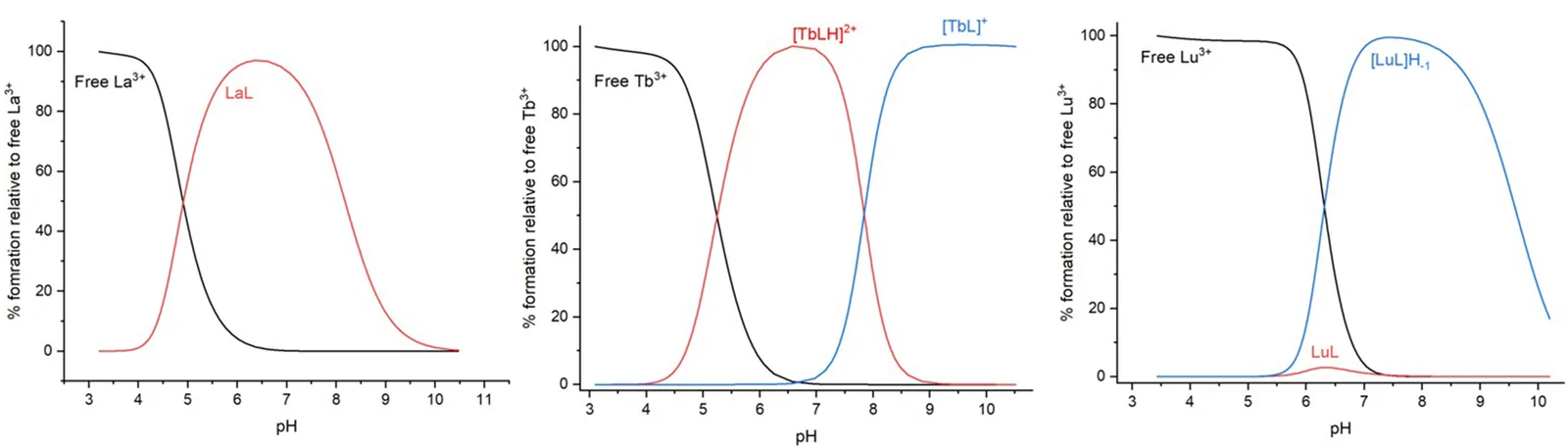
Representative species distribution for [Ln-KHQ]^+^ (Ln = La, Tb, Lu) modelled in HySS. H_2_KHQ*c* = 0.018 mmol, Ln^3+^*c* = 0.018 mmol. Initial volume *V* = 30 mL. Data fitting and speciation distribution over the pH range shown. L represents fully deprotonated ligand, KHQ^2−^.

Speciation plots (Fig. 6) indicate that under the conditions studied here, all available H_2_KHQ ligand molecules were bound to La^3+^ at pH ∼6, Tb^3+^ at pH ∼6.5 and Lu^3+^ at pH ∼7.5. This was also consistent with ^1^H NMR studies which indicated that a higher pH was needed to achieve quantitative coordination of Lu^3+^ by H_2_KHQ, as compared to the analogous La^3+^ complex. The distribution of both La^3+^ and Tb^3+^ species supports the formation of one major species at ambient pH, consistent with ^1^H NMR spectroscopic studies. Significantly, the Lu^3+^ speciation plot indicates that the major species forming between Lu^3+^ and H_2_KHQ at above pH 6.5 is LuL_H−1_. This most likely corresponds to a species where a water molecule is interacting with the complex. Attempts to elucidate the solid-state structure of [Lu-KHQ]^+^*via* crystals were unsuccessful.

### DFT calculations

**Fig. 7:**
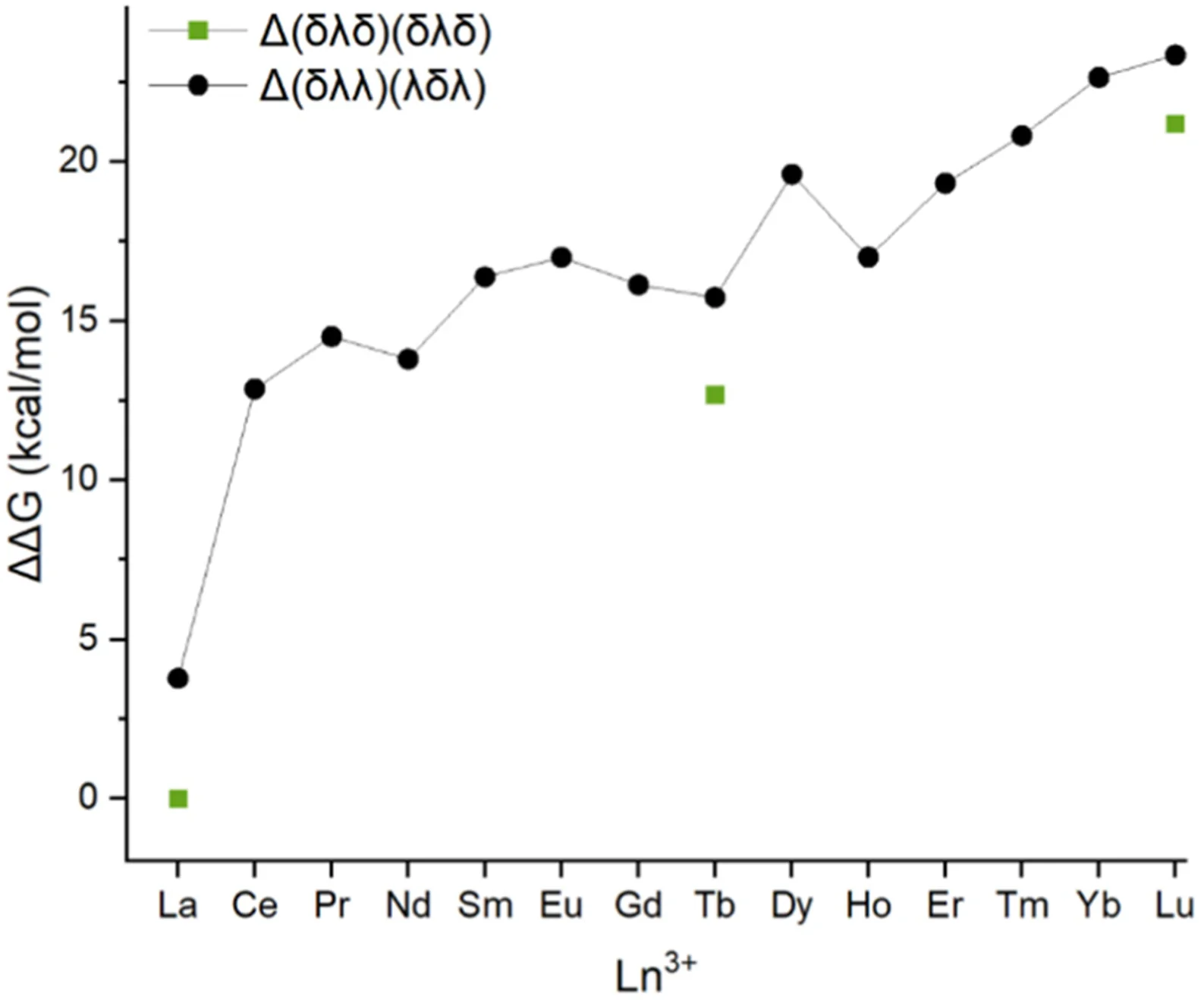
DFT-computed relative Gibbs free energies (ΔΔ*G*) for the transmetallation reaction: values are calculated as: 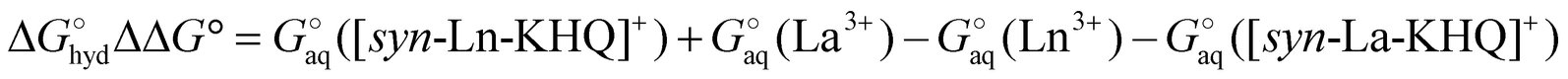.

DFT is a useful tool for exploring the coordination chemistry of Ln^3+^ with macrocyclic chelators and has been used to better understand the origin of size selectivity in macropa and second-generation analogues.^21–23^ Herein, we explored the binding of H_2_KHQ across the lanthanide series (La^3+^–Lu^3+^) to gain insight into the size selectivity of H_2_KHQ complexation. Initially, geometries were taken from the crystal structure of *syn*[La-HKHQ]^2+^ with removal of H8 to model the overall ‘+1’ species. Different conformational arrangements were modelled with the 8-hydroxyquinoline arms in either a *syn* or *anti* arrangement around the metal centre (whilst retaining symmetry). Across the lanthanide series, the *syn* conformation was favoured over the *anti*-conformation (Fig. S30). The relative Gibbs free energy (ΔΔ*G*) for the transmetallation reaction ([LaKHQ]^+^ + Ln^3+^ → [LnKHQ]^+^ + La^3+^) was calculated. The results show a thermodynamic preference for the larger Ln^3+^ ions (Fig. 7). This calculated trend is in good qualitative agreement with experimental data (ΔΔ*G*_exp_ = −2.303RT log(*K*_LnL_ − *K*_LaL_), ΔΔ*G*_Tb–La_ = +1.43 kcal mol^−1^, ΔΔ*G*_Tb–La_ = +4.80 kcal mol^−1^) which shows decreasing thermodynamic stability across the series. The quantitative discrepancy is attributed to the over estimation of gas-phase Ln^3+^ ion energies in our model, which does not include explicit solvation effects. The destabilisation evidently results from a greater degree of ligand strain in the macrocyclic framework to facilitate the coordination of the oxygen atoms to the smaller Ln^3+^ ions (Fig. S34). Furthermore, H_2_KHQ did not exhibit the conformational toggle that is predominant in macrodipa and analogues.^21–23^

Structural analysis revealed a coordination number of 10 for all Ln^3+^, involving four nitrogen atoms and six oxygen atoms in coordination to the metal centre (Fig. S31). Across the series, the hapticity of the KHQ ligand was η^10^, with the metal ion located above the plane of the macrocyclic ring system, enclosed by both 8-hydroxyquinoline units. Additionally, the presence of a water molecule in the first coordination sphere was modelled, however the resulting coordination number of 11 was less favoured across the series (Table S2, 
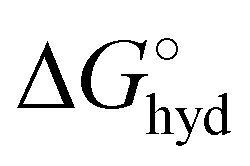
).

Analysis of the structure of [La-HKHQ]^2+^ reveals that there are 16 possible conformations (8 enantiomeric pairs of diastereoisomers) with *C*_2_ symmetry.^24^ To better explore the in-solution behaviour, an in-depth conformational screen of 8 different diastereomers was carried out for La^3+^, Tb^3+^ and Lu^3+^. As shown in Fig. S32, our calculations predict that the Δ(δλδ)(δλδ) conformation is the lowest energy form in aqueous solution across the three Ln^3+^ ions tested, and is lower in energy by *ca*. 1.9 kcal mol^−1^ than the solid-state conformation observed for [La-HKHQ]^2+^. The relative energies of the different conformations in aqueous solution are given in Table S3 and highlight the multiple *C*_2_-symmetric conformational modes that likely exist in equilibrium in solution. This result is consistent with the NMR solution-state studies on [La-KHQ]^+^ where a complex with *C*_2_ symmetry was observed.

To explore the effects of the nature of the pendant arm on the stability of these complexes a parallel conformational screen of macropa complexes (for La^3+^, Tb^3+^ and Lu^3+^) was carried out, where our model also favoured the Δ(δλδ)(δλδ) conformation (see Fig. S33). Analysis of gas-phase Gibbs free binding energies and ligand strain was performed to compare the influence of the pendant arms of macropa and KHQ on ligand preorganisation and metal binding. The results show that whilst ligand strain generally increases with decreasing ionic radius for both ligands, the effect is much less pronounced for macropa (Fig. S34). Additionally, our calculations suggest that macropa provides a consistently greater, albeit modest, metal–ligand stabilisation for La^3+^, Tb^3+^ and Lu^3+^ (Table S4). Whilst experiments show the reverse trend for Lu^3+^, the differences are small enough that calculated values are likely within error. Together with experimental results, it appears that the greater rigidity of the pendant arms in KHQ forces a higher degree of overall ligand strain in the metal-bound conformation of the macrocycle.

### Phosphorescence spectroscopy

**Fig. 8:**
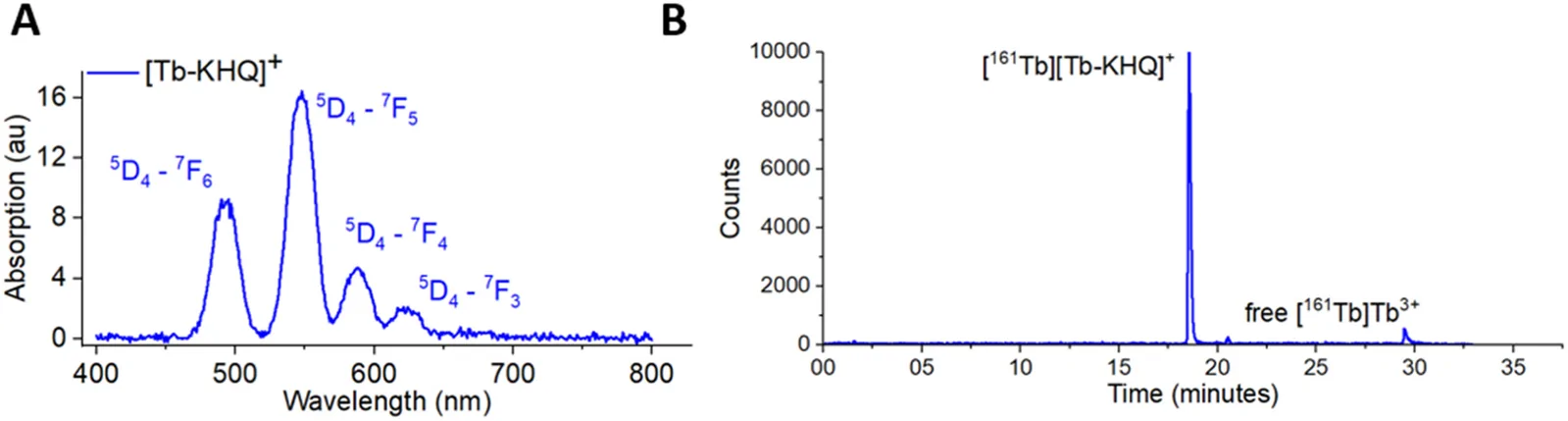
(A) Phosphorescence spectrum for [Tb-KHQ]^+^ (H_2_O, 20 μM, 20 nm slits, 0.1 ms delay). (B) RadioHPLC of H_2_KHQ (1 mM) with [^161^Tb]Tb^3+^ (2.5 MBq, 1 mM HCl) after 60 min at 90 °C. RCY = 92%. [^161^Tb][Tb-KHQ]^+^ retention time = 18.3 min.

The pendant 8-hydroxyquinoline motifs possess useful photophysical properties and can potentially act as antennae to exploit the luminescence emission properties of Tb^3+^. Luminescence studies were carried out to ascertain whether the [Tb-KHQ]^+^ complex exhibited characteristic Tb phosphorescent emissions. Indeed, sharp emission peaks at 518, 565, 583 and 621 nm were observed for [Tb-KHQ]^+^*via* the antenna effect, following excitation of the ligand at 242 nm (Fig. 8A). These are characteristic of the ^5^D_4_ → ^7^F_*n*_ transitions (*n* = 6, 5, 4, 3). This preliminary experiment highlights the potential of 8-hydroxyquinoline based ligands to be used for optical imaging, with further studies needed to optimise ligand design for efficient sensitization.

### Radiolabelling experiments

Finally, [^161^Tb]Tb^3+^ radiolabelling experiments were undertaken, with [^161^Tb]TbCl_3_ provided by the Paul Scherrer Institute. Solutions of H_2_KHQ (1 mM) and [^161^Tb]Tb^3+^ (2.5 MBq, 1 mM HCl) in NH_4_OAc (20 mM) at pH 8.5 were reacted at 25 °C. After 20 min and 180 min, the reactions were analysed by reverse-phase radio-HPLC (Fig. S41–43), which indicated formation of [^161^Tb][Tb-KHQ]^+^ with a retention time (*R*_*t*_) of 18.33 min. We observed that unreacted [^161^Tb]Tb^3+^ was initially retained on the column, presumably as a colloidal species, similar to prior radiochemical observations.^59^ However, upon switching to an aqueous mobile phase at the end of analysis, free [^161^Tb]Tb^3+^ eluted, appearing at >29 min, enabling quantification of radiochemical yield (RCY). Preliminary radiolabelling studies show that at 25 °C, H_2_KHQ was radiolabelled in 33% RCY after 20 min, which increased to 68% after 180 min. Upon heating to 90 °C for 60 min, a high RCY of 92% was achieved (Fig. 8B). In comparison, macropa radiolabelling with [^161^Tb]Tb^3+^ (5 MBq, 2 mM HCl) was achieved at 62 and 55% RCY respectively at room temperature and 90 °C after 30 minutes (Fig. S44 and S45).

## Conclusions

The coordination of H_2_KHQ with Ln^3+^ ions has been interrogated *via* X-ray crystallography, NMR spectroscopy, DFT calculations, analytical chromatography and potentiometric titrations. H_2_KHQ exhibited a higher binding affinity towards larger Ln^3+^ ions compared to the smaller ions. DFT calculations corroborate the experimental evidence: [Ln-KHQ]^+^ complexes demonstrated decreasing thermodynamic stability as ionic radii decrease across the lanthanide series. In solid-state studies, XRD analysis of [La-KHQ]^+^ showed that the La^3+^ metal ion adopts a 10-coordinate conformation with the 8-hydroxyquinoline arms binding in a *syn* orientation relative to the macrocycle. This was consistent with solution-state NMR studies, that suggest a 2-fold symmetric complex present. The optical properties of [Tb-KHQ]^+^ were investigated by ligand sensitization at 242 nm, and characteristic phosphorescence emission peaks were observed. It has been noted that in order to effectively exploit the capabilities of 8-HQ moieties as antennae, optimised ligand analogues can be designed for increased sensitization and signal enhancement. The promising chelation properties seen with La^3+^ and Tb^3+^ prompted us to explore the chelation of KHQ with [^161^Tb]Tb^3+^. Preliminary radiolabelling corroborated the ability of H_2_KHQ to coordinate [^161^Tb]Tb^3+^ in high radiochemical yields (92%). Further in-depth radiolabelling studies are required to explore concentration, time, pH and temperature dependencies as well as maximum molar activity. Complex stability and inertness for *in vivo* applications shall be adequately explored in future work. Still, the combination of radioactive properties with photophysical properties could enable the development of a dual-modal Tb^3+^ probe for future medical applications.^60,61^

## Author contributions

C. S. synthesised the compounds and performed the analyses. The radiolabelling was performed with the help of B. E.O. and M. T. M. DFT and X-ray crystallography was carried out by R. K. B. Potentiometric titrations were performed by C. S. and C. R. M. T. M. and N. J. L. supervised the project, and all the authors contributed to the writing of the manuscript.

## Conflicts of interest

There are no conflicts to declare.

## Supplementary Material

DT-055-D5DT03015C-s001

DT-055-D5DT03015C-s002

## Data Availability

The data supporting this article have been included as part of the supplementary information (SI). Supplementary information is available. See DOI: https://doi.org/10.1039/d5dt03015c. CCDC 2435038 and 2435039 contain the supplementary crystallographic data for this paper.^62*a*,*b*^
