# Supplementary material for: Hydroxyquinoline-functionalised aza-crown macrocycles for lanthanide coordination
Source: Dalton Trans. 2026 Jan 28;55(8):3413–21. doi: 10.1039/d5dt03015c (PMC13591454; doi:10.1039/d5dt03015c)
Supplement: Supplementary file 1 [file DT-055-D5DT03015C-s001.pdf]

Supplementary Information (SI) for Dalton Transactions.
This journal is © The Royal Society of Chemistry 2026

# Supplementary Information

## Hydroxyquinoline-functionalised Aza-crown Macrocycles for Lanthanide Coordination

Christina Siakalli,[a,b] Bradley E. Osborne,[a,b], Ryan K. Brown,[a] Claudia Rocco,[c] Dominik Weiss,[c] Enrique V. García-España,[d] Pascal V. Grundler[e], Anzhelika N. Moiseeva[e], Zeynep Talip[f], Nicholas P. van der Meulen[e,f], Michelle T. Ma,*[b] and Nicholas J. Long *[a]

[a.] Department of Chemistry, Imperial College London, Molecular Sciences Research Hub, White City Campus, London, W12 0BZ, UK.

[b.] School of Biomedical Engineering and Imaging Sciences, King's College London, St Thomas' Hospital, London, SE1 7EH, UK.

[c.] Department of Earth Science and Engineering, Imperial College London, South Kensington Campus, London, SW7 2BP, UK.

[d] ICMol, Departament de Química Inorgànica, Universitat de València, C/Catedrático José Beltrán 2, Paterna 46980, Spain.

[e] Center for Radiopharmaceutical Sciences, PSI Center for Life Sciences, 5232 Villigen-PSI, Switzerland

[f] Laboratory of Radiochemistry, PSI Center for Nuclear Engineering and Science, 5232 Villigen-PSI, Switzerland

Table of Contents

1. ***General Considerations*** ..... 2
2. ***Synthesis*** ..... 3
3. ***X-ray Crystallography*** ..... 8
4. ***NMR spectroscopy of $Ln^{3+}$ complexes*** ..... 10
5. ***LC-MS and HPLC experiments*** ..... 16
6. ***DFT*** ..... 21
7. ***Potentiometric titrations*** ..... 25
8. ***UV-Vis Spectroscopy and Phosphorescence*** ..... 28
9. ***Radiolabelling*** ..... 29
10. ***References*** ..... 33
11. ***XYZ Coordinates*** ..... 35

# 1. General Considerations

**Reagents:** All measurements were carried out at atmospheric pressure with stirring unless otherwise stated. No special precautions were taken to exclude air or moisture during reactions, workups or purifications unless otherwise stated. All solvents were obtained from commercial sources and used without further purifications. The chelator **$H_2$KHQ** (**3**) was prepared following **Scheme 1**. 2-Bromomethyl-8-hydroxyquinoline (**2**) was prepared as previously described. [1,2] Macropa was prepared as previously described.[3]

**Instruments and characterisation:** TLC was performed on Kiesegel 60 F 254 plates (Merck) and visualisation achieved under UV fluorescence (254 and 366 nm). Flash silica column chromatography was performed on a Biotage Isolera™ 4 System advanced automated flash purification unit using 10 g, 25 g, or 50 g Sfär Duo cartridges, unless otherwise told. Reverse-phase flash chromatography was performed using the same Biotage Isolera™ 4 System with Biotage® Sfär Ultra C18 cartridges (12 g). $^1$H, $^{13}$C{$^1$H} and 2D NMR spectra were recorded on a Bruger AV-400, AV-500, or DRX-400 spectrometer. Chemical shifts are reported in ppm and spectra are referenced to $D_2O$ at $\delta$ = 4.79 ppm ($^1$H); $CD_3OD$ at $\delta$ = 3.31 ($^1$H) and 49.2 ppm ($^{13}$C{$^1$H}); $CDCl_3$ at $\delta$ = 7.26 ($^1$H) and 77.2 ($^{13}$C{$^1$H}), DMSO-d6 at $\delta$ = 2.50 ($^1$H).[4] $^{13}$C{$^1$H} were assigned where possible using 2D correlation spectroscopy such as HMBC and HSQC. Spectra were processed and analysed using *MestReNova* (Mestrelab Research S.L.). software. Signal multiplicities are indicated as: s (singlet), d (doublet), t (triplet), q (quartet), dd (doublet of doublets), dt (doublet of triplets), ddd (doublet of doublets of doublets), ddt (doublet of doublets of triplets), ddq (doublet of doublets of quartets), m (multiplet); defined as all multi-peak signals where overlap or complex coupling of signals makes definitive descriptions of peaks difficult. Numbering of magnetically inequivalent environments is recorded where full assignment was achieved.

Analytical HPLC was performed on an Agilent 1260 Infinity II HPLC using a ZORBAX Eclipse XDB-C18 column (4.6 × 150 mm, 5 µm) in at 40 ˚C with a flowrate of 1 mL/min and UV-Vis spectroscopic detection at 254 nm. Mobile phase A of 2mM $NH_4OAc$ in $H_2O$ and mobile phase B of 5 % 2 mM $NH_4OAc$ in $H_2O$ in 95 % MeCN were used as shown below.

| Gradient, min | A, % | B, % |
|---|---|---|
| 0.00 | 95 | 5 |
| 5.00 | 95 | 5 |
| 25.00 | 5 | 95 |
| 27.00 | 5 | 95 |
| 28.00 | 95 | 5 |
| 30.00 | 95 | 5 |

Liquid chromatography – mass spectrometry (LC-MS) was performed on an Agilent InfinityLab LC/MSD XT unit on an Agilent Poroshell HPH C18 3.0x50 mm 2.7 µm column in either an acidic or basic gradient at 40 ˚C with a flowrate of 0.8 mL/min and UV-Vis spectroscopic detection at 254 nm. Acidic methods include mobile phase A of 0.1 % HCOOH in $H_2O$ and mobile phase B of 0.1 % HCOOH in 90:10 MeCN: $H_2O$. Basic methods include mobile phase A of 0.1 % $NH_4OH$ in $H_2O$ and mobile phase B of 0.1 % $NH_4OH$ in 90:10 MeCN: $H_2O$. Gradients are as shown below.

| Gradient, min | A, % | B, % |
|---|---|---|
| 0.00 | 95 | 5 |
| 7.00 | 0 | 100 |
| 8.00 | 0 | 100 |
| 8.10 | 95 | 5 |
| 10.00 | 95 | 5 |

High resolution ES+ mass spectrometry analyses were conducted by Dr. Malgorzata Puchnarewicz at the Mass Spectrometry Service, Imperial College London on a Waters LCT Premier (ES-ToF)/Acquity i-Class spectrometer. UV-visible absorption spectra were measured using an Agilent Technologies Cary 60 Spectrophotometer operating with WinUV software. The sample was held in a quartz cuvette with a path length of 1 cm. Absorbance spectra were collected using 20 µM solutions in $H_2O$, against a baseline of pure solvent in an optically matched cuvette with a scan rate of 300 nm $min^{-1}$ and a data interval of 0.5 nm. Extinction coefficients were calculated from the Beer Lambert Law ($A=\varepsilon cl$) where A = the absorbance at a particular wavelength, $\varepsilon$ is the extinction coefficient, c is the concentration, and l is the path length (width of the quartz cuvette, 1 cm). Phosphorescence spectra were recorded on an Agilent Technologies Carry Eclipse Fluorescence Spectrophotometer in phosphorescence mode, in quartz

cuvettes with a path length of 1 cm. Phosphorescence spectra were collected using 20 µM solutions in $H_2O$ with the excitation and emission slits set at 10 or 20 nm, a gate time of 0.1 ms and delay time of 0.1 ms.

# 2. Synthesis

## 2.1. Experimental details

Synthesis of 1.[1]

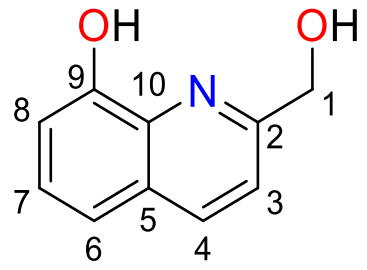


2-Formyl-8-hydroxyquinoline (1.00 g, 5.78 mmol) was dissolved in anhydrous MeOH (20 mL). $NaBH_4$ (0.29 g, 7.51 mmol, 1.3 eq.) was added slowly and the reaction stirred for 5 h at 20 ˚C. The reaction mixture went from dark yellow to pale yellow. The solvent was removed *in vacuo* and $H_2O$ (30 mL) was added. The resultant solution was neutralized using aqueous HCl (2 M) and then extracted with EtOAc (3 x 100 mL). The organic layer was dried over $Na_2SO_4$, filtered and concentrated *in vacuo*. 2-Hydroxymethyl-8-hydroxyquinoline was isolated as a yellow solid (1.01 g, 5.78 mmol, 100 %) and characterised by $^1$H NMR, matching literature.[2] **$^1$H NMR (400 MHz, $CDCl_3$) $\delta_H$ (ppm):** 8.15 (d, $^3J_{HH}$ = 8.5 Hz, 1H, $H_4$), 7.45 (t, $^3J_{HH}$ = 8.0 Hz, 1H, $H_6$), 7.41 – 7.31 (m, 2H,$H_{3,7}$), 7.21 (d, $^3J_{HH}$ = 7.5 Hz, 1H, $H_8$), 4.97 (s, 2H, $H_1$).

*Synthesis of 2.* [1]

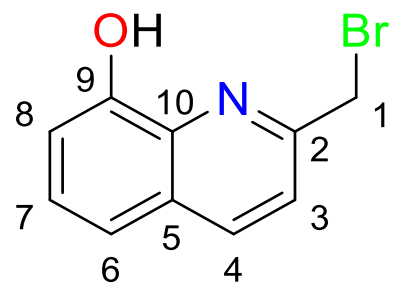


Concentrated HBr (48 % in $H_2O$, 25 mL) was added dropwise to 2-hydroxymethyl-8-hydroxyquinoline (compound **1**) (0.96 g, 5.49 mmol). The resulting orange solution was heated under reflux for 4 h and then cooled in an ice-water bath. Saturated $Na_2CO_3$ (aq.) was added until pH = 7. The product was extracted with DCM (3 x 100 mL) and the organic layer was dried over $Na_2SO_4$, filtered, and concentrated *in vacuo*. The product was isolated by silica column chromatography ($SiO_2$, hexane:EtOAc, 8:2) as a white solid (0.632 g, 2.67 mmol, 49 %). $^1$H NMR data matched literature.[1,2] **$^1$H NMR (400 MHz, $CDCl_3$) $\delta_H$ (ppm):** 8.10 (d, $^3J_{HH}$ = 8.5 Hz, 1H, $H_4$), 7.52 (d, $^3J_{HH}$ = 8.5 Hz, 1H, $H_6$), 7.48 – 7.39 (t, , $^3J_{HH}$ = 8.0, 8.0 Hz, 1H, $H_7$), 7.28 (d, $^3J_{HH}$ = 8.0 Hz, 1H, $H_3$), 7.18 (d, $^3J_{HH}$ = 7.5 Hz, 1H, $H_8$), 4.66 (d, $^3J_{HH}$ = 1.0 Hz, 2H, $H_1$).

*Synthesis of **$H_2$KHQ**.*

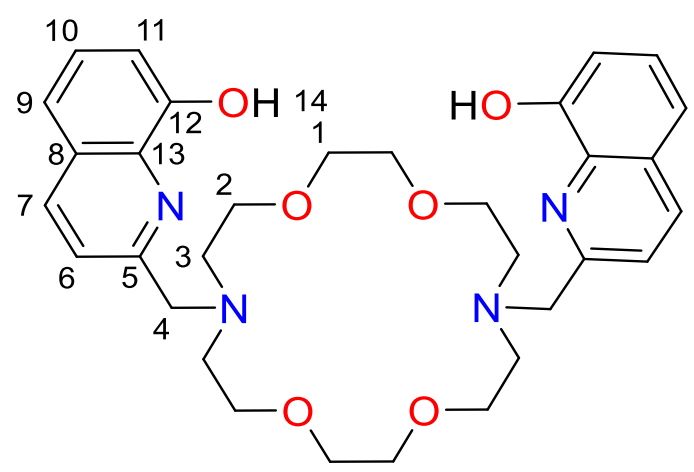


Under a $N_2$ atmosphere, 2-bromomethyl-8-hydroxyquinoline (compound **2**) (0.59 g, 2.5 mmol, 2.1 eq.), $K_{22}$ (0.30 g, 1.19 mmol) and $Na_2CO_3$ (0.76 g, 7.15 mmol, 6 eq.) were added to anhydrous MeCN (40 mL) and heated under reflux for 48 h. After 48 h, the reaction mixture was cooled to room temperature and filtered cold. The solvent was removed *in vacuo* isolating a yellow residue. The crude product was isolated by silica column chromatography ($SiO_2$, DCM:MeOH + 0.1 % TEA, 8:2) as a yellow solid. The pure product was then isolated following reverse phase chromatography (C18, 0 – 100 % MeCN+ 0.1 % FA in $H_2O$ + 0.1 % FA) as a white solid (0.19 g, 0.33 mmol, 27%). **$^1$H NMR (400 MHz, $CDCl_3$) $\delta_H$ (ppm):** 8.07 (d, $^3J_{HH}$ = 8.5 Hz, 2H, $H_7$), 7.66 (d, $^3J_{HH}$ = 8.5 Hz, 2H, $H_9$), 7.40 (t, $^3J_{HH}$ = 8.0 Hz, 2H, $H_{10}$), 7.28 (d, $^3J_{HH}$ = 8.5 Hz, 2H, $H_6$), 7.14 (d, $^3J_{HH}$ = 7.5 Hz, 2H, $H_{11}$), 4.02 (s, 4H, $H_4$), 3.67 (t, $^3J_{HH}$ = 6.0 Hz, 8H, $H_2$), 3.62 (s, 8H, $H_1$), 2.93 (t, $^3J_{HH}$ = 6.0 Hz, 8H, $H_3$). **$^{13}$C{$^1$H} NMR (101 MHz, $CDCl_3$) $\delta_C$ (ppm):** 158.9 ($C_5$), 152.2 ($C_{12}$),

137.5($C_{13}$), 136.4 ($C_7$), 127.6 ($C_8$), 127.3 ($C_{10}$) , 122.1 ($C_9$), 117.7 ($C_6$), 110.06 ($C_{11}$), 70.9 ($C_1$), 70.1 ($C_2$), 62.0 ($C_4$), 54.6 ($C_3$).
**HRMS (ES-TOF+):** *m/z* calcd. for $C_{32}H_{41}N_4O_6$ ($[M+H]^+$) at 577.3026; found as 577.3027. Analytical HPLC: $T_R$ = 20.8 min.

*Synthesis of* ***[Tb-KHQ]⁺***

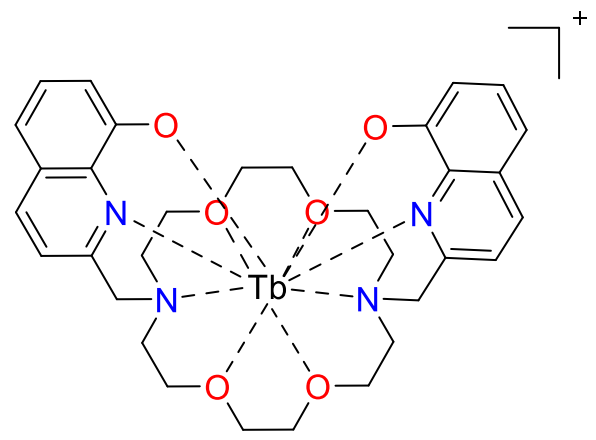

**$H_2$KHQ** (0.010 g, 0.02 mmol) was dissolved in $H_2O$ (pH 4 - 5) and $TbCl_3•6H_2O$ (0.009 g, 0.022 mmol, 1.2 eq.) was added. The reaction was left at RT for 30 mins. The product was isolated by reverse phase chromatography (C18, 0 – 100 % MeCN+ 0.1 % $NH_3$ (aq.) in $H_2O$ + 0.1 % $NH_3$ (aq.)). The product fractions were concentrated *in vacuo* to isolate yellow solid (0.0094 g, 0.013 mmol, 65 %). **$^1$H NMR (400 MHz, $D_2O$) $\delta_H$ (ppm):** 179.95, 114.27, 52.92, 13.34, 8.44, 7.31, 4.79, 3.73, 3.64, 3.38, 3.25, 2.88, 2.10, 1.95, 1.27, -0.34, -13.81, -45.14, -81.33, -95.67, -108.00, -121.37, -127.75, -203.83, -227.95. **HRMS(ES-TOF⁺)**: *m/z* calcd. for $C_{32}H_{38}N_4TbO_6$ $[M]^+$ at 733.2039; found as 733.2076.

## 2.2. Supplementary Data

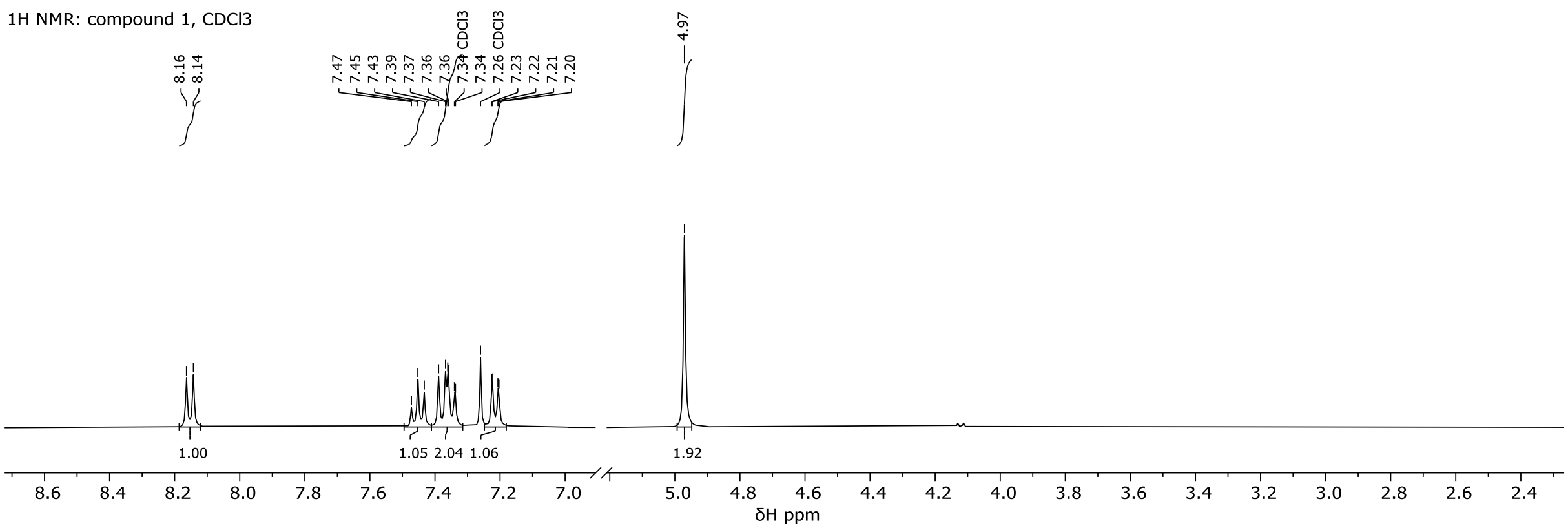


Figure S1. $^1$H NMR spectrum of compound **1** ($CDCl_3$, 400 MHz, 298 K). Matches literature data. [2]

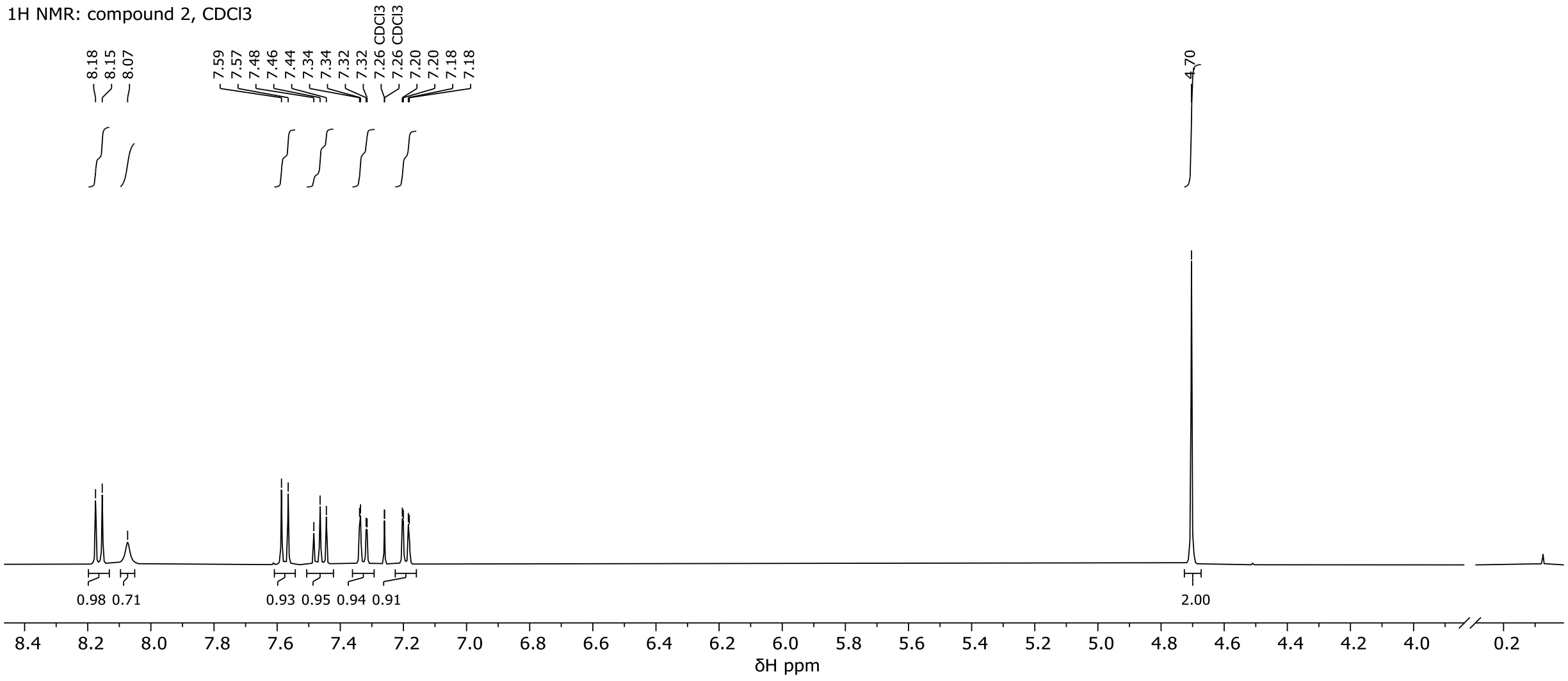


Figure S2. $^{1}H$ NMR spectrum of compound **2** ($CDCl_3$, 400 MHz, 298 K). Matches literature data. [2]

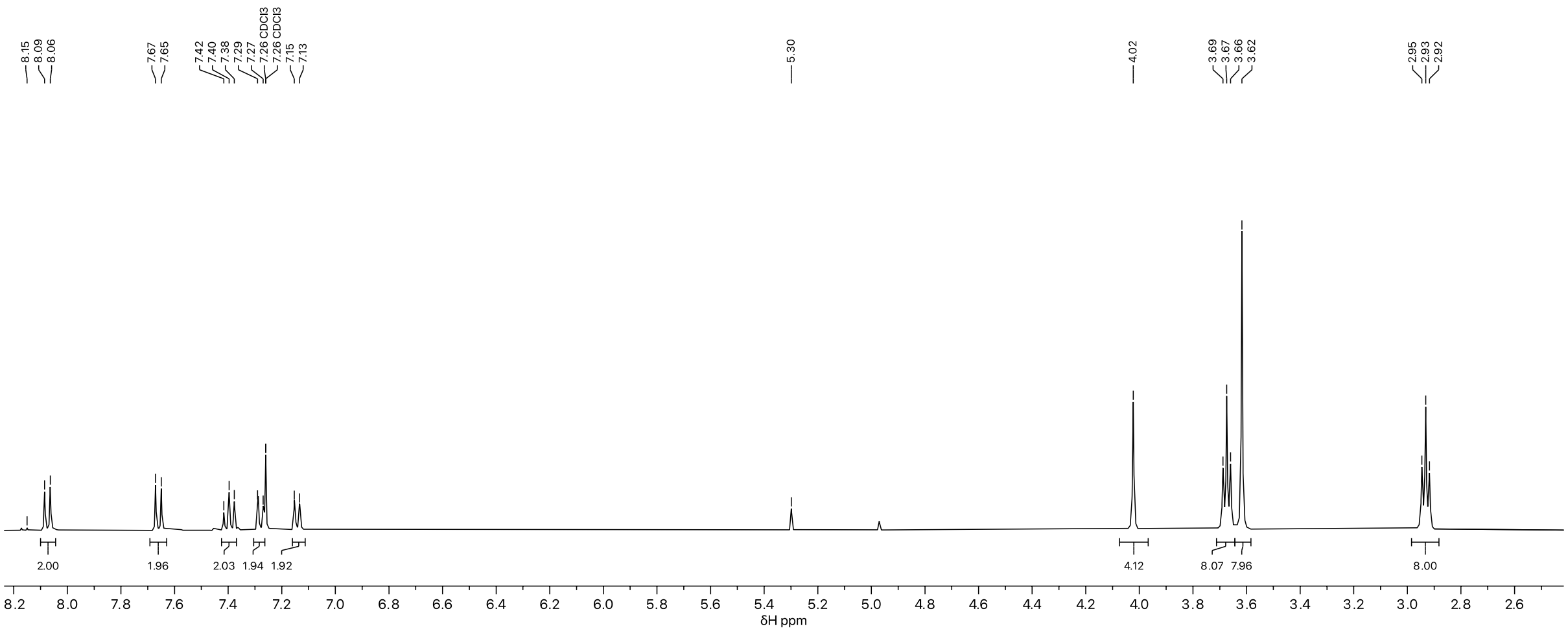


Figure S3. $^{1}H$ NMR spectrum of $\mathbf{H_2KHQ}$. ($CDCl_3$, 400 MHz, 298 K). The peak at 5.30 ppm is residual DCM.

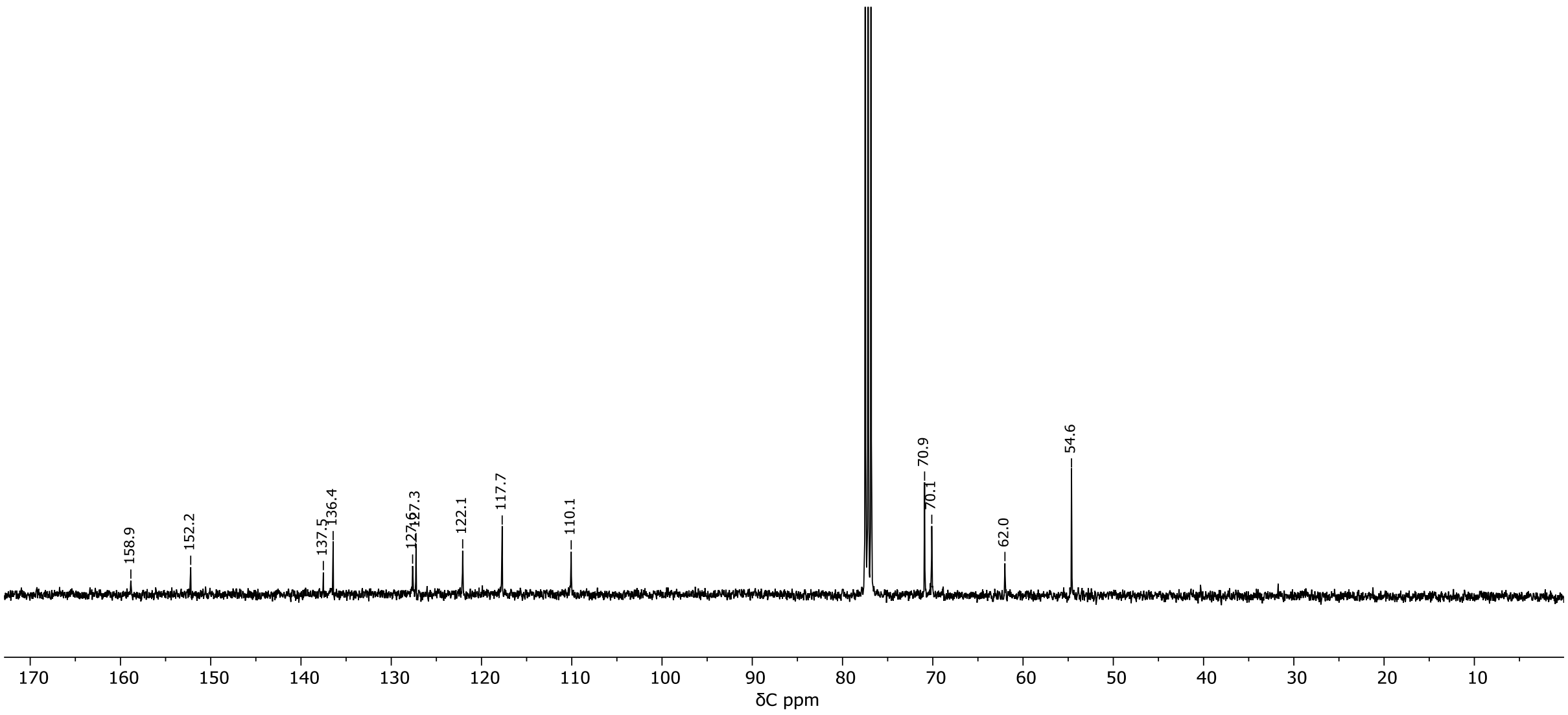


Figure S4. $^{13}C\{^{1}H\}$ NMR spectrum of $\mathbf{H_2KHQ}$ . ($CDCl_3$, 101 MHz, 298 K)

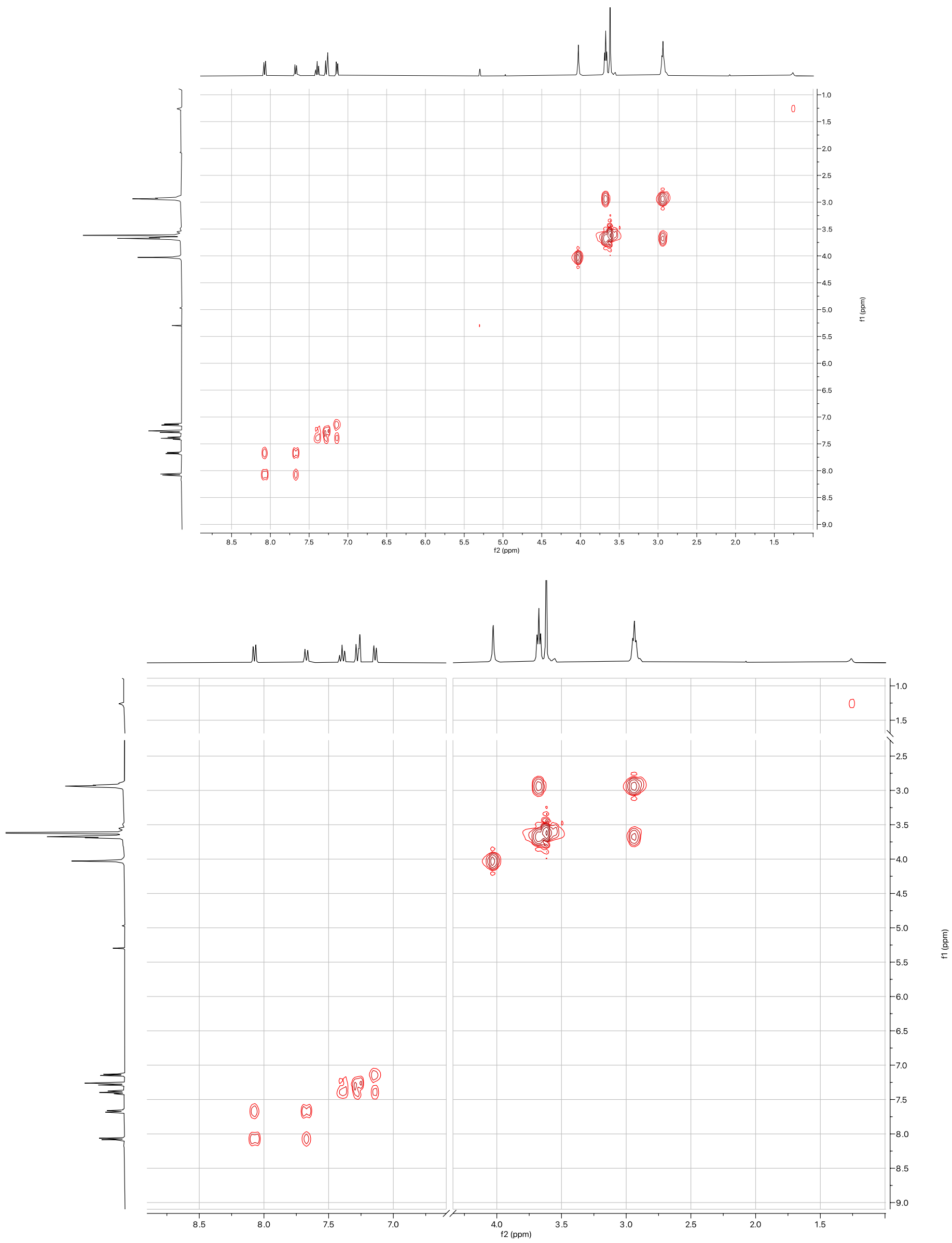


Figure S5. Full width (top) and expanded (bottom) $^1H$-$^1H$ COSY NMR spectrum of $\mathbf{H_2KHQ}$. ($CDCl_3$, 298 K)

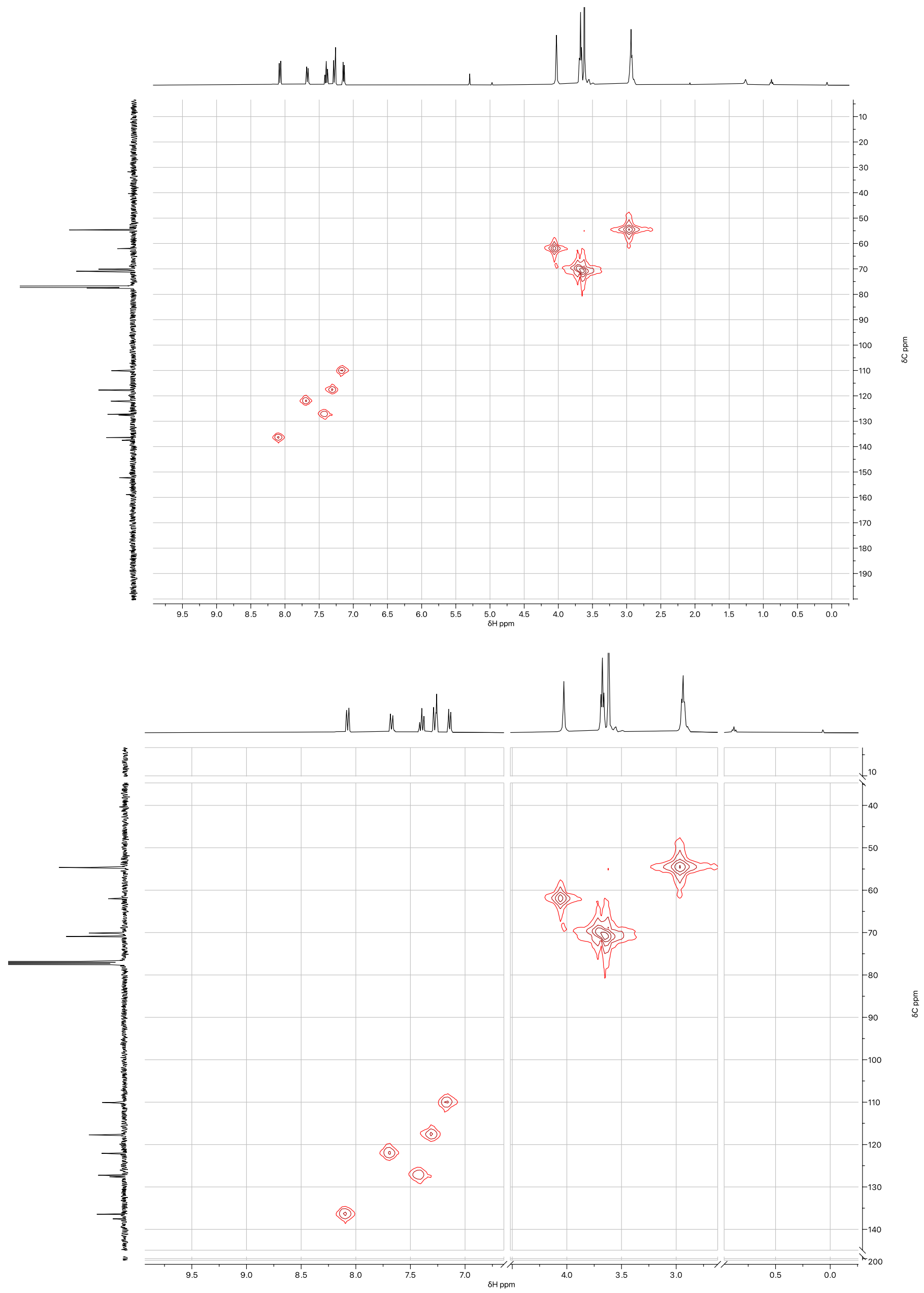


Figure S6. Full width (top) and expanded (bottom) $^{1}H$-$^{13}C$ HMQC NMR spectrum of **$H_2KHQ$**. ($CDCl_3$, 298 K).

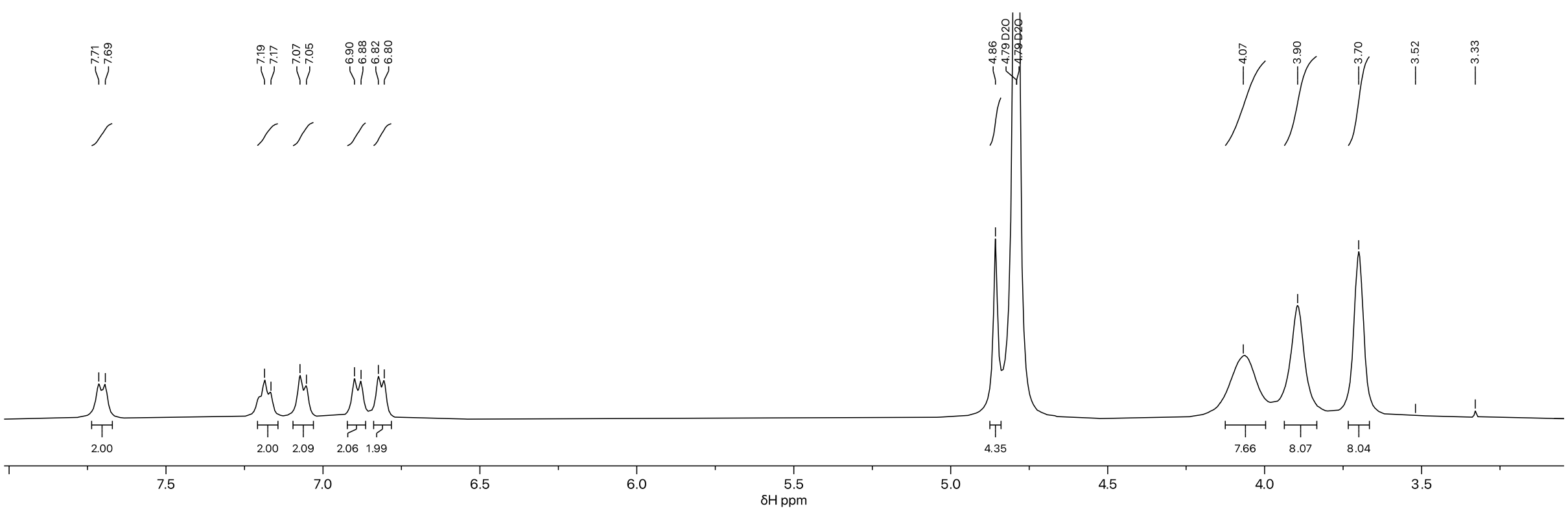


Figure S7. $^{1}H$ NMR spectrum of **$H_2KHQ$** . ($D_2O$, pD = 5, 400 MHz, 298 K).

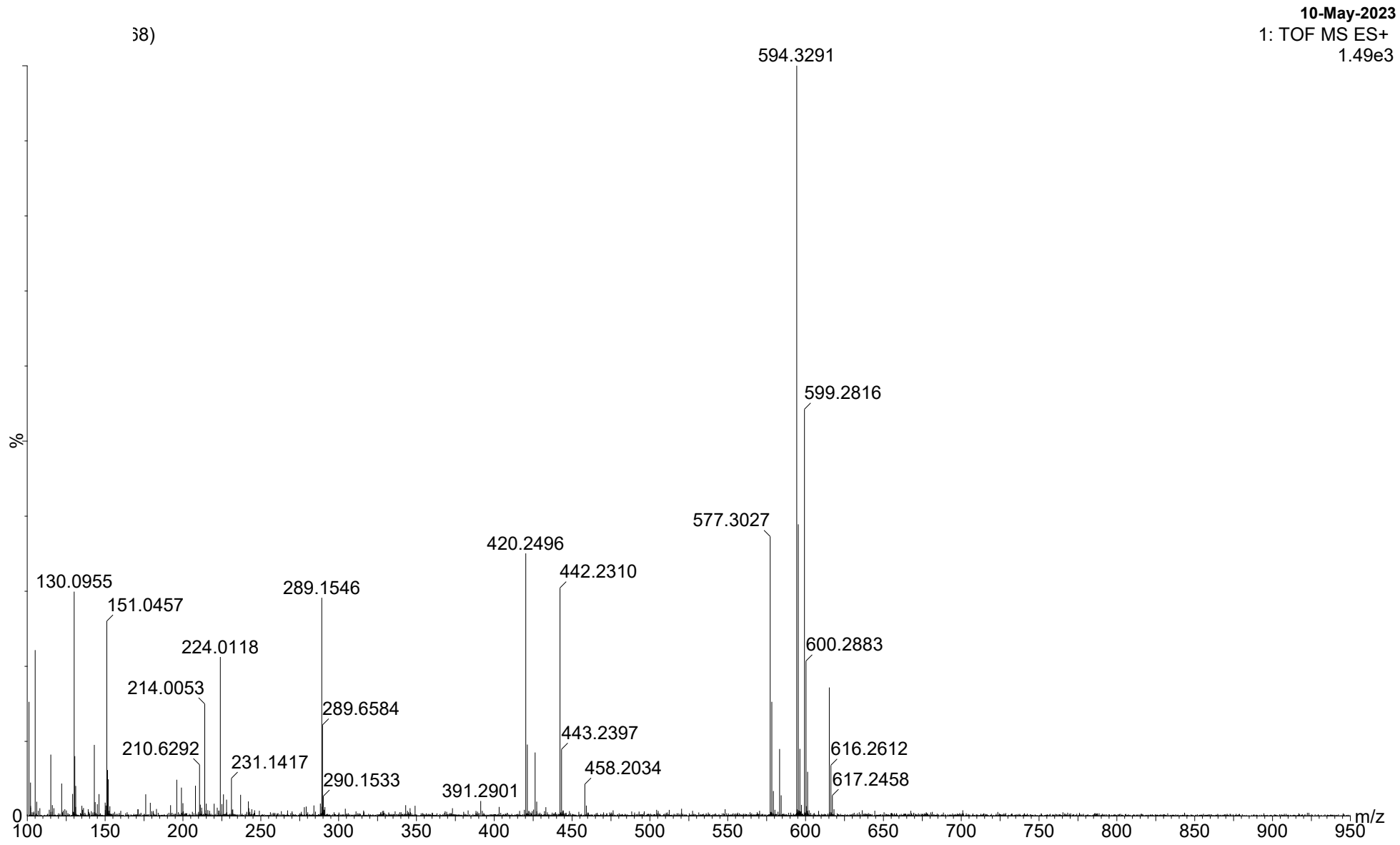


Figure S8. HRMS(ES-TOF+) of **$H_2$KHQ.** *m/z* calcd. for $C_{32}H_{41}N_4O_6$ ([M+H]$^+$) at 577.3026; found as 577.3027.

# 3. X-ray Crystallography

## 3.1. Experimental Details

Single crystal X-ray data were collected using an Agilent Xcalibur PX Ultra A diffractometer, and the structures were refined using the OLEX2 and SHELX-2019 program systems.[5–7] Details of the individual data collection are provided in the relevant CIFs. A summary of the fundamental crystal and refinement data are given in Table S1. Atomic coordinates, anisotropic displacement parameters and bond lengths and angles can be found in the .cif files, which have been deposited in the Cambridge Crystallographic Data Centre.

## 3.2. Supplementary Data

Table S1. Crystal Data, Data Collection and Refinement Parameters for the structures **[$H_2$KHQ]($ClO_4$)$_2$** and **[La-HKHQ]($ClO_4$)$_2$**. [a] Completeness to 0.84 Å resolution. [b] $R_1 = \Sigma||F_o| - |F_c||/\Sigma|F_o|$; $wR_2 = \{\Sigma[w(F_o^2 - F_c^2)^2] / \Sigma[w(F_o^2)^2]\}^{1/2}$; $w^{-1} = \sigma^2(F_o^2) + (aP)^2 + bP$.

| | [$H_2$KHQ]($ClO_4$)$_2$ | [La-HKHQ]($ClO_4$)$_2$ |
|---|---|---|
| Formula | $C_{32}H_{42}Cl_2N_4O_{14}$ | $C_{32}H_{39}Cl_2LaN_4O_{14}$ |
| Solvent | – | – |
| Formula weight | 777.59 | 913.48 |
| Colour, habit | yellow platy | pale green platy |
| Temperature / K | 173 | 173 |
| Crystal system | monoclinic | monoclinic |
| Space group | P2$_1$/c | P2$_1$/c |
| *a* / Å | 10.2238(4) | 20.3932(4) |
| *b* / Å | 14.4135(6) | 11.4737(2) |
| *c* / Å | 12.2816(5) | 16.2053(4) |
| α / deg | 90 | 90 |
| β / deg | 101.310(4) | 109.809(3) |
| γ / deg | 90 | 90 |
| *V* / Å$^3$ | 1774.68(13) | 3567.43(14) |
| Z | 2 | 4 |
| $D_c$ / g cm$^{-3}$ | 1.455 | 1.701 |
| Radiation used | Cu Kα | Cu Kα |
| μ / mm$^{-1}$ | 2.291 | 11.283 |
| No. of unique reflns | | |

| | | |
|---|---|---|
| measured ($R_{int}$) | 11296 (0.0487) | 21197 (0.0606) |
| obs, $\|F_o\| > 4\sigma(\|F_o\|)$ | 2644 | 4382 |
| Completeness (%)[a] | 99.9 | 97.4 |
| No. of variables | 243 | 498 |
| $R_1$(obs), $wR_2$(all)[b] | 0.0488, 0.1413 | 0.0385, 0.0992 |
| CCDC code | 2435038 | 2435039 |

### [$H_2$KHQ]($ClO_4$)$_2$

N–H and O–H hydrogen atoms were refined freely. The interatomic distances of O21–O7 (2.896 Å), and O21–N12 and (2.886 Å) are indicative of a hydrogen bonding interaction (<3.5 Å).

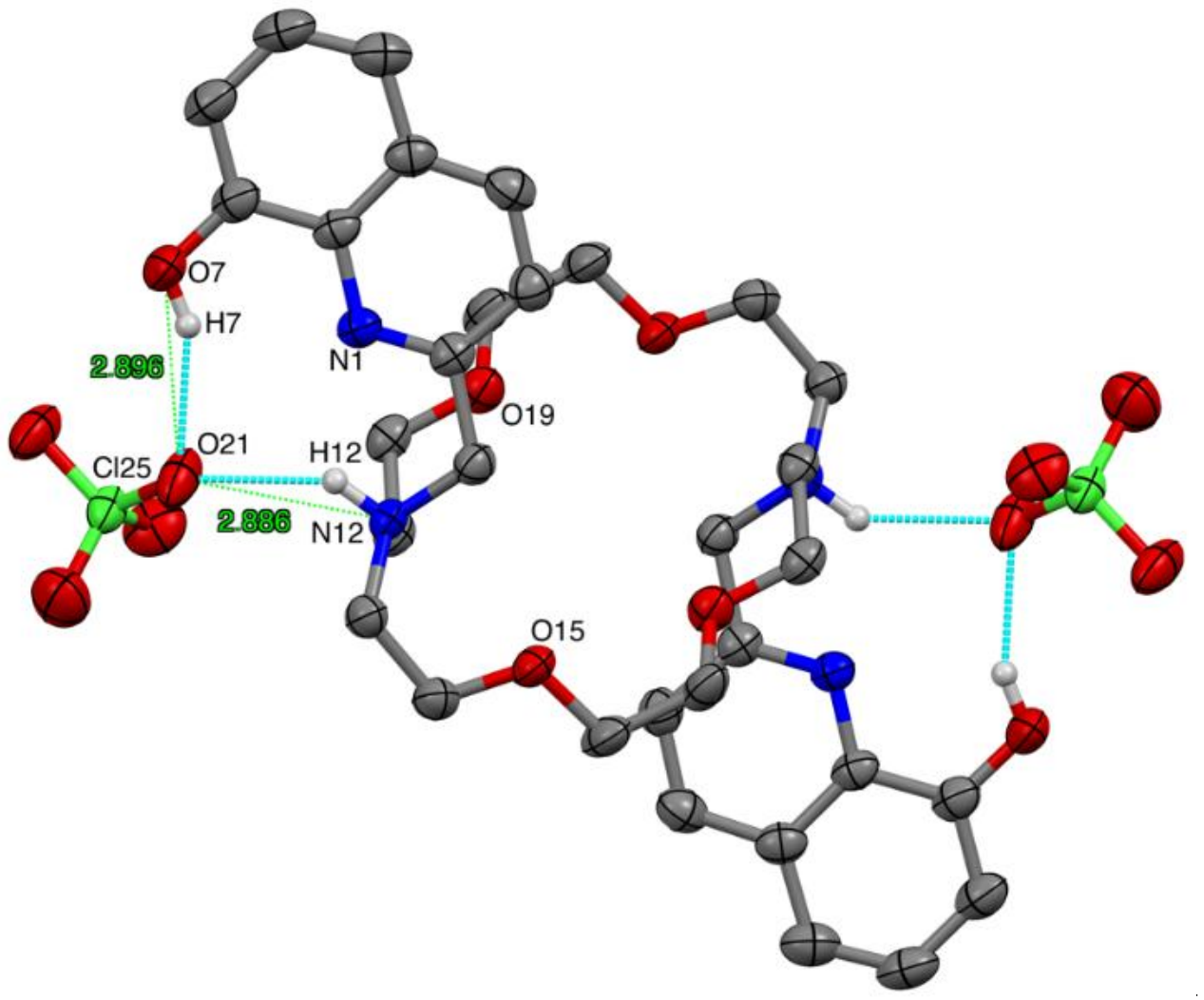


Figure S9. The structure of **[$H_2$KHQ]($ClO_4$)$_2$** (50% probability ellipsoids). H atoms omitted for clarity. H-bonding contacts in teal and interatomic distances in green.

### [La-HKHQ]($ClO_4$)$_2$

O–H hydrogen atoms were refined freely. The interatomic distances of O43–O8 (2.691 Å) are indicative of a hydrogen bonding interaction (<3.5 Å).

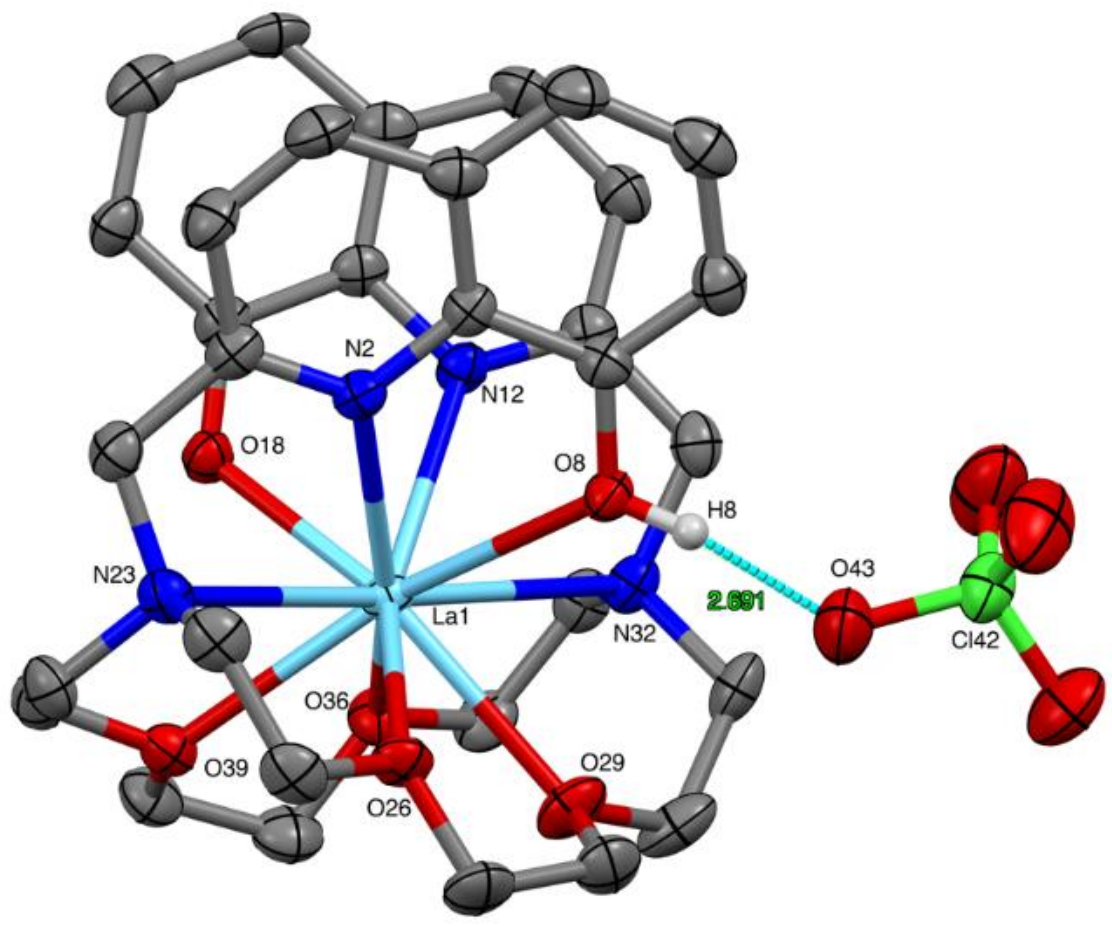


Figure S10. The structure of **[La-HKHQ]($ClO_4$)$_2$** (50% probability ellipsoids). H atoms and second perchlorate anion omitted for clarity. H-bonding contacts in teal and interatomic distances in green.

# 4. NMR spectroscopy of $Ln^{3+}$ complexes

## 4.1. Experimental Details

The NMR samples were prepared by mixing the **$H_2$KHQ** ligand and $LnCl_3$ stock solutions ($LaCl_3$•$7H_2O$, $TbCl_3$•$6H_2O$, $LuCl_3$•$6H_2O$) in a 1:1.2 molar radio in $D_2O$. The pD was carefully adjusted with NaOD/DCl solutions. The $^{1}H$ NMR spectra were referenced to residual internal HDO solvent peak at 4.79 ppm, which was calculated according to the literature equation that accounts for the temperature dependence of the HDO chemical shift.[4] The $^{13}C\{^{1}H\}$ spectra were referenced indirectly to the corresponding $^{1}H$ spectra using the "absolute reference" function provided in *MestReNova* (Mestrelab Research S.L.).

**[La-KHQ]$^+$**

**$^{1}$H NMR (400 MHz, $D_2O$) $\delta_H$ (ppm):** 7.64 (d, $^{3}J_{HH}$ = 8.5 Hz, 2H, $H_{HQ}$), 6.99 (t, $^{3}J_{HH}$ = 8.0 Hz, 2H, $H_{HQ}$), 6.92 (d, $^{3}J_{HH}$ = 8.5 Hz, 2H, $H_{HQ}$), 6.59 (d, $^{3}J_{HH}$ = 8.0 Hz, 2H, $H_{HQ}$), 6.47 (d, $^{3}J_{HH}$ = 7.5 Hz, 2H, $H_{HQ}$), 4.88 (d, $^{3}J_{HH}$ = 16.5 Hz, 2H, $H_{CH2}$), 4.35 (t, $^{3}J_{HH}$ = 10.0 Hz, 2H, $H_{CH2}$), 4.24 – 4.11 (m, 4H, $H_{CH2}$), 4.04 (dd, $^{3}J_{HH}$ = 16.5, 17.5 Hz, 6H, $H_{CH2}$), 3.94 (s, 4H, $H_{CH2}$), 3.71 – 3.64 (m, 2H, $H_{CH2}$), 3.54 (t, $^{3}J_{HH}$ = 11.0 Hz, 2H, $H_{CH2}$), 3.15 (d, $^{3}J_{HH}$ = 13.5 Hz, 2H, $H_{CH2}$), 2.94 (d, $^{3}J_{HH}$ = 8.0 Hz, 2H, $H_{CH2}$), 2.69 (d, $^{3}J_{HH}$ = 14.0 Hz, 2H, $H_{CH2}$). **$^{13}C\{^{1}H\}$ NMR (126 MHz, $D_2O$) $\delta_C$ (ppm):** 171.7, 162.9, 156.5, 141.8, 138.36, 129.1, 128.3, 120.0, 112.6, 111.6, 73.7, 71.9, 71.5, 65.6, 57.5, 55.9. **HRMS(ES-TOF+)**: *m/z* calcd. for $C_{32}H_{38}N_4LaO_6$ [M]$^+$ at 713.1855; found as 713.1829.

**[Lu-KHQ]$^+$**

**$^{1}$H NMR (400 MHz, $D_2O$, pD 9) $\delta_H$ (ppm):** 8.48 (d, $^{3}J_{HH}$ = 8.5 Hz, 2H, $H_{HQ}$), 7.58 – 7.51 (m, 4H, $H_{HQ}$), 7.30 (d, $^{3}J_{HH}$ = 8.5 Hz, 2H, $H_{HQ}$), 6.94 (d, $^{3}J_{HH}$ = 7.5 Hz, 2H, $H_{HQ}$), 4.68 – 4.05 (m, 8H, $H_{CH2}$), 3.85 (s, 2H, $H_{CH2}$), 3.78 (s, 2H, $H_{CH2}$), 3.60 (t, $^{3}J_{HH}$ = 19.0 Hz, 6H, $H_{CH2}$), 3.30 (d, $^{3}J_{HH}$ = 20.0 Hz, 4H, $H_{CH2}$), 3.07 (s, 2H, $H_{CH2}$), 2.79 (d, $^{3}J_{HH}$ = 11.5 Hz, 2H, $H_{CH2}$), 2.59 (d, $^{3}J_{HH}$ = 12.5 Hz, 2H, $H_{CH2}$). **HRMS(ES-TOF$^+$)**: *m/z* calcd. for $C_{32}H_{38}N_4LuO_6$ [M]$^+$ at 749.2199; found as 749.2186.

## 4.2. Supplementary Data

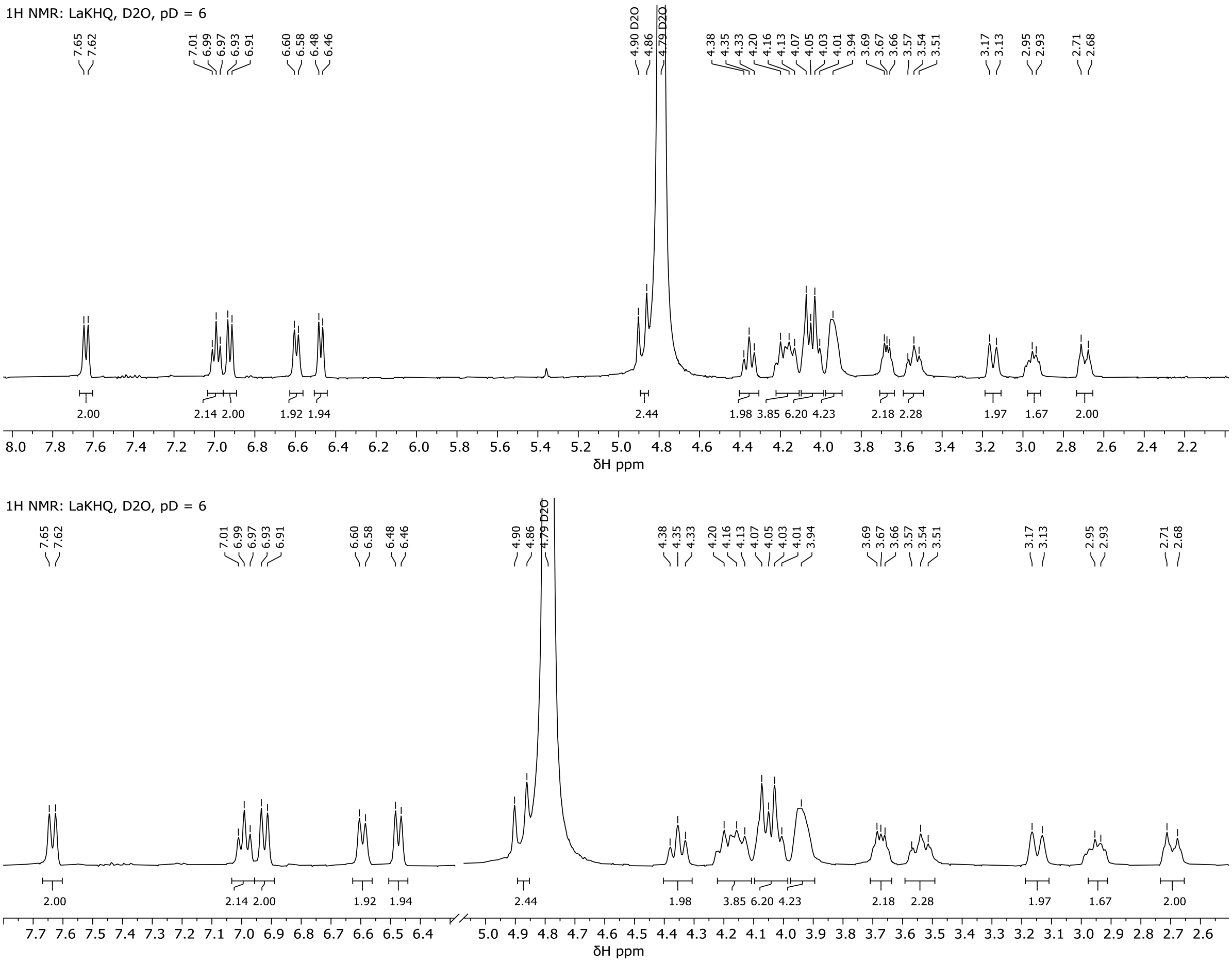


Figure S11. Full width (top) and expanded (bottom) $^{1}H$ NMR spectrum of **[La-KHQ]$^+$** $D_2O$, pD = 6, 400 MHz, 298 K).

13C{1H} NMR of LaKHQ

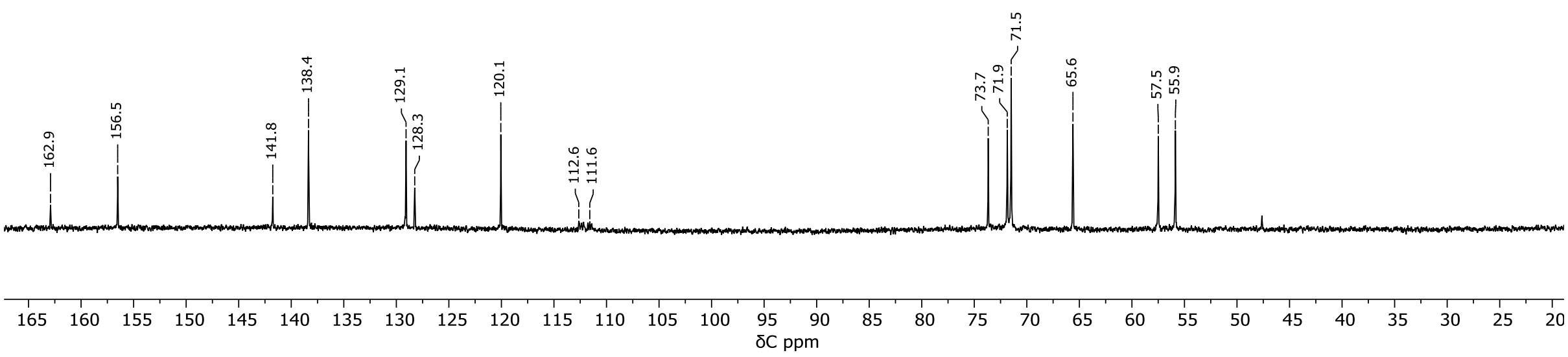


13C{1H} NMR of LaKHQ

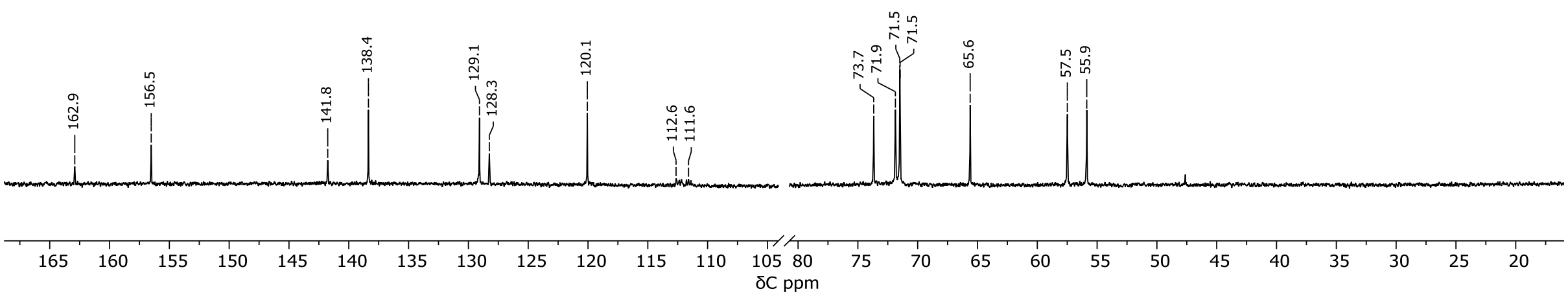


Figure S12. Full width (top) and expanded (bottom) $^{13}C\{^1H\}$ NMR spectrum of **[La-KHQ]$^+$** ($D_2O$, pD = 6, 126 MHz, 353 K).

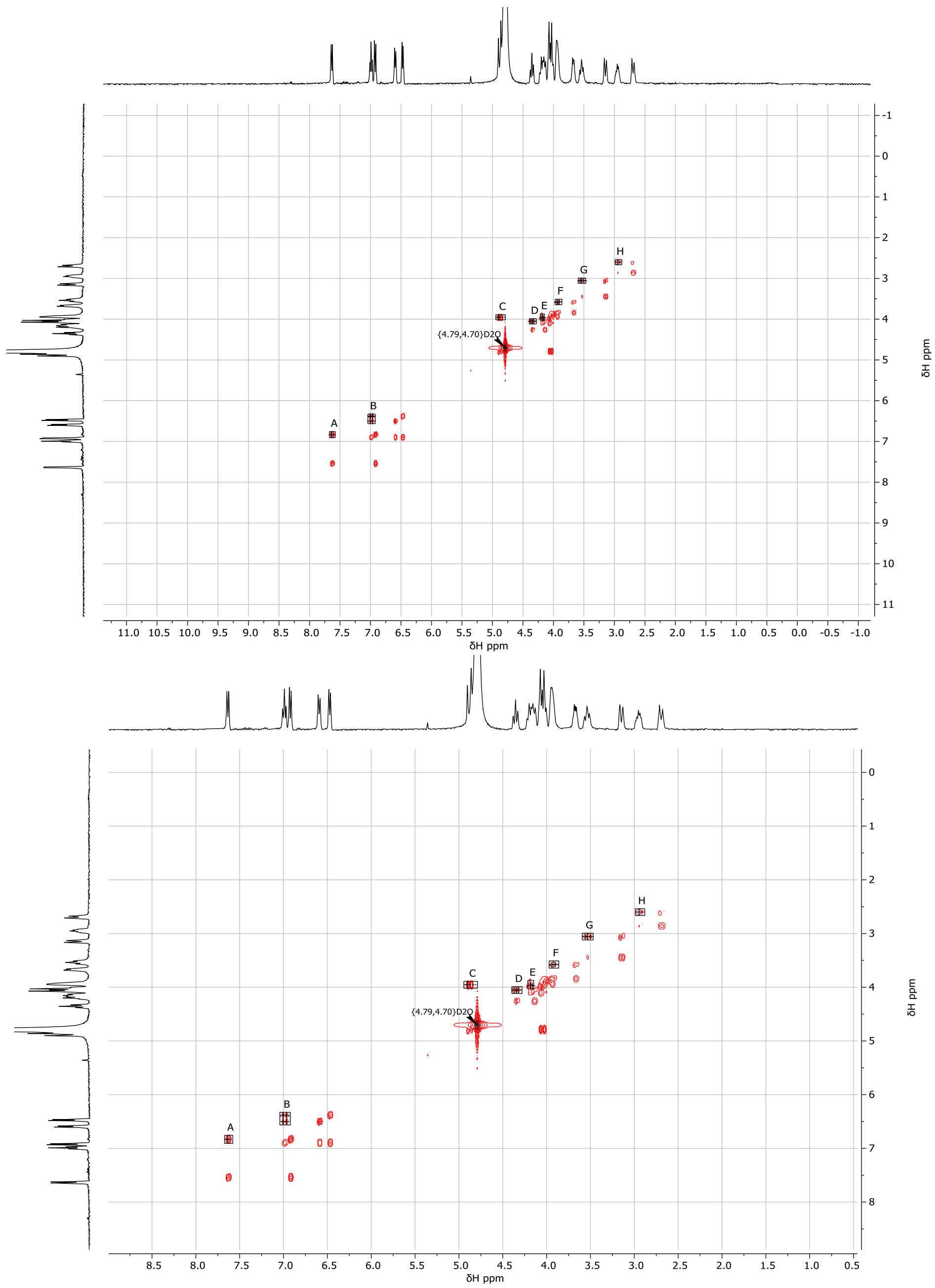


Figure S13. Full width (top) and expanded (bottom) $^{1}H$-$^{1}H$ COSY NMR spectrum of **[La-KHQ]**$^{+}$ ($D_2O$, pD = 6, 298 K).

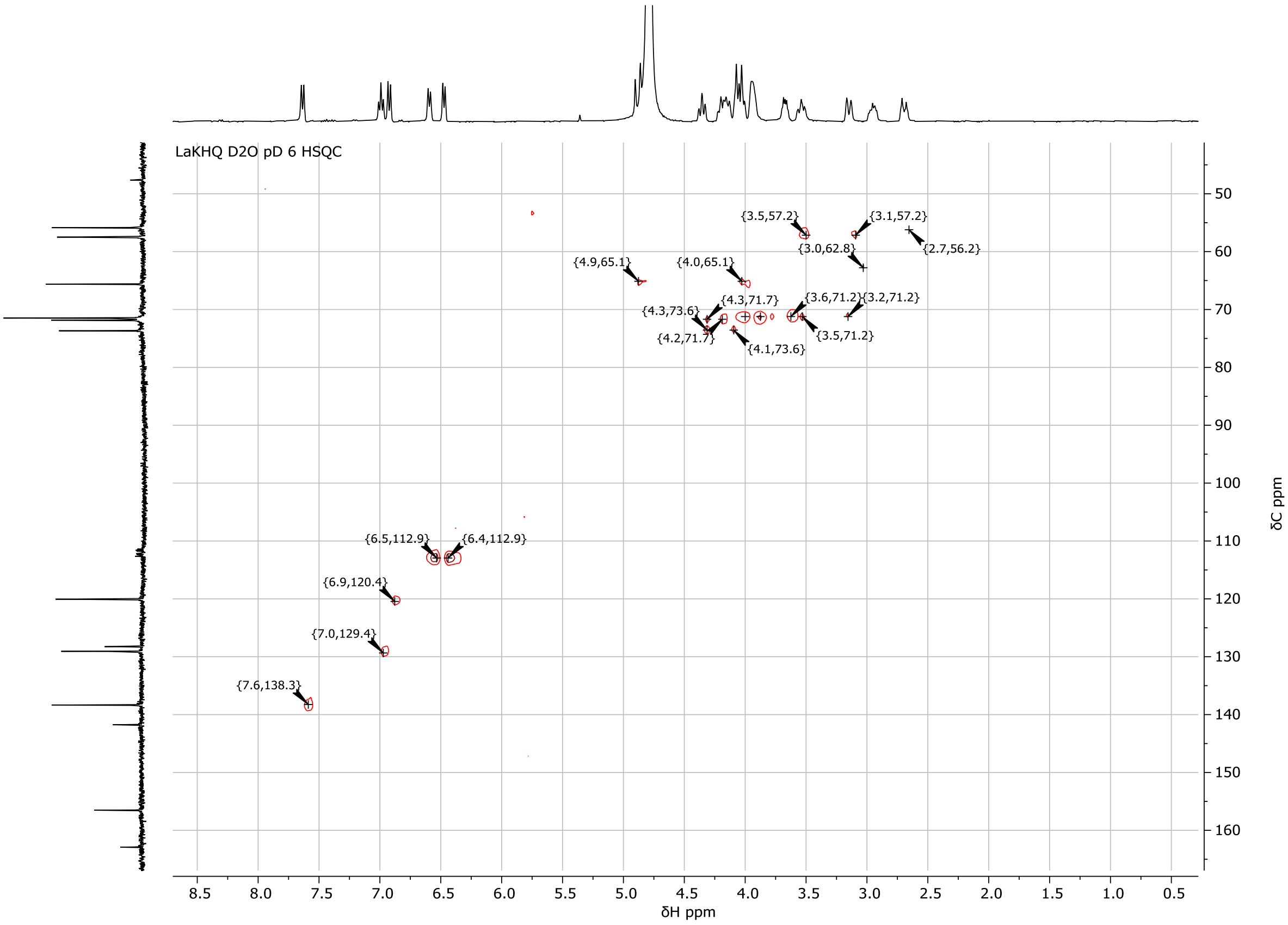


Figure S14. Full width $^1H$-$^{13}C$ HSQC NMR spectrum of **[La-KHQ]**$^+$ ($D_2O$, pD = 6, 298 K).

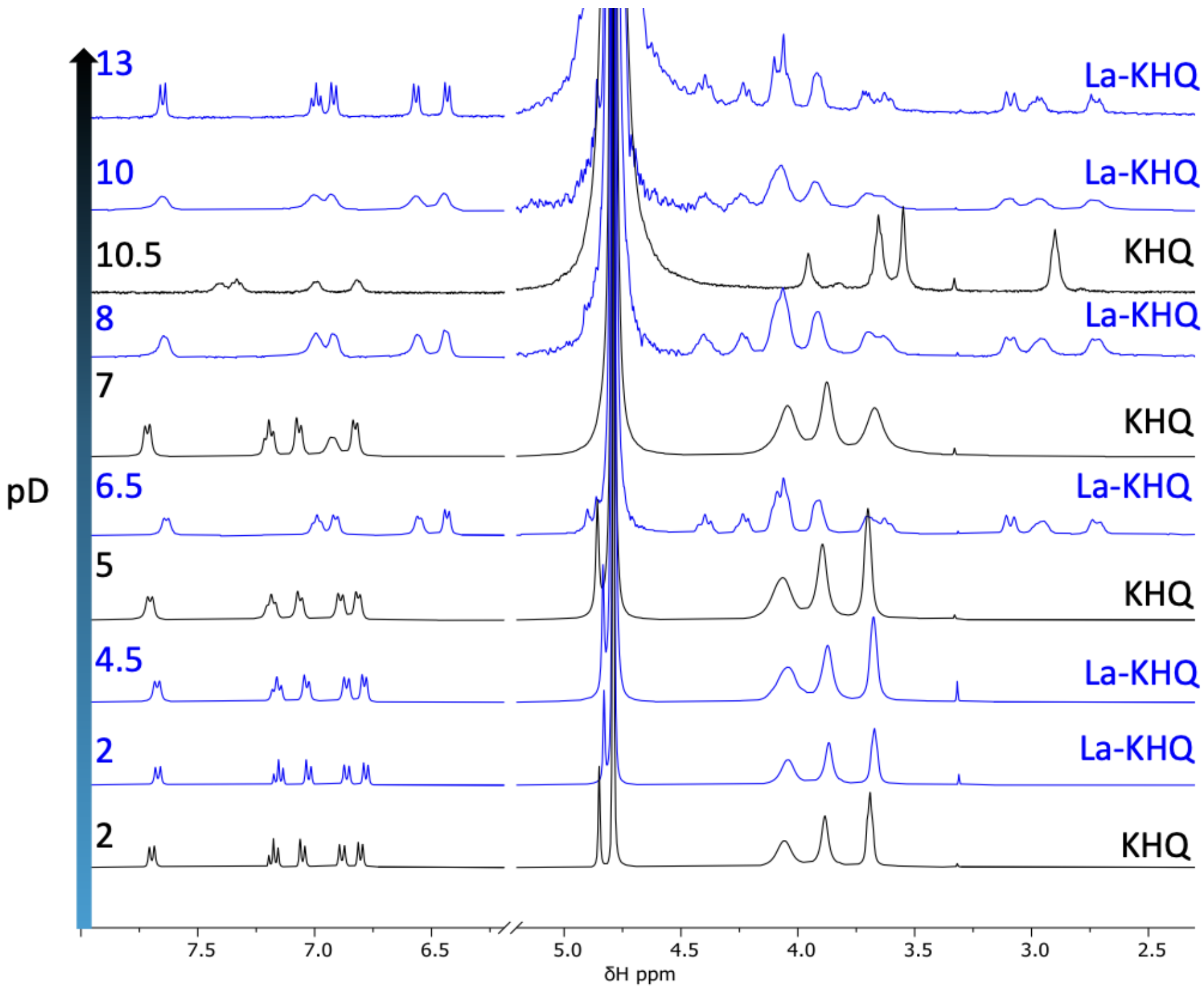


Figure S15. $^1H$ NMR spectroscopic pD-titration series of **$H_2$KHQ** (black spectra) and **[La-KHQ]**$^+$ (blue spectra) obtained from 0.014 M of the compounds in $D_2O$ solutions. The pD range was 2 – 13 (bottom to top) and adjusted using DCl/NaOD. The signal at 4.79 ppm is the residual HDO resonance, overlapping with the bridging $CH_2$ signals. As the pD was increased, the peaks corresponding to free ligand disappeared.

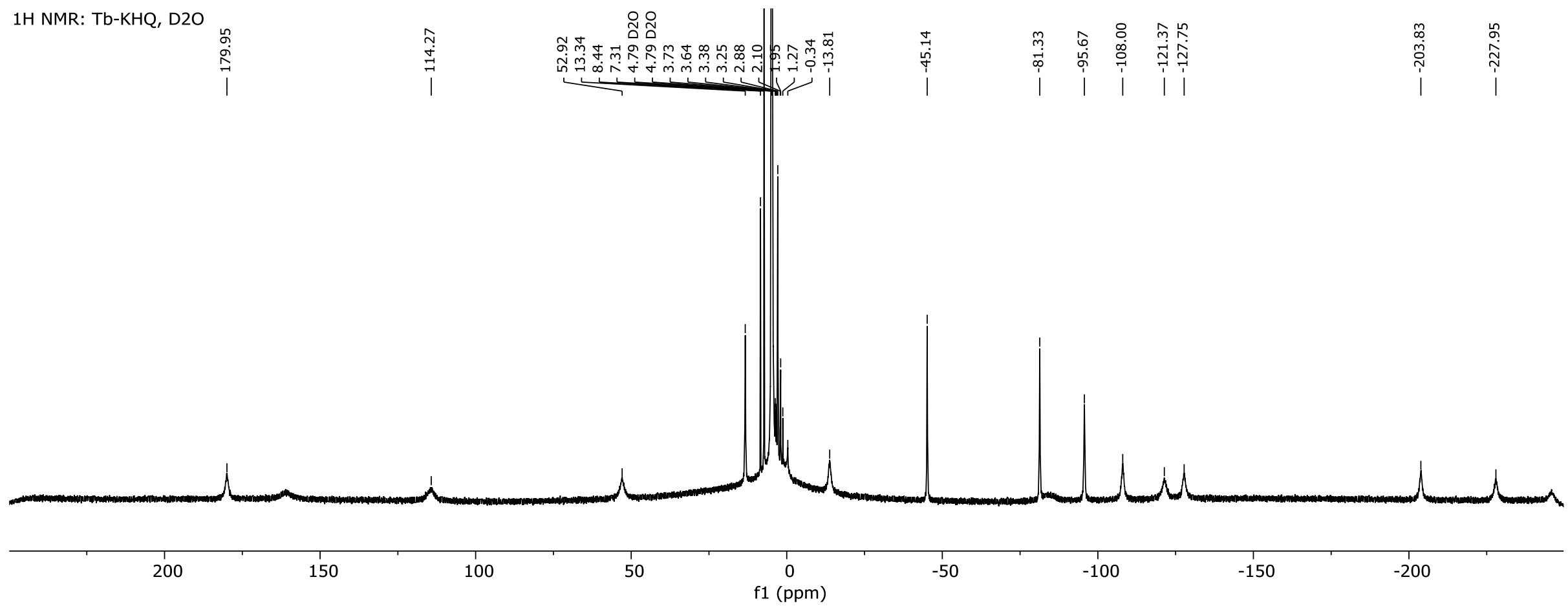


Figure S16. Full width $^{1}H$ NMR spectrum of **[Tb-KHQ]**$^{+}$ ($D_2O$, 400 MHz, 298 K).

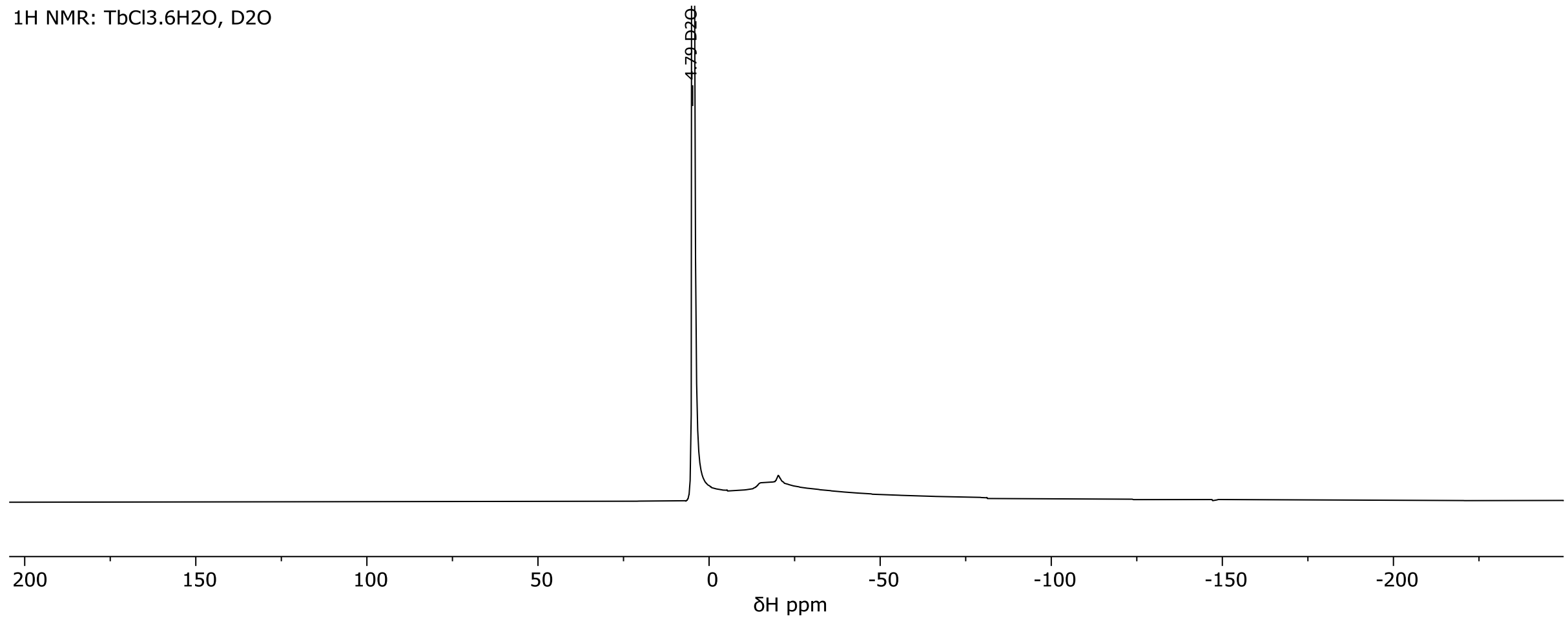


Figure S17. Full-width $^{1}H$ NMR spectrum of **$TbCl_3.6H_2O$** ($D_2O$, 400 MHz, 298 K).

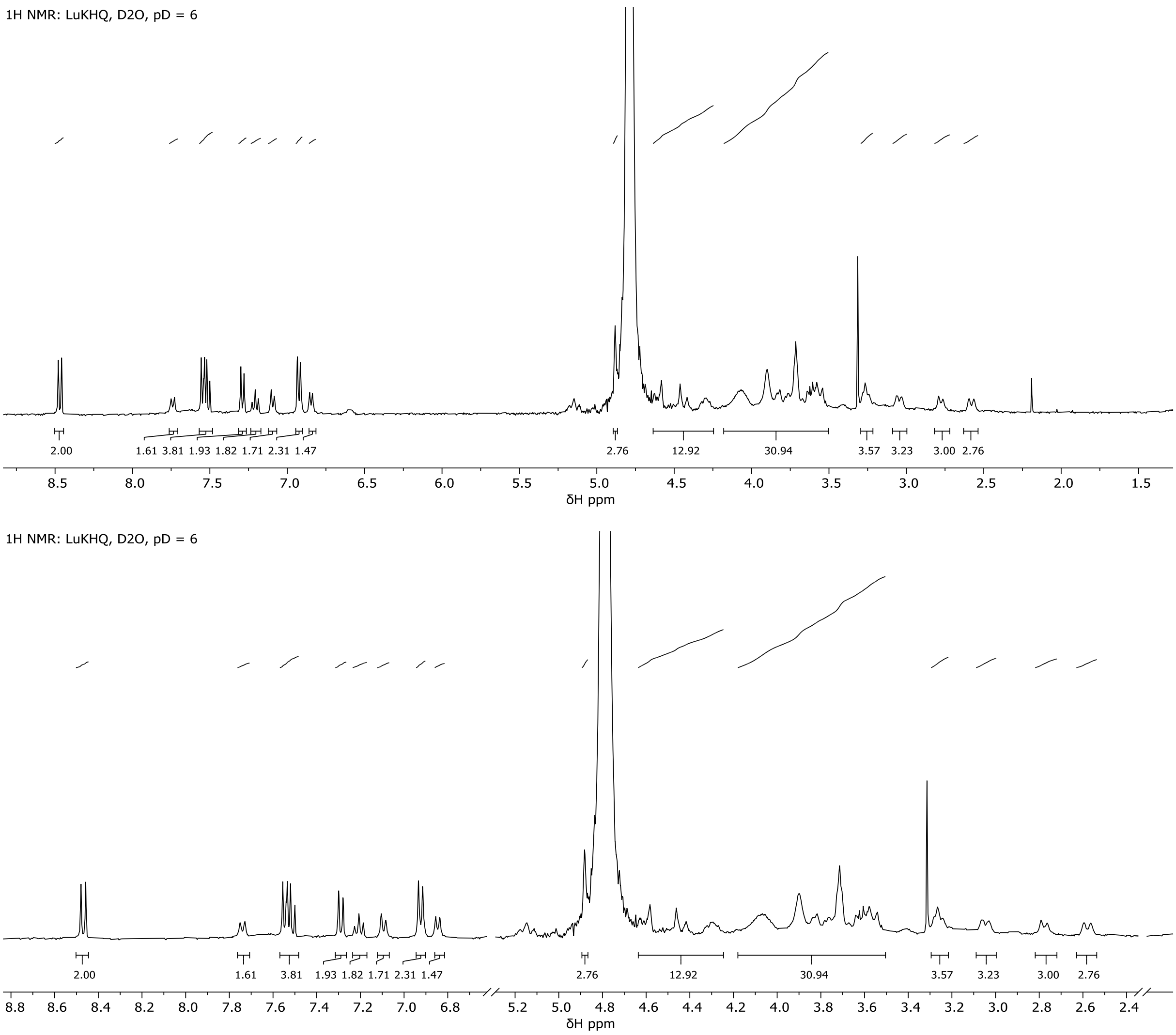


Figure S18. Full width (top) and expanded (bottom) $^1$H NMR spectrum of **[Lu-KHQ]**$^+$ ($D_2O$, pD = 6, 400 MHz, 298 K).

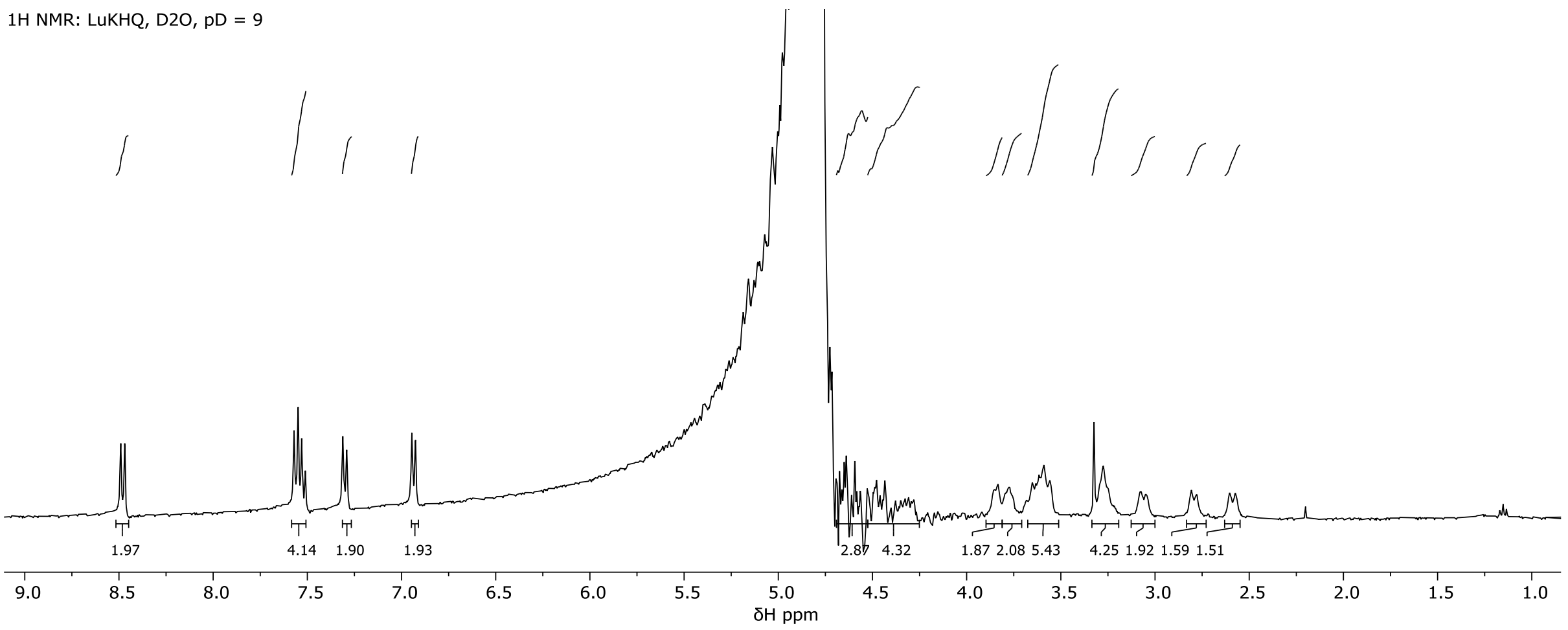


Figure S19. Full width $^1$H NMR spectrum of **[Lu-KHQ]**$^+$ (D2O, pD = 9, 400 MHz, 298 K).

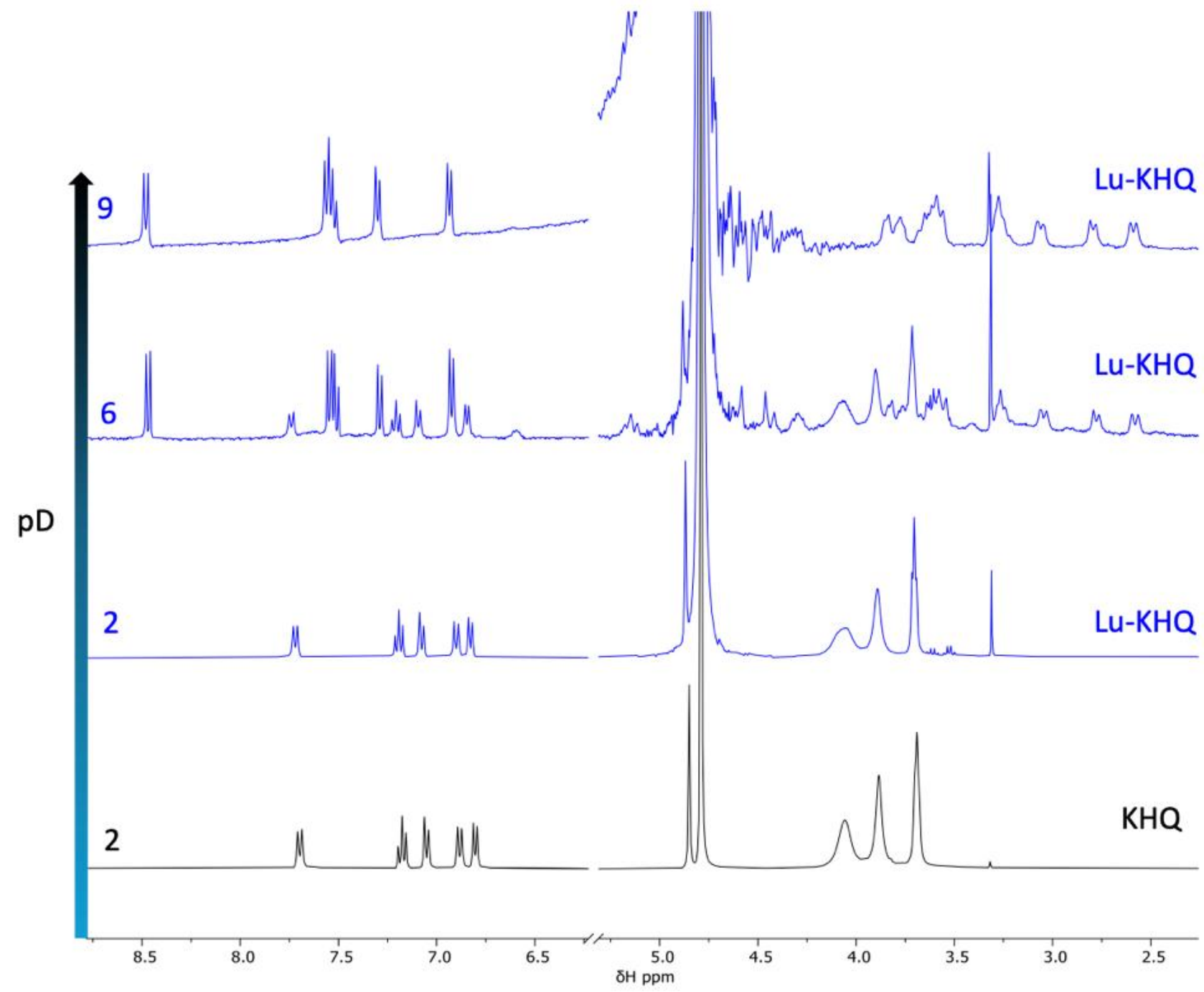


Figure S20. $^{1}$H NMR pD-titration series of **$H_2$KHQ** (black spectra) and **[Lu-KHQ]**$^+$ (blue spectra) obtained from 0.014 M of the compounds in $D_2O$ solutions. The pD range was 2 – 10 (bottom to top) and adjusted using DCl/NaOD. The signal at 4.79 ppm is the residual HDO resonance, overlapping with the bridging $CH_2$ signals. As the pD was increased, the peaks corresponding to free ligand disappeared.

# 5. LC-MS and HPLC experiments

## 5.1. Experimental Details

10 mM **$H_2$KHQ** stock solutions were prepared by dissolving the ligand in 20 mM $NH_4OAc$ buffer (pH 6.5). 24 mM $Ln^{3+}$ stock solutions were prepared by dissolving the chloride salts ($LaCl_3•7H_2O$, $TbCl_3•6H_2O$, $LuCl_3•6H_2O$) in 20 mM $NH_4OAc$ buffer (pH 6.5). The reaction samples were prepared by mixing stock solutions of the **$H_2$KHQ** ligand and $Ln^{3+}$ stock solutions in a 1:1.2 molar ratio in 20 mM $NH_4OAc$ buffer (pH 6.5) to a final concentration of 1 mM. The samples were then left at room temperature or heated to 90 °C for 30 minutes.

## 5.2. Supplementary Data

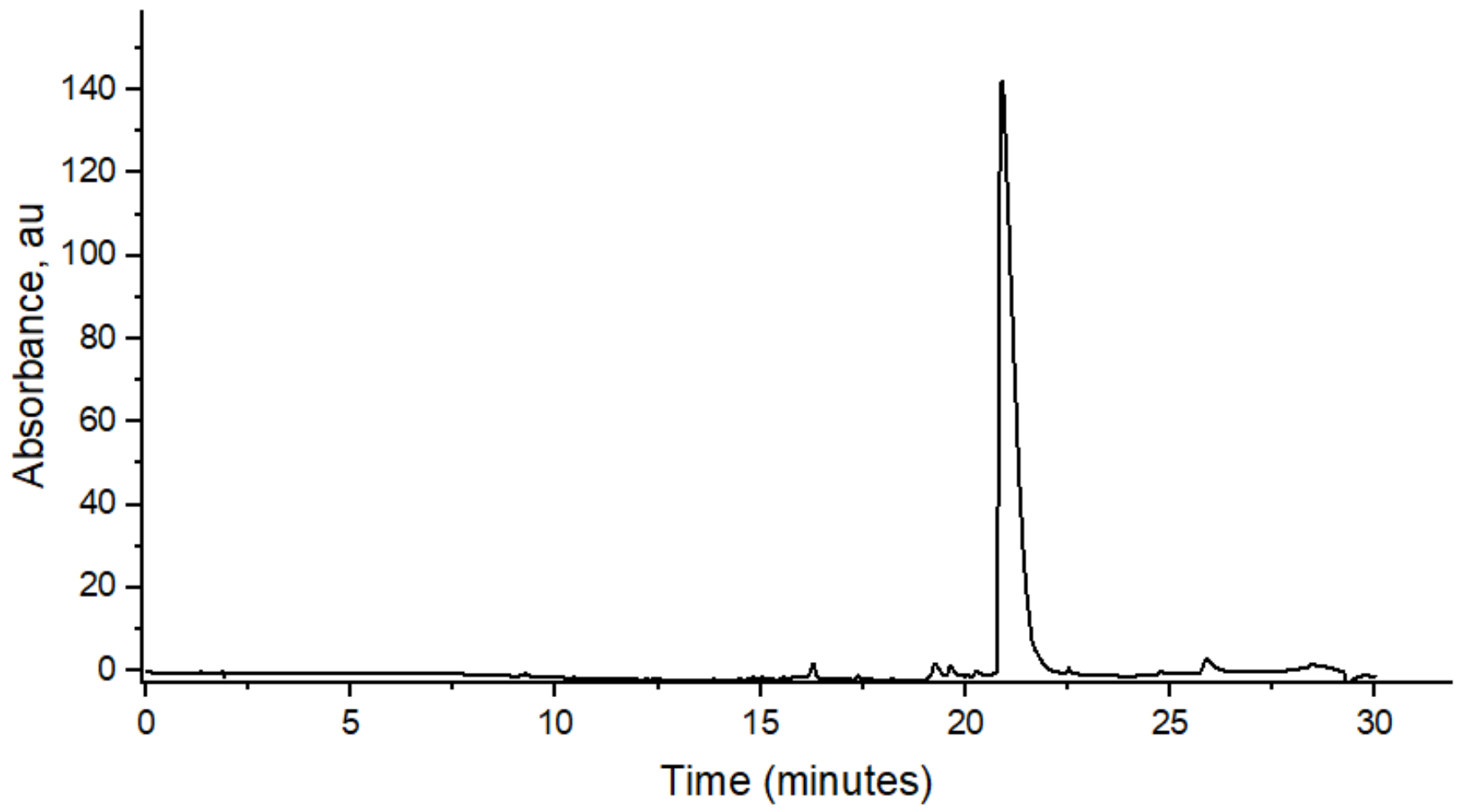


Figure S21. HPLC chromatogram of **$H_2$KHQ** (retention time= 20.9 min).

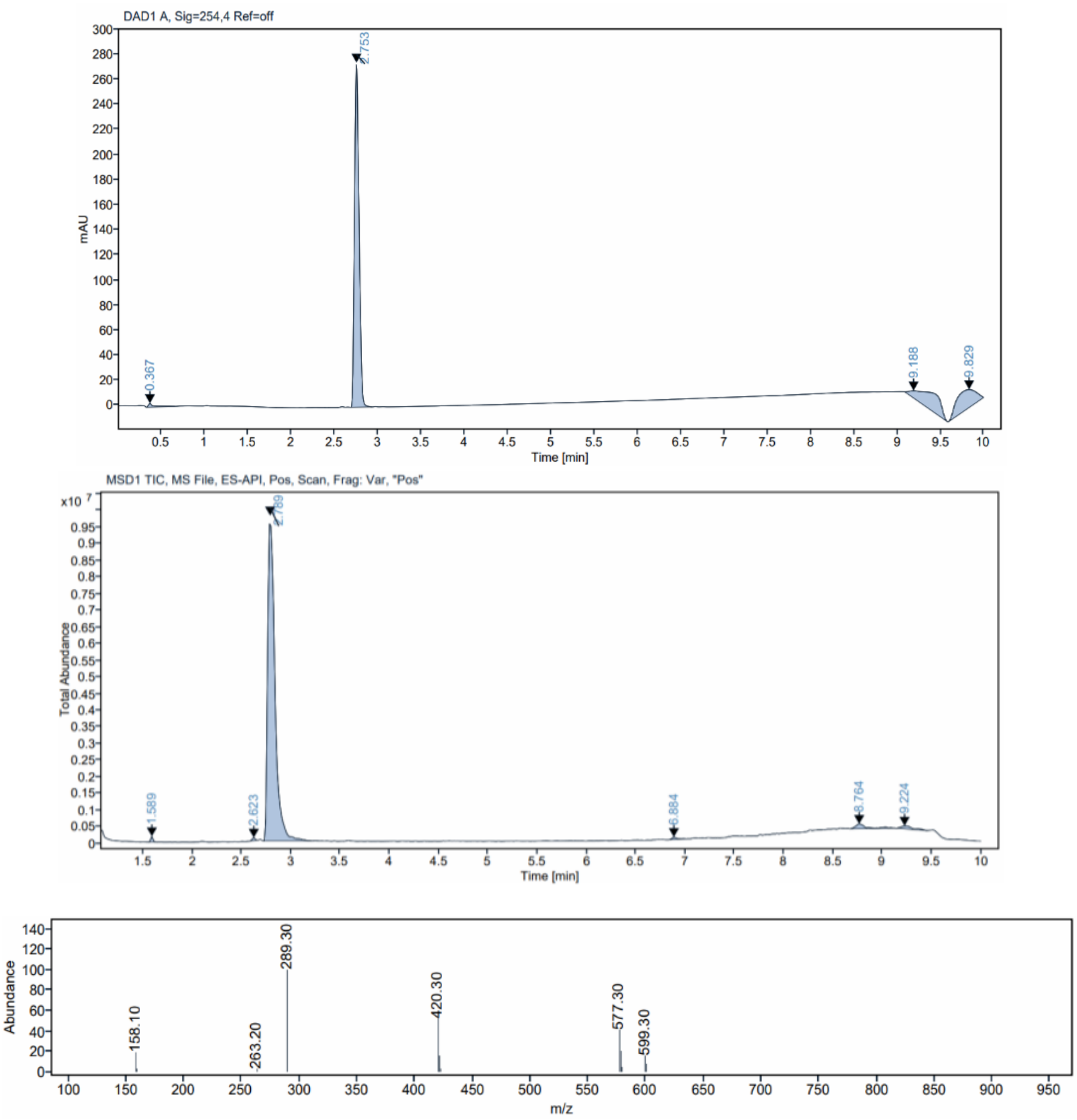


Figure S22. LC-MS of **$H_2$KHQ**. Measured in acidic methods. Retention time = 2.7 min with *m/z* calcd. for $C_{32}H_{41}N_4O_6$ ($[M+H]^+$) at 577.30; found as 577.30. *m/z* calcd. for $C_{32}H_{41}N_4O_6$ ($[M+H]^{2+}$) at 289.65; found as 289.30.

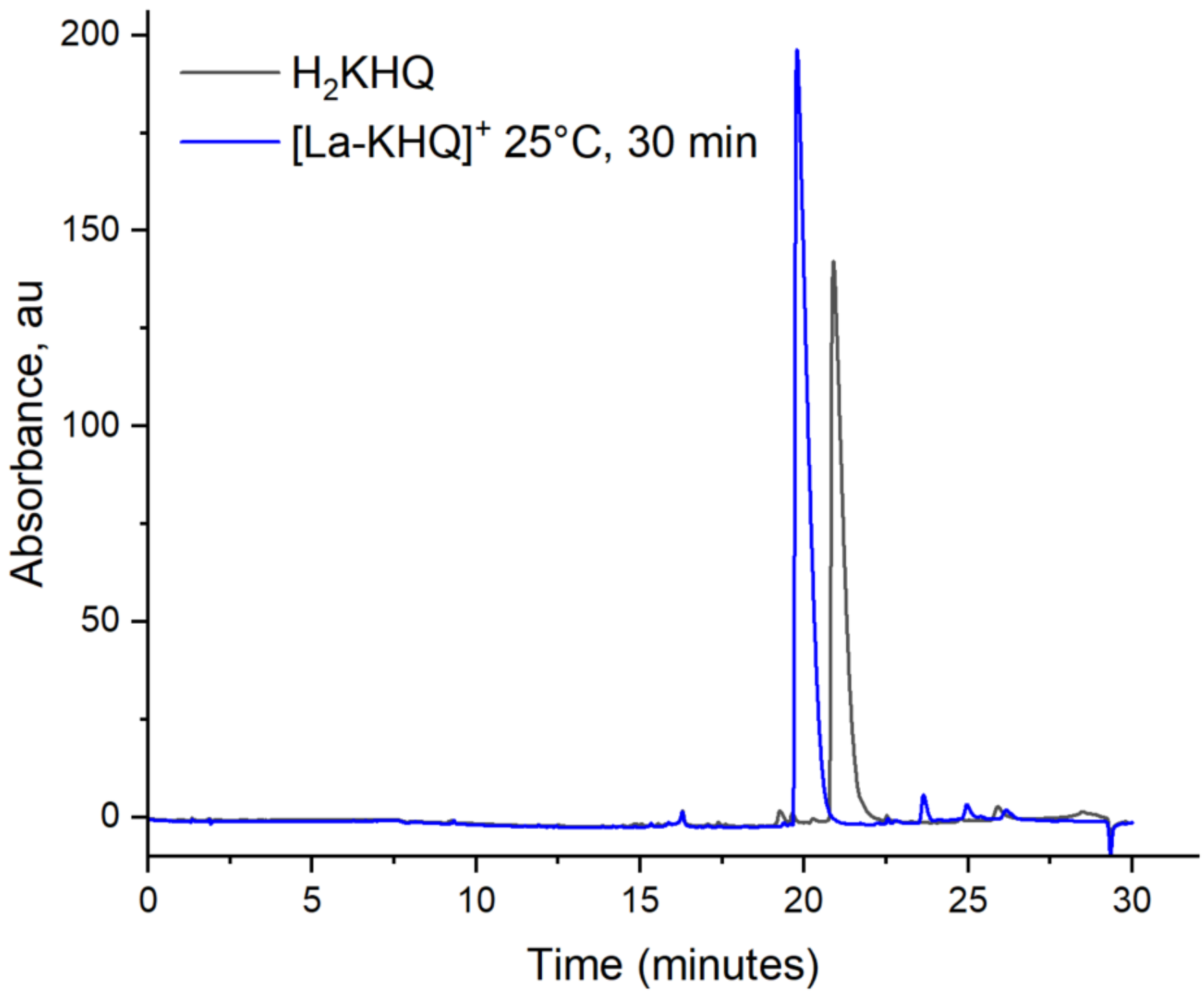


Figure S23. HPLC chromatogram of **$[La\text{-}KHQ]^+$**. **$[La\text{-}KHQ]^+$** retention time= 19.9 min, **$H_2$KHQ** retention time= 20.9 min.

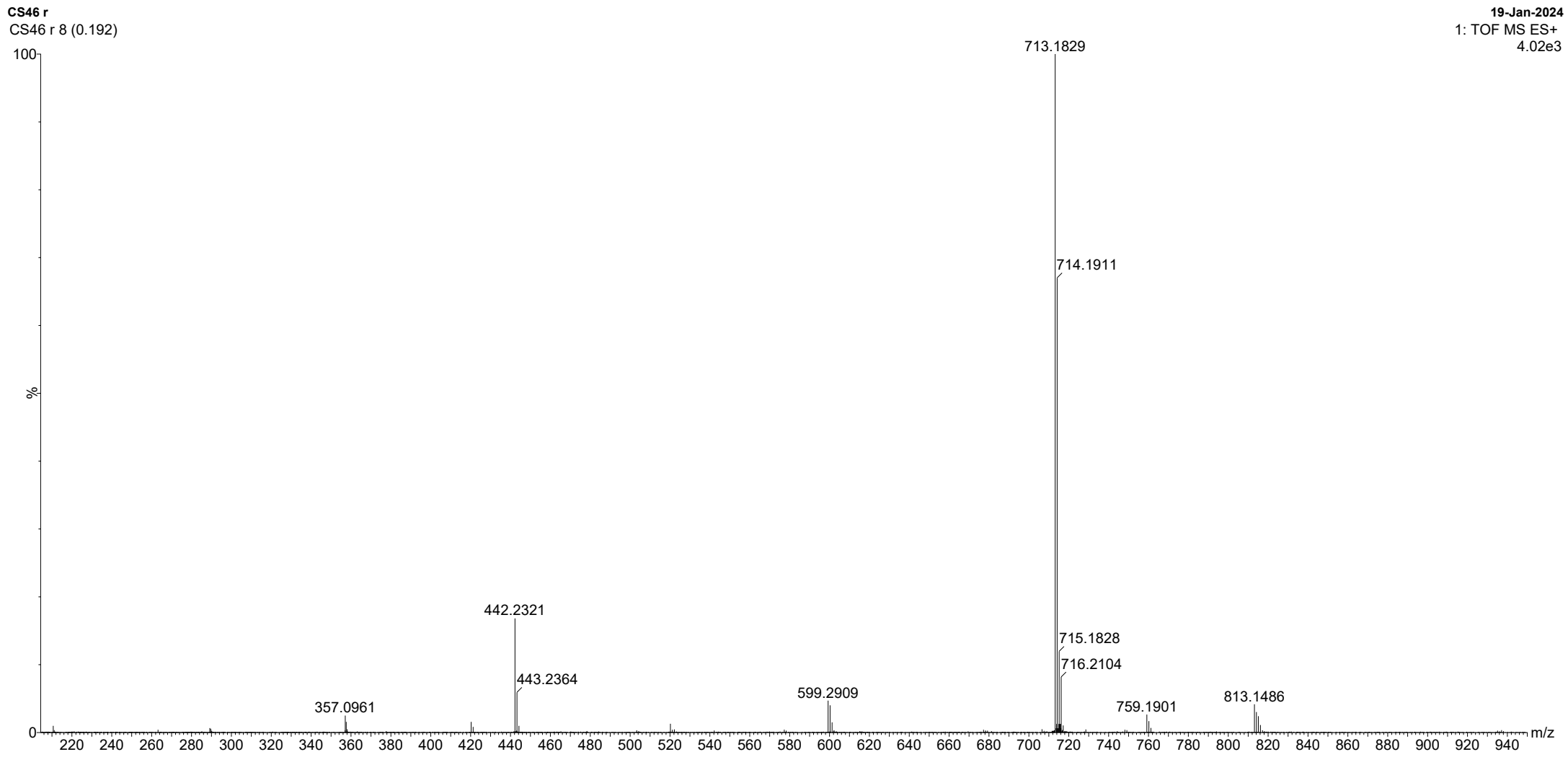


Figure S24. **HRMS(ES-TOF+)** of **[La-KHQ]⁺** *m/z* calcd. for $C_{32}H_{38}N_4LaO_6$ $[M]^+$ at 713.1849; found as 713.1829.

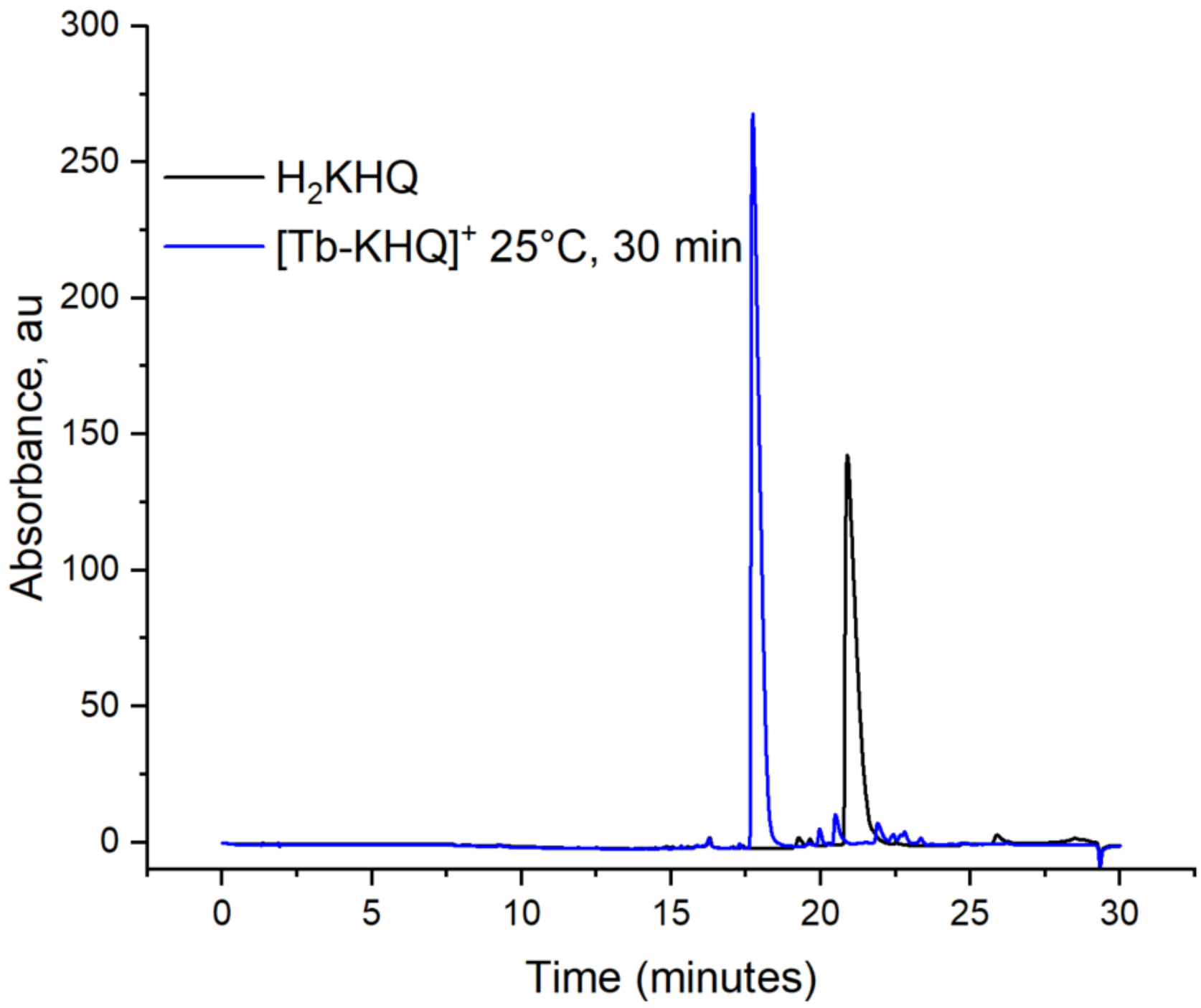


Figure S25. HPLC chromatogram of **[Tb-KHQ]⁺**. **[Tb-KHQ]⁺** retention time= 17.7 min, **$H_2$KHQ** retention time= 20.9 min.

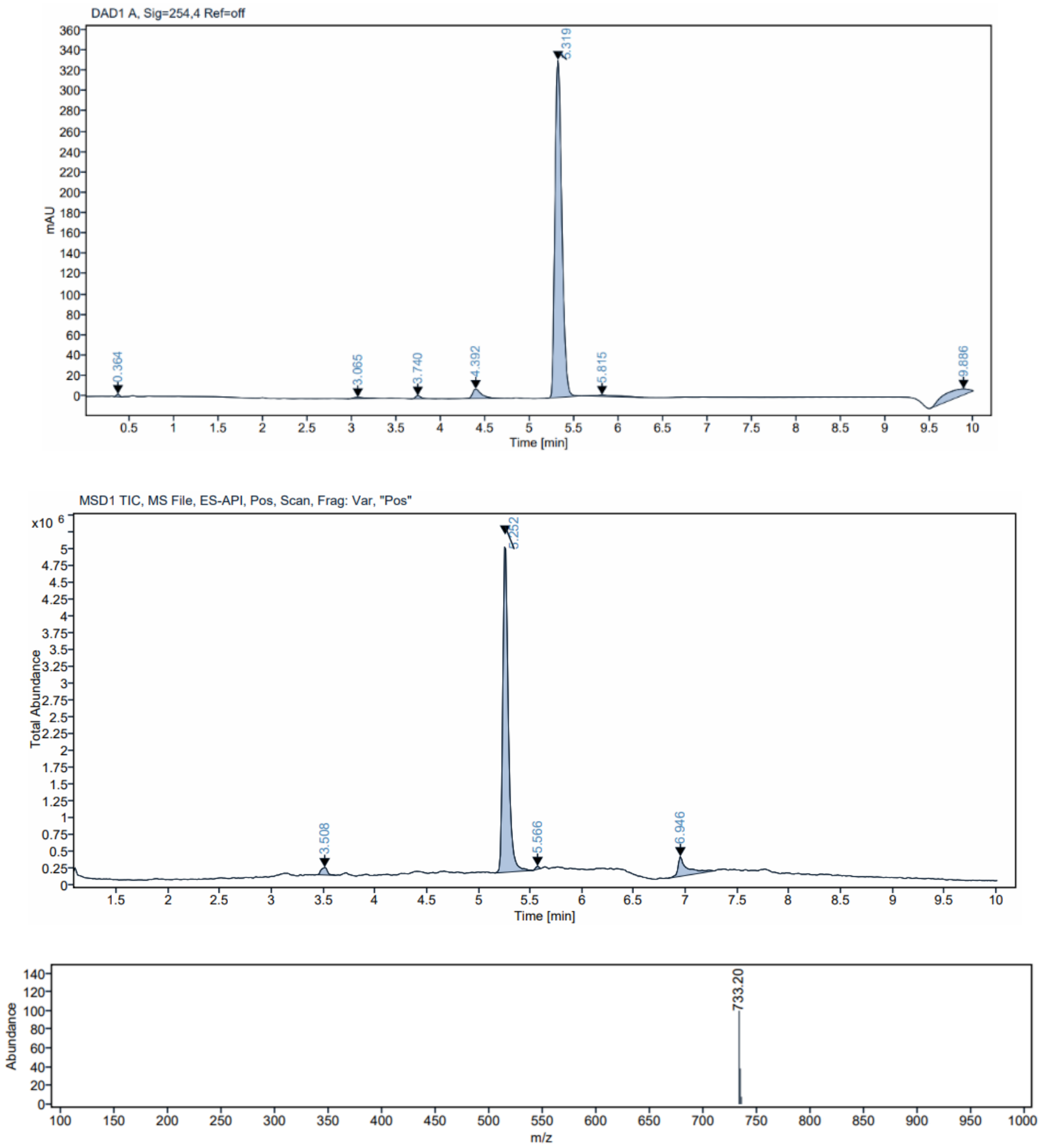


Figure S26. LCMS of **[Tb-KHQ]**$^+$ Measured in basic methods. retention time= 5.3 min with *m/z* calcd. for $C_{32}H_{38}N_4TbO_6$ ($[M+H]^+$) at 733.20; found as 733.20.

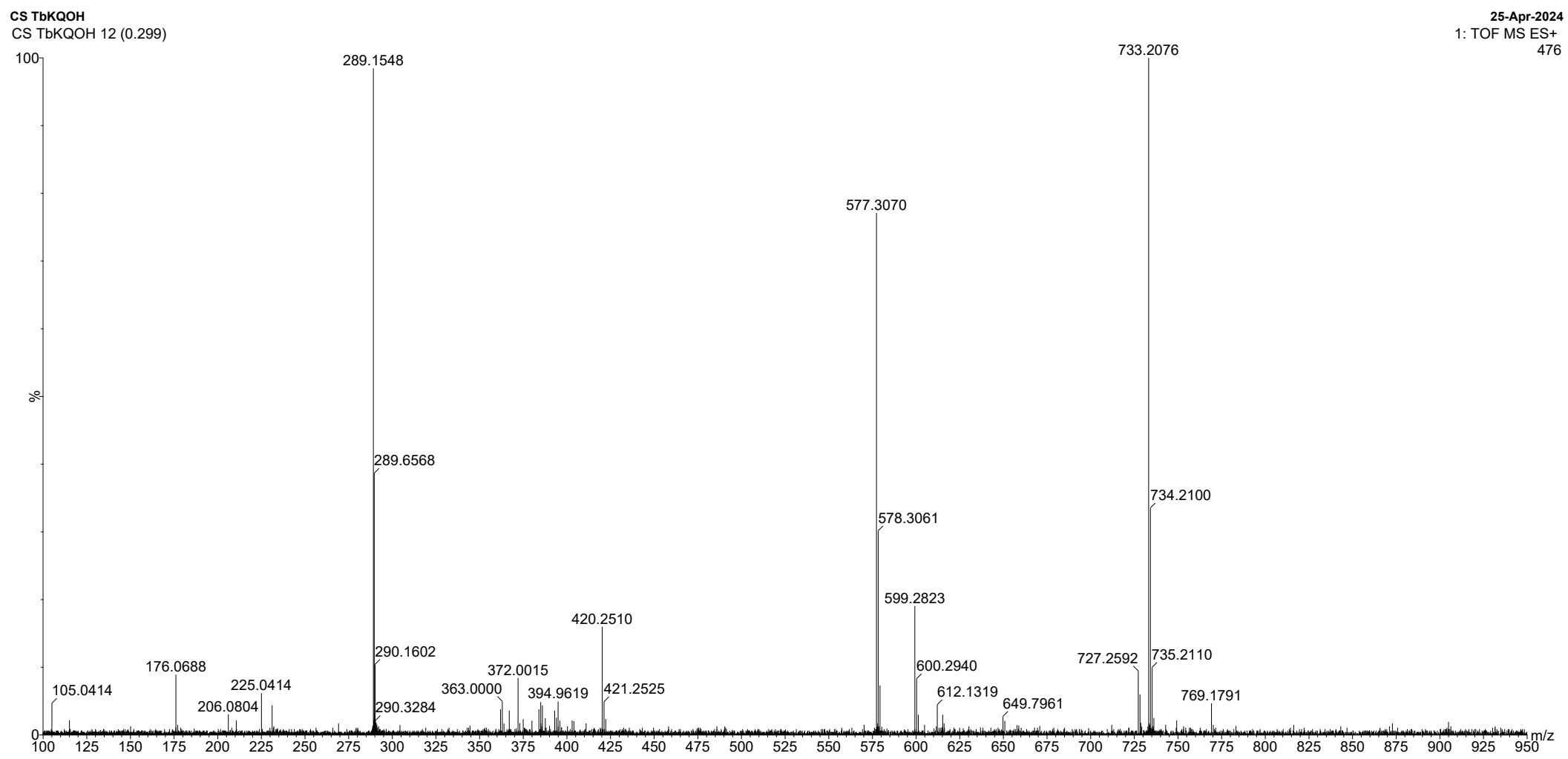


Figure S27. **HRMS(ES-TOF$^+$)** of **[Tb-KHQ]**$^+$ : *m/z* calcd. for $C_{32}H_{38}N_4TbO_6$ $[M]^+$ at 733.2045; found as 733.2076. *m/z* calcd. for $C_{32}H_{38}N_4NaO_6$ $[M]^+$ at 599.2689; found as 599.2844. *m/z* calcd. for $C_{32}H_{38}N_4O_6[M]^+$ at 577.2948; found as 577.3017.

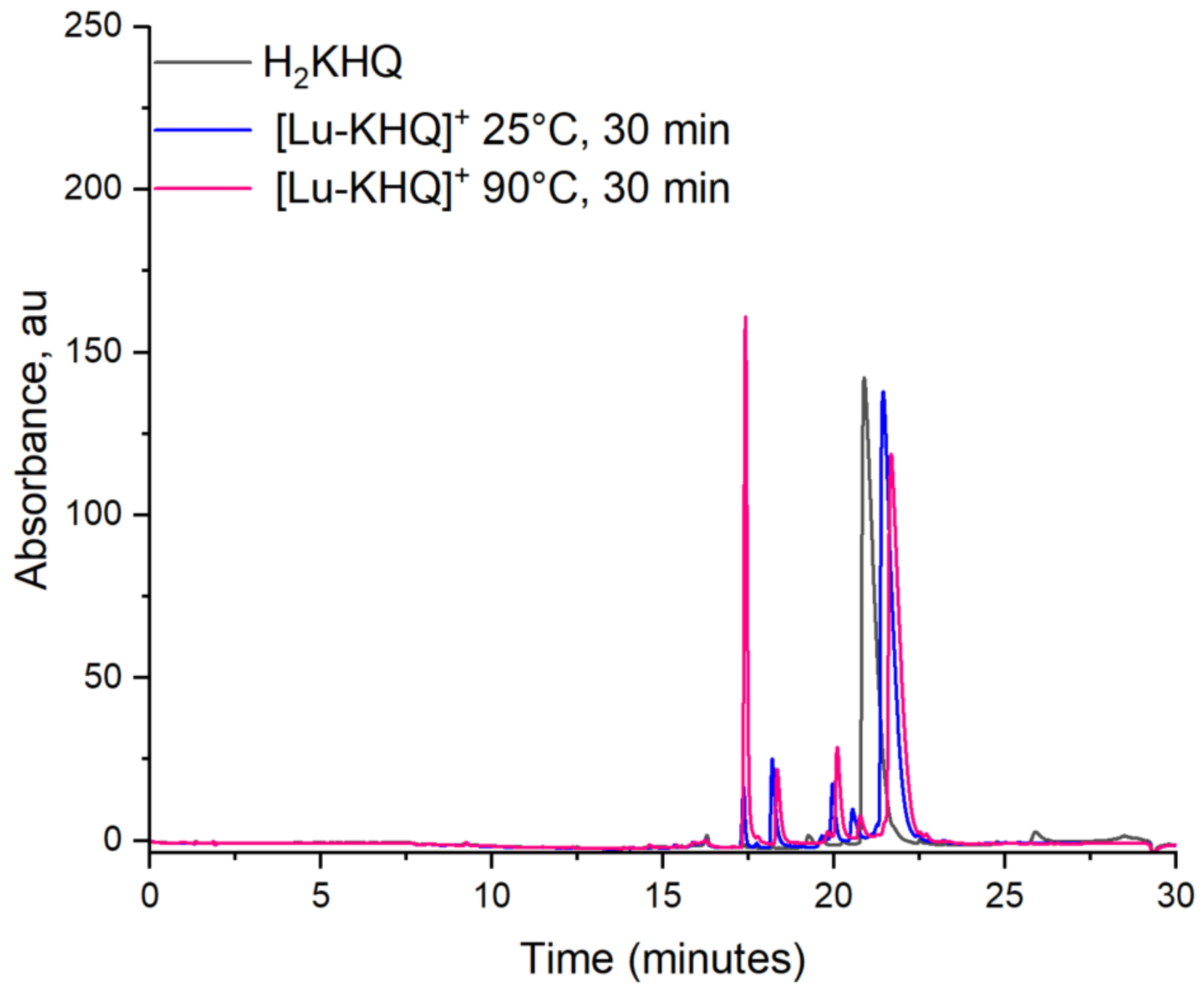


Figure S28. HPLC chromatogram of **[Lu-KHQ]⁺**. **[Lu-KHQ]⁺** retention time= 17.4, 18.3, 20.1, 21.6 min, **$H_2$KHQ** retention time= 20.9 min.

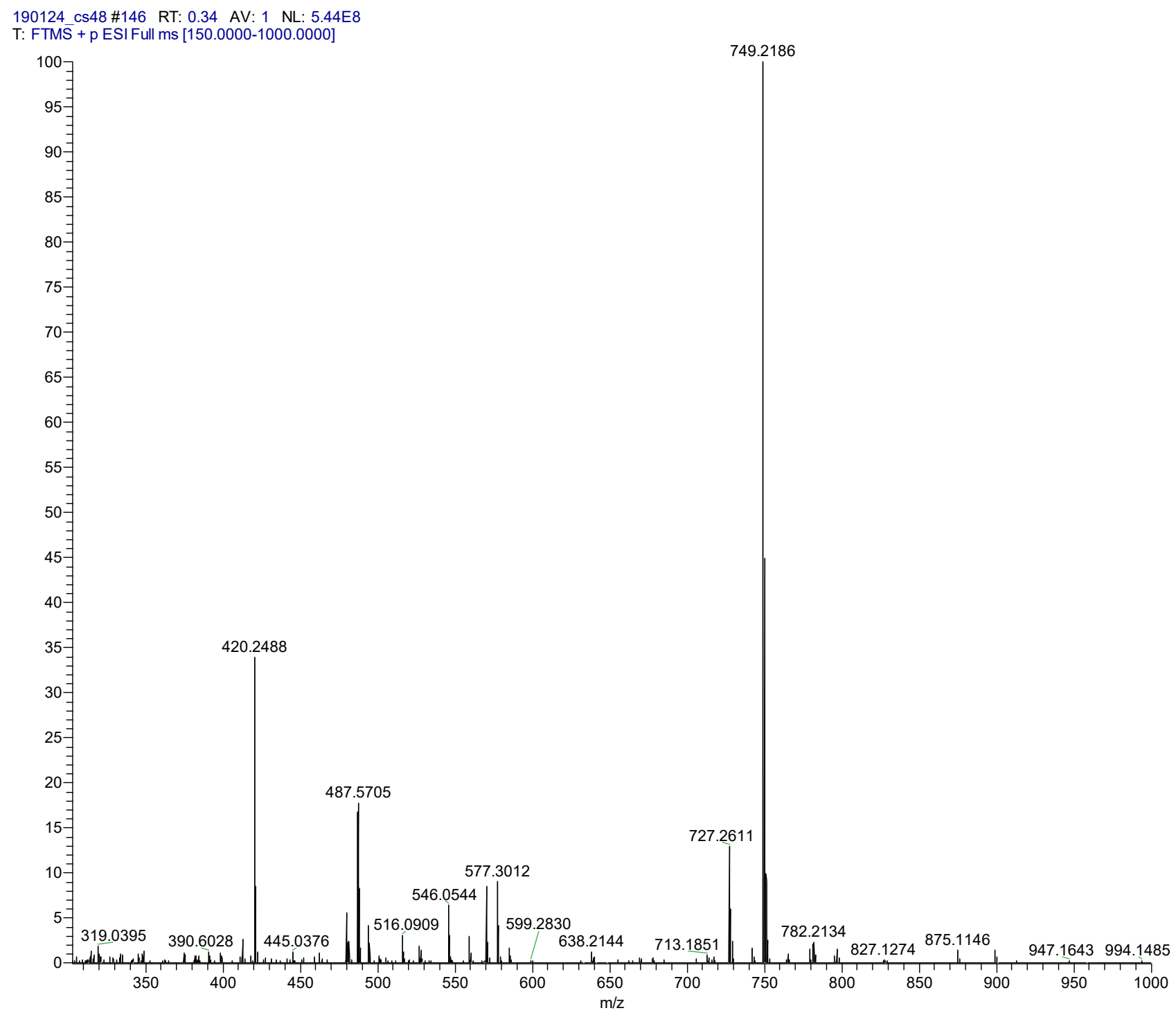


Figure S29. **HRMS(ES-TOF⁺)** of **[Lu-KHQ]⁺** *m/z* calcd. for $C_{32}H_{38}N_4LuO_6$ $[M]^+$ at 749.2199; found as 749.2186.

# 6. DFT

## 6.1. Experimental Details

Geometry optimizations using density functional theory (DFT) were performed in Gaussian16 (Revision C.01.).[8] To investigate the 1:1 complex of $Ln^{3+}$ with the **$H_2$KHQ** ligand in different conformational arrangements. Initial geometries for the *syn*-conformer were taken from the crystal structure of **[La-HKHQ]$^{2+}$** with removal of the H8 atom bound to O8 to model the overall +1 charged species. The $La^{3+}$ centre was replaced with other $Ln^{3+}$ ions to investigate the series. The hybrid $\omega$B97XD functional was employed,[9,10] which includes both dispersion and long-range corrections, and has been shown to give accurate geometries of $Ln^{3+}$ complexes.[11] Specifically For C, H, N and O atoms the def2svp (double $\zeta$) basis set was utilised.[12] For $Ln^{3+}$ ions the large-core relativistic effective core potentials (LCRECP) and associated (7s6p5d)/[5s4p3d] basis sets of Dolg *et al.*[13], were employed. The LCRECP calculation includes 4f electrons in the core (46 + $4f^n$), leaving the valence 5s, 5p, 5d, and 6s electrons to be considered explicitly, and thus all calculations were carried out in the pseudosinglet state. The use of LCRECP for other $Ln^{3+}$ complexes has been validated by previous studies[14,15], and is justified in that 4f orbitals do not have significant contribution to bonding. Geometry optimisations were performed without symmetry constrains (nosymm) and improved numerical integration grid using a pruned grid with 99 radial shells and 590 angular points per shell (int=ultrafine) was used. Additionally, frequency calculations were carried out on optimised structure to confirm, via the absence of imaginary frequencies, that the structures are local minima on the potential energy surface. To simulate the aqueous environment, single point energy corrections using the universal solvation model (SMD)[16] were carried out using water as the solvent, employing the parameterised PCM ionic radii of the $Ln^{3+}$ ions.[17]

Thermal corrections and solution phase corrections were applied using Paton's *GoodVibes* software,[18] which incorporates Grimme's quasi-harmonic approximation to the vibrational entropy below a cut-off of 100 $cm^{-1}$ and a frequency scaling factor = 1.0. The entropic terms for the frequencies below the cut-off are obtained from the free-rotor approximation while the standard rigid-rotor harmonic oscillator (RRHO) approximation is retained for those above the cut-off. A dampening function is used to interpolate between these two expressions close to the cut-off frequency. A temperature of 298 K and concentration of 1 M for **[Ln-KHQ]$^+$** and 55.5 M (concentration of pure water) for $H_2O$ was used to correct the gas-phase standard state free energies ($G°_{gas}$: 298 K, 1 atm) into solution-phase standard state free energies ($G°_{gas}$: 298 K, 1 mol/L) by the relationship with the ideal gas law (1 atm = 1/24.5 M at 298 K).

Interestingly, for macropa we do not see the conformational change reported by Roca-Sabio *et al.* as ionic radius decreases across the Ln series. Their work reports the La (III) favoured conformation to be Δ(δλδ)(δλδ), which then destabilizes for the rest of the series, resulting in the Δ(λδλ)(λδλ) conformation being most stable.[19] Herein, we have also calculated the optimised geometries for the 8 pairs of the three lanthanide ions discussed in this work with macropa**.** Our work demonstrates the Δ(δλδ)(δλδ) to be the lowest energy form for both **[Ln-KHQ]$^+$** and **[Ln-macropa]$^+$**. This may be an artifact of the different theory levels used between both studies, however they both confirm that there exists a number of accessible conformational modes in solution at room temperature.

## 6.2. Methods

The Gibbs free energies of **[*syn*-Ln-KHQ]$^+$**, **[*syn*-$H_2$O-Ln-KHQ]$^+$** and **[*anti*-Ln-KHQ]$^+$** were determined from DFT calculations. The values are summarized in Table S1. The Gibbs free energy difference between **[*syn*-Ln-KHQ]$^+$** and the hydrated form **[*syn*-$H_2$O-Ln-KHQ]$^+$** is calculated by:

$$\Delta G°_{hyd.} = G°_{aq}([\textit{syn}\text{-H}_2\text{O-Ln-KHQ}]^+) - G°_{aq}([\textit{syn}\text{-Ln-KHQ}]^+) - G°_{l}(H_2O) \qquad \textbf{Equation S1}$$

The Gibbs energy difference between **[*syn*-Ln-KHQ]$^+$** and **[*anti*-Ln-KHQ]$^+$** conformations is calculated by:

$$\Delta G°_{conf.} = G°_{aq}([\textit{anti}\text{-Ln-KHQ}]^+) - G°_{aq}([\textit{syn}\text{-Ln-KHQ}]^+) \qquad \textbf{Equation S2}$$

For the Gibbs energy of complexation:

$$\Delta G°_{[\text{Ln-L}]^+} = G°_{gas}([\textit{syn}\text{-Ln-L}]^+) - G°_{gas}(\text{Ln}^{3+}) - G°_{gas}(\text{L}^{2-}) \qquad \textbf{Equation S3}$$

Where L = KHQ or macropa

The term G°$_g$(**L$^{2-}$**) was determined from a geometry optimisation of an initial guess structure based on the optimised structure of **[*syn*-La-L]$^+$** and removing the $La^{3+}$ centre. The optimised structure was then verified as a local minimum via a frequency calculation. Whilst the optimised structure of **L$^{2-}$** may not represent the global minimum, (and in reality **H$_2$KHQ** and H$_2$macropa will exist in different protonation states depending on pH), the value of G°$_g$(**L$^{2-}$**) remains constant for the calculation of ΔG°$_{[Ln-L]}$ for all Ln ions, and therefore relative comparisons and observed trends are valid.

Table S2. Free energies in aqueous solution (G°$_{aq.}$, kcal mol$^{-1}$) of DFT optimised structures and the Gibbs free energy changes (ΔG°$_{aq.}$, kcal mol$^{-1}$) described by equations S1 and S2. a: G°$_l$($H_2O$) = -47901.18 kcal mol$^{-1}$.

| | *G°$_{aq.}$(syn-Ln-KHQ)* | *G°$_{aq.}$(anti-Ln-KHQ)* | *ΔG°$_{conf.}$* | *G°$_{aq.}$(syn-H$_2$O-Ln-KHQ)* | *ΔG°$_{hyd.}$$^a$* | *G°$_{aq.}$(Ln$^{3+}$)* |
|---|---|---|---|---|---|---|
| *La* | -1218165.73 | -1218161.53 | + 4.2 | -1266054.31 | + 12.6 | -19753.40 |
| *Ce* | -1218564.79 | -1218559.91 | + 4.9 | -1266452.41 | + 13.6 | -20161.55 |
| *Pr* | -1218965.48 | -1218961.53 | + 4.0 | -1266854.19 | + 12.5 | -20563.87 |
| *Nd* | -1219352.65 | -1219348.62 | + 4.0 | -1267241.07 | + 12.8 | -20950.34 |
| *Sm* | -1220119.30 | -1220114.51 | + 4.8 | -1268005.42 | + 15.1 | -21719.57 |
| *Eu* | -1220493.50 | -1220489.08 | + 4.4 | -1268379.99 | + 14.7 | -22094.39 |
| *Gd* | -1220865.56 | -1220861.21 | + 4.4 | -1268752.61 | + 14.1 | -22465.58 |
| *Tb* | -1221233.96 | -1221229.29 | + 4.7 | -1269120.39 | + 14.8 | -22833.59 |
| *Dy* | -1221601.45 | -1221596.79 | + 4.7 | -1269488.29 | + 14.3 | -23204.94 |
| *Ho* | -1221965.24 | -1221960.06 | + 5.2 | -1269851.99 | + 14.4 | -23566.13 |
| *Er* | -1222330.72 | -1222326.64 | + 4.1 | -1270217.18 | + 14.7 | -23933.93 |
| *Tm* | -1222695.47 | -1222690.88 | + 4.6 | -1270582.07 | + 14.6 | -24300.18 |
| *Yb* | -1223061.91 | -1223056.63 | + 5.3 | -1270949.94 | + 13.1 | -24668.44 |
| *Lu* | -1223407.64 | -1223402.94 | + 4.7 | -1271296.02 | + 12.8 | -25014.88 |

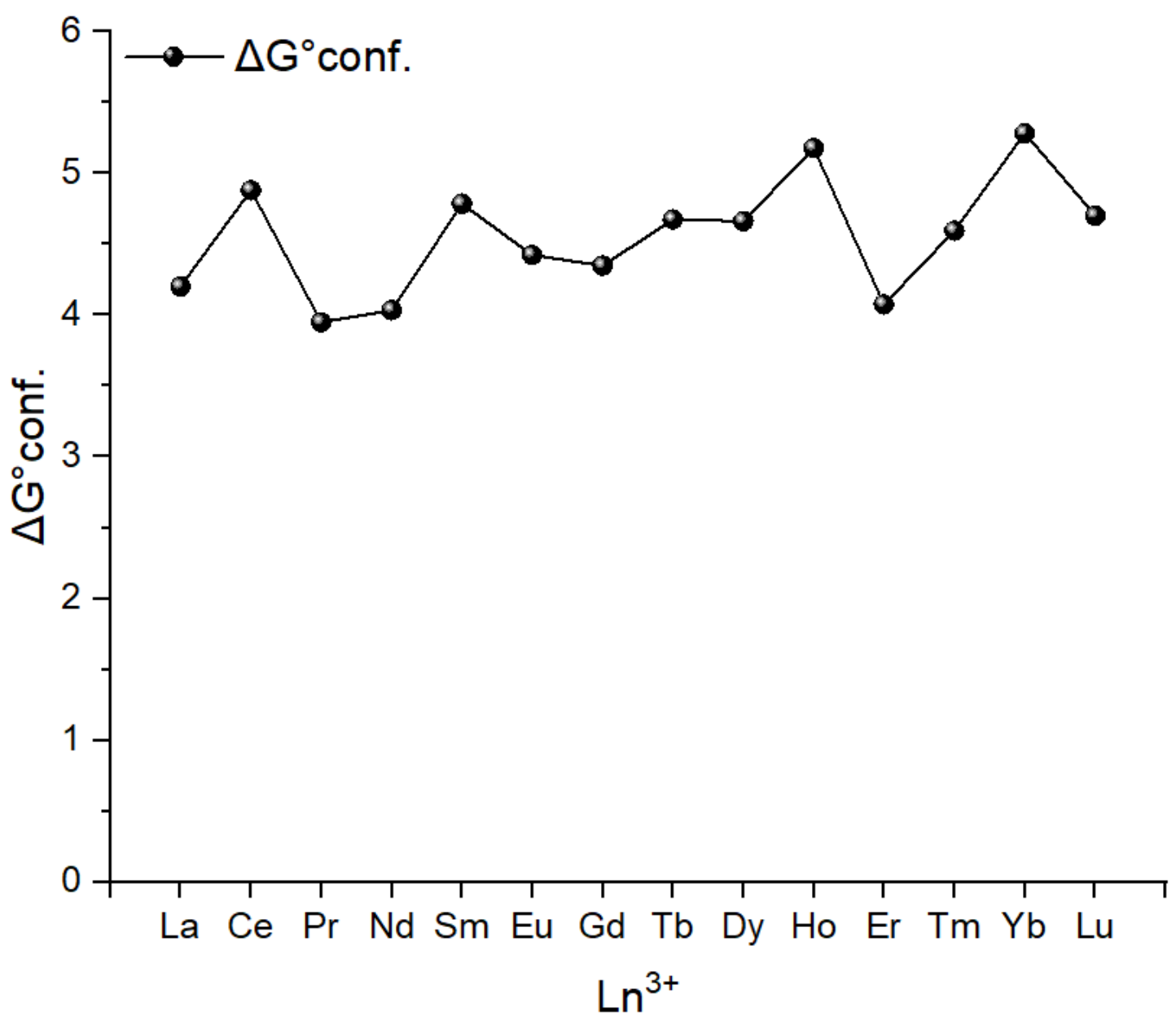


Figure S30. DFT-computed Gibbs free energy difference for the anti/syn conformation equilibrium of **[Ln-KHQ]$^+$** complexes.

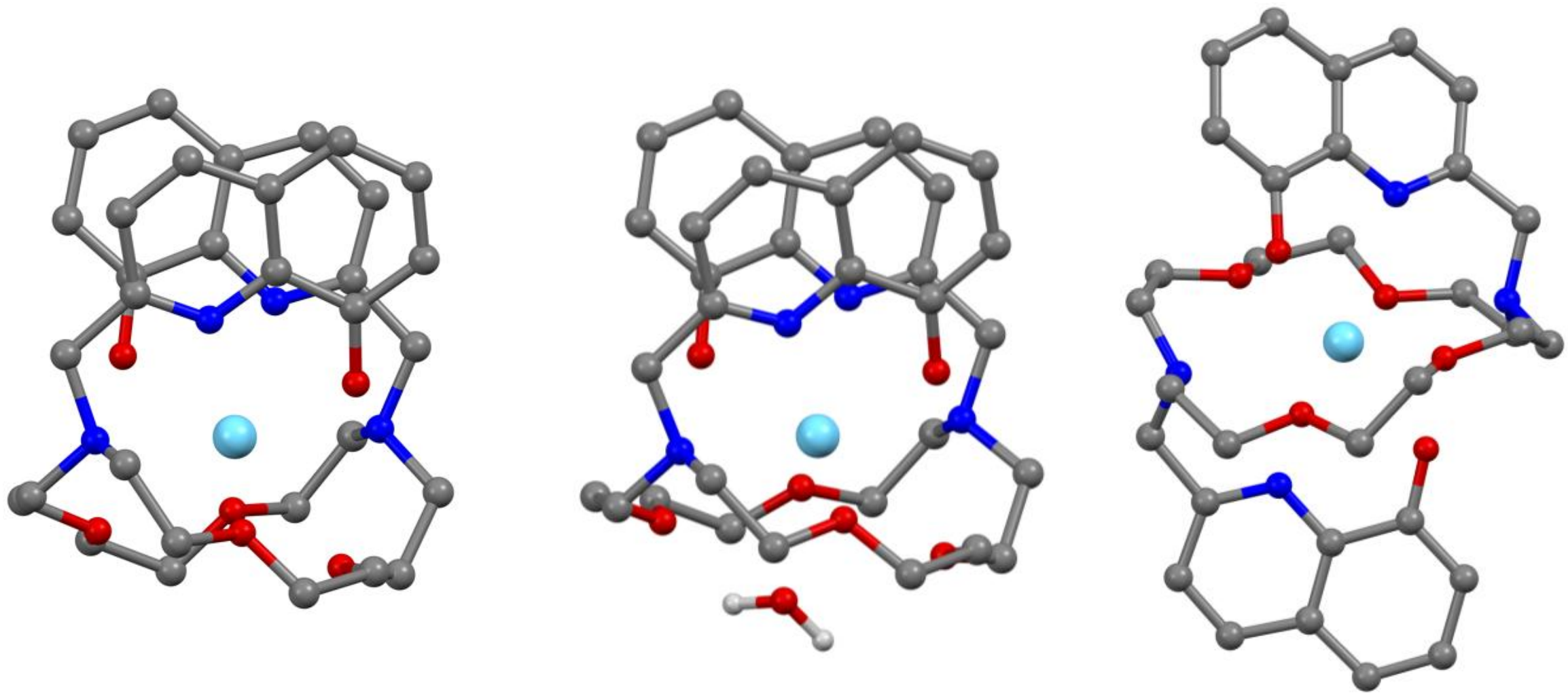

Figure S31. Left to right: **[syn-La-KHQ]⁺**, **[syn-$H_2$O-La-KHQ]⁺**, and **[anti-La-KHQ]⁺** complexes obtained from DFT calculations.

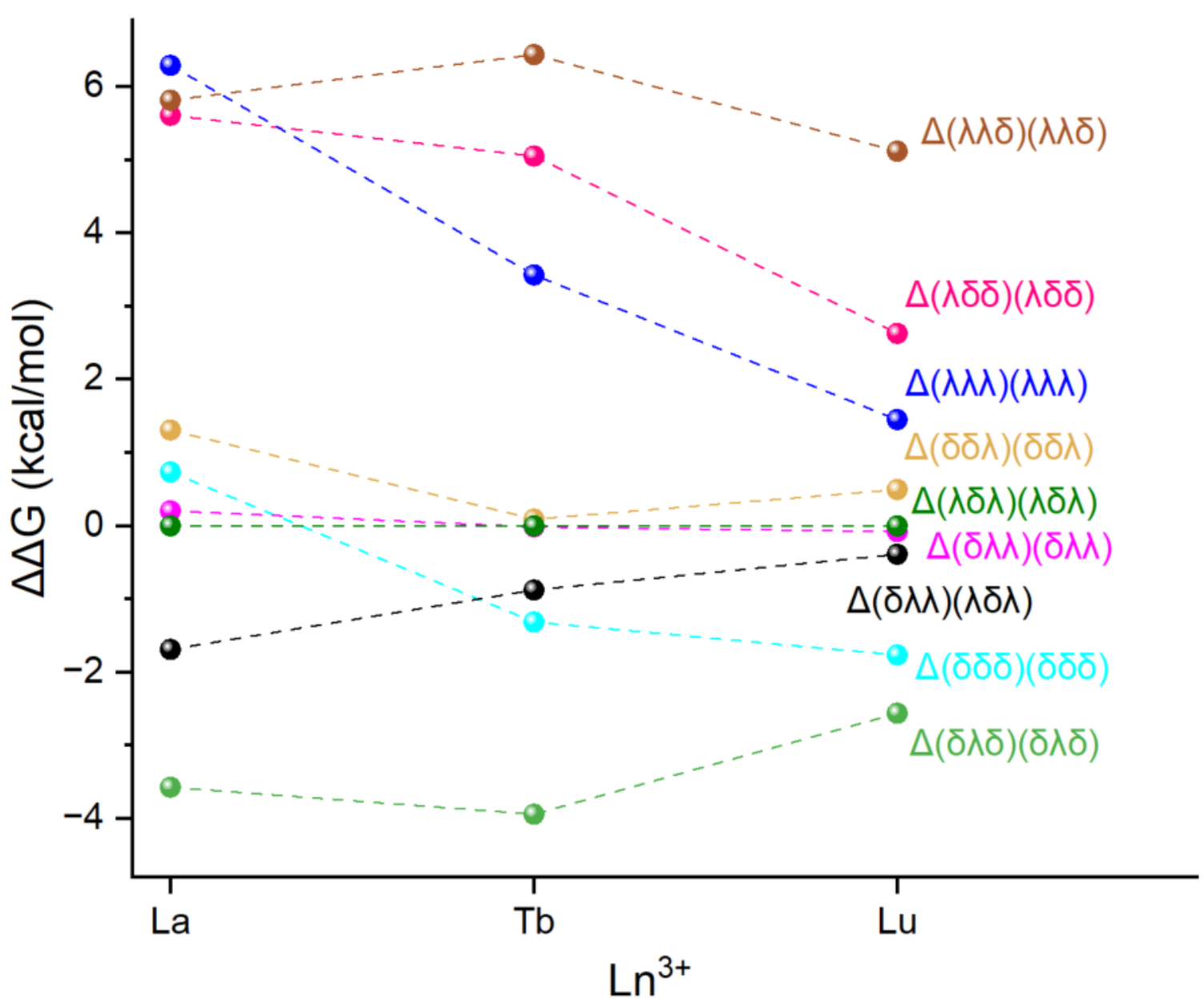


Figure S32. Comparison of relative Gibbs free energies of conformational modes in [Ln-KHQ]⁺. ΔΔG values relative to Δ(λδλ)(λδλ) conformation.

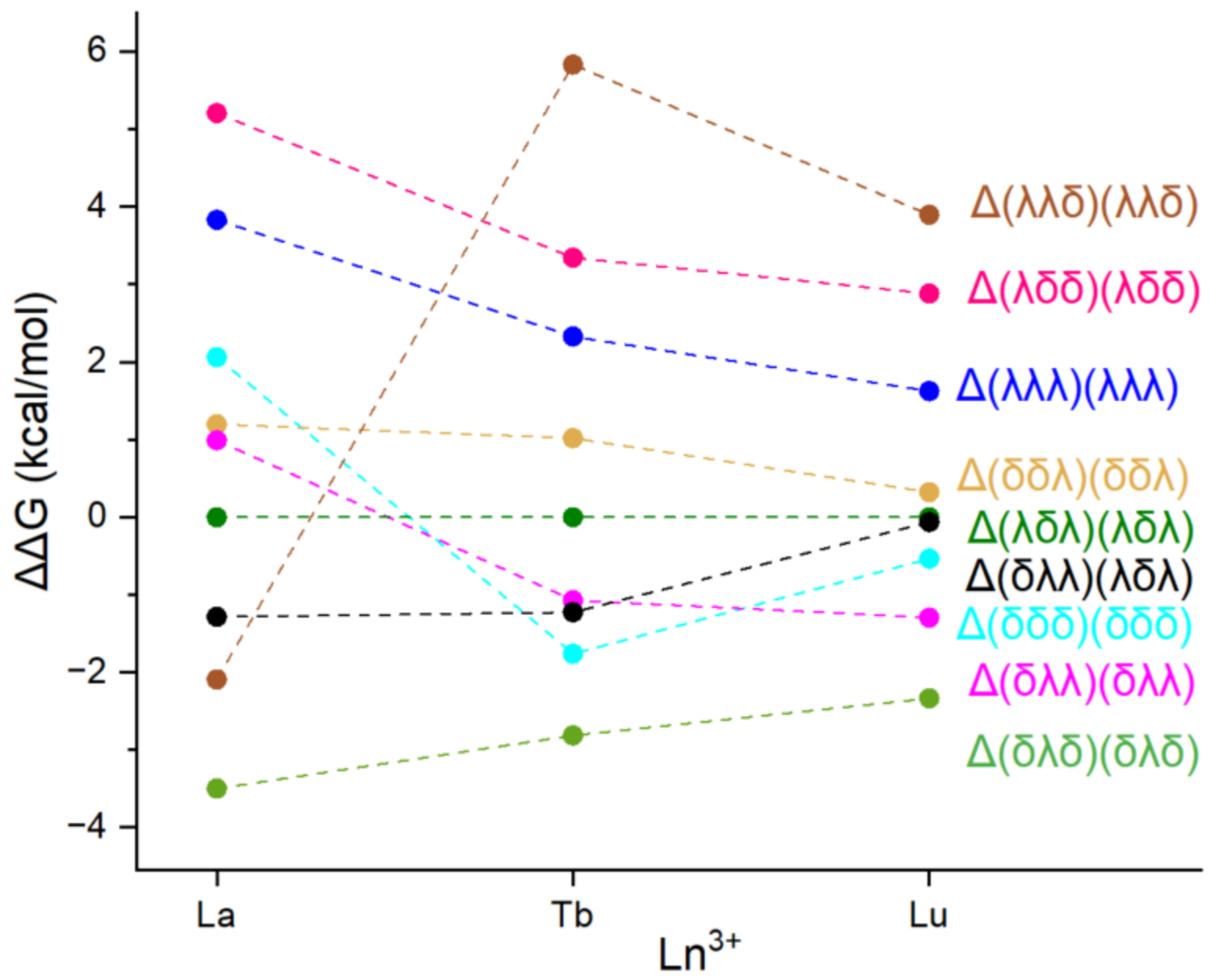


Figure S33. Comparison of relative Gibbs free energies of conformational modes in [Ln-macropa]⁺. ΔΔG values relative to Δ(λδλ)(λδλ) conformation.

## 6.3. Ligand Strain Analysis

Ligand strain energy $\Delta G_s°$ (gas phase standard state) is the standard free energy required to change the free ligand from the relaxed (geometry optimised) conformation, $G°_g$(**L**$^{2-}$), to the conformation found in the $Ln^{3+}$ complex of either KHQ or Macropa in the gas phase, $G°_g$(**L**$_{\mathbf{complex}}^{2-}$). $G°_g$(**L**$_{\mathbf{complex}}^{2-}$) is determined from single point frequency calculations of the frozen ligand structure as found in the metal complex, where the $Ln^{3+}$ ion is removed from the optimised structures of **[Ln-L]⁺** (where L = KHQ or Macropa). It is clear in **Figure S34**, that as the size of the $Ln^{3+}$ ion decreases, ligand strain increases due to a higher degree of folding in the azamacrocyclic ring as the oxygen atoms move closer to accommodate the smaller central metal ion.

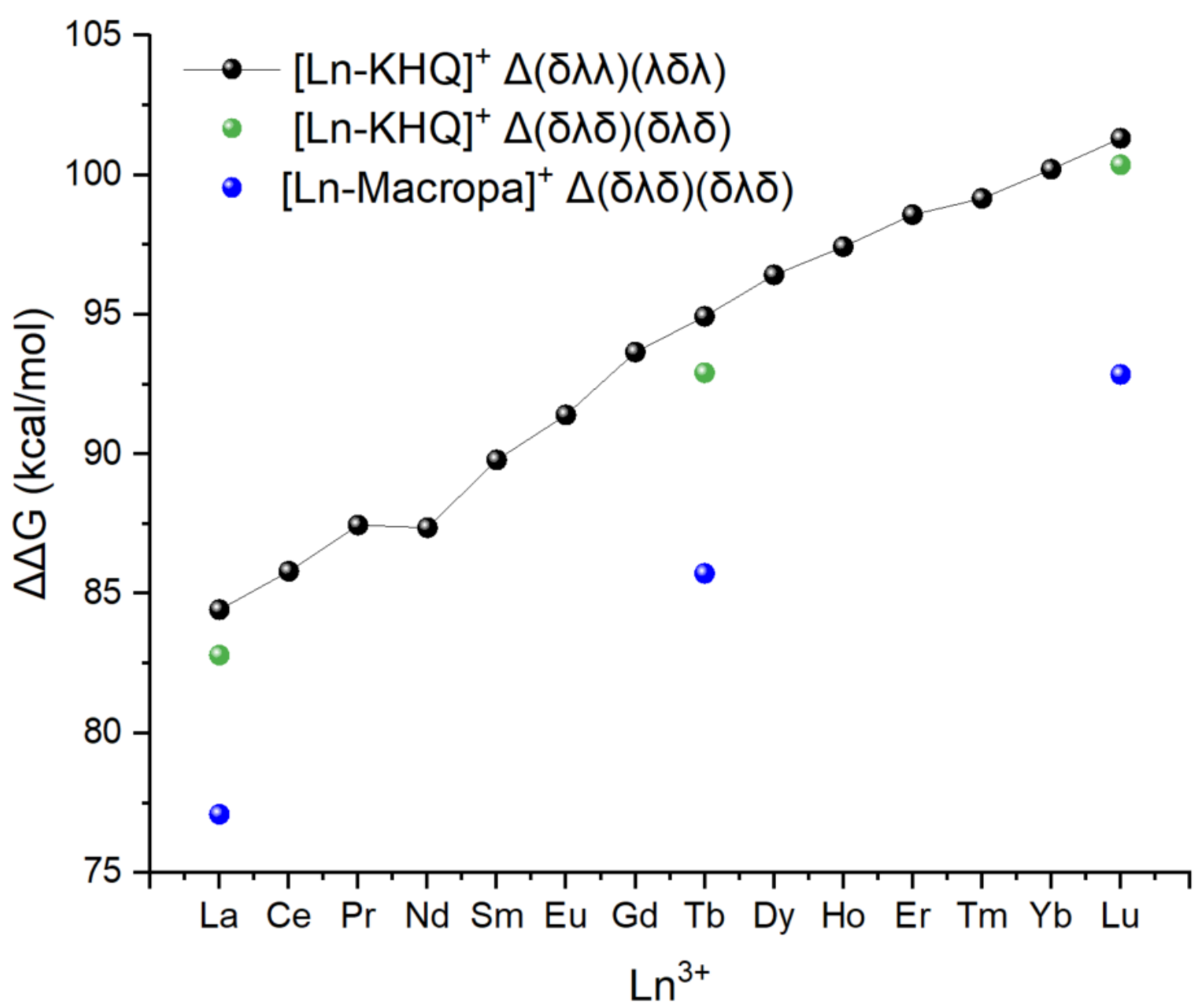


Figure S34. DFT-computed ligand strain energy for **[Ln-KHQ]⁺** and [Ln-Macropa]⁺ complexes.

Table S3. Relative Gibbs free energies for the studied ring conformations of both KHQ and Macropa

| Conformation | $H_2KHQ$, ΔΔG (kcal/mol) | | | Macropa, ΔΔG (kcal/mol) | | |
|---|---|---|---|---|---|---|
| | La | Tb | Lu | La | Tb | Lu |
| Δ(δδδ)(δδδ) | 0.735189544 | -1.3133199 | -1.7640094 | 2.06274131 | -1.7601627 | -0.5357797 |
| Δ(δδλ)(δδλ) | 1.309692859 | 0.09077545 | 0.4966671 | 1.197036169 | 1.01849103 | 0.32037472 |
| Δ(δλδ)(δλδ) | -3.57297977 | -3.9382088 | -2.5607074 | -3.497565739 | -2.8128844 | -2.3351806 |
| Δ(δλλ)(δλλ) | 0.203833748 | -0.0140939 | -0.0771585 | 0.990742585 | -1.0707186 | -1.2937479 |
| Δ(λδδ)(λδδ) | 5.608248736 | 5.05240126 | 2.62835282 | 5.205475809 | 3.34198116 | 2.88110717 |
| Δ(λδλ)(λδλ) | 0 | 0 | 0 | 0 | 0 | 0 |
| Δ(λλδ)(λλδ) | 5.815107078 | 6.43620921 | 5.11767475 | -2.091142367 | 5.83131564 | 3.89660499 |
| Δ(λλλ)(λλλ) | 6.29180684 | 3.42818834 | 1.45152244 | 3.831136973 | 2.32746226 | 1.62379249 |
| Δ(δλλ)(λδλ) | -1.687735656 | -0.8772639 | -0.3900533 | -1.284743101 | -1.2262216 | -0.0648782 |

Table S4. Gas phase comparison of Gibbs free energies of complexation (Equation S3) of $[Ln\text{-}macropa]^+$ and $[Ln\text{-}KHQ]^+$. Values given in kcal/mol. ΔΔG = ΔG°[Ln-KHQ] – ΔG°[Ln-Macropa]

| $Ln^{3+}$ | $\Delta G°_{[Ln\text{-}KHQ]+}$ | $\Delta G°_{[Ln\text{-}Macropa]+}$ | ΔΔG |
|---|---|---|---|
| La | -977.5103024 | -978.602168 | -1.0918657 |
| Tb | -1029.790587 | -1029.894754 | -0.1041665 |
| Lu | -1060.424322 | -1061.302207 | -0.8778851 |

# 7. Potentiometric titrations

## 7.1. Experimental Data

Potentiometric titrations were carried out using a Metrohm Titrando 888 titrator equipped with a combined glass electrode (Metrohm model 6.0260.010) and a Metrohm 801 Stirrer unit. This titration system was controlled by *Tiamo* (ver. 2.34) software. The titration vessel was fitted into a removable glass cell (≈ 70 mL) and thermostated at 25.0 °C (p*K*w(NaCl, 0.1 M) = 13.78)[20] using a circulating water bath. $CO_2$ was excluded from the vessel prior to and during the titrations by a nitrogen flow, which was passed through an aqueous solution. NaOH (0.1 M) was standardized by potentiometric titration against potassium hydrogen phthalate. Hydrochloric acid (0.1 M) was standardized against Tris (base form). Titration solutions were maintained at a constant ionic strength of 0.1 M with NaCl (prepared by adding 5.8 g NaCl to 1 L de-ionised $H_2O$). The electrode was calibrated before each titration by titrating a solution of standardized HCl (0.1 M) with standardized NaOH (0.1 M), and the data were analysed using the program *GLEE*5 (ver. 3.0.21)[21] to obtain the standard electrode potential and slope factor.

$Ln^{3+}$ stock solutions were made by dissolving the corresponding chloride salts (98 % purity or higher) in $H_2O$ or standardized HCl (0.1 M, for $La^{3+}$ only). The exact concentrations were determined by complexometric titrations[22] with a standardized 0.01 M EDTA solution . The complexometric titrations were performed at pH 5.4 maintained by a HOAc-NaOAc buffer, and the end point was indicated by xylenol orange. Ligand aliquots were added directly into the electrolyte solution prior to analysis.

Protonation constant or stability constant determinations were carried out by titrating an acidic solution containing free ligand or both ligand and metal (in 30 mL 0.1 M NaCl ) with standardized 0.1 M NaOH. For protonation constant determinations, the ligand concentration was 0.018 mmol. For stability constant determinations, both the ligand and metal ion concentrations were 0.018 mmol. The solutions were inspected throughout the titrations for signs of $Ln(OH)_3$ precipitation. Titration data were refined with *Hyperquad 2013*[23] software to determine protonation and stability constants. The data points within the pH range of 3 – 10.5 were used for analysis. At least three independent titrations for protonation constant measurements and three titrations for stability constant

measurements were carried out for each L and LnL system. Within the data fitting models for the [Ln-KHQ]$^+$ complexes, we included the deprotonated complex species ($LnLH_{-1}$, Figure S31-34), which was necessary to obtain robust fit and values for log$K_{LnL}$.

Equilibration between the addition of NaOH aliquots was monitored via the electrode potential. If the system is not at equilibrium, the electrode potential will drift as the system continues to adjust. To ensure that the thermodynamic equilibration is reached after an aliquot of NaOH is added, the next NaOH aliquot was not added until either the electrode drifting is slow (< 0.2 mV/min), or a maximum time limit is reached (2 min for both protonation constant determinations and stability constant determinations).

### 7.2. Supplementary Data

$$K_{ai} = \frac{[H_iL]}{[H^+][H_{i-1}L]}$$

Equation S4. Ligand protonation constants ($K_{ai}$). L = fully deprotonated KHQ, i = number of protons

$$K_{LnL} = \frac{[LnL]}{[Ln^{3+}][L]}$$

Equation S5. Complex stability constants ($K_{LnL}$). L = fully deprotonated KHQ, Ln = La, Tb, Lu

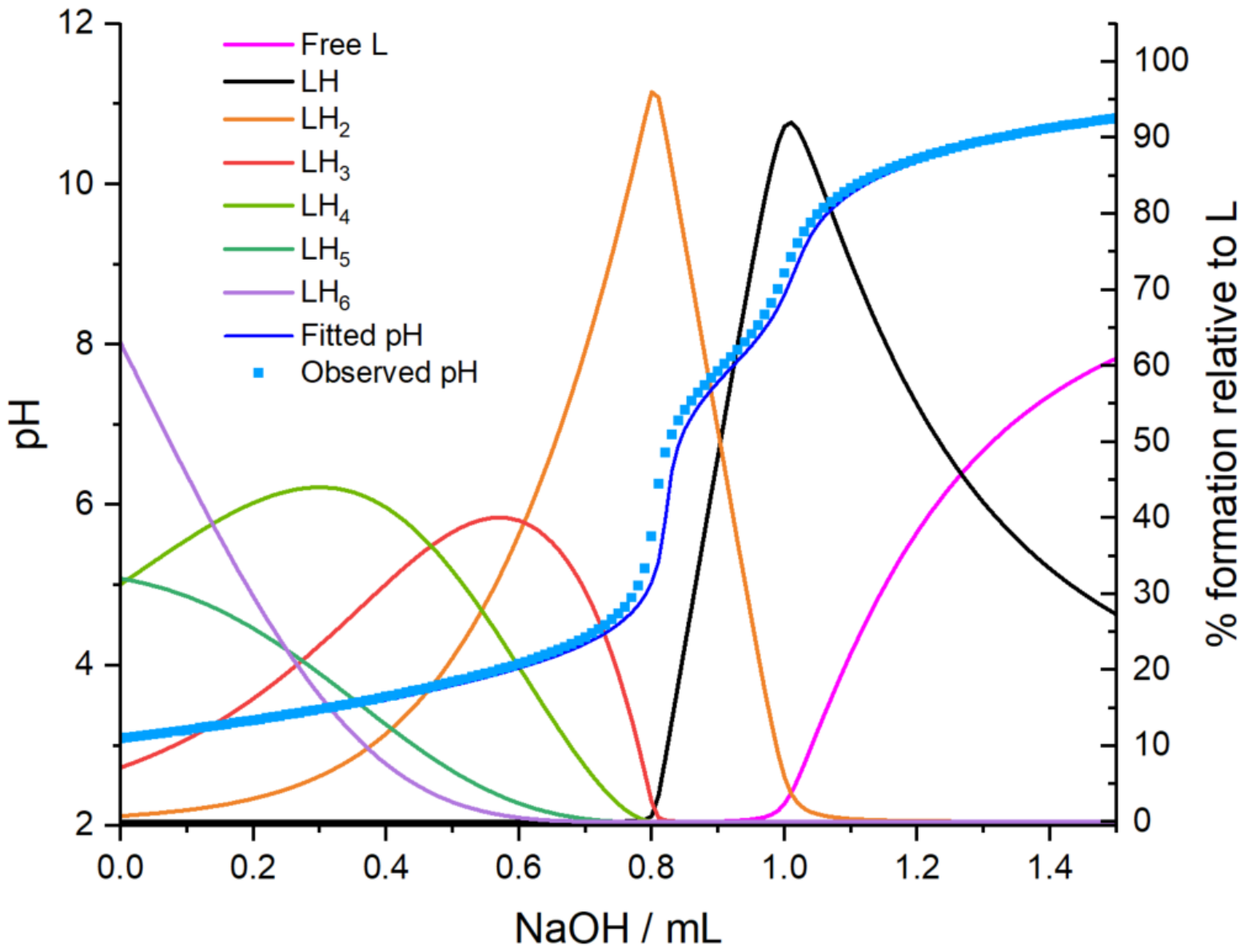


Figure S35. Representative protonation constant determination of **$H_2$KHQ** modelled in HySS. **$H_2$KHQ** *c* = 0.018 mM. Initial volume V = 30 mL. Data fitting and speciation distribution over the pH range shown.

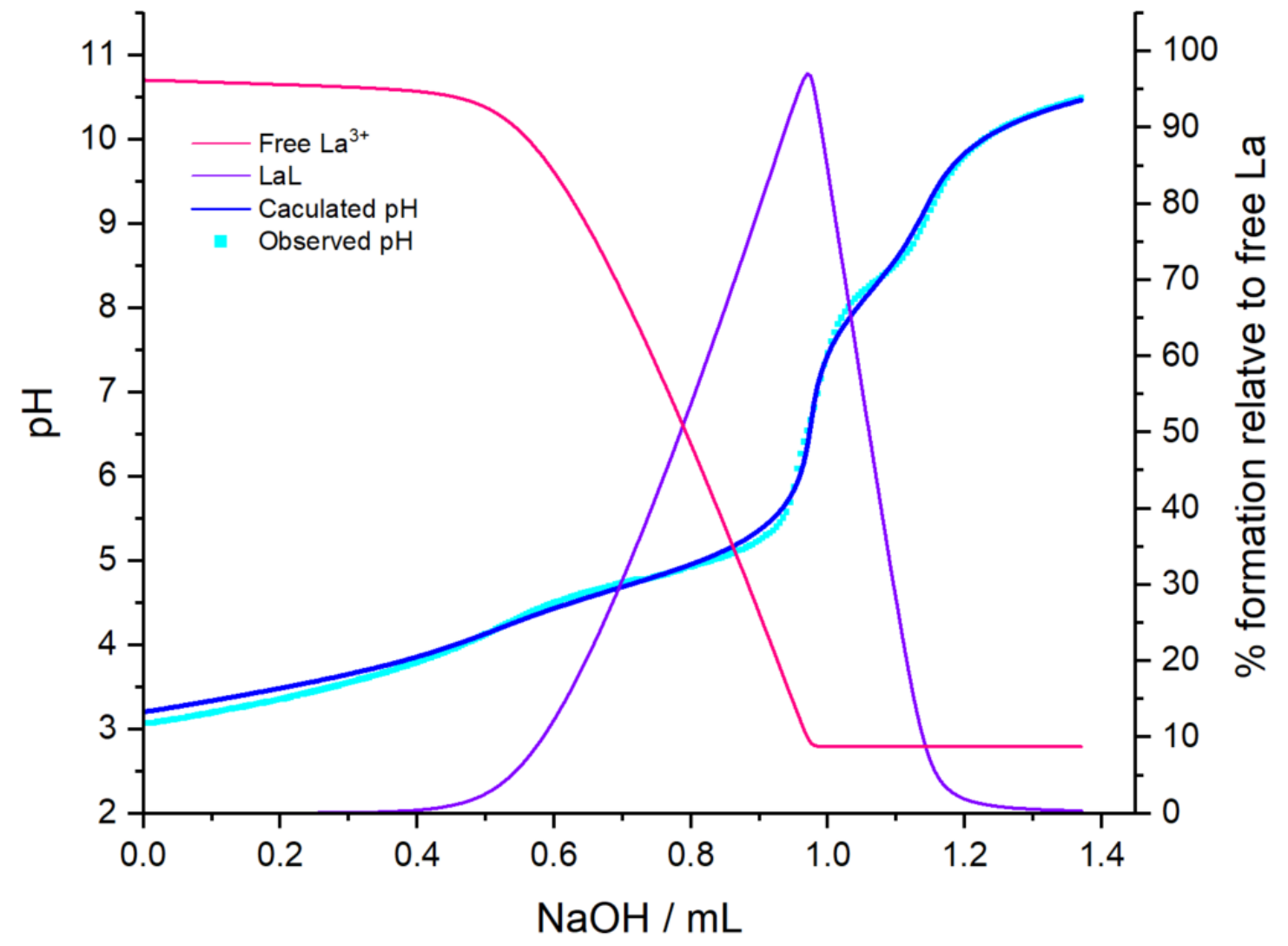


Figure S36. Representative stability constant determination of **[La-KHQ]**$^+$ modelled in HySS. **$H_2$KHQ** $c$ = 0.018 mM, **$La^{3+}$** $c$ = 0.018 mM. Initial volume V = 30 mL. Data fitting and speciation distribution over the pH range shown.

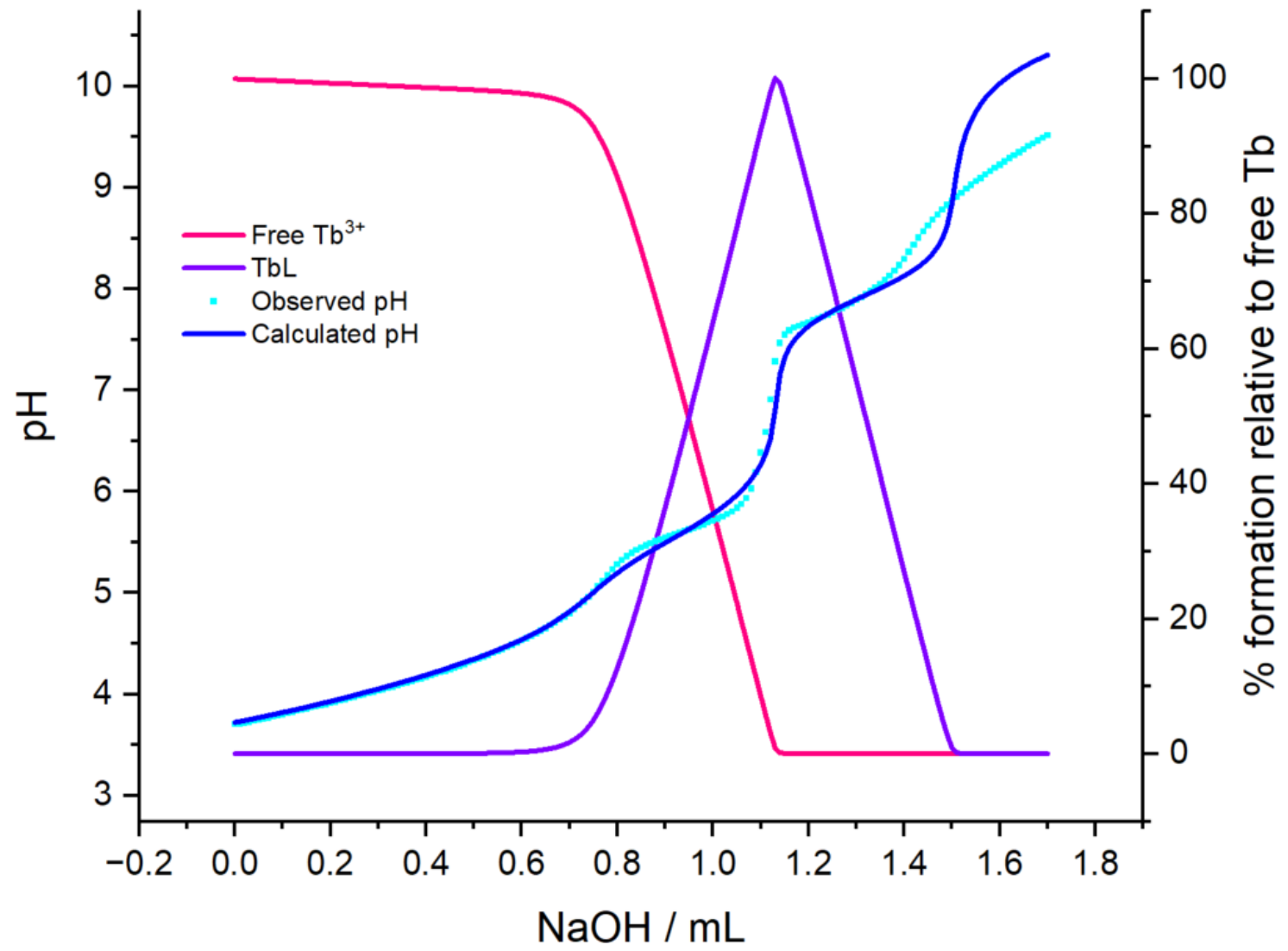


Figure S37. Representative stability constant determination of **[Tb-KHQ]**$^+$ modelled in HySS. **$H_2$KHQ** $c$ = 0.018 mM, **$Tb^{3+}$** $c$ = 0.018 mM. Initial volume V = 30 mL. Data fitting and speciation distribution over the pH range shown.

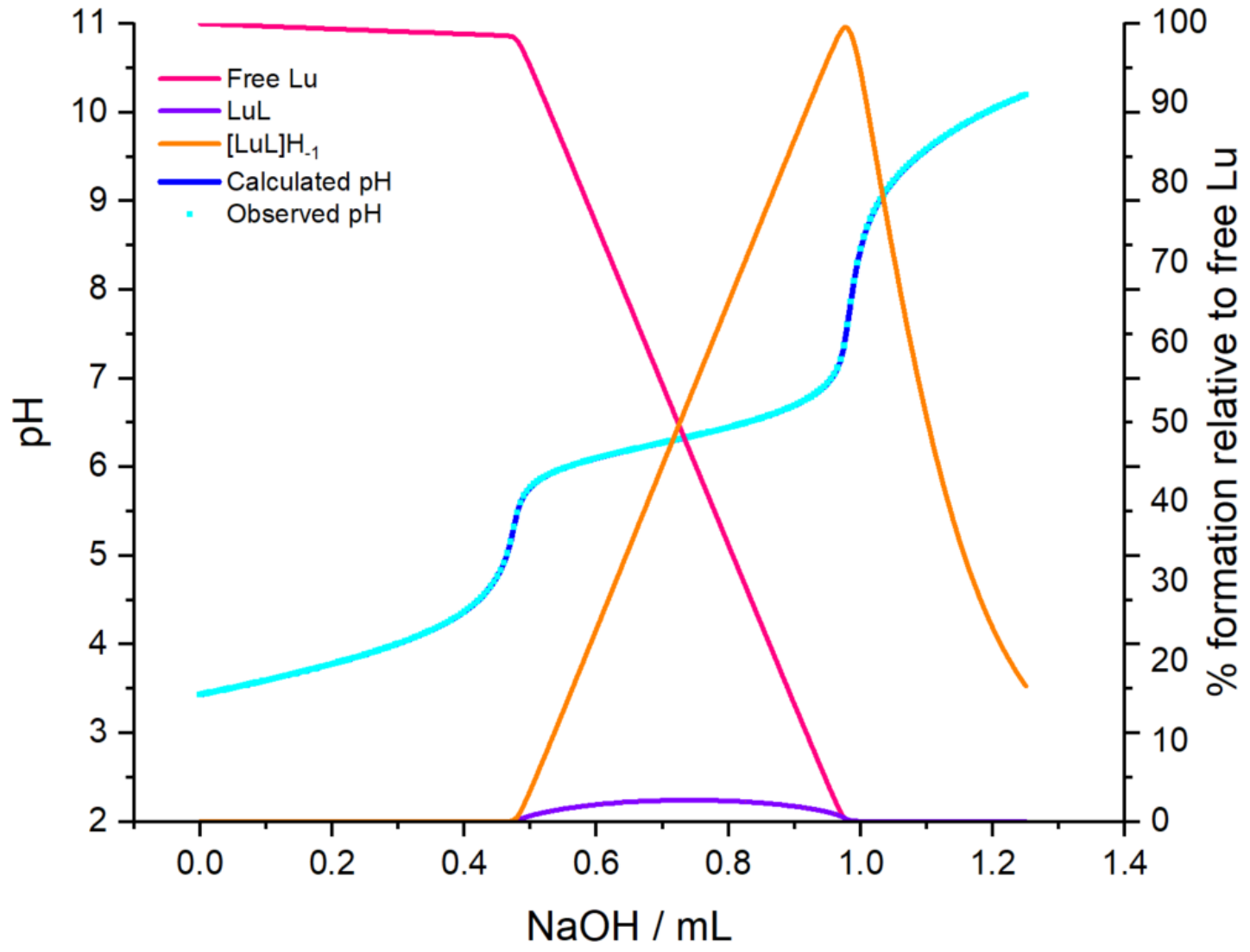


Figure S38. Representative stability constant determination of **[Lu-KHQ]**$^{+}$ modelled in HySS. **$H_2$KHQ** $c$ = 0.018 mM, **$Lu^{3+}$** $c$ = 0.018 mM. Initial volume V = 30 mL. Data fitting and speciation distribution over the pH range shown.

## 8. UV-Vis Spectroscopy and Phosphorescence

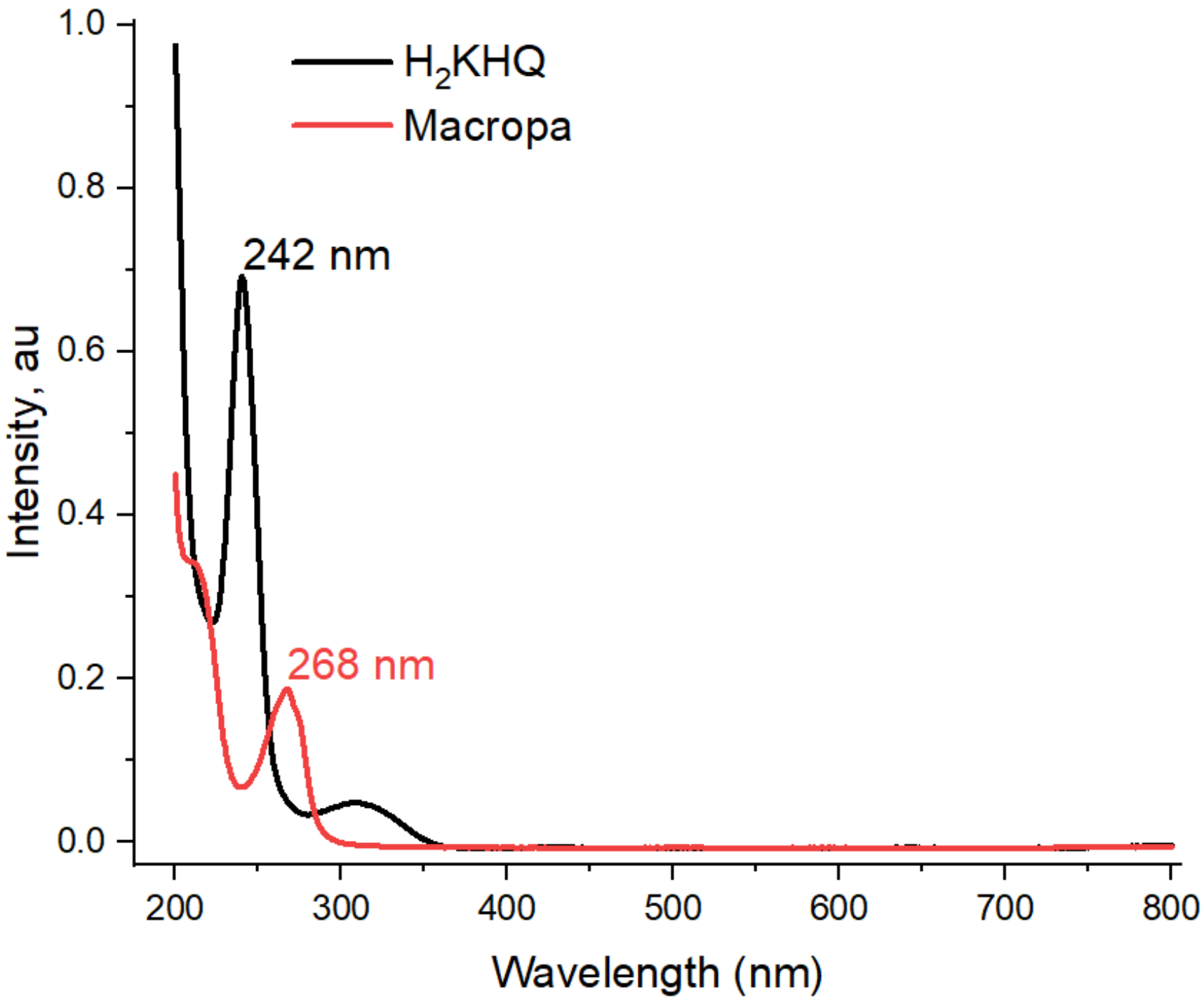


Figure S39. UV-Vis spectrum showing the absorption wavelengths of **$H_2$KHQ** and **macropa**. (20 µM, 10 nm slits, $H_2O$).

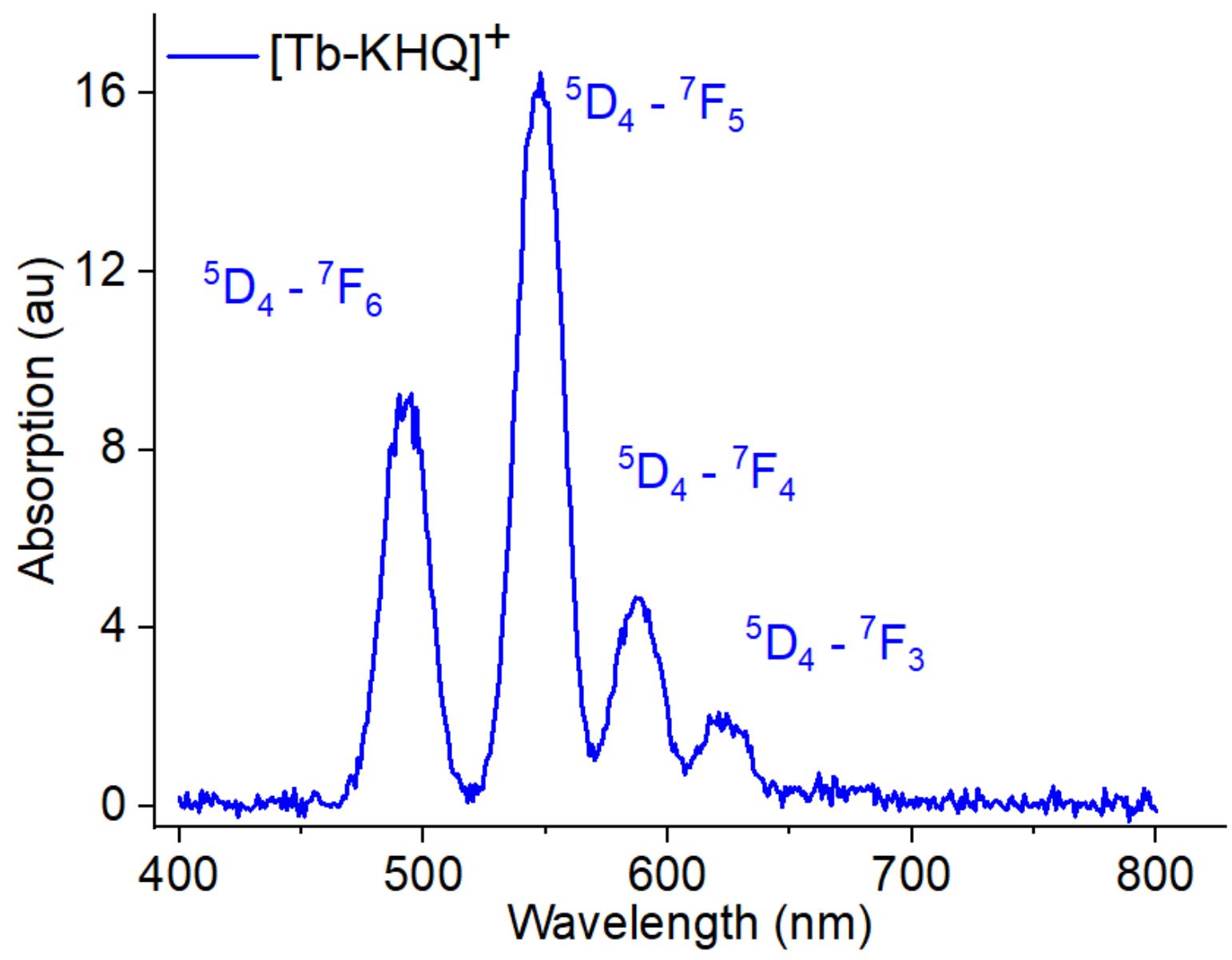


Figure S40. Phosphorescence spectrum for **[Tb-KHQ]**$^+$ ($H_2O$, 20 µM, 20 nm slits, 0.1 ms delay).

# 9. Radiolabelling

## 9.1. Experimental Details

**Caution:** $^{161}Tb$ emits ionizing radiation and should only be used in a facility in accordance with appropriate safety controls.

Radiolabelling studies of **H$_2$KHQ** with $^{161}Tb^{3+}$ were performed to establish suitable labelling conditions. A 2.5 mM stock solution of **H$_2$KHQ** was made by dissolving **H$_2$KHQ** in 20 mM $NH_4OAc$ buffer (20 mM, pH 8.5). For labelling experiments, [$^{161}$Tb]$Tb^{3+}$ (1 mM HCl, 2.5 MBq, 10 µL) was added to a solution of **H$_2$KHQ** (concentration 1 mM). $V_T$ = 100 µL. Reactions were allowed to stand at room temperature or 90 ˚C for 20 min – 180 min and then monitored by radio-HPLC using mobile phase A: 2mM $NH_4OAc$ in $H_2O$ and mobile phase B: 5 % 2 mM $NH_4OAc$ in $H_2O$ in 95 % MeCN. Gradients are as shown below. Analytical reverse-phase radio-HPLC was performed on an Agilent 1200 Series Liquid Chromatograph (with UV and gamma detectors) using an Agilent Eclipse XDB-C18 column (4.6 × 150 mm, 5 µm) with a 1 mL/min$^{-1}$ flow rate and UV spectroscopic detection at 254 nm. Under these conditions **[$^{161}$Tb][Tb-KHQ]$^+$** eluted at 18.33 min while free [$^{161}$Tb]$Tb^{3+}$ eluted during the aqueous phase washes at 1.33 and 29.30 min.

| Gradient, min | A, % | B, % |
|---|---|---|
| 0.00 | 95 | 5 |
| 5.00 | 95 | 5 |
| 30.00 | 5 | 95 |
| 30.10 | 95 | 5 |
| 33.00 | 95 | 5 |

Radiolabelling studies of **macropa** with $^{161}Tb^{3+}$ were performed to establish suitable labelling conditions. A 10 mM stock solution of **macropa** was made by dissolving **macropa** in 200 mM $NH_4OAc$ buffer (20 mM, pH 6.5). For labelling experiments, [$^{161}$Tb]$Tb^{3+}$ (2 mM HCl, 5 MBq, 10 µL) was added to a solution of **macropa** (concentration 1 mM). $V_T$ = 100 µL. Reactions were allowed to stand at room temperature or 90 ˚C for 30 min and then monitored by radio-HPLC using mobile phase A: 2mM $NH_4OAc$ in $H_2O$ and mobile phase B: 5 % 2 mM $NH_4OAc$ in $H_2O$ in 95 % MeCN. Gradients are as shown below. Analytical reverse-phase radio-HPLC was performed on an Agilent 1200 Series Liquid Chromatograph (with UV and gamma detectors) using an Agilent Eclipse XDB-C18 column (4.6 × 150 mm, 5 µm) with a 1 mL/min$^{-1}$ flow rate and UV spectroscopic detection at 254 nm. Under these conditions **[$^{161}$Tb][Tb-macropa]$^+$** eluted at 11.1 min while free [$^{161}$Tb]$Tb^{3+}$ eluted during the aqueous phase washes at 1.37and 32.50 min.

| Gradient, min | A, % | B, % |
|---|---|---|
| 0.00 | 95 | 5 |
| 5.00 | 95 | 5 |
| 25.00 | 5 | 95 |
| 30.00 | 5 | 95 |
| 32.000 | 95 | 5 |
| 35.00 | 95 | 5 |

## 9.2. Supplementary Data

Table S5. The RCY values for non-carried added radiolabelling of **$H_2$KHQ** with $^{161}$Tb[$Tb^{3+}$] in $NH_4$OAc buffer (20 mM, pH 8.5) at room temperature and 90 ˚C for 20 min – 180 min.

| **Time, min** | **Temperature** | **RCY, %** |
|---|---|---|
| 20 | Room temperature | 33 |
| 180 | Room temperature | 68 |
| 20 | 90 ˚C | 92 |

Table S6. The RCY values for non-carried added radiolabelling of **macropa** with $^{161}$Tb[$Tb^{3+}$] in $NH_4$OAc buffer (200 mM, pH 6.5) at room temperature and 90 ˚C for 20 min – 180 min.

| **Time, min** | **Temperature** | **RCY, %** |
|---|---|---|
| 30 | Room temperature | 62 |
| 30 | 90 ˚C | 55 |

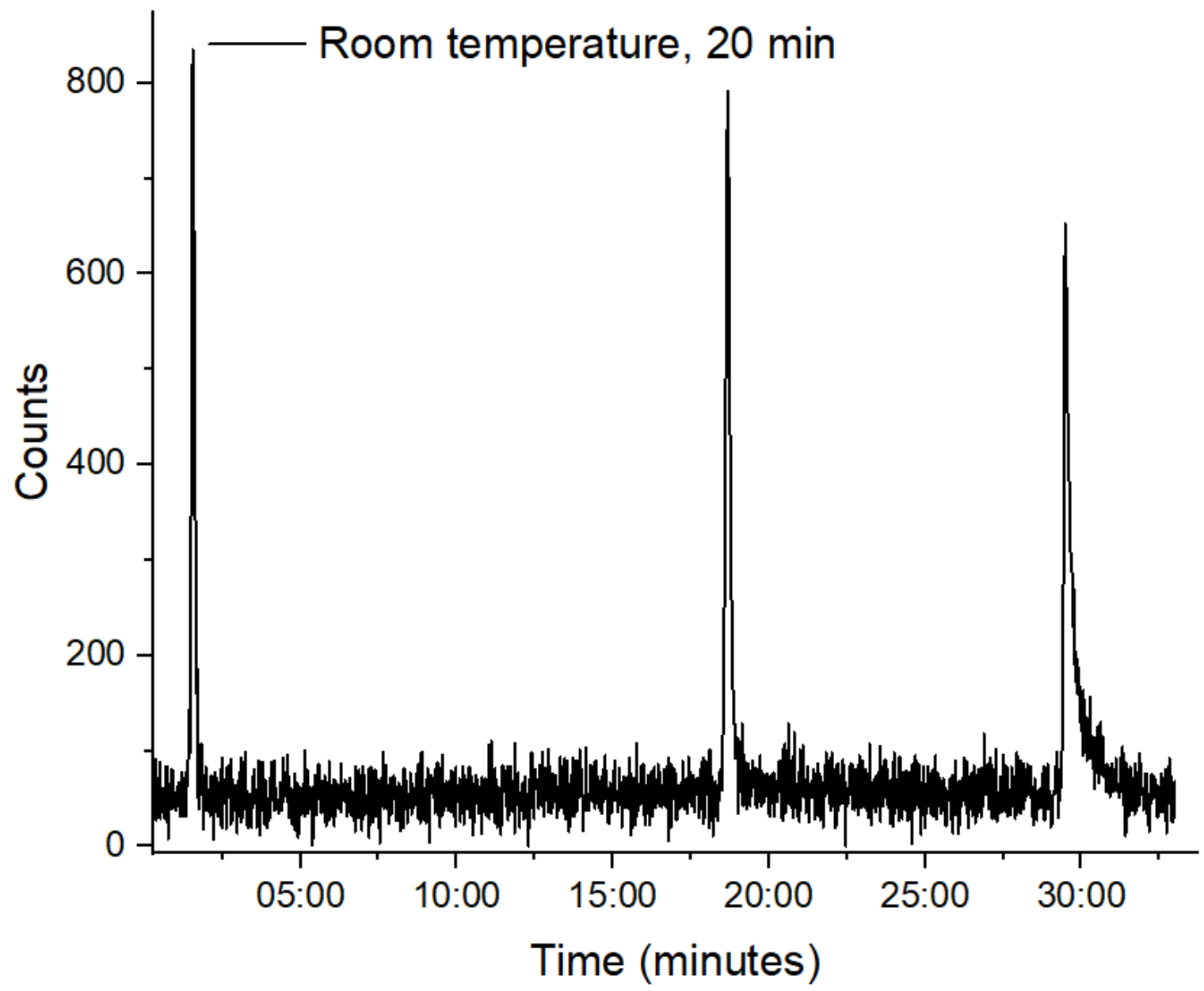


Figure S41. RadioHPLC of **$H_2$KHQ** (1 mM, 20 mM $NH_4OAc$, pH 8.5) with $^{161}Tb[Tb^{3+}]$ (3 MBq, 1mM HCl) after 20 minutes at room temperature. RCY = 33 %. **[$^{161}$Tb][Tb-KHQ]$^+$** retention time = 18.4 min and free [$^{161}$Tb]$Tb^{3+}$ retention time = 1.3 and 29.3 min.

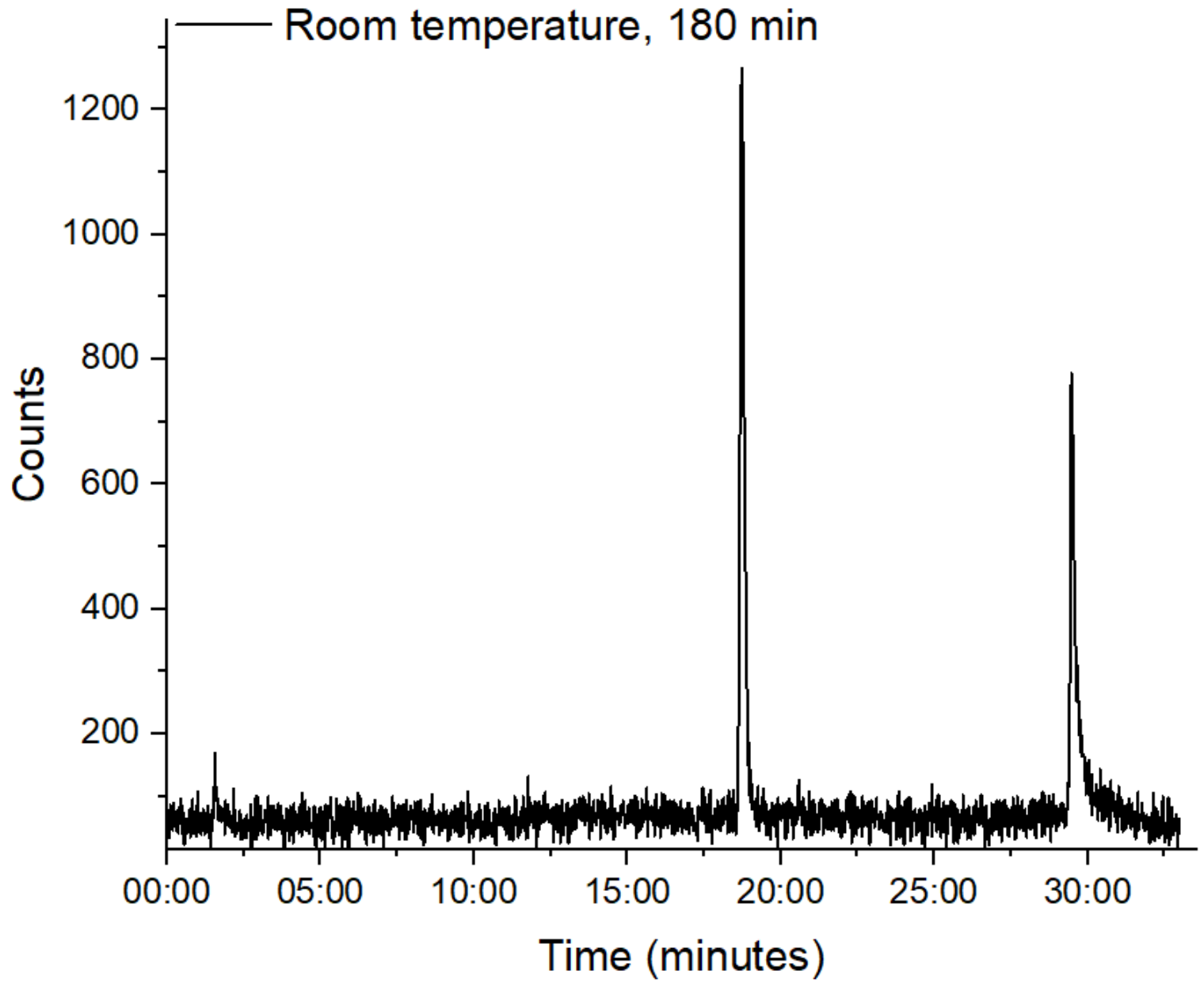


Figure S42. RadioHPLC of **$H_2$KHQ** (1 mM, 20 mM $NH_4OAc$, pH 8.5) with $^{161}Tb[Tb^{3+}]$ (3 MBq, 1mM HCl) after 180 minutes at room temperature. RCY = 66 %. **[$^{161}$Tb][Tb-KHQ]$^+$** retention time = 18.4 min and free [$^{161}$Tb]$Tb^{3+}$ retention time = 1.3 and 29.3 min.

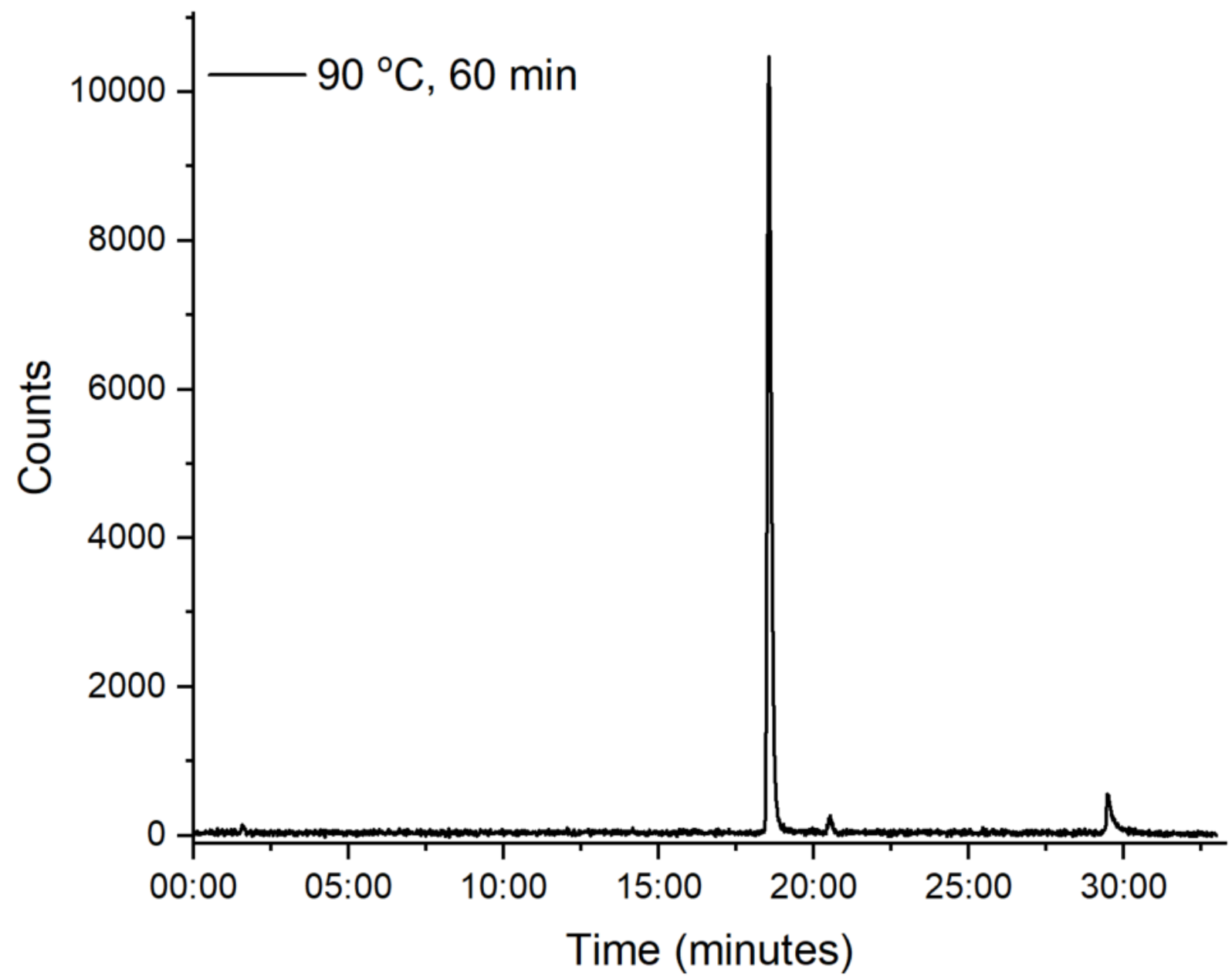


Figure S43. RadioHPLC of **$H_2$KHQ** (1 mM, 20 mM $NH_4OAc$, pH 8.5) with $^{161}Tb[Tb^{3+}]$ (3 MBq, 1mM HCl) after 60 minutes at 90 ˚C. RCY = 92 %. **$[^{161}Tb][Tb\text{-}KHQ]^+$** retention time = 18.3 min and free $[^{161}Tb]Tb^{3+}$ retention time = 29.3 min.

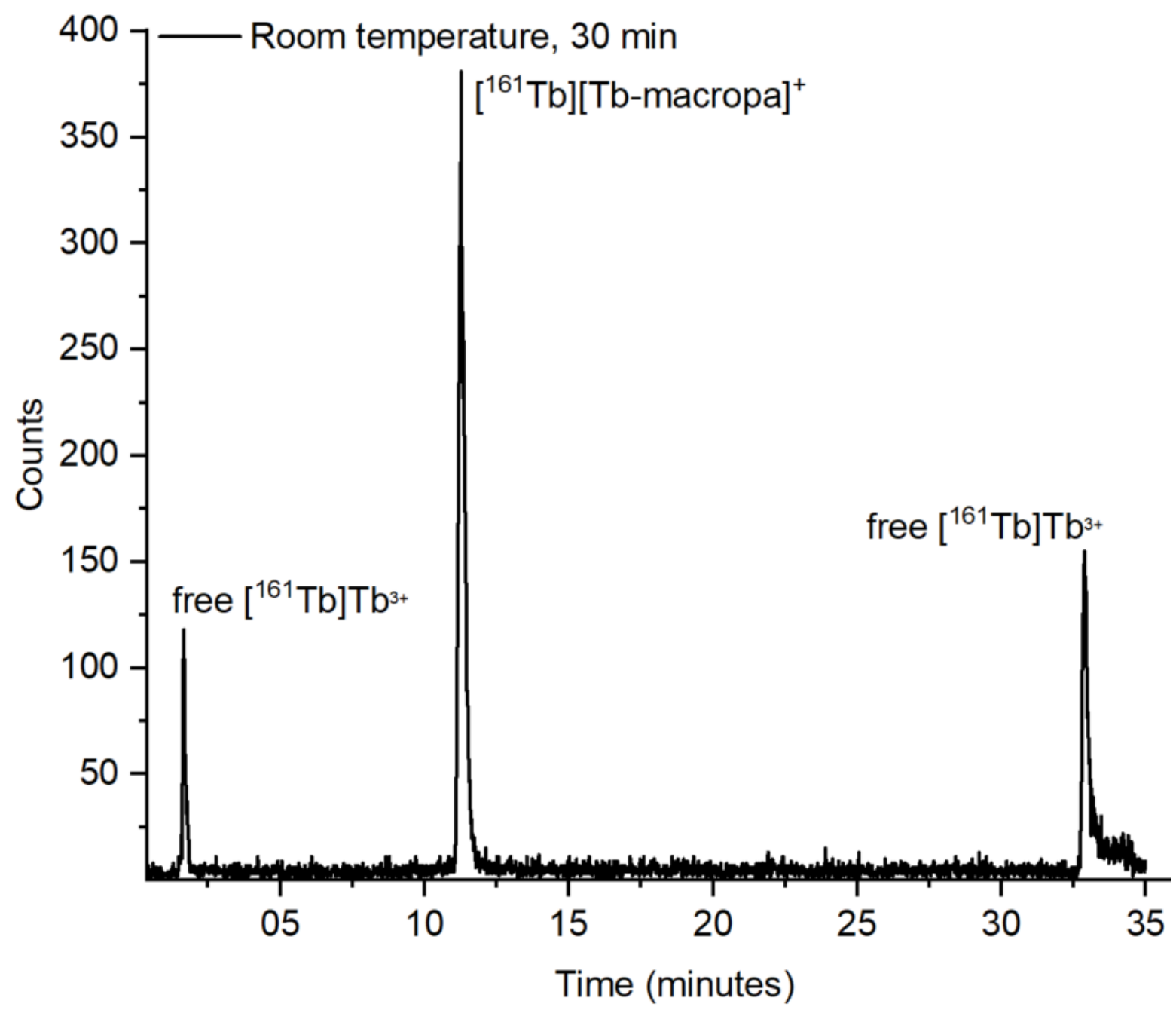


Figure S 44. RadioHPLC of **macropa** (1 mM) with $[^{161}Tb]Tb^{3+}$ (5 MBq, 2 mM HCl) after 30 min at 25 °C. RCY = 62 %. **$[^{161}Tb][Tb\text{-}macropa]^+$** retention time = 11.1 min and free $[^{161}Tb]Tb^{3+}$ retention time = 1.3 and 30.3 min.

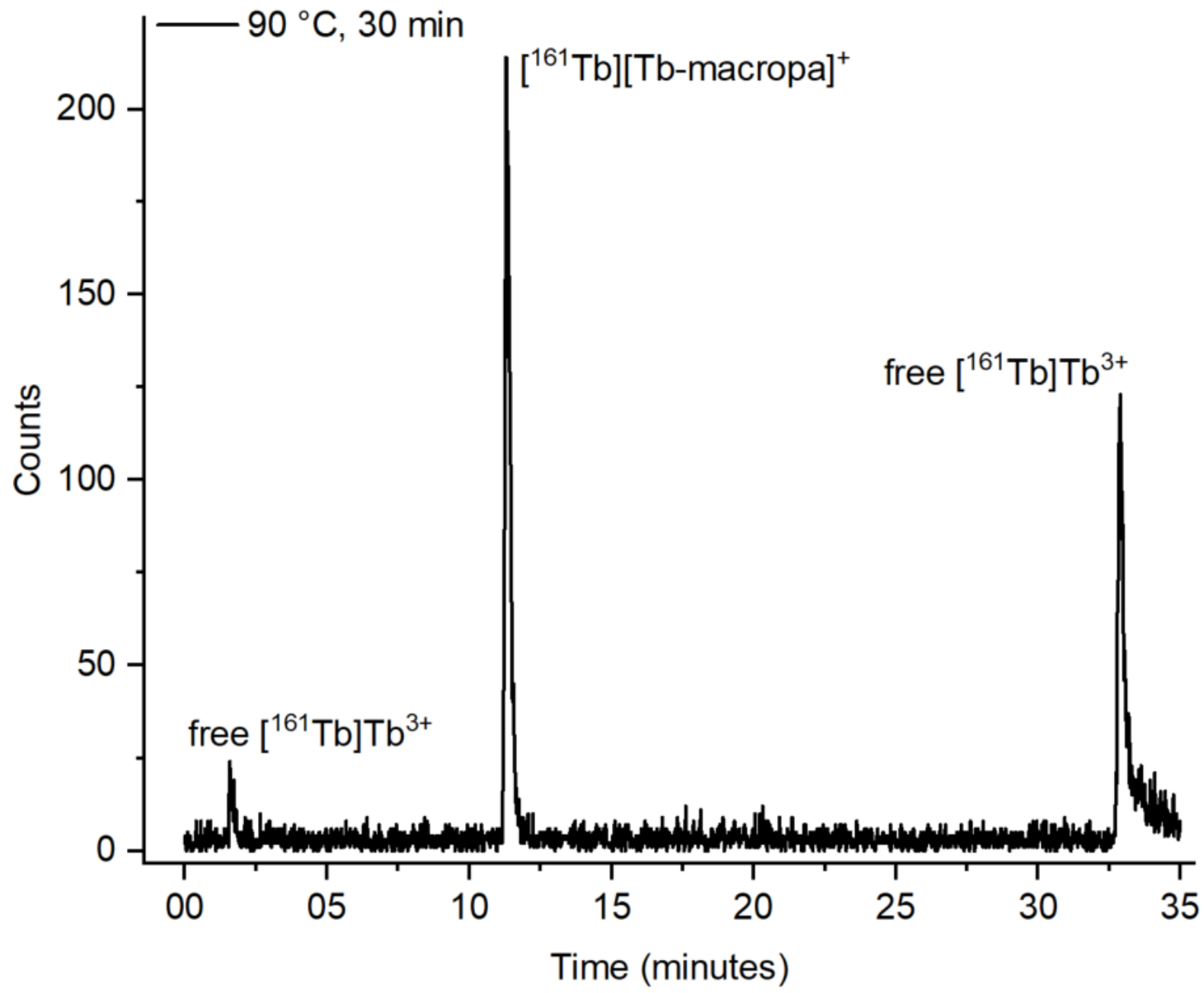


Figure S 45. RadioHPLC of **macropa** (1 mM) with $[^{161}Tb]Tb^{3+}$ (5 MBq, 2 mM HCl) after 30 min at 25 °C. RCY = 55 %. **$[^{161}Tb][Tb\text{-}macropa]^{+}$** retention time = 11.1 min and free $[^{161}Tb]Tb^{3+}$ retention time = 1.3 and 30.3 min.

# 10.References


1 M. El Safadi, M. Bhadbhade, R. Shimmon, A. T. Baker and A. M. McDonagh, Cyclen-based chelators for the inhibition of Aβ aggregation: Synthesis, anti-oxidant and aggregation evaluation, *Inorganica Chim Acta*, 2017, 467, 343–350.

2 L. Southcott, X. Wang, L. Wharton, H. Yang, V. Radchenko, M. Kubeil, H. Stephan, M. de Guadalupe Jaraquemada-Peláez and C. Orvig, High denticity oxinate-linear-backbone chelating ligand for diagnostic radiometal ions [111In]In3+and [89Zr]Zr4+, *Dalton Transactions*, 2021, 50, 3874–3886.

3 N. A. Thiele, V. Brown, J. M. Kelly, A. Amor-Coarasa, U. Jermilova, S. N. MacMillan, A. Nikolopoulou, S. Ponnala, C. F. Ramogida, A. K. H. Robertson, C. Rodríguez-Rodríguez, P. Schaffer, C. Williams, J. W. Babich, V. Radchenko and J. J. Wilson, An Eighteen-Membered Macrocyclic Ligand for Actinium-225 Targeted Alpha Therapy, *Angewandte Chemie*, 2017, 129, 14904–14909.

4 H. E. Gottlieb, V. Kotlyar and A. Nudelman, NMR Chemical Shifts of Common Laboratory Solvents as Trace Impurities, *J Org Chem*, 1997, 62, 7512–7515.

5 G. M. Sheldrick, Crystal structure refinement with SHELXL, *Acta Crystallogr C Struct Chem*, 2015, 71, 3–8.

6 O. V. Dolomanov, L. J. Bourhis, R. J. Gildea, J. A. K. Howard and H. Puschmann, OLEX2: a complete structure solution, refinement and analysis program, *J Appl Crystallogr*, 2009, 42, 339–341.

7 G. M. Sheldrick, SHELXT – Integrated space-group and crystal-structure determination, *Acta Crystallogr A Found Adv*, 2015, 71, 3–8.

8 Gaussian.com, https://gaussian.com/citation/, (accessed 6 March 2025).

9 J.-D. Chai and M. Head-Gordon, Long-range corrected hybrid density functionals with damped atom–atom dispersion corrections, *Physical Chemistry Chemical Physics*, 2008, 10, 6615.

10 J.-D. Chai and M. Head-Gordon, Systematic optimization of long-range corrected hybrid density functionals, *J Chem Phys*, DOI:10.1063/1.2834918.

11 A. Roca-Sabio, M. Regueiro-Figueroa, D. Esteban-Gómez, A. de Blas, T. Rodríguez-Blas and C. Platas-Iglesias, Density functional dependence of molecular geometries in lanthanide(III) complexes relevant to bioanalytical and biomedical applications, *Comput Theor Chem*, 2012, 999, 93–104.

12 F. Weigend and R. Ahlrichs, Balanced basis sets of split valence, triple zeta valence and quadruple zeta valence quality for H to Rn: Design and assessment of accuracy, *Physical Chemistry Chemical Physics*, 2005, 7, 3297.

13 M. Dolg, H. Stoll, A. Savin and H. Preuss, Energy-adjusted pseudopotentials for the rare earth elements, *Theor Chim Acta*, 1989, 75, 173–194.

14 O. Eisenstein and L. Maron, DFT studies of some structures and reactions of lanthanides complexes, *J Organomet Chem*, 2002, 647, 190–197.

15 L. Maron and O. Eisenstein, Do f Electrons Play a Role in the Lanthanide−Ligand Bonds? A DFT Study of Ln(NR 2 ) 3 ; R = H, SiH 3, *J Phys Chem A*, 2000, 104, 7140–7143.

16 A. V. Marenich, C. J. Cramer and D. G. Truhlar, Universal Solvation Model Based on Solute Electron Density and on a Continuum Model of the Solvent Defined by the Bulk Dielectric Constant and Atomic Surface Tensions, *J Phys Chem B*, 2009, 113, 6378–6396.

17 M. Regueiro-Figueroa, D. Esteban-Gómez, A. de Blas, T. Rodríguez-Blas and C. Platas-Iglesias, Understanding Stability Trends along the Lanthanide Series, *Chemistry – A European Journal*, 2014, 20, 3974–3981.

18 G. Luchini, J. V. Alegre-Requena, I. Funes-Ardoiz and R. S. Paton, GoodVibes: automated thermochemistry for heterogeneous computational chemistry data, *F1000Res*, 2020, 9, 291.

19 A. Roca-Sabio, M. Mato-Iglesias, D. Esteban-Gómez, É. Toth, A. De Bias, C. Platas-Iglesias and T. Rodríguez-Blas, Macrocyclic receptor exhibiting unprecedented selectivity for light lanthanides, *J Am Chem Soc*, 2009, 131, 3331–3341.

20 F. H. Sweeton, R. E. Mesmer and C. F. ’ Baes, *Acidity Measurements at Elevated Temperatures. VII. Dissociation of Water*, 1974, vol. 3.

21 P. Gans and B. O’sullivan, *GLEE, a new computer program for glass electrode calibration*, 2000, vol. 51.

22 Wuhan University, *Analytical Chemistry I*, Higher Education Press, Beijing, 5th edn., 2006.

23 P. Garw, A. Sabatinib and A. Vaccab, *Investigation of equilibria in solution. Determination of equilibrium constants with the HYPERQUAD suite of programs*, 1996, vol. 43.

# 11.XYZ Coordinates

Ce-anti-KHQ-Ligand(2–)

SCF (RwB97XD) = -1910.50741294
E(SCF)+ZPE(0 K)= -1909.841038
H(298 K)= -1909.807021
G(298 K)= -1909.902171
Lowest Frequency = -50.0982cm-1

| | | | |
|---|---|---|---|
| C | 5.839215 | 1.391568 | 17.400938 |
| C | 5.673871 | 1.621101 | 18.792816 |
| C | 5.296247 | 0.499050 | 19.575289 |
| H | 5.169540 | 0.617177 | 20.654405 |
| C | 5.057829 | -0.713839 | 18.974486 |
| H | 4.723824 | -1.574013 | 19.558321 |
| C | 5.236245 | -0.837094 | 17.575066 |
| C | 4.815193 | -2.094441 | 16.852618 |
| H | 4.955824 | -2.976730 | 17.502110 |
| H | 3.722164 | -2.017820 | 16.724715 |
| C | 4.466803 | -2.479190 | 14.476246 |
| H | 3.711114 | -3.244030 | 14.744697 |
| H | 5.013967 | -2.821186 | 13.588921 |
| C | 3.748063 | -1.194539 | 14.106094 |
| H | 2.983899 | -1.428285 | 13.346112 |
| H | 3.236764 | -0.733110 | 14.970192 |
| C | 4.208077 | 0.712336 | 12.677334 |
| H | 3.943999 | 1.623437 | 13.240164 |
| H | 3.316866 | 0.347929 | 12.140248 |
| C | 5.304495 | 1.005426 | 11.676165 |
| H | 5.522228 | 0.115570 | 11.060554 |
| H | 4.981635 | 1.827879 | 11.016529 |
| C | 7.369666 | 2.273664 | 11.777042 |
| H | 6.807751 | 3.015908 | 11.185542 |
| H | 8.028670 | 1.721330 | 11.086877 |
| C | 6.457424 | -3.342896 | 15.576012 |
| H | 6.871113 | -3.426863 | 14.562872 |
| H | 6.026528 | -4.324085 | 15.863246 |
| N | 5.660964 | 0.166408 | 16.839616 |
| N | 5.445029 | -2.287279 | 15.549093 |
| O | 6.296926 | 2.195571 | 15.226536 |
| O | 4.685115 | -0.279219 | 13.565759 |
| O | 6.459311 | 1.385107 | 12.399845 |
| C | 7.763561 | -2.226629 | 12.043133 |
| C | 8.874068 | -1.371465 | 11.713481 |
| C | 9.691958 | -1.634301 | 10.581509 |
| C | 10.722892 | -0.698898 | 10.314903 |
| H | 11.377476 | -0.851962 | 9.453360 |
| C | 10.877822 | 0.400268 | 11.123475 |
| H | 11.649735 | 1.144633 | 10.919687 |
| C | 10.017105 | 0.566336 | 12.234239 |
| C | 10.179320 | 1.747691 | 13.154698 |
| H | 10.682152 | 2.577107 | 12.619633 |
| H | 10.872823 | 1.440558 | 13.951969 |
| C | 9.180836 | 2.925607 | 15.006443 |
| H | 9.873651 | 3.777721 | 14.853550 |
| H | 8.222535 | 3.330268 | 15.343041 |
| C | 9.759867 | 2.054325 | 16.102217 |

| | | | |
|---|---|---|---|
| H | 9.683578 | 2.593057 | 17.062131 |
| H | 10.828456 | 1.834381 | 15.929888 |
| C | 9.741260 | -0.126531 | 16.989721 |
| H | 10.582948 | -0.550716 | 16.412475 |
| H | 10.147460 | 0.355079 | 17.895182 |
| C | 8.789363 | -1.208020 | 17.417418 |
| H | 8.021343 | -0.804054 | 18.102772 |
| H | 9.363987 | -1.982142 | 17.953302 |
| C | 7.566903 | -3.031767 | 16.556148 |
| H | 8.340816 | -3.815381 | 16.500142 |
| H | 7.183381 | -3.015959 | 17.591263 |
| C | 8.125055 | 3.026866 | 12.857172 |
| H | 7.370600 | 3.530737 | 13.475626 |
| H | 8.752417 | 3.799931 | 12.370117 |
| N | 9.069065 | -0.297722 | 12.526872 |
| N | 8.929805 | 2.195621 | 13.762383 |
| O | 9.057018 | 0.821785 | 16.194253 |
| O | 8.157444 | -1.764072 | 16.278824 |
| O | 7.029941 | -1.916042 | 13.069044 |
| C | 7.547444 | -3.333366 | 11.224052 |
| H | 6.710369 | -3.995894 | 11.453980 |
| C | 8.378390 | -3.597962 | 10.116077 |
| H | 8.170338 | -4.478472 | 9.503570 |
| C | 9.432540 | -2.775448 | 9.782385 |
| H | 10.061863 | -2.983450 | 8.915535 |
| C | 6.155502 | 2.465732 | 16.487384 |
| C | 6.299225 | 3.736915 | 17.049423 |
| H | 6.495755 | 4.581425 | 16.384990 |
| C | 6.164041 | 3.949484 | 18.437087 |
| H | 6.289325 | 4.963607 | 18.824349 |
| C | 5.864732 | 2.926004 | 19.310907 |
| H | 5.748147 | 3.108739 | 20.380374 |

Ce-anti-KHQ(1+)

SCF (RwB97XD) = -1943.05035903
E(SCF)+ZPE(0 K)= -1942.368398
H(298 K)= -1942.331048
G(298 K)= -1942.434339
Lowest Frequency = 15.5893cm-1

| | | | |
|---|---|---|---|
| C | 5.839215 | 1.391568 | 17.400938 |
| C | 5.673871 | 1.621101 | 18.792816 |
| C | 5.296247 | 0.499050 | 19.575289 |
| H | 5.169540 | 0.617177 | 20.654405 |
| C | 5.057829 | -0.713839 | 18.974486 |
| H | 4.723824 | -1.574013 | 19.558321 |
| C | 5.236245 | -0.837094 | 17.575066 |
| C | 4.815193 | -2.094441 | 16.852618 |
| H | 4.955824 | -2.976730 | 17.502110 |
| H | 3.722164 | -2.017820 | 16.724715 |
| C | 4.466803 | -2.479190 | 14.476246 |
| H | 3.711114 | -3.244030 | 14.744697 |
| H | 5.013967 | -2.821186 | 13.588921 |
| C | 3.748063 | -1.194539 | 14.106094 |
| H | 2.983899 | -1.428285 | 13.346112 |
| H | 3.236764 | -0.733110 | 14.970192 |

| | | | |
|---|---|---|---|
| C | 4.208077 | 0.712336 | 12.677334 |
| H | 3.943999 | 1.623437 | 13.240164 |
| H | 3.316866 | 0.347929 | 12.140248 |
| C | 5.304495 | 1.005426 | 11.676165 |
| H | 5.522228 | 0.115570 | 11.060554 |
| H | 4.981635 | 1.827879 | 11.016529 |
| C | 7.369666 | 2.273664 | 11.777042 |
| H | 6.807751 | 3.015908 | 11.185542 |
| H | 8.028670 | 1.721330 | 11.086877 |
| C | 6.457424 | -3.342896 | 15.576012 |
| H | 6.871113 | -3.426863 | 14.562872 |
| H | 6.026528 | -4.324085 | 15.863246 |
| N | 5.660964 | 0.166408 | 16.839616 |
| N | 5.445029 | -2.287279 | 15.549093 |
| O | 6.296926 | 2.195571 | 15.226536 |
| O | 4.685115 | -0.279219 | 13.565759 |
| O | 6.459311 | 1.385107 | 12.399845 |
| C | 7.763561 | -2.226629 | 12.043133 |
| C | 8.874068 | -1.371465 | 11.713481 |
| C | 9.691958 | -1.634301 | 10.581509 |
| C | 10.722892 | -0.698898 | 10.314903 |
| H | 11.377476 | -0.851962 | 9.453360 |
| C | 10.877822 | 0.400268 | 11.123475 |
| H | 11.649735 | 1.144633 | 10.919687 |
| C | 10.017105 | 0.566336 | 12.234239 |
| C | 10.179320 | 1.747691 | 13.154698 |
| H | 10.682152 | 2.577107 | 12.619633 |
| H | 10.872823 | 1.440558 | 13.951969 |
| C | 9.180836 | 2.925607 | 15.006443 |
| H | 9.873651 | 3.777721 | 14.853550 |
| H | 8.222535 | 3.330268 | 15.343041 |
| C | 9.759867 | 2.054325 | 16.102217 |
| H | 9.683578 | 2.593057 | 17.062131 |
| H | 10.828456 | 1.834381 | 15.929888 |
| C | 9.741260 | -0.126531 | 16.989721 |
| H | 10.582948 | -0.550716 | 16.412475 |
| H | 10.147460 | 0.355079 | 17.895182 |
| C | 8.789363 | -1.208020 | 17.417418 |
| H | 8.021343 | -0.804054 | 18.102772 |
| H | 9.363987 | -1.982142 | 17.953302 |
| C | 7.566903 | -3.031767 | 16.556148 |
| H | 8.340816 | -3.815381 | 16.500142 |
| H | 7.183381 | -3.015959 | 17.591263 |
| C | 8.125055 | 3.026866 | 12.857172 |
| H | 7.370600 | 3.530737 | 13.475626 |
| H | 8.752417 | 3.799931 | 12.370117 |
| N | 9.069065 | -0.297722 | 12.526872 |
| N | 8.929805 | 2.195621 | 13.762383 |
| O | 9.057018 | 0.821785 | 16.194253 |
| O | 8.157444 | -1.764072 | 16.278824 |
| O | 7.029941 | -1.916042 | 13.069044 |
| C | 7.547444 | -3.333366 | 11.224052 |
| H | 6.710369 | -3.995894 | 11.453980 |
| C | 8.378390 | -3.597962 | 10.116077 |
| H | 8.170338 | -4.478472 | 9.503570 |
| C | 9.432540 | -2.775448 | 9.782385 |
| H | 10.061863 | -2.983450 | 8.915535 |

| | | | |
|---|---|---|---|
| C | 6.155502 | 2.465732 | 16.487384 |
| C | 6.299225 | 3.736915 | 17.049423 |
| H | 6.495755 | 4.581425 | 16.384990 |
| C | 6.164041 | 3.949484 | 18.437087 |
| H | 6.289325 | 4.963607 | 18.824349 |
| C | 5.864732 | 2.926004 | 19.310907 |
| H | 5.748147 | 3.108739 | 20.380374 |
| Ce | 7.101623 | 0.004712 | 14.512866 |

Ce3+_Ion

SCF (RwB97XD) = -30.8172967268
E(SCF)+ZPE(0 K)= -30.817297
H(298 K)= -30.814936
G(298 K)= -30.834283

| | | | |
|---|---|---|---|
| Ce | 0.887400 | 3.945900 | 9.220400 |

Ce-syn-KHQ_Ligand(2–)

SCF (RwB97XD) = -1910.51323826
E(SCF)+ZPE(0 K)= -1909.846041
H(298 K)= -1909.810738
G(298 K)= -1909.909183
Lowest Frequency = -37.7082cm-1

| | | | |
|---|---|---|---|
| N | 3.120387 | 2.590545 | 9.213233 |
| C | 3.132225 | 1.284401 | 9.354883 |
| C | 4.249211 | 0.621776 | 9.912644 |
| H | 4.233545 | -0.461754 | 10.040348 |
| C | 5.341511 | 1.364971 | 10.296833 |
| H | 6.209164 | 0.874121 | 10.744240 |
| C | 5.369729 | 2.770223 | 10.092519 |
| C | 4.204490 | 3.340674 | 9.527197 |
| C | 4.093030 | 4.752139 | 9.245957 |
| O | 2.962678 | 5.203360 | 8.775381 |
| C | 5.210775 | 5.530306 | 9.538387 |
| H | 5.181501 | 6.599185 | 9.314229 |
| C | 6.369440 | 4.959873 | 10.111882 |
| H | 7.216606 | 5.614163 | 10.331460 |
| C | 6.466122 | 3.615627 | 10.399862 |
| H | 7.370829 | 3.194908 | 10.841731 |
| N | 1.910260 | 4.436571 | 11.584699 |
| C | 2.522639 | 5.554144 | 11.903434 |
| C | 3.500551 | 5.589948 | 12.924397 |
| H | 4.007470 | 6.527192 | 13.159534 |
| C | 3.807878 | 4.430185 | 13.595932 |
| H | 4.573559 | 4.432272 | 14.375444 |
| C | 3.119416 | 3.223282 | 13.302581 |
| C | 2.152441 | 3.292076 | 12.271891 |
| C | 1.365876 | 2.149991 | 11.879347 |
| O | 0.527926 | 2.290495 | 10.888757 |
| C | 1.592171 | 0.967376 | 12.578533 |
| H | 0.999659 | 0.084751 | 12.326687 |
| C | 2.565905 | 0.898648 | 13.599900 |
| H | 2.713345 | -0.053329 | 14.115610 |
| C | 3.328336 | 1.986983 | 13.965305 |

| | | | |
|---|---|---|---|
| H | 4.074213 | 1.914712 | 14.758557 |
| C | 1.893616 | 0.546618 | 8.899406 |
| H | 2.181869 | -0.430985 | 8.463916 |
| H | 1.272897 | 0.348186 | 9.784810 |
| N | 1.098272 | 1.331518 | 7.946146 |
| C | 1.811341 | 1.464369 | 6.672099 |
| H | 1.810156 | 0.510480 | 6.107755 |
| H | 2.857342 | 1.714306 | 6.890556 |
| C | 1.252214 | 2.551077 | 5.779561 |
| H | 1.914009 | 2.653640 | 4.902035 |
| H | 0.242004 | 2.313518 | 5.398900 |
| O | 1.210143 | 3.769583 | 6.493442 |
| C | 0.982172 | 4.897687 | 5.674259 |
| H | -0.022716 | 4.830960 | 5.218425 |
| H | 1.731676 | 4.937325 | 4.864596 |
| C | 1.103386 | 6.141784 | 6.513517 |
| H | 2.121988 | 6.227067 | 6.927931 |
| H | 0.873748 | 7.019450 | 5.886758 |
| O | 0.160786 | 6.042847 | 7.575569 |
| C | -0.183514 | 7.264457 | 8.204961 |
| H | -1.217481 | 7.148877 | 8.546108 |
| H | -0.183281 | 8.078862 | 7.461101 |
| C | 0.768186 | 7.639035 | 9.333963 |
| H | 1.770473 | 7.754130 | 8.898038 |
| H | 0.469798 | 8.632732 | 9.726438 |
| N | 0.874886 | 6.645692 | 10.404030 |
| C | 2.159622 | 6.782688 | 11.104439 |
| H | 2.164338 | 7.671247 | 11.766290 |
| H | 2.942730 | 6.926471 | 10.348111 |
| C | -0.214193 | 6.687671 | 11.382973 |
| H | -0.347211 | 7.709097 | 11.793498 |
| H | 0.077788 | 6.038121 | 12.218296 |
| C | -1.562125 | 6.206391 | 10.880251 |
| H | -2.213171 | 6.031343 | 11.754369 |
| H | -2.068827 | 6.957615 | 10.250367 |
| O | -1.398565 | 5.000975 | 10.158780 |
| C | -2.605431 | 4.407515 | 9.711442 |
| H | -2.857249 | 4.787409 | 8.704910 |
| H | -3.431329 | 4.665527 | 10.394462 |
| C | -2.447878 | 2.910544 | 9.707538 |
| H | -2.174178 | 2.564564 | 10.719871 |
| H | -3.407961 | 2.447847 | 9.416763 |
| O | -1.434304 | 2.532830 | 8.796067 |
| C | -1.231588 | 1.123101 | 8.810813 |
| H | -2.188387 | 0.617444 | 8.591812 |
| H | -0.889667 | 0.831741 | 9.816348 |
| C | -0.230314 | 0.747377 | 7.745454 |
| H | -0.176368 | -0.357079 | 7.684232 |
| H | -0.625364 | 1.094108 | 6.780947 |

Ce-syn-KHQ(1+)

SCF (RwB97XD) = -1943.06342609
E(SCF)+ZPE(0 K)= -1942.380887
H(298 K)= -1942.343934
G(298 K)= -1942.444617
Lowest Frequency = 35.6606cm-1

| | | | |
|---|---|---|---|
| Ce | 0.907874 | 3.974727 | 9.194455 |
| N | 3.120387 | 2.590545 | 9.213233 |
| C | 3.132225 | 1.284401 | 9.354883 |
| C | 4.249211 | 0.621776 | 9.912644 |
| H | 4.233545 | -0.461754 | 10.040348 |
| C | 5.341511 | 1.364971 | 10.296833 |
| H | 6.209164 | 0.874121 | 10.744240 |
| C | 5.369729 | 2.770223 | 10.092519 |
| C | 4.204490 | 3.340674 | 9.527197 |
| C | 4.093030 | 4.752139 | 9.245957 |
| O | 2.962678 | 5.203360 | 8.775381 |
| C | 5.210775 | 5.530306 | 9.538387 |
| H | 5.181501 | 6.599185 | 9.314229 |
| C | 6.369440 | 4.959873 | 10.111882 |
| H | 7.216606 | 5.614163 | 10.331460 |
| C | 6.466122 | 3.615627 | 10.399862 |
| H | 7.370829 | 3.194908 | 10.841731 |
| N | 1.910260 | 4.436571 | 11.584699 |
| C | 2.522639 | 5.554144 | 11.903434 |
| C | 3.500551 | 5.589948 | 12.924397 |
| H | 4.007470 | 6.527192 | 13.159534 |
| C | 3.807878 | 4.430185 | 13.595932 |
| H | 4.573559 | 4.432272 | 14.375444 |
| C | 3.119416 | 3.223282 | 13.302581 |
| C | 2.152441 | 3.292076 | 12.271891 |
| C | 1.365876 | 2.149991 | 11.879347 |
| O | 0.527926 | 2.290495 | 10.888757 |
| C | 1.592171 | 0.967376 | 12.578533 |
| H | 0.999659 | 0.084751 | 12.326687 |
| C | 2.565905 | 0.898648 | 13.599900 |
| H | 2.713345 | -0.053329 | 14.115610 |
| C | 3.328336 | 1.986983 | 13.965305 |
| H | 4.074213 | 1.914712 | 14.758557 |
| C | 1.893616 | 0.546618 | 8.899406 |
| H | 2.181869 | -0.430985 | 8.463916 |
| H | 1.272897 | 0.348186 | 9.784810 |
| N | 1.098272 | 1.331518 | 7.946146 |
| C | 1.811341 | 1.464369 | 6.672099 |
| H | 1.810156 | 0.510480 | 6.107755 |
| H | 2.857342 | 1.714306 | 6.890556 |
| C | 1.252214 | 2.551077 | 5.779561 |
| H | 1.914009 | 2.653640 | 4.902035 |
| H | 0.242004 | 2.313518 | 5.398900 |
| O | 1.210143 | 3.769583 | 6.493442 |
| C | 0.982172 | 4.897687 | 5.674259 |
| H | -0.022716 | 4.830960 | 5.218425 |
| H | 1.731676 | 4.937325 | 4.864596 |
| C | 1.103386 | 6.141784 | 6.513517 |
| H | 2.121988 | 6.227067 | 6.927931 |
| H | 0.873748 | 7.019450 | 5.886758 |
| O | 0.160786 | 6.042847 | 7.575569 |
| C | -0.183514 | 7.264457 | 8.204961 |
| H | -1.217481 | 7.148877 | 8.546108 |
| H | -0.183281 | 8.078862 | 7.461101 |
| C | 0.768186 | 7.639035 | 9.333963 |
| H | 1.770473 | 7.754130 | 8.898038 |

| | | | |
|---|---|---|---|
| H | 0.469798 | 8.632732 | 9.726438 |
| N | 0.874886 | 6.645692 | 10.404030 |
| C | 2.159622 | 6.782688 | 11.104439 |
| H | 2.164338 | 7.671247 | 11.766290 |
| H | 2.942730 | 6.926471 | 10.348111 |
| C | -0.214193 | 6.687671 | 11.382973 |
| H | -0.347211 | 7.709097 | 11.793498 |
| H | 0.077788 | 6.038121 | 12.218296 |
| C | -1.562125 | 6.206391 | 10.880251 |
| H | -2.213171 | 6.031343 | 11.754369 |
| H | -2.068827 | 6.957615 | 10.250367 |
| O | -1.398565 | 5.000975 | 10.158780 |
| C | -2.605431 | 4.407515 | 9.711442 |
| H | -2.857249 | 4.787409 | 8.704910 |
| H | -3.431329 | 4.665527 | 10.394462 |
| C | -2.447878 | 2.910544 | 9.707538 |
| H | -2.174178 | 2.564564 | 10.719871 |
| H | -3.407961 | 2.447847 | 9.416763 |
| O | -1.434304 | 2.532830 | 8.796067 |
| C | -1.231588 | 1.123101 | 8.810813 |
| H | -2.188387 | 0.617444 | 8.591812 |
| H | -0.889667 | 0.831741 | 9.816348 |
| C | -0.230314 | 0.747377 | 7.745454 |
| H | -0.176368 | -0.357079 | 7.684232 |
| H | -0.625364 | 1.094108 | 6.780947 |

Ce-syn-H2O-KHQ(1+)

SCF (RwB97XD) = -2019.42687097
E(SCF)+ZPE(0 K)= -2018.718659
H(298 K)= -2018.679328
G(298 K)= -2018.783906
Lowest Frequency = 32.2166cm-1

| | | | |
|---|---|---|---|
| Ce | 0.903315 | 4.000833 | 9.071929 |
| N | 3.103868 | 2.580916 | 9.117116 |
| C | 3.108503 | 1.269641 | 9.197786 |
| C | 4.228522 | 0.569794 | 9.702379 |
| H | 4.206376 | -0.519296 | 9.767383 |
| C | 5.329313 | 1.281788 | 10.116571 |
| H | 6.199747 | 0.761945 | 10.524065 |
| C | 5.357427 | 2.696581 | 10.003921 |
| C | 4.192734 | 3.305322 | 9.476086 |
| C | 4.090510 | 4.733184 | 9.292798 |
| O | 2.972699 | 5.224304 | 8.834346 |
| C | 5.204980 | 5.487346 | 9.652576 |
| H | 5.176258 | 6.569896 | 9.507810 |
| C | 6.361054 | 4.876926 | 10.187443 |
| H | 7.206972 | 5.512645 | 10.459888 |
| C | 6.455234 | 3.514958 | 10.372391 |
| H | 7.356958 | 3.060547 | 10.786105 |
| N | 1.814701 | 4.481215 | 11.546299 |
| C | 2.348435 | 5.619451 | 11.924854 |
| C | 3.220400 | 5.699339 | 13.036830 |
| H | 3.650546 | 6.661346 | 13.320606 |
| C | 3.525754 | 4.554666 | 13.731427 |
| H | 4.210930 | 4.589725 | 14.581849 |

| | | | |
|---|---|---|---|
| C | 2.941078 | 3.316097 | 13.359702 |
| C | 2.066094 | 3.345346 | 12.246770 |
| C | 1.390184 | 2.161478 | 11.782768 |
| O | 0.614471 | 2.265195 | 10.742211 |
| C | 1.637188 | 0.981106 | 12.479742 |
| H | 1.129488 | 0.066462 | 12.164049 |
| C | 2.521214 | 0.954647 | 13.580586 |
| H | 2.688683 | 0.004555 | 14.093609 |
| C | 3.170924 | 2.085127 | 14.025306 |
| H | 3.848672 | 2.049215 | 14.879828 |
| C | 1.855015 | 0.550361 | 8.759138 |
| H | 2.124984 | -0.410207 | 8.276522 |
| H | 1.278914 | 0.318877 | 9.664125 |
| N | 1.019246 | 1.362946 | 7.864319 |
| C | 1.691202 | 1.480662 | 6.559941 |
| H | 1.549857 | 0.558302 | 5.963002 |
| H | 2.768814 | 1.587142 | 6.740426 |
| C | 1.254225 | 2.664794 | 5.728589 |
| H | 1.798430 | 2.629976 | 4.768231 |
| H | 0.172533 | 2.643569 | 5.503604 |
| O | 1.564115 | 3.861891 | 6.410333 |
| C | 1.407163 | 5.007935 | 5.602045 |
| H | 0.433029 | 4.963181 | 5.080456 |
| H | 2.204481 | 5.043316 | 4.838316 |
| C | 1.477443 | 6.256481 | 6.444399 |
| H | 2.434241 | 6.297539 | 6.989828 |
| H | 1.377759 | 7.133348 | 5.781786 |
| O | 0.395340 | 6.225333 | 7.369952 |
| C | 0.062562 | 7.452606 | 7.983686 |
| H | -1.008159 | 7.400859 | 8.223238 |
| H | 0.186537 | 8.282369 | 7.266303 |
| C | 0.907026 | 7.739637 | 9.211306 |
| H | 1.952835 | 7.797116 | 8.880583 |
| H | 0.634496 | 8.740866 | 9.603128 |
| N | 0.846372 | 6.719977 | 10.263344 |
| C | 2.060082 | 6.834707 | 11.084815 |
| H | 2.019431 | 7.735301 | 11.729120 |
| H | 2.912681 | 6.953210 | 10.403299 |
| C | -0.335348 | 6.811027 | 11.127361 |
| H | -0.571449 | 7.867074 | 11.369001 |
| H | -0.088501 | 6.312580 | 12.074960 |
| C | -1.606786 | 6.156011 | 10.618937 |
| H | -2.366934 | 6.262465 | 11.412435 |
| H | -2.018127 | 6.645637 | 9.719272 |
| O | -1.387501 | 4.782527 | 10.356810 |
| C | -2.574759 | 4.010118 | 10.366730 |
| H | -3.228027 | 4.315228 | 9.525925 |
| H | -3.129931 | 4.198141 | 11.302034 |
| C | -2.261799 | 2.535995 | 10.297048 |
| H | -1.595589 | 2.250042 | 11.126032 |
| H | -3.211074 | 1.974581 | 10.367582 |
| O | -1.613361 | 2.255379 | 9.073028 |
| C | -1.235100 | 0.899574 | 8.905269 |
| H | -2.135841 | 0.285345 | 8.726276 |
| H | -0.748089 | 0.555305 | 9.829107 |
| C | -0.313701 | 0.767126 | 7.706492 |
| H | -0.228441 | -0.311094 | 7.462744 |

| | | | |
|---|---|---|---|
| H | -0.803440 | 1.242984 | 6.843855 |
| O | -1.139874 | 4.002695 | 7.273416 |
| H | -1.804228 | 3.427682 | 7.686220 |
| H | -1.540678 | 4.822377 | 6.971335 |

Dy-anti-KHQ-Ligand(2–)

SCF (RwB97XD) = -1910.48319972
E(SCF)+ZPE(0 K)= -1909.817631
H(298 K)= -1909.783201
G(298 K)= -1909.881087
Lowest Frequency = -76.3671cm-1

| | | | |
|---|---|---|---|
| C | 5.784349 | 1.423899 | 17.325255 |
| C | 5.435402 | 1.678492 | 18.676013 |
| C | 5.110831 | 0.545787 | 19.467534 |
| H | 4.831747 | 0.684551 | 20.514941 |
| C | 5.109804 | -0.705562 | 18.904941 |
| H | 4.817865 | -1.583415 | 19.484850 |
| C | 5.489321 | -0.855929 | 17.547746 |
| C | 5.480964 | -2.224163 | 16.914123 |
| H | 6.319359 | -2.794835 | 17.343355 |
| H | 4.571286 | -2.764975 | 17.241679 |
| C | 4.302554 | -2.148552 | 14.771277 |
| H | 3.555761 | -2.810504 | 15.252457 |
| H | 4.465399 | -2.504588 | 13.745930 |
| C | 3.712776 | -0.751610 | 14.656923 |
| H | 2.753616 | -0.826620 | 14.118698 |
| H | 3.519140 | -0.284452 | 15.636732 |
| C | 4.116126 | 1.063893 | 13.063065 |
| H | 4.017443 | 2.002600 | 13.632357 |
| H | 3.134727 | 0.772637 | 12.654283 |
| C | 5.101990 | 1.232686 | 11.922104 |
| H | 5.149101 | 0.316380 | 11.311194 |
| H | 4.786456 | 2.081222 | 11.293131 |
| C | 7.319141 | 2.261500 | 11.770963 |
| H | 6.793511 | 2.960887 | 11.100278 |
| H | 7.948128 | 1.601789 | 11.150801 |
| C | 6.401094 | -3.334691 | 14.984130 |
| H | 6.332125 | -3.344590 | 13.894645 |
| H | 6.028316 | -4.310502 | 15.355325 |
| N | 5.855368 | 0.165644 | 16.808175 |
| N | 5.594826 | -2.214799 | 15.461762 |
| O | 6.292706 | 2.198554 | 15.162688 |
| O | 4.612797 | 0.045209 | 13.910521 |
| O | 6.372595 | 1.492330 | 12.488266 |
| C | 7.572850 | -2.059962 | 12.118363 |
| C | 8.864059 | -1.429110 | 12.038843 |
| C | 9.907364 | -1.975070 | 11.248510 |
| C | 11.097332 | -1.206754 | 11.157779 |
| H | 11.932531 | -1.578456 | 10.559048 |
| C | 11.176257 | 0.010698 | 11.785695 |
| H | 12.065242 | 0.637261 | 11.689486 |
| C | 10.082275 | 0.460644 | 12.566084 |
| C | 10.151378 | 1.800842 | 13.253444 |
| H | 10.617924 | 2.533403 | 12.565674 |
| H | 10.859310 | 1.708818 | 14.092011 |

| | | | |
|---|---|---|---|
| C | 9.031053 | 3.004433 | 15.013630 |
| H | 9.775333 | 3.822750 | 14.940669 |
| H | 8.065915 | 3.452575 | 15.257272 |
| C | 9.464088 | 2.079328 | 16.150323 |
| H | 9.120494 | 2.493506 | 17.111457 |
| H | 10.562201 | 2.000640 | 16.193145 |
| C | 9.856851 | -0.264011 | 16.314188 |
| H | 10.570814 | -0.388363 | 15.479319 |
| H | 10.420435 | -0.009623 | 17.226691 |
| C | 9.116941 | -1.548689 | 16.564574 |
| H | 8.498909 | -1.464311 | 17.477346 |
| H | 9.860195 | -2.349050 | 16.713118 |
| C | 7.865209 | -3.205743 | 15.400736 |
| H | 8.500585 | -3.746664 | 14.681618 |
| H | 8.029289 | -3.652837 | 16.394328 |
| C | 8.121115 | 3.078348 | 12.772244 |
| H | 7.398060 | 3.685422 | 13.331836 |
| H | 8.788827 | 3.764967 | 12.215410 |
| N | 8.992638 | -0.256367 | 12.719435 |
| N | 8.871575 | 2.286108 | 13.752411 |
| O | 8.933613 | 0.760667 | 15.998331 |
| O | 8.286363 | -1.841038 | 15.456926 |
| O | 6.645648 | -1.483612 | 12.818714 |
| C | 7.414206 | -3.262989 | 11.428355 |
| H | 6.438535 | -3.753485 | 11.451152 |
| C | 8.471511 | -3.822479 | 10.681396 |
| H | 8.299635 | -4.765154 | 10.156381 |
| C | 9.700520 | -3.206189 | 10.578398 |
| H | 10.502318 | -3.637784 | 9.977101 |
| C | 6.026151 | 2.496744 | 16.396601 |
| C | 5.956978 | 3.796412 | 16.901553 |
| H | 6.112400 | 4.632123 | 16.215643 |
| C | 5.653892 | 4.039237 | 18.257248 |
| H | 5.609147 | 5.073657 | 18.606243 |
| C | 5.392852 | 3.015458 | 19.143058 |
| H | 5.135547 | 3.218423 | 20.183885 |

Dy-anti-KHQ(1+)

SCF (RwB97XD) = -1947.89729577
E(SCF)+ZPE(0 K)= -1947.214853
H(298 K)= -1947.177531
G(298 K)= -1947.280724
Lowest Frequency = 18.9282cm-1

| | | | |
|---|---|---|---|
| C | 5.784349 | 1.423899 | 17.325255 |
| C | 5.435402 | 1.678492 | 18.676013 |
| C | 5.110831 | 0.545787 | 19.467534 |
| H | 4.831747 | 0.684551 | 20.514941 |
| C | 5.109804 | -0.705562 | 18.904941 |
| H | 4.817865 | -1.583415 | 19.484850 |
| C | 5.489321 | -0.855929 | 17.547746 |
| C | 5.480964 | -2.224163 | 16.914123 |
| H | 6.319359 | -2.794835 | 17.343355 |
| H | 4.571286 | -2.764975 | 17.241679 |
| C | 4.302554 | -2.148552 | 14.771277 |
| H | 3.555761 | -2.810504 | 15.252457 |

| | | | |
|---|---|---|---|
| H | 4.465399 | -2.504588 | 13.745930 |
| C | 3.712776 | -0.751610 | 14.656923 |
| H | 2.753616 | -0.826620 | 14.118698 |
| H | 3.519140 | -0.284452 | 15.636732 |
| C | 4.116126 | 1.063893 | 13.063065 |
| H | 4.017443 | 2.002600 | 13.632357 |
| H | 3.134727 | 0.772637 | 12.654283 |
| C | 5.101990 | 1.232686 | 11.922104 |
| H | 5.149101 | 0.316380 | 11.311194 |
| H | 4.786456 | 2.081222 | 11.293131 |
| C | 7.319141 | 2.261500 | 11.770963 |
| H | 6.793511 | 2.960887 | 11.100278 |
| H | 7.948128 | 1.601789 | 11.150801 |
| C | 6.401094 | -3.334691 | 14.984130 |
| H | 6.332125 | -3.344590 | 13.894645 |
| H | 6.028316 | -4.310502 | 15.355325 |
| N | 5.855368 | 0.165644 | 16.808175 |
| N | 5.594826 | -2.214799 | 15.461762 |
| O | 6.292706 | 2.198554 | 15.162688 |
| O | 4.612797 | 0.045209 | 13.910521 |
| O | 6.372595 | 1.492330 | 12.488266 |
| C | 7.572850 | -2.059962 | 12.118363 |
| C | 8.864059 | -1.429110 | 12.038843 |
| C | 9.907364 | -1.975070 | 11.248510 |
| C | 11.097332 | -1.206754 | 11.157779 |
| H | 11.932531 | -1.578456 | 10.559048 |
| C | 11.176257 | 0.010698 | 11.785695 |
| H | 12.065242 | 0.637261 | 11.689486 |
| C | 10.082275 | 0.460644 | 12.566084 |
| C | 10.151378 | 1.800842 | 13.253444 |
| H | 10.617924 | 2.533403 | 12.565674 |
| H | 10.859310 | 1.708818 | 14.092011 |
| C | 9.031053 | 3.004433 | 15.013630 |
| H | 9.775333 | 3.822750 | 14.940669 |
| H | 8.065915 | 3.452575 | 15.257272 |
| C | 9.464088 | 2.079328 | 16.150323 |
| H | 9.120494 | 2.493506 | 17.111457 |
| H | 10.562201 | 2.000640 | 16.193145 |
| C | 9.856851 | -0.264011 | 16.314188 |
| H | 10.570814 | -0.388363 | 15.479319 |
| H | 10.420435 | -0.009623 | 17.226691 |
| C | 9.116941 | -1.548689 | 16.564574 |
| H | 8.498909 | -1.464311 | 17.477346 |
| H | 9.860195 | -2.349050 | 16.713118 |
| C | 7.865209 | -3.205743 | 15.400736 |
| H | 8.500585 | -3.746664 | 14.681618 |
| H | 8.029289 | -3.652837 | 16.394328 |
| C | 8.121115 | 3.078348 | 12.772244 |
| H | 7.398060 | 3.685422 | 13.331836 |
| H | 8.788827 | 3.764967 | 12.215410 |
| N | 8.992638 | -0.256367 | 12.719435 |
| N | 8.871575 | 2.286108 | 13.752411 |
| O | 8.933613 | 0.760667 | 15.998331 |
| O | 8.286363 | -1.841038 | 15.456926 |
| O | 6.645648 | -1.483612 | 12.818714 |
| C | 7.414206 | -3.262989 | 11.428355 |
| H | 6.438535 | -3.753485 | 11.451152 |

| | | | |
|---|---|---|---|
| C | 8.471511 | -3.822479 | 10.681396 |
| H | 8.299635 | -4.765154 | 10.156381 |
| C | 9.700520 | -3.206189 | 10.578398 |
| H | 10.502318 | -3.637784 | 9.977101 |
| C | 6.026151 | 2.496744 | 16.396601 |
| C | 5.956978 | 3.796412 | 16.901553 |
| H | 6.112400 | 4.632123 | 16.215643 |
| C | 5.653892 | 4.039237 | 18.257248 |
| H | 5.609147 | 5.073657 | 18.606243 |
| C | 5.392852 | 3.015458 | 19.143058 |
| H | 5.135547 | 3.218423 | 20.183885 |
| Dy | 7.009308 | 0.130182 | 14.427695 |

Dy3+_Ion

SCF (RwB97XD) = -35.5745387418
E(SCF)+ZPE(0 K)= -35.574539
H(298 K)= -35.572178
G(298 K)= -35.591750

| | | | |
|---|---|---|---|
| Dy | 0.887400 | 3.945900 | 9.220400 |

Dy-syn-KHQ_Ligand(2–)

SCF (RwB97XD) = -1910.49860017
E(SCF)+ZPE(0 K)= -1909.830380
H(298 K)= -1909.795428
G(298 K)= -1909.892250
Lowest Frequency = -38.3594cm-1

| | | | |
|---|---|---|---|
| N | 3.077487 | 2.633192 | 9.275769 |
| C | 3.128542 | 1.331072 | 9.450146 |
| C | 4.278456 | 0.712580 | 9.990433 |
| H | 4.289576 | -0.366297 | 10.152659 |
| C | 5.371683 | 1.487869 | 10.303286 |
| H | 6.265794 | 1.029035 | 10.732200 |
| C | 5.366676 | 2.882092 | 10.034333 |
| C | 4.165002 | 3.406508 | 9.506276 |
| C | 4.011944 | 4.795171 | 9.158863 |
| O | 2.841546 | 5.190906 | 8.738555 |
| C | 5.128683 | 5.606281 | 9.336330 |
| H | 5.069773 | 6.659621 | 9.052626 |
| C | 6.327552 | 5.084258 | 9.875206 |
| H | 7.174979 | 5.761161 | 10.007711 |
| C | 6.462686 | 3.760762 | 10.234432 |
| H | 7.398252 | 3.379319 | 10.646667 |
| N | 1.969027 | 4.404649 | 11.529020 |
| C | 2.617957 | 5.497719 | 11.861265 |
| C | 3.599906 | 5.486981 | 12.878223 |
| H | 4.143014 | 6.402636 | 13.117299 |
| C | 3.857222 | 4.314060 | 13.549646 |
| H | 4.622371 | 4.282613 | 14.329057 |
| C | 3.105399 | 3.143884 | 13.263966 |
| C | 2.152265 | 3.258551 | 12.227263 |
| C | 1.295190 | 2.171176 | 11.841209 |
| O | 0.487477 | 2.370120 | 10.835110 |
| C | 1.427125 | 0.986601 | 12.558072 |

| | | | |
|---|---|---|---|
| H | 0.773893 | 0.144277 | 12.318865 |
| C | 2.390493 | 0.866371 | 13.586759 |
| H | 2.467437 | -0.085425 | 14.117825 |
| C | 3.226795 | 1.902736 | 13.941212 |
| H | 3.960489 | 1.788592 | 14.740872 |
| C | 1.911915 | 0.554532 | 9.006047 |
| H | 2.229605 | -0.426363 | 8.598917 |
| H | 1.276515 | 0.360001 | 9.880771 |
| N | 1.131920 | 1.308567 | 8.022546 |
| C | 1.866227 | 1.438600 | 6.764670 |
| H | 1.886334 | 0.484622 | 6.200735 |
| H | 2.905515 | 1.703514 | 6.995283 |
| C | 1.296570 | 2.516687 | 5.872950 |
| H | 1.974973 | 2.658345 | 5.013715 |
| H | 0.305323 | 2.249482 | 5.463376 |
| O | 1.190464 | 3.722926 | 6.600455 |
| C | 0.869194 | 4.821218 | 5.774283 |
| H | -0.156862 | 4.701814 | 5.380110 |
| H | 1.566859 | 4.872725 | 4.920317 |
| C | 0.986765 | 6.084992 | 6.577014 |
| H | 2.024664 | 6.233224 | 6.914949 |
| H | 0.662479 | 6.934178 | 5.953554 |
| O | 0.132451 | 5.959319 | 7.707718 |
| C | -0.220769 | 7.189524 | 8.317708 |
| H | -1.241206 | 7.069041 | 8.693545 |
| H | -0.258081 | 7.982693 | 7.553257 |
| C | 0.760556 | 7.605342 | 9.404170 |
| H | 1.740444 | 7.748821 | 8.928576 |
| H | 0.445149 | 8.590797 | 9.804615 |
| N | 0.934532 | 6.615363 | 10.462191 |
| C | 2.243281 | 6.749362 | 11.107056 |
| H | 2.272311 | 7.616803 | 11.795932 |
| H | 2.996514 | 6.921531 | 10.327822 |
| C | -0.115455 | 6.616508 | 11.479111 |
| H | -0.256059 | 7.623901 | 11.920659 |
| H | 0.211787 | 5.951361 | 12.287737 |
| C | -1.462151 | 6.123536 | 10.987170 |
| H | -2.078305 | 5.837858 | 11.856918 |
| H | -2.014415 | 6.911592 | 10.446721 |
| O | -1.280842 | 5.003224 | 10.141092 |
| C | -2.475181 | 4.529321 | 9.543703 |
| H | -2.567749 | 4.949017 | 8.525818 |
| H | -3.351141 | 4.846180 | 10.131548 |
| C | -2.438306 | 3.028539 | 9.512593 |
| H | -2.330080 | 2.643314 | 10.541566 |
| H | -3.382227 | 2.645665 | 9.084814 |
| O | -1.342473 | 2.597175 | 8.733118 |
| C | -1.216976 | 1.180071 | 8.802116 |
| H | -2.187977 | 0.718871 | 8.550882 |
| H | -0.941287 | 0.907243 | 9.832140 |
| C | -0.187287 | 0.719191 | 7.803489 |
| H | -0.144960 | -0.387157 | 7.816842 |
| H | -0.540773 | 1.004070 | 6.803799 |

Dy-syn-KHQ(1+)

SCF (RwB97XD) = -1947.90817749

E(SCF)+ZPE(0 K)= -1947.224145
H(298 K)= -1947.187584
G(298 K)= -1947.286971
Lowest Frequency = 40.3200cm-1

| | | | |
|---|---|---|---|
| Dy | 0.948624 | 3.966089 | 9.242925 |
| N | 3.077487 | 2.633192 | 9.275769 |
| C | 3.128542 | 1.331072 | 9.450146 |
| C | 4.278456 | 0.712580 | 9.990433 |
| H | 4.289576 | -0.366297 | 10.152659 |
| C | 5.371683 | 1.487869 | 10.303286 |
| H | 6.265794 | 1.029035 | 10.732200 |
| C | 5.366676 | 2.882092 | 10.034333 |
| C | 4.165002 | 3.406508 | 9.506276 |
| C | 4.011944 | 4.795171 | 9.158863 |
| O | 2.841546 | 5.190906 | 8.738555 |
| C | 5.128683 | 5.606281 | 9.336330 |
| H | 5.069773 | 6.659621 | 9.052626 |
| C | 6.327552 | 5.084258 | 9.875206 |
| H | 7.174979 | 5.761161 | 10.007711 |
| C | 6.462686 | 3.760762 | 10.234432 |
| H | 7.398252 | 3.379319 | 10.646667 |
| N | 1.969027 | 4.404649 | 11.529020 |
| C | 2.617957 | 5.497719 | 11.861265 |
| C | 3.599906 | 5.486981 | 12.878223 |
| H | 4.143014 | 6.402636 | 13.117299 |
| C | 3.857222 | 4.314060 | 13.549646 |
| H | 4.622371 | 4.282613 | 14.329057 |
| C | 3.105399 | 3.143884 | 13.263966 |
| C | 2.152265 | 3.258551 | 12.227263 |
| C | 1.295190 | 2.171176 | 11.841209 |
| O | 0.487477 | 2.370120 | 10.835110 |
| C | 1.427125 | 0.986601 | 12.558072 |
| H | 0.773893 | 0.144277 | 12.318865 |
| C | 2.390493 | 0.866371 | 13.586759 |
| H | 2.467437 | -0.085425 | 14.117825 |
| C | 3.226795 | 1.902736 | 13.941212 |
| H | 3.960489 | 1.788592 | 14.740872 |
| C | 1.911915 | 0.554532 | 9.006047 |
| H | 2.229605 | -0.426363 | 8.598917 |
| H | 1.276515 | 0.360001 | 9.880771 |
| N | 1.131920 | 1.308567 | 8.022546 |
| C | 1.866227 | 1.438600 | 6.764670 |
| H | 1.886334 | 0.484622 | 6.200735 |
| H | 2.905515 | 1.703514 | 6.995283 |
| C | 1.296570 | 2.516687 | 5.872950 |
| H | 1.974973 | 2.658345 | 5.013715 |
| H | 0.305323 | 2.249482 | 5.463376 |
| O | 1.190464 | 3.722926 | 6.600455 |
| C | 0.869194 | 4.821218 | 5.774283 |
| H | -0.156862 | 4.701814 | 5.380110 |
| H | 1.566859 | 4.872725 | 4.920317 |
| C | 0.986765 | 6.084992 | 6.577014 |
| H | 2.024664 | 6.233224 | 6.914949 |
| H | 0.662479 | 6.934178 | 5.953554 |
| O | 0.132451 | 5.959319 | 7.707718 |
| C | -0.220769 | 7.189524 | 8.317708 |

| | | | |
|---|---|---|---|
| H | -1.241206 | 7.069041 | 8.693545 |
| H | -0.258081 | 7.982693 | 7.553257 |
| C | 0.760556 | 7.605342 | 9.404170 |
| H | 1.740444 | 7.748821 | 8.928576 |
| H | 0.445149 | 8.590797 | 9.804615 |
| N | 0.934532 | 6.615363 | 10.462191 |
| C | 2.243281 | 6.749362 | 11.107056 |
| H | 2.272311 | 7.616803 | 11.795932 |
| H | 2.996514 | 6.921531 | 10.327822 |
| C | -0.115455 | 6.616508 | 11.479111 |
| H | -0.256059 | 7.623901 | 11.920659 |
| H | 0.211787 | 5.951361 | 12.287737 |
| C | -1.462151 | 6.123536 | 10.987170 |
| H | -2.078305 | 5.837858 | 11.856918 |
| H | -2.014415 | 6.911592 | 10.446721 |
| O | -1.280842 | 5.003224 | 10.141092 |
| C | -2.475181 | 4.529321 | 9.543703 |
| H | -2.567749 | 4.949017 | 8.525818 |
| H | -3.351141 | 4.846180 | 10.131548 |
| C | -2.438306 | 3.028539 | 9.512593 |
| H | -2.330080 | 2.643314 | 10.541566 |
| H | -3.382227 | 2.645665 | 9.084814 |
| O | -1.342473 | 2.597175 | 8.733118 |
| C | -1.216976 | 1.180071 | 8.802116 |
| H | -2.187977 | 0.718871 | 8.550882 |
| H | -0.941287 | 0.907243 | 9.832140 |
| C | -0.187287 | 0.719191 | 7.803489 |
| H | -0.144960 | -0.387157 | 7.816842 |
| H | -0.540773 | 1.004070 | 6.803799 |

Dy-syn-H2O-KHQ(1+)

SCF (RwB97XD) = -2024.26905011
E(SCF)+ZPE(0 K)= -2023.559437
H(298 K)= -2023.520632
G(298 K)= -2023.624716
Lowest Frequency = 22.3421cm-1

| | | | |
|---|---|---|---|
| Dy | 1.017858 | 4.021475 | 9.092705 |
| N | 3.093586 | 2.621892 | 9.132507 |
| C | 3.109777 | 1.309831 | 9.217551 |
| C | 4.253038 | 0.620331 | 9.682368 |
| H | 4.237554 | -0.468476 | 9.752930 |
| C | 5.369914 | 1.337164 | 10.042495 |
| H | 6.258860 | 0.822176 | 10.414673 |
| C | 5.389587 | 2.749944 | 9.910312 |
| C | 4.199280 | 3.347334 | 9.432991 |
| C | 4.084295 | 4.767119 | 9.225292 |
| O | 2.938528 | 5.233647 | 8.808743 |
| C | 5.208544 | 5.534933 | 9.511143 |
| H | 5.169004 | 6.613830 | 9.344113 |
| C | 6.393016 | 4.937358 | 9.999144 |
| H | 7.249655 | 5.580308 | 10.214840 |
| C | 6.501454 | 3.579764 | 10.206084 |
| H | 7.425523 | 3.136696 | 10.580923 |
| N | 1.843455 | 4.489404 | 11.479470 |
| C | 2.371044 | 5.617767 | 11.895241 |

| | | | |
|---|---|---|---|
| C | 3.172405 | 5.677588 | 13.059618 |
| H | 3.603953 | 6.630326 | 13.370994 |
| C | 3.399110 | 4.528567 | 13.779078 |
| H | 4.025025 | 4.551177 | 14.674424 |
| C | 2.796825 | 3.307423 | 13.379418 |
| C | 2.009338 | 3.357370 | 12.206053 |
| C | 1.305614 | 2.208083 | 11.708656 |
| O | 0.613930 | 2.354491 | 10.615196 |
| C | 1.431197 | 1.027817 | 12.434875 |
| H | 0.894705 | 0.136065 | 12.101654 |
| C | 2.234583 | 0.974613 | 13.597210 |
| H | 2.311845 | 0.026294 | 14.134307 |
| C | 2.913075 | 2.074928 | 14.073131 |
| H | 3.523617 | 2.015476 | 14.975612 |
| C | 1.857378 | 0.579117 | 8.800827 |
| H | 2.134521 | -0.381698 | 8.322254 |
| H | 1.289209 | 0.343626 | 9.708408 |
| N | 1.016200 | 1.380957 | 7.905457 |
| C | 1.690664 | 1.489377 | 6.602809 |
| H | 1.535970 | 0.571238 | 6.002789 |
| H | 2.770458 | 1.579970 | 6.778180 |
| C | 1.268326 | 2.681809 | 5.782168 |
| H | 1.794231 | 2.638933 | 4.812022 |
| H | 0.182435 | 2.681858 | 5.578051 |
| O | 1.614078 | 3.872254 | 6.458214 |
| C | 1.448555 | 5.004633 | 5.632935 |
| H | 0.477469 | 4.938626 | 5.107288 |
| H | 2.247542 | 5.037852 | 4.870868 |
| C | 1.489134 | 6.268348 | 6.448939 |
| H | 2.444770 | 6.347951 | 6.989577 |
| H | 1.362520 | 7.128168 | 5.768618 |
| O | 0.410715 | 6.224233 | 7.376262 |
| C | 0.079830 | 7.455414 | 7.981561 |
| H | -0.987308 | 7.405574 | 8.235386 |
| H | 0.194269 | 8.279439 | 7.256244 |
| C | 0.940422 | 7.744035 | 9.194980 |
| H | 1.981533 | 7.807822 | 8.851462 |
| H | 0.669023 | 8.740621 | 9.598929 |
| N | 0.893871 | 6.714331 | 10.235291 |
| C | 2.106369 | 6.836611 | 11.055993 |
| H | 2.061475 | 7.734966 | 11.702753 |
| H | 2.960892 | 6.958898 | 10.378070 |
| C | -0.290121 | 6.787895 | 11.097105 |
| H | -0.557458 | 7.841119 | 11.316415 |
| H | -0.034915 | 6.317107 | 12.056332 |
| C | -1.537681 | 6.085802 | 10.597494 |
| H | -2.310525 | 6.211097 | 11.376052 |
| H | -1.946644 | 6.534103 | 9.675340 |
| O | -1.296100 | 4.707684 | 10.390094 |
| C | -2.498025 | 3.954001 | 10.427184 |
| H | -3.163079 | 4.274536 | 9.601199 |
| H | -3.026409 | 4.162708 | 11.373685 |
| C | -2.254636 | 2.467557 | 10.352165 |
| H | -1.541364 | 2.163874 | 11.132827 |
| H | -3.221931 | 1.956248 | 10.510930 |
| O | -1.728835 | 2.145956 | 9.085416 |
| C | -1.243908 | 0.828597 | 8.928504 |

| | | | |
|---|---|---|---|
| H | -2.089614 | 0.141669 | 8.744734 |
| H | -0.738139 | 0.522358 | 9.855443 |
| C | -0.303980 | 0.757346 | 7.737217 |
| H | -0.184848 | -0.312056 | 7.468605 |
| H | -0.804205 | 1.235818 | 6.882198 |
| O | -0.929688 | 3.950575 | 7.512439 |
| H | -1.607953 | 3.361202 | 7.895554 |
| H | -1.298382 | 4.780322 | 7.195715 |

Er-anti-KHQ-Ligand(2–)

SCF (RwB97XD) = -1910.47998499
E(SCF)+ZPE(0 K)= -1909.814116
H(298 K)= -1909.779809
G(298 K)= -1909.877020
Lowest Frequency = -69.1480cm-1

| | | | |
|---|---|---|---|
| C | 5.802381 | 1.416955 | 17.312594 |
| C | 5.460667 | 1.674548 | 18.664392 |
| C | 5.151858 | 0.542463 | 19.462982 |
| H | 4.879760 | 0.682776 | 20.512006 |
| C | 5.155535 | -0.710673 | 18.904307 |
| H | 4.873983 | -1.588707 | 19.489011 |
| C | 5.524368 | -0.862983 | 17.544491 |
| C | 5.516948 | -2.231146 | 16.911867 |
| H | 6.374556 | -2.791132 | 17.316894 |
| H | 4.623344 | -2.784407 | 17.262472 |
| C | 4.287209 | -2.136186 | 14.798170 |
| H | 3.540869 | -2.781438 | 15.302242 |
| H | 4.418822 | -2.505533 | 13.773117 |
| C | 3.716372 | -0.731207 | 14.683374 |
| H | 2.751763 | -0.794847 | 14.153681 |
| H | 3.536901 | -0.257371 | 15.662320 |
| C | 4.125478 | 1.061505 | 13.067368 |
| H | 4.026104 | 2.005932 | 13.626831 |
| H | 3.144697 | 0.766124 | 12.660258 |
| C | 5.112815 | 1.217737 | 11.926161 |
| H | 5.158444 | 0.296143 | 11.323350 |
| H | 4.800538 | 2.061967 | 11.290010 |
| C | 7.329729 | 2.244180 | 11.769737 |
| H | 6.803108 | 2.934912 | 11.091055 |
| H | 7.961814 | 1.580742 | 11.157116 |
| C | 6.385770 | -3.334242 | 14.957568 |
| H | 6.281742 | -3.348735 | 13.871207 |
| H | 6.027933 | -4.309808 | 15.343668 |
| N | 5.879093 | 0.158116 | 16.798691 |
| N | 5.593593 | -2.215893 | 15.458125 |
| O | 6.293686 | 2.182581 | 15.144878 |
| O | 4.620233 | 0.051251 | 13.926230 |
| O | 6.383688 | 1.478398 | 12.491556 |
| C | 7.563968 | -2.044648 | 12.134620 |
| C | 8.860803 | -1.424814 | 12.069329 |
| C | 9.908453 | -1.979378 | 11.291234 |
| C | 11.105534 | -1.220653 | 11.214229 |
| H | 11.945286 | -1.599542 | 10.626488 |
| C | 11.185046 | -0.002346 | 11.840356 |
| H | 12.079165 | 0.618214 | 11.753327 |

| | | | |
|---|---|---|---|
| C | 10.084544 | 0.457723 | 12.605556 |
| C | 10.151090 | 1.801155 | 13.286152 |
| H | 10.632593 | 2.527084 | 12.601458 |
| H | 10.844568 | 1.711469 | 14.137348 |
| C | 9.008616 | 3.016053 | 15.020009 |
| H | 9.770065 | 3.819377 | 14.959821 |
| H | 8.047926 | 3.485425 | 15.238623 |
| C | 9.394660 | 2.086485 | 16.173477 |
| H | 8.990300 | 2.485382 | 17.117024 |
| H | 10.489546 | 2.026151 | 16.276257 |
| C | 9.838808 | -0.256631 | 16.273963 |
| H | 10.544451 | -0.358432 | 15.429307 |
| H | 10.409085 | -0.009493 | 17.184010 |
| C | 9.117617 | -1.554486 | 16.510668 |
| H | 8.515777 | -1.496130 | 17.436065 |
| H | 9.871121 | -2.350353 | 16.627821 |
| C | 7.863778 | -3.197444 | 15.326030 |
| H | 8.480415 | -3.704744 | 14.567260 |
| H | 8.068951 | -3.673349 | 16.298162 |
| C | 8.127072 | 3.073668 | 12.764377 |
| H | 7.402164 | 3.691955 | 13.308987 |
| H | 8.800503 | 3.749862 | 12.201554 |
| N | 8.988900 | -0.252074 | 12.749518 |
| N | 8.866021 | 2.293241 | 13.761029 |
| O | 8.896659 | 0.759011 | 15.986082 |
| O | 8.270200 | -1.829542 | 15.411850 |
| O | 6.636640 | -1.461868 | 12.829119 |
| C | 7.400695 | -3.243611 | 11.438853 |
| H | 6.419951 | -3.724287 | 11.448192 |
| C | 8.461728 | -3.812044 | 10.703803 |
| H | 8.286976 | -4.751779 | 10.174502 |
| C | 9.698138 | -3.208067 | 10.617644 |
| H | 10.503000 | -3.646740 | 10.025639 |
| C | 6.028006 | 2.486857 | 16.377314 |
| C | 5.945866 | 3.788645 | 16.874280 |
| H | 6.086469 | 4.622270 | 16.182687 |
| C | 5.649865 | 4.035365 | 18.231032 |
| H | 5.595536 | 5.071107 | 18.574663 |
| C | 5.407654 | 3.013647 | 19.124510 |
| H | 5.156043 | 3.219634 | 20.166130 |

Er-anti-KHQ(1+)

SCF (RwB97XD) = -1949.06281829
E(SCF)+ZPE(0 K)= -1948.380232
H(298 K)= -1948.342976
G(298 K)= -1948.446060
Lowest Frequency = 14.5571cm-1

| | | | |
|---|---|---|---|
| C | 5.802381 | 1.416955 | 17.312594 |
| C | 5.460667 | 1.674548 | 18.664392 |
| C | 5.151858 | 0.542463 | 19.462982 |
| H | 4.879760 | 0.682776 | 20.512006 |
| C | 5.155535 | -0.710673 | 18.904307 |
| H | 4.873983 | -1.588707 | 19.489011 |
| C | 5.524368 | -0.862983 | 17.544491 |
| C | 5.516948 | -2.231146 | 16.911867 |

| | | | |
|---|---|---|---|
| H | 6.374556 | -2.791132 | 17.316894 |
| H | 4.623344 | -2.784407 | 17.262472 |
| C | 4.287209 | -2.136186 | 14.798170 |
| H | 3.540869 | -2.781438 | 15.302242 |
| H | 4.418822 | -2.505533 | 13.773117 |
| C | 3.716372 | -0.731207 | 14.683374 |
| H | 2.751763 | -0.794847 | 14.153681 |
| H | 3.536901 | -0.257371 | 15.662320 |
| C | 4.125478 | 1.061505 | 13.067368 |
| H | 4.026104 | 2.005932 | 13.626831 |
| H | 3.144697 | 0.766124 | 12.660258 |
| C | 5.112815 | 1.217737 | 11.926161 |
| H | 5.158444 | 0.296143 | 11.323350 |
| H | 4.800538 | 2.061967 | 11.290010 |
| C | 7.329729 | 2.244180 | 11.769737 |
| H | 6.803108 | 2.934912 | 11.091055 |
| H | 7.961814 | 1.580742 | 11.157116 |
| C | 6.385770 | -3.334242 | 14.957568 |
| H | 6.281742 | -3.348735 | 13.871207 |
| H | 6.027933 | -4.309808 | 15.343668 |
| N | 5.879093 | 0.158116 | 16.798691 |
| N | 5.593593 | -2.215893 | 15.458125 |
| O | 6.293686 | 2.182581 | 15.144878 |
| O | 4.620233 | 0.051251 | 13.926230 |
| O | 6.383688 | 1.478398 | 12.491556 |
| C | 7.563968 | -2.044648 | 12.134620 |
| C | 8.860803 | -1.424814 | 12.069329 |
| C | 9.908453 | -1.979378 | 11.291234 |
| C | 11.105534 | -1.220653 | 11.214229 |
| H | 11.945286 | -1.599542 | 10.626488 |
| C | 11.185046 | -0.002346 | 11.840356 |
| H | 12.079165 | 0.618214 | 11.753327 |
| C | 10.084544 | 0.457723 | 12.605556 |
| C | 10.151090 | 1.801155 | 13.286152 |
| H | 10.632593 | 2.527084 | 12.601458 |
| H | 10.844568 | 1.711469 | 14.137348 |
| C | 9.008616 | 3.016053 | 15.020009 |
| H | 9.770065 | 3.819377 | 14.959821 |
| H | 8.047926 | 3.485425 | 15.238623 |
| C | 9.394660 | 2.086485 | 16.173477 |
| H | 8.990300 | 2.485382 | 17.117024 |
| H | 10.489546 | 2.026151 | 16.276257 |
| C | 9.838808 | -0.256631 | 16.273963 |
| H | 10.544451 | -0.358432 | 15.429307 |
| H | 10.409085 | -0.009493 | 17.184010 |
| C | 9.117617 | -1.554486 | 16.510668 |
| H | 8.515777 | -1.496130 | 17.436065 |
| H | 9.871121 | -2.350353 | 16.627821 |
| C | 7.863778 | -3.197444 | 15.326030 |
| H | 8.480415 | -3.704744 | 14.567260 |
| H | 8.068951 | -3.673349 | 16.298162 |
| C | 8.127072 | 3.073668 | 12.764377 |
| H | 7.402164 | 3.691955 | 13.308987 |
| H | 8.800503 | 3.749862 | 12.201554 |
| N | 8.988900 | -0.252074 | 12.749518 |
| N | 8.866021 | 2.293241 | 13.761029 |
| O | 8.896659 | 0.759011 | 15.986082 |

| | | | |
|---|---|---|---|
| O | 8.270200 | -1.829542 | 15.411850 |
| O | 6.636640 | -1.461868 | 12.829119 |
| C | 7.400695 | -3.243611 | 11.438853 |
| H | 6.419951 | -3.724287 | 11.448192 |
| C | 8.461728 | -3.812044 | 10.703803 |
| H | 8.286976 | -4.751779 | 10.174502 |
| C | 9.698138 | -3.208067 | 10.617644 |
| H | 10.503000 | -3.646740 | 10.025639 |
| C | 6.028006 | 2.486857 | 16.377314 |
| C | 5.945866 | 3.788645 | 16.874280 |
| H | 6.086469 | 4.622270 | 16.182687 |
| C | 5.649865 | 4.035365 | 18.231032 |
| H | 5.595536 | 5.071107 | 18.574663 |
| C | 5.407654 | 3.013647 | 19.124510 |
| H | 5.156043 | 3.219634 | 20.166130 |
| Er | 6.998241 | 0.133823 | 14.421425 |

Er3+_Ion

SCF (RwB97XD) = -36.7207183585
E(SCF)+ZPE(0 K)= -36.720718
H(298 K)= -36.718358
G(298 K)= -36.737946

| | | | |
|---|---|---|---|
| Er | 0.887400 | 3.945900 | 9.220400 |

Er-syn-KHQ_Ligand(2–)

SCF (RwB97XD) = -1910.49525541
E(SCF)+ZPE(0 K)= -1909.826993
H(298 K)= -1909.792062
G(298 K)= -1909.888826
Lowest Frequency = -39.2690cm-1

| | | | |
|---|---|---|---|
| N | 3.067758 | 2.630927 | 9.307560 |
| C | 3.124047 | 1.331578 | 9.502337 |
| C | 4.278317 | 0.725480 | 10.047090 |
| H | 4.292587 | -0.350549 | 10.226952 |
| C | 5.372187 | 1.507782 | 10.339795 |
| H | 6.269773 | 1.058088 | 10.771121 |
| C | 5.363152 | 2.896365 | 10.044018 |
| C | 4.156711 | 3.408487 | 9.515512 |
| C | 3.999036 | 4.788563 | 9.140217 |
| O | 2.823385 | 5.171542 | 8.722613 |
| C | 5.115678 | 5.604935 | 9.289282 |
| H | 5.053205 | 6.651657 | 8.982816 |
| C | 6.319815 | 5.095827 | 9.829337 |
| H | 7.167596 | 5.776230 | 9.939667 |
| C | 6.459544 | 3.780672 | 10.216150 |
| H | 7.399162 | 3.409300 | 10.628344 |
| N | 1.974548 | 4.425747 | 11.511860 |
| C | 2.628184 | 5.518558 | 11.836395 |
| C | 3.602776 | 5.513266 | 12.860308 |
| H | 4.150495 | 6.428175 | 13.091573 |
| C | 3.845636 | 4.347805 | 13.550075 |
| H | 4.604437 | 4.321112 | 14.335841 |
| C | 3.084298 | 3.181088 | 13.276231 |

| | | | |
|---|---|---|---|
| C | 2.141666 | 3.289728 | 12.229797 |
| C | 1.274168 | 2.207958 | 11.854493 |
| O | 0.479156 | 2.403706 | 10.837538 |
| C | 1.382820 | 1.034190 | 12.592050 |
| H | 0.719801 | 0.196905 | 12.362357 |
| C | 2.336588 | 0.918698 | 13.630575 |
| H | 2.396250 | -0.024805 | 14.178362 |
| C | 3.183990 | 1.949530 | 13.974486 |
| H | 3.909308 | 1.838994 | 14.782248 |
| C | 1.911013 | 0.544054 | 9.068835 |
| H | 2.232936 | -0.444500 | 8.683987 |
| H | 1.269669 | 0.366140 | 9.942692 |
| N | 1.139069 | 1.278694 | 8.066301 |
| C | 1.881089 | 1.390111 | 6.812363 |
| H | 1.904143 | 0.429213 | 6.260295 |
| H | 2.919320 | 1.657641 | 7.044528 |
| C | 1.315206 | 2.457087 | 5.905792 |
| H | 1.999016 | 2.593035 | 5.049891 |
| H | 0.327948 | 2.181968 | 5.491825 |
| O | 1.198840 | 3.670472 | 6.619649 |
| C | 0.861451 | 4.751109 | 5.777354 |
| H | -0.168158 | 4.617857 | 5.396961 |
| H | 1.548914 | 4.791214 | 4.914538 |
| C | 0.979646 | 6.030046 | 6.554152 |
| H | 2.020047 | 6.192346 | 6.876758 |
| H | 0.641932 | 6.864557 | 5.918211 |
| O | 0.139748 | 5.920670 | 7.696855 |
| C | -0.211798 | 7.162922 | 8.283640 |
| H | -1.232037 | 7.052776 | 8.663025 |
| H | -0.248952 | 7.940707 | 7.503744 |
| C | 0.771854 | 7.598511 | 9.359773 |
| H | 1.750206 | 7.734526 | 8.878947 |
| H | 0.456262 | 8.590291 | 9.744354 |
| N | 0.949052 | 6.625879 | 10.431852 |
| C | 2.259107 | 6.764439 | 11.070499 |
| H | 2.292915 | 7.637895 | 11.751568 |
| H | 3.010997 | 6.927471 | 10.288128 |
| C | -0.098859 | 6.637248 | 11.449703 |
| H | -0.243755 | 7.649674 | 11.878268 |
| H | 0.230990 | 5.984317 | 12.267061 |
| C | -1.442815 | 6.132308 | 10.962260 |
| H | -2.056266 | 5.843080 | 11.832666 |
| H | -2.000701 | 6.915965 | 10.421287 |
| O | -1.255676 | 5.012812 | 10.115927 |
| C | -2.444697 | 4.552713 | 9.497544 |
| H | -2.507998 | 4.964707 | 8.474292 |
| H | -3.327856 | 4.888313 | 10.063555 |
| C | -2.429887 | 3.052106 | 9.479245 |
| H | -2.352001 | 2.674443 | 10.513727 |
| H | -3.368836 | 2.678792 | 9.032433 |
| O | -1.322171 | 2.599352 | 8.729999 |
| C | -1.214189 | 1.182499 | 8.827021 |
| H | -2.187988 | 0.728142 | 8.574273 |
| H | -0.952984 | 0.926430 | 9.865008 |
| C | -0.179675 | 0.690014 | 7.849292 |
| H | -0.143979 | -0.415880 | 7.891040 |
| H | -0.522721 | 0.950982 | 6.839541 |

Er-syn-KHQ(1+)

SCF (RwB97XD) = -1949.07221369
E(SCF)+ZPE(0 K)= -1948.388008
H(298 K)= -1948.351482
G(298 K)= -1948.450720
Lowest Frequency = 38.3129cm-1

| | | | |
|---|---|---|---|
| Er | 0.961654 | 3.959611 | 9.250540 |
| N | 3.067758 | 2.630927 | 9.307560 |
| C | 3.124047 | 1.331578 | 9.502337 |
| C | 4.278317 | 0.725480 | 10.047090 |
| H | 4.292587 | -0.350549 | 10.226952 |
| C | 5.372187 | 1.507782 | 10.339795 |
| H | 6.269773 | 1.058088 | 10.771121 |
| C | 5.363152 | 2.896365 | 10.044018 |
| C | 4.156711 | 3.408487 | 9.515512 |
| C | 3.999036 | 4.788563 | 9.140217 |
| O | 2.823385 | 5.171542 | 8.722613 |
| C | 5.115678 | 5.604935 | 9.289282 |
| H | 5.053205 | 6.651657 | 8.982816 |
| C | 6.319815 | 5.095827 | 9.829337 |
| H | 7.167596 | 5.776230 | 9.939667 |
| C | 6.459544 | 3.780672 | 10.216150 |
| H | 7.399162 | 3.409300 | 10.628344 |
| N | 1.974548 | 4.425747 | 11.511860 |
| C | 2.628184 | 5.518558 | 11.836395 |
| C | 3.602776 | 5.513266 | 12.860308 |
| H | 4.150495 | 6.428175 | 13.091573 |
| C | 3.845636 | 4.347805 | 13.550075 |
| H | 4.604437 | 4.321112 | 14.335841 |
| C | 3.084298 | 3.181088 | 13.276231 |
| C | 2.141666 | 3.289728 | 12.229797 |
| C | 1.274168 | 2.207958 | 11.854493 |
| O | 0.479156 | 2.403706 | 10.837538 |
| C | 1.382820 | 1.034190 | 12.592050 |
| H | 0.719801 | 0.196905 | 12.362357 |
| C | 2.336588 | 0.918698 | 13.630575 |
| H | 2.396250 | -0.024805 | 14.178362 |
| C | 3.183990 | 1.949530 | 13.974486 |
| H | 3.909308 | 1.838994 | 14.782248 |
| C | 1.911013 | 0.544054 | 9.068835 |
| H | 2.232936 | -0.444500 | 8.683987 |
| H | 1.269669 | 0.366140 | 9.942692 |
| N | 1.139069 | 1.278694 | 8.066301 |
| C | 1.881089 | 1.390111 | 6.812363 |
| H | 1.904143 | 0.429213 | 6.260295 |
| H | 2.919320 | 1.657641 | 7.044528 |
| C | 1.315206 | 2.457087 | 5.905792 |
| H | 1.999016 | 2.593035 | 5.049891 |
| H | 0.327948 | 2.181968 | 5.491825 |
| O | 1.198840 | 3.670472 | 6.619649 |
| C | 0.861451 | 4.751109 | 5.777354 |
| H | -0.168158 | 4.617857 | 5.396961 |
| H | 1.548914 | 4.791214 | 4.914538 |
| C | 0.979646 | 6.030046 | 6.554152 |

| | | | |
|---|---|---|---|
| H | 2.020047 | 6.192346 | 6.876758 |
| H | 0.641932 | 6.864557 | 5.918211 |
| O | 0.139748 | 5.920670 | 7.696855 |
| C | -0.211798 | 7.162922 | 8.283640 |
| H | -1.232037 | 7.052776 | 8.663025 |
| H | -0.248952 | 7.940707 | 7.503744 |
| C | 0.771854 | 7.598511 | 9.359773 |
| H | 1.750206 | 7.734526 | 8.878947 |
| H | 0.456262 | 8.590291 | 9.744354 |
| N | 0.949052 | 6.625879 | 10.431852 |
| C | 2.259107 | 6.764439 | 11.070499 |
| H | 2.292915 | 7.637895 | 11.751568 |
| H | 3.010997 | 6.927471 | 10.288128 |
| C | -0.098859 | 6.637248 | 11.449703 |
| H | -0.243755 | 7.649674 | 11.878268 |
| H | 0.230990 | 5.984317 | 12.267061 |
| C | -1.442815 | 6.132308 | 10.962260 |
| H | -2.056266 | 5.843080 | 11.832666 |
| H | -2.000701 | 6.915965 | 10.421287 |
| O | -1.255676 | 5.012812 | 10.115927 |
| C | -2.444697 | 4.552713 | 9.497544 |
| H | -2.507998 | 4.964707 | 8.474292 |
| H | -3.327856 | 4.888313 | 10.063555 |
| C | -2.429887 | 3.052106 | 9.479245 |
| H | -2.352001 | 2.674443 | 10.513727 |
| H | -3.368836 | 2.678792 | 9.032433 |
| O | -1.322171 | 2.599352 | 8.729999 |
| C | -1.214189 | 1.182499 | 8.827021 |
| H | -2.187988 | 0.728142 | 8.574273 |
| H | -0.952984 | 0.926430 | 9.865008 |
| C | -0.179675 | 0.690014 | 7.849292 |
| H | -0.143979 | -0.415880 | 7.891040 |
| H | -0.522721 | 0.950982 | 6.839541 |

Er-syn-H2O-KHQ(1+)

SCF (RwB97XD) = -2025.43321683
E(SCF)+ZPE(0 K)= -2024.723033
H(298 K)= -2024.684419
G(298 K)= -2024.788122
Lowest Frequency = 22.2619cm-1

| | | | |
|---|---|---|---|
| Er | 1.052664 | 4.020938 | 9.101749 |
| N | 3.098357 | 2.626794 | 9.128092 |
| C | 3.116523 | 1.314064 | 9.207885 |
| C | 4.266126 | 0.624480 | 9.656476 |
| H | 4.252403 | -0.464620 | 9.722689 |
| C | 5.387425 | 1.340522 | 10.004733 |
| H | 6.281289 | 0.824559 | 10.363548 |
| C | 5.404893 | 2.753537 | 9.875959 |
| C | 4.208183 | 3.351114 | 9.416025 |
| C | 4.089789 | 4.770345 | 9.210560 |
| O | 2.937503 | 5.233720 | 8.807314 |
| C | 5.216100 | 5.539394 | 9.482409 |
| H | 5.173551 | 6.618359 | 9.316805 |
| C | 6.407523 | 4.941930 | 9.954045 |
| H | 7.266454 | 5.585265 | 10.159084 |

| | | | |
|---|---|---|---|
| C | 6.519930 | 3.584175 | 10.157627 |
| H | 7.449499 | 3.141563 | 10.519145 |
| N | 1.849827 | 4.488796 | 11.468950 |
| C | 2.369136 | 5.617138 | 11.895657 |
| C | 3.145005 | 5.676052 | 13.077053 |
| H | 3.570789 | 6.628422 | 13.397323 |
| C | 3.352558 | 4.527499 | 13.803519 |
| H | 3.957945 | 4.550245 | 14.712868 |
| C | 2.754093 | 3.307988 | 13.393557 |
| C | 1.994661 | 3.358758 | 12.202338 |
| C | 1.291839 | 2.214674 | 11.693668 |
| O | 0.623627 | 2.367373 | 10.586363 |
| C | 1.390414 | 1.035863 | 12.425679 |
| H | 0.853288 | 0.147404 | 12.084789 |
| C | 2.167808 | 0.980414 | 13.605933 |
| H | 2.224925 | 0.033499 | 14.147955 |
| C | 2.844553 | 2.076636 | 14.093495 |
| H | 3.433543 | 2.015093 | 15.010035 |
| C | 1.862245 | 0.582998 | 8.797630 |
| H | 2.138657 | -0.377041 | 8.316922 |
| H | 1.298532 | 0.345047 | 9.707156 |
| N | 1.017716 | 1.385588 | 7.907151 |
| C | 1.687474 | 1.496421 | 6.602709 |
| H | 1.527102 | 0.581478 | 5.999230 |
| H | 2.768489 | 1.582095 | 6.773223 |
| C | 1.266213 | 2.693670 | 5.789344 |
| H | 1.784452 | 2.650770 | 4.815073 |
| H | 0.178774 | 2.699569 | 5.593569 |
| O | 1.622832 | 3.881079 | 6.465247 |
| C | 1.455370 | 5.012538 | 5.638789 |
| H | 0.485563 | 4.942686 | 5.111191 |
| H | 2.255453 | 5.047403 | 4.877941 |
| C | 1.487893 | 6.278176 | 6.451139 |
| H | 2.440876 | 6.362272 | 6.995558 |
| H | 1.362719 | 7.135488 | 5.767191 |
| O | 0.405067 | 6.233973 | 7.372421 |
| C | 0.082170 | 7.464122 | 7.983266 |
| H | -0.983794 | 7.418378 | 8.242605 |
| H | 0.196544 | 8.290346 | 7.260306 |
| C | 0.950333 | 7.744350 | 9.192911 |
| H | 1.990432 | 7.803318 | 8.845489 |
| H | 0.686425 | 8.741187 | 9.601247 |
| N | 0.902276 | 6.712223 | 10.230472 |
| C | 2.113193 | 6.835308 | 11.053359 |
| H | 2.068098 | 7.734762 | 11.698461 |
| H | 2.969595 | 6.954826 | 10.377344 |
| C | -0.284091 | 6.785640 | 11.089656 |
| H | -0.559036 | 7.839194 | 11.297192 |
| H | -0.028296 | 6.327374 | 12.054809 |
| C | -1.525529 | 6.069050 | 10.595590 |
| H | -2.303878 | 6.206779 | 11.366794 |
| H | -1.929947 | 6.499107 | 9.662575 |
| O | -1.277934 | 4.689192 | 10.415114 |
| C | -2.480531 | 3.936874 | 10.453481 |
| H | -3.147873 | 4.260318 | 9.630332 |
| H | -3.005915 | 4.145365 | 11.401907 |
| C | -2.246970 | 2.448817 | 10.373370 |

| | | | |
|---|---|---|---|
| H | -1.518765 | 2.141915 | 11.138579 |
| H | -3.213657 | 1.944389 | 10.555648 |
| O | -1.755181 | 2.120549 | 9.094454 |
| C | -1.241751 | 0.814528 | 8.934040 |
| H | -2.073448 | 0.110856 | 8.750328 |
| H | -0.728670 | 0.515739 | 9.859206 |
| C | -0.302231 | 0.760258 | 7.740590 |
| H | -0.179749 | -0.306357 | 7.462147 |
| H | -0.807258 | 1.243372 | 6.891025 |
| O | -0.880810 | 3.937545 | 7.576990 |
| H | -1.558977 | 3.341803 | 7.955464 |
| H | -1.247119 | 4.765862 | 7.253169 |

Eu-anti-KHQ-Ligand(2–)

SCF (RwB97XD) = -1910.49304572
E(SCF)+ZPE(0 K)= -1909.826943
H(298 K)= -1909.792771
G(298 K)= -1909.889425
Lowest Frequency = -58.3213cm-1

| | | | |
|---|---|---|---|
| C | 5.893881 | 1.358735 | 17.382362 |
| C | 5.770320 | 1.550629 | 18.782360 |
| C | 5.498722 | 0.391240 | 19.555298 |
| H | 5.409693 | 0.479421 | 20.640986 |
| C | 5.308871 | -0.819687 | 18.936486 |
| H | 5.048555 | -1.710442 | 19.512002 |
| C | 5.434924 | -0.904254 | 17.527371 |
| C | 5.049989 | -2.176335 | 16.810013 |
| H | 5.428090 | -3.049829 | 17.367191 |
| H | 3.950868 | -2.255958 | 16.892703 |
| C | 4.301869 | -2.262079 | 14.493000 |
| H | 3.547419 | -3.010168 | 14.807977 |
| H | 4.677581 | -2.539321 | 13.500374 |
| C | 3.626916 | -0.907915 | 14.362553 |
| H | 2.736927 | -1.015946 | 13.721889 |
| H | 3.298222 | -0.507982 | 15.337989 |
| C | 4.057259 | 0.954990 | 12.836974 |
| H | 3.857427 | 1.905891 | 13.357596 |
| H | 3.129176 | 0.596223 | 12.363222 |
| C | 5.117221 | 1.146385 | 11.771088 |
| H | 5.276174 | 0.213241 | 11.205552 |
| H | 4.799853 | 1.947183 | 11.082706 |
| C | 7.275671 | 2.275054 | 11.716019 |
| H | 6.760354 | 2.968338 | 11.030567 |
| H | 7.914562 | 1.609183 | 11.112344 |
| C | 6.402889 | -3.303606 | 15.140766 |
| H | 6.601756 | -3.315821 | 14.064654 |
| H | 6.010116 | -4.301423 | 15.424967 |
| N | 5.773337 | 0.134971 | 16.798301 |
| N | 5.445636 | -2.233742 | 15.406549 |
| O | 6.204518 | 2.225424 | 15.210532 |
| O | 4.535496 | -0.000416 | 13.764530 |
| O | 6.315061 | 1.517211 | 12.427977 |
| C | 7.605503 | -2.085584 | 12.101559 |
| C | 8.871886 | -1.411398 | 11.996871 |
| C | 9.913448 | -1.920921 | 11.179613 |

| | | | |
|---|---|---|---|
| C | 11.074113 | -1.113428 | 11.060982 |
| H | 11.905227 | -1.454969 | 10.439069 |
| C | 11.129355 | 0.103060 | 11.693391 |
| H | 11.994962 | 0.758433 | 11.578251 |
| C | 10.043085 | 0.512348 | 12.506014 |
| C | 10.097383 | 1.844712 | 13.209036 |
| H | 10.565149 | 2.589635 | 12.535529 |
| H | 10.798409 | 1.741703 | 14.051464 |
| C | 8.971906 | 3.065509 | 14.954297 |
| H | 9.685361 | 3.908002 | 14.852048 |
| H | 7.995368 | 3.483008 | 15.207622 |
| C | 9.458029 | 2.180328 | 16.094879 |
| H | 9.169875 | 2.632418 | 17.057891 |
| H | 10.556675 | 2.090086 | 16.081237 |
| C | 9.788216 | -0.143484 | 16.454022 |
| H | 10.518765 | -0.371601 | 15.655998 |
| H | 10.337798 | 0.188916 | 17.349900 |
| C | 9.002571 | -1.369840 | 16.820967 |
| H | 8.357015 | -1.165696 | 17.695394 |
| H | 9.712475 | -2.171434 | 17.085829 |
| C | 7.696156 | -3.095466 | 15.900107 |
| H | 8.452652 | -3.818791 | 15.553732 |
| H | 7.542180 | -3.264203 | 16.979924 |
| C | 8.057364 | 3.100700 | 12.722874 |
| H | 7.320258 | 3.696347 | 13.277686 |
| H | 8.718640 | 3.799066 | 12.172938 |
| N | 8.979121 | -0.238324 | 12.681726 |
| N | 8.812775 | 2.319849 | 13.707884 |
| O | 8.904855 | 0.867335 | 16.009329 |
| O | 8.194937 | -1.770087 | 15.729144 |
| O | 6.669486 | -1.534444 | 12.813178 |
| C | 7.473694 | -3.296330 | 11.421592 |
| H | 6.518653 | -3.823601 | 11.474808 |
| C | 8.531034 | -3.819585 | 10.648924 |
| H | 8.380835 | -4.769828 | 10.131034 |
| C | 9.732888 | -3.158272 | 10.513240 |
| H | 10.534443 | -3.561168 | 9.892023 |
| C | 6.094321 | 2.466937 | 16.481359 |
| C | 6.167973 | 3.736538 | 17.058776 |
| H | 6.275369 | 4.603201 | 16.402737 |
| C | 6.077708 | 3.914875 | 18.454967 |
| H | 6.146246 | 4.928350 | 18.857626 |
| C | 5.890404 | 2.856558 | 19.318665 |
| H | 5.806267 | 3.011509 | 20.395545 |

Eu-anti-KHQ(1+)

SCF (RwB97XD) = -1946.12802516
E(SCF)+ZPE(0 K)= -1945.445655
H(298 K)= -1945.408424
G(298 K)= -1945.511570
Lowest Frequency = 13.7953cm-1

| | | | |
|---|---|---|---|
| C | 5.893881 | 1.358735 | 17.382362 |
| C | 5.770320 | 1.550629 | 18.782360 |
| C | 5.498722 | 0.391240 | 19.555298 |
| H | 5.409693 | 0.479421 | 20.640986 |

| | | | |
|---|---|---|---|
| C | 5.308871 | -0.819687 | 18.936486 |
| H | 5.048555 | -1.710442 | 19.512002 |
| C | 5.434924 | -0.904254 | 17.527371 |
| C | 5.049989 | -2.176335 | 16.810013 |
| H | 5.428090 | -3.049829 | 17.367191 |
| H | 3.950868 | -2.255958 | 16.892703 |
| C | 4.301869 | -2.262079 | 14.493000 |
| H | 3.547419 | -3.010168 | 14.807977 |
| H | 4.677581 | -2.539321 | 13.500374 |
| C | 3.626916 | -0.907915 | 14.362553 |
| H | 2.736927 | -1.015946 | 13.721889 |
| H | 3.298222 | -0.507982 | 15.337989 |
| C | 4.057259 | 0.954990 | 12.836974 |
| H | 3.857427 | 1.905891 | 13.357596 |
| H | 3.129176 | 0.596223 | 12.363222 |
| C | 5.117221 | 1.146385 | 11.771088 |
| H | 5.276174 | 0.213241 | 11.205552 |
| H | 4.799853 | 1.947183 | 11.082706 |
| C | 7.275671 | 2.275054 | 11.716019 |
| H | 6.760354 | 2.968338 | 11.030567 |
| H | 7.914562 | 1.609183 | 11.112344 |
| C | 6.402889 | -3.303606 | 15.140766 |
| H | 6.601756 | -3.315821 | 14.064654 |
| H | 6.010116 | -4.301423 | 15.424967 |
| N | 5.773337 | 0.134971 | 16.798301 |
| N | 5.445636 | -2.233742 | 15.406549 |
| O | 6.204518 | 2.225424 | 15.210532 |
| O | 4.535496 | -0.000416 | 13.764530 |
| O | 6.315061 | 1.517211 | 12.427977 |
| C | 7.605503 | -2.085584 | 12.101559 |
| C | 8.871886 | -1.411398 | 11.996871 |
| C | 9.913448 | -1.920921 | 11.179613 |
| C | 11.074113 | -1.113428 | 11.060982 |
| H | 11.905227 | -1.454969 | 10.439069 |
| C | 11.129355 | 0.103060 | 11.693391 |
| H | 11.994962 | 0.758433 | 11.578251 |
| C | 10.043085 | 0.512348 | 12.506014 |
| C | 10.097383 | 1.844712 | 13.209036 |
| H | 10.565149 | 2.589635 | 12.535529 |
| H | 10.798409 | 1.741703 | 14.051464 |
| C | 8.971906 | 3.065509 | 14.954297 |
| H | 9.685361 | 3.908002 | 14.852048 |
| H | 7.995368 | 3.483008 | 15.207622 |
| C | 9.458029 | 2.180328 | 16.094879 |
| H | 9.169875 | 2.632418 | 17.057891 |
| H | 10.556675 | 2.090086 | 16.081237 |
| C | 9.788216 | -0.143484 | 16.454022 |
| H | 10.518765 | -0.371601 | 15.655998 |
| H | 10.337798 | 0.188916 | 17.349900 |
| C | 9.002571 | -1.369840 | 16.820967 |
| H | 8.357015 | -1.165696 | 17.695394 |
| H | 9.712475 | -2.171434 | 17.085829 |
| C | 7.696156 | -3.095466 | 15.900107 |
| H | 8.452652 | -3.818791 | 15.553732 |
| H | 7.542180 | -3.264203 | 16.979924 |
| C | 8.057364 | 3.100700 | 12.722874 |
| H | 7.320258 | 3.696347 | 13.277686 |

| | | | |
|---|---|---|---|
| H | 8.718640 | 3.799066 | 12.172938 |
| N | 8.979121 | -0.238324 | 12.681726 |
| N | 8.812775 | 2.319849 | 13.707884 |
| O | 8.904855 | 0.867335 | 16.009329 |
| O | 8.194937 | -1.770087 | 15.729144 |
| O | 6.669486 | -1.534444 | 12.813178 |
| C | 7.473694 | -3.296330 | 11.421592 |
| H | 6.518653 | -3.823601 | 11.474808 |
| C | 8.531034 | -3.819585 | 10.648924 |
| H | 8.380835 | -4.769828 | 10.131034 |
| C | 9.732888 | -3.158272 | 10.513240 |
| H | 10.534443 | -3.561168 | 9.892023 |
| C | 6.094321 | 2.466937 | 16.481359 |
| C | 6.167973 | 3.736538 | 17.058776 |
| H | 6.275369 | 4.603201 | 16.402737 |
| C | 6.077708 | 3.914875 | 18.454967 |
| H | 6.146246 | 4.928350 | 18.857626 |
| C | 5.890404 | 2.856558 | 19.318665 |
| H | 5.806267 | 3.011509 | 20.395545 |
| Eu | 6.952111 | 0.143012 | 14.422929 |

Eu3+_Ion

SCF (RwB97XD) = -33.8359439519
E(SCF)+ZPE(0 K)= -33.835944
H(298 K)= -33.833583
G(298 K)= -33.853056

| | | | |
|---|---|---|---|
| Eu | 0.887400 | 3.945900 | 9.220400 |

Eu-syn-KHQ_Ligand(2–)

SCF (RwB97XD) = -1910.50396308
E(SCF)+ZPE(0 K)= -1909.835880
H(298 K)= -1909.800017
G(298 K)= -1909.900246
Lowest Frequency = -33.1976cm-1

| | | | |
|---|---|---|---|
| N | 3.092205 | 2.619667 | 9.262318 |
| C | 3.128091 | 1.316262 | 9.427915 |
| C | 4.264132 | 0.682751 | 9.980348 |
| H | 4.264415 | -0.397584 | 10.133136 |
| C | 5.356762 | 1.447100 | 10.320590 |
| H | 6.239786 | 0.977379 | 10.760538 |
| C | 5.365777 | 2.844999 | 10.070577 |
| C | 4.178647 | 3.384901 | 9.524195 |
| C | 4.041940 | 4.781312 | 9.196298 |
| O | 2.885839 | 5.195259 | 8.754525 |
| C | 5.159704 | 5.581628 | 9.415149 |
| H | 5.113015 | 6.640308 | 9.149546 |
| C | 6.343015 | 5.043233 | 9.971097 |
| H | 7.190954 | 5.712630 | 10.134908 |
| C | 6.462489 | 3.712702 | 10.309481 |
| H | 7.386121 | 3.318180 | 10.736092 |
| N | 1.942321 | 4.415436 | 11.548067 |
| C | 2.579999 | 5.516329 | 11.875772 |
| C | 3.560251 | 5.520446 | 12.894604 |

| | | | |
|---|---|---|---|
| H | 4.092491 | 6.442862 | 13.132194 |
| C | 3.832703 | 4.351507 | 13.566510 |
| H | 4.597896 | 4.330655 | 14.346227 |
| C | 3.100733 | 3.169311 | 13.278314 |
| C | 2.143606 | 3.269418 | 12.242902 |
| C | 1.309741 | 2.163669 | 11.853397 |
| O | 0.493369 | 2.343871 | 10.850925 |
| C | 1.471547 | 0.978836 | 12.564699 |
| H | 0.838185 | 0.122826 | 12.320600 |
| C | 2.437434 | 0.875224 | 13.592084 |
| H | 2.536715 | -0.077269 | 14.118206 |
| C | 3.249877 | 1.928957 | 13.950902 |
| H | 3.987115 | 1.828121 | 14.749093 |
| C | 1.905263 | 0.553877 | 8.974736 |
| H | 2.213634 | -0.426371 | 8.559225 |
| H | 1.270493 | 0.359133 | 9.850269 |
| N | 1.125499 | 1.319480 | 7.997408 |
| C | 1.859248 | 1.447804 | 6.737375 |
| H | 1.873679 | 0.492854 | 6.175003 |
| H | 2.899975 | 1.706757 | 6.968745 |
| C | 1.298581 | 2.528131 | 5.841274 |
| H | 1.976286 | 2.655357 | 4.979244 |
| H | 0.302672 | 2.271190 | 5.436398 |
| O | 1.211237 | 3.739108 | 6.563239 |
| C | 0.928632 | 4.848570 | 5.737204 |
| H | -0.087464 | 4.747506 | 5.313358 |
| H | 1.651149 | 4.896984 | 4.903907 |
| C | 1.041433 | 6.104597 | 6.554980 |
| H | 2.071189 | 6.231689 | 6.926223 |
| H | 0.751559 | 6.964929 | 5.929658 |
| O | 0.149830 | 5.983610 | 7.657552 |
| C | -0.207733 | 7.209404 | 8.272669 |
| H | -1.233080 | 7.085015 | 8.634346 |
| H | -0.234186 | 8.009707 | 7.514826 |
| C | 0.759633 | 7.614817 | 9.376209 |
| H | 1.748119 | 7.752734 | 8.916709 |
| H | 0.445482 | 8.601712 | 9.773770 |
| N | 0.911054 | 6.623026 | 10.438323 |
| C | 2.210265 | 6.759935 | 11.105260 |
| H | 2.227633 | 7.634652 | 11.785191 |
| H | 2.975495 | 6.923560 | 10.335637 |
| C | -0.153858 | 6.638322 | 11.441337 |
| H | -0.289916 | 7.649941 | 11.874462 |
| H | 0.158235 | 5.975284 | 12.258023 |
| C | -1.500776 | 6.154319 | 10.941535 |
| H | -2.131979 | 5.904976 | 11.811831 |
| H | -2.034646 | 6.933703 | 10.370777 |
| O | -1.324417 | 5.005331 | 10.134622 |
| C | -2.524133 | 4.489776 | 9.584203 |
| H | -2.668500 | 4.896589 | 8.567107 |
| H | -3.387110 | 4.786211 | 10.201716 |
| C | -2.445765 | 2.989225 | 9.566037 |
| H | -2.284190 | 2.616868 | 10.592860 |
| H | -3.396147 | 2.577948 | 9.181149 |
| O | -1.372247 | 2.577271 | 8.744925 |
| C | -1.220705 | 1.162045 | 8.795294 |
| H | -2.186368 | 0.686156 | 8.550572 |

| | | | |
|---|---|---|---|
| H | -0.926986 | 0.881243 | 9.818426 |
| C | -0.195689 | 0.731663 | 7.777355 |
| H | -0.148269 | -0.374555 | 7.763688 |
| H | -0.559049 | 1.039619 | 6.787953 |

Eu-syn-KHQ(1+)

SCF (RwB97XD) = -1946.14081393
E(SCF)+ZPE(0 K)= -1945.457290
H(298 K)= -1945.420629
G(298 K)= -1945.520270
Lowest Frequency = 39.4672cm-1

| | | | |
|---|---|---|---|
| Eu | 0.935166 | 3.969669 | 9.224383 |
| N | 3.092205 | 2.619667 | 9.262318 |
| C | 3.128091 | 1.316262 | 9.427915 |
| C | 4.264132 | 0.682751 | 9.980348 |
| H | 4.264415 | -0.397584 | 10.133136 |
| C | 5.356762 | 1.447100 | 10.320590 |
| H | 6.239786 | 0.977379 | 10.760538 |
| C | 5.365777 | 2.844999 | 10.070577 |
| C | 4.178647 | 3.384901 | 9.524195 |
| C | 4.041940 | 4.781312 | 9.196298 |
| O | 2.885839 | 5.195259 | 8.754525 |
| C | 5.159704 | 5.581628 | 9.415149 |
| H | 5.113015 | 6.640308 | 9.149546 |
| C | 6.343015 | 5.043233 | 9.971097 |
| H | 7.190954 | 5.712630 | 10.134908 |
| C | 6.462489 | 3.712702 | 10.309481 |
| H | 7.386121 | 3.318180 | 10.736092 |
| N | 1.942321 | 4.415436 | 11.548067 |
| C | 2.579999 | 5.516329 | 11.875772 |
| C | 3.560251 | 5.520446 | 12.894604 |
| H | 4.092491 | 6.442862 | 13.132194 |
| C | 3.832703 | 4.351507 | 13.566510 |
| H | 4.597896 | 4.330655 | 14.346227 |
| C | 3.100733 | 3.169311 | 13.278314 |
| C | 2.143606 | 3.269418 | 12.242902 |
| C | 1.309741 | 2.163669 | 11.853397 |
| O | 0.493369 | 2.343871 | 10.850925 |
| C | 1.471547 | 0.978836 | 12.564699 |
| H | 0.838185 | 0.122826 | 12.320600 |
| C | 2.437434 | 0.875224 | 13.592084 |
| H | 2.536715 | -0.077269 | 14.118206 |
| C | 3.249877 | 1.928957 | 13.950902 |
| H | 3.987115 | 1.828121 | 14.749093 |
| C | 1.905263 | 0.553877 | 8.974736 |
| H | 2.213634 | -0.426371 | 8.559225 |
| H | 1.270493 | 0.359133 | 9.850269 |
| N | 1.125499 | 1.319480 | 7.997408 |
| C | 1.859248 | 1.447804 | 6.737375 |
| H | 1.873679 | 0.492854 | 6.175003 |
| H | 2.899975 | 1.706757 | 6.968745 |
| C | 1.298581 | 2.528131 | 5.841274 |
| H | 1.976286 | 2.655357 | 4.979244 |
| H | 0.302672 | 2.271190 | 5.436398 |
| O | 1.211237 | 3.739108 | 6.563239 |

| | | | |
|---|---|---|---|
| C | 0.928632 | 4.848570 | 5.737204 |
| H | -0.087464 | 4.747506 | 5.313358 |
| H | 1.651149 | 4.896984 | 4.903907 |
| C | 1.041433 | 6.104597 | 6.554980 |
| H | 2.071189 | 6.231689 | 6.926223 |
| H | 0.751559 | 6.964929 | 5.929658 |
| O | 0.149830 | 5.983610 | 7.657552 |
| C | -0.207733 | 7.209404 | 8.272669 |
| H | -1.233080 | 7.085015 | 8.634346 |
| H | -0.234186 | 8.009707 | 7.514826 |
| C | 0.759633 | 7.614817 | 9.376209 |
| H | 1.748119 | 7.752734 | 8.916709 |
| H | 0.445482 | 8.601712 | 9.773770 |
| N | 0.911054 | 6.623026 | 10.438323 |
| C | 2.210265 | 6.759935 | 11.105260 |
| H | 2.227633 | 7.634652 | 11.785191 |
| H | 2.975495 | 6.923560 | 10.335637 |
| C | -0.153858 | 6.638322 | 11.441337 |
| H | -0.289916 | 7.649941 | 11.874462 |
| H | 0.158235 | 5.975284 | 12.258023 |
| C | -1.500776 | 6.154319 | 10.941535 |
| H | -2.131979 | 5.904976 | 11.811831 |
| H | -2.034646 | 6.933703 | 10.370777 |
| O | -1.324417 | 5.005331 | 10.134622 |
| C | -2.524133 | 4.489776 | 9.584203 |
| H | -2.668500 | 4.896589 | 8.567107 |
| H | -3.387110 | 4.786211 | 10.201716 |
| C | -2.445765 | 2.989225 | 9.566037 |
| H | -2.284190 | 2.616868 | 10.592860 |
| H | -3.396147 | 2.577948 | 9.181149 |
| O | -1.372247 | 2.577271 | 8.744925 |
| C | -1.220705 | 1.162045 | 8.795294 |
| H | -2.186368 | 0.686156 | 8.550572 |
| H | -0.926986 | 0.881243 | 9.818426 |
| C | -0.195689 | 0.731663 | 7.777355 |
| H | -0.148269 | -0.374555 | 7.763688 |
| H | -0.559049 | 1.039619 | 6.787953 |

Eu-syn-H2O-KHQ(1+)

SCF (RwB97XD) = -2022.50218869
E(SCF)+ZPE(0 K)= -2021.792981
H(298 K)= -2021.754049
G(298 K)= -2021.857993
Lowest Frequency = 23.7271cm-1

| | | | |
|---|---|---|---|
| Eu | 0.971322 | 4.015376 | 9.083076 |
| N | 3.091833 | 2.609572 | 9.126933 |
| C | 3.104753 | 1.297835 | 9.209622 |
| C | 4.239454 | 0.604991 | 9.690318 |
| H | 4.221928 | -0.483950 | 9.758521 |
| C | 5.349848 | 1.320515 | 10.072072 |
| H | 6.231957 | 0.804137 | 10.458312 |
| C | 5.372104 | 2.734097 | 9.947188 |
| C | 4.191028 | 3.334865 | 9.450302 |
| C | 4.079771 | 4.757360 | 9.251790 |
| O | 2.943258 | 5.231278 | 8.819891 |

| | | | |
|---|---|---|---|
| C | 5.200119 | 5.521351 | 9.564951 |
| H | 5.164192 | 6.601629 | 9.405938 |
| C | 6.374589 | 4.919900 | 10.071080 |
| H | 7.227093 | 5.560905 | 10.308062 |
| C | 6.478405 | 3.560774 | 10.270513 |
| H | 7.394569 | 3.114388 | 10.660540 |
| N | 1.833036 | 4.483575 | 11.501642 |
| C | 2.365505 | 5.613844 | 11.905259 |
| C | 3.197606 | 5.677077 | 13.047986 |
| H | 3.630760 | 6.632086 | 13.350116 |
| C | 3.455242 | 4.527342 | 13.754930 |
| H | 4.106593 | 4.551444 | 14.631898 |
| C | 2.856672 | 3.301291 | 13.364584 |
| C | 2.032591 | 3.347971 | 12.215677 |
| C | 1.336609 | 2.187829 | 11.729671 |
| O | 0.611006 | 2.323339 | 10.657106 |
| C | 1.507828 | 1.004547 | 12.442439 |
| H | 0.979774 | 0.105563 | 12.115261 |
| C | 2.345035 | 0.956890 | 13.580102 |
| H | 2.456421 | 0.005832 | 14.106312 |
| C | 3.016057 | 2.066541 | 14.044961 |
| H | 3.654729 | 2.012204 | 14.928106 |
| C | 1.853036 | 0.571384 | 8.782348 |
| H | 2.128552 | -0.387898 | 8.300026 |
| H | 1.281211 | 0.334578 | 9.687684 |
| N | 1.014278 | 1.378792 | 7.888051 |
| C | 1.689082 | 1.491557 | 6.585412 |
| H | 1.539027 | 0.572642 | 5.985447 |
| H | 2.768050 | 1.587270 | 6.763327 |
| C | 1.261956 | 2.682393 | 5.763133 |
| H | 1.794050 | 2.644198 | 4.796193 |
| H | 0.177261 | 2.676125 | 5.552448 |
| O | 1.596063 | 3.873441 | 6.443972 |
| C | 1.436935 | 5.011883 | 5.625744 |
| H | 0.465493 | 4.955503 | 5.099794 |
| H | 2.236640 | 5.045376 | 4.864465 |
| C | 1.488660 | 6.268779 | 6.453258 |
| H | 2.445034 | 6.333984 | 6.995214 |
| H | 1.369944 | 7.136129 | 5.781245 |
| O | 0.409913 | 6.225738 | 7.380926 |
| C | 0.073883 | 7.454675 | 7.988557 |
| H | -0.994738 | 7.401209 | 8.235905 |
| H | 0.190445 | 8.280986 | 7.266171 |
| C | 0.926294 | 7.744051 | 9.208176 |
| H | 1.969134 | 7.809807 | 8.870204 |
| H | 0.650697 | 8.740625 | 9.609370 |
| N | 0.876685 | 6.715472 | 10.250159 |
| C | 2.089970 | 6.833919 | 11.070355 |
| H | 2.045771 | 7.730694 | 11.719548 |
| H | 2.943175 | 6.958895 | 10.391172 |
| C | -0.305805 | 6.791851 | 11.113575 |
| H | -0.562988 | 7.845272 | 11.344318 |
| H | -0.052789 | 6.308223 | 12.067037 |
| C | -1.561119 | 6.107060 | 10.608305 |
| H | -2.329076 | 6.221298 | 11.393185 |
| H | -1.972403 | 6.573434 | 9.696316 |
| O | -1.326454 | 4.731121 | 10.375367 |

| | | | |
|---|---|---|---|
| C | -2.523445 | 3.970807 | 10.408129 |
| H | -3.188086 | 4.286938 | 9.580248 |
| H | -3.056663 | 4.171690 | 11.353474 |
| C | -2.254801 | 2.489041 | 10.331941 |
| H | -1.558009 | 2.190939 | 11.130091 |
| H | -3.216686 | 1.958548 | 10.456350 |
| O | -1.681989 | 2.187564 | 9.079754 |
| C | -1.243087 | 0.854342 | 8.914417 |
| H | -2.112102 | 0.197467 | 8.729014 |
| H | -0.745990 | 0.527686 | 9.839146 |
| C | -0.309246 | 0.763192 | 7.720830 |
| H | -0.200346 | -0.308926 | 7.459453 |
| H | -0.805448 | 1.241659 | 6.863457 |
| O | -1.004817 | 3.977460 | 7.425322 |
| H | -1.681769 | 3.395607 | 7.815100 |
| H | -1.379759 | 4.806316 | 7.114447 |

Gd-anti-KHQ-Ligand(2–)

SCF (RwB97XD) = -1910.49129446
E(SCF)+ZPE(0 K)= -1909.825221
H(298 K)= -1909.791015
G(298 K)= -1909.887464
Lowest Frequency = -64.7640cm-1

| | | | |
|---|---|---|---|
| C | 5.888399 | 1.358144 | 17.374758 |
| C | 5.754251 | 1.551641 | 18.773181 |
| C | 5.489794 | 0.391081 | 19.547084 |
| H | 5.392926 | 0.480457 | 20.631986 |
| C | 5.315686 | -0.822240 | 18.929243 |
| H | 5.060029 | -1.714579 | 19.504410 |
| C | 5.451452 | -0.908439 | 17.520832 |
| C | 5.085365 | -2.188384 | 16.807787 |
| H | 5.507485 | -3.050017 | 17.350937 |
| H | 3.991830 | -2.304936 | 16.920773 |
| C | 4.282364 | -2.235488 | 14.506612 |
| H | 3.525756 | -2.977124 | 14.831493 |
| H | 4.632779 | -2.509251 | 13.503658 |
| C | 3.621169 | -0.872645 | 14.399205 |
| H | 2.716897 | -0.965347 | 13.776439 |
| H | 3.318632 | -0.474142 | 15.383543 |
| C | 4.048375 | 0.992504 | 12.873032 |
| H | 3.867495 | 1.941552 | 13.403774 |
| H | 3.109889 | 0.646864 | 12.410202 |
| C | 5.096236 | 1.178728 | 11.794148 |
| H | 5.236453 | 0.247480 | 11.220682 |
| H | 4.779565 | 1.988228 | 11.115777 |
| C | 7.269004 | 2.281668 | 11.720451 |
| H | 6.755049 | 2.968997 | 11.028180 |
| H | 7.908224 | 1.609703 | 11.123846 |
| C | 6.385434 | -3.305489 | 15.091712 |
| H | 6.546337 | -3.316056 | 14.009904 |
| H | 5.996015 | -4.301773 | 15.385445 |
| N | 5.784673 | 0.131891 | 16.791439 |
| N | 5.446235 | -2.230030 | 15.394679 |
| O | 6.198443 | 2.222679 | 15.203282 |
| O | 4.529713 | 0.024958 | 13.786571 |

| | | | |
|---|---|---|---|
| O | 6.307104 | 1.530200 | 12.437620 |
| C | 7.597944 | -2.071327 | 12.122009 |
| C | 8.875569 | -1.414294 | 12.046502 |
| C | 9.937311 | -1.950954 | 11.274153 |
| C | 11.111029 | -1.158900 | 11.179431 |
| H | 11.958904 | -1.522490 | 10.593678 |
| C | 11.158302 | 0.070269 | 11.787151 |
| H | 12.034227 | 0.714390 | 11.687025 |
| C | 10.049288 | 0.508992 | 12.552861 |
| C | 10.091647 | 1.862025 | 13.217139 |
| H | 10.545368 | 2.589982 | 12.516027 |
| H | 10.802291 | 1.796477 | 14.055645 |
| C | 8.954057 | 3.074673 | 14.960988 |
| H | 9.674879 | 3.912558 | 14.873482 |
| H | 7.977872 | 3.498390 | 15.204422 |
| C | 9.418341 | 2.176482 | 16.103756 |
| H | 9.090273 | 2.606323 | 17.063670 |
| H | 10.517688 | 2.106443 | 16.124883 |
| C | 9.817223 | -0.163072 | 16.320309 |
| H | 10.490466 | -0.351326 | 15.463541 |
| H | 10.425893 | 0.138977 | 17.187891 |
| C | 9.070053 | -1.412206 | 16.690780 |
| H | 8.479813 | -1.247302 | 17.611347 |
| H | 9.802803 | -2.214875 | 16.879640 |
| C | 7.707036 | -3.109543 | 15.804513 |
| H | 8.449674 | -3.825644 | 15.415524 |
| H | 7.595730 | -3.297385 | 16.886579 |
| C | 8.051092 | 3.114331 | 12.721417 |
| H | 7.314546 | 3.714017 | 13.272497 |
| H | 8.712225 | 3.808971 | 12.166570 |
| N | 8.973690 | -0.228537 | 12.710784 |
| N | 8.805365 | 2.337533 | 13.709400 |
| O | 8.892954 | 0.853406 | 15.982849 |
| O | 8.198216 | -1.781128 | 15.638701 |
| O | 6.647192 | -1.499313 | 12.796308 |
| C | 7.473301 | -3.291393 | 11.456804 |
| H | 6.509654 | -3.804850 | 11.484358 |
| C | 8.549139 | -3.841192 | 10.729571 |
| H | 8.403727 | -4.797934 | 10.222385 |
| C | 9.763885 | -3.197994 | 10.624363 |
| H | 10.580633 | -3.621781 | 10.037802 |
| C | 6.079789 | 2.466491 | 16.473061 |
| C | 6.135343 | 3.738399 | 17.047058 |
| H | 6.236189 | 4.604517 | 16.389278 |
| C | 6.035644 | 3.918845 | 18.442313 |
| H | 6.090207 | 4.933865 | 18.843159 |
| C | 5.855891 | 2.860081 | 19.307103 |
| H | 5.763822 | 3.016285 | 20.383151 |

Gd-anti-KHQ(1+)

SCF (RwB97XD) = -1946.72250111
E(SCF)+ZPE(0 K)= -1946.039988
H(298 K)= -1946.002763
G(298 K)= -1946.105950
Lowest Frequency = 15.9917cm-1

| | | | |
|---|---|---|---|
| C | 5.888399 | 1.358144 | 17.374758 |
| C | 5.754251 | 1.551641 | 18.773181 |
| C | 5.489794 | 0.391081 | 19.547084 |
| H | 5.392926 | 0.480457 | 20.631986 |
| C | 5.315686 | -0.822240 | 18.929243 |
| H | 5.060029 | -1.714579 | 19.504410 |
| C | 5.451452 | -0.908439 | 17.520832 |
| C | 5.085365 | -2.188384 | 16.807787 |
| H | 5.507485 | -3.050017 | 17.350937 |
| H | 3.991830 | -2.304936 | 16.920773 |
| C | 4.282364 | -2.235488 | 14.506612 |
| H | 3.525756 | -2.977124 | 14.831493 |
| H | 4.632779 | -2.509251 | 13.503658 |
| C | 3.621169 | -0.872645 | 14.399205 |
| H | 2.716897 | -0.965347 | 13.776439 |
| H | 3.318632 | -0.474142 | 15.383543 |
| C | 4.048375 | 0.992504 | 12.873032 |
| H | 3.867495 | 1.941552 | 13.403774 |
| H | 3.109889 | 0.646864 | 12.410202 |
| C | 5.096236 | 1.178728 | 11.794148 |
| H | 5.236453 | 0.247480 | 11.220682 |
| H | 4.779565 | 1.988228 | 11.115777 |
| C | 7.269004 | 2.281668 | 11.720451 |
| H | 6.755049 | 2.968997 | 11.028180 |
| H | 7.908224 | 1.609703 | 11.123846 |
| C | 6.385434 | -3.305489 | 15.091712 |
| H | 6.546337 | -3.316056 | 14.009904 |
| H | 5.996015 | -4.301773 | 15.385445 |
| N | 5.784673 | 0.131891 | 16.791439 |
| N | 5.446235 | -2.230030 | 15.394679 |
| O | 6.198443 | 2.222679 | 15.203282 |
| O | 4.529713 | 0.024958 | 13.786571 |
| O | 6.307104 | 1.530200 | 12.437620 |
| C | 7.597944 | -2.071327 | 12.122009 |
| C | 8.875569 | -1.414294 | 12.046502 |
| C | 9.937311 | -1.950954 | 11.274153 |
| C | 11.111029 | -1.158900 | 11.179431 |
| H | 11.958904 | -1.522490 | 10.593678 |
| C | 11.158302 | 0.070269 | 11.787151 |
| H | 12.034227 | 0.714390 | 11.687025 |
| C | 10.049288 | 0.508992 | 12.552861 |
| C | 10.091647 | 1.862025 | 13.217139 |
| H | 10.545368 | 2.589982 | 12.516027 |
| H | 10.802291 | 1.796477 | 14.055645 |
| C | 8.954057 | 3.074673 | 14.960988 |
| H | 9.674879 | 3.912558 | 14.873482 |
| H | 7.977872 | 3.498390 | 15.204422 |
| C | 9.418341 | 2.176482 | 16.103756 |
| H | 9.090273 | 2.606323 | 17.063670 |
| H | 10.517688 | 2.106443 | 16.124883 |
| C | 9.817223 | -0.163072 | 16.320309 |
| H | 10.490466 | -0.351326 | 15.463541 |
| H | 10.425893 | 0.138977 | 17.187891 |
| C | 9.070053 | -1.412206 | 16.690780 |
| H | 8.479813 | -1.247302 | 17.611347 |
| H | 9.802803 | -2.214875 | 16.879640 |
| C | 7.707036 | -3.109543 | 15.804513 |

| | | | |
|---|---|---|---|
| H | 8.449674 | -3.825644 | 15.415524 |
| H | 7.595730 | -3.297385 | 16.886579 |
| C | 8.051092 | 3.114331 | 12.721417 |
| H | 7.314546 | 3.714017 | 13.272497 |
| H | 8.712225 | 3.808971 | 12.166570 |
| N | 8.973690 | -0.228537 | 12.710784 |
| N | 8.805365 | 2.337533 | 13.709400 |
| O | 8.892954 | 0.853406 | 15.982849 |
| O | 8.198216 | -1.781128 | 15.638701 |
| O | 6.647192 | -1.499313 | 12.796308 |
| C | 7.473301 | -3.291393 | 11.456804 |
| H | 6.509654 | -3.804850 | 11.484358 |
| C | 8.549139 | -3.841192 | 10.729571 |
| H | 8.403727 | -4.797934 | 10.222385 |
| C | 9.763885 | -3.197994 | 10.624363 |
| H | 10.580633 | -3.621781 | 10.037802 |
| C | 6.079789 | 2.466491 | 16.473061 |
| C | 6.135343 | 3.738399 | 17.047058 |
| H | 6.236189 | 4.604517 | 16.389278 |
| C | 6.035644 | 3.918845 | 18.442313 |
| H | 6.090207 | 4.933865 | 18.843159 |
| C | 5.855891 | 2.860081 | 19.307103 |
| H | 5.763822 | 3.016285 | 20.383151 |
| Gd | 6.937053 | 0.153056 | 14.414948 |

Gd3+_Ion

SCF (RwB97XD) = -34.4205805528
E(SCF)+ZPE(0 K)= -34.420581
H(298 K)= -34.418220
G(298 K)= -34.437739

| | | | |
|---|---|---|---|
| Gd | 0.887400 | 3.945900 | 9.220400 |

Gd-syn-KHQ_Ligand(2–)

SCF (RwB97XD) = -1910.50238230
E(SCF)+ZPE(0 K)= -1909.834576
H(298 K)= -1909.799504
G(298 K)= -1909.896666
Lowest Frequency = -34.3686cm-1

| | | | |
|---|---|---|---|
| N | 3.084613 | 2.623514 | 9.259552 |
| C | 3.124866 | 1.320136 | 9.425535 |
| C | 4.266497 | 0.689535 | 9.969567 |
| H | 4.269801 | -0.390629 | 10.123476 |
| C | 5.360917 | 1.455959 | 10.299308 |
| H | 6.248406 | 0.988258 | 10.732368 |
| C | 5.365839 | 2.853177 | 10.045736 |
| C | 4.172877 | 3.390071 | 9.509583 |
| C | 4.031184 | 4.784857 | 9.178344 |
| O | 2.869619 | 5.194755 | 8.747408 |
| C | 5.150096 | 5.587310 | 9.382264 |
| H | 5.099751 | 6.644873 | 9.112904 |
| C | 6.339540 | 5.052153 | 9.928398 |
| H | 7.188423 | 5.722996 | 10.080974 |
| C | 6.463826 | 3.723082 | 10.270695 |

| | | | |
|---|---|---|---|
| H | 7.392203 | 3.331171 | 10.689330 |
| N | 1.957159 | 4.408023 | 11.541699 |
| C | 2.600587 | 5.505318 | 11.870509 |
| C | 3.585481 | 5.501993 | 12.884791 |
| H | 4.122849 | 6.421413 | 13.122432 |
| C | 3.855083 | 4.330251 | 13.553063 |
| H | 4.623480 | 4.303906 | 14.329455 |
| C | 3.114824 | 3.152924 | 13.266287 |
| C | 2.154759 | 3.260378 | 12.234613 |
| C | 1.311466 | 2.161514 | 11.847277 |
| O | 0.493952 | 2.350062 | 10.847230 |
| C | 1.465979 | 0.975207 | 12.557429 |
| H | 0.824865 | 0.124299 | 12.315749 |
| C | 2.435131 | 0.863711 | 13.581113 |
| H | 2.528903 | -0.089716 | 14.106538 |
| C | 3.257192 | 1.910841 | 13.937312 |
| H | 3.996503 | 1.803788 | 14.732768 |
| C | 1.902023 | 0.554863 | 8.977828 |
| H | 2.211278 | -0.425100 | 8.562226 |
| H | 1.269788 | 0.359232 | 9.854890 |
| N | 1.119531 | 1.319091 | 8.002464 |
| C | 1.849172 | 1.449660 | 6.740818 |
| H | 1.862292 | 0.496142 | 6.175953 |
| H | 2.890589 | 1.708797 | 6.968734 |
| C | 1.283511 | 2.531724 | 5.850404 |
| H | 1.958345 | 2.664514 | 4.986967 |
| H | 0.287242 | 2.273407 | 5.447330 |
| O | 1.194279 | 3.739976 | 6.576838 |
| C | 0.897642 | 4.847783 | 5.753628 |
| H | -0.123144 | 4.742489 | 5.342177 |
| H | 1.609693 | 4.898077 | 4.911506 |
| C | 1.015903 | 6.104735 | 6.568393 |
| H | 2.049550 | 6.236522 | 6.926535 |
| H | 0.714357 | 6.963020 | 5.945836 |
| O | 0.139102 | 5.981411 | 7.682383 |
| C | -0.213433 | 7.208176 | 8.298841 |
| H | -1.236461 | 7.086231 | 8.667524 |
| H | -0.244127 | 8.007515 | 7.540255 |
| C | 0.762058 | 7.613684 | 9.394927 |
| H | 1.746545 | 7.753930 | 8.927688 |
| H | 0.449374 | 8.599458 | 9.796521 |
| N | 0.923104 | 6.620034 | 10.453122 |
| C | 2.227650 | 6.753383 | 11.109060 |
| H | 2.251702 | 7.624308 | 11.793684 |
| H | 2.986793 | 6.920653 | 10.334312 |
| C | -0.133737 | 6.629679 | 11.464090 |
| H | -0.268808 | 7.639128 | 11.902604 |
| H | 0.185272 | 5.963742 | 12.275656 |
| C | -1.482693 | 6.145380 | 10.970070 |
| H | -2.106894 | 5.883934 | 11.841802 |
| H | -2.023062 | 6.929180 | 10.411664 |
| O | -1.308582 | 5.006316 | 10.148388 |
| C | -2.508939 | 4.506250 | 9.585335 |
| H | -2.639552 | 4.919488 | 8.568949 |
| H | -3.374239 | 4.808608 | 10.196553 |
| C | -2.445150 | 3.005508 | 9.560356 |
| H | -2.297641 | 2.626932 | 10.587021 |

| | | | |
|---|---|---|---|
| H | -3.395234 | 2.604656 | 9.163903 |
| O | -1.367040 | 2.587953 | 8.748359 |
| C | -1.225083 | 1.171728 | 8.801841 |
| H | -2.193807 | 0.702061 | 8.557327 |
| H | -0.934190 | 0.891415 | 9.825820 |
| C | -0.202202 | 0.732165 | 7.786082 |
| H | -0.157300 | -0.374197 | 7.779473 |
| H | -0.565295 | 1.034341 | 6.794844 |

Gd-syn-KHQ(1+)

SCF (RwB97XD) = -1946.73483415
E(SCF)+ZPE(0 K)= -1946.051088
H(298 K)= -1946.014471
G(298 K)= -1946.113965
Lowest Frequency = 40.2146cm-1

| | | | |
|---|---|---|---|
| Gd | 0.938251 | 3.969188 | 9.233516 |
| N | 3.084613 | 2.623514 | 9.259552 |
| C | 3.124866 | 1.320136 | 9.425535 |
| C | 4.266497 | 0.689535 | 9.969567 |
| H | 4.269801 | -0.390629 | 10.123476 |
| C | 5.360917 | 1.455959 | 10.299308 |
| H | 6.248406 | 0.988258 | 10.732368 |
| C | 5.365839 | 2.853177 | 10.045736 |
| C | 4.172877 | 3.390071 | 9.509583 |
| C | 4.031184 | 4.784857 | 9.178344 |
| O | 2.869619 | 5.194755 | 8.747408 |
| C | 5.150096 | 5.587310 | 9.382264 |
| H | 5.099751 | 6.644873 | 9.112904 |
| C | 6.339540 | 5.052153 | 9.928398 |
| H | 7.188423 | 5.722996 | 10.080974 |
| C | 6.463826 | 3.723082 | 10.270695 |
| H | 7.392203 | 3.331171 | 10.689330 |
| N | 1.957159 | 4.408023 | 11.541699 |
| C | 2.600587 | 5.505318 | 11.870509 |
| C | 3.585481 | 5.501993 | 12.884791 |
| H | 4.122849 | 6.421413 | 13.122432 |
| C | 3.855083 | 4.330251 | 13.553063 |
| H | 4.623480 | 4.303906 | 14.329455 |
| C | 3.114824 | 3.152924 | 13.266287 |
| C | 2.154759 | 3.260378 | 12.234613 |
| C | 1.311466 | 2.161514 | 11.847277 |
| O | 0.493952 | 2.350062 | 10.847230 |
| C | 1.465979 | 0.975207 | 12.557429 |
| H | 0.824865 | 0.124299 | 12.315749 |
| C | 2.435131 | 0.863711 | 13.581113 |
| H | 2.528903 | -0.089716 | 14.106538 |
| C | 3.257192 | 1.910841 | 13.937312 |
| H | 3.996503 | 1.803788 | 14.732768 |
| C | 1.902023 | 0.554863 | 8.977828 |
| H | 2.211278 | -0.425100 | 8.562226 |
| H | 1.269788 | 0.359232 | 9.854890 |
| N | 1.119531 | 1.319091 | 8.002464 |
| C | 1.849172 | 1.449660 | 6.740818 |
| H | 1.862292 | 0.496142 | 6.175953 |
| H | 2.890589 | 1.708797 | 6.968734 |

| | | | |
|---|---|---|---|
| C | 1.283511 | 2.531724 | 5.850404 |
| H | 1.958345 | 2.664514 | 4.986967 |
| H | 0.287242 | 2.273407 | 5.447330 |
| O | 1.194279 | 3.739976 | 6.576838 |
| C | 0.897642 | 4.847783 | 5.753628 |
| H | -0.123144 | 4.742489 | 5.342177 |
| H | 1.609693 | 4.898077 | 4.911506 |
| C | 1.015903 | 6.104735 | 6.568393 |
| H | 2.049550 | 6.236522 | 6.926535 |
| H | 0.714357 | 6.963020 | 5.945836 |
| O | 0.139102 | 5.981411 | 7.682383 |
| C | -0.213433 | 7.208176 | 8.298841 |
| H | -1.236461 | 7.086231 | 8.667524 |
| H | -0.244127 | 8.007515 | 7.540255 |
| C | 0.762058 | 7.613684 | 9.394927 |
| H | 1.746545 | 7.753930 | 8.927688 |
| H | 0.449374 | 8.599458 | 9.796521 |
| N | 0.923104 | 6.620034 | 10.453122 |
| C | 2.227650 | 6.753383 | 11.109060 |
| H | 2.251702 | 7.624308 | 11.793684 |
| H | 2.986793 | 6.920653 | 10.334312 |
| C | -0.133737 | 6.629679 | 11.464090 |
| H | -0.268808 | 7.639128 | 11.902604 |
| H | 0.185272 | 5.963742 | 12.275656 |
| C | -1.482693 | 6.145380 | 10.970070 |
| H | -2.106894 | 5.883934 | 11.841802 |
| H | -2.023062 | 6.929180 | 10.411664 |
| O | -1.308582 | 5.006316 | 10.148388 |
| C | -2.508939 | 4.506250 | 9.585335 |
| H | -2.639552 | 4.919488 | 8.568949 |
| H | -3.374239 | 4.808608 | 10.196553 |
| C | -2.445150 | 3.005508 | 9.560356 |
| H | -2.297641 | 2.626932 | 10.587021 |
| H | -3.395234 | 2.604656 | 9.163903 |
| O | -1.367040 | 2.587953 | 8.748359 |
| C | -1.225083 | 1.171728 | 8.801841 |
| H | -2.193807 | 0.702061 | 8.557327 |
| H | -0.934190 | 0.891415 | 9.825820 |
| C | -0.202202 | 0.732165 | 7.786082 |
| H | -0.157300 | -0.374197 | 7.779473 |
| H | -0.565295 | 1.034341 | 6.794844 |

Gd-syn-H2O-KHQ(1+)

SCF (RwB97XD) = -2023.09605905
E(SCF)+ZPE(0 K)= -2022.386826
H(298 K)= -2022.347888
G(298 K)= -2022.452139
Lowest Frequency = 21.7548cm-1

| | | | |
|---|---|---|---|
| Gd | 0.985970 | 4.017722 | 9.086093 |
| N | 3.091677 | 2.614122 | 9.126416 |
| C | 3.105247 | 1.302218 | 9.209346 |
| C | 4.242147 | 0.609848 | 9.685420 |
| H | 4.224848 | -0.479076 | 9.753915 |
| C | 5.354483 | 1.325282 | 10.061679 |
| H | 6.238310 | 0.808938 | 10.444018 |

| | | | |
|---|---|---|---|
| C | 5.376375 | 2.738679 | 9.935367 |
| C | 4.192984 | 3.339008 | 9.443804 |
| C | 4.081095 | 4.760961 | 9.243582 |
| O | 2.942302 | 5.233303 | 8.815738 |
| C | 5.202681 | 5.525560 | 9.550080 |
| H | 5.166099 | 6.605583 | 9.389537 |
| C | 6.379683 | 4.924626 | 10.051137 |
| H | 7.233458 | 5.565845 | 10.282854 |
| C | 6.484407 | 3.565768 | 10.251801 |
| H | 7.402579 | 3.119845 | 10.637599 |
| N | 1.836839 | 4.484281 | 11.493750 |
| C | 2.367596 | 5.613802 | 11.901907 |
| C | 3.190601 | 5.675342 | 13.051212 |
| H | 3.623067 | 6.629562 | 13.356778 |
| C | 3.439467 | 4.525344 | 13.761092 |
| H | 4.083358 | 4.548479 | 14.643578 |
| C | 2.840099 | 3.300877 | 13.367091 |
| C | 2.026687 | 3.349245 | 12.210921 |
| C | 1.328722 | 2.192429 | 11.720747 |
| O | 0.613043 | 2.331850 | 10.642038 |
| C | 1.486642 | 1.009521 | 12.436934 |
| H | 0.956230 | 0.112762 | 12.107451 |
| C | 2.313959 | 0.959549 | 13.581936 |
| H | 2.415448 | 0.008913 | 14.110881 |
| C | 2.987021 | 2.066284 | 14.050732 |
| H | 3.617454 | 2.009916 | 14.939645 |
| C | 1.853231 | 0.574638 | 8.785190 |
| H | 2.129191 | -0.384427 | 8.302612 |
| H | 1.283287 | 0.337004 | 9.691356 |
| N | 1.012796 | 1.381089 | 7.892079 |
| C | 1.686536 | 1.493155 | 6.588913 |
| H | 1.534284 | 0.574906 | 5.988469 |
| H | 2.765968 | 1.586778 | 6.765198 |
| C | 1.260447 | 2.685112 | 5.768324 |
| H | 1.790116 | 2.646218 | 4.800080 |
| H | 0.175252 | 2.680795 | 5.560082 |
| O | 1.598615 | 3.875457 | 6.448435 |
| C | 1.438743 | 5.012486 | 5.628227 |
| H | 0.467682 | 4.953970 | 5.101685 |
| H | 2.238655 | 5.045323 | 4.867161 |
| C | 1.487903 | 6.271271 | 6.452432 |
| H | 2.443690 | 6.339256 | 6.994933 |
| H | 1.368529 | 7.136676 | 5.777979 |
| O | 0.408459 | 6.228911 | 7.379052 |
| C | 0.076458 | 7.458525 | 7.987486 |
| H | -0.991704 | 7.407894 | 8.237319 |
| H | 0.193463 | 8.284689 | 7.265002 |
| C | 0.932527 | 7.744947 | 9.204962 |
| H | 1.974794 | 7.809016 | 8.864924 |
| H | 0.659889 | 8.741531 | 9.608137 |
| N | 0.882756 | 6.715221 | 10.245628 |
| C | 2.095449 | 6.834142 | 11.066599 |
| H | 2.050683 | 7.730864 | 11.715771 |
| H | 2.949365 | 6.958969 | 10.388336 |
| C | -0.300730 | 6.791027 | 11.107881 |
| H | -0.562318 | 7.844557 | 11.332884 |
| H | -0.047033 | 6.313601 | 12.064304 |

| | | | |
|---|---|---|---|
| C | -1.552588 | 6.098450 | 10.604961 |
| H | -2.322918 | 6.217752 | 11.386819 |
| H | -1.962684 | 6.556306 | 9.688029 |
| O | -1.314561 | 4.721311 | 10.384011 |
| C | -2.512993 | 3.962912 | 10.420504 |
| H | -3.180324 | 4.281755 | 9.595775 |
| H | -3.041847 | 4.164993 | 11.368089 |
| C | -2.253113 | 2.479629 | 10.341815 |
| H | -1.548646 | 2.178442 | 11.131815 |
| H | -3.216694 | 1.955463 | 10.479313 |
| O | -1.698886 | 2.173155 | 9.082762 |
| C | -1.245011 | 0.845263 | 8.917834 |
| H | -2.106519 | 0.178726 | 8.732031 |
| H | -0.744938 | 0.523413 | 9.842566 |
| C | -0.309475 | 0.762352 | 7.724634 |
| H | -0.196709 | -0.308630 | 7.460061 |
| H | -0.807489 | 1.241008 | 6.868399 |
| O | -0.982231 | 3.973728 | 7.460512 |
| H | -1.659223 | 3.386938 | 7.845047 |
| H | -1.355402 | 4.800239 | 7.141238 |

H2O

SCF (RwB97XD) = -76.3370013200
E(SCF)+ZPE(0 K)= -76.315287
H(298 K)= -76.311507
G(298 K)= -76.333588
Lowest Frequency = 1645.6741cm-1

| | | | |
|---|---|---|---|
| O | -2.815524 | -1.087365 | 0.000000 |
| H | -1.855311 | -1.036742 | 0.000000 |
| H | -3.088329 | -0.165332 | 0.000000 |

Ho-anti-KHQ-Ligand(2–)

SCF (RwB97XD) = -1910.48146758
E(SCF)+ZPE(0 K)= -1909.815236
H(298 K)= -1909.781069
G(298 K)= -1909.877846
Lowest Frequency = -71.6505cm-1

| | | | |
|---|---|---|---|
| C | 5.790852 | 1.423152 | 17.318744 |
| C | 5.441411 | 1.680062 | 18.668820 |
| C | 5.125269 | 0.547857 | 19.464343 |
| H | 4.846222 | 0.687958 | 20.511573 |
| C | 5.131481 | -0.705168 | 18.905289 |
| H | 4.845390 | -1.583119 | 19.487908 |
| C | 5.510017 | -0.857141 | 17.548155 |
| C | 5.510390 | -2.225621 | 16.915992 |
| H | 6.367902 | -2.781749 | 17.326293 |
| H | 4.616998 | -2.782132 | 17.261792 |
| C | 4.290725 | -2.137230 | 14.796271 |
| H | 3.545369 | -2.785415 | 15.297910 |
| H | 4.428336 | -2.506624 | 13.772014 |
| C | 3.714048 | -0.734948 | 14.677596 |
| H | 2.750429 | -0.804231 | 14.146650 |
| H | 3.531259 | -0.259587 | 15.655270 |

| | | | |
|---|---|---|---|
| C | 4.120523 | 1.062864 | 13.065968 |
| H | 4.021275 | 2.004727 | 13.629883 |
| H | 3.139586 | 0.769238 | 12.657810 |
| C | 5.107614 | 1.224749 | 11.925161 |
| H | 5.154591 | 0.305102 | 11.319390 |
| H | 4.793711 | 2.070417 | 11.291652 |
| C | 7.323725 | 2.255577 | 11.773564 |
| H | 6.797106 | 2.951473 | 11.100091 |
| H | 7.955128 | 1.595892 | 11.155977 |
| C | 6.388185 | -3.333330 | 14.967430 |
| H | 6.293810 | -3.345316 | 13.880037 |
| H | 6.022766 | -4.307839 | 15.349082 |
| N | 5.869347 | 0.164321 | 16.805040 |
| N | 5.595149 | -2.212045 | 15.462101 |
| O | 6.293402 | 2.191095 | 15.153719 |
| O | 4.616000 | 0.048640 | 13.919613 |
| O | 6.378025 | 1.485714 | 12.491278 |
| C | 7.568275 | -2.052001 | 12.128281 |
| C | 8.862730 | -1.427171 | 12.057562 |
| C | 9.909777 | -1.979009 | 11.276522 |
| C | 11.103836 | -1.216052 | 11.194148 |
| H | 11.942534 | -1.592617 | 10.603407 |
| C | 11.182135 | 0.002762 | 11.819446 |
| H | 12.074174 | 0.625822 | 11.728906 |
| C | 10.083265 | 0.459375 | 12.589087 |
| C | 10.150265 | 1.802163 | 13.271280 |
| H | 10.621996 | 2.531128 | 12.583135 |
| H | 10.852841 | 1.713578 | 14.114965 |
| C | 9.019973 | 3.007821 | 15.021359 |
| H | 9.774066 | 3.817704 | 14.956137 |
| H | 8.057713 | 3.467766 | 15.253287 |
| C | 9.428139 | 2.077488 | 16.165283 |
| H | 9.050351 | 2.480399 | 17.118140 |
| H | 10.524950 | 2.010154 | 16.241121 |
| C | 9.855190 | -0.266907 | 16.274841 |
| H | 10.555555 | -0.373145 | 15.426238 |
| H | 10.431940 | -0.021165 | 17.181271 |
| C | 9.129899 | -1.561341 | 16.518058 |
| H | 8.530655 | -1.497624 | 17.444890 |
| H | 9.881258 | -2.359137 | 16.636532 |
| C | 7.862855 | -3.204277 | 15.349162 |
| H | 8.482522 | -3.725704 | 14.602457 |
| H | 8.054896 | -3.670575 | 16.328724 |
| C | 8.122338 | 3.077583 | 12.773382 |
| H | 7.397553 | 3.687911 | 13.327054 |
| H | 8.792059 | 3.761054 | 12.215015 |
| N | 8.990086 | -0.253447 | 12.736440 |
| N | 8.868027 | 2.289776 | 13.759951 |
| O | 8.916892 | 0.753518 | 15.990553 |
| O | 8.278493 | -1.838643 | 15.422841 |
| O | 6.639808 | -1.471317 | 12.823033 |
| C | 7.408396 | -3.253919 | 11.436590 |
| H | 6.429898 | -3.739050 | 11.451013 |
| C | 8.469032 | -3.819355 | 10.698809 |
| H | 8.296500 | -4.761232 | 10.172581 |
| C | 9.702279 | -3.209867 | 10.606116 |
| H | 10.506794 | -3.646204 | 10.011916 |

| | | | |
|---|---|---|---|
| C | 6.024196 | 2.493780 | 16.385848 |
| C | 5.944849 | 3.795001 | 16.884884 |
| H | 6.093006 | 4.629135 | 16.195471 |
| C | 5.641284 | 4.040791 | 18.240095 |
| H | 5.588760 | 5.076219 | 18.584970 |
| C | 5.389382 | 3.018671 | 19.130427 |
| H | 5.131756 | 3.224000 | 20.170708 |

Ho-anti-KHQ(1+)

SCF (RwB97XD) = -1948.47895604
E(SCF)+ZPE(0 K)= -1947.796181
H(298 K)= -1947.758987
G(298 K)= -1947.861504
Lowest Frequency = 19.3098cm-1

| | | | |
|---|---|---|---|
| C | 5.790852 | 1.423152 | 17.318744 |
| C | 5.441411 | 1.680062 | 18.668820 |
| C | 5.125269 | 0.547857 | 19.464343 |
| H | 4.846222 | 0.687958 | 20.511573 |
| C | 5.131481 | -0.705168 | 18.905289 |
| H | 4.845390 | -1.583119 | 19.487908 |
| C | 5.510017 | -0.857141 | 17.548155 |
| C | 5.510390 | -2.225621 | 16.915992 |
| H | 6.367902 | -2.781749 | 17.326293 |
| H | 4.616998 | -2.782132 | 17.261792 |
| C | 4.290725 | -2.137230 | 14.796271 |
| H | 3.545369 | -2.785415 | 15.297910 |
| H | 4.428336 | -2.506624 | 13.772014 |
| C | 3.714048 | -0.734948 | 14.677596 |
| H | 2.750429 | -0.804231 | 14.146650 |
| H | 3.531259 | -0.259587 | 15.655270 |
| C | 4.120523 | 1.062864 | 13.065968 |
| H | 4.021275 | 2.004727 | 13.629883 |
| H | 3.139586 | 0.769238 | 12.657810 |
| C | 5.107614 | 1.224749 | 11.925161 |
| H | 5.154591 | 0.305102 | 11.319390 |
| H | 4.793711 | 2.070417 | 11.291652 |
| C | 7.323725 | 2.255577 | 11.773564 |
| H | 6.797106 | 2.951473 | 11.100091 |
| H | 7.955128 | 1.595892 | 11.155977 |
| C | 6.388185 | -3.333330 | 14.967430 |
| H | 6.293810 | -3.345316 | 13.880037 |
| H | 6.022766 | -4.307839 | 15.349082 |
| N | 5.869347 | 0.164321 | 16.805040 |
| N | 5.595149 | -2.212045 | 15.462101 |
| O | 6.293402 | 2.191095 | 15.153719 |
| O | 4.616000 | 0.048640 | 13.919613 |
| O | 6.378025 | 1.485714 | 12.491278 |
| C | 7.568275 | -2.052001 | 12.128281 |
| C | 8.862730 | -1.427171 | 12.057562 |
| C | 9.909777 | -1.979009 | 11.276522 |
| C | 11.103836 | -1.216052 | 11.194148 |
| H | 11.942534 | -1.592617 | 10.603407 |
| C | 11.182135 | 0.002762 | 11.819446 |
| H | 12.074174 | 0.625822 | 11.728906 |
| C | 10.083265 | 0.459375 | 12.589087 |

| | | | |
|---|---|---|---|
| C | 10.150265 | 1.802163 | 13.271280 |
| H | 10.621996 | 2.531128 | 12.583135 |
| H | 10.852841 | 1.713578 | 14.114965 |
| C | 9.019973 | 3.007821 | 15.021359 |
| H | 9.774066 | 3.817704 | 14.956137 |
| H | 8.057713 | 3.467766 | 15.253287 |
| C | 9.428139 | 2.077488 | 16.165283 |
| H | 9.050351 | 2.480399 | 17.118140 |
| H | 10.524950 | 2.010154 | 16.241121 |
| C | 9.855190 | -0.266907 | 16.274841 |
| H | 10.555555 | -0.373145 | 15.426238 |
| H | 10.431940 | -0.021165 | 17.181271 |
| C | 9.129899 | -1.561341 | 16.518058 |
| H | 8.530655 | -1.497624 | 17.444890 |
| H | 9.881258 | -2.359137 | 16.636532 |
| C | 7.862855 | -3.204277 | 15.349162 |
| H | 8.482522 | -3.725704 | 14.602457 |
| H | 8.054896 | -3.670575 | 16.328724 |
| C | 8.122338 | 3.077583 | 12.773382 |
| H | 7.397553 | 3.687911 | 13.327054 |
| H | 8.792059 | 3.761054 | 12.215015 |
| N | 8.990086 | -0.253447 | 12.736440 |
| N | 8.868027 | 2.289776 | 13.759951 |
| O | 8.916892 | 0.753518 | 15.990553 |
| O | 8.278493 | -1.838643 | 15.422841 |
| O | 6.639808 | -1.471317 | 12.823033 |
| C | 7.408396 | -3.253919 | 11.436590 |
| H | 6.429898 | -3.739050 | 11.451013 |
| C | 8.469032 | -3.819355 | 10.698809 |
| H | 8.296500 | -4.761232 | 10.172581 |
| C | 9.702279 | -3.209867 | 10.606116 |
| H | 10.506794 | -3.646204 | 10.011916 |
| C | 6.024196 | 2.493780 | 16.385848 |
| C | 5.944849 | 3.795001 | 16.884884 |
| H | 6.093006 | 4.629135 | 16.195471 |
| C | 5.641284 | 4.040791 | 18.240095 |
| H | 5.588760 | 5.076219 | 18.584970 |
| C | 5.389382 | 3.018671 | 19.130427 |
| H | 5.131756 | 3.224000 | 20.170708 |
| Ho | 7.003080 | 0.131991 | 14.425158 |

Ho3+_Ion

SCF (RwB97XD) = -36.1463484872
E(SCF)+ZPE(0 K)= -36.146348
H(298 K)= -36.143988
G(298 K)= -36.163568

| | | | |
|---|---|---|---|
| Ho | 0.887400 | 3.945900 | 9.220400 |

Ho-syn-KHQ_Ligand(2–)

SCF (RwB97XD) = -1910.49687183
E(SCF)+ZPE(0 K)= -1909.828594
H(298 K)= -1909.793653
G(298 K)= -1909.890642
Lowest Frequency = -39.4109cm-1

| | | | |
|---|---|---|---|
| N | 3.072879 | 2.635546 | 9.281730 |
| C | 3.127685 | 1.333602 | 9.457449 |
| C | 4.281289 | 0.718359 | 9.993454 |
| H | 4.294980 | -0.360251 | 10.157231 |
| C | 5.375065 | 1.495799 | 10.299081 |
| H | 6.272124 | 1.039180 | 10.724189 |
| C | 5.366629 | 2.889137 | 10.026090 |
| C | 4.161088 | 3.410222 | 9.503958 |
| C | 4.004098 | 4.796968 | 9.152321 |
| O | 2.829903 | 5.188351 | 8.738461 |
| C | 5.120626 | 5.610410 | 9.318702 |
| H | 5.058616 | 6.662457 | 9.030917 |
| C | 6.323662 | 5.091941 | 9.851999 |
| H | 7.171132 | 5.770358 | 9.976162 |
| C | 6.462729 | 3.770118 | 10.215738 |
| H | 7.401473 | 3.391528 | 10.623341 |
| N | 1.974415 | 4.402832 | 11.524216 |
| C | 2.625507 | 5.493840 | 11.859311 |
| C | 3.604911 | 5.479313 | 12.878618 |
| H | 4.150156 | 6.393234 | 13.119453 |
| C | 3.856245 | 4.305570 | 13.551011 |
| H | 4.619055 | 4.271510 | 14.332601 |
| C | 3.099855 | 3.138689 | 13.264165 |
| C | 2.150847 | 3.257047 | 12.224341 |
| C | 1.288372 | 2.174853 | 11.837348 |
| O | 0.485729 | 2.379018 | 10.828147 |
| C | 1.409825 | 0.990729 | 12.556441 |
| H | 0.751656 | 0.152295 | 12.317141 |
| C | 2.369615 | 0.866147 | 13.588177 |
| H | 2.438808 | -0.085181 | 14.121129 |
| C | 3.211533 | 1.897650 | 13.943437 |
| H | 3.941945 | 1.780046 | 14.745593 |
| C | 1.912574 | 0.553767 | 9.015491 |
| H | 2.232397 | -0.428006 | 8.612069 |
| H | 1.275903 | 0.360875 | 9.889578 |
| N | 1.134300 | 1.304386 | 8.028922 |
| C | 1.870172 | 1.433987 | 6.772507 |
| H | 1.891175 | 0.480365 | 6.207948 |
| H | 2.909238 | 1.699102 | 7.003816 |
| C | 1.300239 | 2.512367 | 5.881759 |
| H | 1.979515 | 2.657105 | 5.023715 |
| H | 0.310173 | 2.243753 | 5.470254 |
| O | 1.190225 | 3.717356 | 6.610775 |
| C | 0.859741 | 4.812417 | 5.784199 |
| H | -0.168419 | 4.688266 | 5.396955 |
| H | 1.551526 | 4.864318 | 4.925488 |
| C | 0.977854 | 6.078449 | 6.582538 |
| H | 2.017287 | 6.231925 | 6.912971 |
| H | 0.645853 | 6.924469 | 5.958879 |
| O | 0.131898 | 5.952624 | 7.719293 |
| C | -0.220029 | 7.184655 | 8.326627 |
| H | -1.239810 | 7.066411 | 8.704865 |
| H | -0.258632 | 7.975413 | 7.559846 |
| C | 0.763917 | 7.603370 | 9.409400 |
| H | 1.742214 | 7.746964 | 8.930650 |
| H | 0.448536 | 8.589131 | 9.809224 |

| | | | |
|---|---|---|---|
| N | 0.941618 | 6.614805 | 10.467415 |
| C | 2.251787 | 6.747591 | 11.108378 |
| H | 2.283434 | 7.613286 | 11.799364 |
| H | 3.003241 | 6.921292 | 10.327857 |
| C | -0.106015 | 6.613529 | 11.486183 |
| H | -0.248840 | 7.620434 | 11.928185 |
| H | 0.223950 | 5.949331 | 12.294381 |
| C | -1.451681 | 6.117068 | 10.994798 |
| H | -2.065025 | 5.822733 | 11.863604 |
| H | -2.008123 | 6.906483 | 10.460718 |
| O | -1.267892 | 5.003612 | 10.140020 |
| C | -2.459973 | 4.541276 | 9.529214 |
| H | -2.537705 | 4.964065 | 8.511361 |
| H | -3.339423 | 4.864102 | 10.108398 |
| C | -2.434756 | 3.040711 | 9.494522 |
| H | -2.341913 | 2.652304 | 10.523788 |
| H | -3.376411 | 2.665929 | 9.054714 |
| O | -1.333006 | 2.603432 | 8.727198 |
| C | -1.215598 | 1.185948 | 8.801740 |
| H | -2.188161 | 0.729236 | 8.548435 |
| H | -0.945422 | 0.915520 | 9.833805 |
| C | -0.184579 | 0.715696 | 7.809199 |
| H | -0.144685 | -0.390624 | 7.830168 |
| H | -0.534440 | 0.994319 | 6.806502 |

Ho-syn-KHQ(1+)

SCF (RwB97XD) = -1948.48910366
E(SCF)+ZPE(0 K)= -1947.804956
H(298 K)= -1947.768422
G(298 K)= -1947.867795
Lowest Frequency = 38.7977cm-1

| | | | |
|---|---|---|---|
| Ho | 0.954420 | 3.965434 | 9.248529 |
| N | 3.072879 | 2.635546 | 9.281730 |
| C | 3.127685 | 1.333602 | 9.457449 |
| C | 4.281289 | 0.718359 | 9.993454 |
| H | 4.294980 | -0.360251 | 10.157231 |
| C | 5.375065 | 1.495799 | 10.299081 |
| H | 6.272124 | 1.039180 | 10.724189 |
| C | 5.366629 | 2.889137 | 10.026090 |
| C | 4.161088 | 3.410222 | 9.503958 |
| C | 4.004098 | 4.796968 | 9.152321 |
| O | 2.829903 | 5.188351 | 8.738461 |
| C | 5.120626 | 5.610410 | 9.318702 |
| H | 5.058616 | 6.662457 | 9.030917 |
| C | 6.323662 | 5.091941 | 9.851999 |
| H | 7.171132 | 5.770358 | 9.976162 |
| C | 6.462729 | 3.770118 | 10.215738 |
| H | 7.401473 | 3.391528 | 10.623341 |
| N | 1.974415 | 4.402832 | 11.524216 |
| C | 2.625507 | 5.493840 | 11.859311 |
| C | 3.604911 | 5.479313 | 12.878618 |
| H | 4.150156 | 6.393234 | 13.119453 |
| C | 3.856245 | 4.305570 | 13.551011 |
| H | 4.619055 | 4.271510 | 14.332601 |
| C | 3.099855 | 3.138689 | 13.264165 |

| | | | |
|---|---|---|---|
| C | 2.150847 | 3.257047 | 12.224341 |
| C | 1.288372 | 2.174853 | 11.837348 |
| O | 0.485729 | 2.379018 | 10.828147 |
| C | 1.409825 | 0.990729 | 12.556441 |
| H | 0.751656 | 0.152295 | 12.317141 |
| C | 2.369615 | 0.866147 | 13.588177 |
| H | 2.438808 | -0.085181 | 14.121129 |
| C | 3.211533 | 1.897650 | 13.943437 |
| H | 3.941945 | 1.780046 | 14.745593 |
| C | 1.912574 | 0.553767 | 9.015491 |
| H | 2.232397 | -0.428006 | 8.612069 |
| H | 1.275903 | 0.360875 | 9.889578 |
| N | 1.134300 | 1.304386 | 8.028922 |
| C | 1.870172 | 1.433987 | 6.772507 |
| H | 1.891175 | 0.480365 | 6.207948 |
| H | 2.909238 | 1.699102 | 7.003816 |
| C | 1.300239 | 2.512367 | 5.881759 |
| H | 1.979515 | 2.657105 | 5.023715 |
| H | 0.310173 | 2.243753 | 5.470254 |
| O | 1.190225 | 3.717356 | 6.610775 |
| C | 0.859741 | 4.812417 | 5.784199 |
| H | -0.168419 | 4.688266 | 5.396955 |
| H | 1.551526 | 4.864318 | 4.925488 |
| C | 0.977854 | 6.078449 | 6.582538 |
| H | 2.017287 | 6.231925 | 6.912971 |
| H | 0.645853 | 6.924469 | 5.958879 |
| O | 0.131898 | 5.952624 | 7.719293 |
| C | -0.220029 | 7.184655 | 8.326627 |
| H | -1.239810 | 7.066411 | 8.704865 |
| H | -0.258632 | 7.975413 | 7.559846 |
| C | 0.763917 | 7.603370 | 9.409400 |
| H | 1.742214 | 7.746964 | 8.930650 |
| H | 0.448536 | 8.589131 | 9.809224 |
| N | 0.941618 | 6.614805 | 10.467415 |
| C | 2.251787 | 6.747591 | 11.108378 |
| H | 2.283434 | 7.613286 | 11.799364 |
| H | 3.003241 | 6.921292 | 10.327857 |
| C | -0.106015 | 6.613529 | 11.486183 |
| H | -0.248840 | 7.620434 | 11.928185 |
| H | 0.223950 | 5.949331 | 12.294381 |
| C | -1.451681 | 6.117068 | 10.994798 |
| H | -2.065025 | 5.822733 | 11.863604 |
| H | -2.008123 | 6.906483 | 10.460718 |
| O | -1.267892 | 5.003612 | 10.140020 |
| C | -2.459973 | 4.541276 | 9.529214 |
| H | -2.537705 | 4.964065 | 8.511361 |
| H | -3.339423 | 4.864102 | 10.108398 |
| C | -2.434756 | 3.040711 | 9.494522 |
| H | -2.341913 | 2.652304 | 10.523788 |
| H | -3.376411 | 2.665929 | 9.054714 |
| O | -1.333006 | 2.603432 | 8.727198 |
| C | -1.215598 | 1.185948 | 8.801740 |
| H | -2.188161 | 0.729236 | 8.548435 |
| H | -0.945422 | 0.915520 | 9.833805 |
| C | -0.184579 | 0.715696 | 7.809199 |
| H | -0.144685 | -0.390624 | 7.830168 |
| H | -0.534440 | 0.994319 | 6.806502 |

Ho-syn-H2O-KHQ(1+)

SCF (RwB97XD) = -2024.85000471
E(SCF)+ZPE(0 K)= -2024.140106
H(298 K)= -2024.101389
G(298 K)= -2024.205472
Lowest Frequency = 21.0601cm-1

| | | | |
|---|---|---|---|
| Ho | 1.035358 | 4.022451 | 9.097534 |
| N | 3.096167 | 2.625603 | 9.130364 |
| C | 3.113701 | 1.313173 | 9.212429 |
| C | 4.260483 | 0.623765 | 9.668559 |
| H | 4.246128 | -0.465213 | 9.736681 |
| C | 5.379617 | 1.340314 | 10.022422 |
| H | 6.271307 | 0.824920 | 10.387410 |
| C | 5.397865 | 2.753229 | 9.892151 |
| C | 4.203982 | 3.350588 | 9.424223 |
| C | 4.087072 | 4.770079 | 9.217762 |
| O | 2.937801 | 5.234921 | 8.808352 |
| C | 5.212393 | 5.538655 | 9.496109 |
| H | 5.171110 | 6.617584 | 9.329828 |
| C | 6.400698 | 4.941255 | 9.975285 |
| H | 7.258535 | 5.584483 | 10.185248 |
| C | 6.511390 | 3.583578 | 10.180368 |
| H | 7.438482 | 3.140809 | 10.548015 |
| N | 1.846669 | 4.488996 | 11.474510 |
| C | 2.370089 | 5.617188 | 11.896257 |
| C | 3.158295 | 5.676142 | 13.069528 |
| H | 3.587015 | 6.628542 | 13.385792 |
| C | 3.374889 | 4.527126 | 13.792390 |
| H | 3.990161 | 4.549494 | 14.695089 |
| C | 2.774392 | 3.307003 | 13.387080 |
| C | 2.001529 | 3.357765 | 12.204310 |
| C | 1.298093 | 2.211431 | 11.700688 |
| O | 0.618701 | 2.361543 | 10.599936 |
| C | 1.409413 | 1.031709 | 12.429743 |
| H | 0.872479 | 0.141808 | 12.092324 |
| C | 2.199226 | 0.976944 | 13.601537 |
| H | 2.265825 | 0.029204 | 14.141052 |
| C | 2.876992 | 2.074897 | 14.083831 |
| H | 3.476290 | 2.014013 | 14.993715 |
| C | 1.860563 | 0.581961 | 8.798879 |
| H | 2.137689 | -0.378367 | 8.319230 |
| H | 1.294673 | 0.344964 | 9.707376 |
| N | 1.017632 | 1.383998 | 7.905926 |
| C | 1.690029 | 1.493860 | 6.602556 |
| H | 1.532874 | 0.577268 | 6.000765 |
| H | 2.770377 | 1.582264 | 6.775733 |
| C | 1.268132 | 2.688554 | 5.785444 |
| H | 1.790370 | 2.645749 | 4.813301 |
| H | 0.181487 | 2.691331 | 5.585406 |
| O | 1.618983 | 3.877534 | 6.461470 |
| C | 1.450787 | 5.009456 | 5.636081 |
| H | 0.479912 | 4.940921 | 5.110317 |
| H | 2.249575 | 5.044440 | 4.873864 |
| C | 1.487128 | 6.273860 | 6.450620 |

| | | | |
|---|---|---|---|
| H | 2.441771 | 6.356132 | 6.992512 |
| H | 1.360220 | 7.132564 | 5.768863 |
| O | 0.407186 | 6.228990 | 7.375809 |
| C | 0.080389 | 7.459756 | 7.983812 |
| H | -0.986117 | 7.412154 | 8.240558 |
| H | 0.194737 | 8.284788 | 7.259577 |
| C | 0.945112 | 7.744106 | 9.195106 |
| H | 1.985623 | 7.805626 | 8.849372 |
| H | 0.677448 | 8.740663 | 9.601626 |
| N | 0.898174 | 6.712843 | 10.233705 |
| C | 2.109713 | 6.835854 | 11.055733 |
| H | 2.064234 | 7.734598 | 11.701847 |
| H | 2.965240 | 6.957230 | 10.378926 |
| C | -0.287207 | 6.785417 | 11.093957 |
| H | -0.558860 | 7.838587 | 11.307919 |
| H | -0.031767 | 6.320422 | 12.055953 |
| C | -1.531281 | 6.075897 | 10.596282 |
| H | -2.306838 | 6.206131 | 11.371428 |
| H | -1.938382 | 6.515952 | 9.669266 |
| O | -1.286156 | 4.697065 | 10.400642 |
| C | -2.488471 | 3.943923 | 10.439664 |
| H | -3.155372 | 4.265879 | 9.615664 |
| H | -3.014362 | 4.152919 | 11.387576 |
| C | -2.250766 | 2.456542 | 10.362416 |
| H | -1.529292 | 2.151293 | 11.134716 |
| H | -3.217949 | 1.949370 | 10.534389 |
| O | -1.743726 | 2.130840 | 9.089048 |
| C | -1.241788 | 0.820074 | 8.930707 |
| H | -2.079100 | 0.122929 | 8.747395 |
| H | -0.731322 | 0.518993 | 9.856675 |
| C | -0.302264 | 0.759303 | 7.738010 |
| H | -0.181131 | -0.308229 | 7.462724 |
| H | -0.805343 | 1.241620 | 6.886785 |
| O | -0.904515 | 3.941546 | 7.543960 |
| H | -1.581674 | 3.348467 | 7.926078 |
| H | -1.273218 | 4.771156 | 7.226547 |

KHQ-Ligand(2–)

SCF (RwB97XD) = -1910.64998644
E(SCF)+ZPE(0 K)= -1909.977650
H(298 K)= -1909.940357
G(298 K)= -1910.045908
Lowest Frequency = 15.3711cm-1

| | | | |
|---|---|---|---|
| N | 3.968043 | 4.472791 | 8.323716 |
| C | 3.696370 | 3.190266 | 8.433335 |
| C | 4.631210 | 2.204829 | 8.023147 |
| H | 4.360679 | 1.151660 | 8.125221 |
| C | 5.836427 | 2.608185 | 7.505214 |
| H | 6.580029 | 1.869277 | 7.185557 |
| C | 6.143236 | 3.990758 | 7.366170 |
| C | 5.137202 | 4.905508 | 7.801905 |
| C | 5.316315 | 6.375879 | 7.669172 |
| O | 4.432144 | 7.187689 | 8.014756 |
| C | 6.577763 | 6.760570 | 7.106534 |
| H | 6.754290 | 7.835171 | 6.996420 |

| | | | |
|---|---|---|---|
| C | 7.545502 | 5.835822 | 6.705212 |
| H | 8.485667 | 6.213418 | 6.282002 |
| C | 7.363905 | 4.464706 | 6.816253 |
| H | 8.128398 | 3.753198 | 6.490658 |
| N | 1.686954 | 2.875098 | 12.420157 |
| C | 1.561549 | 4.158214 | 12.688270 |
| C | 0.980204 | 4.598723 | 13.906673 |
| H | 0.926428 | 5.669473 | 14.124254 |
| C | 0.490369 | 3.674561 | 14.794518 |
| H | 0.026127 | 3.993118 | 15.734278 |
| C | 0.596167 | 2.287151 | 14.514997 |
| C | 1.248963 | 1.938181 | 13.286983 |
| C | 1.489982 | 0.509906 | 12.936334 |
| O | 2.111935 | 0.171553 | 11.913356 |
| C | 0.947443 | -0.429242 | 13.878632 |
| H | 1.086877 | -1.487749 | 13.639433 |
| C | 0.295865 | -0.043200 | 15.048238 |
| H | -0.080724 | -0.824592 | 15.721157 |
| C | 0.112361 | 1.288937 | 15.397224 |
| H | -0.388203 | 1.578695 | 16.325610 |
| C | 2.359766 | 2.796885 | 9.025379 |
| H | 2.512146 | 2.448715 | 10.062933 |
| H | 1.737277 | 3.706912 | 9.101523 |
| N | 1.719483 | 1.707402 | 8.304955 |
| C | 1.352026 | 1.938389 | 6.933386 |
| H | 1.225839 | 0.957694 | 6.430787 |
| H | 2.207916 | 2.426334 | 6.431999 |
| C | 0.075178 | 2.734736 | 6.639820 |
| H | -0.123529 | 2.706208 | 5.544781 |
| H | -0.765079 | 2.249522 | 7.154616 |
| O | 0.108267 | 4.061895 | 7.099861 |
| C | 0.751208 | 4.983552 | 6.262372 |
| H | 0.004222 | 5.454014 | 5.586570 |
| H | 1.507226 | 4.487386 | 5.624370 |
| C | 1.477269 | 6.034611 | 7.062835 |
| H | 2.253460 | 5.554532 | 7.678937 |
| H | 2.011085 | 6.716703 | 6.370380 |
| O | 0.558772 | 6.753489 | 7.847946 |
| C | 1.146209 | 7.507464 | 8.874392 |
| H | 0.320909 | 8.100618 | 9.305644 |
| H | 1.890083 | 8.212571 | 8.455048 |
| C | 1.898559 | 6.748751 | 9.965527 |
| H | 2.808808 | 6.312893 | 9.523511 |
| H | 2.257451 | 7.553993 | 10.657673 |
| N | 1.191024 | 5.724019 | 10.718199 |
| C | 2.112949 | 5.174803 | 11.710604 |
| H | 2.557616 | 6.003770 | 12.314461 |
| H | 2.943846 | 4.711621 | 11.158482 |
| C | -0.121941 | 6.106395 | 11.185526 |
| H | -0.188335 | 7.197469 | 11.403545 |
| H | -0.350129 | 5.589309 | 12.127032 |
| C | -1.230004 | 5.714191 | 10.219102 |
| H | -2.141669 | 6.320402 | 10.430125 |
| H | -0.914686 | 5.904040 | 9.179963 |
| O | -1.519665 | 4.352418 | 10.399186 |
| C | -2.345231 | 3.821331 | 9.409208 |
| H | -1.931164 | 4.029717 | 8.404045 |

| | | | |
|---|---|---|---|
| H | -3.358259 | 4.286723 | 9.463076 |
| C | -2.497622 | 2.317775 | 9.588917 |
| H | -2.652215 | 2.107044 | 10.667071 |
| H | -3.409629 | 2.003125 | 9.051229 |
| O | -1.453030 | 1.555322 | 9.069749 |
| C | -0.281021 | 1.494708 | 9.878408 |
| H | -0.500245 | 0.882594 | 10.774263 |
| H | 0.003009 | 2.497432 | 10.234444 |
| C | 0.883354 | 0.853517 | 9.127896 |
| H | 1.517146 | 0.416365 | 9.916422 |
| H | 0.489572 | 0.028488 | 8.506217 |

La-anti-KHQ-Ligand(2–)

SCF (RwB97XD) = -1910.51144960
E(SCF)+ZPE(0 K)= -1909.844632
H(298 K)= -1909.809848
G(298 K)= -1909.907958
Lowest Frequency = -46.2280cm-1

| | | | |
|---|---|---|---|
| C | 5.836825 | 1.398865 | 17.423985 |
| C | 5.666446 | 1.621093 | 18.816763 |
| C | 5.259119 | 0.501505 | 19.587498 |
| H | 5.126469 | 0.614285 | 20.666474 |
| C | 5.001730 | -0.702571 | 18.976205 |
| H | 4.647465 | -1.560279 | 19.551628 |
| C | 5.188634 | -0.819147 | 17.577666 |
| C | 4.754492 | -2.062646 | 16.839308 |
| H | 4.831322 | -2.948285 | 17.495730 |
| H | 3.673469 | -1.943457 | 16.657861 |
| C | 4.503028 | -2.520915 | 14.464255 |
| H | 3.750538 | -3.291211 | 14.726014 |
| H | 5.089895 | -2.877983 | 13.608629 |
| C | 3.777487 | -1.260849 | 14.030172 |
| H | 3.048600 | -1.530961 | 13.247898 |
| H | 3.222800 | -0.785145 | 14.858906 |
| C | 4.247290 | 0.610572 | 12.566520 |
| H | 3.907415 | 1.518599 | 13.092808 |
| H | 3.406385 | 0.194471 | 11.987331 |
| C | 5.383449 | 0.937845 | 11.623155 |
| H | 5.679194 | 0.048284 | 11.039735 |
| H | 5.059723 | 1.730090 | 10.927918 |
| C | 7.371619 | 2.303717 | 11.801065 |
| H | 6.797148 | 3.055262 | 11.233392 |
| H | 8.032959 | 1.778570 | 11.091794 |
| C | 6.453888 | -3.331470 | 15.663423 |
| H | 6.911980 | -3.436679 | 14.671098 |
| H | 6.013164 | -4.307063 | 15.954477 |
| N | 5.636089 | 0.182289 | 16.852710 |
| N | 5.437303 | -2.282888 | 15.566832 |
| O | 6.331621 | 2.209218 | 15.259909 |
| O | 4.717101 | -0.341099 | 13.500844 |
| O | 6.474602 | 1.387585 | 12.402776 |
| C | 7.791653 | -2.249362 | 12.052660 |
| C | 8.859403 | -1.357598 | 11.678600 |
| C | 9.623125 | -1.580837 | 10.500898 |
| C | 10.624477 | -0.622553 | 10.204652 |

| | | | |
|---|---|---|---|
| H | 11.238014 | -0.746344 | 9.308863 |
| C | 10.804539 | 0.459780 | 11.030596 |
| H | 11.556777 | 1.218716 | 10.807927 |
| C | 9.994389 | 0.589665 | 12.183404 |
| C | 10.183337 | 1.752258 | 13.122898 |
| H | 10.675183 | 2.591371 | 12.592807 |
| H | 10.894275 | 1.426503 | 13.897061 |
| C | 9.231872 | 2.906345 | 15.015783 |
| H | 9.924749 | 3.757059 | 14.855604 |
| H | 8.282526 | 3.311244 | 15.377864 |
| C | 9.828813 | 2.025115 | 16.092361 |
| H | 9.805305 | 2.573723 | 17.049504 |
| H | 10.883386 | 1.771709 | 15.882552 |
| C | 9.743215 | -0.120058 | 17.058715 |
| H | 10.582098 | -0.583645 | 16.508487 |
| H | 10.148728 | 0.383281 | 17.952620 |
| C | 8.759411 | -1.163814 | 17.508201 |
| H | 7.988344 | -0.718448 | 18.163784 |
| H | 9.306142 | -1.930560 | 18.082516 |
| C | 7.519510 | -2.993507 | 16.681734 |
| H | 8.284978 | -3.787430 | 16.691669 |
| H | 7.090716 | -2.931136 | 17.697319 |
| C | 8.124078 | 3.035923 | 12.896471 |
| H | 7.367949 | 3.508193 | 13.537489 |
| H | 8.735547 | 3.831712 | 12.426021 |
| N | 9.072436 | -0.293853 | 12.500260 |
| N | 8.950114 | 2.191097 | 13.769443 |
| O | 9.092389 | 0.816653 | 16.222358 |
| O | 8.137584 | -1.746179 | 16.377686 |
| O | 7.112888 | -1.981765 | 13.127033 |
| C | 7.555294 | -3.342545 | 11.220929 |
| H | 6.750981 | -4.032606 | 11.484339 |
| C | 8.328347 | -3.563057 | 10.062597 |
| H | 8.106666 | -4.435191 | 9.442916 |
| C | 9.344975 | -2.710075 | 9.691253 |
| H | 9.931870 | -2.886313 | 8.788334 |
| C | 6.186276 | 2.472695 | 16.521228 |
| C | 6.356577 | 3.735182 | 17.095380 |
| H | 6.579192 | 4.580137 | 16.439798 |
| C | 6.214852 | 3.939743 | 18.483573 |
| H | 6.361428 | 4.947420 | 18.880106 |
| C | 5.883779 | 2.917042 | 19.346804 |
| H | 5.763166 | 3.094084 | 20.416774 |

La-anti-KHQ(1+)

SCF (RwB97XD) = -1942.41643622
E(SCF)+ZPE(0 K)= -1941.734227
H(298 K)= -1941.696907
G(298 K)= -1941.800071
Lowest Frequency = 16.5511cm-1

| | | | |
|---|---|---|---|
| C | 5.836825 | 1.398865 | 17.423985 |
| C | 5.666446 | 1.621093 | 18.816763 |
| C | 5.259119 | 0.501505 | 19.587498 |
| H | 5.126469 | 0.614285 | 20.666474 |
| C | 5.001730 | -0.702571 | 18.976205 |

| | | | |
|---|---|---|---|
| H | 4.647465 | -1.560279 | 19.551628 |
| C | 5.188634 | -0.819147 | 17.577666 |
| C | 4.754492 | -2.062646 | 16.839308 |
| H | 4.831322 | -2.948285 | 17.495730 |
| H | 3.673469 | -1.943457 | 16.657861 |
| C | 4.503028 | -2.520915 | 14.464255 |
| H | 3.750538 | -3.291211 | 14.726014 |
| H | 5.089895 | -2.877983 | 13.608629 |
| C | 3.777487 | -1.260849 | 14.030172 |
| H | 3.048600 | -1.530961 | 13.247898 |
| H | 3.222800 | -0.785145 | 14.858906 |
| C | 4.247290 | 0.610572 | 12.566520 |
| H | 3.907415 | 1.518599 | 13.092808 |
| H | 3.406385 | 0.194471 | 11.987331 |
| C | 5.383449 | 0.937845 | 11.623155 |
| H | 5.679194 | 0.048284 | 11.039735 |
| H | 5.059723 | 1.730090 | 10.927918 |
| C | 7.371619 | 2.303717 | 11.801065 |
| H | 6.797148 | 3.055262 | 11.233392 |
| H | 8.032959 | 1.778570 | 11.091794 |
| C | 6.453888 | -3.331470 | 15.663423 |
| H | 6.911980 | -3.436679 | 14.671098 |
| H | 6.013164 | -4.307063 | 15.954477 |
| N | 5.636089 | 0.182289 | 16.852710 |
| N | 5.437303 | -2.282888 | 15.566832 |
| O | 6.331621 | 2.209218 | 15.259909 |
| O | 4.717101 | -0.341099 | 13.500844 |
| O | 6.474602 | 1.387585 | 12.402776 |
| C | 7.791653 | -2.249362 | 12.052660 |
| C | 8.859403 | -1.357598 | 11.678600 |
| C | 9.623125 | -1.580837 | 10.500898 |
| C | 10.624477 | -0.622553 | 10.204652 |
| H | 11.238014 | -0.746344 | 9.308863 |
| C | 10.804539 | 0.459780 | 11.030596 |
| H | 11.556777 | 1.218716 | 10.807927 |
| C | 9.994389 | 0.589665 | 12.183404 |
| C | 10.183337 | 1.752258 | 13.122898 |
| H | 10.675183 | 2.591371 | 12.592807 |
| H | 10.894275 | 1.426503 | 13.897061 |
| C | 9.231872 | 2.906345 | 15.015783 |
| H | 9.924749 | 3.757059 | 14.855604 |
| H | 8.282526 | 3.311244 | 15.377864 |
| C | 9.828813 | 2.025115 | 16.092361 |
| H | 9.805305 | 2.573723 | 17.049504 |
| H | 10.883386 | 1.771709 | 15.882552 |
| C | 9.743215 | -0.120058 | 17.058715 |
| H | 10.582098 | -0.583645 | 16.508487 |
| H | 10.148728 | 0.383281 | 17.952620 |
| C | 8.759411 | -1.163814 | 17.508201 |
| H | 7.988344 | -0.718448 | 18.163784 |
| H | 9.306142 | -1.930560 | 18.082516 |
| C | 7.519510 | -2.993507 | 16.681734 |
| H | 8.284978 | -3.787430 | 16.691669 |
| H | 7.090716 | -2.931136 | 17.697319 |
| C | 8.124078 | 3.035923 | 12.896471 |
| H | 7.367949 | 3.508193 | 13.537489 |
| H | 8.735547 | 3.831712 | 12.426021 |

| | | | |
|---|---|---|---|
| N | 9.072436 | -0.293853 | 12.500260 |
| N | 8.950114 | 2.191097 | 13.769443 |
| O | 9.092389 | 0.816653 | 16.222358 |
| O | 8.137584 | -1.746179 | 16.377686 |
| O | 7.112888 | -1.981765 | 13.127033 |
| C | 7.555294 | -3.342545 | 11.220929 |
| H | 6.750981 | -4.032606 | 11.484339 |
| C | 8.328347 | -3.563057 | 10.062597 |
| H | 8.106666 | -4.435191 | 9.442916 |
| C | 9.344975 | -2.710075 | 9.691253 |
| H | 9.931870 | -2.886313 | 8.788334 |
| C | 6.186276 | 2.472695 | 16.521228 |
| C | 6.356577 | 3.735182 | 17.095380 |
| H | 6.579192 | 4.580137 | 16.439798 |
| C | 6.214852 | 3.939743 | 18.483573 |
| H | 6.361428 | 4.947420 | 18.880106 |
| C | 5.883779 | 2.917042 | 19.346804 |
| H | 5.763166 | 3.094084 | 20.416774 |
| La | 7.121697 | -0.006201 | 14.534860 |

La3+_Ion

SCF (RwB97XD) = -30.1902022435
E(SCF)+ZPE(0 K)= -30.190202
H(298 K)= -30.187842
G(298 K)= -30.207179

| | | | |
|---|---|---|---|
| La | 0.887400 | 3.945900 | 9.220400 |

La-syn-KHQ_Ligand(2–)

SCF (RwB97XD) = -1910.51561708
E(SCF)+ZPE(0 K)= -1909.848424
H(298 K)= -1909.813132
G(298 K)= -1909.911373
Lowest Frequency = -35.9135cm-1

| | | | |
|---|---|---|---|
| N | 3.127379 | 2.586003 | 9.199107 |
| C | 3.132573 | 1.279614 | 9.337873 |
| C | 4.243853 | 0.610559 | 9.899333 |
| H | 4.223548 | -0.473323 | 10.023410 |
| C | 5.336015 | 1.348651 | 10.293270 |
| H | 6.199164 | 0.853185 | 10.744285 |
| C | 5.369697 | 2.754957 | 10.097086 |
| C | 4.210825 | 3.332328 | 9.525109 |
| C | 4.105915 | 4.746926 | 9.253660 |
| O | 2.982540 | 5.207520 | 8.775838 |
| C | 5.223234 | 5.519228 | 9.563711 |
| H | 5.198280 | 6.590055 | 9.348512 |
| C | 6.375091 | 4.941420 | 10.142939 |
| H | 7.221749 | 5.591775 | 10.375818 |
| C | 6.465708 | 3.594573 | 10.420605 |
| H | 7.365049 | 3.167743 | 10.867530 |
| N | 1.900300 | 4.441154 | 11.593638 |
| C | 2.508551 | 5.561903 | 11.908789 |
| C | 3.486645 | 5.604591 | 12.929329 |
| H | 3.989123 | 6.544650 | 13.162770 |

| | | | |
|---|---|---|---|
| C | 3.800960 | 4.447128 | 13.601317 |
| H | 4.567311 | 4.454061 | 14.380140 |
| C | 3.121086 | 3.235329 | 13.308027 |
| C | 2.151151 | 3.297259 | 12.279305 |
| C | 1.374821 | 2.147382 | 11.886541 |
| O | 0.532108 | 2.278338 | 10.898917 |
| C | 1.615926 | 0.965802 | 12.582942 |
| H | 1.032564 | 0.077569 | 12.329479 |
| C | 2.591818 | 0.904852 | 13.602372 |
| H | 2.750190 | -0.046671 | 14.115694 |
| C | 3.343397 | 2.000428 | 13.968651 |
| H | 4.091623 | 1.934528 | 14.760243 |
| C | 1.890502 | 0.547294 | 8.882001 |
| H | 2.174013 | -0.430490 | 8.443914 |
| H | 1.272379 | 0.350209 | 9.769645 |
| N | 1.092539 | 1.335352 | 7.932823 |
| C | 1.802064 | 1.470371 | 6.656652 |
| H | 1.799624 | 0.516840 | 6.091744 |
| H | 2.848381 | 1.720274 | 6.873650 |
| C | 1.242807 | 2.557773 | 5.764345 |
| H | 1.901738 | 2.655763 | 4.884186 |
| H | 0.230202 | 2.323551 | 5.388095 |
| O | 1.208673 | 3.777728 | 6.476205 |
| C | 0.992819 | 4.909755 | 5.658957 |
| H | -0.009493 | 4.850967 | 5.196568 |
| H | 1.748038 | 4.947264 | 4.854556 |
| C | 1.116458 | 6.150626 | 6.503479 |
| H | 2.131736 | 6.224562 | 6.928746 |
| H | 0.902096 | 7.032510 | 5.877235 |
| O | 0.161691 | 6.057588 | 7.555467 |
| C | -0.177891 | 7.278432 | 8.188768 |
| H | -1.213917 | 7.166513 | 8.525008 |
| H | -0.170226 | 8.096126 | 7.448476 |
| C | 0.770641 | 7.644145 | 9.323460 |
| H | 1.775961 | 7.752250 | 8.892687 |
| H | 0.477558 | 8.639646 | 9.715201 |
| N | 0.866324 | 6.650183 | 10.394784 |
| C | 2.148123 | 6.786415 | 11.102161 |
| H | 2.151572 | 7.678966 | 11.758540 |
| H | 2.934703 | 6.923223 | 10.347959 |
| C | -0.227897 | 6.697452 | 11.368327 |
| H | -0.360537 | 7.720779 | 11.774103 |
| H | 0.060243 | 6.050482 | 12.207072 |
| C | -1.575932 | 6.215756 | 10.865561 |
| H | -2.230959 | 6.058361 | 11.740085 |
| H | -2.076880 | 6.958789 | 10.221667 |
| O | -1.415142 | 4.997679 | 10.165555 |
| C | -2.622426 | 4.383182 | 9.748631 |
| H | -2.904330 | 4.754632 | 8.746992 |
| H | -3.435619 | 4.631028 | 10.450530 |
| C | -2.441724 | 2.888365 | 9.746861 |
| H | -2.132436 | 2.551311 | 10.751983 |
| H | -3.402700 | 2.410250 | 9.485761 |
| O | -1.449018 | 2.520800 | 8.807936 |
| C | -1.232663 | 1.113035 | 8.812361 |
| H | -2.186587 | 0.599441 | 8.599426 |
| H | -0.878534 | 0.819267 | 9.812981 |

| | | | |
|---|---|---|---|
| C | -0.237398 | 0.752252 | 7.735643 |
| H | -0.182171 | -0.351375 | 7.661354 |
| H | -0.639809 | 1.109650 | 6.778033 |

La-syn-KHQ(1+)

SCF (RwB97XD) = -1942.42891623
E(SCF)+ZPE(0 K)= -1941.746307
H(298 K)= -1941.709416
G(298 K)= -1941.809708
Lowest Frequency = 35.0894cm-1

| | | | |
|---|---|---|---|
| La | 0.900306 | 3.977809 | 9.185785 |
| N | 3.127379 | 2.586003 | 9.199107 |
| C | 3.132573 | 1.279614 | 9.337873 |
| C | 4.243853 | 0.610559 | 9.899333 |
| H | 4.223548 | -0.473323 | 10.023410 |
| C | 5.336015 | 1.348651 | 10.293270 |
| H | 6.199164 | 0.853185 | 10.744285 |
| C | 5.369697 | 2.754957 | 10.097086 |
| C | 4.210825 | 3.332328 | 9.525109 |
| C | 4.105915 | 4.746926 | 9.253660 |
| O | 2.982540 | 5.207520 | 8.775838 |
| C | 5.223234 | 5.519228 | 9.563711 |
| H | 5.198280 | 6.590055 | 9.348512 |
| C | 6.375091 | 4.941420 | 10.142939 |
| H | 7.221749 | 5.591775 | 10.375818 |
| C | 6.465708 | 3.594573 | 10.420605 |
| H | 7.365049 | 3.167743 | 10.867530 |
| N | 1.900300 | 4.441154 | 11.593638 |
| C | 2.508551 | 5.561903 | 11.908789 |
| C | 3.486645 | 5.604591 | 12.929329 |
| H | 3.989123 | 6.544650 | 13.162770 |
| C | 3.800960 | 4.447128 | 13.601317 |
| H | 4.567311 | 4.454061 | 14.380140 |
| C | 3.121086 | 3.235329 | 13.308027 |
| C | 2.151151 | 3.297259 | 12.279305 |
| C | 1.374821 | 2.147382 | 11.886541 |
| O | 0.532108 | 2.278338 | 10.898917 |
| C | 1.615926 | 0.965802 | 12.582942 |
| H | 1.032564 | 0.077569 | 12.329479 |
| C | 2.591818 | 0.904852 | 13.602372 |
| H | 2.750190 | -0.046671 | 14.115694 |
| C | 3.343397 | 2.000428 | 13.968651 |
| H | 4.091623 | 1.934528 | 14.760243 |
| C | 1.890502 | 0.547294 | 8.882001 |
| H | 2.174013 | -0.430490 | 8.443914 |
| H | 1.272379 | 0.350209 | 9.769645 |
| N | 1.092539 | 1.335352 | 7.932823 |
| C | 1.802064 | 1.470371 | 6.656652 |
| H | 1.799624 | 0.516840 | 6.091744 |
| H | 2.848381 | 1.720274 | 6.873650 |
| C | 1.242807 | 2.557773 | 5.764345 |
| H | 1.901738 | 2.655763 | 4.884186 |
| H | 0.230202 | 2.323551 | 5.388095 |
| O | 1.208673 | 3.777728 | 6.476205 |
| C | 0.992819 | 4.909755 | 5.658957 |

| | | | |
|---|---|---|---|
| H | -0.009493 | 4.850967 | 5.196568 |
| H | 1.748038 | 4.947264 | 4.854556 |
| C | 1.116458 | 6.150626 | 6.503479 |
| H | 2.131736 | 6.224562 | 6.928746 |
| H | 0.902096 | 7.032510 | 5.877235 |
| O | 0.161691 | 6.057588 | 7.555467 |
| C | -0.177891 | 7.278432 | 8.188768 |
| H | -1.213917 | 7.166513 | 8.525008 |
| H | -0.170226 | 8.096126 | 7.448476 |
| C | 0.770641 | 7.644145 | 9.323460 |
| H | 1.775961 | 7.752250 | 8.892687 |
| H | 0.477558 | 8.639646 | 9.715201 |
| N | 0.866324 | 6.650183 | 10.394784 |
| C | 2.148123 | 6.786415 | 11.102161 |
| H | 2.151572 | 7.678966 | 11.758540 |
| H | 2.934703 | 6.923223 | 10.347959 |
| C | -0.227897 | 6.697452 | 11.368327 |
| H | -0.360537 | 7.720779 | 11.774103 |
| H | 0.060243 | 6.050482 | 12.207072 |
| C | -1.575932 | 6.215756 | 10.865561 |
| H | -2.230959 | 6.058361 | 11.740085 |
| H | -2.076880 | 6.958789 | 10.221667 |
| O | -1.415142 | 4.997679 | 10.165555 |
| C | -2.622426 | 4.383182 | 9.748631 |
| H | -2.904330 | 4.754632 | 8.746992 |
| H | -3.435619 | 4.631028 | 10.450530 |
| C | -2.441724 | 2.888365 | 9.746861 |
| H | -2.132436 | 2.551311 | 10.751983 |
| H | -3.402700 | 2.410250 | 9.485761 |
| O | -1.449018 | 2.520800 | 8.807936 |
| C | -1.232663 | 1.113035 | 8.812361 |
| H | -2.186587 | 0.599441 | 8.599426 |
| H | -0.878534 | 0.819267 | 9.812981 |
| C | -0.237398 | 0.752252 | 7.735643 |
| H | -0.182171 | -0.351375 | 7.661354 |
| H | -0.639809 | 1.109650 | 6.778033 |

La-syn-H2O-KHQ(1+)

SCF (RwB97XD) = -2018.79330160
E(SCF)+ZPE(0 K)= -2018.084969
H(298 K)= -2018.045696
G(298 K)= -2018.150270
Lowest Frequency = 23.0026cm-1

| | | | |
|---|---|---|---|
| La | 0.892641 | 4.001072 | 9.067559 |
| N | 3.110817 | 2.576732 | 9.111799 |
| C | 3.111367 | 1.265582 | 9.192787 |
| C | 4.226698 | 0.562715 | 9.703497 |
| H | 4.201905 | -0.526346 | 9.768090 |
| C | 5.325858 | 1.272441 | 10.125537 |
| H | 6.192581 | 0.750680 | 10.538455 |
| C | 5.357184 | 2.687459 | 10.015931 |
| C | 4.197806 | 3.299741 | 9.479910 |
| C | 4.099924 | 4.729230 | 9.301018 |
| O | 2.988259 | 5.226878 | 8.835361 |
| C | 5.213183 | 5.479230 | 9.673989 |

| | | | |
|---|---|---|---|
| H | 5.187205 | 6.562427 | 9.533613 |
| C | 6.363470 | 4.864910 | 10.216171 |
| H | 7.208126 | 5.498056 | 10.498407 |
| C | 6.453537 | 3.502022 | 10.396494 |
| H | 7.350695 | 3.044122 | 10.816241 |
| N | 1.809800 | 4.481514 | 11.556840 |
| C | 2.343189 | 5.621584 | 11.930004 |
| C | 3.222422 | 5.705252 | 13.035990 |
| H | 3.651685 | 6.668685 | 13.316246 |
| C | 3.537116 | 4.561688 | 13.727991 |
| H | 4.228611 | 4.599197 | 14.573180 |
| C | 2.955910 | 3.320382 | 13.359778 |
| C | 2.071202 | 3.345589 | 12.254130 |
| C | 1.399839 | 2.156717 | 11.794164 |
| O | 0.614481 | 2.252904 | 10.760172 |
| C | 1.662453 | 0.977482 | 12.487719 |
| H | 1.159393 | 0.059585 | 12.174083 |
| C | 2.555467 | 0.955802 | 13.581046 |
| H | 2.734436 | 0.006180 | 14.091073 |
| C | 3.200190 | 2.090587 | 14.022083 |
| H | 3.885429 | 2.058782 | 14.870776 |
| C | 1.856465 | 0.548587 | 8.753128 |
| H | 2.124190 | -0.413201 | 8.271776 |
| H | 1.280280 | 0.320482 | 9.659129 |
| N | 1.020304 | 1.360924 | 7.858151 |
| C | 1.690769 | 1.481120 | 6.553219 |
| H | 1.552504 | 0.558018 | 5.956708 |
| H | 2.767907 | 1.591798 | 6.733947 |
| C | 1.249722 | 2.663441 | 5.720395 |
| H | 1.793416 | 2.628561 | 4.759794 |
| H | 0.167939 | 2.639820 | 5.496343 |
| O | 1.557372 | 3.862115 | 6.400529 |
| C | 1.399258 | 5.009988 | 5.595010 |
| H | 0.423715 | 4.967724 | 5.076084 |
| H | 2.195002 | 5.046386 | 4.829743 |
| C | 1.474164 | 6.256098 | 6.441300 |
| H | 2.430118 | 6.289524 | 6.989281 |
| H | 1.381188 | 7.135721 | 5.781490 |
| O | 0.389677 | 6.228664 | 7.364759 |
| C | 0.059708 | 7.455118 | 7.981897 |
| H | -1.011301 | 7.404521 | 8.220313 |
| H | 0.185845 | 8.286357 | 7.266610 |
| C | 0.903587 | 7.739240 | 9.211037 |
| H | 1.950008 | 7.792705 | 8.881479 |
| H | 0.633905 | 8.741838 | 9.601319 |
| N | 0.839448 | 6.721507 | 10.265022 |
| C | 2.052812 | 6.835262 | 11.087698 |
| H | 2.013114 | 7.737326 | 11.729957 |
| H | 2.905414 | 6.950712 | 10.405511 |
| C | -0.343409 | 6.813667 | 11.127515 |
| H | -0.576503 | 7.869804 | 11.371638 |
| H | -0.098410 | 6.311778 | 12.073777 |
| C | -1.617198 | 6.163777 | 10.617058 |
| H | -2.377238 | 6.270395 | 11.410594 |
| H | -2.026493 | 6.656388 | 9.718159 |
| O | -1.401023 | 4.790410 | 10.351930 |
| C | -2.585700 | 4.014581 | 10.358799 |

| | | | |
|---|---|---|---|
| H | -3.237521 | 4.316525 | 9.515799 |
| H | -3.144262 | 4.200439 | 11.292497 |
| C | -2.264105 | 2.541967 | 10.291238 |
| H | -1.597452 | 2.260881 | 11.121693 |
| H | -3.209850 | 1.974626 | 10.359813 |
| O | -1.610363 | 2.262469 | 9.068739 |
| C | -1.233438 | 0.904997 | 8.902997 |
| H | -2.135612 | 0.292181 | 8.727029 |
| H | -0.745263 | 0.563233 | 9.827112 |
| C | -0.314119 | 0.767909 | 7.702979 |
| H | -0.232049 | -0.311028 | 7.461486 |
| H | -0.803754 | 1.243247 | 6.840047 |
| O | -1.167890 | 4.003847 | 7.244361 |
| H | -1.826577 | 3.429222 | 7.666012 |
| H | -1.575872 | 4.822641 | 6.949699 |

Lu-anti-KHQ-Ligand(2–)

SCF (RwB97XD) = -1910.47572041
E(SCF)+ZPE(0 K)= -1909.809129
H(298 K)= -1909.774289
G(298 K)= -1909.872107
Lowest Frequency = -59.0545cm-1

| | | | |
|---|---|---|---|
| C | 5.828291 | 1.401974 | 17.294391 |
| C | 5.494764 | 1.663667 | 18.647186 |
| C | 5.201511 | 0.533143 | 19.453717 |
| H | 4.938531 | 0.675702 | 20.504744 |
| C | 5.205908 | -0.721433 | 18.898641 |
| H | 4.932982 | -1.599004 | 19.488098 |
| C | 5.561005 | -0.876935 | 17.535483 |
| C | 5.540382 | -2.245510 | 16.904654 |
| H | 6.402542 | -2.809550 | 17.294729 |
| H | 4.650484 | -2.795299 | 17.270385 |
| C | 4.281704 | -2.132421 | 14.808675 |
| H | 3.532689 | -2.769606 | 15.319397 |
| H | 4.396383 | -2.500643 | 13.781206 |
| C | 3.727723 | -0.719750 | 14.705489 |
| H | 2.760254 | -0.767273 | 14.179706 |
| H | 3.557870 | -0.251037 | 15.688488 |
| C | 4.145889 | 1.062205 | 13.081531 |
| H | 4.054180 | 2.013249 | 13.630736 |
| H | 3.162204 | 0.768074 | 12.680907 |
| C | 5.130192 | 1.199298 | 11.935614 |
| H | 5.166593 | 0.271780 | 11.341568 |
| H | 4.822788 | 2.040126 | 11.292841 |
| C | 7.347744 | 2.219941 | 11.766395 |
| H | 6.817460 | 2.898098 | 11.078234 |
| H | 7.986157 | 1.554377 | 11.162944 |
| C | 6.381324 | -3.338724 | 14.935443 |
| H | 6.249485 | -3.361338 | 13.852488 |
| H | 6.044020 | -4.316139 | 15.335080 |
| N | 5.908438 | 0.142210 | 16.783230 |
| N | 5.593555 | -2.227110 | 15.451700 |
| O | 6.303291 | 2.157801 | 15.121782 |
| O | 4.636994 | 0.059210 | 13.950962 |
| O | 6.405521 | 1.454373 | 12.493712 |

| | | | |
|---|---|---|---|
| C | 7.549674 | -2.024487 | 12.152240 |
| C | 8.852256 | -1.417848 | 12.096394 |
| C | 9.897096 | -1.977671 | 11.318855 |
| C | 11.101752 | -1.230240 | 11.251344 |
| H | 11.941165 | -1.614113 | 10.666394 |
| C | 11.187276 | -0.014422 | 11.881188 |
| H | 12.086002 | 0.600145 | 11.799434 |
| C | 10.086447 | 0.453584 | 12.641137 |
| C | 10.155160 | 1.800692 | 13.313331 |
| H | 10.651078 | 2.517730 | 12.629301 |
| H | 10.836053 | 1.713385 | 14.174970 |
| C | 8.992950 | 3.030011 | 15.021001 |
| H | 9.765859 | 3.823118 | 14.972486 |
| H | 8.035184 | 3.513938 | 15.218929 |
| C | 9.343119 | 2.098239 | 16.185254 |
| H | 8.903559 | 2.489896 | 17.115943 |
| H | 10.433965 | 2.043698 | 16.325595 |
| C | 9.802024 | -0.241709 | 16.285607 |
| H | 10.527475 | -0.339570 | 15.457545 |
| H | 10.349325 | 0.008204 | 17.208779 |
| C | 9.080177 | -1.542027 | 16.503797 |
| H | 8.463605 | -1.490096 | 17.419591 |
| H | 9.832431 | -2.337729 | 16.627394 |
| C | 7.868686 | -3.181452 | 15.264480 |
| H | 8.473450 | -3.646244 | 14.470094 |
| H | 8.112933 | -3.686872 | 16.211910 |
| C | 8.137356 | 3.066794 | 12.752520 |
| H | 7.408781 | 3.694137 | 13.281640 |
| H | 8.814102 | 3.734133 | 12.182834 |
| N | 8.985561 | -0.248961 | 12.782210 |
| N | 8.867071 | 2.301719 | 13.765402 |
| O | 8.860870 | 0.767939 | 15.975952 |
| O | 8.251920 | -1.808923 | 15.388483 |
| O | 6.629305 | -1.438626 | 12.853193 |
| C | 7.373302 | -3.212968 | 11.442411 |
| H | 6.386610 | -3.681341 | 11.441997 |
| C | 8.431244 | -3.787456 | 10.707393 |
| H | 8.247702 | -4.719634 | 10.167847 |
| C | 9.675637 | -3.198395 | 10.634265 |
| H | 10.478260 | -3.641616 | 10.042615 |
| C | 6.037437 | 2.468396 | 16.352522 |
| C | 5.940652 | 3.772377 | 16.840304 |
| H | 6.065214 | 4.603187 | 16.142314 |
| C | 5.652715 | 4.023999 | 18.197959 |
| H | 5.588030 | 5.061096 | 18.535587 |
| C | 5.431373 | 3.004914 | 19.099802 |
| H | 5.186234 | 3.214361 | 20.142270 |

Lu-anti-KHQ(1+)

SCF (RwB97XD) = -1950.78065488
E(SCF)+ZPE(0 K)= -1950.097712
H(298 K)= -1950.060544
G(298 K)= -1950.163137
Lowest Frequency = 22.7375cm-1

| | | | |
|---|---|---|---|
| C | 5.828291 | 1.401974 | 17.294391 |

| | | | |
|---|---|---|---|
| C | 5.494764 | 1.663667 | 18.647186 |
| C | 5.201511 | 0.533143 | 19.453717 |
| H | 4.938531 | 0.675702 | 20.504744 |
| C | 5.205908 | -0.721433 | 18.898641 |
| H | 4.932982 | -1.599004 | 19.488098 |
| C | 5.561005 | -0.876935 | 17.535483 |
| C | 5.540382 | -2.245510 | 16.904654 |
| H | 6.402542 | -2.809550 | 17.294729 |
| H | 4.650484 | -2.795299 | 17.270385 |
| C | 4.281704 | -2.132421 | 14.808675 |
| H | 3.532689 | -2.769606 | 15.319397 |
| H | 4.396383 | -2.500643 | 13.781206 |
| C | 3.727723 | -0.719750 | 14.705489 |
| H | 2.760254 | -0.767273 | 14.179706 |
| H | 3.557870 | -0.251037 | 15.688488 |
| C | 4.145889 | 1.062205 | 13.081531 |
| H | 4.054180 | 2.013249 | 13.630736 |
| H | 3.162204 | 0.768074 | 12.680907 |
| C | 5.130192 | 1.199298 | 11.935614 |
| H | 5.166593 | 0.271780 | 11.341568 |
| H | 4.822788 | 2.040126 | 11.292841 |
| C | 7.347744 | 2.219941 | 11.766395 |
| H | 6.817460 | 2.898098 | 11.078234 |
| H | 7.986157 | 1.554377 | 11.162944 |
| C | 6.381324 | -3.338724 | 14.935443 |
| H | 6.249485 | -3.361338 | 13.852488 |
| H | 6.044020 | -4.316139 | 15.335080 |
| N | 5.908438 | 0.142210 | 16.783230 |
| N | 5.593555 | -2.227110 | 15.451700 |
| O | 6.303291 | 2.157801 | 15.121782 |
| O | 4.636994 | 0.059210 | 13.950962 |
| O | 6.405521 | 1.454373 | 12.493712 |
| C | 7.549674 | -2.024487 | 12.152240 |
| C | 8.852256 | -1.417848 | 12.096394 |
| C | 9.897096 | -1.977671 | 11.318855 |
| C | 11.101752 | -1.230240 | 11.251344 |
| H | 11.941165 | -1.614113 | 10.666394 |
| C | 11.187276 | -0.014422 | 11.881188 |
| H | 12.086002 | 0.600145 | 11.799434 |
| C | 10.086447 | 0.453584 | 12.641137 |
| C | 10.155160 | 1.800692 | 13.313331 |
| H | 10.651078 | 2.517730 | 12.629301 |
| H | 10.836053 | 1.713385 | 14.174970 |
| C | 8.992950 | 3.030011 | 15.021001 |
| H | 9.765859 | 3.823118 | 14.972486 |
| H | 8.035184 | 3.513938 | 15.218929 |
| C | 9.343119 | 2.098239 | 16.185254 |
| H | 8.903559 | 2.489896 | 17.115943 |
| H | 10.433965 | 2.043698 | 16.325595 |
| C | 9.802024 | -0.241709 | 16.285607 |
| H | 10.527475 | -0.339570 | 15.457545 |
| H | 10.349325 | 0.008204 | 17.208779 |
| C | 9.080177 | -1.542027 | 16.503797 |
| H | 8.463605 | -1.490096 | 17.419591 |
| H | 9.832431 | -2.337729 | 16.627394 |
| C | 7.868686 | -3.181452 | 15.264480 |
| H | 8.473450 | -3.646244 | 14.470094 |

| | | | |
|---|---|---|---|
| H | 8.112933 | -3.686872 | 16.211910 |
| C | 8.137356 | 3.066794 | 12.752520 |
| H | 7.408781 | 3.694137 | 13.281640 |
| H | 8.814102 | 3.734133 | 12.182834 |
| N | 8.985561 | -0.248961 | 12.782210 |
| N | 8.867071 | 2.301719 | 13.765402 |
| O | 8.860870 | 0.767939 | 15.975952 |
| O | 8.251920 | -1.808923 | 15.388483 |
| O | 6.629305 | -1.438626 | 12.853193 |
| C | 7.373302 | -3.212968 | 11.442411 |
| H | 6.386610 | -3.681341 | 11.441997 |
| C | 8.431244 | -3.787456 | 10.707393 |
| H | 8.247702 | -4.719634 | 10.167847 |
| C | 9.675637 | -3.198395 | 10.634265 |
| H | 10.478260 | -3.641616 | 10.042615 |
| C | 6.037437 | 2.468396 | 16.352522 |
| C | 5.940652 | 3.772377 | 16.840304 |
| H | 6.065214 | 4.603187 | 16.142314 |
| C | 5.652715 | 4.023999 | 18.197959 |
| H | 5.588030 | 5.061096 | 18.535587 |
| C | 5.431373 | 3.004914 | 19.099802 |
| H | 5.186234 | 3.214361 | 20.142270 |
| Lu | 6.995365 | 0.136430 | 14.416843 |

Lu3+_Ion

SCF (RwB97XD) = -38.4130689912
E(SCF)+ZPE(0 K)= -38.413069
H(298 K)= -38.410709
G(298 K)= -38.430372

| | | | |
|---|---|---|---|
| Lu | 0.887400 | 3.945900 | 9.220400 |

Lu-syn-KHQ_Ligand(2–)

SCF (RwB97XD) = -1910.49104648
E(SCF)+ZPE(0 K)= -1909.822476
H(298 K)= -1909.787618
G(298 K)= -1909.884447
Lowest Frequency = -38.5807cm-1

| | | | |
|---|---|---|---|
| N | 3.070425 | 2.662721 | 9.310581 |
| C | 3.151155 | 1.361520 | 9.486371 |
| C | 4.318930 | 0.768313 | 10.016252 |
| H | 4.351668 | -0.309728 | 10.181001 |
| C | 5.402836 | 1.563656 | 10.310517 |
| H | 6.310839 | 1.123001 | 10.729188 |
| C | 5.369667 | 2.954624 | 10.029095 |
| C | 4.150441 | 3.453041 | 9.517867 |
| C | 3.969139 | 4.832873 | 9.156018 |
| O | 2.782381 | 5.199685 | 8.754838 |
| C | 5.074333 | 5.665094 | 9.298128 |
| H | 4.993053 | 6.712881 | 8.999853 |
| C | 6.291741 | 5.169769 | 9.821546 |
| H | 7.130700 | 5.861623 | 9.927765 |
| C | 6.454573 | 3.854042 | 10.196820 |
| H | 7.403975 | 3.493379 | 10.595878 |

| | | | |
|---|---|---|---|
| N | 1.968979 | 4.396556 | 11.516282 |
| C | 2.613136 | 5.485683 | 11.871266 |
| C | 3.575995 | 5.463537 | 12.905855 |
| H | 4.116895 | 6.376031 | 13.161405 |
| C | 3.814559 | 4.286157 | 13.577048 |
| H | 4.563823 | 4.247298 | 14.371416 |
| C | 3.057862 | 3.123745 | 13.274145 |
| C | 2.129429 | 3.249901 | 12.217614 |
| C | 1.263884 | 2.178024 | 11.814388 |
| O | 0.483892 | 2.396682 | 10.790331 |
| C | 1.357472 | 0.992997 | 12.534983 |
| H | 0.693761 | 0.161926 | 12.285640 |
| C | 2.298555 | 0.858472 | 13.583254 |
| H | 2.348017 | -0.093412 | 14.117358 |
| C | 3.145256 | 1.880649 | 13.953665 |
| H | 3.859671 | 1.754806 | 14.768860 |
| C | 1.955377 | 0.556542 | 9.038297 |
| H | 2.298846 | -0.423450 | 8.649686 |
| H | 1.309261 | 0.359666 | 9.904311 |
| N | 1.183106 | 1.286948 | 8.035957 |
| C | 1.932588 | 1.430291 | 6.791656 |
| H | 1.971953 | 0.481656 | 6.219353 |
| H | 2.966120 | 1.706214 | 7.034634 |
| C | 1.356784 | 2.508231 | 5.905527 |
| H | 2.042348 | 2.673302 | 5.056128 |
| H | 0.375928 | 2.226859 | 5.480694 |
| O | 1.217363 | 3.705297 | 6.642878 |
| C | 0.847878 | 4.786850 | 5.816087 |
| H | -0.183928 | 4.636779 | 5.447818 |
| H | 1.522445 | 4.848622 | 4.944409 |
| C | 0.951033 | 6.060407 | 6.601769 |
| H | 1.991534 | 6.241394 | 6.912977 |
| H | 0.588706 | 6.892796 | 5.976780 |
| O | 0.128019 | 5.925565 | 7.753499 |
| C | -0.234547 | 7.159760 | 8.351561 |
| H | -1.255617 | 7.042420 | 8.726474 |
| H | -0.274184 | 7.944211 | 7.578796 |
| C | 0.743288 | 7.591953 | 9.433597 |
| H | 1.720183 | 7.744935 | 8.955146 |
| H | 0.416057 | 8.574728 | 9.831533 |
| N | 0.930122 | 6.605924 | 10.490126 |
| C | 2.236950 | 6.745457 | 11.132669 |
| H | 2.261457 | 7.603090 | 11.833970 |
| H | 2.988735 | 6.934372 | 10.356204 |
| C | -0.120847 | 6.582411 | 11.503315 |
| H | -0.288562 | 7.585102 | 11.946276 |
| H | 0.217724 | 5.924883 | 12.313117 |
| C | -1.451543 | 6.058125 | 10.998957 |
| H | -2.059844 | 5.726749 | 11.857741 |
| H | -2.026201 | 6.845145 | 10.481267 |
| O | -1.238824 | 4.971125 | 10.115987 |
| C | -2.413080 | 4.534490 | 9.452777 |
| H | -2.440513 | 4.969134 | 8.437437 |
| H | -3.309771 | 4.865760 | 9.999281 |
| C | -2.409135 | 3.035474 | 9.401003 |
| H | -2.364954 | 2.634883 | 10.428854 |
| H | -3.337209 | 2.678810 | 8.919018 |

| | | | |
|---|---|---|---|
| O | -1.282916 | 2.593002 | 8.674992 |
| C | -1.175833 | 1.175874 | 8.765345 |
| H | -2.145733 | 0.722854 | 8.495670 |
| H | -0.930439 | 0.914108 | 9.805658 |
| C | -0.126442 | 0.687761 | 7.801869 |
| H | -0.084263 | -0.417845 | 7.846657 |
| H | -0.458018 | 0.943162 | 6.786893 |

Lu-syn-KHQ(1+)

SCF (RwB97XD) = -1950.78795512
E(SCF)+ZPE(0 K)= -1950.103118
H(298 K)= -1950.066708
G(298 K)= -1950.165834
Lowest Frequency = 40.4148cm-1

| | | | |
|---|---|---|---|
| Lu | 0.976655 | 3.964926 | 9.268324 |
| N | 3.070425 | 2.662721 | 9.310581 |
| C | 3.151155 | 1.361520 | 9.486371 |
| C | 4.318930 | 0.768313 | 10.016252 |
| H | 4.351668 | -0.309728 | 10.181001 |
| C | 5.402836 | 1.563656 | 10.310517 |
| H | 6.310839 | 1.123001 | 10.729188 |
| C | 5.369667 | 2.954624 | 10.029095 |
| C | 4.150441 | 3.453041 | 9.517867 |
| C | 3.969139 | 4.832873 | 9.156018 |
| O | 2.782381 | 5.199685 | 8.754838 |
| C | 5.074333 | 5.665094 | 9.298128 |
| H | 4.993053 | 6.712881 | 8.999853 |
| C | 6.291741 | 5.169769 | 9.821546 |
| H | 7.130700 | 5.861623 | 9.927765 |
| C | 6.454573 | 3.854042 | 10.196820 |
| H | 7.403975 | 3.493379 | 10.595878 |
| N | 1.968979 | 4.396556 | 11.516282 |
| C | 2.613136 | 5.485683 | 11.871266 |
| C | 3.575995 | 5.463537 | 12.905855 |
| H | 4.116895 | 6.376031 | 13.161405 |
| C | 3.814559 | 4.286157 | 13.577048 |
| H | 4.563823 | 4.247298 | 14.371416 |
| C | 3.057862 | 3.123745 | 13.274145 |
| C | 2.129429 | 3.249901 | 12.217614 |
| C | 1.263884 | 2.178024 | 11.814388 |
| O | 0.483892 | 2.396682 | 10.790331 |
| C | 1.357472 | 0.992997 | 12.534983 |
| H | 0.693761 | 0.161926 | 12.285640 |
| C | 2.298555 | 0.858472 | 13.583254 |
| H | 2.348017 | -0.093412 | 14.117358 |
| C | 3.145256 | 1.880649 | 13.953665 |
| H | 3.859671 | 1.754806 | 14.768860 |
| C | 1.955377 | 0.556542 | 9.038297 |
| H | 2.298846 | -0.423450 | 8.649686 |
| H | 1.309261 | 0.359666 | 9.904311 |
| N | 1.183106 | 1.286948 | 8.035957 |
| C | 1.932588 | 1.430291 | 6.791656 |
| H | 1.971953 | 0.481656 | 6.219353 |
| H | 2.966120 | 1.706214 | 7.034634 |
| C | 1.356784 | 2.508231 | 5.905527 |

| | | | |
|---|---|---|---|
| H | 2.042348 | 2.673302 | 5.056128 |
| H | 0.375928 | 2.226859 | 5.480694 |
| O | 1.217363 | 3.705297 | 6.642878 |
| C | 0.847878 | 4.786850 | 5.816087 |
| H | -0.183928 | 4.636779 | 5.447818 |
| H | 1.522445 | 4.848622 | 4.944409 |
| C | 0.951033 | 6.060407 | 6.601769 |
| H | 1.991534 | 6.241394 | 6.912977 |
| H | 0.588706 | 6.892796 | 5.976780 |
| O | 0.128019 | 5.925565 | 7.753499 |
| C | -0.234547 | 7.159760 | 8.351561 |
| H | -1.255617 | 7.042420 | 8.726474 |
| H | -0.274184 | 7.944211 | 7.578796 |
| C | 0.743288 | 7.591953 | 9.433597 |
| H | 1.720183 | 7.744935 | 8.955146 |
| H | 0.416057 | 8.574728 | 9.831533 |
| N | 0.930122 | 6.605924 | 10.490126 |
| C | 2.236950 | 6.745457 | 11.132669 |
| H | 2.261457 | 7.603090 | 11.833970 |
| H | 2.988735 | 6.934372 | 10.356204 |
| C | -0.120847 | 6.582411 | 11.503315 |
| H | -0.288562 | 7.585102 | 11.946276 |
| H | 0.217724 | 5.924883 | 12.313117 |
| C | -1.451543 | 6.058125 | 10.998957 |
| H | -2.059844 | 5.726749 | 11.857741 |
| H | -2.026201 | 6.845145 | 10.481267 |
| O | -1.238824 | 4.971125 | 10.115987 |
| C | -2.413080 | 4.534490 | 9.452777 |
| H | -2.440513 | 4.969134 | 8.437437 |
| H | -3.309771 | 4.865760 | 9.999281 |
| C | -2.409135 | 3.035474 | 9.401003 |
| H | -2.364954 | 2.634883 | 10.428854 |
| H | -3.337209 | 2.678810 | 8.919018 |
| O | -1.282916 | 2.593002 | 8.674992 |
| C | -1.175833 | 1.175874 | 8.765345 |
| H | -2.145733 | 0.722854 | 8.495670 |
| H | -0.930439 | 0.914108 | 9.805658 |
| C | -0.126442 | 0.687761 | 7.801869 |
| H | -0.084263 | -0.417845 | 7.846657 |
| H | -0.458018 | 0.943162 | 6.786893 |

Lu-syn-H2O-KHQ(1+)

SCF (RwB97XD) = -2027.14968320
E(SCF)+ZPE(0 K)= -2026.439895
H(298 K)= -2026.401148
G(298 K)= -2026.505439
Lowest Frequency = 21.4374cm-1

| | | | |
|---|---|---|---|
| Lu | 1.095201 | 4.009959 | 9.123682 |
| N | 3.102931 | 2.626951 | 9.126609 |
| C | 3.124484 | 1.313274 | 9.197202 |
| C | 4.283155 | 0.624037 | 9.621985 |
| H | 4.272811 | -0.465511 | 9.680906 |
| C | 5.409824 | 1.339754 | 9.954289 |
| H | 6.310696 | 0.822847 | 10.293702 |
| C | 5.423400 | 2.753233 | 9.831537 |

| | | | |
|---|---|---|---|
| C | 4.217607 | 3.350462 | 9.396804 |
| C | 4.093085 | 4.768945 | 9.194827 |
| O | 2.931490 | 5.226984 | 8.810728 |
| C | 5.221689 | 5.540386 | 9.446677 |
| H | 5.174419 | 6.619442 | 9.283224 |
| C | 6.422990 | 4.943756 | 9.894782 |
| H | 7.284517 | 5.588233 | 10.084622 |
| C | 6.542013 | 3.585818 | 10.093295 |
| H | 7.479475 | 3.144571 | 10.435546 |
| N | 1.854673 | 4.485916 | 11.460456 |
| C | 2.361375 | 5.616493 | 11.897244 |
| C | 3.105563 | 5.678859 | 13.098484 |
| H | 3.521825 | 6.632587 | 13.427062 |
| C | 3.292913 | 4.533073 | 13.835279 |
| H | 3.873283 | 4.559581 | 14.760690 |
| C | 2.702861 | 3.312972 | 13.415430 |
| C | 1.976802 | 3.360168 | 12.203967 |
| C | 1.279055 | 2.219066 | 11.684071 |
| O | 0.637800 | 2.375261 | 10.560980 |
| C | 1.350101 | 1.043458 | 12.423365 |
| H | 0.815758 | 0.156582 | 12.074163 |
| C | 2.096588 | 0.989927 | 13.624008 |
| H | 2.133395 | 0.045736 | 14.172430 |
| C | 2.766892 | 2.084549 | 14.123764 |
| H | 3.330332 | 2.024154 | 15.056282 |
| C | 1.867067 | 0.582978 | 8.794765 |
| H | 2.141003 | -0.376542 | 8.311486 |
| H | 1.307817 | 0.343632 | 9.706577 |
| N | 1.020880 | 1.389219 | 7.910343 |
| C | 1.685429 | 1.502266 | 6.603915 |
| H | 1.518263 | 0.590646 | 5.997073 |
| H | 2.767826 | 1.581778 | 6.768997 |
| C | 1.266617 | 2.704518 | 5.797099 |
| H | 1.776289 | 2.659112 | 4.818319 |
| H | 0.177670 | 2.716319 | 5.609039 |
| O | 1.635972 | 3.888847 | 6.470640 |
| C | 1.466998 | 5.019233 | 5.642889 |
| H | 0.500178 | 4.944546 | 5.110203 |
| H | 2.270070 | 5.057184 | 4.885281 |
| C | 1.487154 | 6.287437 | 6.450830 |
| H | 2.432962 | 6.372765 | 7.007349 |
| H | 1.373015 | 7.142251 | 5.761449 |
| O | 0.391683 | 6.248016 | 7.355949 |
| C | 0.083561 | 7.475029 | 7.979096 |
| H | -0.980845 | 7.436204 | 8.246074 |
| H | 0.199691 | 8.306747 | 7.262384 |
| C | 0.962479 | 7.739200 | 9.184372 |
| H | 2.001506 | 7.787238 | 8.832190 |
| H | 0.712851 | 8.737858 | 9.597365 |
| N | 0.908411 | 6.706109 | 10.220780 |
| C | 2.116417 | 6.831399 | 11.047782 |
| H | 2.070443 | 7.733844 | 11.688488 |
| H | 2.976158 | 6.945794 | 10.375249 |
| C | -0.281263 | 6.785961 | 11.075923 |
| H | -0.563899 | 7.841235 | 11.262583 |
| H | -0.025897 | 6.348713 | 12.051009 |
| C | -1.515478 | 6.050310 | 10.592697 |

| | | | |
|---|---|---|---|
| H | -2.302114 | 6.210016 | 11.351591 |
| H | -1.911250 | 6.451009 | 9.642620 |
| O | -1.261547 | 4.668032 | 10.456534 |
| C | -2.465140 | 3.918499 | 10.491692 |
| H | -3.133176 | 4.247572 | 9.671123 |
| H | -2.989811 | 4.123163 | 11.441665 |
| C | -2.240479 | 2.429550 | 10.399609 |
| H | -1.502055 | 2.114440 | 11.151179 |
| H | -3.206416 | 1.929771 | 10.597387 |
| O | -1.776287 | 2.102722 | 9.109511 |
| C | -1.239964 | 0.806793 | 8.940531 |
| H | -2.060812 | 0.091284 | 8.754449 |
| H | -0.721380 | 0.509765 | 9.863064 |
| C | -0.301459 | 0.768439 | 7.744840 |
| H | -0.180907 | -0.295169 | 7.453830 |
| H | -0.809475 | 1.260844 | 6.902420 |
| O | -0.810344 | 3.933214 | 7.669314 |
| H | -1.500097 | 3.338932 | 8.033918 |
| H | -1.152007 | 4.762948 | 7.321168 |

Nd-anti-KHQ-Ligand(2–)

SCF (RwB97XD) = -1910.50064662
E(SCF)+ZPE(0 K)= -1909.833961
H(298 K)= -1909.800097
G(298 K)= -1909.894581
Lowest Frequency = -48.0591cm-1

| | | | |
|---|---|---|---|
| C | 5.858766 | 1.379640 | 17.387924 |
| C | 5.711695 | 1.601820 | 18.782260 |
| C | 5.382139 | 0.467484 | 19.569125 |
| H | 5.272299 | 0.579501 | 20.650730 |
| C | 5.165583 | -0.749293 | 18.969052 |
| H | 4.864487 | -1.619607 | 19.555803 |
| C | 5.320817 | -0.864483 | 17.565687 |
| C | 4.913422 | -2.132193 | 16.853332 |
| H | 5.154952 | -3.012817 | 17.473448 |
| H | 3.810105 | -2.118974 | 16.816865 |
| C | 4.393843 | -2.388189 | 14.489788 |
| H | 3.636073 | -3.144862 | 14.774998 |
| H | 4.869711 | -2.702386 | 13.552722 |
| C | 3.690964 | -1.068256 | 14.226429 |
| H | 2.870225 | -1.244137 | 13.511660 |
| H | 3.255577 | -0.633218 | 15.143877 |
| C | 4.148886 | 0.829295 | 12.768110 |
| H | 3.938415 | 1.761317 | 13.318473 |
| H | 3.227942 | 0.485366 | 12.269492 |
| C | 5.222070 | 1.060079 | 11.724613 |
| H | 5.390038 | 0.148217 | 11.126837 |
| H | 4.908292 | 1.879284 | 11.056672 |
| C | 7.341046 | 2.255948 | 11.745718 |
| H | 6.797000 | 2.979647 | 11.116086 |
| H | 7.995277 | 1.660087 | 11.088274 |
| C | 6.442566 | -3.335570 | 15.402162 |
| H | 6.770728 | -3.382968 | 14.357071 |
| H | 6.027620 | -4.325488 | 15.682946 |
| N | 5.707453 | 0.150544 | 16.825752 |

| | | | |
|---|---|---|---|
| N | 5.446061 | -2.271071 | 15.501288 |
| O | 6.249727 | 2.198527 | 15.207265 |
| O | 4.619461 | -0.157527 | 13.665290 |
| O | 6.411893 | 1.414456 | 12.404058 |
| C | 7.697275 | -2.170652 | 12.048080 |
| C | 8.880383 | -1.388662 | 11.805007 |
| C | 9.797574 | -1.743028 | 10.780245 |
| C | 10.882061 | -0.854807 | 10.568347 |
| H | 11.613205 | -1.076024 | 9.786907 |
| C | 10.989673 | 0.287440 | 11.322489 |
| H | 11.798509 | 1.000362 | 11.151767 |
| C | 10.033486 | 0.539833 | 12.335216 |
| C | 10.148972 | 1.771058 | 13.195320 |
| H | 10.654274 | 2.577274 | 12.627974 |
| H | 10.828816 | 1.521790 | 14.024062 |
| C | 9.085536 | 2.977881 | 14.988546 |
| H | 9.784162 | 3.827968 | 14.852035 |
| H | 8.116393 | 3.386042 | 15.284721 |
| C | 9.628122 | 2.111570 | 16.110236 |
| H | 9.460238 | 2.621414 | 17.073895 |
| H | 10.714860 | 1.949548 | 16.003652 |
| C | 9.748727 | -0.135115 | 16.815441 |
| H | 10.572806 | -0.480448 | 16.164657 |
| H | 10.184814 | 0.292459 | 17.733811 |
| C | 8.863110 | -1.283037 | 17.209143 |
| H | 8.124347 | -0.961792 | 17.966496 |
| H | 9.495736 | -2.071994 | 17.649570 |
| C | 7.631114 | -3.073126 | 16.301391 |
| H | 8.406771 | -3.836709 | 16.125355 |
| H | 7.336780 | -3.136442 | 17.363296 |
| C | 8.102298 | 3.040969 | 12.799341 |
| H | 7.351631 | 3.586284 | 13.386473 |
| H | 8.748598 | 3.781917 | 12.288135 |
| N | 9.037643 | -0.282715 | 12.583529 |
| N | 8.879166 | 2.231803 | 13.746893 |
| O | 8.987910 | 0.840137 | 16.130072 |
| O | 8.181629 | -1.777516 | 16.070518 |
| O | 6.866780 | -1.763717 | 12.959801 |
| C | 7.520750 | -3.315943 | 11.273359 |
| H | 6.628587 | -3.924052 | 11.437465 |
| C | 8.455592 | -3.679453 | 10.282244 |
| H | 8.274841 | -4.585225 | 9.698591 |
| C | 9.574893 | -2.918789 | 10.021270 |
| H | 10.281403 | -3.199662 | 9.238524 |
| C | 6.121868 | 2.463891 | 16.471240 |
| C | 6.232593 | 3.739113 | 17.031041 |
| H | 6.387616 | 4.590025 | 16.363859 |
| C | 6.117470 | 3.945783 | 18.421485 |
| H | 6.216068 | 4.963113 | 18.807976 |
| C | 5.869680 | 2.911670 | 19.298923 |
| H | 5.767390 | 3.089236 | 20.370729 |

Nd-anti-KHQ(1+)

SCF (RwB97XD) = -1944.31022510
E(SCF)+ZPE(0 K)= -1943.627836
H(298 K)= -1943.590584

G(298 K)= -1943.693732
Lowest Frequency = 13.3797cm-1

| | | | |
|---|---|---|---|
| C | 5.858766 | 1.379640 | 17.387924 |
| C | 5.711695 | 1.601820 | 18.782260 |
| C | 5.382139 | 0.467484 | 19.569125 |
| H | 5.272299 | 0.579501 | 20.650730 |
| C | 5.165583 | -0.749293 | 18.969052 |
| H | 4.864487 | -1.619607 | 19.555803 |
| C | 5.320817 | -0.864483 | 17.565687 |
| C | 4.913422 | -2.132193 | 16.853332 |
| H | 5.154952 | -3.012817 | 17.473448 |
| H | 3.810105 | -2.118974 | 16.816865 |
| C | 4.393843 | -2.388189 | 14.489788 |
| H | 3.636073 | -3.144862 | 14.774998 |
| H | 4.869711 | -2.702386 | 13.552722 |
| C | 3.690964 | -1.068256 | 14.226429 |
| H | 2.870225 | -1.244137 | 13.511660 |
| H | 3.255577 | -0.633218 | 15.143877 |
| C | 4.148886 | 0.829295 | 12.768110 |
| H | 3.938415 | 1.761317 | 13.318473 |
| H | 3.227942 | 0.485366 | 12.269492 |
| C | 5.222070 | 1.060079 | 11.724613 |
| H | 5.390038 | 0.148217 | 11.126837 |
| H | 4.908292 | 1.879284 | 11.056672 |
| C | 7.341046 | 2.255948 | 11.745718 |
| H | 6.797000 | 2.979647 | 11.116086 |
| H | 7.995277 | 1.660087 | 11.088274 |
| C | 6.442566 | -3.335570 | 15.402162 |
| H | 6.770728 | -3.382968 | 14.357071 |
| H | 6.027620 | -4.325488 | 15.682946 |
| N | 5.707453 | 0.150544 | 16.825752 |
| N | 5.446061 | -2.271071 | 15.501288 |
| O | 6.249727 | 2.198527 | 15.207265 |
| O | 4.619461 | -0.157527 | 13.665290 |
| O | 6.411893 | 1.414456 | 12.404058 |
| C | 7.697275 | -2.170652 | 12.048080 |
| C | 8.880383 | -1.388662 | 11.805007 |
| C | 9.797574 | -1.743028 | 10.780245 |
| C | 10.882061 | -0.854807 | 10.568347 |
| H | 11.613205 | -1.076024 | 9.786907 |
| C | 10.989673 | 0.287440 | 11.322489 |
| H | 11.798509 | 1.000362 | 11.151767 |
| C | 10.033486 | 0.539833 | 12.335216 |
| C | 10.148972 | 1.771058 | 13.195320 |
| H | 10.654274 | 2.577274 | 12.627974 |
| H | 10.828816 | 1.521790 | 14.024062 |
| C | 9.085536 | 2.977881 | 14.988546 |
| H | 9.784162 | 3.827968 | 14.852035 |
| H | 8.116393 | 3.386042 | 15.284721 |
| C | 9.628122 | 2.111570 | 16.110236 |
| H | 9.460238 | 2.621414 | 17.073895 |
| H | 10.714860 | 1.949548 | 16.003652 |
| C | 9.748727 | -0.135115 | 16.815441 |
| H | 10.572806 | -0.480448 | 16.164657 |
| H | 10.184814 | 0.292459 | 17.733811 |
| C | 8.863110 | -1.283037 | 17.209143 |

| | | | |
|---|---|---|---|
| H | 8.124347 | -0.961792 | 17.966496 |
| H | 9.495736 | -2.071994 | 17.649570 |
| C | 7.631114 | -3.073126 | 16.301391 |
| H | 8.406771 | -3.836709 | 16.125355 |
| H | 7.336780 | -3.136442 | 17.363296 |
| C | 8.102298 | 3.040969 | 12.799341 |
| H | 7.351631 | 3.586284 | 13.386473 |
| H | 8.748598 | 3.781917 | 12.288135 |
| N | 9.037643 | -0.282715 | 12.583529 |
| N | 8.879166 | 2.231803 | 13.746893 |
| O | 8.987910 | 0.840137 | 16.130072 |
| O | 8.181629 | -1.777516 | 16.070518 |
| O | 6.866780 | -1.763717 | 12.959801 |
| C | 7.520750 | -3.315943 | 11.273359 |
| H | 6.628587 | -3.924052 | 11.437465 |
| C | 8.455592 | -3.679453 | 10.282244 |
| H | 8.274841 | -4.585225 | 9.698591 |
| C | 9.574893 | -2.918789 | 10.021270 |
| H | 10.281403 | -3.199662 | 9.238524 |
| C | 6.121868 | 2.463891 | 16.471240 |
| C | 6.232593 | 3.739113 | 17.031041 |
| H | 6.387616 | 4.590025 | 16.363859 |
| C | 6.117470 | 3.945783 | 18.421485 |
| H | 6.216068 | 4.963113 | 18.807976 |
| C | 5.869680 | 2.911670 | 19.298923 |
| H | 5.767390 | 3.089236 | 20.370729 |
| Nd | 7.045009 | 0.053332 | 14.476569 |

Nd3+_Ion

SCF (RwB97XD) = -32.0519929666
E(SCF)+ZPE(0 K)= -32.051993
H(298 K)= -32.049633
G(298 K)= -32.069000

| | | | |
|---|---|---|---|
| Nd | 0.887400 | 3.945900 | 9.220400 |

Nd-syn-KHQ_Ligand(2–)

SCF (RwB97XD) = -1910.50951265
E(SCF)+ZPE(0 K)= -1909.841836
H(298 K)= -1909.805814
G(298 K)= -1909.906695
Lowest Frequency = -31.7202cm-1

| | | | |
|---|---|---|---|
| N | 3.104500 | 2.604706 | 9.228479 |
| C | 3.127097 | 1.299426 | 9.378650 |
| C | 4.254313 | 0.648479 | 9.929205 |
| H | 4.245767 | -0.433915 | 10.066630 |
| C | 5.348430 | 1.400337 | 10.291200 |
| H | 6.224338 | 0.917925 | 10.731637 |
| C | 5.368076 | 2.802837 | 10.068291 |
| C | 4.191184 | 3.360967 | 9.516340 |
| C | 4.068279 | 4.766379 | 9.216163 |
| O | 2.924798 | 5.202141 | 8.763021 |
| C | 5.187973 | 5.553785 | 9.472419 |
| H | 5.150918 | 6.618601 | 9.230808 |

| | | | |
|---|---|---|---|
| C | 6.359489 | 4.996336 | 10.033334 |
| H | 7.208417 | 5.656883 | 10.225608 |
| C | 6.466460 | 3.657521 | 10.342117 |
| H | 7.381097 | 3.247484 | 10.773458 |
| N | 1.931675 | 4.422862 | 11.567586 |
| C | 2.557670 | 5.532216 | 11.888878 |
| C | 3.542036 | 5.551792 | 12.903863 |
| H | 4.062115 | 6.481839 | 13.138735 |
| C | 3.836708 | 4.386624 | 13.572195 |
| H | 4.606312 | 4.376825 | 14.347772 |
| C | 3.126662 | 3.191331 | 13.282868 |
| C | 2.158682 | 3.276186 | 12.255149 |
| C | 1.348536 | 2.150845 | 11.866480 |
| O | 0.514904 | 2.310793 | 10.875023 |
| C | 1.549242 | 0.965650 | 12.568297 |
| H | 0.936765 | 0.095264 | 12.321701 |
| C | 2.524851 | 0.879372 | 13.587193 |
| H | 2.653165 | -0.073970 | 14.105465 |
| C | 3.311998 | 1.951784 | 13.947150 |
| H | 4.058257 | 1.865282 | 14.738613 |
| C | 1.893600 | 0.552099 | 8.926969 |
| H | 2.188939 | -0.425681 | 8.496603 |
| H | 1.269683 | 0.353186 | 9.809657 |
| N | 1.101892 | 1.331155 | 7.967490 |
| C | 1.819836 | 1.462546 | 6.696998 |
| H | 1.823156 | 0.508370 | 6.133179 |
| H | 2.864398 | 1.715934 | 6.917731 |
| C | 1.257441 | 2.547140 | 5.805399 |
| H | 1.923582 | 2.660877 | 4.932543 |
| H | 0.252014 | 2.301736 | 5.417169 |
| O | 1.198495 | 3.761619 | 6.524423 |
| C | 0.946158 | 4.883655 | 5.704769 |
| H | -0.064708 | 4.803673 | 5.264411 |
| H | 1.682624 | 4.928369 | 4.883514 |
| C | 1.066451 | 6.130882 | 6.537464 |
| H | 2.091219 | 6.232355 | 6.931387 |
| H | 0.809625 | 7.003130 | 5.913904 |
| O | 0.148134 | 6.019618 | 7.619193 |
| C | -0.199471 | 7.241625 | 8.246338 |
| H | -1.227998 | 7.120853 | 8.600993 |
| H | -0.214848 | 8.051483 | 7.497941 |
| C | 0.764555 | 7.627797 | 9.360364 |
| H | 1.758958 | 7.752908 | 8.909693 |
| H | 0.461624 | 8.618160 | 9.757845 |
| N | 0.895051 | 6.633393 | 10.425048 |
| C | 2.188782 | 6.768996 | 11.106182 |
| H | 2.201294 | 7.650136 | 11.777838 |
| H | 2.961303 | 6.923226 | 10.341535 |
| C | -0.179221 | 6.663602 | 11.419426 |
| H | -0.309605 | 7.680092 | 11.842817 |
| H | 0.123832 | 6.005475 | 12.243811 |
| C | -1.529779 | 6.185230 | 10.922669 |
| H | -2.168586 | 5.975793 | 11.798109 |
| H | -2.050032 | 6.952087 | 10.323234 |
| O | -1.365377 | 5.005910 | 10.158993 |
| C | -2.572869 | 4.445283 | 9.673021 |
| H | -2.783814 | 4.837329 | 8.661797 |

| | | | |
|---|---|---|---|
| H | -3.412592 | 4.719144 | 10.332183 |
| C | -2.448698 | 2.946304 | 9.663371 |
| H | -2.216492 | 2.588383 | 10.681876 |
| H | -3.407465 | 2.504899 | 9.337273 |
| O | -1.412813 | 2.555219 | 8.784223 |
| C | -1.232455 | 1.142726 | 8.810977 |
| H | -2.194272 | 0.650723 | 8.583227 |
| H | -0.907862 | 0.853463 | 9.822642 |
| C | -0.224272 | 0.745776 | 7.761456 |
| H | -0.172821 | -0.359558 | 7.717860 |
| H | -0.609015 | 1.078391 | 6.788044 |

Nd-syn-KHQ(1+)

SCF (RwB97XD) = -1944.32377135
E(SCF)+ZPE(0 K)= -1943.640223
H(298 K)= -1943.603651
G(298 K)= -1943.702707
Lowest Frequency = 40.4939cm-1

| | | | |
|---|---|---|---|
| Nd | 0.916804 | 3.972201 | 9.210283 |
| N | 3.104500 | 2.604706 | 9.228479 |
| C | 3.127097 | 1.299426 | 9.378650 |
| C | 4.254313 | 0.648479 | 9.929205 |
| H | 4.245767 | -0.433915 | 10.066630 |
| C | 5.348430 | 1.400337 | 10.291200 |
| H | 6.224338 | 0.917925 | 10.731637 |
| C | 5.368076 | 2.802837 | 10.068291 |
| C | 4.191184 | 3.360967 | 9.516340 |
| C | 4.068279 | 4.766379 | 9.216163 |
| O | 2.924798 | 5.202141 | 8.763021 |
| C | 5.187973 | 5.553785 | 9.472419 |
| H | 5.150918 | 6.618601 | 9.230808 |
| C | 6.359489 | 4.996336 | 10.033334 |
| H | 7.208417 | 5.656883 | 10.225608 |
| C | 6.466460 | 3.657521 | 10.342117 |
| H | 7.381097 | 3.247484 | 10.773458 |
| N | 1.931675 | 4.422862 | 11.567586 |
| C | 2.557670 | 5.532216 | 11.888878 |
| C | 3.542036 | 5.551792 | 12.903863 |
| H | 4.062115 | 6.481839 | 13.138735 |
| C | 3.836708 | 4.386624 | 13.572195 |
| H | 4.606312 | 4.376825 | 14.347772 |
| C | 3.126662 | 3.191331 | 13.282868 |
| C | 2.158682 | 3.276186 | 12.255149 |
| C | 1.348536 | 2.150845 | 11.866480 |
| O | 0.514904 | 2.310793 | 10.875023 |
| C | 1.549242 | 0.965650 | 12.568297 |
| H | 0.936765 | 0.095264 | 12.321701 |
| C | 2.524851 | 0.879372 | 13.587193 |
| H | 2.653165 | -0.073970 | 14.105465 |
| C | 3.311998 | 1.951784 | 13.947150 |
| H | 4.058257 | 1.865282 | 14.738613 |
| C | 1.893600 | 0.552099 | 8.926969 |
| H | 2.188939 | -0.425681 | 8.496603 |
| H | 1.269683 | 0.353186 | 9.809657 |
| N | 1.101892 | 1.331155 | 7.967490 |

| | | | |
|---|---|---|---|
| C | 1.819836 | 1.462546 | 6.696998 |
| H | 1.823156 | 0.508370 | 6.133179 |
| H | 2.864398 | 1.715934 | 6.917731 |
| C | 1.257441 | 2.547140 | 5.805399 |
| H | 1.923582 | 2.660877 | 4.932543 |
| H | 0.252014 | 2.301736 | 5.417169 |
| O | 1.198495 | 3.761619 | 6.524423 |
| C | 0.946158 | 4.883655 | 5.704769 |
| H | -0.064708 | 4.803673 | 5.264411 |
| H | 1.682624 | 4.928369 | 4.883514 |
| C | 1.066451 | 6.130882 | 6.537464 |
| H | 2.091219 | 6.232355 | 6.931387 |
| H | 0.809625 | 7.003130 | 5.913904 |
| O | 0.148134 | 6.019618 | 7.619193 |
| C | -0.199471 | 7.241625 | 8.246338 |
| H | -1.227998 | 7.120853 | 8.600993 |
| H | -0.214848 | 8.051483 | 7.497941 |
| C | 0.764555 | 7.627797 | 9.360364 |
| H | 1.758958 | 7.752908 | 8.909693 |
| H | 0.461624 | 8.618160 | 9.757845 |
| N | 0.895051 | 6.633393 | 10.425048 |
| C | 2.188782 | 6.768996 | 11.106182 |
| H | 2.201294 | 7.650136 | 11.777838 |
| H | 2.961303 | 6.923226 | 10.341535 |
| C | -0.179221 | 6.663602 | 11.419426 |
| H | -0.309605 | 7.680092 | 11.842817 |
| H | 0.123832 | 6.005475 | 12.243811 |
| C | -1.529779 | 6.185230 | 10.922669 |
| H | -2.168586 | 5.975793 | 11.798109 |
| H | -2.050032 | 6.952087 | 10.323234 |
| O | -1.365377 | 5.005910 | 10.158993 |
| C | -2.572869 | 4.445283 | 9.673021 |
| H | -2.783814 | 4.837329 | 8.661797 |
| H | -3.412592 | 4.719144 | 10.332183 |
| C | -2.448698 | 2.946304 | 9.663371 |
| H | -2.216492 | 2.588383 | 10.681876 |
| H | -3.407465 | 2.504899 | 9.337273 |
| O | -1.412813 | 2.555219 | 8.784223 |
| C | -1.232455 | 1.142726 | 8.810977 |
| H | -2.194272 | 0.650723 | 8.583227 |
| H | -0.907862 | 0.853463 | 9.822642 |
| C | -0.224272 | 0.745776 | 7.761456 |
| H | -0.172821 | -0.359558 | 7.717860 |
| H | -0.609015 | 1.078391 | 6.788044 |

Nd-syn-H2O-KHQ(1+)

SCF (RwB97XD) = -2020.68624387
E(SCF)+ZPE(0 K)= -2019.977368
H(298 K)= -2019.938279
G(298 K)= -2020.042591
Lowest Frequency = 24.9564cm-1

| | | | |
|---|---|---|---|
| Nd | 0.928096 | 4.002562 | 9.077741 |
| N | 3.095568 | 2.592631 | 9.124547 |
| C | 3.105233 | 1.281198 | 9.206786 |
| C | 4.231707 | 0.585816 | 9.702931 |

| | | | |
|---|---|---|---|
| H | 4.212393 | -0.503166 | 9.770328 |
| C | 5.335632 | 1.300714 | 10.103796 |
| H | 6.211142 | 0.783558 | 10.503756 |
| C | 5.360320 | 2.714849 | 9.983904 |
| C | 4.188330 | 3.318563 | 9.467867 |
| C | 4.081334 | 4.743660 | 9.275626 |
| O | 2.954911 | 5.225673 | 8.828009 |
| C | 5.198170 | 5.503403 | 9.613810 |
| H | 5.166019 | 6.584672 | 9.460579 |
| C | 6.362399 | 4.898495 | 10.138032 |
| H | 7.210901 | 5.537477 | 10.394223 |
| C | 6.461279 | 3.538174 | 10.332098 |
| H | 7.369401 | 3.088526 | 10.736918 |
| N | 1.821867 | 4.479764 | 11.527437 |
| C | 2.356754 | 5.614503 | 11.914867 |
| C | 3.215302 | 5.687362 | 13.037553 |
| H | 3.647905 | 6.646410 | 13.327495 |
| C | 3.502540 | 4.540592 | 13.736961 |
| H | 4.176161 | 4.570999 | 14.596738 |
| C | 2.910833 | 3.307244 | 13.359206 |
| C | 2.053738 | 3.343956 | 12.233401 |
| C | 1.368810 | 2.169681 | 11.761907 |
| O | 0.611616 | 2.286730 | 10.709171 |
| C | 1.586408 | 0.987656 | 12.465017 |
| H | 1.069753 | 0.079357 | 12.145521 |
| C | 2.453987 | 0.952269 | 13.579245 |
| H | 2.599856 | 0.001505 | 14.097550 |
| C | 3.113566 | 2.074348 | 14.030600 |
| H | 3.777579 | 2.030682 | 14.895470 |
| C | 1.853888 | 0.557764 | 8.771229 |
| H | 2.127630 | -0.401609 | 8.288298 |
| H | 1.278486 | 0.322811 | 9.675332 |
| N | 1.017692 | 1.368380 | 7.875973 |
| C | 1.691837 | 1.483878 | 6.572770 |
| H | 1.547331 | 0.562763 | 5.974770 |
| H | 2.769889 | 1.586018 | 6.752954 |
| C | 1.258975 | 2.670577 | 5.744943 |
| H | 1.798760 | 2.634668 | 4.782157 |
| H | 0.176038 | 2.655500 | 5.525807 |
| O | 1.577993 | 3.865299 | 6.426714 |
| C | 1.418823 | 5.007759 | 5.613998 |
| H | 0.446447 | 4.956259 | 5.089577 |
| H | 2.217782 | 5.043572 | 4.852009 |
| C | 1.478675 | 6.260342 | 6.449471 |
| H | 2.434274 | 6.312328 | 6.995235 |
| H | 1.371510 | 7.132790 | 5.782163 |
| O | 0.396326 | 6.224523 | 7.374235 |
| C | 0.064554 | 7.452524 | 7.986633 |
| H | -1.005030 | 7.401258 | 8.230663 |
| H | 0.185516 | 8.281077 | 7.267402 |
| C | 0.914296 | 7.739071 | 9.209846 |
| H | 1.958465 | 7.799076 | 8.874703 |
| H | 0.642075 | 8.738569 | 9.606202 |
| N | 0.858697 | 6.715575 | 10.257576 |
| C | 2.072761 | 6.831809 | 11.077506 |
| H | 2.030846 | 7.730602 | 11.724206 |
| H | 2.925120 | 6.953656 | 10.396549 |

| | | | |
|---|---|---|---|
| C | -0.322024 | 6.801811 | 11.122762 |
| H | -0.564175 | 7.856908 | 11.362417 |
| H | -0.072694 | 6.306796 | 12.071431 |
| C | -1.588510 | 6.138396 | 10.615162 |
| H | -2.350145 | 6.244953 | 11.407181 |
| H | -2.001397 | 6.622837 | 9.713477 |
| O | -1.364467 | 4.764666 | 10.358399 |
| C | -2.556218 | 3.997876 | 10.378254 |
| H | -3.213575 | 4.306726 | 9.541946 |
| H | -3.102867 | 4.192427 | 11.317183 |
| C | -2.260301 | 2.520812 | 10.307572 |
| H | -1.586398 | 2.229811 | 11.128176 |
| H | -3.215336 | 1.971091 | 10.393635 |
| O | -1.634166 | 2.233383 | 9.075716 |
| C | -1.238125 | 0.884419 | 8.909556 |
| H | -2.129251 | 0.256986 | 8.727678 |
| H | -0.748913 | 0.545606 | 9.834272 |
| C | -0.311404 | 0.764839 | 7.713607 |
| H | -0.217065 | -0.311556 | 7.465015 |
| H | -0.802456 | 1.240504 | 6.851750 |
| O | -1.080719 | 3.990257 | 7.324323 |
| H | -1.755255 | 3.421999 | 7.732272 |
| H | -1.458394 | 4.824947 | 7.032941 |

Pr-anti-KHQ-Ligand(2–)

SCF (RwB97XD) = -1910.50408090
E(SCF)+ZPE(0 K)= -1909.837596
H(298 K)= -1909.802673
G(298 K)= -1909.901681
Lowest Frequency = -47.9884cm-1

| | | | |
|---|---|---|---|
| C | 5.846347 | 1.384091 | 17.388088 |
| C | 5.689097 | 1.613240 | 18.780559 |
| C | 5.335644 | 0.486583 | 19.567887 |
| H | 5.216757 | 0.604109 | 20.647956 |
| C | 5.108784 | -0.729798 | 18.970208 |
| H | 4.791080 | -1.593859 | 19.557416 |
| C | 5.276530 | -0.852182 | 17.569140 |
| C | 4.862601 | -2.116113 | 16.854040 |
| H | 5.052091 | -2.997124 | 17.492143 |
| H | 3.763236 | -2.070191 | 16.769436 |
| C | 4.434899 | -2.438880 | 14.480616 |
| H | 3.678041 | -3.200899 | 14.753706 |
| H | 4.950227 | -2.764655 | 13.568450 |
| C | 3.722610 | -1.136077 | 14.164567 |
| H | 2.930945 | -1.339882 | 13.424505 |
| H | 3.247529 | -0.688662 | 15.056274 |
| C | 4.178021 | 0.790549 | 12.753485 |
| H | 3.965824 | 1.704062 | 13.333713 |
| H | 3.256445 | 0.458956 | 12.247535 |
| C | 5.246578 | 1.056917 | 11.714632 |
| H | 5.412596 | 0.165653 | 11.085664 |
| H | 4.927740 | 1.895771 | 11.074041 |
| C | 7.359355 | 2.257060 | 11.757521 |
| H | 6.807011 | 2.989925 | 11.146020 |
| H | 8.015812 | 1.682085 | 11.083966 |

| | | | |
|---|---|---|---|
| C | 6.453756 | -3.344827 | 15.495688 |
| H | 6.828817 | -3.411827 | 14.466968 |
| H | 6.030681 | -4.330155 | 15.780443 |
| N | 5.682720 | 0.155471 | 16.829268 |
| N | 5.447546 | -2.284484 | 15.526949 |
| O | 6.270823 | 2.190519 | 15.208441 |
| O | 4.656547 | -0.222845 | 13.616286 |
| O | 6.439147 | 1.392004 | 12.398589 |
| C | 7.737879 | -2.206899 | 12.042947 |
| C | 8.882989 | -1.384307 | 11.753620 |
| C | 9.749632 | -1.687576 | 10.669460 |
| C | 10.805171 | -0.772682 | 10.428746 |
| H | 11.496955 | -0.955620 | 9.602834 |
| C | 10.935655 | 0.345785 | 11.214664 |
| H | 11.724292 | 1.076739 | 11.026866 |
| C | 10.028806 | 0.549461 | 12.281752 |
| C | 10.167010 | 1.752465 | 13.177577 |
| H | 10.675362 | 2.571511 | 12.631837 |
| H | 10.849187 | 1.468734 | 13.993121 |
| C | 9.131208 | 2.948308 | 14.995741 |
| H | 9.825237 | 3.800721 | 14.850401 |
| H | 8.165922 | 3.353001 | 15.310291 |
| C | 9.692726 | 2.082752 | 16.106523 |
| H | 9.568874 | 2.608940 | 17.068271 |
| H | 10.771723 | 1.893162 | 15.968006 |
| C | 9.742829 | -0.130823 | 16.910449 |
| H | 10.578906 | -0.518043 | 16.299902 |
| H | 10.160244 | 0.327368 | 17.822823 |
| C | 8.822746 | -1.244752 | 17.322945 |
| H | 8.066031 | -0.879139 | 18.041512 |
| H | 9.425310 | -2.025822 | 17.816370 |
| C | 7.599717 | -3.055098 | 16.440068 |
| H | 8.376686 | -3.829808 | 16.330187 |
| H | 7.255824 | -3.074688 | 17.488860 |
| C | 8.117562 | 3.027440 | 12.823844 |
| H | 7.364835 | 3.554744 | 13.424577 |
| H | 8.756530 | 3.782521 | 12.324010 |
| N | 9.058569 | -0.296600 | 12.553167 |
| N | 8.905584 | 2.207784 | 13.753527 |
| O | 9.022036 | 0.829373 | 16.163089 |
| O | 8.170293 | -1.773551 | 16.182882 |
| O | 6.956275 | -1.853308 | 13.018020 |
| C | 7.542593 | -3.330887 | 11.242250 |
| H | 6.679205 | -3.969103 | 11.441742 |
| C | 8.424753 | -3.639427 | 10.186440 |
| H | 8.231140 | -4.531187 | 9.585585 |
| C | 9.509647 | -2.844164 | 9.886649 |
| H | 10.176872 | -3.084292 | 9.057261 |
| C | 6.135300 | 2.461443 | 16.470227 |
| C | 6.260690 | 3.736364 | 17.027800 |
| H | 6.435652 | 4.582816 | 16.359839 |
| C | 6.134838 | 3.949311 | 18.416370 |
| H | 6.245504 | 4.966266 | 18.800609 |
| C | 5.862114 | 2.922245 | 19.294630 |
| H | 5.752031 | 3.104958 | 20.364796 |

Pr-anti-KHQ(1+)

SCF (RwB97XD) = -1943.69177634
E(SCF)+ZPE(0 K)= -1943.009540
H(298 K)= -1942.972233
G(298 K)= -1943.075528
Lowest Frequency = 15.0646cm-1

| | | | |
|---|---|---|---|
| C | 5.846347 | 1.384091 | 17.388088 |
| C | 5.689097 | 1.613240 | 18.780559 |
| C | 5.335644 | 0.486583 | 19.567887 |
| H | 5.216757 | 0.604109 | 20.647956 |
| C | 5.108784 | -0.729798 | 18.970208 |
| H | 4.791080 | -1.593859 | 19.557416 |
| C | 5.276530 | -0.852182 | 17.569140 |
| C | 4.862601 | -2.116113 | 16.854040 |
| H | 5.052091 | -2.997124 | 17.492143 |
| H | 3.763236 | -2.070191 | 16.769436 |
| C | 4.434899 | -2.438880 | 14.480616 |
| H | 3.678041 | -3.200899 | 14.753706 |
| H | 4.950227 | -2.764655 | 13.568450 |
| C | 3.722610 | -1.136077 | 14.164567 |
| H | 2.930945 | -1.339882 | 13.424505 |
| H | 3.247529 | -0.688662 | 15.056274 |
| C | 4.178021 | 0.790549 | 12.753485 |
| H | 3.965824 | 1.704062 | 13.333713 |
| H | 3.256445 | 0.458956 | 12.247535 |
| C | 5.246578 | 1.056917 | 11.714632 |
| H | 5.412596 | 0.165653 | 11.085664 |
| H | 4.927740 | 1.895771 | 11.074041 |
| C | 7.359355 | 2.257060 | 11.757521 |
| H | 6.807011 | 2.989925 | 11.146020 |
| H | 8.015812 | 1.682085 | 11.083966 |
| C | 6.453756 | -3.344827 | 15.495688 |
| H | 6.828817 | -3.411827 | 14.466968 |
| H | 6.030681 | -4.330155 | 15.780443 |
| N | 5.682720 | 0.155471 | 16.829268 |
| N | 5.447546 | -2.284484 | 15.526949 |
| O | 6.270823 | 2.190519 | 15.208441 |
| O | 4.656547 | -0.222845 | 13.616286 |
| O | 6.439147 | 1.392004 | 12.398589 |
| C | 7.737879 | -2.206899 | 12.042947 |
| C | 8.882989 | -1.384307 | 11.753620 |
| C | 9.749632 | -1.687576 | 10.669460 |
| C | 10.805171 | -0.772682 | 10.428746 |
| H | 11.496955 | -0.955620 | 9.602834 |
| C | 10.935655 | 0.345785 | 11.214664 |
| H | 11.724292 | 1.076739 | 11.026866 |
| C | 10.028806 | 0.549461 | 12.281752 |
| C | 10.167010 | 1.752465 | 13.177577 |
| H | 10.675362 | 2.571511 | 12.631837 |
| H | 10.849187 | 1.468734 | 13.993121 |
| C | 9.131208 | 2.948308 | 14.995741 |
| H | 9.825237 | 3.800721 | 14.850401 |
| H | 8.165922 | 3.353001 | 15.310291 |
| C | 9.692726 | 2.082752 | 16.106523 |
| H | 9.568874 | 2.608940 | 17.068271 |
| H | 10.771723 | 1.893162 | 15.968006 |

C 9.742829 -0.130823 16.910449
H 10.578906 -0.518043 16.299902
H 10.160244 0.327368 17.822823
C 8.822746 -1.244752 17.322945
H 8.066031 -0.879139 18.041512
H 9.425310 -2.025822 17.816370
C 7.599717 -3.055098 16.440068
H 8.376686 -3.829808 16.330187
H 7.255824 -3.074688 17.488860
C 8.117562 3.027440 12.823844
H 7.364835 3.554744 13.424577
H 8.756530 3.782521 12.324010
N 9.058569 -0.296600 12.553167
N 8.905584 2.207784 13.753527
O 9.022036 0.829373 16.163089
O 8.170293 -1.773551 16.182882
O 6.956275 -1.853308 13.018020
C 7.542593 -3.330887 11.242250
H 6.679205 -3.969103 11.441742
C 8.424753 -3.639427 10.186440
H 8.231140 -4.531187 9.585585
C 9.509647 -2.844164 9.886649
H 10.176872 -3.084292 9.057261
C 6.135300 2.461443 16.470227
C 6.260690 3.736364 17.027800
H 6.435652 4.582816 16.359839
C 6.134838 3.949311 18.416370
H 6.245504 4.966266 18.800609
C 5.862114 2.922245 19.294630
H 5.752031 3.104958 20.364796
Pr 7.077519 0.022200 14.492220

Pr3+_Ion

SCF (RwB97XD) = -31.4459258508
E(SCF)+ZPE(0 K)= -31.445926
H(298 K)= -31.443565
G(298 K)= -31.462922

Pr 0.887400 3.945900 9.220400

Pr-syn-KHQ_Ligand(2–)

SCF (RwB97XD) = -1910.51139089
E(SCF)+ZPE(0 K)= -1909.843980
H(298 K)= -1909.808788
G(298 K)= -1909.906538
Lowest Frequency = -31.1384cm-1

N 3.113140 2.599151 9.226567
C 3.130648 1.293714 9.375076
C 4.251723 0.638587 9.933310
H 4.239718 -0.444094 10.068261
C 5.343806 1.387415 10.307263
H 6.214772 0.902111 10.754278
C 5.367800 2.790854 10.090445
C 4.197672 3.353500 9.528108

| | | | |
|---|---|---|---|
| C | 4.080720 | 4.761278 | 9.233789 |
| O | 2.944530 | 5.203491 | 8.768890 |
| C | 5.198930 | 5.545006 | 9.508360 |
| H | 5.166028 | 6.611315 | 9.272764 |
| C | 6.363059 | 4.982715 | 10.079228 |
| H | 7.210662 | 5.640854 | 10.285167 |
| C | 6.464508 | 3.641893 | 10.381187 |
| H | 7.373455 | 3.227772 | 10.820582 |
| N | 1.917814 | 4.429488 | 11.575852 |
| C | 2.537631 | 5.542702 | 11.895592 |
| C | 3.518779 | 5.570170 | 12.913604 |
| H | 4.033082 | 6.503533 | 13.148085 |
| C | 3.818726 | 4.407822 | 13.584233 |
| H | 4.586355 | 4.403759 | 14.361818 |
| C | 3.118245 | 3.207163 | 13.293701 |
| C | 2.151110 | 3.284149 | 12.264167 |
| C | 1.351522 | 2.150948 | 11.874289 |
| O | 0.516534 | 2.301103 | 10.882627 |
| C | 1.563317 | 0.967420 | 12.576163 |
| H | 0.959728 | 0.091366 | 12.327698 |
| C | 2.537653 | 0.889746 | 13.596617 |
| H | 2.674185 | -0.062611 | 14.114620 |
| C | 3.313793 | 1.969572 | 13.958396 |
| H | 4.059580 | 1.890062 | 14.751039 |
| C | 1.896092 | 0.549699 | 8.919704 |
| H | 2.189575 | -0.428343 | 8.488703 |
| H | 1.271747 | 0.351725 | 9.802479 |
| N | 1.104384 | 1.329947 | 7.960351 |
| C | 1.822844 | 1.460710 | 6.689586 |
| H | 1.825896 | 0.505897 | 6.126848 |
| H | 2.867409 | 1.713437 | 6.911329 |
| C | 1.263287 | 2.544727 | 5.794807 |
| H | 1.929360 | 2.652596 | 4.921167 |
| H | 0.256654 | 2.301806 | 5.408138 |
| O | 1.209732 | 3.762011 | 6.509773 |
| C | 0.969739 | 4.885877 | 5.688536 |
| H | -0.037611 | 4.811183 | 5.239354 |
| H | 1.713672 | 4.927544 | 4.873878 |
| C | 1.088704 | 6.132645 | 6.523102 |
| H | 2.110033 | 6.227579 | 6.927959 |
| H | 0.844778 | 7.006680 | 5.896787 |
| O | 0.157578 | 6.027599 | 7.594616 |
| C | -0.190057 | 7.249752 | 8.221403 |
| H | -1.221592 | 7.130872 | 8.568262 |
| H | -0.197364 | 8.061190 | 7.474497 |
| C | 0.766275 | 7.631624 | 9.343768 |
| H | 1.765173 | 7.751740 | 8.901622 |
| H | 0.464605 | 8.623811 | 9.737583 |
| N | 0.883891 | 6.638504 | 10.411997 |
| C | 2.172148 | 6.775416 | 11.104532 |
| H | 2.179221 | 7.660038 | 11.771642 |
| H | 2.950975 | 6.925279 | 10.345218 |
| C | -0.199264 | 6.674626 | 11.397109 |
| H | -0.331342 | 7.693490 | 11.814226 |
| H | 0.096800 | 6.020417 | 12.227210 |
| C | -1.547842 | 6.195546 | 10.894776 |
| H | -2.194002 | 6.001286 | 11.768370 |

| | | | |
|---|---|---|---|
| H | -2.060531 | 6.955960 | 10.280872 |
| O | -1.382554 | 5.004616 | 10.149655 |
| C | -2.588625 | 4.430091 | 9.676311 |
| H | -2.813059 | 4.816185 | 8.665685 |
| H | -3.424934 | 4.698303 | 10.342274 |
| C | -2.451150 | 2.931680 | 9.671257 |
| H | -2.206312 | 2.579433 | 10.688767 |
| H | -3.409559 | 2.481567 | 9.356212 |
| O | -1.420851 | 2.544839 | 8.783178 |
| C | -1.230371 | 1.133454 | 8.807758 |
| H | -2.189308 | 0.634914 | 8.582063 |
| H | -0.900770 | 0.845220 | 9.818174 |
| C | -0.222235 | 0.744248 | 7.754543 |
| H | -0.169111 | -0.360796 | 7.705476 |
| H | -0.609182 | 1.081043 | 6.783356 |

Pr-syn-KHQ(1+)

SCF (RwB97XD) = -1943.70512542
E(SCF)+ZPE(0 K)= -1943.021909
H(298 K)= -1942.985234
G(298 K)= -1943.084591
Lowest Frequency = 41.2453cm-1

| | | | |
|---|---|---|---|
| Pr | 0.912933 | 3.972685 | 9.201510 |
| N | 3.113140 | 2.599151 | 9.226567 |
| C | 3.130648 | 1.293714 | 9.375076 |
| C | 4.251723 | 0.638587 | 9.933310 |
| H | 4.239718 | -0.444094 | 10.068261 |
| C | 5.343806 | 1.387415 | 10.307263 |
| H | 6.214772 | 0.902111 | 10.754278 |
| C | 5.367800 | 2.790854 | 10.090445 |
| C | 4.197672 | 3.353500 | 9.528108 |
| C | 4.080720 | 4.761278 | 9.233789 |
| O | 2.944530 | 5.203491 | 8.768890 |
| C | 5.198930 | 5.545006 | 9.508360 |
| H | 5.166028 | 6.611315 | 9.272764 |
| C | 6.363059 | 4.982715 | 10.079228 |
| H | 7.210662 | 5.640854 | 10.285167 |
| C | 6.464508 | 3.641893 | 10.381187 |
| H | 7.373455 | 3.227772 | 10.820582 |
| N | 1.917814 | 4.429488 | 11.575852 |
| C | 2.537631 | 5.542702 | 11.895592 |
| C | 3.518779 | 5.570170 | 12.913604 |
| H | 4.033082 | 6.503533 | 13.148085 |
| C | 3.818726 | 4.407822 | 13.584233 |
| H | 4.586355 | 4.403759 | 14.361818 |
| C | 3.118245 | 3.207163 | 13.293701 |
| C | 2.151110 | 3.284149 | 12.264167 |
| C | 1.351522 | 2.150948 | 11.874289 |
| O | 0.516534 | 2.301103 | 10.882627 |
| C | 1.563317 | 0.967420 | 12.576163 |
| H | 0.959728 | 0.091366 | 12.327698 |
| C | 2.537653 | 0.889746 | 13.596617 |
| H | 2.674185 | -0.062611 | 14.114620 |
| C | 3.313793 | 1.969572 | 13.958396 |
| H | 4.059580 | 1.890062 | 14.751039 |

| | | | |
|---|---|---|---|
| C | 1.896092 | 0.549699 | 8.919704 |
| H | 2.189575 | -0.428343 | 8.488703 |
| H | 1.271747 | 0.351725 | 9.802479 |
| N | 1.104384 | 1.329947 | 7.960351 |
| C | 1.822844 | 1.460710 | 6.689586 |
| H | 1.825896 | 0.505897 | 6.126848 |
| H | 2.867409 | 1.713437 | 6.911329 |
| C | 1.263287 | 2.544727 | 5.794807 |
| H | 1.929360 | 2.652596 | 4.921167 |
| H | 0.256654 | 2.301806 | 5.408138 |
| O | 1.209732 | 3.762011 | 6.509773 |
| C | 0.969739 | 4.885877 | 5.688536 |
| H | -0.037611 | 4.811183 | 5.239354 |
| H | 1.713672 | 4.927544 | 4.873878 |
| C | 1.088704 | 6.132645 | 6.523102 |
| H | 2.110033 | 6.227579 | 6.927959 |
| H | 0.844778 | 7.006680 | 5.896787 |
| O | 0.157578 | 6.027599 | 7.594616 |
| C | -0.190057 | 7.249752 | 8.221403 |
| H | -1.221592 | 7.130872 | 8.568262 |
| H | -0.197364 | 8.061190 | 7.474497 |
| C | 0.766275 | 7.631624 | 9.343768 |
| H | 1.765173 | 7.751740 | 8.901622 |
| H | 0.464605 | 8.623811 | 9.737583 |
| N | 0.883891 | 6.638504 | 10.411997 |
| C | 2.172148 | 6.775416 | 11.104532 |
| H | 2.179221 | 7.660038 | 11.771642 |
| H | 2.950975 | 6.925279 | 10.345218 |
| C | -0.199264 | 6.674626 | 11.397109 |
| H | -0.331342 | 7.693490 | 11.814226 |
| H | 0.096800 | 6.020417 | 12.227210 |
| C | -1.547842 | 6.195546 | 10.894776 |
| H | -2.194002 | 6.001286 | 11.768370 |
| H | -2.060531 | 6.955960 | 10.280872 |
| O | -1.382554 | 5.004616 | 10.149655 |
| C | -2.588625 | 4.430091 | 9.676311 |
| H | -2.813059 | 4.816185 | 8.665685 |
| H | -3.424934 | 4.698303 | 10.342274 |
| C | -2.451150 | 2.931680 | 9.671257 |
| H | -2.206312 | 2.579433 | 10.688767 |
| H | -3.409559 | 2.481567 | 9.356212 |
| O | -1.420851 | 2.544839 | 8.783178 |
| C | -1.230371 | 1.133454 | 8.807758 |
| H | -2.189308 | 0.634914 | 8.582063 |
| H | -0.900770 | 0.845220 | 9.818174 |
| C | -0.222235 | 0.744248 | 7.754543 |
| H | -0.169111 | -0.360796 | 7.705476 |
| H | -0.609182 | 1.081043 | 6.783356 |

Pr-syn-H2O-KHQ(1+)

SCF (RwB97XD) = -2020.06803374
E(SCF)+ZPE(0 K)= -2019.359614
H(298 K)= -2019.320362
G(298 K)= -2019.424800
Lowest Frequency = 31.3267cm-1

| | | | |
|---|---|---|---|
| Pr | 0.915427 | 4.003331 | 9.073081 |
| N | 3.098778 | 2.588868 | 9.121713 |
| C | 3.105337 | 1.277608 | 9.204930 |
| C | 4.227492 | 0.580167 | 9.707989 |
| H | 4.206074 | -0.508754 | 9.775817 |
| C | 5.329560 | 1.293674 | 10.116168 |
| H | 6.201649 | 0.775369 | 10.522071 |
| C | 5.356870 | 2.707954 | 9.997751 |
| C | 4.189581 | 3.314034 | 9.473230 |
| C | 4.086056 | 4.740345 | 9.283264 |
| O | 2.964795 | 5.227116 | 8.828489 |
| C | 5.201871 | 5.497112 | 9.632535 |
| H | 5.172286 | 6.578824 | 9.481846 |
| C | 6.360903 | 4.889589 | 10.164637 |
| H | 7.208178 | 5.526834 | 10.429142 |
| C | 6.456264 | 3.528668 | 10.356464 |
| H | 7.360329 | 3.076687 | 10.767719 |
| N | 1.819028 | 4.480068 | 11.535042 |
| C | 2.355284 | 5.615840 | 11.917401 |
| C | 3.223083 | 5.690904 | 13.032869 |
| H | 3.656308 | 6.650827 | 13.318977 |
| C | 3.519457 | 4.544625 | 13.728964 |
| H | 4.200805 | 4.576277 | 14.582587 |
| C | 2.928664 | 3.309522 | 13.355409 |
| C | 2.060496 | 3.343791 | 12.237680 |
| C | 1.377502 | 2.165678 | 11.771106 |
| O | 0.609906 | 2.276938 | 10.725415 |
| C | 1.609088 | 0.984193 | 12.471182 |
| H | 1.094840 | 0.073467 | 12.154717 |
| C | 2.486916 | 0.951998 | 13.577111 |
| H | 2.642976 | 0.001367 | 14.092711 |
| C | 3.144369 | 2.077274 | 14.023654 |
| H | 3.816856 | 2.036500 | 14.882099 |
| C | 1.853390 | 0.556027 | 8.766889 |
| H | 2.125852 | -0.403808 | 8.284196 |
| H | 1.276958 | 0.322499 | 9.670879 |
| N | 1.018048 | 1.367520 | 7.871065 |
| C | 1.692362 | 1.482811 | 6.567730 |
| H | 1.550125 | 0.560397 | 5.971156 |
| H | 2.769983 | 1.587500 | 6.749036 |
| C | 1.258161 | 2.667273 | 5.736518 |
| H | 1.801948 | 2.631647 | 4.775966 |
| H | 0.176327 | 2.648158 | 5.512222 |
| O | 1.570754 | 3.863657 | 6.418182 |
| C | 1.413001 | 5.007579 | 5.607197 |
| H | 0.439535 | 4.959583 | 5.084549 |
| H | 2.210964 | 5.042595 | 4.844138 |
| C | 1.478780 | 6.258049 | 6.446178 |
| H | 2.436015 | 6.305559 | 6.989839 |
| H | 1.372694 | 7.132807 | 5.781790 |
| O | 0.398617 | 6.222557 | 7.373672 |
| C | 0.063253 | 7.449993 | 7.985516 |
| H | -1.006833 | 7.396154 | 8.227143 |
| H | 0.183997 | 8.278799 | 7.266549 |
| C | 0.909490 | 7.739177 | 9.210932 |
| H | 1.954367 | 7.800170 | 8.878047 |
| H | 0.634821 | 8.738894 | 9.605020 |

| | | | |
|---|---|---|---|
| N | 0.853283 | 6.717178 | 10.260509 |
| C | 2.067994 | 6.832748 | 11.080017 |
| H | 2.026793 | 7.731840 | 11.726363 |
| H | 2.919730 | 6.954007 | 10.398061 |
| C | -0.326976 | 6.804884 | 11.126559 |
| H | -0.565502 | 7.860185 | 11.368991 |
| H | -0.077955 | 6.306639 | 12.073637 |
| C | -1.596710 | 6.146880 | 10.618954 |
| H | -2.356604 | 6.252341 | 11.412800 |
| H | -2.009794 | 6.635546 | 9.719598 |
| O | -1.375850 | 4.773574 | 10.357082 |
| C | -2.565813 | 4.004561 | 10.372002 |
| H | -3.221081 | 4.312080 | 9.533608 |
| H | -3.116435 | 4.196133 | 11.309234 |
| C | -2.262535 | 2.528686 | 10.301778 |
| H | -1.592043 | 2.239944 | 11.126120 |
| H | -3.215121 | 1.973843 | 10.380578 |
| O | -1.626101 | 2.244095 | 9.073455 |
| C | -1.237753 | 0.892071 | 8.906526 |
| H | -2.133046 | 0.270528 | 8.725206 |
| H | -0.749986 | 0.550698 | 9.831015 |
| C | -0.312760 | 0.767479 | 7.709703 |
| H | -0.222363 | -0.309547 | 7.462522 |
| H | -0.802795 | 1.244090 | 6.847662 |
| O | -1.111290 | 3.996992 | 7.300528 |
| H | -1.778374 | 3.422512 | 7.711213 |
| H | -1.505246 | 4.820915 | 7.000835 |

Sm-anti-KHQ-Ligand(2–)

SCF (RwB97XD) = -1910.49519680
E(SCF)+ZPE(0 K)= -1909.828907
H(298 K)= -1909.794829
G(298 K)= -1909.890384
Lowest Frequency = -63.3831cm-1

| | | | |
|---|---|---|---|
| C | 5.884247 | 1.360286 | 17.383169 |
| C | 5.759406 | 1.558673 | 18.782506 |
| C | 5.473532 | 0.405109 | 19.558857 |
| H | 5.382692 | 0.498428 | 20.643979 |
| C | 5.273203 | -0.806777 | 18.944467 |
| H | 5.003084 | -1.692521 | 19.523182 |
| C | 5.402540 | -0.897909 | 17.536291 |
| C | 5.008229 | -2.166945 | 16.818481 |
| H | 5.341890 | -3.046164 | 17.395294 |
| H | 3.905461 | -2.214495 | 16.864020 |
| C | 4.331221 | -2.301149 | 14.482774 |
| H | 3.577369 | -3.055171 | 14.784699 |
| H | 4.739807 | -2.583948 | 13.504851 |
| C | 3.642324 | -0.957887 | 14.317495 |
| H | 2.774948 | -1.086077 | 13.649686 |
| H | 3.276102 | -0.553040 | 15.277665 |
| C | 4.078047 | 0.940748 | 12.841916 |
| H | 3.896862 | 1.880212 | 13.389531 |
| H | 3.139954 | 0.605894 | 12.370300 |
| C | 5.128390 | 1.146222 | 11.769324 |
| H | 5.269613 | 0.225609 | 11.178827 |

| | | | |
|---|---|---|---|
| H | 4.811645 | 1.965576 | 11.102819 |
| C | 7.294064 | 2.261136 | 11.716300 |
| H | 6.774855 | 2.961903 | 11.041546 |
| H | 7.933716 | 1.606423 | 11.101744 |
| C | 6.421573 | -3.313046 | 15.211992 |
| H | 6.661799 | -3.332969 | 14.143771 |
| H | 6.023929 | -4.310158 | 15.492049 |
| N | 5.752311 | 0.135810 | 16.804550 |
| N | 5.447443 | -2.246710 | 15.428911 |
| O | 6.210623 | 2.216903 | 15.208194 |
| O | 4.556157 | -0.044303 | 13.737676 |
| O | 6.337992 | 1.489154 | 12.419326 |
| C | 7.632739 | -2.113557 | 12.078817 |
| C | 8.878730 | -1.409167 | 11.934221 |
| C | 9.890754 | -1.875230 | 11.055374 |
| C | 11.030132 | -1.043286 | 10.907706 |
| H | 11.836497 | -1.350305 | 10.237116 |
| C | 11.095915 | 0.153253 | 11.576754 |
| H | 11.945384 | 0.826113 | 11.444282 |
| C | 10.042823 | 0.516749 | 12.451476 |
| C | 10.112644 | 1.817716 | 13.208976 |
| H | 10.601263 | 2.584296 | 12.575815 |
| H | 10.798132 | 1.662933 | 14.056063 |
| C | 8.996023 | 3.043442 | 14.956204 |
| H | 9.704137 | 3.888956 | 14.842514 |
| H | 8.019349 | 3.457223 | 15.215837 |
| C | 9.496361 | 2.170535 | 16.097736 |
| H | 9.236191 | 2.642757 | 17.059494 |
| H | 10.593557 | 2.063737 | 16.062176 |
| C | 9.766423 | -0.131803 | 16.581672 |
| H | 10.542661 | -0.395370 | 15.839873 |
| H | 10.263947 | 0.233849 | 17.495125 |
| C | 8.950139 | -1.337965 | 16.949477 |
| H | 8.263631 | -1.099597 | 17.782924 |
| H | 9.638230 | -2.135135 | 17.277582 |
| C | 7.682787 | -3.088545 | 16.018772 |
| H | 8.449732 | -3.823881 | 15.724613 |
| H | 7.483009 | -3.227377 | 17.095290 |
| C | 8.074536 | 3.077964 | 12.731391 |
| H | 7.336809 | 3.669682 | 13.289521 |
| H | 8.737420 | 3.780000 | 12.188046 |
| N | 8.997235 | -0.255049 | 12.648274 |
| N | 8.829218 | 2.291474 | 13.713647 |
| O | 8.921896 | 0.866378 | 16.043411 |
| O | 8.194791 | -1.770004 | 15.832184 |
| O | 6.719187 | -1.600330 | 12.845746 |
| C | 7.493316 | -3.308441 | 11.373475 |
| H | 6.553931 | -3.858818 | 11.460017 |
| C | 8.522597 | -3.787734 | 10.537068 |
| H | 8.367343 | -4.727146 | 10.001215 |
| C | 9.702659 | -3.097015 | 10.362763 |
| H | 10.481814 | -3.465905 | 9.693753 |
| C | 6.100093 | 2.463098 | 16.477966 |
| C | 6.187352 | 3.733500 | 17.052122 |
| H | 6.306346 | 4.596883 | 16.393716 |
| C | 6.095367 | 3.917757 | 18.447477 |
| H | 6.174648 | 4.931852 | 18.846638 |

| | | | |
|---|---|---|---|
| C | 5.893368 | 2.865009 | 19.314595 |
| H | 5.808203 | 3.024897 | 20.390674 |

Sm-anti-KHQ(1+)2

SCF (RwB97XD) = -1945.53099151
E(SCF)+ZPE(0 K)= -1944.848587
H(298 K)= -1944.811315
G(298 K)= -1944.914932
Lowest Frequency = 12.4938cm-1

| | | | |
|---|---|---|---|
| C | 5.884247 | 1.360286 | 17.383169 |
| C | 5.759406 | 1.558673 | 18.782506 |
| C | 5.473532 | 0.405109 | 19.558857 |
| H | 5.382692 | 0.498428 | 20.643979 |
| C | 5.273203 | -0.806777 | 18.944467 |
| H | 5.003084 | -1.692521 | 19.523182 |
| C | 5.402540 | -0.897909 | 17.536291 |
| C | 5.008229 | -2.166945 | 16.818481 |
| H | 5.341890 | -3.046164 | 17.395294 |
| H | 3.905461 | -2.214495 | 16.864020 |
| C | 4.331221 | -2.301149 | 14.482774 |
| H | 3.577369 | -3.055171 | 14.784699 |
| H | 4.739807 | -2.583948 | 13.504851 |
| C | 3.642324 | -0.957887 | 14.317495 |
| H | 2.774948 | -1.086077 | 13.649686 |
| H | 3.276102 | -0.553040 | 15.277665 |
| C | 4.078047 | 0.940748 | 12.841916 |
| H | 3.896862 | 1.880212 | 13.389531 |
| H | 3.139954 | 0.605894 | 12.370300 |
| C | 5.128390 | 1.146222 | 11.769324 |
| H | 5.269613 | 0.225609 | 11.178827 |
| H | 4.811645 | 1.965576 | 11.102819 |
| C | 7.294064 | 2.261136 | 11.716300 |
| H | 6.774855 | 2.961903 | 11.041546 |
| H | 7.933716 | 1.606423 | 11.101744 |
| C | 6.421573 | -3.313046 | 15.211992 |
| H | 6.661799 | -3.332969 | 14.143771 |
| H | 6.023929 | -4.310158 | 15.492049 |
| N | 5.752311 | 0.135810 | 16.804550 |
| N | 5.447443 | -2.246710 | 15.428911 |
| O | 6.210623 | 2.216903 | 15.208194 |
| O | 4.556157 | -0.044303 | 13.737676 |
| O | 6.337992 | 1.489154 | 12.419326 |
| C | 7.632739 | -2.113557 | 12.078817 |
| C | 8.878730 | -1.409167 | 11.934221 |
| C | 9.890754 | -1.875230 | 11.055374 |
| C | 11.030132 | -1.043286 | 10.907706 |
| H | 11.836497 | -1.350305 | 10.237116 |
| C | 11.095915 | 0.153253 | 11.576754 |
| H | 11.945384 | 0.826113 | 11.444282 |
| C | 10.042823 | 0.516749 | 12.451476 |
| C | 10.112644 | 1.817716 | 13.208976 |
| H | 10.601263 | 2.584296 | 12.575815 |
| H | 10.798132 | 1.662933 | 14.056063 |
| C | 8.996023 | 3.043442 | 14.956204 |
| H | 9.704137 | 3.888956 | 14.842514 |

| | | | |
|---|---|---|---|
| H | 8.019349 | 3.457223 | 15.215837 |
| C | 9.496361 | 2.170535 | 16.097736 |
| H | 9.236191 | 2.642757 | 17.059494 |
| H | 10.593557 | 2.063737 | 16.062176 |
| C | 9.766423 | -0.131803 | 16.581672 |
| H | 10.542661 | -0.395370 | 15.839873 |
| H | 10.263947 | 0.233849 | 17.495125 |
| C | 8.950139 | -1.337965 | 16.949477 |
| H | 8.263631 | -1.099597 | 17.782924 |
| H | 9.638230 | -2.135135 | 17.277582 |
| C | 7.682787 | -3.088545 | 16.018772 |
| H | 8.449732 | -3.823881 | 15.724613 |
| H | 7.483009 | -3.227377 | 17.095290 |
| C | 8.074536 | 3.077964 | 12.731391 |
| H | 7.336809 | 3.669682 | 13.289521 |
| H | 8.737420 | 3.780000 | 12.188046 |
| N | 8.997235 | -0.255049 | 12.648274 |
| N | 8.829218 | 2.291474 | 13.713647 |
| O | 8.921896 | 0.866378 | 16.043411 |
| O | 8.194791 | -1.770004 | 15.832184 |
| O | 6.719187 | -1.600330 | 12.845746 |
| C | 7.493316 | -3.308441 | 11.373475 |
| H | 6.553931 | -3.858818 | 11.460017 |
| C | 8.522597 | -3.787734 | 10.537068 |
| H | 8.367343 | -4.727146 | 10.001215 |
| C | 9.702659 | -3.097015 | 10.362763 |
| H | 10.481814 | -3.465905 | 9.693753 |
| C | 6.100093 | 2.463098 | 16.477966 |
| C | 6.187352 | 3.733500 | 17.052122 |
| H | 6.306346 | 4.596883 | 16.393716 |
| C | 6.095367 | 3.917757 | 18.447477 |
| H | 6.174648 | 4.931852 | 18.846638 |
| C | 5.893368 | 2.865009 | 19.314595 |
| H | 5.808203 | 3.024897 | 20.390674 |
| Sm | 6.977950 | 0.115972 | 14.433727 |

Sm3+_Ion

SCF (RwB97XD) = -33.2499966812
E(SCF)+ZPE(0 K)= -33.249997
H(298 K)= -33.247636
G(298 K)= -33.267100

| | | | |
|---|---|---|---|
| Sm | 0.887400 | 3.945900 | 9.220400 |

Sm-syn-KHQ_Ligand(2–)

SCF (RwB97XD) = -1910.50576787
E(SCF)+ZPE(0 K)= -1909.837807
H(298 K)= -1909.801843
G(298 K)= -1909.902823
Lowest Frequency = -35.9308cm-1

| | | | |
|---|---|---|---|
| N | 3.094179 | 2.614824 | 9.246232 |
| C | 3.125477 | 1.310656 | 9.405856 |
| C | 4.259950 | 0.670463 | 9.953853 |
| H | 4.257171 | -0.410658 | 10.101002 |

| | | | |
|---|---|---|---|
| C | 5.354658 | 1.429935 | 10.298177 |
| H | 6.236451 | 0.955284 | 10.735295 |
| C | 5.367353 | 2.829493 | 10.057501 |
| C | 4.182118 | 3.376315 | 9.513364 |
| C | 4.049715 | 4.775903 | 9.195372 |
| O | 2.896420 | 5.197740 | 8.753969 |
| C | 5.169468 | 5.571331 | 9.422965 |
| H | 5.125757 | 6.632187 | 9.165653 |
| C | 6.350452 | 5.025760 | 9.976489 |
| H | 7.199766 | 5.691748 | 10.146999 |
| C | 6.466055 | 3.692263 | 10.304477 |
| H | 7.387944 | 3.291967 | 10.729479 |
| N | 1.944449 | 4.415654 | 11.553527 |
| C | 2.580225 | 5.518571 | 11.878000 |
| C | 3.564760 | 5.526216 | 12.892752 |
| H | 4.094530 | 6.450526 | 13.128521 |
| C | 3.845307 | 4.357798 | 13.562020 |
| H | 4.614161 | 4.339395 | 14.338191 |
| C | 3.118502 | 3.172131 | 13.274910 |
| C | 2.154992 | 3.268795 | 12.244776 |
| C | 1.326657 | 2.157634 | 11.857232 |
| O | 0.502103 | 2.332651 | 10.860665 |
| C | 1.502056 | 0.971935 | 12.564222 |
| H | 0.874011 | 0.111910 | 12.320501 |
| C | 2.473925 | 0.872442 | 13.586089 |
| H | 2.583272 | -0.080843 | 14.108780 |
| C | 3.280183 | 1.931361 | 13.943611 |
| H | 4.022540 | 1.833996 | 14.737476 |
| C | 1.897578 | 0.554394 | 8.955429 |
| H | 2.200154 | -0.424569 | 8.532780 |
| H | 1.268327 | 0.357202 | 9.834475 |
| N | 1.112026 | 1.326349 | 7.986836 |
| C | 1.837722 | 1.455872 | 6.721628 |
| H | 1.846337 | 0.501448 | 6.158295 |
| H | 2.880469 | 1.711998 | 6.947077 |
| C | 1.275236 | 2.538545 | 5.829002 |
| H | 1.947486 | 2.661666 | 4.962113 |
| H | 0.275208 | 2.286272 | 5.431351 |
| O | 1.199389 | 3.749971 | 6.551324 |
| C | 0.925763 | 4.864667 | 5.729149 |
| H | -0.089474 | 4.771408 | 5.301540 |
| H | 1.651463 | 4.912724 | 4.898580 |
| C | 1.042758 | 6.116579 | 6.553478 |
| H | 2.071874 | 6.235140 | 6.929744 |
| H | 0.761589 | 6.981784 | 5.930829 |
| O | 0.145209 | 5.996304 | 7.651366 |
| C | -0.209012 | 7.219616 | 8.272774 |
| H | -1.234000 | 7.094293 | 8.635389 |
| H | -0.235527 | 8.023855 | 7.518955 |
| C | 0.760410 | 7.618728 | 9.377044 |
| H | 1.749250 | 7.753705 | 8.917361 |
| H | 0.450407 | 8.606100 | 9.776582 |
| N | 0.909122 | 6.624993 | 10.438475 |
| C | 2.209034 | 6.760641 | 11.105455 |
| H | 2.227610 | 7.637070 | 11.783114 |
| H | 2.973964 | 6.921161 | 10.334752 |
| C | -0.155113 | 6.644855 | 11.442740 |

| | | | |
|---|---|---|---|
| H | -0.286713 | 7.657631 | 11.874510 |
| H | 0.156180 | 5.981967 | 12.259951 |
| C | -1.505345 | 6.164674 | 10.948008 |
| H | -2.136298 | 5.927461 | 11.821940 |
| H | -2.036194 | 6.941430 | 10.370867 |
| O | -1.336258 | 5.006738 | 10.152820 |
| C | -2.540843 | 4.477472 | 9.626700 |
| H | -2.710415 | 4.881122 | 8.612256 |
| H | -3.394424 | 4.765637 | 10.261137 |
| C | -2.447771 | 2.977301 | 9.609116 |
| H | -2.260734 | 2.607766 | 10.632649 |
| H | -3.402285 | 2.556896 | 9.244929 |
| O | -1.388199 | 2.573340 | 8.765750 |
| C | -1.228192 | 1.158731 | 8.805530 |
| H | -2.193674 | 0.678755 | 8.568099 |
| H | -0.921940 | 0.873028 | 9.823708 |
| C | -0.211654 | 0.740389 | 7.773723 |
| H | -0.163481 | -0.365599 | 7.748053 |
| H | -0.584470 | 1.058607 | 6.791060 |

Sm-syn-KHQ(1+)

SCF (RwB97XD) = -1945.54409397
E(SCF)+ZPE(0 K)= -1944.860709
H(298 K)= -1944.824024
G(298 K)= -1944.923704
Lowest Frequency = 39.3530cm-1

| | | | |
|---|---|---|---|
| Sm | 0.927939 | 3.970804 | 9.221802 |
| N | 3.094179 | 2.614824 | 9.246232 |
| C | 3.125477 | 1.310656 | 9.405856 |
| C | 4.259950 | 0.670463 | 9.953853 |
| H | 4.257171 | -0.410658 | 10.101002 |
| C | 5.354658 | 1.429935 | 10.298177 |
| H | 6.236451 | 0.955284 | 10.735295 |
| C | 5.367353 | 2.829493 | 10.057501 |
| C | 4.182118 | 3.376315 | 9.513364 |
| C | 4.049715 | 4.775903 | 9.195372 |
| O | 2.896420 | 5.197740 | 8.753969 |
| C | 5.169468 | 5.571331 | 9.422965 |
| H | 5.125757 | 6.632187 | 9.165653 |
| C | 6.350452 | 5.025760 | 9.976489 |
| H | 7.199766 | 5.691748 | 10.146999 |
| C | 6.466055 | 3.692263 | 10.304477 |
| H | 7.387944 | 3.291967 | 10.729479 |
| N | 1.944449 | 4.415654 | 11.553527 |
| C | 2.580225 | 5.518571 | 11.878000 |
| C | 3.564760 | 5.526216 | 12.892752 |
| H | 4.094530 | 6.450526 | 13.128521 |
| C | 3.845307 | 4.357798 | 13.562020 |
| H | 4.614161 | 4.339395 | 14.338191 |
| C | 3.118502 | 3.172131 | 13.274910 |
| C | 2.154992 | 3.268795 | 12.244776 |
| C | 1.326657 | 2.157634 | 11.857232 |
| O | 0.502103 | 2.332651 | 10.860665 |
| C | 1.502056 | 0.971935 | 12.564222 |
| H | 0.874011 | 0.111910 | 12.320501 |

| | | | |
|---|---|---|---|
| C | 2.473925 | 0.872442 | 13.586089 |
| H | 2.583272 | -0.080843 | 14.108780 |
| C | 3.280183 | 1.931361 | 13.943611 |
| H | 4.022540 | 1.833996 | 14.737476 |
| C | 1.897578 | 0.554394 | 8.955429 |
| H | 2.200154 | -0.424569 | 8.532780 |
| H | 1.268327 | 0.357202 | 9.834475 |
| N | 1.112026 | 1.326349 | 7.986836 |
| C | 1.837722 | 1.455872 | 6.721628 |
| H | 1.846337 | 0.501448 | 6.158295 |
| H | 2.880469 | 1.711998 | 6.947077 |
| C | 1.275236 | 2.538545 | 5.829002 |
| H | 1.947486 | 2.661666 | 4.962113 |
| H | 0.275208 | 2.286272 | 5.431351 |
| O | 1.199389 | 3.749971 | 6.551324 |
| C | 0.925763 | 4.864667 | 5.729149 |
| H | -0.089474 | 4.771408 | 5.301540 |
| H | 1.651463 | 4.912724 | 4.898580 |
| C | 1.042758 | 6.116579 | 6.553478 |
| H | 2.071874 | 6.235140 | 6.929744 |
| H | 0.761589 | 6.981784 | 5.930829 |
| O | 0.145209 | 5.996304 | 7.651366 |
| C | -0.209012 | 7.219616 | 8.272774 |
| H | -1.234000 | 7.094293 | 8.635389 |
| H | -0.235527 | 8.023855 | 7.518955 |
| C | 0.760410 | 7.618728 | 9.377044 |
| H | 1.749250 | 7.753705 | 8.917361 |
| H | 0.450407 | 8.606100 | 9.776582 |
| N | 0.909122 | 6.624993 | 10.438475 |
| C | 2.209034 | 6.760641 | 11.105455 |
| H | 2.227610 | 7.637070 | 11.783114 |
| H | 2.973964 | 6.921161 | 10.334752 |
| C | -0.155113 | 6.644855 | 11.442740 |
| H | -0.286713 | 7.657631 | 11.874510 |
| H | 0.156180 | 5.981967 | 12.259951 |
| C | -1.505345 | 6.164674 | 10.948008 |
| H | -2.136298 | 5.927461 | 11.821940 |
| H | -2.036194 | 6.941430 | 10.370867 |
| O | -1.336258 | 5.006738 | 10.152820 |
| C | -2.540843 | 4.477472 | 9.626700 |
| H | -2.710415 | 4.881122 | 8.612256 |
| H | -3.394424 | 4.765637 | 10.261137 |
| C | -2.447771 | 2.977301 | 9.609116 |
| H | -2.260734 | 2.607766 | 10.632649 |
| H | -3.402285 | 2.556896 | 9.244929 |
| O | -1.388199 | 2.573340 | 8.765750 |
| C | -1.228192 | 1.158731 | 8.805530 |
| H | -2.193674 | 0.678755 | 8.568099 |
| H | -0.921940 | 0.873028 | 9.823708 |
| C | -0.211654 | 0.740389 | 7.773723 |
| H | -0.163481 | -0.365599 | 7.748053 |
| H | -0.584470 | 1.058607 | 6.791060 |

Sm-syn-H2O-KHQ(1+)

SCF (RwB97XD) = -2021.90572700
E(SCF)+ZPE(0 K)= -2021.196321

H(298 K)= -2021.157448
G(298 K)= -2021.261151
Lowest Frequency = 24.7545cm-1

| | | | |
|---|---|---|---|
| Sm | 0.958098 | 4.012483 | 9.081065 |
| N | 3.092713 | 2.605159 | 9.126776 |
| C | 3.104391 | 1.293492 | 9.209658 |
| C | 4.236686 | 0.599725 | 9.694662 |
| H | 4.218335 | -0.489203 | 9.762910 |
| C | 5.345615 | 1.314833 | 10.081272 |
| H | 6.225840 | 0.798076 | 10.471284 |
| C | 5.369027 | 2.728515 | 9.957284 |
| C | 4.190527 | 3.330436 | 9.455179 |
| C | 4.081339 | 4.753655 | 9.257559 |
| O | 2.948011 | 5.230593 | 8.821291 |
| C | 5.201360 | 5.515939 | 9.577054 |
| H | 5.167001 | 6.596415 | 9.418986 |
| C | 6.372777 | 4.913191 | 10.088278 |
| H | 7.224631 | 5.553282 | 10.330077 |
| C | 6.474447 | 3.553769 | 10.286895 |
| H | 7.388237 | 3.106100 | 10.681002 |
| N | 1.830680 | 4.482915 | 11.509648 |
| C | 2.364622 | 5.614338 | 11.907977 |
| C | 3.205073 | 5.680331 | 13.044483 |
| H | 3.638708 | 6.636396 | 13.342573 |
| C | 3.471074 | 4.531488 | 13.749530 |
| H | 4.129361 | 4.557342 | 14.621253 |
| C | 2.873568 | 3.303489 | 13.363627 |
| C | 2.039358 | 3.347313 | 12.221653 |
| C | 1.345332 | 2.183307 | 11.740906 |
| O | 0.609867 | 2.313374 | 10.674456 |
| C | 1.529833 | 1.000479 | 12.451444 |
| H | 1.004267 | 0.098970 | 12.127243 |
| C | 2.376500 | 0.956323 | 13.581958 |
| H | 2.497616 | 0.005438 | 14.106350 |
| C | 3.045175 | 2.069410 | 14.042018 |
| H | 3.691657 | 2.018151 | 14.919652 |
| C | 1.852662 | 0.567861 | 8.780377 |
| H | 2.127658 | -0.391880 | 8.298745 |
| H | 1.279615 | 0.332455 | 9.685475 |
| N | 1.014849 | 1.375495 | 7.884974 |
| C | 1.689884 | 1.489068 | 6.582437 |
| H | 1.542095 | 0.569312 | 5.983205 |
| H | 2.768457 | 1.587306 | 6.761361 |
| C | 1.260735 | 2.678342 | 5.758232 |
| H | 1.795148 | 2.640840 | 4.792543 |
| H | 0.176548 | 2.669368 | 5.545251 |
| O | 1.590045 | 3.870751 | 6.439141 |
| C | 1.429810 | 5.010101 | 5.622415 |
| H | 0.457413 | 4.954831 | 5.098281 |
| H | 2.228396 | 5.044196 | 4.859968 |
| C | 1.484582 | 6.265948 | 6.451891 |
| H | 2.441405 | 6.327914 | 6.993725 |
| H | 1.368056 | 7.134534 | 5.781121 |
| O | 0.405848 | 6.225005 | 7.380014 |
| C | 0.071052 | 7.453794 | 7.988681 |
| H | -0.997778 | 7.400927 | 8.235282 |

| | | | |
|---|---|---|---|
| H | 0.188696 | 8.280538 | 7.266944 |
| C | 0.922971 | 7.742656 | 9.209099 |
| H | 1.966043 | 7.807004 | 8.871551 |
| H | 0.648168 | 8.739918 | 9.609145 |
| N | 0.872425 | 6.715298 | 10.252591 |
| C | 2.086255 | 6.833465 | 11.072275 |
| H | 2.042711 | 7.730895 | 11.720639 |
| H | 2.938896 | 6.957661 | 10.392179 |
| C | -0.309265 | 6.793753 | 11.117001 |
| H | -0.561733 | 7.847446 | 11.351796 |
| H | -0.057136 | 6.305430 | 12.068298 |
| C | -1.568304 | 6.116304 | 10.610573 |
| H | -2.333961 | 6.227366 | 11.398095 |
| H | -1.980317 | 6.589475 | 9.702523 |
| O | -1.337552 | 4.741118 | 10.368384 |
| C | -2.533430 | 3.979321 | 10.397593 |
| H | -3.195326 | 4.293061 | 9.566670 |
| H | -3.070753 | 4.179362 | 11.340755 |
| C | -2.257290 | 2.498766 | 10.324022 |
| H | -1.567465 | 2.203144 | 11.129263 |
| H | -3.217611 | 1.962985 | 10.436988 |
| O | -1.668198 | 2.200789 | 9.078124 |
| C | -1.241927 | 0.862800 | 8.913360 |
| H | -2.117456 | 0.214249 | 8.729389 |
| H | -0.746919 | 0.533204 | 9.838223 |
| C | -0.310210 | 0.763291 | 7.718990 |
| H | -0.205486 | -0.310011 | 7.460992 |
| H | -0.804986 | 1.241511 | 6.860659 |
| O | -1.030384 | 3.978438 | 7.389114 |
| H | -1.705434 | 3.401534 | 7.787551 |
| H | -1.407916 | 4.810734 | 7.090827 |

Tb-anti-KHQ-Ligand(2–)

SCF (RwB97XD) = -1910.48877346
E(SCF)+ZPE(0 K)= -1909.822433
H(298 K)= -1909.788357
G(298 K)= -1909.883823
Lowest Frequency = -83.3634cm-1

| | | | |
|---|---|---|---|
| C | 5.882564 | 1.363434 | 17.362377 |
| C | 5.725195 | 1.567149 | 18.756586 |
| C | 5.459984 | 0.410525 | 19.536279 |
| H | 5.344506 | 0.507792 | 20.618661 |
| C | 5.307688 | -0.808768 | 18.925450 |
| H | 5.051186 | -1.699175 | 19.503279 |
| C | 5.467883 | -0.905550 | 17.519978 |
| C | 5.137457 | -2.201188 | 16.818317 |
| H | 5.614091 | -3.039203 | 17.352453 |
| H | 4.053558 | -2.368703 | 16.960744 |
| C | 4.285050 | -2.221573 | 14.533525 |
| H | 3.527169 | -2.956273 | 14.871001 |
| H | 4.612498 | -2.495941 | 13.522976 |
| C | 3.635093 | -0.852315 | 14.440741 |
| H | 2.720710 | -0.934862 | 13.831471 |
| H | 3.351018 | -0.453787 | 15.430357 |
| C | 4.060024 | 1.011461 | 12.910500 |

| | | | |
|---|---|---|---|
| H | 3.897199 | 1.961353 | 13.445317 |
| H | 3.111864 | 0.676582 | 12.459755 |
| C | 5.096308 | 1.187672 | 11.818476 |
| H | 5.217347 | 0.256425 | 11.240835 |
| H | 4.781281 | 2.003109 | 11.146560 |
| C | 7.282961 | 2.263783 | 11.722574 |
| H | 6.770890 | 2.946405 | 11.024397 |
| H | 7.919132 | 1.584131 | 11.131795 |
| C | 6.390371 | -3.312166 | 15.064580 |
| H | 6.517004 | -3.321559 | 13.978762 |
| H | 6.005726 | -4.307495 | 15.367411 |
| N | 5.802088 | 0.131296 | 16.786589 |
| N | 5.466540 | -2.232793 | 15.397771 |
| O | 6.210157 | 2.212933 | 15.189245 |
| O | 4.543011 | 0.037164 | 13.816028 |
| O | 6.319614 | 1.521771 | 12.447145 |
| C | 7.586644 | -2.060383 | 12.126224 |
| C | 8.870703 | -1.415487 | 12.060088 |
| C | 9.931331 | -1.958484 | 11.290868 |
| C | 11.112562 | -1.176661 | 11.205551 |
| H | 11.960526 | -1.545582 | 10.623275 |
| C | 11.166546 | 0.050579 | 11.816779 |
| H | 12.047940 | 0.688090 | 11.722612 |
| C | 10.056870 | 0.496799 | 12.576961 |
| C | 10.102239 | 1.849654 | 13.240948 |
| H | 10.569572 | 2.573822 | 12.544772 |
| H | 10.801681 | 1.780845 | 14.088716 |
| C | 8.951177 | 3.073911 | 14.965557 |
| H | 9.683015 | 3.902772 | 14.884755 |
| H | 7.976814 | 3.510520 | 15.192581 |
| C | 9.387193 | 2.173956 | 16.120401 |
| H | 9.020879 | 2.594420 | 17.070427 |
| H | 10.485678 | 2.116332 | 16.178560 |
| C | 9.818165 | -0.166499 | 16.297864 |
| H | 10.490853 | -0.335941 | 15.437081 |
| H | 10.425897 | 0.129114 | 17.168176 |
| C | 9.085159 | -1.429049 | 16.650849 |
| H | 8.500805 | -1.286711 | 17.578763 |
| H | 9.826846 | -2.228078 | 16.818679 |
| C | 7.735467 | -3.123932 | 15.736815 |
| H | 8.471245 | -3.818766 | 15.299317 |
| H | 7.664292 | -3.347989 | 16.815149 |
| C | 8.069375 | 3.102919 | 12.715278 |
| H | 7.336534 | 3.716819 | 13.255292 |
| H | 8.738335 | 3.784279 | 12.153441 |
| N | 8.975027 | -0.232975 | 12.728728 |
| N | 8.812833 | 2.332484 | 13.716105 |
| O | 8.881767 | 0.844588 | 15.978185 |
| O | 8.208149 | -1.785343 | 15.598798 |
| O | 6.639490 | -1.483301 | 12.800869 |
| C | 7.452569 | -3.274626 | 11.452295 |
| H | 6.483293 | -3.777781 | 11.470811 |
| C | 8.526954 | -3.831357 | 10.728025 |
| H | 8.374450 | -4.783426 | 10.214171 |
| C | 9.749045 | -3.200525 | 10.633834 |
| H | 10.564691 | -3.629503 | 10.049517 |
| C | 6.072693 | 2.466374 | 16.454978 |

| | | | |
|---|---|---|---|
| C | 6.105827 | 3.743873 | 17.018006 |
| H | 6.205910 | 4.605159 | 16.353829 |
| C | 5.984594 | 3.934841 | 18.410087 |
| H | 6.022062 | 4.953622 | 18.803236 |
| C | 5.804249 | 2.881022 | 19.280810 |
| H | 5.694534 | 3.044889 | 20.354059 |

Tb-anti-KHQ(1+)

SCF (RwB97XD) = -1947.31128663
E(SCF)+ZPE(0 K)= -1946.628546
H(298 K)= -1946.591406
G(298 K)= -1946.694192
Lowest Frequency = 16.9225cm-1

| | | | |
|---|---|---|---|
| C | 5.882564 | 1.363434 | 17.362377 |
| C | 5.725195 | 1.567149 | 18.756586 |
| C | 5.459984 | 0.410525 | 19.536279 |
| H | 5.344506 | 0.507792 | 20.618661 |
| C | 5.307688 | -0.808768 | 18.925450 |
| H | 5.051186 | -1.699175 | 19.503279 |
| C | 5.467883 | -0.905550 | 17.519978 |
| C | 5.137457 | -2.201188 | 16.818317 |
| H | 5.614091 | -3.039203 | 17.352453 |
| H | 4.053558 | -2.368703 | 16.960744 |
| C | 4.285050 | -2.221573 | 14.533525 |
| H | 3.527169 | -2.956273 | 14.871001 |
| H | 4.612498 | -2.495941 | 13.522976 |
| C | 3.635093 | -0.852315 | 14.440741 |
| H | 2.720710 | -0.934862 | 13.831471 |
| H | 3.351018 | -0.453787 | 15.430357 |
| C | 4.060024 | 1.011461 | 12.910500 |
| H | 3.897199 | 1.961353 | 13.445317 |
| H | 3.111864 | 0.676582 | 12.459755 |
| C | 5.096308 | 1.187672 | 11.818476 |
| H | 5.217347 | 0.256425 | 11.240835 |
| H | 4.781281 | 2.003109 | 11.146560 |
| C | 7.282961 | 2.263783 | 11.722574 |
| H | 6.770890 | 2.946405 | 11.024397 |
| H | 7.919132 | 1.584131 | 11.131795 |
| C | 6.390371 | -3.312166 | 15.064580 |
| H | 6.517004 | -3.321559 | 13.978762 |
| H | 6.005726 | -4.307495 | 15.367411 |
| N | 5.802088 | 0.131296 | 16.786589 |
| N | 5.466540 | -2.232793 | 15.397771 |
| O | 6.210157 | 2.212933 | 15.189245 |
| O | 4.543011 | 0.037164 | 13.816028 |
| O | 6.319614 | 1.521771 | 12.447145 |
| C | 7.586644 | -2.060383 | 12.126224 |
| C | 8.870703 | -1.415487 | 12.060088 |
| C | 9.931331 | -1.958484 | 11.290868 |
| C | 11.112562 | -1.176661 | 11.205551 |
| H | 11.960526 | -1.545582 | 10.623275 |
| C | 11.166546 | 0.050579 | 11.816779 |
| H | 12.047940 | 0.688090 | 11.722612 |
| C | 10.056870 | 0.496799 | 12.576961 |
| C | 10.102239 | 1.849654 | 13.240948 |

| | | | |
|---|---|---|---|
| H | 10.569572 | 2.573822 | 12.544772 |
| H | 10.801681 | 1.780845 | 14.088716 |
| C | 8.951177 | 3.073911 | 14.965557 |
| H | 9.683015 | 3.902772 | 14.884755 |
| H | 7.976814 | 3.510520 | 15.192581 |
| C | 9.387193 | 2.173956 | 16.120401 |
| H | 9.020879 | 2.594420 | 17.070427 |
| H | 10.485678 | 2.116332 | 16.178560 |
| C | 9.818165 | -0.166499 | 16.297864 |
| H | 10.490853 | -0.335941 | 15.437081 |
| H | 10.425897 | 0.129114 | 17.168176 |
| C | 9.085159 | -1.429049 | 16.650849 |
| H | 8.500805 | -1.286711 | 17.578763 |
| H | 9.826846 | -2.228078 | 16.818679 |
| C | 7.735467 | -3.123932 | 15.736815 |
| H | 8.471245 | -3.818766 | 15.299317 |
| H | 7.664292 | -3.347989 | 16.815149 |
| C | 8.069375 | 3.102919 | 12.715278 |
| H | 7.336534 | 3.716819 | 13.255292 |
| H | 8.738335 | 3.784279 | 12.153441 |
| N | 8.975027 | -0.232975 | 12.728728 |
| N | 8.812833 | 2.332484 | 13.716105 |
| O | 8.881767 | 0.844588 | 15.978185 |
| O | 8.208149 | -1.785343 | 15.598798 |
| O | 6.639490 | -1.483301 | 12.800869 |
| C | 7.452569 | -3.274626 | 11.452295 |
| H | 6.483293 | -3.777781 | 11.470811 |
| C | 8.526954 | -3.831357 | 10.728025 |
| H | 8.374450 | -4.783426 | 10.214171 |
| C | 9.749045 | -3.200525 | 10.633834 |
| H | 10.564691 | -3.629503 | 10.049517 |
| C | 6.072693 | 2.466374 | 16.454978 |
| C | 6.105827 | 3.743873 | 17.018006 |
| H | 6.205910 | 4.605159 | 16.353829 |
| C | 5.984594 | 3.934841 | 18.410087 |
| H | 6.022062 | 4.953622 | 18.803236 |
| C | 5.804249 | 2.881022 | 19.280810 |
| H | 5.694534 | 3.044889 | 20.354059 |
| Tb | 6.942652 | 0.152257 | 14.415454 |

Tb3+_Ion

SCF (RwB97XD) = -34.9986652250
E(SCF)+ZPE(0 K)= -34.998665
H(298 K)= -34.996305
G(298 K)= -35.015832

| | | | |
|---|---|---|---|
| Tb | 0.887400 | 3.945900 | 9.220400 |

Tb-syn-KHQ_Ligand(2–)

SCF (RwB97XD) = -1910.50037905
E(SCF)+ZPE(0 K)= -1909.832513
H(298 K)= -1909.797441
G(298 K)= -1909.894619
Lowest Frequency = -36.3692cm-1

| | | | |
|---|---|---|---|
| N | 3.082009 | 2.630046 | 9.268686 |
| C | 3.128966 | 1.327598 | 9.440420 |
| C | 4.275742 | 0.704893 | 9.982655 |
| H | 4.284175 | -0.374418 | 10.142172 |
| C | 5.369129 | 1.477310 | 10.301927 |
| H | 6.260762 | 1.015476 | 10.732776 |
| C | 5.367613 | 2.872698 | 10.038607 |
| C | 4.169246 | 3.401425 | 9.506946 |
| C | 4.020064 | 4.792451 | 9.165569 |
| O | 2.853115 | 5.193456 | 8.740779 |
| C | 5.137393 | 5.600642 | 9.353604 |
| H | 5.081462 | 6.655607 | 9.075360 |
| C | 6.332689 | 5.074046 | 9.895683 |
| H | 7.180411 | 5.748990 | 10.036129 |
| C | 6.464046 | 3.748438 | 10.248557 |
| H | 7.396884 | 3.363362 | 10.663595 |
| N | 1.963565 | 4.406659 | 11.534692 |
| C | 2.609819 | 5.502003 | 11.864416 |
| C | 3.593729 | 5.495408 | 12.879558 |
| H | 4.134137 | 6.413020 | 13.117254 |
| C | 3.857291 | 4.323359 | 13.549941 |
| H | 4.624393 | 4.294799 | 14.327540 |
| C | 3.110837 | 3.149569 | 13.264807 |
| C | 2.153926 | 3.260227 | 12.230883 |
| C | 1.302944 | 2.167338 | 11.845276 |
| O | 0.490253 | 2.360835 | 10.842287 |
| C | 1.445837 | 0.982299 | 12.559653 |
| H | 0.798103 | 0.135818 | 12.320137 |
| C | 2.412495 | 0.866728 | 13.585547 |
| H | 2.497563 | -0.085569 | 14.114485 |
| C | 3.242419 | 1.908308 | 13.939768 |
| H | 3.979160 | 1.797895 | 14.737146 |
| C | 1.909753 | 0.555359 | 8.995372 |
| H | 2.224025 | -0.424995 | 8.584412 |
| H | 1.276260 | 0.360082 | 9.871419 |
| N | 1.127745 | 1.313363 | 8.015567 |
| C | 1.859712 | 1.443384 | 6.755729 |
| H | 1.877687 | 0.489078 | 6.192325 |
| H | 2.899649 | 1.707115 | 6.984837 |
| C | 1.290745 | 2.521762 | 5.863482 |
| H | 1.967531 | 2.659255 | 5.002300 |
| H | 0.297545 | 2.257183 | 5.456921 |
| O | 1.190982 | 3.729439 | 6.589388 |
| C | 0.880342 | 4.831391 | 5.763852 |
| H | -0.143455 | 4.717815 | 5.362208 |
| H | 1.584314 | 4.882113 | 4.915014 |
| C | 0.997923 | 6.092590 | 6.571377 |
| H | 2.034079 | 6.234480 | 6.917664 |
| H | 0.682776 | 6.945358 | 5.948071 |
| O | 0.134214 | 5.967802 | 7.695185 |
| C | -0.219462 | 7.196290 | 8.307879 |
| H | -1.241043 | 7.074741 | 8.680348 |
| H | -0.254057 | 7.992186 | 7.546017 |
| C | 0.758877 | 7.608004 | 9.398774 |
| H | 1.740755 | 7.750389 | 8.926917 |
| H | 0.444224 | 8.593407 | 9.799804 |
| N | 0.927716 | 6.616391 | 10.456900 |

| | | | |
|---|---|---|---|
| C | 2.234755 | 6.751355 | 11.106429 |
| H | 2.260953 | 7.620744 | 11.792916 |
| H | 2.990261 | 6.921550 | 10.328879 |
| C | -0.125013 | 6.621112 | 11.471615 |
| H | -0.263055 | 7.629362 | 11.911946 |
| H | 0.198964 | 5.955472 | 12.281284 |
| C | -1.472828 | 6.131841 | 10.979338 |
| H | -2.092439 | 5.857598 | 11.850361 |
| H | -2.019921 | 6.917645 | 10.430377 |
| O | -1.294798 | 5.002430 | 10.144987 |
| C | -2.492171 | 4.515623 | 9.564221 |
| H | -2.603206 | 4.932179 | 8.546906 |
| H | -3.363118 | 4.825365 | 10.163369 |
| C | -2.442035 | 3.014794 | 9.536136 |
| H | -2.315342 | 2.632771 | 10.564211 |
| H | -3.388845 | 2.623098 | 9.122996 |
| O | -1.354144 | 2.590426 | 8.741530 |
| C | -1.219433 | 1.173915 | 8.804071 |
| H | -2.188773 | 0.707549 | 8.555834 |
| H | -0.936768 | 0.898358 | 9.831537 |
| C | -0.192066 | 0.723627 | 7.797918 |
| H | -0.147324 | -0.382711 | 7.802347 |
| H | -0.550218 | 1.015881 | 6.801989 |

Tb-syn-KHQ(1+)

SCF (RwB97XD) = -1947.32290624
E(SCF)+ZPE(0 K)= -1946.638977
H(298 K)= -1946.602395
G(298 K)= -1946.701882
Lowest Frequency = 39.1863cm-1

| | | | |
|---|---|---|---|
| Tb | 0.942653 | 3.967616 | 9.238382 |
| N | 3.082009 | 2.630046 | 9.268686 |
| C | 3.128966 | 1.327598 | 9.440420 |
| C | 4.275742 | 0.704893 | 9.982655 |
| H | 4.284175 | -0.374418 | 10.142172 |
| C | 5.369129 | 1.477310 | 10.301927 |
| H | 6.260762 | 1.015476 | 10.732776 |
| C | 5.367613 | 2.872698 | 10.038607 |
| C | 4.169246 | 3.401425 | 9.506946 |
| C | 4.020064 | 4.792451 | 9.165569 |
| O | 2.853115 | 5.193456 | 8.740779 |
| C | 5.137393 | 5.600642 | 9.353604 |
| H | 5.081462 | 6.655607 | 9.075360 |
| C | 6.332689 | 5.074046 | 9.895683 |
| H | 7.180411 | 5.748990 | 10.036129 |
| C | 6.464046 | 3.748438 | 10.248557 |
| H | 7.396884 | 3.363362 | 10.663595 |
| N | 1.963565 | 4.406659 | 11.534692 |
| C | 2.609819 | 5.502003 | 11.864416 |
| C | 3.593729 | 5.495408 | 12.879558 |
| H | 4.134137 | 6.413020 | 13.117254 |
| C | 3.857291 | 4.323359 | 13.549941 |
| H | 4.624393 | 4.294799 | 14.327540 |
| C | 3.110837 | 3.149569 | 13.264807 |
| C | 2.153926 | 3.260227 | 12.230883 |

| | | | |
|---|---|---|---|
| C | 1.302944 | 2.167338 | 11.845276 |
| O | 0.490253 | 2.360835 | 10.842287 |
| C | 1.445837 | 0.982299 | 12.559653 |
| H | 0.798103 | 0.135818 | 12.320137 |
| C | 2.412495 | 0.866728 | 13.585547 |
| H | 2.497563 | -0.085569 | 14.114485 |
| C | 3.242419 | 1.908308 | 13.939768 |
| H | 3.979160 | 1.797895 | 14.737146 |
| C | 1.909753 | 0.555359 | 8.995372 |
| H | 2.224025 | -0.424995 | 8.584412 |
| H | 1.276260 | 0.360082 | 9.871419 |
| N | 1.127745 | 1.313363 | 8.015567 |
| C | 1.859712 | 1.443384 | 6.755729 |
| H | 1.877687 | 0.489078 | 6.192325 |
| H | 2.899649 | 1.707115 | 6.984837 |
| C | 1.290745 | 2.521762 | 5.863482 |
| H | 1.967531 | 2.659255 | 5.002300 |
| H | 0.297545 | 2.257183 | 5.456921 |
| O | 1.190982 | 3.729439 | 6.589388 |
| C | 0.880342 | 4.831391 | 5.763852 |
| H | -0.143455 | 4.717815 | 5.362208 |
| H | 1.584314 | 4.882113 | 4.915014 |
| C | 0.997923 | 6.092590 | 6.571377 |
| H | 2.034079 | 6.234480 | 6.917664 |
| H | 0.682776 | 6.945358 | 5.948071 |
| O | 0.134214 | 5.967802 | 7.695185 |
| C | -0.219462 | 7.196290 | 8.307879 |
| H | -1.241043 | 7.074741 | 8.680348 |
| H | -0.254057 | 7.992186 | 7.546017 |
| C | 0.758877 | 7.608004 | 9.398774 |
| H | 1.740755 | 7.750389 | 8.926917 |
| H | 0.444224 | 8.593407 | 9.799804 |
| N | 0.927716 | 6.616391 | 10.456900 |
| C | 2.234755 | 6.751355 | 11.106429 |
| H | 2.260953 | 7.620744 | 11.792916 |
| H | 2.990261 | 6.921550 | 10.328879 |
| C | -0.125013 | 6.621112 | 11.471615 |
| H | -0.263055 | 7.629362 | 11.911946 |
| H | 0.198964 | 5.955472 | 12.281284 |
| C | -1.472828 | 6.131841 | 10.979338 |
| H | -2.092439 | 5.857598 | 11.850361 |
| H | -2.019921 | 6.917645 | 10.430377 |
| O | -1.294798 | 5.002430 | 10.144987 |
| C | -2.492171 | 4.515623 | 9.564221 |
| H | -2.603206 | 4.932179 | 8.546906 |
| H | -3.363118 | 4.825365 | 10.163369 |
| C | -2.442035 | 3.014794 | 9.536136 |
| H | -2.315342 | 2.632771 | 10.564211 |
| H | -3.388845 | 2.623098 | 9.122996 |
| O | -1.354144 | 2.590426 | 8.741530 |
| C | -1.219433 | 1.173915 | 8.804071 |
| H | -2.188773 | 0.707549 | 8.555834 |
| H | -0.936768 | 0.898358 | 9.831537 |
| C | -0.192066 | 0.723627 | 7.797918 |
| H | -0.147324 | -0.382711 | 7.802347 |
| H | -0.550218 | 1.015881 | 6.801989 |

Tb-syn-H2O-KHQ(1+)

SCF (RwB97XD) = -2023.68386261
E(SCF)+ZPE(0 K)= -2022.974169
H(298 K)= -2022.935391
G(298 K)= -2023.039301
Lowest Frequency = 21.7781cm-1

| | | | |
|---|---|---|---|
| Tb | 1.002431 | 4.020350 | 9.088277 |
| N | 3.092913 | 2.617820 | 9.133590 |
| C | 3.107400 | 1.306076 | 9.220508 |
| C | 4.247345 | 0.616013 | 9.692626 |
| H | 4.230769 | -0.472682 | 9.764687 |
| C | 5.362197 | 1.332825 | 10.058926 |
| H | 6.248556 | 0.817843 | 10.437231 |
| C | 5.383375 | 2.745610 | 9.926215 |
| C | 4.196489 | 3.343564 | 9.440725 |
| C | 4.083325 | 4.763904 | 9.233075 |
| O | 2.941005 | 5.232659 | 8.810192 |
| C | 5.206622 | 5.530581 | 9.526710 |
| H | 5.168763 | 6.609611 | 9.360050 |
| C | 6.387423 | 4.932303 | 10.022356 |
| H | 7.242934 | 5.574754 | 10.244003 |
| C | 6.493702 | 3.574587 | 10.229757 |
| H | 7.414895 | 3.130864 | 10.610861 |
| N | 1.839918 | 4.489897 | 11.485464 |
| C | 2.370051 | 5.618967 | 11.895742 |
| C | 3.182408 | 5.680568 | 13.052453 |
| H | 3.615374 | 6.634061 | 13.359545 |
| C | 3.418970 | 4.531808 | 13.768910 |
| H | 4.053908 | 4.555356 | 14.657854 |
| C | 2.816687 | 3.309048 | 13.374088 |
| C | 2.016459 | 3.357294 | 12.209041 |
| C | 1.313825 | 2.204537 | 11.717079 |
| O | 0.610801 | 2.346610 | 10.630421 |
| C | 1.453677 | 1.023974 | 12.440577 |
| H | 0.918826 | 0.129926 | 12.110887 |
| C | 2.268949 | 0.973147 | 13.594452 |
| H | 2.356838 | 0.024413 | 14.129202 |
| C | 2.946486 | 2.076416 | 14.064939 |
| H | 3.566847 | 2.019042 | 14.960840 |
| C | 1.855072 | 0.576682 | 8.800848 |
| H | 2.131473 | -0.384535 | 8.322757 |
| H | 1.284928 | 0.342740 | 9.707723 |
| N | 1.015659 | 1.379342 | 7.903871 |
| C | 1.691890 | 1.486924 | 6.601794 |
| H | 1.539445 | 0.567475 | 6.003250 |
| H | 2.771175 | 1.579537 | 6.779121 |
| C | 1.269119 | 2.677364 | 5.777985 |
| H | 1.798419 | 2.634394 | 4.809702 |
| H | 0.183925 | 2.675135 | 5.570233 |
| O | 1.610246 | 3.869092 | 6.454102 |
| C | 1.447000 | 5.002163 | 5.629371 |
| H | 0.476004 | 4.938296 | 5.103366 |
| H | 2.246553 | 5.034598 | 4.867873 |
| C | 1.490427 | 6.264711 | 6.447553 |
| H | 2.446409 | 6.341383 | 6.988181 |

| | | | |
|---|---|---|---|
| H | 1.365014 | 7.126062 | 5.769019 |
| O | 0.412273 | 6.220745 | 7.375512 |
| C | 0.077725 | 7.451700 | 7.979496 |
| H | -0.989937 | 7.399541 | 8.230766 |
| H | 0.192091 | 8.275477 | 7.253909 |
| C | 0.934731 | 7.743368 | 9.194934 |
| H | 1.976407 | 7.808957 | 8.853454 |
| H | 0.660330 | 8.739933 | 9.596873 |
| N | 0.888318 | 6.714874 | 10.236704 |
| C | 2.101294 | 6.837378 | 11.056840 |
| H | 2.056191 | 7.735768 | 11.703586 |
| H | 2.955148 | 6.960388 | 10.378177 |
| C | -0.294815 | 6.789436 | 11.099571 |
| H | -0.558143 | 7.842740 | 11.323458 |
| H | -0.040121 | 6.313562 | 12.056412 |
| C | -1.545559 | 6.094089 | 10.598192 |
| H | -2.316046 | 6.215130 | 11.379666 |
| H | -1.955978 | 6.549650 | 9.680325 |
| O | -1.307058 | 4.716734 | 10.380577 |
| C | -2.507688 | 3.961237 | 10.417345 |
| H | -3.172303 | 4.279349 | 9.590131 |
| H | -3.037609 | 4.169111 | 11.363105 |
| C | -2.256723 | 2.476054 | 10.344291 |
| H | -1.549057 | 2.175111 | 11.131331 |
| H | -3.222763 | 1.959185 | 10.492302 |
| O | -1.715157 | 2.159162 | 9.082867 |
| C | -1.245453 | 0.836302 | 8.924950 |
| H | -2.098678 | 0.159212 | 8.739118 |
| H | -0.744677 | 0.523344 | 9.852329 |
| C | -0.305215 | 0.757670 | 7.734749 |
| H | -0.188586 | -0.312860 | 7.469789 |
| H | -0.802918 | 1.235104 | 6.877686 |
| O | -0.953117 | 3.955935 | 7.480708 |
| H | -1.631162 | 3.370277 | 7.867536 |
| H | -1.323364 | 4.786421 | 7.168012 |

Tm-anti-KHQ-Ligand(2–)

SCF (RwB97XD) = -1910.47850265
E(SCF)+ZPE(0 K)= -1909.812747
H(298 K)= -1909.778336
G(298 K)= -1909.876253
Lowest Frequency = -98.6645cm-1

| | | | |
|---|---|---|---|
| C | 5.812841 | 1.411676 | 17.306359 |
| C | 5.474877 | 1.670067 | 18.658882 |
| C | 5.172234 | 0.538118 | 19.459976 |
| H | 4.903947 | 0.678735 | 20.509936 |
| C | 5.176064 | -0.715359 | 18.902091 |
| H | 4.898106 | -1.593517 | 19.488326 |
| C | 5.539287 | -0.868106 | 17.540838 |
| C | 5.528252 | -2.235988 | 16.907980 |
| H | 6.389457 | -2.796182 | 17.305380 |
| H | 4.637668 | -2.790028 | 17.265176 |
| C | 4.284472 | -2.132485 | 14.802705 |
| H | 3.537501 | -2.773934 | 15.310792 |
| H | 4.407958 | -2.502144 | 13.776773 |

| | | | |
|---|---|---|---|
| C | 3.720544 | -0.724344 | 14.691946 |
| H | 2.754296 | -0.781857 | 14.164672 |
| H | 3.545858 | -0.251587 | 15.672195 |
| C | 4.132557 | 1.061905 | 13.070117 |
| H | 4.034161 | 2.009366 | 13.624490 |
| H | 3.151464 | 0.765455 | 12.664647 |
| C | 5.119823 | 1.211041 | 11.927981 |
| H | 5.163077 | 0.286927 | 11.328986 |
| H | 4.809529 | 2.053499 | 11.288607 |
| C | 7.336972 | 2.235873 | 11.768540 |
| H | 6.809757 | 2.922514 | 11.086276 |
| H | 7.971311 | 1.571280 | 11.159590 |
| C | 6.382891 | -3.334942 | 14.946899 |
| H | 6.265203 | -3.353751 | 13.862115 |
| H | 6.033266 | -4.310501 | 15.340387 |
| N | 5.890678 | 0.152753 | 16.792988 |
| N | 5.594014 | -2.218351 | 15.454329 |
| O | 6.296859 | 2.174993 | 15.136861 |
| O | 4.626121 | 0.055808 | 13.934447 |
| O | 6.391599 | 1.470642 | 12.491843 |
| C | 7.557767 | -2.037314 | 12.139855 |
| C | 8.857019 | -1.422828 | 12.078286 |
| C | 9.903566 | -1.979667 | 11.300571 |
| C | 11.103783 | -1.225544 | 11.227441 |
| H | 11.943390 | -1.606523 | 10.640858 |
| C | 11.185900 | -0.008266 | 11.855131 |
| H | 12.082039 | 0.609688 | 11.770374 |
| C | 10.085261 | 0.455042 | 12.618201 |
| C | 10.152767 | 1.799581 | 13.296088 |
| H | 10.639386 | 2.522533 | 12.611799 |
| H | 10.841876 | 1.710155 | 14.150835 |
| C | 9.003875 | 3.019940 | 15.020908 |
| H | 9.768181 | 3.820787 | 14.963848 |
| H | 8.043690 | 3.492876 | 15.233666 |
| C | 9.380785 | 2.089959 | 16.176949 |
| H | 8.970784 | 2.488384 | 17.118291 |
| H | 10.474949 | 2.028548 | 16.286820 |
| C | 9.820816 | -0.252099 | 16.288094 |
| H | 10.539261 | -0.354324 | 15.454326 |
| H | 10.376939 | -0.004369 | 17.206711 |
| C | 9.095335 | -1.549368 | 16.513802 |
| H | 8.481793 | -1.491570 | 17.431423 |
| H | 9.846150 | -2.346320 | 16.639249 |
| C | 7.866209 | -3.190377 | 15.295706 |
| H | 8.476120 | -3.675302 | 14.517282 |
| H | 8.091052 | -3.683718 | 16.254458 |
| C | 8.131509 | 3.070824 | 12.760813 |
| H | 7.405321 | 3.692350 | 13.299983 |
| H | 8.806638 | 3.743860 | 12.196162 |
| N | 8.987395 | -0.251632 | 12.760673 |
| N | 8.866568 | 2.294953 | 13.763157 |
| O | 8.883324 | 0.763051 | 15.984812 |
| O | 8.262554 | -1.820626 | 15.402906 |
| O | 6.633220 | -1.453215 | 12.836768 |
| C | 7.389173 | -3.232089 | 11.438352 |
| H | 6.405961 | -3.707734 | 11.443671 |
| C | 8.449011 | -3.803122 | 10.703485 |

| | | | |
|---|---|---|---|
| H | 8.270667 | -4.739859 | 10.170096 |
| C | 9.688721 | -3.205228 | 10.622635 |
| H | 10.492714 | -3.645874 | 10.030917 |
| C | 6.031978 | 2.480798 | 16.369004 |
| C | 5.944764 | 3.783152 | 16.863415 |
| H | 6.079177 | 4.616136 | 16.169835 |
| C | 5.652488 | 4.030958 | 18.220802 |
| H | 5.594605 | 5.067047 | 18.562785 |
| C | 5.418332 | 3.009731 | 19.116979 |
| H | 5.169672 | 3.216454 | 20.159161 |

Tm-anti-KHQ(1+)

SCF (RwB97XD) = -1949.64330447
E(SCF)+ZPE(0 K)= -1948.960766
H(298 K)= -1948.923478
G(298 K)= -1949.026583
Lowest Frequency = 21.1575cm-1

| | | | |
|---|---|---|---|
| C | 5.812841 | 1.411676 | 17.306359 |
| C | 5.474877 | 1.670067 | 18.658882 |
| C | 5.172234 | 0.538118 | 19.459976 |
| H | 4.903947 | 0.678735 | 20.509936 |
| C | 5.176064 | -0.715359 | 18.902091 |
| H | 4.898106 | -1.593517 | 19.488326 |
| C | 5.539287 | -0.868106 | 17.540838 |
| C | 5.528252 | -2.235988 | 16.907980 |
| H | 6.389457 | -2.796182 | 17.305380 |
| H | 4.637668 | -2.790028 | 17.265176 |
| C | 4.284472 | -2.132485 | 14.802705 |
| H | 3.537501 | -2.773934 | 15.310792 |
| H | 4.407958 | -2.502144 | 13.776773 |
| C | 3.720544 | -0.724344 | 14.691946 |
| H | 2.754296 | -0.781857 | 14.164672 |
| H | 3.545858 | -0.251587 | 15.672195 |
| C | 4.132557 | 1.061905 | 13.070117 |
| H | 4.034161 | 2.009366 | 13.624490 |
| H | 3.151464 | 0.765455 | 12.664647 |
| C | 5.119823 | 1.211041 | 11.927981 |
| H | 5.163077 | 0.286927 | 11.328986 |
| H | 4.809529 | 2.053499 | 11.288607 |
| C | 7.336972 | 2.235873 | 11.768540 |
| H | 6.809757 | 2.922514 | 11.086276 |
| H | 7.971311 | 1.571280 | 11.159590 |
| C | 6.382891 | -3.334942 | 14.946899 |
| H | 6.265203 | -3.353751 | 13.862115 |
| H | 6.033266 | -4.310501 | 15.340387 |
| N | 5.890678 | 0.152753 | 16.792988 |
| N | 5.594014 | -2.218351 | 15.454329 |
| O | 6.296859 | 2.174993 | 15.136861 |
| O | 4.626121 | 0.055808 | 13.934447 |
| O | 6.391599 | 1.470642 | 12.491843 |
| C | 7.557767 | -2.037314 | 12.139855 |
| C | 8.857019 | -1.422828 | 12.078286 |
| C | 9.903566 | -1.979667 | 11.300571 |
| C | 11.103783 | -1.225544 | 11.227441 |
| H | 11.943390 | -1.606523 | 10.640858 |

| | | | |
|---|---|---|---|
| C | 11.185900 | -0.008266 | 11.855131 |
| H | 12.082039 | 0.609688 | 11.770374 |
| C | 10.085261 | 0.455042 | 12.618201 |
| C | 10.152767 | 1.799581 | 13.296088 |
| H | 10.639386 | 2.522533 | 12.611799 |
| H | 10.841876 | 1.710155 | 14.150835 |
| C | 9.003875 | 3.019940 | 15.020908 |
| H | 9.768181 | 3.820787 | 14.963848 |
| H | 8.043690 | 3.492876 | 15.233666 |
| C | 9.380785 | 2.089959 | 16.176949 |
| H | 8.970784 | 2.488384 | 17.118291 |
| H | 10.474949 | 2.028548 | 16.286820 |
| C | 9.820816 | -0.252099 | 16.288094 |
| H | 10.539261 | -0.354324 | 15.454326 |
| H | 10.376939 | -0.004369 | 17.206711 |
| C | 9.095335 | -1.549368 | 16.513802 |
| H | 8.481793 | -1.491570 | 17.431423 |
| H | 9.846150 | -2.346320 | 16.639249 |
| C | 7.866209 | -3.190377 | 15.295706 |
| H | 8.476120 | -3.675302 | 14.517282 |
| H | 8.091052 | -3.683718 | 16.254458 |
| C | 8.131509 | 3.070824 | 12.760813 |
| H | 7.405321 | 3.692350 | 13.299983 |
| H | 8.806638 | 3.743860 | 12.196162 |
| N | 8.987395 | -0.251632 | 12.760673 |
| N | 8.866568 | 2.294953 | 13.763157 |
| O | 8.883324 | 0.763051 | 15.984812 |
| O | 8.262554 | -1.820626 | 15.402906 |
| O | 6.633220 | -1.453215 | 12.836768 |
| C | 7.389173 | -3.232089 | 11.438352 |
| H | 6.405961 | -3.707734 | 11.443671 |
| C | 8.449011 | -3.803122 | 10.703485 |
| H | 8.270667 | -4.739859 | 10.170096 |
| C | 9.688721 | -3.205228 | 10.622635 |
| H | 10.492714 | -3.645874 | 10.030917 |
| C | 6.031978 | 2.480798 | 16.369004 |
| C | 5.944764 | 3.783152 | 16.863415 |
| H | 6.079177 | 4.616136 | 16.169835 |
| C | 5.652488 | 4.030958 | 18.220802 |
| H | 5.594605 | 5.067047 | 18.562785 |
| C | 5.418332 | 3.009731 | 19.116979 |
| H | 5.169672 | 3.216454 | 20.159161 |
| Tm | 6.997165 | 0.135760 | 14.418995 |

Tm3+_Ion

SCF (RwB97XD) = -37.2924025798
E(SCF)+ZPE(0 K)= -37.292403
H(298 K)= -37.290042
G(298 K)= -37.309656

| | | | |
|---|---|---|---|
| Tm | 0.887400 | 3.945900 | 9.220400 |

Tm-syn-KHQ_Ligand(2–)

SCF (RwB97XD) = -1910.49371273
E(SCF)+ZPE(0 K)= -1909.825592

H(298 K)= -1909.790583
G(298 K)= -1909.887883
Lowest Frequency = -38.9870cm-1

| | | | |
|---|---|---|---|
| N | 3.065973 | 2.635677 | 9.314922 |
| C | 3.128018 | 1.336781 | 9.511663 |
| C | 4.286501 | 0.736069 | 10.053366 |
| H | 4.305098 | -0.339561 | 10.235203 |
| C | 5.379185 | 1.522480 | 10.339487 |
| H | 6.280131 | 1.076706 | 10.767876 |
| C | 5.364472 | 2.910013 | 10.039343 |
| C | 4.153782 | 3.416652 | 9.515546 |
| C | 3.990221 | 4.794448 | 9.135505 |
| O | 2.810887 | 5.171569 | 8.722994 |
| C | 5.105110 | 5.614758 | 9.274476 |
| H | 5.037984 | 6.659908 | 8.963665 |
| C | 6.313680 | 5.111320 | 9.810164 |
| H | 7.160166 | 5.794526 | 9.912804 |
| C | 6.459215 | 3.798267 | 10.201850 |
| H | 7.402176 | 3.431311 | 10.610346 |
| N | 1.976011 | 4.425462 | 11.509260 |
| C | 2.629446 | 5.517453 | 11.836990 |
| C | 3.600265 | 5.510508 | 12.864441 |
| H | 4.148201 | 6.424720 | 13.097949 |
| C | 3.838482 | 4.344811 | 13.555559 |
| H | 4.594037 | 4.317087 | 14.344417 |
| C | 3.075258 | 3.179840 | 13.279675 |
| C | 2.137669 | 3.290116 | 12.229073 |
| C | 1.267249 | 2.211751 | 11.851985 |
| O | 0.477609 | 2.410589 | 10.831442 |
| C | 1.367235 | 1.038894 | 12.591958 |
| H | 0.701216 | 0.204205 | 12.361517 |
| C | 2.316578 | 0.921138 | 13.634465 |
| H | 2.369871 | -0.021620 | 14.184181 |
| C | 3.167070 | 1.948829 | 13.980090 |
| H | 3.888473 | 1.836489 | 14.791103 |
| C | 1.918166 | 0.543696 | 9.079871 |
| H | 2.244184 | -0.445192 | 8.699241 |
| H | 1.275614 | 0.366448 | 9.952867 |
| N | 1.146975 | 1.273014 | 8.073685 |
| C | 1.890869 | 1.384347 | 6.821396 |
| H | 1.916634 | 0.423377 | 6.269506 |
| H | 2.928359 | 1.653932 | 7.054437 |
| C | 1.323527 | 2.449967 | 5.914544 |
| H | 2.008154 | 2.588738 | 5.059733 |
| H | 0.337815 | 2.171900 | 5.498844 |
| O | 1.201977 | 3.662748 | 6.628554 |
| C | 0.855856 | 4.739318 | 5.784886 |
| H | -0.175269 | 4.600754 | 5.410437 |
| H | 1.538367 | 4.779502 | 4.918149 |
| C | 0.972985 | 6.020950 | 6.556660 |
| H | 2.014281 | 6.189148 | 6.872946 |
| H | 0.627917 | 6.851667 | 5.919753 |
| O | 0.140086 | 5.911897 | 7.704345 |
| C | -0.211733 | 7.156088 | 8.287283 |
| H | -1.232281 | 7.048499 | 8.666488 |
| H | -0.248306 | 7.931175 | 7.504789 |

| | | | |
|---|---|---|---|
| C | 0.771862 | 7.595147 | 9.361812 |
| H | 1.749686 | 7.730913 | 8.879942 |
| H | 0.455245 | 8.587389 | 9.744466 |
| N | 0.950768 | 6.624661 | 10.434926 |
| C | 2.260576 | 6.764344 | 11.072874 |
| H | 2.293844 | 7.636585 | 11.755551 |
| H | 3.012309 | 6.929791 | 10.290941 |
| C | -0.097174 | 6.633290 | 11.452268 |
| H | -0.246187 | 7.645361 | 11.880322 |
| H | 0.234331 | 5.982120 | 12.270301 |
| C | -1.438691 | 6.123248 | 10.963249 |
| H | -2.050925 | 5.826855 | 11.832061 |
| H | -2.000076 | 6.906779 | 10.425819 |
| O | -1.246798 | 5.008805 | 10.111025 |
| C | -2.432325 | 4.556735 | 9.480012 |
| H | -2.482545 | 4.970956 | 8.456968 |
| H | -3.319170 | 4.896300 | 10.037698 |
| C | -2.425440 | 3.056457 | 9.458604 |
| H | -2.361817 | 2.676505 | 10.493203 |
| H | -3.361076 | 2.688787 | 9.000264 |
| O | -1.311397 | 2.599807 | 8.721578 |
| C | -1.208004 | 1.183071 | 8.825784 |
| H | -2.181950 | 0.730467 | 8.570510 |
| H | -0.952656 | 0.931493 | 9.866264 |
| C | -0.170348 | 0.682480 | 7.855775 |
| H | -0.135211 | -0.423063 | 7.906260 |
| H | -0.509502 | 0.935385 | 6.842680 |

Tm-syn-KHQ(1+)

SCF (RwB97XD) = -1949.65187867
E(SCF)+ZPE(0 K)= -1948.967705
H(298 K)= -1948.931128
G(298 K)= -1949.030731
Lowest Frequency = 35.7590cm-1

| | | | |
|---|---|---|---|
| Tm | 0.966323 | 3.958461 | 9.255314 |
| N | 3.065973 | 2.635677 | 9.314922 |
| C | 3.128018 | 1.336781 | 9.511663 |
| C | 4.286501 | 0.736069 | 10.053366 |
| H | 4.305098 | -0.339561 | 10.235203 |
| C | 5.379185 | 1.522480 | 10.339487 |
| H | 6.280131 | 1.076706 | 10.767876 |
| C | 5.364472 | 2.910013 | 10.039343 |
| C | 4.153782 | 3.416652 | 9.515546 |
| C | 3.990221 | 4.794448 | 9.135505 |
| O | 2.810887 | 5.171569 | 8.722994 |
| C | 5.105110 | 5.614758 | 9.274476 |
| H | 5.037984 | 6.659908 | 8.963665 |
| C | 6.313680 | 5.111320 | 9.810164 |
| H | 7.160166 | 5.794526 | 9.912804 |
| C | 6.459215 | 3.798267 | 10.201850 |
| H | 7.402176 | 3.431311 | 10.610346 |
| N | 1.976011 | 4.425462 | 11.509260 |
| C | 2.629446 | 5.517453 | 11.836990 |
| C | 3.600265 | 5.510508 | 12.864441 |
| H | 4.148201 | 6.424720 | 13.097949 |

| | | | |
|---|---|---|---|
| C | 3.838482 | 4.344811 | 13.555559 |
| H | 4.594037 | 4.317087 | 14.344417 |
| C | 3.075258 | 3.179840 | 13.279675 |
| C | 2.137669 | 3.290116 | 12.229073 |
| C | 1.267249 | 2.211751 | 11.851985 |
| O | 0.477609 | 2.410589 | 10.831442 |
| C | 1.367235 | 1.038894 | 12.591958 |
| H | 0.701216 | 0.204205 | 12.361517 |
| C | 2.316578 | 0.921138 | 13.634465 |
| H | 2.369871 | -0.021620 | 14.184181 |
| C | 3.167070 | 1.948829 | 13.980090 |
| H | 3.888473 | 1.836489 | 14.791103 |
| C | 1.918166 | 0.543696 | 9.079871 |
| H | 2.244184 | -0.445192 | 8.699241 |
| H | 1.275614 | 0.366448 | 9.952867 |
| N | 1.146975 | 1.273014 | 8.073685 |
| C | 1.890869 | 1.384347 | 6.821396 |
| H | 1.916634 | 0.423377 | 6.269506 |
| H | 2.928359 | 1.653932 | 7.054437 |
| C | 1.323527 | 2.449967 | 5.914544 |
| H | 2.008154 | 2.588738 | 5.059733 |
| H | 0.337815 | 2.171900 | 5.498844 |
| O | 1.201977 | 3.662748 | 6.628554 |
| C | 0.855856 | 4.739318 | 5.784886 |
| H | -0.175269 | 4.600754 | 5.410437 |
| H | 1.538367 | 4.779502 | 4.918149 |
| C | 0.972985 | 6.020950 | 6.556660 |
| H | 2.014281 | 6.189148 | 6.872946 |
| H | 0.627917 | 6.851667 | 5.919753 |
| O | 0.140086 | 5.911897 | 7.704345 |
| C | -0.211733 | 7.156088 | 8.287283 |
| H | -1.232281 | 7.048499 | 8.666488 |
| H | -0.248306 | 7.931175 | 7.504789 |
| C | 0.771862 | 7.595147 | 9.361812 |
| H | 1.749686 | 7.730913 | 8.879942 |
| H | 0.455245 | 8.587389 | 9.744466 |
| N | 0.950768 | 6.624661 | 10.434926 |
| C | 2.260576 | 6.764344 | 11.072874 |
| H | 2.293844 | 7.636585 | 11.755551 |
| H | 3.012309 | 6.929791 | 10.290941 |
| C | -0.097174 | 6.633290 | 11.452268 |
| H | -0.246187 | 7.645361 | 11.880322 |
| H | 0.234331 | 5.982120 | 12.270301 |
| C | -1.438691 | 6.123248 | 10.963249 |
| H | -2.050925 | 5.826855 | 11.832061 |
| H | -2.000076 | 6.906779 | 10.425819 |
| O | -1.246798 | 5.008805 | 10.111025 |
| C | -2.432325 | 4.556735 | 9.480012 |
| H | -2.482545 | 4.970956 | 8.456968 |
| H | -3.319170 | 4.896300 | 10.037698 |
| C | -2.425440 | 3.056457 | 9.458604 |
| H | -2.361817 | 2.676505 | 10.493203 |
| H | -3.361076 | 2.688787 | 9.000264 |
| O | -1.311397 | 2.599807 | 8.721578 |
| C | -1.208004 | 1.183071 | 8.825784 |
| H | -2.181950 | 0.730467 | 8.570510 |
| H | -0.952656 | 0.931493 | 9.866264 |

C -0.170348 0.682480 7.855775
H -0.135211 -0.423063 7.906260
H -0.509502 0.935385 6.842680

Tm-syn-H2O-KHQ(1+)

SCF (RwB97XD) = -2026.01309233
E(SCF)+ZPE(0 K)= -2025.303178
H(298 K)= -2025.264507
G(298 K)= -2025.368266
Lowest Frequency = 23.4511cm-1

Tm 1.067016 4.021124 9.107353
N 3.099698 2.629457 9.128138
C 3.119059 1.316443 9.205935
C 4.271994 0.627220 9.646406
H 4.259274 -0.461984 9.711093
C 5.395483 1.343214 9.987836
H 6.291960 0.827106 10.339864
C 5.411723 2.756248 9.859687
C 4.211566 3.353549 9.408791
C 4.091602 4.772381 9.203146
O 2.936157 5.234358 8.807073
C 5.219367 5.542058 9.466189
H 5.175353 6.620874 9.300078
C 6.414412 4.944991 9.929358
H 7.274736 5.588570 10.127634
C 6.528618 3.587362 10.132683
H 7.461043 3.145138 10.487243
N 1.852788 4.488863 11.464917
C 2.369446 5.617006 11.895620
C 3.135304 5.675306 13.083465
H 3.559427 6.627319 13.406972
C 3.334541 4.526938 13.812787
H 3.931851 4.549546 14.727464
C 2.736554 3.308445 13.399226
C 1.988345 3.359750 12.201131
C 1.284481 2.218385 11.688539
O 0.625846 2.373776 10.575854
C 1.370985 1.040469 12.423258
H 0.832540 0.153790 12.079824
C 2.138143 0.983794 13.610329
H 2.186225 0.037655 14.154557
C 2.815532 2.077791 14.101919
H 3.395963 2.015181 15.023823
C 1.863974 0.584667 8.799393
H 2.140226 -0.375757 8.319260
H 1.301903 0.347014 9.709957
N 1.018330 1.386356 7.909725
C 1.686658 1.497398 6.604792
H 1.523895 0.583594 6.000176
H 2.768153 1.580720 6.773523
C 1.266606 2.696445 5.793747
H 1.781463 2.652578 4.817705
H 0.178520 2.705355 5.601742
O 1.628591 3.882763 6.468735
C 1.459350 5.013529 5.641729

| | | | |
|---|---|---|---|
| H | 0.489853 | 4.941544 | 5.113790 |
| H | 2.259456 | 5.049605 | 4.880924 |
| C | 1.488115 | 6.279958 | 6.452534 |
| H | 2.440239 | 6.366816 | 6.997902 |
| H | 1.362146 | 7.136156 | 5.767271 |
| O | 0.404199 | 6.234761 | 7.372240 |
| C | 0.082650 | 7.464547 | 7.984172 |
| H | -0.982806 | 7.419160 | 8.245586 |
| H | 0.195931 | 8.291190 | 7.261475 |
| C | 0.953342 | 7.743542 | 9.192174 |
| H | 1.992927 | 7.801645 | 8.843115 |
| H | 0.691121 | 8.740379 | 9.601586 |
| N | 0.905874 | 6.710947 | 10.229177 |
| C | 2.116690 | 6.834855 | 11.052140 |
| H | 2.071628 | 7.734766 | 11.696555 |
| H | 2.973423 | 6.953366 | 10.376412 |
| C | -0.280622 | 6.784440 | 11.088366 |
| H | -0.557479 | 7.838082 | 11.292710 |
| H | -0.024206 | 6.329776 | 12.055056 |
| C | -1.520615 | 6.063855 | 10.596763 |
| H | -2.299843 | 6.204854 | 11.366596 |
| H | -1.924629 | 6.489157 | 9.661327 |
| O | -1.271449 | 4.683736 | 10.423227 |
| C | -2.474837 | 3.932650 | 10.460645 |
| H | -3.141807 | 4.257519 | 9.637682 |
| H | -3.000224 | 4.141653 | 11.409044 |
| C | -2.246260 | 2.443738 | 10.379641 |
| H | -1.514025 | 2.135143 | 11.140114 |
| H | -3.213309 | 1.943028 | 10.569927 |
| O | -1.766349 | 2.111426 | 9.097160 |
| C | -1.241780 | 0.809658 | 8.937159 |
| H | -2.068026 | 0.099705 | 8.753359 |
| H | -0.727119 | 0.514589 | 9.862530 |
| C | -0.301305 | 0.760068 | 7.743883 |
| H | -0.177334 | -0.306013 | 7.463797 |
| H | -0.807502 | 1.243136 | 6.895027 |
| O | -0.857539 | 3.929837 | 7.602395 |
| H | -1.536264 | 3.333685 | 7.981556 |
| H | -1.221664 | 4.759449 | 7.279042 |

Yb-anti-KHQ-Ligand(2–)

SCF (RwB97XD) = -1910.47711962
E(SCF)+ZPE(0 K)= -1909.810950
H(298 K)= -1909.776767
G(298 K)= -1909.873267
Lowest Frequency = -68.0613cm-1

| | | | |
|---|---|---|---|
| C | 5.821005 | 1.407045 | 17.300756 |
| C | 5.486098 | 1.666717 | 18.653693 |
| C | 5.187407 | 0.535342 | 19.457056 |
| H | 4.922389 | 0.676639 | 20.507749 |
| C | 5.190126 | -0.718430 | 18.899960 |
| H | 4.914219 | -1.596342 | 19.487533 |
| C | 5.548413 | -0.872179 | 17.537477 |
| C | 5.531186 | -2.240067 | 16.904891 |
| H | 6.392422 | -2.803188 | 17.298218 |

| | | | |
|---|---|---|---|
| H | 4.640463 | -2.791328 | 17.266202 |
| C | 4.280018 | -2.129636 | 14.804291 |
| H | 3.531109 | -2.767861 | 15.313731 |
| H | 4.398573 | -2.498884 | 13.777628 |
| C | 3.723051 | -0.718467 | 14.697161 |
| H | 2.756019 | -0.769547 | 14.170836 |
| H | 3.551798 | -0.247257 | 15.678693 |
| C | 4.139555 | 1.061175 | 13.069915 |
| H | 4.040425 | 2.011691 | 13.618827 |
| H | 3.159074 | 0.762611 | 12.664658 |
| C | 5.128442 | 1.203700 | 11.928520 |
| H | 5.172057 | 0.276583 | 11.334280 |
| H | 4.820037 | 2.043392 | 11.284695 |
| C | 7.344914 | 2.228225 | 11.767618 |
| H | 6.817288 | 2.910482 | 11.081369 |
| H | 7.980970 | 1.561680 | 11.162682 |
| C | 6.378360 | -3.335476 | 14.939139 |
| H | 6.252948 | -3.355980 | 13.855329 |
| H | 6.034933 | -4.311986 | 15.335744 |
| N | 5.898289 | 0.147919 | 16.787803 |
| N | 5.590526 | -2.221395 | 15.451617 |
| O | 6.301299 | 2.167239 | 15.130092 |
| O | 4.631457 | 0.059957 | 13.940918 |
| O | 6.399625 | 1.464783 | 12.493210 |
| C | 7.553486 | -2.029930 | 12.146617 |
| C | 8.855035 | -1.420628 | 12.089048 |
| C | 9.901150 | -1.980514 | 11.313159 |
| C | 11.104487 | -1.231029 | 11.244044 |
| H | 11.944420 | -1.614685 | 10.659688 |
| C | 11.188696 | -0.014179 | 11.872187 |
| H | 12.086915 | 0.601072 | 11.789841 |
| C | 10.087322 | 0.453157 | 12.631783 |
| C | 10.155761 | 1.799456 | 13.305864 |
| H | 10.647562 | 2.518681 | 12.621246 |
| H | 10.840537 | 1.711394 | 14.164282 |
| C | 8.999817 | 3.024858 | 15.021569 |
| H | 9.768853 | 3.821520 | 14.969529 |
| H | 8.040861 | 3.503596 | 15.226422 |
| C | 9.362231 | 2.093447 | 16.181660 |
| H | 8.937849 | 2.488908 | 17.117850 |
| H | 10.454813 | 2.034310 | 16.306778 |
| C | 9.808704 | -0.247894 | 16.288310 |
| H | 10.531812 | -0.348546 | 15.458438 |
| H | 10.359373 | 0.000224 | 17.210049 |
| C | 9.083429 | -1.546000 | 16.509083 |
| H | 8.467588 | -1.490855 | 17.425239 |
| H | 9.834038 | -2.343123 | 16.634050 |
| C | 7.864111 | -3.184818 | 15.277026 |
| H | 8.470806 | -3.657767 | 14.488838 |
| H | 8.099681 | -3.686255 | 16.228864 |
| C | 8.137212 | 3.069135 | 12.756581 |
| H | 7.410049 | 3.694353 | 13.290140 |
| H | 8.813685 | 3.738595 | 12.189179 |
| N | 8.987062 | -0.250229 | 12.772412 |
| N | 8.868738 | 2.298595 | 13.764659 |
| O | 8.870917 | 0.765462 | 15.980595 |
| O | 8.253728 | -1.813831 | 15.395095 |

| | | | |
|---|---|---|---|
| O | 6.630937 | -1.443635 | 12.844450 |
| C | 7.380476 | -3.221608 | 11.441198 |
| H | 6.395011 | -3.692568 | 11.442611 |
| C | 8.439731 | -3.795895 | 10.707897 |
| H | 8.258455 | -4.730410 | 10.171626 |
| C | 9.682537 | -3.203828 | 10.632286 |
| H | 10.486151 | -3.646993 | 10.041936 |
| C | 6.036251 | 2.475124 | 16.361701 |
| C | 5.945693 | 3.778138 | 16.853474 |
| H | 6.075902 | 4.610285 | 16.158107 |
| C | 5.656229 | 4.027417 | 18.211235 |
| H | 5.596125 | 5.063901 | 18.551610 |
| C | 5.427712 | 3.007012 | 19.109780 |
| H | 5.181440 | 3.214800 | 20.152317 |

Yb-anti-KHQ(1+)

SCF (RwB97XD) = -1950.22815543
E(SCF)+ZPE(0 K)= -1949.545301
H(298 K)= -1949.508130
G(298 K)= -1949.610549
Lowest Frequency = 23.2100cm-1

| | | | |
|---|---|---|---|
| C | 5.821005 | 1.407045 | 17.300756 |
| C | 5.486098 | 1.666717 | 18.653693 |
| C | 5.187407 | 0.535342 | 19.457056 |
| H | 4.922389 | 0.676639 | 20.507749 |
| C | 5.190126 | -0.718430 | 18.899960 |
| H | 4.914219 | -1.596342 | 19.487533 |
| C | 5.548413 | -0.872179 | 17.537477 |
| C | 5.531186 | -2.240067 | 16.904891 |
| H | 6.392422 | -2.803188 | 17.298218 |
| H | 4.640463 | -2.791328 | 17.266202 |
| C | 4.280018 | -2.129636 | 14.804291 |
| H | 3.531109 | -2.767861 | 15.313731 |
| H | 4.398573 | -2.498884 | 13.777628 |
| C | 3.723051 | -0.718467 | 14.697161 |
| H | 2.756019 | -0.769547 | 14.170836 |
| H | 3.551798 | -0.247257 | 15.678693 |
| C | 4.139555 | 1.061175 | 13.069915 |
| H | 4.040425 | 2.011691 | 13.618827 |
| H | 3.159074 | 0.762611 | 12.664658 |
| C | 5.128442 | 1.203700 | 11.928520 |
| H | 5.172057 | 0.276583 | 11.334280 |
| H | 4.820037 | 2.043392 | 11.284695 |
| C | 7.344914 | 2.228225 | 11.767618 |
| H | 6.817288 | 2.910482 | 11.081369 |
| H | 7.980970 | 1.561680 | 11.162682 |
| C | 6.378360 | -3.335476 | 14.939139 |
| H | 6.252948 | -3.355980 | 13.855329 |
| H | 6.034933 | -4.311986 | 15.335744 |
| N | 5.898289 | 0.147919 | 16.787803 |
| N | 5.590526 | -2.221395 | 15.451617 |
| O | 6.301299 | 2.167239 | 15.130092 |
| O | 4.631457 | 0.059957 | 13.940918 |
| O | 6.399625 | 1.464783 | 12.493210 |
| C | 7.553486 | -2.029930 | 12.146617 |

| | | | |
|---|---|---|---|
| C | 8.855035 | -1.420628 | 12.089048 |
| C | 9.901150 | -1.980514 | 11.313159 |
| C | 11.104487 | -1.231029 | 11.244044 |
| H | 11.944420 | -1.614685 | 10.659688 |
| C | 11.188696 | -0.014179 | 11.872187 |
| H | 12.086915 | 0.601072 | 11.789841 |
| C | 10.087322 | 0.453157 | 12.631783 |
| C | 10.155761 | 1.799456 | 13.305864 |
| H | 10.647562 | 2.518681 | 12.621246 |
| H | 10.840537 | 1.711394 | 14.164282 |
| C | 8.999817 | 3.024858 | 15.021569 |
| H | 9.768853 | 3.821520 | 14.969529 |
| H | 8.040861 | 3.503596 | 15.226422 |
| C | 9.362231 | 2.093447 | 16.181660 |
| H | 8.937849 | 2.488908 | 17.117850 |
| H | 10.454813 | 2.034310 | 16.306778 |
| C | 9.808704 | -0.247894 | 16.288310 |
| H | 10.531812 | -0.348546 | 15.458438 |
| H | 10.359373 | 0.000224 | 17.210049 |
| C | 9.083429 | -1.546000 | 16.509083 |
| H | 8.467588 | -1.490855 | 17.425239 |
| H | 9.834038 | -2.343123 | 16.634050 |
| C | 7.864111 | -3.184818 | 15.277026 |
| H | 8.470806 | -3.657767 | 14.488838 |
| H | 8.099681 | -3.686255 | 16.228864 |
| C | 8.137212 | 3.069135 | 12.756581 |
| H | 7.410049 | 3.694353 | 13.290140 |
| H | 8.813685 | 3.738595 | 12.189179 |
| N | 8.987062 | -0.250229 | 12.772412 |
| N | 8.868738 | 2.298595 | 13.764659 |
| O | 8.870917 | 0.765462 | 15.980595 |
| O | 8.253728 | -1.813831 | 15.395095 |
| O | 6.630937 | -1.443635 | 12.844450 |
| C | 7.380476 | -3.221608 | 11.441198 |
| H | 6.395011 | -3.692568 | 11.442611 |
| C | 8.439731 | -3.795895 | 10.707897 |
| H | 8.258455 | -4.730410 | 10.171626 |
| C | 9.682537 | -3.203828 | 10.632286 |
| H | 10.486151 | -3.646993 | 10.041936 |
| C | 6.036251 | 2.475124 | 16.361701 |
| C | 5.945693 | 3.778138 | 16.853474 |
| H | 6.075902 | 4.610285 | 16.158107 |
| C | 5.656229 | 4.027417 | 18.211235 |
| H | 5.596125 | 5.063901 | 18.551610 |
| C | 5.427712 | 3.007012 | 19.109780 |
| H | 5.181440 | 3.214800 | 20.152317 |
| Yb | 6.994421 | 0.137799 | 14.416866 |

Yb3+_Ion

SCF (RwB97XD) = -37.8683700782
E(SCF)+ZPE(0 K)= -37.868370
H(298 K)= -37.866010
G(298 K)= -37.885665

| | | | |
|---|---|---|---|
| Yb | 0.887400 | 3.945900 | 9.220400 |

Yb-syn-KHQ_Ligand(2–)

SCF (RwB97XD) = -1910.49220570
E(SCF)+ZPE(0 K)= -1909.824093
H(298 K)= -1909.789093
G(298 K)= -1909.886224
Lowest Frequency = -38.5923cm-1

| | | | |
|---|---|---|---|
| N | 3.064512 | 2.642280 | 9.317809 |
| C | 3.133588 | 1.343235 | 9.512573 |
| C | 4.296972 | 0.747196 | 10.048846 |
| H | 4.320706 | -0.328576 | 10.229242 |
| C | 5.387953 | 1.537517 | 10.330474 |
| H | 6.292709 | 1.095024 | 10.754211 |
| C | 5.366307 | 2.924874 | 10.030380 |
| C | 4.150895 | 3.426743 | 9.513239 |
| C | 3.980537 | 4.803398 | 9.133249 |
| O | 2.797089 | 5.175089 | 8.727458 |
| C | 5.092892 | 5.628089 | 9.264744 |
| H | 5.020132 | 6.672651 | 8.953241 |
| C | 6.306371 | 5.129458 | 9.793980 |
| H | 7.151024 | 5.815718 | 9.891092 |
| C | 6.458718 | 3.817310 | 10.185976 |
| H | 7.405324 | 3.454040 | 10.589293 |
| N | 1.978018 | 4.421025 | 11.508006 |
| C | 2.630247 | 5.512064 | 11.841637 |
| C | 3.598393 | 5.501652 | 12.871535 |
| H | 4.145535 | 6.415231 | 13.109347 |
| C | 3.834413 | 4.334037 | 13.560247 |
| H | 4.587682 | 4.303948 | 14.351200 |
| C | 3.070600 | 3.170648 | 13.279652 |
| C | 2.136728 | 3.284427 | 12.226325 |
| C | 1.264761 | 2.209296 | 11.845098 |
| O | 0.479415 | 2.413429 | 10.822215 |
| C | 1.358583 | 1.035185 | 12.583577 |
| H | 0.690602 | 0.202826 | 12.350452 |
| C | 2.304757 | 0.913392 | 13.628674 |
| H | 2.353747 | -0.030226 | 14.177300 |
| C | 3.157123 | 1.938165 | 13.978266 |
| H | 3.875677 | 1.822601 | 14.791349 |
| C | 1.927736 | 0.544035 | 9.081333 |
| H | 2.259119 | -0.443787 | 8.702397 |
| H | 1.285123 | 0.364309 | 9.953693 |
| N | 1.155293 | 1.269136 | 8.074181 |
| C | 1.899157 | 1.385866 | 6.823079 |
| H | 1.927799 | 0.427074 | 6.267448 |
| H | 2.935994 | 1.657566 | 7.056620 |
| C | 1.328030 | 2.453072 | 5.920859 |
| H | 2.011330 | 2.597494 | 5.065911 |
| H | 0.342914 | 2.173102 | 5.505033 |
| O | 1.202616 | 3.663249 | 6.638828 |
| C | 0.846235 | 4.737747 | 5.796973 |
| H | -0.186530 | 4.594025 | 5.428944 |
| H | 1.523000 | 4.780595 | 4.925866 |
| C | 0.961945 | 6.020430 | 6.566479 |
| H | 2.004094 | 6.194132 | 6.876622 |
| H | 0.609234 | 6.848581 | 5.930435 |

| | | | |
|---|---|---|---|
| O | 0.136317 | 5.908992 | 7.718936 |
| C | -0.215471 | 7.153502 | 8.301535 |
| H | -1.236194 | 7.047554 | 8.680731 |
| H | -0.251861 | 7.928047 | 7.518579 |
| C | 0.768356 | 7.593396 | 9.375266 |
| H | 1.745356 | 7.731082 | 8.892348 |
| H | 0.450220 | 8.584783 | 9.758999 |
| N | 0.950131 | 6.622182 | 10.446601 |
| C | 2.259847 | 6.761990 | 11.083544 |
| H | 2.292392 | 7.630846 | 11.770599 |
| H | 3.010869 | 6.932894 | 10.302170 |
| C | -0.097620 | 6.623722 | 11.463643 |
| H | -0.252364 | 7.634078 | 11.893780 |
| H | 0.236824 | 5.972897 | 12.280696 |
| C | -1.436057 | 6.107622 | 10.972046 |
| H | -2.046220 | 5.800634 | 11.838590 |
| H | -2.002326 | 6.891671 | 10.440679 |
| O | -1.238097 | 5.001173 | 10.110514 |
| C | -2.418862 | 4.558920 | 9.463536 |
| H | -2.453022 | 4.977330 | 8.441542 |
| H | -3.310279 | 4.902013 | 10.011528 |
| C | -2.420629 | 3.059011 | 9.435954 |
| H | -2.372178 | 2.674728 | 10.469782 |
| H | -3.352619 | 2.698301 | 8.964777 |
| O | -1.300104 | 2.599635 | 8.710966 |
| C | -1.200735 | 1.182953 | 8.819728 |
| H | -2.174825 | 0.732149 | 8.561836 |
| H | -0.950417 | 0.934013 | 9.862018 |
| C | -0.160420 | 0.676317 | 7.855813 |
| H | -0.125193 | -0.428869 | 7.913587 |
| H | -0.496695 | 0.922075 | 6.840007 |

Yb-syn-KHQ(1+)

SCF (RwB97XD) = -1950.23603501
E(SCF)+ZPE(0 K)= -1949.551688
H(298 K)= -1949.515159
G(298 K)= -1949.614564
Lowest Frequency = 37.9442cm-1

| | | | |
|---|---|---|---|
| Yb | 0.971878 | 3.958290 | 9.262481 |
| N | 3.064512 | 2.642280 | 9.317809 |
| C | 3.133588 | 1.343235 | 9.512573 |
| C | 4.296972 | 0.747196 | 10.048846 |
| H | 4.320706 | -0.328576 | 10.229242 |
| C | 5.387953 | 1.537517 | 10.330474 |
| H | 6.292709 | 1.095024 | 10.754211 |
| C | 5.366307 | 2.924874 | 10.030380 |
| C | 4.150895 | 3.426743 | 9.513239 |
| C | 3.980537 | 4.803398 | 9.133249 |
| O | 2.797089 | 5.175089 | 8.727458 |
| C | 5.092892 | 5.628089 | 9.264744 |
| H | 5.020132 | 6.672651 | 8.953241 |
| C | 6.306371 | 5.129458 | 9.793980 |
| H | 7.151024 | 5.815718 | 9.891092 |
| C | 6.458718 | 3.817310 | 10.185976 |
| H | 7.405324 | 3.454040 | 10.589293 |

| | | | |
|---|---|---|---|
| N | 1.978018 | 4.421025 | 11.508006 |
| C | 2.630247 | 5.512064 | 11.841637 |
| C | 3.598393 | 5.501652 | 12.871535 |
| H | 4.145535 | 6.415231 | 13.109347 |
| C | 3.834413 | 4.334037 | 13.560247 |
| H | 4.587682 | 4.303948 | 14.351200 |
| C | 3.070600 | 3.170648 | 13.279652 |
| C | 2.136728 | 3.284427 | 12.226325 |
| C | 1.264761 | 2.209296 | 11.845098 |
| O | 0.479415 | 2.413429 | 10.822215 |
| C | 1.358583 | 1.035185 | 12.583577 |
| H | 0.690602 | 0.202826 | 12.350452 |
| C | 2.304757 | 0.913392 | 13.628674 |
| H | 2.353747 | -0.030226 | 14.177300 |
| C | 3.157123 | 1.938165 | 13.978266 |
| H | 3.875677 | 1.822601 | 14.791349 |
| C | 1.927736 | 0.544035 | 9.081333 |
| H | 2.259119 | -0.443787 | 8.702397 |
| H | 1.285123 | 0.364309 | 9.953693 |
| N | 1.155293 | 1.269136 | 8.074181 |
| C | 1.899157 | 1.385866 | 6.823079 |
| H | 1.927799 | 0.427074 | 6.267448 |
| H | 2.935994 | 1.657566 | 7.056620 |
| C | 1.328030 | 2.453072 | 5.920859 |
| H | 2.011330 | 2.597494 | 5.065911 |
| H | 0.342914 | 2.173102 | 5.505033 |
| O | 1.202616 | 3.663249 | 6.638828 |
| C | 0.846235 | 4.737747 | 5.796973 |
| H | -0.186530 | 4.594025 | 5.428944 |
| H | 1.523000 | 4.780595 | 4.925866 |
| C | 0.961945 | 6.020430 | 6.566479 |
| H | 2.004094 | 6.194132 | 6.876622 |
| H | 0.609234 | 6.848581 | 5.930435 |
| O | 0.136317 | 5.908992 | 7.718936 |
| C | -0.215471 | 7.153502 | 8.301535 |
| H | -1.236194 | 7.047554 | 8.680731 |
| H | -0.251861 | 7.928047 | 7.518579 |
| C | 0.768356 | 7.593396 | 9.375266 |
| H | 1.745356 | 7.731082 | 8.892348 |
| H | 0.450220 | 8.584783 | 9.758999 |
| N | 0.950131 | 6.622182 | 10.446601 |
| C | 2.259847 | 6.761990 | 11.083544 |
| H | 2.292392 | 7.630846 | 11.770599 |
| H | 3.010869 | 6.932894 | 10.302170 |
| C | -0.097620 | 6.623722 | 11.463643 |
| H | -0.252364 | 7.634078 | 11.893780 |
| H | 0.236824 | 5.972897 | 12.280696 |
| C | -1.436057 | 6.107622 | 10.972046 |
| H | -2.046220 | 5.800634 | 11.838590 |
| H | -2.002326 | 6.891671 | 10.440679 |
| O | -1.238097 | 5.001173 | 10.110514 |
| C | -2.418862 | 4.558920 | 9.463536 |
| H | -2.453022 | 4.977330 | 8.441542 |
| H | -3.310279 | 4.902013 | 10.011528 |
| C | -2.420629 | 3.059011 | 9.435954 |
| H | -2.372178 | 2.674728 | 10.469782 |
| H | -3.352619 | 2.698301 | 8.964777 |

| | | | |
|---|---|---|---|
| O | -1.300104 | 2.599635 | 8.710966 |
| C | -1.200735 | 1.182953 | 8.819728 |
| H | -2.174825 | 0.732149 | 8.561836 |
| H | -0.950417 | 0.934013 | 9.862018 |
| C | -0.160420 | 0.676317 | 7.855813 |
| H | -0.125193 | -0.428869 | 7.913587 |
| H | -0.496695 | 0.922075 | 6.840007 |

Yb-syn-H2O-KHQ(1+)

SCF (RwB97XD) = -2026.59748734
E(SCF)+ZPE(0 K)= -2025.887902
H(298 K)= -2025.849061
G(298 K)= -2025.953618
Lowest Frequency = 22.1715cm-1

| | | | |
|---|---|---|---|
| Yb | 1.083592 | 4.017571 | 9.114990 |
| N | 3.102472 | 2.629471 | 9.126157 |
| C | 3.123094 | 1.316062 | 9.200147 |
| C | 4.279612 | 0.626795 | 9.630911 |
| H | 4.268185 | -0.462607 | 9.692426 |
| C | 5.405244 | 1.342520 | 9.966206 |
| H | 6.304491 | 0.825908 | 10.310347 |
| C | 5.419911 | 2.755790 | 9.841066 |
| C | 4.216212 | 3.353160 | 9.400269 |
| C | 4.093881 | 4.771865 | 9.196897 |
| O | 2.934783 | 5.232032 | 8.808579 |
| C | 5.222434 | 5.542360 | 9.452837 |
| H | 5.176464 | 6.621334 | 9.288404 |
| C | 6.421335 | 4.945359 | 9.906464 |
| H | 7.282611 | 5.589283 | 10.099350 |
| C | 6.538153 | 3.587529 | 10.106866 |
| H | 7.473625 | 3.145627 | 10.453707 |
| N | 1.854120 | 4.488243 | 11.462743 |
| C | 2.365516 | 5.617277 | 11.897767 |
| C | 3.118006 | 5.677006 | 13.093981 |
| H | 3.538021 | 6.629590 | 13.421131 |
| C | 3.308898 | 4.529715 | 13.827439 |
| H | 3.895626 | 4.553847 | 14.748900 |
| C | 2.714855 | 3.310890 | 13.409380 |
| C | 1.980616 | 3.360727 | 12.202797 |
| C | 1.279623 | 2.220268 | 11.685076 |
| O | 0.632283 | 2.376887 | 10.565777 |
| C | 1.355392 | 1.043496 | 12.422418 |
| H | 0.818801 | 0.157235 | 12.075053 |
| C | 2.109477 | 0.987717 | 13.618084 |
| H | 2.149621 | 0.042569 | 14.164647 |
| C | 2.783413 | 2.081313 | 14.115206 |
| H | 3.353131 | 2.019256 | 15.043799 |
| C | 1.866583 | 0.584631 | 8.797139 |
| H | 2.141566 | -0.375767 | 8.316172 |
| H | 1.306558 | 0.346814 | 9.708892 |
| N | 1.020132 | 1.387687 | 7.909997 |
| C | 1.685967 | 1.499372 | 6.604033 |
| H | 1.520362 | 0.586713 | 5.998376 |
| H | 2.768051 | 1.580361 | 6.770290 |
| C | 1.266642 | 2.700160 | 5.795290 |

| | | | |
|---|---|---|---|
| H | 1.777659 | 2.654923 | 4.817243 |
| H | 0.177871 | 2.711525 | 5.606965 |
| O | 1.634155 | 3.885347 | 6.468867 |
| C | 1.463295 | 5.015953 | 5.642074 |
| H | 0.494280 | 4.942439 | 5.113340 |
| H | 2.263608 | 5.053732 | 4.881532 |
| C | 1.488419 | 6.282708 | 6.452207 |
| H | 2.438582 | 6.369665 | 7.000951 |
| H | 1.365448 | 7.138580 | 5.765887 |
| O | 0.401050 | 6.238366 | 7.367550 |
| C | 0.083719 | 7.467285 | 7.983068 |
| H | -0.981215 | 7.423839 | 8.246981 |
| H | 0.197233 | 8.295481 | 7.262087 |
| C | 0.957724 | 7.741788 | 9.189622 |
| H | 1.997036 | 7.796277 | 8.839193 |
| H | 0.699928 | 8.739399 | 9.600022 |
| N | 0.908028 | 6.709319 | 10.226598 |
| C | 2.117686 | 6.833827 | 11.051191 |
| H | 2.072703 | 7.735108 | 11.693644 |
| H | 2.975776 | 6.949650 | 10.376745 |
| C | -0.279899 | 6.785035 | 11.083990 |
| H | -0.559826 | 7.839305 | 11.280499 |
| H | -0.023765 | 6.338253 | 12.054462 |
| C | -1.517145 | 6.057223 | 10.596270 |
| H | -2.299388 | 6.206168 | 11.361704 |
| H | -1.918147 | 6.471858 | 9.654608 |
| O | -1.265499 | 4.676187 | 10.438828 |
| C | -2.469037 | 3.925817 | 10.475447 |
| H | -3.136685 | 4.252577 | 9.653698 |
| H | -2.993891 | 4.133227 | 11.424618 |
| C | -2.243321 | 2.436618 | 10.389991 |
| H | -1.506829 | 2.125267 | 11.145050 |
| H | -3.209789 | 1.937331 | 10.586586 |
| O | -1.774282 | 2.104798 | 9.103131 |
| C | -1.240292 | 0.807097 | 8.939747 |
| H | -2.062177 | 0.092293 | 8.755541 |
| H | -0.722963 | 0.513143 | 9.863924 |
| C | -0.300802 | 0.763818 | 7.745073 |
| H | -0.178245 | -0.301026 | 7.459521 |
| H | -0.808387 | 1.251354 | 6.899583 |
| O | -0.829686 | 3.926488 | 7.636717 |
| H | -1.512657 | 3.331702 | 8.012537 |
| H | -1.184320 | 4.757382 | 7.305430 |

KHQLaΔ_λλλ_λλλ_opt.log

SCF (RwB97XD) = -1942.42343658
E(SCF)+ZPE(0 K)= -1941.740634
H(298 K)= -1941.703805
G(298 K)= -1941.804991
Lowest Frequency = 10.9892cm-1

81

| | | | |
|---|---|---|---|
| C | -3.691406 | 1.074612 | 0.378470 |
| N | -2.640801 | 0.599783 | 1.296318 |
| O | -2.042382 | 1.992321 | -1.090127 |
| C | -3.251659 | 1.243277 | -1.068524 |

| | | | |
|---|---|---|---|
| C | -2.495741 | 1.466396 | 2.472611 |
| C | -1.862009 | 2.809365 | 2.146508 |
| O | -0.507028 | 2.691019 | 1.737213 |
| C | 0.456144 | 2.635049 | 2.782601 |
| C | 1.815536 | 2.895033 | 2.173183 |
| O | 2.045926 | 2.002339 | 1.093430 |
| O | 0.506979 | 2.694746 | -1.733366 |
| C | -0.455923 | 2.634838 | -2.778757 |
| C | -1.816642 | 2.887163 | -2.169068 |
| N | 2.651158 | 0.613851 | -1.293719 |
| C | 2.501561 | 1.480241 | -2.469624 |
| C | 1.861339 | 2.819924 | -2.142689 |
| C | 3.259056 | 1.259561 | 1.071319 |
| C | 3.699444 | 1.093672 | -0.375802 |
| C | -2.835688 | -0.795757 | 1.701220 |
| C | 2.853241 | -0.780474 | -1.699297 |
| C | 2.494885 | -1.774676 | -0.621372 |
| C | -2.471942 | -1.787668 | 0.622967 |
| C | 1.287579 | -2.201337 | 1.319046 |
| C | 1.712432 | -3.551115 | 1.374571 |
| C | 2.578880 | -3.979508 | 0.333815 |
| C | 2.985254 | -3.101837 | -0.642736 |
| C | -2.955134 | -3.117464 | 0.643932 |
| C | -2.544036 | -3.992630 | -0.332890 |
| C | -1.679941 | -3.559251 | -1.373549 |
| C | -1.262381 | -2.207214 | -1.317606 |
| La | 0.005149 | 0.676182 | 0.001338 |
| H | -4.065228 | 2.046804 | 0.733731 |
| H | -4.558204 | 0.391339 | 0.393755 |
| H | -4.041031 | 1.784001 | -1.615018 |
| H | -3.083217 | 0.274329 | -1.562666 |
| H | 3.095783 | 0.289569 | 1.565152 |
| H | 4.045727 | 1.804175 | 1.617849 |
| H | 4.068307 | 2.067871 | -0.730753 |
| H | 4.569692 | 0.414812 | -0.391432 |
| H | -3.874089 | -0.977040 | 2.040588 |
| H | -2.173545 | -0.993716 | 2.555765 |
| H | 3.892496 | -0.956178 | -2.038978 |
| H | 2.191923 | -0.981535 | -2.553767 |
| C | 1.270003 | -4.369322 | 2.444342 |
| H | 2.933526 | -5.013094 | 0.327250 |
| H | 3.667694 | -3.417149 | -1.433745 |
| H | -3.635841 | -3.436710 | 1.434857 |
| H | -2.893081 | -5.028123 | -0.326621 |
| C | -1.233116 | -4.374719 | -2.443583 |
| N | -1.641641 | -1.379130 | -0.309818 |
| N | 1.662377 | -1.370890 | 0.311526 |
| C | 0.439726 | -1.623559 | 2.334011 |
| O | 0.081799 | -0.374014 | 2.211409 |
| C | 0.047236 | -2.467494 | 3.370586 |
| C | -0.417644 | -1.624551 | -2.332370 |
| O | -0.066360 | -0.373161 | -2.209310 |
| C | -0.020657 | -2.466018 | -3.369243 |
| H | 0.431478 | 1.639560 | 3.256983 |
| H | 0.243959 | 3.415130 | 3.533543 |
| H | 2.582455 | 2.768806 | 2.952744 |
| H | 1.872255 | 3.925573 | 1.786576 |

| | | | |
|---|---|---|---|
| H | -0.426057 | 1.640010 | -3.254193 |
| H | -0.247827 | 3.416776 | -3.528915 |
| H | -1.878695 | 3.917048 | -1.781531 |
| H | -2.582907 | 2.757681 | -2.948736 |
| H | 2.378206 | 3.320427 | -1.311810 |
| H | 1.921875 | 3.482965 | -3.021565 |
| H | 3.474260 | 1.663931 | -2.968144 |
| H | 1.857085 | 0.951422 | -3.185354 |
| H | -1.925824 | 3.471551 | 3.025799 |
| H | -2.381211 | 3.307917 | 1.315922 |
| H | -3.469438 | 1.645084 | 2.971002 |
| H | -1.848843 | 0.940474 | 3.188284 |
| C | 0.457077 | -3.818434 | 3.411078 |
| C | -0.423204 | -3.819139 | -3.410168 |
| H | -0.082638 | -4.439130 | -4.242905 |
| H | -1.540759 | -5.420400 | -2.495167 |
| H | 0.602700 | -2.056098 | -4.167315 |
| H | 0.119845 | -4.440521 | 4.243608 |
| H | 1.583296 | -5.413339 | 2.495606 |
| H | -0.578341 | -2.061200 | 4.168774 |

KHQLuΔ_δδδ_δδδ_opt2.log

SCF (RwB97XD) = -1950.78988945
E(SCF)+ZPE(0 K)= -1950.106168
H(298 K)= -1950.069489
G(298 K)= -1950.170115
Lowest Frequency = 23.7976cm-1

81

| | | | |
|---|---|---|---|
| C | 3.634828 | -0.774246 | -0.455427 |
| N | 2.882152 | -0.524360 | 0.769066 |
| O | 1.703640 | -1.601432 | -1.562265 |
| C | 3.047442 | -1.915926 | -1.252194 |
| C | 3.141758 | -1.536031 | 1.791089 |
| C | 1.869055 | -2.074118 | 2.406647 |
| O | 1.007327 | -2.495122 | 1.339426 |
| C | 0.067834 | -3.487681 | 1.703715 |
| C | -1.125886 | -2.871855 | 2.392151 |
| O | -1.672626 | -1.914340 | 1.507317 |
| O | -0.945275 | -2.509402 | -1.449687 |
| C | 0.078203 | -3.247998 | -2.097191 |
| C | 1.133990 | -2.315958 | -2.645178 |
| N | -2.860496 | -0.572026 | -0.573917 |
| C | -3.180062 | -1.662628 | -1.491734 |
| C | -1.997870 | -2.097357 | -2.326205 |
| C | -3.084883 | -1.868768 | 1.523576 |
| C | -3.569110 | -0.695900 | 0.698533 |
| C | 3.053769 | 0.841052 | 1.251157 |
| C | -3.078477 | 0.749031 | -1.165898 |
| C | -2.428057 | 1.818540 | -0.319673 |
| C | 2.417614 | 1.838269 | 0.311242 |
| C | -0.924192 | 2.228466 | 1.397109 |
| C | -1.282301 | 3.590708 | 1.500228 |
| C | -2.255714 | 4.046542 | 0.572471 |
| C | -2.847481 | 3.167413 | -0.307455 |
| C | 2.854317 | 3.180557 | 0.232924 |

| | | | |
|---|---|---|---|
| C | 2.277066 | 4.024743 | -0.686642 |
| C | 1.294015 | 3.538062 | -1.587206 |
| C | 0.919090 | 2.184842 | -1.424581 |
| Lu | -0.015206 | -0.536460 | 0.021663 |
| H | 0.541281 | -4.252150 | 2.341464 |
| H | -0.262532 | -3.966348 | 0.770565 |
| H | -0.849227 | -2.382061 | 3.340971 |
| H | -1.861053 | -3.663837 | 2.611698 |
| H | -0.350049 | -3.878579 | -2.893438 |
| H | 0.524383 | -3.910231 | -1.339278 |
| H | 1.905014 | -2.885458 | -3.186698 |
| H | 0.689955 | -1.576537 | -3.327322 |
| H | 4.124061 | 1.098473 | 1.383799 |
| H | 2.569510 | 0.923719 | 2.233492 |
| H | -4.159010 | 0.971031 | -1.274587 |
| H | -2.630304 | 0.760005 | -2.168596 |
| C | -0.676405 | 4.372689 | 2.518048 |
| H | -2.560268 | 5.095819 | 0.587800 |
| H | -3.634281 | 3.497984 | -0.987252 |
| H | 3.643350 | 3.531567 | 0.899845 |
| H | 2.594272 | 5.068307 | -0.751966 |
| C | 0.692609 | 4.293597 | -2.626927 |
| N | 1.448700 | 1.392576 | -0.459650 |
| N | -1.459552 | 1.405771 | 0.467474 |
| C | 0.010206 | 1.597181 | 2.288921 |
| O | 0.295005 | 0.340763 | 2.067437 |
| C | 0.545793 | 2.395027 | 3.293150 |
| C | -0.042420 | 1.547166 | -2.283795 |
| O | -0.361067 | 0.306959 | -2.023686 |
| C | -0.578937 | 2.318986 | -3.307916 |
| H | 4.703990 | -0.983574 | -0.251927 |
| H | 3.626326 | -2.034142 | -2.182834 |
| H | 3.091831 | -2.872201 | -0.699965 |
| H | -4.664062 | -0.794145 | 0.556621 |
| H | -3.398811 | 0.221184 | 1.274475 |
| H | -3.451285 | -1.747987 | 2.557367 |
| H | -3.476504 | -2.831408 | 1.146170 |
| H | -1.637239 | -1.278794 | -2.966399 |
| H | -2.297615 | -2.954054 | -2.952409 |
| H | -3.514721 | -2.531524 | -0.909222 |
| H | -4.016397 | -1.399068 | -2.166579 |
| H | 2.104721 | -2.943731 | 3.041621 |
| H | 1.357912 | -1.301598 | 2.997904 |
| H | 3.792526 | -1.148541 | 2.595452 |
| H | 3.687505 | -2.375839 | 1.336646 |
| H | 3.592949 | 0.124565 | -1.084377 |
| C | 0.204239 | 3.765580 | 3.386908 |
| C | -0.212579 | 3.676116 | -3.461734 |
| H | -0.665665 | 4.248600 | -4.274566 |
| H | -1.291483 | 1.862307 | -3.998565 |
| H | 0.965219 | 5.341219 | -2.764636 |
| H | 0.657200 | 4.358635 | 4.184986 |
| H | -0.927837 | 5.430008 | 2.615934 |
| H | 1.233705 | 1.952652 | 4.017185 |

KHQLuΔ_δδλ_δδλ_opt.log

SCF (RwB97XD) = -1950.78648661
E(SCF)+ZPE(0 K)= -1950.102776
H(298 K)= -1950.066105
G(298 K)= -1950.166356
Lowest Frequency = 25.2323cm-1

81

| | | | |
|---|---|---|---|
| C | 3.522819 | 1.060975 | -0.221150 |
| N | 2.633240 | 0.855622 | 0.919906 |
| O | 2.517773 | -0.884226 | -1.198932 |
| C | 3.727823 | -0.163423 | -1.092942 |
| C | 3.178928 | 0.033585 | 1.993467 |
| C | 3.100066 | -1.465046 | 1.742915 |
| O | 1.761605 | -1.851887 | 1.471957 |
| C | 1.201256 | -2.899146 | 2.247933 |
| C | -0.209277 | -2.499045 | 2.617067 |
| O | -0.935367 | -2.179444 | 1.444383 |
| O | 0.489283 | -2.714872 | -1.199533 |
| C | 1.825447 | -3.127320 | -1.431736 |
| C | 2.578675 | -1.991166 | -2.078593 |
| N | -2.108778 | -1.711869 | -1.069159 |
| C | -1.654126 | -2.414881 | -2.261405 |
| C | -0.488521 | -3.349003 | -2.016211 |
| C | -1.755022 | -3.198253 | 0.913662 |
| C | -2.733980 | -2.559614 | -0.054980 |
| C | 2.175185 | 2.158983 | 1.414021 |
| C | -2.996154 | -0.610813 | -1.447288 |
| C | -3.032697 | 0.470113 | -0.397534 |
| C | 1.207875 | 2.787179 | 0.439369 |
| C | -1.996320 | 1.431504 | 1.437888 |
| C | -3.032848 | 2.373988 | 1.630720 |
| C | -4.103727 | 2.324599 | 0.700406 |
| C | -4.120922 | 1.366700 | -0.285270 |
| C | 0.959417 | 4.177077 | 0.380589 |
| C | 0.121390 | 4.668183 | -0.595362 |
| C | -0.436526 | 3.798047 | -1.569077 |
| C | -0.133156 | 2.426706 | -1.416548 |
| Lu | 0.228610 | -0.437138 | 0.012425 |
| H | 2.578223 | 0.237237 | 2.889871 |
| H | 4.232104 | 0.294381 | 2.225148 |
| H | 3.460288 | -1.980081 | 2.646833 |
| H | 3.738537 | -1.793317 | 0.910655 |
| H | 1.790540 | -3.058850 | 3.164466 |
| H | 1.216186 | -3.835763 | 1.662244 |
| H | -0.186260 | -1.582247 | 3.220408 |
| H | -0.708638 | -3.304886 | 3.177996 |
| H | 1.853739 | -4.026257 | -2.065812 |
| H | 2.280869 | -3.375423 | -0.461723 |
| H | 3.625372 | -2.293232 | -2.249101 |
| H | 2.126326 | -1.714591 | -3.045546 |
| H | -1.316563 | -1.641736 | -2.962112 |
| H | -2.467805 | -2.998723 | -2.738735 |
| H | -0.793598 | -4.297099 | -1.540882 |
| H | -0.052789 | -3.601847 | -2.996170 |
| H | 3.022780 | 2.850495 | 1.588861 |
| H | 1.664420 | 2.007897 | 2.372528 |
| H | -4.024872 | -0.967168 | -1.650423 |

| | | | |
|---|---|---|---|
| H | -2.623141 | -0.167533 | -2.379742 |
| C | -2.951336 | 3.266235 | 2.730462 |
| H | -4.928639 | 3.035040 | 0.794368 |
| H | -4.958123 | 1.290264 | -0.981019 |
| H | 1.434320 | 4.844575 | 1.101397 |
| H | -0.090241 | 5.738852 | -0.649927 |
| C | -1.230090 | 4.196160 | -2.676241 |
| N | 0.625499 | 1.959498 | -0.398988 |
| N | -1.997945 | 0.545818 | 0.409777 |
| C | -0.881820 | 1.323514 | 2.340820 |
| O | 0.029206 | 0.427694 | 2.079401 |
| C | -0.860758 | 2.202680 | 3.419385 |
| C | -0.565422 | 1.423210 | -2.352013 |
| O | -0.240419 | 0.184710 | -2.097810 |
| C | -1.310353 | 1.868975 | -3.438929 |
| H | 3.074550 | 1.843417 | -0.845368 |
| H | 4.517741 | 1.431476 | 0.098358 |
| H | 4.065097 | 0.165713 | -2.090545 |
| H | 4.512812 | -0.828123 | -0.687920 |
| H | -3.361100 | -3.350920 | -0.511590 |
| H | -3.401645 | -1.925736 | 0.545213 |
| H | -2.331780 | -3.672806 | 1.725549 |
| H | -1.136217 | -3.981086 | 0.446213 |
| C | -1.639085 | 3.238415 | -3.578871 |
| H | -1.488324 | 5.247596 | -2.812245 |
| H | -2.234071 | 3.542500 | -4.443400 |
| H | -1.631656 | 1.149611 | -4.195748 |
| C | -1.883372 | 3.162472 | 3.594741 |
| H | -3.737511 | 4.006179 | 2.889090 |
| H | -1.823159 | 3.836918 | 4.452230 |
| H | -0.044495 | 2.133732 | 4.142176 |

KHQLuΔ_δλδ_δλδ_opt.log

SCF (RwB97XD) = -1950.78598718
E(SCF)+ZPE(0 K)= -1950.101531
H(298 K)= -1950.064803
G(298 K)= -1950.166175
Lowest Frequency = 12.8609cm-1

81

| | | | |
|---|---|---|---|
| C | 3.500271 | -0.702062 | -0.790486 |
| N | 2.912478 | -0.485165 | 0.526176 |
| O | 1.581657 | -2.052160 | -1.315958 |
| C | 2.994292 | -1.979816 | -1.423279 |
| C | 3.342621 | -1.487397 | 1.496762 |
| C | 2.243921 | -1.880500 | 2.450822 |
| O | 1.133678 | -2.370245 | 1.707636 |
| C | 0.205448 | -3.001473 | 2.558364 |
| C | -1.042435 | -3.293501 | 1.778418 |
| O | -1.579891 | -2.067899 | 1.312351 |
| O | -1.131686 | -2.370189 | -1.710042 |
| C | -0.199577 | -2.986047 | -2.567790 |
| C | 1.051466 | -3.276791 | -1.792585 |
| N | -2.920711 | -0.500212 | -0.517352 |
| C | -3.346234 | -1.503675 | -1.488606 |
| C | -2.247908 | -1.885648 | -2.447520 |

| | | | |
|---|---|---|---|
| C | -2.991990 | -1.999272 | 1.429170 |
| C | -3.505191 | -0.722382 | 0.799966 |
| C | 3.131157 | 0.870920 | 1.018391 |
| C | -3.147784 | 0.855318 | -1.007540 |
| C | -2.425518 | 1.875762 | -0.165155 |
| C | 2.403512 | 1.888686 | 0.177383 |
| C | -0.822038 | 2.225978 | 1.466939 |
| C | -1.142401 | 3.593692 | 1.610425 |
| C | -2.155450 | 4.086924 | 0.746815 |
| C | -2.813042 | 3.233887 | -0.109766 |
| C | 2.783186 | 3.249196 | 0.125800 |
| C | 2.120909 | 4.100780 | -0.728557 |
| C | 1.110728 | 3.604151 | -1.593558 |
| C | 0.798316 | 2.234153 | -1.454114 |
| Lu | -0.004411 | -0.542873 | 0.002490 |
| H | 4.210360 | 1.125126 | 1.046907 |
| H | 2.753028 | 0.928693 | 2.048968 |
| H | -4.228465 | 1.103214 | -1.035048 |
| H | -2.770367 | 0.916923 | -2.038178 |
| C | -0.461561 | 4.347937 | 2.601474 |
| H | -2.434017 | 5.142425 | 0.793572 |
| H | -3.627647 | 3.590379 | -0.742042 |
| H | 3.595461 | 3.608657 | 0.759392 |
| H | 2.393322 | 5.158016 | -0.772290 |
| C | 0.425297 | 4.357609 | -2.582031 |
| N | 1.406060 | 1.432753 | -0.547283 |
| N | -1.425053 | 1.423789 | 0.557778 |
| C | 0.152867 | 1.567421 | 2.290038 |
| O | 0.402157 | 0.312832 | 2.027808 |
| C | 0.762601 | 2.336495 | 3.274407 |
| C | -0.173520 | 1.572868 | -2.278702 |
| O | -0.416318 | 0.316264 | -2.020087 |
| C | -0.788020 | 2.341553 | -3.260410 |
| H | 4.608038 | -0.745208 | -0.758907 |
| H | 3.285533 | -1.997131 | -2.487467 |
| H | 3.432662 | -2.873280 | -0.946317 |
| H | -4.612791 | -0.770636 | 0.770361 |
| H | -3.233472 | 0.120387 | 1.447283 |
| H | -3.276409 | -2.018087 | 2.495161 |
| H | -3.431106 | -2.893456 | 0.954313 |
| H | -1.916385 | -1.027801 | -3.052168 |
| H | -2.616684 | -2.683902 | -3.115203 |
| H | -3.661563 | -2.411490 | -0.958614 |
| H | -4.226743 | -1.164294 | -2.066521 |
| H | 2.616185 | -2.678562 | 3.116768 |
| H | 1.904240 | -1.027107 | 3.057215 |
| H | 4.218217 | -1.142163 | 2.078611 |
| H | 3.667408 | -2.391370 | 0.965882 |
| H | 0.854506 | -3.946533 | -0.936744 |
| H | 1.773203 | -3.776175 | -2.457392 |
| H | 0.040966 | -2.308479 | -3.405895 |
| H | -0.614771 | -3.924611 | -2.978124 |
| H | 3.225841 | 0.140636 | -1.436798 |
| H | -1.761975 | -3.804398 | 2.436737 |
| H | -0.839267 | -3.953141 | 0.916266 |
| H | 0.627217 | -3.940854 | 2.960041 |
| H | -0.041199 | -2.334091 | 3.402849 |

| | | | |
|---|---|---|---|
| C | 0.455391 | 3.711147 | 3.409454 |
| C | -0.488493 | 3.718250 | -3.391544 |
| H | -1.002525 | 4.289476 | -4.168247 |
| H | 0.643128 | 5.419731 | -2.705293 |
| H | -1.507619 | 1.871435 | -3.934578 |
| H | 0.965838 | 4.282844 | 4.188174 |
| H | -0.685383 | 5.408430 | 2.727976 |
| H | 1.484262 | 1.868338 | 3.947734 |

KHQLuΔ_δλλ_δλλ_opt.log

SCF (RwB97XD) = -1950.79135692
E(SCF)+ZPE(0 K)= -1950.106470
H(298 K)= -1950.070281
G(298 K)= -1950.168483
Lowest Frequency = 36.6030cm-1

81

| | | | |
|---|---|---|---|
| C | -3.655652 | -0.665856 | 0.306325 |
| N | -2.782428 | -0.485324 | -0.851058 |
| O | -1.863071 | -1.820958 | 1.395783 |
| C | -3.263395 | -1.808216 | 1.219944 |
| C | -2.925935 | -1.503643 | -1.883830 |
| C | -2.146697 | -2.775755 | -1.582425 |
| O | -0.757562 | -2.529880 | -1.428628 |
| C | -0.001819 | -2.628061 | -2.629650 |
| C | 1.433312 | -2.865697 | -2.252551 |
| O | 1.857733 | -1.835074 | -1.387001 |
| O | 0.751002 | -2.520546 | 1.443018 |
| C | -0.005346 | -2.609355 | 2.644316 |
| C | -1.440873 | -2.846499 | 2.268425 |
| N | 2.779736 | -0.484459 | 0.849902 |
| C | 2.921236 | -1.494918 | 1.890613 |
| C | 2.139649 | -2.767868 | 1.599078 |
| C | 3.258015 | -1.824227 | -1.210900 |
| C | 3.652607 | -0.675717 | -0.306037 |
| C | -2.945723 | 0.872685 | -1.372201 |
| C | 2.945700 | 0.877263 | 1.360404 |
| C | 2.343257 | 1.876313 | 0.400878 |
| C | -2.340882 | 1.878074 | -0.420837 |
| C | 0.953475 | 2.177065 | -1.429892 |
| C | 1.298202 | 3.540030 | -1.572000 |
| C | 2.199355 | 4.057313 | -0.604635 |
| C | 2.740174 | 3.231632 | 0.354447 |
| C | -2.734391 | 3.234727 | -0.385585 |
| C | -2.191364 | 4.066946 | 0.566579 |
| C | -1.291431 | 3.555422 | 1.538135 |
| C | -0.950220 | 2.190445 | 1.407322 |
| Lu | -0.001467 | -0.555224 | -0.000246 |
| H | -4.011350 | 1.123840 | -1.541400 |
| H | -2.432239 | 0.942074 | -2.338783 |
| H | 4.011795 | 1.127534 | 1.527958 |
| H | 2.432037 | 0.955278 | 2.326232 |
| C | 0.750518 | 4.269819 | -2.658991 |
| H | 2.487519 | 5.110356 | -0.649871 |
| H | 3.470424 | 3.607164 | 1.072913 |
| H | -3.463730 | 3.606170 | -1.107096 |

| | | | |
|---|---|---|---|
| H | -2.476820 | 5.121066 | 0.603082 |
| C | -0.741716 | 4.292813 | 2.618945 |
| N | -1.438694 | 1.410621 | 0.414293 |
| N | 1.439915 | 1.404220 | -0.430405 |
| C | 0.076174 | 1.500596 | -2.346969 |
| O | -0.227004 | 0.257383 | -2.086839 |
| C | -0.401889 | 2.247784 | -3.418069 |
| C | -0.074501 | 1.519351 | 2.329844 |
| O | 0.225539 | 0.273270 | 2.079951 |
| C | 0.405578 | 2.274180 | 3.394671 |
| H | -4.711949 | -0.808155 | 0.001388 |
| H | -3.760125 | -1.666263 | 2.195732 |
| H | -3.600519 | -2.787435 | 0.834502 |
| H | 4.708605 | -0.817818 | 0.000025 |
| H | 3.612545 | 0.242961 | -0.904023 |
| H | 3.755336 | -1.690906 | -2.187608 |
| H | 3.592818 | -2.801213 | -0.817857 |
| H | -1.558350 | -3.827051 | 1.770035 |
| H | -2.055172 | -2.846262 | 3.185410 |
| H | 0.095677 | -1.671520 | 3.212180 |
| H | 0.348818 | -3.461293 | 3.246978 |
| H | -3.613724 | 0.257310 | 0.897192 |
| H | 2.047404 | -2.872905 | -3.169650 |
| H | 1.548919 | -3.843078 | -1.747537 |
| H | -0.358016 | -3.483112 | -3.226673 |
| H | -0.100944 | -1.693508 | -3.203316 |
| H | -3.987332 | -1.771062 | -2.063558 |
| H | -2.533197 | -1.084275 | -2.820392 |
| H | -2.303877 | -3.498509 | -2.399177 |
| H | -2.476488 | -3.262455 | -0.659336 |
| H | 2.468781 | -3.262572 | 0.680013 |
| H | 2.295252 | -3.484401 | 2.421587 |
| H | 2.529210 | -1.067499 | 2.823828 |
| H | 3.982111 | -1.762932 | 2.072537 |
| C | -0.067419 | 3.615714 | -3.554235 |
| C | 0.074649 | 3.644056 | 3.519505 |
| H | 0.483785 | 4.201796 | 4.365282 |
| H | -0.985725 | 5.349836 | 2.736855 |
| H | 1.045517 | 1.795982 | 4.139801 |
| H | -0.475018 | 4.167457 | -4.404676 |
| H | 0.997279 | 5.325187 | -2.785663 |
| H | -1.042996 | 1.765048 | -4.159260 |

KHQLuΔ_λδδ_λδδ_opt.log

SCF (RwB97XD) = -1950.78961773
E(SCF)+ZPE(0 K)= -1950.105533
H(298 K)= -1950.069153
G(298 K)= -1950.167884
Lowest Frequency = 43.3500cm-1

81

| | | | |
|---|---|---|---|
| C | 3.720161 | 0.695709 | 0.506752 |
| N | 2.905252 | 0.590709 | -0.711771 |
| O | 1.807170 | 1.796863 | 1.417817 |
| C | 2.898882 | 0.962411 | 1.765498 |
| C | 3.109447 | 1.715312 | -1.620277 |

| | | | |
|---|---|---|---|
| C | 1.863120 | 2.129008 | -2.368152 |
| O | 0.864947 | 2.509023 | -1.413912 |
| C | -0.155960 | 3.348667 | -1.927559 |
| C | -1.287715 | 2.529113 | -2.494991 |
| O | -1.803971 | 1.776093 | -1.419573 |
| O | -0.868295 | 2.505308 | 1.410316 |
| C | 0.144876 | 3.356003 | 1.921009 |
| C | 1.284269 | 2.548767 | 2.490803 |
| N | -2.891049 | 0.566629 | 0.713921 |
| C | -3.105227 | 1.691549 | 1.619787 |
| C | -1.862414 | 2.118513 | 2.366087 |
| C | -2.888100 | 0.930640 | -1.764456 |
| C | -3.706998 | 0.661294 | -0.504781 |
| C | 3.021621 | -0.717998 | -1.346656 |
| C | -2.995765 | -0.741444 | 1.352100 |
| C | -2.383403 | -1.793819 | 0.456122 |
| C | 2.418832 | -1.773469 | -0.447812 |
| C | -0.954454 | -2.179557 | -1.330901 |
| C | -1.294821 | -3.547227 | -1.418078 |
| C | -2.218120 | -4.019258 | -0.447411 |
| C | -2.780457 | -3.150673 | 0.460455 |
| C | 2.828373 | -3.126607 | -0.448197 |
| C | 2.273930 | -3.997723 | 0.462101 |
| C | 1.346274 | -3.531411 | 1.431382 |
| C | 0.993344 | -2.167191 | 1.340229 |
| Lu | 0.006939 | 0.596059 | 0.000834 |
| H | 4.459590 | 1.501509 | 0.381988 |
| H | 4.301301 | -0.225655 | 0.666984 |
| H | 3.530493 | 1.472097 | 2.511865 |
| H | 2.513376 | 0.036945 | 2.219018 |
| H | 0.255313 | 4.032849 | -2.686106 |
| H | -0.529518 | 3.947577 | -1.083550 |
| H | -0.932480 | 1.854810 | -3.293829 |
| H | -2.066895 | 3.194410 | -2.906629 |
| H | -0.272533 | 4.038768 | 2.677485 |
| H | 0.512668 | 3.955660 | 1.074997 |
| H | 2.057418 | 3.222550 | 2.899998 |
| H | 0.935443 | 1.873975 | 3.292048 |
| H | -2.494233 | 0.006979 | -2.214477 |
| H | -3.524167 | 1.431958 | -2.512702 |
| H | -4.452689 | 1.461657 | -0.382406 |
| H | -4.280924 | -0.265109 | -0.662018 |
| H | 4.075191 | -0.978633 | -1.571247 |
| H | 2.472429 | -0.701172 | -2.298231 |
| H | -4.047015 | -1.010928 | 1.577136 |
| H | -2.447089 | -0.717231 | 2.303786 |
| C | -0.721822 | -4.322461 | -2.459409 |
| H | -2.505877 | -5.073358 | -0.449977 |
| H | -3.526824 | -3.494354 | 1.178220 |
| H | 3.577993 | -3.465456 | -1.164868 |
| H | 2.571383 | -5.049117 | 0.467719 |
| C | 0.780435 | -4.308829 | 2.475003 |
| N | 1.492847 | -1.348922 | 0.383894 |
| N | -1.461511 | -1.363119 | -0.376964 |
| C | -0.067453 | -1.539580 | -2.264511 |
| O | 0.208509 | -0.275790 | -2.063511 |
| C | 0.437248 | -2.331072 | -3.289003 |

| | | | |
|---|---|---|---|
| C | 0.100675 | -1.532613 | 2.272119 |
| O | -0.186680 | -0.271929 | 2.067602 |
| C | -0.396719 | -2.325713 | 3.298940 |
| H | -2.110110 | 2.990602 | 2.993007 |
| H | -1.461757 | 1.312279 | 2.998292 |
| H | -3.905599 | 1.485052 | 2.357679 |
| H | -3.433477 | 2.551533 | 1.021114 |
| H | 2.103130 | 3.001779 | -2.997114 |
| H | 1.470289 | 1.317620 | -2.998627 |
| H | 3.429516 | 2.579721 | -1.023549 |
| H | 3.912005 | 1.514404 | -2.357345 |
| C | -0.059462 | -3.697510 | 3.380613 |
| C | 0.112515 | -3.706112 | -3.366747 |
| H | 0.539958 | -4.294822 | -4.181847 |
| H | -0.962441 | -5.383461 | -2.544114 |
| H | 1.090815 | -1.879628 | -4.038683 |
| H | -0.481445 | -4.287742 | 4.197455 |
| H | 1.030785 | -5.367323 | 2.562791 |
| H | -1.054281 | -1.878071 | 4.047404 |

KHQLuΔ_λδλ_λδλ_opt.log

SCF (RwB97XD) = -1950.79826200
E(SCF)+ZPE(0 K)= -1950.114154
H(298 K)= -1950.077781
G(298 K)= -1950.176789
Lowest Frequency = 29.8080cm-1

81

| | | | |
|---|---|---|---|
| C | 3.692738 | 0.876218 | -0.161193 |
| N | 2.685951 | 0.529948 | -1.171864 |
| O | 1.937527 | 1.821931 | 1.128374 |
| C | 3.123313 | 1.051624 | 1.238913 |
| C | 2.606614 | 1.499692 | -2.258281 |
| C | 1.985895 | 2.806763 | -1.807562 |
| O | 0.645528 | 2.581584 | -1.386167 |
| C | -0.374278 | 3.373791 | -1.967369 |
| C | -1.536078 | 2.483003 | -2.326987 |
| O | -1.943543 | 1.837492 | -1.137300 |
| O | -0.645292 | 2.587735 | 1.376891 |
| C | 0.381169 | 3.372051 | 1.957060 |
| C | 1.535563 | 2.471994 | 2.317453 |
| N | -2.702032 | 0.552659 | 1.163736 |
| C | -2.614784 | 1.522039 | 2.249886 |
| C | -1.983593 | 2.823936 | 1.798781 |
| C | -3.135616 | 1.076875 | -1.247203 |
| C | -3.706156 | 0.906783 | 0.153107 |
| C | 2.811485 | -0.842372 | -1.649525 |
| C | -2.838810 | -0.818374 | 1.642122 |
| C | -2.363963 | -1.805562 | 0.604527 |
| C | 2.328095 | -1.825099 | -0.611618 |
| C | -1.131680 | -2.121067 | -1.333279 |
| C | -1.513462 | -3.476221 | -1.454361 |
| C | -2.350557 | -3.981536 | -0.424279 |
| C | -2.796506 | -3.151946 | 0.577856 |
| C | 2.749356 | -3.175049 | -0.584409 |
| C | 2.296512 | -4.000483 | 0.418058 |

| | | | |
|---|---|---|---|
| C | 1.463717 | -3.487760 | 1.447952 |
| C | 1.093207 | -2.129527 | 1.326296 |
| Lu | -0.008063 | 0.609390 | -0.004044 |
| H | 4.191506 | 1.813919 | -0.452811 |
| H | 4.486458 | 0.111502 | -0.119786 |
| H | 3.855944 | 1.589030 | 1.864346 |
| H | 2.893525 | 0.091332 | 1.724692 |
| H | 1.959659 | 1.072987 | -3.036577 |
| H | 3.598324 | 1.711531 | -2.707432 |
| H | 1.995149 | 3.519510 | -2.645955 |
| H | 2.541302 | 3.259572 | -0.971523 |
| H | -0.004834 | 3.872493 | -2.876450 |
| H | -0.690033 | 4.147583 | -1.246850 |
| H | -1.231860 | 1.728816 | -3.071949 |
| H | -2.361398 | 3.095721 | -2.729238 |
| H | 0.016051 | 3.874847 | 2.865632 |
| H | 0.703250 | 4.142373 | 1.235631 |
| H | 2.365999 | 3.078224 | 2.719005 |
| H | 1.225196 | 1.721110 | 3.063212 |
| H | -1.971224 | 1.090254 | 3.028196 |
| H | -3.604711 | 1.741966 | 2.699071 |
| H | -2.535616 | 3.281216 | 0.962932 |
| H | -1.986689 | 3.536801 | 2.637121 |
| H | -2.913851 | 0.114488 | -1.732571 |
| H | -3.863972 | 1.619963 | -1.872728 |
| H | -4.197102 | 1.848699 | 0.444407 |
| H | -4.506169 | 0.148625 | 0.112210 |
| H | 3.853182 | -1.089355 | -1.933777 |
| H | 2.193507 | -0.957890 | -2.549414 |
| H | -3.882391 | -1.056513 | 1.926980 |
| H | -2.221271 | -0.938530 | 2.541718 |
| C | -1.070728 | -4.209936 | -2.584808 |
| H | -2.665628 | -5.027406 | -0.454742 |
| H | -3.477350 | -3.516603 | 1.348624 |
| H | 3.427144 | -3.545680 | -1.355022 |
| H | 2.602840 | -5.048934 | 0.448949 |
| C | 1.015007 | -4.217297 | 2.578747 |
| N | 1.487454 | -1.356832 | 0.283925 |
| N | -1.519489 | -1.344688 | -0.291239 |
| C | -0.336532 | -1.450722 | -2.326779 |
| O | -0.016622 | -0.203481 | -2.106875 |
| C | 0.040095 | -2.202231 | -3.435420 |
| C | 0.303705 | -1.452204 | 2.319572 |
| O | -0.005997 | -0.202465 | 2.099154 |
| C | -0.078991 | -2.200073 | 3.428593 |
| C | 0.269711 | -3.566113 | 3.537824 |
| C | -0.319937 | -3.565376 | -3.544084 |
| H | 0.006286 | -4.120148 | -4.427022 |
| H | -1.345414 | -5.260380 | -2.693669 |
| H | 0.615188 | -1.722593 | -4.230752 |
| H | -0.061018 | -4.117764 | 4.421041 |
| H | 1.280965 | -5.269944 | 2.688024 |
| H | -0.649999 | -1.715327 | 4.223772 |

KHQLuΔ_λλδ_λλδ_opt.log

SCF (RwB97XD) = -1950.78342069

E(SCF)+ZPE(0 K)= -1950.099461
H(298 K)= -1950.063024
G(298 K)= -1950.161810
Lowest Frequency = 48.7851cm-1

81

| | | | |
|---|---|---|---|
| C | -3.592630 | -0.671526 | 0.761775 |
| N | -2.924282 | -0.531190 | -0.536622 |
| O | -1.672708 | -2.043461 | 1.156123 |
| C | -2.647659 | -1.245459 | 1.824042 |
| C | -3.247581 | -1.641707 | -1.425363 |
| C | -2.163609 | -1.964222 | -2.423124 |
| O | -0.989857 | -2.375499 | -1.734931 |
| C | -0.017338 | -2.879350 | -2.625135 |
| C | 1.208504 | -3.197128 | -1.809888 |
| O | 1.669844 | -2.033703 | -1.154558 |
| O | 0.988233 | -2.376562 | 1.735547 |
| C | 0.017711 | -2.886926 | 2.624204 |
| C | -1.206850 | -3.207053 | 1.807950 |
| N | 2.914770 | -0.520762 | 0.542464 |
| C | 3.242771 | -1.632393 | 1.428127 |
| C | 2.160184 | -1.962288 | 2.424984 |
| C | 2.641520 | -1.229840 | -1.820207 |
| C | 3.583858 | -0.654685 | -0.756258 |
| C | -3.111371 | 0.783854 | -1.134287 |
| C | 3.096578 | 0.793348 | 1.143879 |
| C | 2.404480 | 1.835857 | 0.297518 |
| C | -2.423324 | 1.826736 | -0.285078 |
| C | 0.867709 | 2.196603 | -1.399013 |
| C | 1.175838 | 3.570366 | -1.504366 |
| C | 2.144239 | 4.059511 | -0.587954 |
| C | 2.774495 | 3.200136 | 0.283468 |
| C | -2.798979 | 3.189429 | -0.267096 |
| C | -2.172254 | 4.048913 | 0.606752 |
| C | -1.201808 | 3.561150 | 1.521730 |
| C | -0.887960 | 2.188993 | 1.412395 |
| Lu | -0.004312 | -0.590901 | 0.002763 |
| H | -4.486364 | -1.309810 | 0.660053 |
| H | -3.951955 | 0.304476 | 1.115487 |
| H | -3.202245 | -1.867386 | 2.543483 |
| H | -2.131736 | -0.446076 | 2.377717 |
| H | 2.122324 | -0.431230 | -2.371952 |
| H | 3.198888 | -1.847550 | -2.541132 |
| H | 4.480342 | -1.289359 | -0.656152 |
| H | 3.938991 | 0.323812 | -1.107289 |
| H | -4.182239 | 1.046233 | -1.250456 |
| H | -2.661433 | 0.784570 | -2.137268 |
| H | 4.166383 | 1.059609 | 1.260955 |
| H | 2.646425 | 0.789407 | 2.146770 |
| C | 0.527107 | 4.333118 | -2.510236 |
| H | 2.411171 | 5.118947 | -0.604176 |
| H | 3.554842 | 3.556460 | 0.957618 |
| H | -3.580860 | 3.544430 | -0.940165 |
| H | -2.443594 | 5.107177 | 0.626040 |
| C | -0.556311 | 4.323697 | 2.529831 |
| N | -1.460332 | 1.379506 | 0.490921 |
| N | 1.443423 | 1.386877 | -0.479856 |

| | | | |
|---|---|---|---|
| C | -0.063959 | 1.540653 | -2.273705 |
| O | -0.304382 | 0.274091 | -2.047742 |
| C | -0.643244 | 2.319111 | -3.267895 |
| C | 0.046425 | 1.534439 | 2.285237 |
| O | 0.292179 | 0.269556 | 2.055663 |
| C | 0.622411 | 2.312429 | 3.281710 |
| H | 2.513032 | -2.789531 | 3.065414 |
| H | 1.905036 | -1.101203 | 3.062707 |
| H | 4.187317 | -1.451505 | 1.979794 |
| H | 3.399183 | -2.524601 | 0.807100 |
| H | -2.512963 | -2.791126 | -3.065899 |
| H | -1.912149 | -1.100255 | -3.058404 |
| H | -3.400222 | -2.536294 | -0.806823 |
| H | -4.192878 | -1.463309 | -1.976563 |
| H | 0.212538 | -2.120422 | -3.394454 |
| H | -0.389147 | -3.790540 | -3.127282 |
| H | 2.001159 | -3.629188 | -2.440647 |
| H | 0.961318 | -3.922491 | -1.019536 |
| H | -0.215197 | -2.131229 | 3.395793 |
| H | 0.393112 | -3.798145 | 3.123619 |
| H | -0.956786 | -3.929052 | 1.015423 |
| H | -1.997828 | -3.644127 | 2.437354 |
| C | 0.321386 | 3.691572 | 3.383929 |
| C | -0.347985 | 3.699795 | -3.366121 |
| H | -0.833652 | 4.279239 | -4.154845 |
| H | 0.741963 | 5.398378 | -2.609641 |
| H | -1.332685 | 1.854553 | -3.976396 |
| H | 0.804586 | 4.270748 | 4.174363 |
| H | -0.775639 | 5.387753 | 2.632311 |
| H | 1.313735 | 1.848695 | 3.988914 |

KHQLuΔ_λλλ_λλλ_opt.log

SCF (RwB97XD) = -1950.79364119
E(SCF)+ZPE(0 K)= -1950.109845
H(298 K)= -1950.073407
G(298 K)= -1950.172468
Lowest Frequency = 30.7016cm-1

81

| | | | |
|---|---|---|---|
| C | -3.707962 | 0.861834 | 0.343500 |
| N | -2.673620 | 0.489291 | 1.312664 |
| O | -1.942443 | 1.778771 | -0.985065 |
| C | -3.168603 | 1.062789 | -1.068221 |
| C | -2.538975 | 1.443625 | 2.405604 |
| C | -1.920010 | 2.746863 | 1.933883 |
| O | -0.576456 | 2.575566 | 1.499633 |
| C | 0.397094 | 2.750817 | 2.519004 |
| C | 1.736132 | 2.896079 | 1.842872 |
| O | 1.947656 | 1.787750 | 0.988857 |
| O | 0.577640 | 2.579527 | -1.494938 |
| C | -0.396684 | 2.750961 | -2.514224 |
| C | -1.736488 | 2.888917 | -1.838066 |
| N | 2.685111 | 0.503300 | -1.309637 |
| C | 2.545694 | 1.457849 | -2.401798 |
| C | 1.920399 | 2.757668 | -1.928976 |
| C | 3.177284 | 1.077699 | 1.071633 |

| | | | |
|---|---|---|---|
| C | 3.717624 | 0.880203 | -0.340201 |
| C | -2.773254 | -0.891864 | 1.760301 |
| C | 2.791653 | -0.876970 | -1.758425 |
| C | 2.343528 | -1.842038 | -0.687746 |
| C | -2.320537 | -1.853845 | 0.688767 |
| C | 1.176653 | -2.140253 | 1.292891 |
| C | 1.561498 | -3.495407 | 1.410991 |
| C | 2.361812 | -4.010760 | 0.357418 |
| C | 2.772974 | -3.189718 | -0.665405 |
| C | -2.743500 | -3.203552 | 0.665334 |
| C | -2.328398 | -4.021787 | -0.358149 |
| C | -1.530563 | -3.501748 | -1.411289 |
| C | -1.152240 | -2.144850 | -1.292102 |
| Lu | 0.005567 | 0.593265 | 0.001435 |
| H | -4.195982 | 1.793501 | 0.671487 |
| H | -4.505709 | 0.100525 | 0.306335 |
| H | -3.896571 | 1.633808 | -1.664380 |
| H | -2.979115 | 0.107387 | -1.576382 |
| H | 2.992451 | 0.121113 | 1.579272 |
| H | 3.902459 | 1.651929 | 1.668112 |
| H | 4.201155 | 1.814414 | -0.667609 |
| H | 4.519008 | 0.122707 | -0.303488 |
| H | -3.802199 | -1.155498 | 2.075396 |
| H | -2.124716 | -1.024997 | 2.636005 |
| H | 3.821934 | -1.135243 | -2.073583 |
| H | 2.143915 | -1.012567 | -2.634347 |
| C | 1.159241 | -4.223349 | 2.559978 |
| H | 2.676000 | -5.056950 | 0.385552 |
| H | 3.425614 | -3.561210 | -1.457077 |
| H | -3.394350 | -3.578811 | 1.456702 |
| H | -2.637562 | -5.069449 | -0.387129 |
| C | -1.124801 | -4.226846 | -2.560844 |
| N | -1.511121 | -1.376681 | -0.231668 |
| N | 1.531818 | -1.369523 | 0.233059 |
| C | 0.411096 | -1.468692 | 2.309152 |
| O | 0.074742 | -0.225369 | 2.095102 |
| C | 0.075106 | -2.214010 | 3.435095 |
| C | -0.389904 | -1.468824 | -2.307820 |
| O | -0.059573 | -0.224051 | -2.092816 |
| C | -0.050304 | -2.211635 | -3.434334 |
| H | 0.395096 | 1.875718 | 3.188595 |
| H | 0.179536 | 3.666729 | 3.093458 |
| H | 2.525105 | 2.956824 | 2.607402 |
| H | 1.759445 | 3.817701 | 1.236845 |
| H | -0.390306 | 1.876521 | -3.184647 |
| H | -0.183594 | 3.668488 | -3.087772 |
| H | -1.764484 | 3.809887 | -1.231244 |
| H | -2.525697 | 2.946349 | -2.602607 |
| H | 2.468360 | 3.174756 | -1.073320 |
| H | 1.946565 | 3.505982 | -2.736962 |
| H | 3.515129 | 1.685663 | -2.890349 |
| H | 1.887255 | 1.008233 | -3.157155 |
| H | -1.949750 | 3.494327 | 2.742537 |
| H | -2.470027 | 3.162102 | 1.078650 |
| H | -3.509548 | 1.666281 | 2.894273 |
| H | -1.878389 | 0.996666 | 3.160659 |
| C | 0.441404 | -3.575118 | 3.541348 |

| | | | |
|---|---|---|---|
| C | -0.410056 | -3.574404 | -3.541677 |
| H | -0.112911 | -4.121941 | -4.439235 |
| H | -1.399808 | -5.277594 | -2.665844 |
| H | 0.496830 | -1.724257 | -4.244596 |
| H | 0.146914 | -4.124788 | 4.438477 |
| H | 1.439284 | -5.272849 | 2.664143 |
| H | -0.474339 | -1.729905 | 4.245756 |

KHQTbΔ_δδδ_δδδ_opt.log

SCF (RwB97XD) = -1947.32248925
E(SCF)+ZPE(0 K)= -1946.639177
H(298 K)= -1946.602426
G(298 K)= -1946.703395
Lowest Frequency = 22.0172cm-1

81

| | | | |
|---|---|---|---|
| C | 3.616356 | -0.805667 | -0.474650 |
| N | 2.862090 | -0.552537 | 0.752328 |
| O | 1.700620 | -1.697119 | -1.563616 |
| C | 3.056301 | -1.967973 | -1.264026 |
| C | 3.145161 | -1.555303 | 1.782442 |
| C | 1.891077 | -2.073927 | 2.451207 |
| O | 1.016836 | -2.548978 | 1.419638 |
| C | 0.070769 | -3.516892 | 1.828004 |
| C | -1.137731 | -2.869859 | 2.463625 |
| O | -1.680498 | -1.960807 | 1.524520 |
| O | -0.970428 | -2.551476 | -1.506354 |
| C | 0.045973 | -3.300577 | -2.150646 |
| C | 1.138449 | -2.389849 | -2.665332 |
| N | -2.853589 | -0.591672 | -0.586500 |
| C | -3.182178 | -1.661041 | -1.530407 |
| C | -2.004763 | -2.086996 | -2.377558 |
| C | -3.094093 | -1.931115 | 1.493148 |
| C | -3.565439 | -0.741365 | 0.683582 |
| C | 3.058489 | 0.814685 | 1.233839 |
| C | -3.082852 | 0.739402 | -1.157661 |
| C | -2.446347 | 1.814375 | -0.307419 |
| C | 2.436987 | 1.830848 | 0.304086 |
| C | -0.935648 | 2.254481 | 1.399426 |
| C | -1.282404 | 3.622856 | 1.464437 |
| C | -2.258539 | 4.060710 | 0.530351 |
| C | -2.857977 | 3.165932 | -0.327687 |
| C | 2.865907 | 3.177371 | 0.262141 |
| C | 2.280123 | 4.040467 | -0.634429 |
| C | 1.295687 | 3.573583 | -1.544810 |
| C | 0.931933 | 2.212567 | -1.422261 |
| Tb | -0.010952 | -0.578438 | 0.025991 |
| H | 0.533188 | -4.243014 | 2.517125 |
| H | -0.241324 | -4.051133 | 0.918225 |
| H | -0.876097 | -2.327849 | 3.387983 |
| H | -1.871078 | -3.653772 | 2.715188 |
| H | -0.382038 | -3.904545 | -2.967802 |
| H | 0.459907 | -3.988354 | -1.397074 |
| H | 1.907004 | -2.977552 | -3.190897 |
| H | 0.730356 | -1.636074 | -3.355525 |
| H | 4.134363 | 1.047756 | 1.364499 |

| | | | |
|---|---|---|---|
| H | 2.576462 | 0.903020 | 2.217390 |
| H | -4.165924 | 0.947729 | -1.267839 |
| H | -2.631006 | 0.767615 | -2.158995 |
| C | -0.659441 | 4.433791 | 2.448221 |
| H | -2.554085 | 5.112618 | 0.519749 |
| H | -3.639635 | 3.487189 | -1.017821 |
| H | 3.650424 | 3.518199 | 0.939591 |
| H | 2.588564 | 5.088059 | -0.671311 |
| C | 0.679758 | 4.357903 | -2.553798 |
| N | 1.475408 | 1.400267 | -0.482494 |
| N | -1.486469 | 1.412934 | 0.494811 |
| C | 0.011563 | 1.647144 | 2.297508 |
| O | 0.295973 | 0.384665 | 2.116329 |
| C | 0.566594 | 2.477117 | 3.265912 |
| C | -0.035389 | 1.593799 | -2.291880 |
| O | -0.347067 | 0.344471 | -2.071662 |
| C | -0.587137 | 2.397612 | -3.284197 |
| H | 4.689019 | -0.991696 | -0.268005 |
| H | 3.633358 | -2.069331 | -2.197749 |
| H | 3.133497 | -2.921968 | -0.711801 |
| H | -4.660062 | -0.832116 | 0.534629 |
| H | -3.391688 | 0.164100 | 1.277122 |
| H | -3.495751 | -1.835167 | 2.516157 |
| H | -3.461922 | -2.889122 | 1.082308 |
| H | -1.623412 | -1.250700 | -2.983060 |
| H | -2.317475 | -2.913311 | -3.037636 |
| H | -3.522366 | -2.540979 | -0.967898 |
| H | -4.017767 | -1.373263 | -2.195717 |
| H | 2.148933 | -2.911208 | 3.120523 |
| H | 1.385750 | -1.278445 | 3.018595 |
| H | 3.828746 | -1.161562 | 2.555835 |
| H | 3.663649 | -2.408408 | 1.321566 |
| H | 3.556044 | 0.086424 | -1.111266 |
| C | 0.232109 | 3.850652 | 3.322553 |
| C | -0.230564 | 3.760804 | -3.398307 |
| H | -0.696243 | 4.356031 | -4.187425 |
| H | -1.305250 | 1.959279 | -3.980982 |
| H | 0.943646 | 5.411377 | -2.660359 |
| H | 0.700518 | 4.466750 | 4.093879 |
| H | -0.904312 | 5.495090 | 2.514835 |
| H | 1.266775 | 2.054779 | 3.990227 |

KHQTbΔ_δδλ_δδλ_opt.log

SCF (RwB97XD) = -1947.31813228
E(SCF)+ZPE(0 K)= -1946.635422
H(298 K)= -1946.598476
G(298 K)= -1946.699560
Lowest Frequency = 20.6508cm-1

81

| | | | |
|---|---|---|---|
| C | 3.535530 | 1.013174 | -0.263190 |
| N | 2.647513 | 0.830102 | 0.885829 |
| O | 2.579141 | -0.976034 | -1.200553 |
| C | 3.777489 | -0.235624 | -1.089995 |
| C | 3.201855 | 0.026759 | 1.974021 |
| C | 3.118289 | -1.480430 | 1.772935 |

| | | | |
|---|---|---|---|
| O | 1.777450 | -1.870094 | 1.525634 |
| C | 1.213667 | -2.898537 | 2.322431 |
| C | -0.213317 | -2.507047 | 2.642303 |
| O | -0.917075 | -2.218617 | 1.448574 |
| O | 0.503234 | -2.733179 | -1.268124 |
| C | 1.828881 | -3.203485 | -1.436471 |
| C | 2.664356 | -2.104857 | -2.049263 |
| N | -2.094685 | -1.730972 | -1.075129 |
| C | -1.647374 | -2.410118 | -2.287945 |
| C | -0.485480 | -3.357791 | -2.076152 |
| C | -1.708226 | -3.245529 | 0.891848 |
| C | -2.698560 | -2.605952 | -0.067366 |
| C | 2.216120 | 2.148513 | 1.373794 |
| C | -3.011927 | -0.644329 | -1.435460 |
| C | -3.072413 | 0.434661 | -0.383428 |
| C | 1.258985 | 2.807067 | 0.409104 |
| C | -2.039136 | 1.444087 | 1.431809 |
| C | -3.085638 | 2.380522 | 1.605792 |
| C | -4.162718 | 2.293816 | 0.684860 |
| C | -4.173374 | 1.317254 | -0.282498 |
| C | 1.021051 | 4.199986 | 0.387483 |
| C | 0.159450 | 4.716561 | -0.553974 |
| C | -0.439105 | 3.871567 | -1.525925 |
| C | -0.137125 | 2.494688 | -1.418546 |
| Tb | 0.242539 | -0.446524 | -0.014708 |
| H | 2.612763 | 0.257236 | 2.871692 |
| H | 4.257474 | 0.292579 | 2.187200 |
| H | 3.484730 | -1.963419 | 2.692399 |
| H | 3.750038 | -1.838859 | 0.947726 |
| H | 1.778858 | -3.015535 | 3.260880 |
| H | 1.259180 | -3.856376 | 1.773174 |
| H | -0.215428 | -1.575211 | 3.223877 |
| H | -0.717710 | -3.304782 | 3.210743 |
| H | 1.849528 | -4.103791 | -2.069364 |
| H | 2.226762 | -3.468423 | -0.445033 |
| H | 3.709878 | -2.446220 | -2.135071 |
| H | 2.296151 | -1.838622 | -3.054459 |
| H | -1.311919 | -1.620175 | -2.972040 |
| H | -2.468221 | -2.976869 | -2.773022 |
| H | -0.790074 | -4.309414 | -1.606796 |
| H | -0.065234 | -3.600286 | -3.065887 |
| H | 3.081241 | 2.816595 | 1.553298 |
| H | 1.696334 | 2.005017 | 2.329444 |
| H | -4.031195 | -1.026349 | -1.639090 |
| H | -2.650528 | -0.185052 | -2.365156 |
| C | -3.005611 | 3.308413 | 2.675352 |
| H | -4.995933 | 2.995803 | 0.768856 |
| H | -5.012037 | 1.219441 | -0.973844 |
| H | 1.517006 | 4.848660 | 1.111309 |
| H | -0.047029 | 5.789341 | -0.578923 |
| C | -1.278212 | 4.301497 | -2.586166 |
| N | 0.655776 | 2.004312 | -0.437795 |
| N | -2.040422 | 0.535528 | 0.423678 |
| C | -0.914025 | 1.377199 | 2.329054 |
| O | 0.005876 | 0.482140 | 2.097719 |
| C | -0.893205 | 2.296715 | 3.374451 |
| C | -0.620002 | 1.515326 | -2.357752 |

| | | | |
|---|---|---|---|
| O | -0.294664 | 0.267394 | -2.156073 |
| C | -1.415828 | 1.993417 | -3.394639 |
| H | 3.068845 | 1.761761 | -0.915225 |
| H | 4.520196 | 1.418090 | 0.046033 |
| H | 4.134138 | 0.067629 | -2.089118 |
| H | 4.560888 | -0.876679 | -0.645785 |
| H | -3.314635 | -3.399685 | -0.534348 |
| H | -3.373768 | -1.991478 | 0.544476 |
| H | -2.278115 | -3.753134 | 1.688619 |
| H | -1.068329 | -4.001356 | 0.408074 |
| C | -1.738852 | 3.367445 | -3.488890 |
| H | -1.534106 | 5.357635 | -2.685171 |
| H | -2.372650 | 3.695228 | -4.316423 |
| H | -1.780833 | 1.293110 | -4.149513 |
| C | -1.925879 | 3.248481 | 3.529364 |
| H | -3.799676 | 4.043236 | 2.817765 |
| H | -1.864291 | 3.953067 | 4.362225 |
| H | -0.067302 | 2.263738 | 4.088800 |

KHQTbΔ_δλδ_δλδ_opt.log

SCF (RwB97XD) = -1947.32139573
E(SCF)+ZPE(0 K)= -1946.638010
H(298 K)= -1946.601070
G(298 K)= -1946.702817
Lowest Frequency = 20.3935cm-1

81

| | | | |
|---|---|---|---|
| C | 3.511080 | -0.757346 | -0.795153 |
| N | 2.900927 | -0.530690 | 0.512783 |
| O | 1.614693 | -2.105990 | -1.375789 |
| C | 3.029028 | -2.041249 | -1.435482 |
| C | 3.327785 | -1.527487 | 1.493557 |
| C | 2.248709 | -1.870409 | 2.488205 |
| O | 1.116570 | -2.376595 | 1.789417 |
| C | 0.186940 | -2.941391 | 2.686488 |
| C | -1.061820 | -3.302465 | 1.935182 |
| O | -1.610262 | -2.121277 | 1.377243 |
| O | -1.109743 | -2.380363 | -1.789443 |
| C | -0.175919 | -2.938661 | -2.686235 |
| C | 1.075054 | -3.290965 | -1.934366 |
| N | -2.907599 | -0.550718 | -0.509431 |
| C | -3.326855 | -1.546680 | -1.494453 |
| C | -2.244179 | -1.879671 | -2.488554 |
| C | -3.025252 | -2.069607 | 1.433068 |
| C | -3.517056 | -0.787333 | 0.797100 |
| C | 3.143214 | 0.827697 | 0.998356 |
| C | -3.160247 | 0.807749 | -0.989509 |
| C | -2.460384 | 1.844480 | -0.147149 |
| C | 2.435568 | 1.862276 | 0.159839 |
| C | -0.844397 | 2.243825 | 1.464882 |
| C | -1.172152 | 3.613846 | 1.573437 |
| C | -2.201073 | 4.076783 | 0.711388 |
| C | -2.859750 | 3.200235 | -0.119874 |
| C | 2.824713 | 3.221080 | 0.137289 |
| C | 2.159445 | 4.095519 | -0.690965 |
| C | 1.134125 | 3.627805 | -1.554740 |

| | | | |
|---|---|---|---|
| C | 0.816682 | 2.254985 | -1.450916 |
| Tb | -0.003372 | -0.592380 | 0.002207 |
| H | 4.227739 | 1.056920 | 1.027353 |
| H | 2.763734 | 0.896805 | 2.027849 |
| H | -4.246507 | 1.028776 | -1.017279 |
| H | -2.781839 | 0.883906 | -2.018860 |
| C | -0.479173 | 4.404587 | 2.526333 |
| H | -2.486179 | 5.131305 | 0.735369 |
| H | -3.679263 | 3.537570 | -0.756349 |
| H | 3.641663 | 3.562370 | 0.774949 |
| H | 2.436590 | 5.152237 | -0.711276 |
| C | 0.435349 | 4.416515 | -2.505093 |
| N | 1.435620 | 1.428863 | -0.573794 |
| N | -1.457235 | 1.416039 | 0.585019 |
| C | 0.155116 | 1.619082 | 2.288388 |
| O | 0.415779 | 0.359582 | 2.066811 |
| C | 0.779664 | 2.427395 | 3.232646 |
| C | -0.177957 | 1.625501 | -2.276719 |
| O | -0.429138 | 0.363339 | -2.059387 |
| C | -0.808397 | 2.432263 | -3.218380 |
| H | 4.618082 | -0.790608 | -0.740560 |
| H | 3.357285 | -2.060174 | -2.488934 |
| H | 3.450987 | -2.934557 | -0.943078 |
| H | -4.623692 | -0.829316 | 0.741233 |
| H | -3.254108 | 0.050102 | 1.455562 |
| H | -3.356271 | -2.096002 | 2.485501 |
| H | -3.437738 | -2.964582 | 0.935678 |
| H | -1.932108 | -0.996528 | -3.066944 |
| H | -2.618274 | -2.656604 | -3.177994 |
| H | -3.601477 | -2.474368 | -0.976624 |
| H | -4.230809 | -1.219077 | -2.042324 |
| H | 2.628875 | -2.646776 | 3.174970 |
| H | 1.931714 | -0.990997 | 3.069615 |
| H | 4.230651 | -1.196394 | 2.041171 |
| H | 3.607041 | -2.451713 | 0.972035 |
| H | 0.871976 | -4.018965 | -1.128862 |
| H | 1.792288 | -3.747259 | -2.634717 |
| H | 0.065687 | -2.204817 | -3.475223 |
| H | -0.594206 | -3.845346 | -3.159798 |
| H | 3.240655 | 0.080547 | -1.450032 |
| H | -1.775406 | -3.763871 | 2.635943 |
| H | -0.854026 | -4.028994 | 1.129533 |
| H | 0.611667 | -3.845205 | 3.159808 |
| H | -0.059417 | -2.209243 | 3.475582 |
| C | 0.462066 | 3.801952 | 3.332779 |
| C | -0.501147 | 3.809515 | -3.313784 |
| H | -1.028040 | 4.408608 | -4.060363 |
| H | 0.658554 | 5.480533 | -2.598998 |
| H | -1.547747 | 1.988795 | -3.889321 |
| H | 0.984599 | 4.402477 | 4.081270 |
| H | -0.710382 | 5.466567 | 2.623865 |
| H | 1.522492 | 1.987246 | 3.901932 |

KHQTbΔ_δλλ_δλλ_opt.log

SCF (RwB97XD) = -1947.32430041
E(SCF)+ZPE(0 K)= -1946.640476

H(298 K)= -1946.604000
G(298 K)= -1946.702956
Lowest Frequency = 36.3519cm-1

81

| | | | |
|---|---|---|---|
| C | -3.640949 | -0.727484 | 0.302247 |
| N | -2.766724 | -0.508933 | -0.851164 |
| O | -1.858787 | -1.885106 | 1.406462 |
| C | -3.253983 | -1.891099 | 1.192342 |
| C | -2.915286 | -1.492834 | -1.920653 |
| C | -2.159605 | -2.790233 | -1.670110 |
| O | -0.767238 | -2.580841 | -1.497654 |
| C | 0.008291 | -2.640418 | -2.688345 |
| C | 1.437205 | -2.900053 | -2.295031 |
| O | 1.859348 | -1.892473 | -1.399461 |
| O | 0.768382 | -2.570491 | 1.509237 |
| C | -0.007242 | -2.623488 | 2.700139 |
| C | -1.435884 | -2.886763 | 2.308280 |
| N | 2.766067 | -0.501027 | 0.849201 |
| C | 2.915256 | -1.477549 | 1.925332 |
| C | 2.160950 | -2.777338 | 1.683263 |
| C | 3.254520 | -1.896239 | -1.185167 |
| C | 3.640724 | -0.726694 | -0.302514 |
| C | -2.959813 | 0.859509 | -1.344103 |
| C | 2.957911 | 0.870870 | 1.332922 |
| C | 2.368341 | 1.875881 | 0.371669 |
| C | -2.370690 | 1.871529 | -0.389936 |
| C | 0.958897 | 2.205856 | -1.442247 |
| C | 1.294654 | 3.574562 | -1.555538 |
| C | 2.207834 | 4.074134 | -0.589522 |
| C | 2.761730 | 3.233222 | 0.348199 |
| C | -2.764732 | 3.228816 | -0.375958 |
| C | -2.211165 | 4.076542 | 0.555802 |
| C | -1.297719 | 3.584188 | 1.525282 |
| C | -0.961352 | 2.214878 | 1.421588 |
| Tb | -0.000349 | -0.591258 | -0.000722 |
| H | -4.031905 | 1.086299 | -1.506182 |
| H | -2.451660 | 0.955050 | -2.311671 |
| H | 4.029757 | 1.099600 | 1.493880 |
| H | 2.449263 | 0.972555 | 2.299612 |
| C | 0.724375 | 4.331337 | -2.611489 |
| H | 2.490215 | 5.129382 | -0.616374 |
| H | 3.494201 | 3.599302 | 1.069288 |
| H | -3.497403 | 3.589499 | -1.099560 |
| H | -2.494021 | 5.131826 | 0.575247 |
| C | -0.727742 | 4.348608 | 2.575868 |
| N | -1.467579 | 1.417907 | 0.450464 |
| N | 1.465480 | 1.415920 | -0.465565 |
| C | 0.065501 | 1.549606 | -2.361521 |
| O | -0.231358 | 0.298483 | -2.136539 |
| C | -0.437555 | 2.327202 | -3.400435 |
| C | -0.067640 | 1.565460 | 2.345407 |
| O | 0.229785 | 0.312920 | 2.129178 |
| C | 0.435083 | 2.350558 | 3.378833 |
| H | -4.695612 | -0.864109 | -0.010353 |
| H | -3.779600 | -1.780444 | 2.157060 |
| H | -3.564627 | -2.865140 | 0.774134 |

| | | | |
|---|---|---|---|
| H | 4.695346 | -0.860842 | 0.011295 |
| H | 3.601369 | 0.177269 | -0.922970 |
| H | 3.780174 | -1.791475 | -2.150524 |
| H | 3.565691 | -2.867392 | -0.760685 |
| H | -1.534295 | -3.879321 | 1.830874 |
| H | -2.063366 | -2.871955 | 3.216161 |
| H | 0.075005 | -1.664857 | 3.237022 |
| H | 0.343330 | -3.449871 | 3.339865 |
| H | -3.601714 | 0.180513 | 0.916808 |
| H | 2.064582 | -2.890301 | -3.203053 |
| H | 1.536532 | -3.889559 | -1.811514 |
| H | -0.341653 | -3.470982 | -3.322975 |
| H | -0.074814 | -1.685070 | -3.230950 |
| H | -3.979816 | -1.735835 | -2.115225 |
| H | -2.510622 | -1.043269 | -2.837903 |
| H | -2.328542 | -3.472896 | -2.518832 |
| H | -2.504134 | -3.310502 | -0.770442 |
| H | 2.506163 | -3.303222 | 0.787119 |
| H | 2.330447 | -3.454186 | 2.536517 |
| H | 2.509862 | -1.022227 | 2.839414 |
| H | 3.979969 | -1.718185 | 2.121850 |
| C | -0.110406 | 3.698833 | -3.506946 |
| C | 0.107340 | 3.722765 | 3.475720 |
| H | 0.534371 | 4.301822 | 4.298054 |
| H | -0.969055 | 5.408560 | 2.670888 |
| H | 1.090706 | 1.891110 | 4.122191 |
| H | -0.537670 | 4.271914 | -4.333336 |
| H | 0.965232 | 5.390700 | -2.713949 |
| H | -1.092970 | 1.862252 | -4.140548 |

KHQTbΔ_λδδ_λδδ_opt.log

SCF (RwB97XD) = -1947.32107665
E(SCF)+ZPE(0 K)= -1946.637127
H(298 K)= -1946.600750
G(298 K)= -1946.699164
Lowest Frequency = 46.2203cm-1

81

| | | | |
|---|---|---|---|
| C | 3.709428 | 0.738856 | 0.493944 |
| N | 2.877807 | 0.615225 | -0.716517 |
| O | 1.830466 | 1.859853 | 1.459165 |
| C | 2.913829 | 0.998694 | 1.768652 |
| C | 3.101655 | 1.721825 | -1.649035 |
| C | 1.874360 | 2.120664 | -2.435964 |
| O | 0.873981 | 2.557500 | -1.511046 |
| C | -0.146996 | 3.370156 | -2.065427 |
| C | -1.290844 | 2.530144 | -2.578998 |
| O | -1.819489 | 1.845850 | -1.462277 |
| O | -0.866445 | 2.558185 | 1.505774 |
| C | 0.150406 | 3.377841 | 2.057408 |
| C | 1.298526 | 2.545321 | 2.573616 |
| N | -2.860427 | 0.603170 | 0.717626 |
| C | -3.089893 | 1.711817 | 1.646361 |
| C | -1.864709 | 2.119466 | 2.432098 |
| C | -2.898166 | 0.977807 | -1.768869 |
| C | -3.692571 | 0.718495 | -0.493331 |

| | | | |
|---|---|---|---|
| C | 3.014572 | -0.701059 | -1.341999 |
| C | -2.990903 | -0.711605 | 1.347595 |
| C | -2.403785 | -1.778193 | 0.451859 |
| C | 2.432971 | -1.767485 | -0.442469 |
| C | -0.976711 | -2.197353 | -1.332150 |
| C | -1.314221 | -3.568124 | -1.387108 |
| C | -2.240238 | -4.019208 | -0.408398 |
| C | -2.802674 | -3.134358 | 0.483564 |
| C | 2.838856 | -3.121672 | -0.469462 |
| C | 2.281018 | -4.006305 | 0.425595 |
| C | 1.352731 | -3.556601 | 1.402785 |
| C | 1.008090 | -2.187801 | 1.343039 |
| Tb | 0.008535 | 0.642841 | 0.000655 |
| H | 4.434477 | 1.554455 | 0.352336 |
| H | 4.306119 | -0.173373 | 0.649523 |
| H | 3.570363 | 1.481753 | 2.511786 |
| H | 2.519148 | 0.071278 | 2.210730 |
| H | 0.261328 | 4.006058 | -2.866831 |
| H | -0.507513 | 4.021678 | -1.255247 |
| H | -0.944731 | 1.804247 | -3.335707 |
| H | -2.061958 | 3.179205 | -3.030080 |
| H | -0.261087 | 4.014245 | 2.856787 |
| H | 0.507528 | 4.028576 | 1.245098 |
| H | 2.066438 | 3.199778 | 3.022355 |
| H | 0.956156 | 1.820293 | 3.332856 |
| H | -2.498384 | 0.050871 | -2.207387 |
| H | -3.557119 | 1.454613 | -2.513899 |
| H | -4.421633 | 1.531010 | -0.354607 |
| H | -4.284747 | -0.197200 | -0.645756 |
| H | 4.072729 | -0.938744 | -1.569115 |
| H | 2.460905 | -0.699427 | -2.291684 |
| H | -4.047954 | -0.953711 | 1.575193 |
| H | -2.437618 | -0.703968 | 2.297456 |
| C | -0.733098 | -4.369288 | -2.403577 |
| H | -2.526568 | -5.073537 | -0.389530 |
| H | -3.545406 | -3.465854 | 1.210778 |
| H | 3.583286 | -3.451851 | -1.195538 |
| H | 2.572802 | -5.059197 | 0.410395 |
| C | 0.775884 | -4.357205 | 2.422126 |
| N | 1.516100 | -1.355351 | 0.403831 |
| N | -1.489063 | -1.364266 | -0.395880 |
| C | -0.083231 | -1.578635 | -2.276374 |
| O | 0.191385 | -0.309605 | -2.112190 |
| C | 0.432086 | -2.399057 | -3.273759 |
| C | 0.111471 | -1.570441 | 2.285181 |
| O | -0.169742 | -0.303433 | 2.116637 |
| C | -0.399493 | -2.390036 | 3.285480 |
| H | -2.133670 | 2.960381 | 3.092399 |
| H | -1.463372 | 1.290972 | 3.035669 |
| H | -3.909379 | 1.489079 | 2.357539 |
| H | -3.399571 | 2.587044 | 1.060086 |
| H | 2.139002 | 2.960644 | -3.099196 |
| H | 1.477208 | 1.288068 | -3.036617 |
| H | 3.407074 | 2.600564 | -1.065787 |
| H | 3.922137 | 1.500736 | -2.359582 |
| C | -0.069574 | -3.764633 | 3.335322 |
| C | 0.109346 | -3.775525 | -3.318784 |

| | | | |
|---|---|---|---|
| H | 0.545152 | -4.384803 | -4.114173 |
| H | -0.971842 | -5.432455 | -2.462621 |
| H | 1.093016 | -1.966773 | -4.028334 |
| H | -0.502121 | -4.373371 | 4.132899 |
| H | 1.020193 | -5.418893 | 2.484893 |
| H | -1.062609 | -1.958545 | 4.038589 |

KHQTbΔ_λδλ_λδλ_opt.log

SCF (RwB97XD) = -1947.32864350
E(SCF)+ZPE(0 K)= -1946.645614
H(298 K)= -1946.608924
G(298 K)= -1946.708946
Lowest Frequency = 26.8304cm-1

81

| | | | |
|---|---|---|---|
| C | 3.681570 | 0.930091 | -0.187812 |
| N | 2.663364 | 0.558819 | -1.182783 |
| O | 1.967958 | 1.899729 | 1.146876 |
| C | 3.142962 | 1.107710 | 1.223114 |
| C | 2.588799 | 1.505637 | -2.294787 |
| C | 1.977649 | 2.831191 | -1.884822 |
| O | 0.633302 | 2.627543 | -1.467897 |
| C | -0.385327 | 3.393690 | -2.085629 |
| C | -1.561256 | 2.495860 | -2.380703 |
| O | -1.969947 | 1.919130 | -1.154750 |
| O | -0.628307 | 2.633579 | 1.460077 |
| C | 0.397775 | 3.389653 | 2.077887 |
| C | 1.564946 | 2.480412 | 2.372844 |
| N | -2.678504 | 0.584874 | 1.174831 |
| C | -2.594734 | 1.530859 | 2.286885 |
| C | -1.970603 | 2.850365 | 1.876966 |
| C | -3.152625 | 1.138606 | -1.231041 |
| C | -3.692993 | 0.966152 | 0.179851 |
| C | 2.811007 | -0.818281 | -1.653160 |
| C | -2.839644 | -0.790743 | 1.645144 |
| C | -2.390606 | -1.791917 | 0.609416 |
| C | 2.352402 | -1.814981 | -0.617306 |
| C | -1.159737 | -2.146289 | -1.326063 |
| C | -1.531222 | -3.507890 | -1.408860 |
| C | -2.369044 | -3.990004 | -0.367711 |
| C | -2.818458 | -3.140259 | 0.615823 |
| C | 2.767304 | -3.167365 | -0.623541 |
| C | 2.309690 | -4.012664 | 0.360046 |
| C | 1.476462 | -3.522431 | 1.401073 |
| C | 1.118141 | -2.157317 | 1.318168 |
| Tb | -0.007222 | 0.643893 | -0.003965 |
| H | 4.159683 | 1.873175 | -0.495196 |
| H | 4.487798 | 0.178163 | -0.155201 |
| H | 3.900266 | 1.626858 | 1.835124 |
| H | 2.906719 | 0.146964 | 1.705845 |
| H | 1.939098 | 1.062224 | -3.061680 |
| H | 3.582839 | 1.694264 | -2.748277 |
| H | 2.000776 | 3.519566 | -2.743409 |
| H | 2.530297 | 3.303666 | -1.057750 |
| H | -0.021596 | 3.829488 | -3.029184 |
| H | -0.684070 | 4.215422 | -1.412696 |

| | | | |
|---|---|---|---|
| H | -1.271384 | 1.697216 | -3.084488 |
| H | -2.382734 | 3.094098 | -2.811845 |
| H | 0.038336 | 3.828849 | 3.021512 |
| H | 0.704484 | 4.208530 | 1.405056 |
| H | 2.392215 | 3.070662 | 2.803927 |
| H | 1.267430 | 1.684621 | 3.076666 |
| H | -1.949482 | 1.081011 | 3.053789 |
| H | -3.586911 | 1.729202 | 2.740311 |
| H | -2.518541 | 3.328165 | 1.049817 |
| H | -1.987051 | 3.538963 | 2.735529 |
| H | -2.925726 | 0.175630 | -1.713791 |
| H | -3.904819 | 1.665125 | -1.843057 |
| H | -4.161847 | 1.913851 | 0.487307 |
| H | -4.506534 | 0.222145 | 0.147151 |
| H | 3.854884 | -1.041788 | -1.947895 |
| H | 2.185439 | -0.946612 | -2.546672 |
| H | -3.885722 | -1.004052 | 1.939639 |
| H | -2.215630 | -0.925269 | 2.538814 |
| C | -1.073885 | -4.273915 | -2.511344 |
| H | -2.677488 | -5.038294 | -0.372137 |
| H | -3.492916 | -3.491998 | 1.398118 |
| H | 3.438417 | -3.525626 | -1.405753 |
| H | 2.608071 | -5.063862 | 0.364594 |
| C | 1.011695 | -4.283954 | 2.503569 |
| N | 1.523384 | -1.363324 | 0.296135 |
| N | -1.557299 | -1.348363 | -0.304072 |
| C | -0.357893 | -1.500945 | -2.333318 |
| O | -0.045265 | -0.245321 | -2.155445 |
| C | 0.036914 | -2.288231 | -3.411446 |
| C | 0.322550 | -1.504212 | 2.325383 |
| O | 0.022156 | -0.245616 | 2.147475 |
| C | -0.079885 | -2.287585 | 3.403534 |
| C | 0.258530 | -3.658305 | 3.473662 |
| C | -0.314752 | -3.655616 | -3.481502 |
| H | 0.025307 | -4.236185 | -4.342413 |
| H | -1.341385 | -5.328930 | -2.589718 |
| H | 0.620164 | -1.831101 | -4.214111 |
| H | -0.087159 | -4.235522 | 4.334585 |
| H | 1.269001 | -5.341500 | 2.582003 |
| H | -0.658702 | -1.824793 | 4.206164 |

KHQTbΔ_λλδ_λλδ_opt.log

SCF (RwB97XD) = -1947.31682136
E(SCF)+ZPE(0 K)= -1946.632858
H(298 K)= -1946.596477
G(298 K)= -1946.694855
Lowest Frequency = 46.9865cm-1

81

| | | | |
|---|---|---|---|
| C | -3.580914 | -0.720875 | 0.750610 |
| N | -2.880761 | -0.568829 | -0.534946 |
| O | -1.728052 | -2.161444 | 1.205420 |
| C | -2.671585 | -1.298866 | 1.836332 |
| C | -3.210661 | -1.672110 | -1.437055 |
| C | -2.153908 | -1.957654 | -2.474788 |
| O | -0.961637 | -2.381363 | -1.827985 |

| | | | |
|---|---|---|---|
| C | 0.012531 | -2.816269 | -2.753780 |
| C | 1.229580 | -3.231447 | -1.967066 |
| O | 1.726342 | -2.149560 | -1.204939 |
| O | 0.960854 | -2.382693 | 1.827970 |
| C | -0.011290 | -2.825455 | 2.752151 |
| C | -1.226339 | -3.243584 | 1.963917 |
| N | 2.871983 | -0.557481 | 0.540829 |
| C | 3.206646 | -1.661859 | 1.439819 |
| C | 2.150908 | -1.955226 | 2.476409 |
| C | 2.666239 | -1.280811 | -1.832816 |
| C | 3.572979 | -0.702606 | -0.745059 |
| C | -3.097551 | 0.748606 | -1.129853 |
| C | 3.082678 | 0.759154 | 1.139637 |
| C | 2.416395 | 1.819245 | 0.294516 |
| C | -2.435856 | 1.809227 | -0.281792 |
| C | 0.883842 | 2.214279 | -1.401041 |
| C | 1.191783 | 3.590725 | -1.473997 |
| C | 2.162149 | 4.058604 | -0.547881 |
| C | 2.790140 | 3.182633 | 0.308703 |
| C | -2.815684 | 3.170969 | -0.291915 |
| C | -2.191511 | 4.047197 | 0.567195 |
| C | -1.218973 | 3.580924 | 1.491845 |
| C | -0.904920 | 2.206089 | 1.414803 |
| Tb | -0.004313 | -0.641198 | 0.002645 |
| H | -4.471059 | -1.358488 | 0.618851 |
| H | -3.948608 | 0.252663 | 1.102225 |
| H | -3.263432 | -1.869351 | 2.569611 |
| H | -2.129493 | -0.500894 | 2.368977 |
| H | 2.120788 | -0.483391 | -2.362842 |
| H | 3.260606 | -1.846287 | -2.567934 |
| H | 4.465650 | -1.337056 | -0.615156 |
| H | 3.936851 | 0.273508 | -1.093514 |
| H | -4.175273 | 0.979052 | -1.246316 |
| H | -2.645821 | 0.762705 | -2.131915 |
| H | 4.159318 | 0.994131 | 1.257066 |
| H | 2.630611 | 0.768242 | 2.141625 |
| C | 0.538097 | 4.378890 | -2.456394 |
| H | 2.429307 | 5.118096 | -0.541986 |
| H | 3.566503 | 3.526387 | 0.993897 |
| H | -3.593677 | 3.513275 | -0.975986 |
| H | -2.463413 | 5.105495 | 0.564464 |
| C | -0.568756 | 4.369055 | 2.476568 |
| N | -1.479679 | 1.379689 | 0.510661 |
| N | 1.462240 | 1.387755 | -0.499324 |
| C | -0.053220 | 1.578725 | -2.287414 |
| O | -0.294368 | 0.307005 | -2.097309 |
| C | -0.639960 | 2.385439 | -3.255721 |
| C | 0.034874 | 1.572027 | 2.299343 |
| O | 0.281556 | 0.301934 | 2.105499 |
| C | 0.618029 | 2.378441 | 3.270062 |
| H | 2.517102 | -2.765792 | 3.130542 |
| H | 1.916636 | -1.074988 | 3.095916 |
| H | 4.170778 | -1.484173 | 1.956844 |
| H | 3.327229 | -2.566726 | 0.829500 |
| H | -2.516533 | -2.768068 | -3.131096 |
| H | -1.924053 | -1.074591 | -3.091905 |
| H | -3.326976 | -2.579269 | -0.829323 |

H -4.175734 -1.497297 -1.953301
H 0.253203 -1.994422 -3.451848
H -0.368156 -3.674153 -3.336525
H 2.010730 -3.625803 -2.636645
H 0.960713 -4.019645 -1.247755
H -0.255868 -2.007271 3.453158
H 0.373409 -3.683590 3.331885
H -0.953767 -4.028075 1.241953
H -2.005705 -3.643851 2.632070
C 0.316278 3.759068 3.339536
C -0.344276 3.767572 -3.321141
H -0.835722 4.367160 -4.091079
H 0.752918 5.446230 -2.530335
H -1.335959 1.939878 -3.970066
H 0.805059 4.358533 4.111265
H -0.788288 5.435211 2.553679
H 1.315921 1.933830 3.983150

KHQTbΔ_λλλ_λλλ_opt.log

SCF (RwB97XD) = -1947.32407866
E(SCF)+ZPE(0 K)= -1946.640620
H(298 K)= -1946.604105
G(298 K)= -1946.703450
Lowest Frequency = 27.4277cm-1

81
C -3.686580 0.908828 0.359022
N -2.634950 0.518428 1.309526
O -1.978497 1.872545 -1.004394
C -3.185809 1.123181 -1.063482
C -2.510281 1.452027 2.427698
C -1.899946 2.774300 1.998116
O -0.551252 2.628000 1.573577
C 0.419245 2.734270 2.606406
C 1.762739 2.924014 1.945883
O 1.979579 1.884728 1.007728
O 0.547482 2.632554 -1.570093
C -0.423775 2.732941 -2.602779
C -1.768394 2.913691 -1.942047
N 2.644743 0.536436 -1.307007
C 2.514010 1.469620 -2.424815
C 1.895149 2.787748 -1.994687
C 3.191630 1.142996 1.066314
C 3.693828 0.933275 -0.356350
C -2.762521 -0.865527 1.758188
C 2.781321 -0.846478 -1.756226
C 2.360625 -1.829098 -0.691011
C -2.335108 -1.844952 0.692693
C 1.198673 -2.163448 1.289533
C 1.569381 -3.525792 1.369778
C 2.367026 -4.019859 0.303315
C 2.782111 -3.179589 -0.702402
C -2.747244 -3.198332 0.703750
C -2.326344 -4.035463 -0.302170
C -1.532153 -3.535627 -1.368514
C -1.170894 -2.170772 -1.287940

| | | | |
|---|---|---|---|
| Tb | 0.004528 | 0.632790 | 0.001302 |
| H | -4.160649 | 1.840396 | 0.706229 |
| H | -4.489213 | 0.152583 | 0.331894 |
| H | -3.945515 | 1.676995 | -1.636686 |
| H | -2.985961 | 0.171971 | -1.577271 |
| H | 2.997705 | 0.189924 | 1.578951 |
| H | 3.947701 | 1.701022 | 1.640234 |
| H | 4.161358 | 1.868407 | -0.702834 |
| H | 4.501665 | 0.182557 | -0.329955 |
| H | -3.795293 | -1.100242 | 2.082337 |
| H | -2.110365 | -1.009813 | 2.629726 |
| H | 3.815535 | -1.074257 | -2.080720 |
| H | 2.129876 | -0.994690 | -2.627645 |
| C | 1.152144 | -4.283737 | 2.493485 |
| H | 2.671849 | -5.069206 | 0.305358 |
| H | 3.425864 | -3.539559 | -1.506554 |
| H | -3.388476 | -3.562954 | 1.507821 |
| H | -2.623875 | -5.086900 | -0.304451 |
| C | -1.109658 | -4.290404 | -2.492390 |
| N | -1.540745 | -1.382800 | -0.246615 |
| N | 1.563081 | -1.372676 | 0.248403 |
| C | 0.431573 | -1.512986 | 2.320334 |
| O | 0.108200 | -0.259955 | 2.145788 |
| C | 0.076714 | -2.291763 | 3.418391 |
| C | -0.408240 | -1.514785 | -2.318546 |
| O | -0.093428 | -0.259622 | -2.143613 |
| C | -0.048024 | -2.290812 | -3.416800 |
| H | 0.414810 | 1.816208 | 3.216171 |
| H | 0.200216 | 3.609979 | 3.240314 |
| H | 2.548306 | 2.925047 | 2.716523 |
| H | 1.792062 | 3.887386 | 1.410153 |
| H | -0.413469 | 1.815174 | -3.212915 |
| H | -0.210508 | 3.610304 | -3.236368 |
| H | -1.803890 | 3.876585 | -1.405829 |
| H | -2.554010 | 2.910040 | -2.712625 |
| H | 2.434507 | 3.223057 | -1.142393 |
| H | 1.938045 | 3.512450 | -2.823709 |
| H | 3.488131 | 1.673890 | -2.913335 |
| H | 1.857331 | 1.003095 | -3.171560 |
| H | -1.947601 | 3.498402 | 2.827401 |
| H | -2.442061 | 3.206392 | 1.145937 |
| H | -3.485728 | 1.649816 | 2.916237 |
| H | -1.850644 | 0.989500 | 3.174313 |
| C | 0.430086 | -3.658469 | 3.486850 |
| C | -0.391938 | -3.659916 | -3.485595 |
| H | -0.079504 | -4.231347 | -4.362897 |
| H | -1.371693 | -5.347012 | -2.567695 |
| H | 0.502839 | -1.822975 | -4.236059 |
| H | 0.121631 | -4.232256 | 4.364023 |
| H | 1.421506 | -5.338517 | 2.568547 |
| H | -0.477336 | -1.827931 | 4.237777 |

KHQLaΔ_δδδ_δδδ_opt.log

SCF (RwB97XD) = -1942.42487633
E(SCF)+ZPE(0 K)= -1941.742882
H(298 K)= -1941.705709

G(298 K)= -1941.807554
Lowest Frequency = 24.3228cm-1

81

| | | | |
|---|---|---|---|
| C | 3.615262 | -0.868898 | -0.581880 |
| N | 2.872669 | -0.649267 | 0.663077 |
| O | 1.724271 | -1.946735 | -1.585609 |
| C | 3.121764 | -2.058031 | -1.383840 |
| C | 3.194407 | -1.662990 | 1.674037 |
| C | 2.017845 | -2.042519 | 2.547281 |
| O | 1.014075 | -2.615544 | 1.706190 |
| C | 0.021148 | -3.379956 | 2.367233 |
| C | -1.171126 | -2.533789 | 2.760729 |
| O | -1.719902 | -1.960899 | 1.582641 |
| O | -1.006483 | -2.613781 | -1.711341 |
| C | -0.010040 | -3.371588 | -2.374644 |
| C | 1.178419 | -2.518837 | -2.765434 |
| N | -2.874801 | -0.660776 | -0.661176 |
| C | -3.191417 | -1.671962 | -1.676275 |
| C | -2.012768 | -2.042444 | -2.550593 |
| C | -3.116681 | -2.079044 | 1.379914 |
| C | -3.616335 | -0.889212 | 0.582832 |
| C | 3.099272 | 0.708101 | 1.176515 |
| C | -3.108053 | 0.697563 | -1.168993 |
| C | -2.504255 | 1.762052 | -0.280721 |
| C | 2.489936 | 1.773376 | 0.292973 |
| C | -0.978731 | 2.239369 | 1.408729 |
| C | -1.327838 | 3.610484 | 1.439451 |
| C | -2.323031 | 4.022928 | 0.513580 |
| C | -2.925375 | 3.111092 | -0.321970 |
| C | 2.904161 | 3.124355 | 0.340103 |
| C | 2.297018 | 4.036775 | -0.491335 |
| C | 1.303737 | 3.623345 | -1.418817 |
| C | 0.961731 | 2.250325 | -1.394157 |
| La | -0.000875 | -0.686095 | 0.000945 |
| H | 0.446720 | -3.876639 | 3.254994 |
| H | -0.299561 | -4.158965 | 1.658879 |
| H | -0.875024 | -1.714996 | 3.436547 |
| H | -1.915296 | -3.168129 | 3.267739 |
| H | -0.433249 | -3.867486 | -3.263973 |
| H | 0.314167 | -4.151284 | -1.668645 |
| H | 1.925596 | -3.148421 | -3.273955 |
| H | 0.878828 | -1.699630 | -3.439204 |
| H | 4.181869 | 0.908034 | 1.305729 |
| H | 2.624597 | 0.779477 | 2.166193 |
| H | -4.191598 | 0.892658 | -1.297657 |
| H | -2.633453 | 0.775484 | -2.158220 |
| C | -0.684188 | 4.460643 | 2.374265 |
| H | -2.621111 | 5.073761 | 0.484991 |
| H | -3.708969 | 3.417608 | -1.016702 |
| H | 3.686305 | 3.431814 | 1.036053 |
| H | 2.589727 | 5.088982 | -0.458154 |
| C | 0.655468 | 4.474311 | -2.349700 |
| N | 1.535645 | 1.385881 | -0.521945 |
| N | -1.548080 | 1.375852 | 0.532601 |
| C | 0.005442 | 1.673510 | 2.300387 |
| O | 0.307003 | 0.409303 | 2.172467 |

| | | | |
|---|---|---|---|
| C | 0.585819 | 2.547498 | 3.216118 |
| C | -0.019565 | 1.683292 | -2.288233 |
| O | -0.314386 | 0.416950 | -2.165937 |
| C | -0.604638 | 2.558323 | -3.199969 |
| H | 4.699142 | -1.000175 | -0.390454 |
| H | 3.646997 | -2.055454 | -2.352605 |
| H | 3.340615 | -3.020417 | -0.888468 |
| H | -4.699517 | -1.025255 | 0.390799 |
| H | -3.505156 | 0.003281 | 1.211207 |
| H | -3.642247 | -2.083534 | 2.348487 |
| H | -3.330084 | -3.040446 | 0.880268 |
| H | -1.601297 | -1.161726 | -3.069024 |
| H | -2.337835 | -2.793612 | -3.289918 |
| H | -3.528401 | -2.588441 | -1.172373 |
| H | -4.027903 | -1.343178 | -2.321223 |
| H | 2.346827 | -2.794917 | 3.283618 |
| H | 1.602210 | -1.165814 | 3.069217 |
| H | 4.029567 | -1.332820 | 2.319997 |
| H | 3.535533 | -2.575866 | 1.166401 |
| H | 3.499546 | 0.025531 | -1.206649 |
| C | 0.242195 | 3.917786 | 3.238348 |
| C | -0.268210 | 3.930484 | -3.216067 |
| H | -0.761258 | 4.578266 | -3.944988 |
| H | -1.335307 | 2.165574 | -3.910814 |
| H | 0.902390 | 5.536833 | -2.379346 |
| H | 0.731735 | 4.564858 | 3.970256 |
| H | -0.936671 | 5.521716 | 2.408621 |
| H | 1.318479 | 2.155386 | 3.925264 |

KHQLaΔ_δδλ_δδλ_opt.log

SCF (RwB97XD) = -1942.41778323
E(SCF)+ZPE(0 K)= -1941.736471
H(298 K)= -1941.699058
G(298 K)= -1941.802644
Lowest Frequency = 7.7884cm-1

81

| | | | |
|---|---|---|---|
| C | 3.612149 | 0.928243 | -0.329588 |
| N | 2.712329 | 0.820530 | 0.821759 |
| O | 2.685571 | -1.111891 | -1.192764 |
| C | 3.882588 | -0.370125 | -1.068845 |
| C | 3.242803 | 0.052164 | 1.951778 |
| C | 3.136455 | -1.465602 | 1.844161 |
| O | 1.790910 | -1.848112 | 1.624101 |
| C | 1.224360 | -2.869556 | 2.424448 |
| C | -0.225689 | -2.506490 | 2.680590 |
| O | -0.908124 | -2.266817 | 1.464858 |
| O | 0.517517 | -2.738762 | -1.411323 |
| C | 1.814761 | -3.299649 | -1.482108 |
| C | 2.779716 | -2.261679 | -2.010548 |
| N | -2.109485 | -1.775434 | -1.074085 |
| C | -1.669960 | -2.408010 | -2.319038 |
| C | -0.507334 | -3.367738 | -2.164761 |
| C | -1.643998 | -3.314964 | 0.874516 |
| C | -2.662260 | -2.697710 | -0.075545 |
| C | 2.325274 | 2.171331 | 1.266546 |

| | | | |
|---|---|---|---|
| C | -3.072225 | -0.710122 | -1.393175 |
| C | -3.202453 | 0.323651 | -0.299599 |
| C | 1.433630 | 2.881239 | 0.273923 |
| C | -2.188042 | 1.418331 | 1.483981 |
| C | -3.308717 | 2.260043 | 1.697705 |
| C | -4.414617 | 2.066406 | 0.828936 |
| C | -4.376762 | 1.097194 | -0.145542 |
| C | 1.303955 | 4.288461 | 0.244071 |
| C | 0.447647 | 4.859198 | -0.669964 |
| C | -0.267333 | 4.053766 | -1.595178 |
| C | -0.059496 | 2.656017 | -1.496671 |
| La | 0.247523 | -0.411175 | -0.066953 |
| H | 2.655334 | 0.342871 | 2.833363 |
| H | 4.301681 | 0.310825 | 2.157314 |
| H | 3.496694 | -1.888295 | 2.796089 |
| H | 3.764716 | -1.887146 | 1.045896 |
| H | 1.751310 | -2.944100 | 3.389981 |
| H | 1.316732 | -3.843400 | 1.909236 |
| H | -0.267036 | -1.556818 | 3.233355 |
| H | -0.726530 | -3.297342 | 3.262633 |
| H | 1.825692 | -4.188029 | -2.132806 |
| H | 2.115613 | -3.609655 | -0.467890 |
| H | 3.802795 | -2.675047 | -1.980006 |
| H | 2.541002 | -1.994966 | -3.054552 |
| H | -1.347243 | -1.590256 | -2.979036 |
| H | -2.497454 | -2.954459 | -2.816002 |
| H | -0.799538 | -4.313904 | -1.676159 |
| H | -0.136820 | -3.617174 | -3.173064 |
| H | 3.215964 | 2.792875 | 1.483543 |
| H | 1.748877 | 2.056466 | 2.195423 |
| H | -4.067479 | -1.129099 | -1.637948 |
| H | -2.702644 | -0.191010 | -2.289083 |
| C | -3.267979 | 3.210989 | 2.748189 |
| H | -5.305616 | 2.686583 | 0.954405 |
| H | -5.231740 | 0.926661 | -0.802041 |
| H | 1.872905 | 4.904947 | 0.942021 |
| H | 0.322977 | 5.944414 | -0.701727 |
| C | -1.145343 | 4.547241 | -2.593242 |
| N | 0.754344 | 2.120082 | -0.554497 |
| N | -2.158019 | 0.508645 | 0.476775 |
| C | -1.021896 | 1.469585 | 2.335594 |
| O | -0.038486 | 0.643298 | 2.107125 |
| C | -1.037041 | 2.420953 | 3.353270 |
| C | -0.688635 | 1.716622 | -2.395253 |
| O | -0.441943 | 0.444445 | -2.238072 |
| C | -1.534150 | 2.257219 | -3.360749 |
| H | 3.146787 | 1.628067 | -1.035653 |
| H | 4.589938 | 1.361714 | -0.036848 |
| H | 4.286678 | -0.123929 | -2.065787 |
| H | 4.641630 | -0.986769 | -0.553925 |
| H | -3.243801 | -3.509692 | -0.555103 |
| H | -3.363241 | -2.126516 | 0.549250 |
| H | -2.194118 | -3.874506 | 1.650654 |
| H | -0.964987 | -4.024491 | 0.372928 |
| C | -1.754081 | 3.650532 | -3.444603 |
| H | -1.323664 | 5.620332 | -2.679331 |
| H | -2.427750 | 4.024562 | -4.219347 |

| | | | |
|---|---|---|---|
| H | -2.018205 | 1.583159 | -4.071052 |
| C | -2.144570 | 3.275652 | 3.543616 |
| H | -4.120587 | 3.870787 | 2.917065 |
| H | -2.107042 | 4.004346 | 4.356979 |
| H | -0.175374 | 2.483945 | 4.021705 |

KHQLaΔ_δλδ_δλδ_opt.log

SCF (RwB97XD) = -1942.42984924
E(SCF)+ZPE(0 K)= -1941.747045
H(298 K)= -1941.710126
G(298 K)= -1941.810850
Lowest Frequency = 35.9667cm-1

81

| | | | |
|---|---|---|---|
| C | 3.572031 | -0.920584 | -0.782997 |
| N | 2.919751 | -0.654813 | 0.500345 |
| O | 1.675162 | -2.158917 | -1.555141 |
| C | 3.086156 | -2.189928 | -1.449872 |
| C | 3.316118 | -1.637502 | 1.511560 |
| C | 2.278767 | -1.850609 | 2.585190 |
| O | 1.095797 | -2.378410 | 1.996568 |
| C | 0.146004 | -2.713721 | 2.987315 |
| C | -1.113221 | -3.214865 | 2.336386 |
| O | -1.672724 | -2.173708 | 1.555940 |
| O | -1.092019 | -2.379935 | -1.996113 |
| C | -0.140221 | -2.706315 | -2.987934 |
| C | 1.122244 | -3.201320 | -2.338604 |
| N | -2.926713 | -0.671814 | -0.495291 |
| C | -3.317012 | -1.654455 | -1.508888 |
| C | -2.278533 | -1.858441 | -2.583195 |
| C | -3.083523 | -2.212936 | 1.450967 |
| C | -3.577181 | -0.944825 | 0.787454 |
| C | 3.201695 | 0.709411 | 0.960096 |
| C | -3.217066 | 0.691788 | -0.951643 |
| C | -2.563529 | 1.752308 | -0.098760 |
| C | 2.541382 | 1.768075 | 0.110124 |
| C | -0.911863 | 2.256113 | 1.453551 |
| C | -1.278616 | 3.621504 | 1.514273 |
| C | -2.358313 | 4.016747 | 0.681270 |
| C | -3.012091 | 3.093129 | -0.100099 |
| C | 2.981368 | 3.111727 | 0.115238 |
| C | 2.321738 | 4.033332 | -0.663577 |
| C | 1.244637 | 3.633531 | -1.497766 |
| C | 0.886585 | 2.265669 | -1.440857 |
| La | -0.003328 | -0.687069 | 0.002337 |
| H | 4.293900 | 0.894540 | 1.002495 |
| H | 2.806150 | 0.812583 | 1.980990 |
| H | -4.310390 | 0.870229 | -0.993862 |
| H | -2.821822 | 0.800063 | -1.972143 |
| C | -0.567536 | 4.484106 | 2.386813 |
| H | -2.677054 | 5.061868 | 0.680432 |
| H | -3.858085 | 3.383830 | -0.724987 |
| H | 3.825452 | 3.406077 | 0.741001 |
| H | 2.633788 | 5.080463 | -0.659778 |
| C | 0.528108 | 4.494020 | -2.367931 |
| N | 1.517613 | 1.388923 | -0.620442 |

| | | | |
|---|---|---|---|
| N | -1.537332 | 1.377655 | 0.630719 |
| C | 0.153528 | 1.709707 | 2.256936 |
| O | 0.453068 | 0.448776 | 2.107788 |
| C | 0.801969 | 2.594058 | 3.114936 |
| C | -0.175316 | 1.714745 | -2.245774 |
| O | -0.466933 | 0.451555 | -2.100038 |
| C | -0.829326 | 2.597329 | -3.101365 |
| H | 4.673634 | -0.985426 | -0.674991 |
| H | 3.532537 | -2.244340 | -2.457794 |
| H | 3.394855 | -3.100418 | -0.906464 |
| H | -4.678389 | -1.016041 | 0.679448 |
| H | -3.367537 | -0.104031 | 1.461544 |
| H | -3.529305 | -2.272611 | 2.458857 |
| H | -3.386902 | -3.123863 | 0.905296 |
| H | -2.027806 | -0.917104 | -3.097166 |
| H | -2.667442 | -2.582414 | -3.320796 |
| H | -3.483563 | -2.624033 | -1.021780 |
| H | -4.275887 | -1.375449 | -1.987272 |
| H | 2.672148 | -2.573734 | 3.321246 |
| H | 2.021836 | -0.912002 | 3.101132 |
| H | 4.273160 | -1.353628 | 1.990742 |
| H | 3.488835 | -2.604811 | 1.022086 |
| H | 0.929959 | -4.087183 | -1.706779 |
| H | 1.828859 | -3.494056 | -3.132620 |
| H | 0.081529 | -1.808315 | -3.591377 |
| H | -0.539514 | -3.494552 | -3.651298 |
| H | 3.357451 | -0.079332 | -1.454944 |
| H | -1.818020 | -3.514480 | 3.129446 |
| H | -0.915211 | -4.097606 | 1.701962 |
| H | 0.550244 | -3.501316 | 3.648447 |
| H | -0.081553 | -1.818922 | 3.593344 |
| C | 0.441631 | 3.959212 | 3.164810 |
| C | -0.477675 | 3.964879 | -3.147433 |
| H | -1.025161 | 4.620446 | -3.828960 |
| H | 0.786580 | 5.552937 | -2.419516 |
| H | -1.624893 | 2.214973 | -3.745099 |
| H | 0.984952 | 4.616347 | 3.848157 |
| H | -0.832762 | 5.541202 | 2.441378 |
| H | 1.599956 | 2.214980 | 3.757612 |

KHQLaΔ_δλλ_δλλ_opt.log

SCF (RwB97XD) = -1942.42664428
E(SCF)+ZPE(0 K)= -1941.744085
H(298 K)= -1941.707200
G(298 K)= -1941.807923
Lowest Frequency = 21.5255cm-1

81

| | | | |
|---|---|---|---|
| C | -3.646701 | -0.860755 | 0.291893 |
| N | -2.776755 | -0.560024 | -0.850464 |
| O | -1.866138 | -1.976698 | 1.450495 |
| C | -3.243886 | -2.047157 | 1.149504 |
| C | -2.911901 | -1.476763 | -1.986101 |
| C | -2.190335 | -2.809206 | -1.829015 |
| O | -0.794023 | -2.664548 | -1.624522 |
| C | 0.013730 | -2.631384 | -2.795499 |

| | | | |
|---|---|---|---|
| C | 1.434470 | -2.939086 | -2.393227 |
| O | 1.866378 | -1.985134 | -1.442475 |
| O | 0.794697 | -2.653279 | 1.636834 |
| C | -0.013155 | -2.613491 | 2.807508 |
| C | -1.433672 | -2.924525 | 2.407087 |
| N | 2.775965 | -0.552276 | 0.848856 |
| C | 2.911130 | -1.460884 | 1.990999 |
| C | 2.191146 | -2.795143 | 1.842512 |
| C | 3.244129 | -2.052879 | -1.140921 |
| C | 3.646297 | -0.860559 | -0.291200 |
| C | -3.019980 | 0.825260 | -1.284664 |
| C | 3.018523 | 0.836086 | 1.273435 |
| C | 2.461620 | 1.850543 | 0.301541 |
| C | -2.463460 | 1.846763 | -0.319916 |
| C | 1.001591 | 2.255731 | -1.462748 |
| C | 1.350792 | 3.625823 | -1.533276 |
| C | 2.311237 | 4.077100 | -0.588930 |
| C | 2.879874 | 3.201399 | 0.305837 |
| C | -2.882043 | 3.197444 | -0.333854 |
| C | -2.313696 | 4.079611 | 0.554722 |
| C | -1.353239 | 3.635317 | 1.502374 |
| C | -1.003660 | 2.264847 | 1.441617 |
| La | -0.000342 | -0.636905 | -0.000530 |
| H | -4.100388 | 1.011906 | -1.442464 |
| H | -2.514176 | 0.970974 | -2.248368 |
| H | 4.098832 | 1.024310 | 1.430034 |
| H | 2.512607 | 0.988312 | 2.236070 |
| C | 0.742419 | 4.440326 | -2.521692 |
| H | 2.608205 | 5.128680 | -0.592564 |
| H | 3.634640 | 3.536633 | 1.018991 |
| H | -3.636803 | 3.527432 | -1.049455 |
| H | -2.610906 | 5.131122 | 0.550833 |
| C | -0.745165 | 4.456989 | 2.485025 |
| N | -1.542350 | 1.429468 | 0.518396 |
| N | 1.540554 | 1.427033 | -0.533695 |
| C | 0.049834 | 1.656507 | -2.368278 |
| O | -0.259533 | 0.398948 | -2.207100 |
| C | -0.497602 | 2.496994 | -3.335434 |
| C | -0.051752 | 1.672315 | 2.351387 |
| O | 0.258123 | 0.413760 | 2.199102 |
| C | 0.495337 | 2.519816 | 3.312614 |
| H | -4.695064 | -1.014226 | -0.033938 |
| H | -3.832298 | -2.003790 | 2.082977 |
| H | -3.474753 | -3.016865 | 0.675478 |
| H | 4.694632 | -1.011443 | 0.035927 |
| H | 3.635724 | 0.019331 | -0.947461 |
| H | 3.832595 | -2.015402 | -2.074613 |
| H | 3.475466 | -3.019300 | -0.660468 |
| H | -1.504267 | -3.941661 | 1.980191 |
| H | -2.074115 | -2.875516 | 3.304811 |
| H | 0.045666 | -1.611603 | 3.266020 |
| H | 0.327059 | -3.377661 | 3.526042 |
| H | -3.636277 | 0.023563 | 0.942164 |
| H | 2.074863 | -2.895178 | -3.291251 |
| H | 1.505709 | -3.953537 | -1.960098 |
| H | -0.326037 | -3.400159 | -3.509315 |
| H | -0.045759 | -1.632325 | -3.260104 |

| | | | |
|---|---|---|---|
| H | -3.976604 | -1.684343 | -2.217378 |
| H | -2.480966 | -0.968498 | -2.859933 |
| H | -2.372883 | -3.414787 | -2.732364 |
| H | -2.562672 | -3.389911 | -0.977722 |
| H | 2.564258 | -3.380948 | 0.995051 |
| H | 2.374145 | -3.394610 | 2.749831 |
| H | 2.478612 | -0.946884 | 2.860719 |
| H | 3.975851 | -1.665609 | 2.224722 |
| C | -0.153196 | 3.865580 | -3.397227 |
| C | 0.150539 | 3.888709 | 3.364683 |
| H | 0.612932 | 4.512931 | 4.133147 |
| H | -0.999987 | 5.516529 | 2.541721 |
| H | 1.199635 | 2.105299 | 4.037718 |
| H | -0.615814 | 4.484204 | -4.170071 |
| H | 0.996968 | 5.499500 | -2.585948 |
| H | -1.201819 | 2.077138 | -4.057538 |

KHQLaΔ_λδδ_λδδ_opt.log

SCF (RwB97XD) = -1942.42366214
E(SCF)+ZPE(0 K)= -1941.741211
H(298 K)= -1941.704401
G(298 K)= -1941.804195
Lowest Frequency = 36.7292cm-1

81

| | | | |
|---|---|---|---|
| C | 3.726944 | 0.921830 | 0.471050 |
| N | 2.880562 | 0.727684 | -0.725070 |
| O | 1.873937 | 1.939067 | 1.602085 |
| C | 2.971416 | 1.057592 | 1.785454 |
| C | 3.102543 | 1.788871 | -1.717445 |
| C | 1.907726 | 2.089563 | -2.595752 |
| O | 0.870760 | 2.623056 | -1.769986 |
| C | -0.165406 | 3.310704 | -2.453313 |
| C | -1.308118 | 2.391157 | -2.816027 |
| O | -1.870205 | 1.918769 | -1.604443 |
| O | -0.873131 | 2.620753 | 1.765724 |
| C | 0.157326 | 3.319111 | 2.446822 |
| C | 1.307773 | 2.410323 | 2.812232 |
| N | -2.867051 | 0.705907 | 0.726555 |
| C | -3.097821 | 1.767833 | 1.716116 |
| C | -1.905335 | 2.080834 | 2.593290 |
| C | -2.960216 | 1.027444 | -1.784955 |
| C | -3.714789 | 0.889827 | -0.470204 |
| C | 3.057068 | -0.607192 | -1.311575 |
| C | -3.032577 | -0.628757 | 1.316710 |
| C | -2.479011 | -1.710556 | 0.419025 |
| C | 2.512651 | -1.690996 | -0.410678 |
| C | -1.018175 | -2.207664 | -1.320862 |
| C | -1.368705 | -3.578095 | -1.326197 |
| C | -2.331672 | -3.979687 | -0.361644 |
| C | -2.903184 | -3.058137 | 0.485210 |
| C | 2.948045 | -3.035180 | -0.472965 |
| C | 2.384257 | -3.958993 | 0.376609 |
| C | 1.418097 | -3.562613 | 1.340126 |
| C | 1.056085 | -2.195195 | 1.330751 |
| La | 0.006726 | 0.755290 | 0.000612 |

| | | | |
|---|---|---|---|
| H | 4.335695 | 1.826644 | 0.330263 |
| H | 4.439836 | 0.088332 | 0.582321 |
| H | 3.658642 | 1.474367 | 2.542048 |
| H | 2.597393 | 0.090459 | 2.154313 |
| H | 0.232048 | 3.798733 | -3.357639 |
| H | -0.527361 | 4.094541 | -1.770972 |
| H | -0.954001 | 1.535235 | -3.417357 |
| H | -2.065781 | 2.951440 | -3.391779 |
| H | -0.244105 | 3.806535 | 3.349717 |
| H | 0.512625 | 4.103890 | 1.762068 |
| H | 2.060644 | 2.978693 | 3.386339 |
| H | 0.960879 | 1.553224 | 3.416101 |
| H | -2.578034 | 0.062166 | -2.150305 |
| H | -3.650792 | 1.435837 | -2.543062 |
| H | -4.330540 | 1.790326 | -0.332231 |
| H | -4.421165 | 0.050464 | -0.578995 |
| H | 4.122886 | -0.806365 | -1.539092 |
| H | 2.498783 | -0.646587 | -2.258791 |
| H | -4.096764 | -0.836132 | 1.544558 |
| H | -2.474250 | -0.660929 | 2.264161 |
| C | -0.761093 | -4.434620 | -2.278981 |
| H | -2.632046 | -5.029143 | -0.313597 |
| H | -3.664754 | -3.355615 | 1.207744 |
| H | 3.712008 | -3.328413 | -1.194709 |
| H | 2.693335 | -5.006053 | 0.331572 |
| C | 0.817817 | -4.421335 | 2.295583 |
| N | 1.579509 | -1.321670 | 0.436335 |
| N | -1.548974 | -1.335907 | -0.429044 |
| C | -0.084582 | -1.644417 | -2.265583 |
| O | 0.198665 | -0.371610 | -2.172020 |
| C | 0.462333 | -2.524206 | -3.195338 |
| C | 0.117823 | -1.636975 | 2.273820 |
| O | -0.176087 | -0.366870 | 2.176419 |
| C | -0.421581 | -2.518516 | 3.206296 |
| H | -2.207336 | 2.840762 | 3.333263 |
| H | -1.525413 | 1.188823 | 3.116558 |
| H | -3.961941 | 1.530908 | 2.367109 |
| H | -3.344064 | 2.690687 | 1.173982 |
| H | 2.203635 | 2.850006 | -3.337655 |
| H | 1.535500 | 1.193040 | -3.116801 |
| H | 3.340872 | 2.715198 | -1.177702 |
| H | 3.968785 | 1.557499 | -2.367621 |
| C | -0.074264 | -3.888360 | 3.201261 |
| C | 0.126589 | -3.896904 | -3.186175 |
| H | 0.587707 | -4.549665 | -3.931281 |
| H | -1.011620 | -5.496591 | -2.294310 |
| H | 1.158917 | -2.135693 | -3.941696 |
| H | -0.529789 | -4.542722 | 3.948399 |
| H | 1.077261 | -5.481113 | 2.314084 |
| H | -1.121359 | -2.133632 | 3.951545 |

KHQLaΔ_λδλ_λδλ_opt.log

SCF (RwB97XD) = -1942.42721527
E(SCF)+ZPE(0 K)= -1941.745936
H(298 K)= -1941.708571
G(298 K)= -1941.811282

Lowest Frequency = 27.6234cm-1

81

| | | | |
|---|---|---|---|
| C | 3.692726 | 1.073979 | -0.197752 |
| N | 2.680608 | 0.632886 | -1.174781 |
| O | 2.024461 | 2.030511 | 1.217284 |
| C | 3.190859 | 1.220201 | 1.229138 |
| C | 2.599049 | 1.528667 | -2.334743 |
| C | 1.967260 | 2.870877 | -2.012172 |
| O | 0.611699 | 2.680938 | -1.629148 |
| C | -0.403160 | 3.379415 | -2.328750 |
| C | -1.596008 | 2.469961 | -2.508423 |
| O | -2.028085 | 2.047546 | -1.225870 |
| O | -0.609855 | 2.686486 | 1.620092 |
| C | 0.410780 | 3.375644 | 2.320581 |
| C | 1.595761 | 2.455903 | 2.499987 |
| N | -2.696086 | 0.656075 | 1.166241 |
| C | -2.607160 | 1.551677 | 2.325820 |
| C | -1.963839 | 2.888242 | 2.002683 |
| C | -3.201345 | 1.247219 | -1.237852 |
| C | -3.704496 | 1.105229 | 0.189019 |
| C | 2.880784 | -0.754565 | -1.609375 |
| C | -2.908070 | -0.729458 | 1.601466 |
| C | -2.505730 | -1.747274 | 0.561191 |
| C | 2.470055 | -1.768443 | -0.568541 |
| C | -1.234435 | -2.191809 | -1.335508 |
| C | -1.630887 | -3.550360 | -1.373519 |
| C | -2.518899 | -3.972587 | -0.347965 |
| C | -2.972110 | -3.082575 | 0.597048 |
| C | 2.925557 | -3.107502 | -0.603591 |
| C | 2.465056 | -3.993251 | 0.341897 |
| C | 1.580412 | -3.563220 | 1.367124 |
| C | 1.195031 | -2.201499 | 1.328287 |
| La | -0.007654 | 0.684397 | -0.004311 |
| H | 4.098091 | 2.046758 | -0.515066 |
| H | 4.548583 | 0.377576 | -0.185246 |
| H | 3.975527 | 1.707991 | 1.833494 |
| H | 2.955004 | 0.245866 | 1.685985 |
| H | 1.963778 | 1.035667 | -3.083962 |
| H | 3.595614 | 1.701898 | -2.788074 |
| H | 2.018603 | 3.513629 | -2.904718 |
| H | 2.489640 | 3.390299 | -1.192965 |
| H | -0.043467 | 3.685510 | -3.324145 |
| H | -0.684117 | 4.284725 | -1.764095 |
| H | -1.315130 | 1.586392 | -3.108525 |
| H | -2.403650 | 3.023661 | -3.018427 |
| H | 0.053397 | 3.684041 | 3.316097 |
| H | 0.699667 | 4.278945 | 1.756711 |
| H | 2.407978 | 3.002453 | 3.010438 |
| H | 1.307291 | 1.574400 | 3.099543 |
| H | -1.976302 | 1.053726 | 3.075477 |
| H | -3.602323 | 1.733607 | 2.778820 |
| H | -2.481558 | 3.411602 | 1.183040 |
| H | -2.009917 | 3.531966 | 2.894813 |
| H | -2.973788 | 0.270990 | -1.694840 |
| H | -3.981806 | 1.741732 | -1.842193 |
| H | -4.102013 | 2.081294 | 0.506183 |

| | | | |
|---|---|---|---|
| H | -4.565959 | 0.415782 | 0.176511 |
| H | 3.929586 | -0.934938 | -1.915698 |
| H | 2.246674 | -0.923533 | -2.491428 |
| H | -3.958423 | -0.900802 | 1.907657 |
| H | -2.275603 | -0.903377 | 2.483720 |
| C | -1.139409 | -4.380226 | -2.412345 |
| H | -2.852907 | -5.012847 | -0.327883 |
| H | -3.671144 | -3.395197 | 1.374454 |
| H | 3.622111 | -3.426236 | -1.380741 |
| H | 2.790627 | -5.036194 | 0.322469 |
| C | 1.082147 | -4.388457 | 2.406400 |
| N | 1.620470 | -1.364487 | 0.347901 |
| N | -1.652942 | -1.350809 | -0.355553 |
| C | -0.368537 | -1.613693 | -2.335799 |
| O | -0.038522 | -0.355297 | -2.223999 |
| C | 0.070415 | -2.470272 | -3.343279 |
| C | 0.333860 | -1.615788 | 2.328218 |
| O | 0.014132 | -0.354808 | 2.215720 |
| C | -0.112100 | -2.468180 | 3.336178 |
| C | 0.257409 | -3.831264 | 3.359956 |
| C | -0.310205 | -3.830312 | -3.366250 |
| H | 0.064152 | -4.461794 | -4.175581 |
| H | -1.428724 | -5.431647 | -2.451858 |
| H | 0.710798 | -2.067728 | -4.131518 |
| H | -0.122106 | -4.459208 | 4.169636 |
| H | 1.362905 | -5.442171 | 2.446534 |
| H | -0.749208 | -2.059964 | 4.124154 |

KHQLaΔ_λλδ_λλδ_opt.log

SCF (RwB97XD) = -1942.42259001
E(SCF)+ZPE(0 K)= -1941.739863
H(298 K)= -1941.703148
G(298 K)= -1941.802317
Lowest Frequency = 43.0274cm-1

81

| | | | |
|---|---|---|---|
| C | -3.599088 | -0.840345 | 0.744138 |
| N | -2.874740 | -0.654167 | -0.528845 |
| O | -1.803742 | -2.312371 | 1.331865 |
| C | -2.710610 | -1.355051 | 1.872362 |
| C | -3.185538 | -1.746188 | -1.458932 |
| C | -2.163134 | -1.957051 | -2.551647 |
| O | -0.943393 | -2.405155 | -1.975192 |
| C | 0.040352 | -2.700822 | -2.948951 |
| C | 1.257365 | -3.241904 | -2.235505 |
| O | 1.803295 | -2.300429 | -1.330053 |
| O | 0.943493 | -2.405509 | 1.976728 |
| C | -0.038860 | -2.707605 | 2.949924 |
| C | -1.253305 | -3.253044 | 2.235423 |
| N | 2.866369 | -0.641921 | 0.534442 |
| C | 3.182423 | -1.734867 | 1.461666 |
| C | 2.161258 | -1.953221 | 2.554050 |
| C | 2.705821 | -1.337834 | -1.868430 |
| C | 3.591697 | -0.821347 | -0.738948 |
| C | -3.123970 | 0.670129 | -1.107959 |
| C | 3.109240 | 0.682032 | 1.117059 |

| | | | |
|---|---|---|---|
| C | 2.482401 | 1.764746 | 0.268735 |
| C | -2.502041 | 1.753623 | -0.257016 |
| C | 0.931713 | 2.236712 | -1.396566 |
| C | 1.250686 | 3.614165 | -1.412759 |
| C | 2.246076 | 4.033679 | -0.489614 |
| C | 2.875722 | 3.121638 | 0.326430 |
| C | -2.901690 | 3.108808 | -0.311334 |
| C | -2.276295 | 4.021752 | 0.506963 |
| C | -1.278945 | 3.604597 | 1.429062 |
| C | -0.953575 | 2.228686 | 1.409463 |
| La | -0.003942 | -0.725713 | 0.002718 |
| H | -4.446709 | -1.528404 | 0.590540 |
| H | -4.031167 | 0.113923 | 1.076867 |
| H | -3.331840 | -1.826350 | 2.651692 |
| H | -2.141330 | -0.529597 | 2.333253 |
| H | 2.132843 | -0.514086 | -2.327778 |
| H | 3.329363 | -1.804672 | -2.648599 |
| H | 4.442775 | -1.505486 | -0.586963 |
| H | 4.018974 | 0.135883 | -1.069342 |
| H | -4.207687 | 0.862302 | -1.235086 |
| H | -2.658874 | 0.711005 | -2.103734 |
| H | 4.192000 | 0.878969 | 1.245009 |
| H | 2.643585 | 0.718125 | 2.112784 |
| C | 0.578314 | 4.457705 | -2.333540 |
| H | 2.523720 | 5.089651 | -0.448023 |
| H | 3.660475 | 3.433700 | 1.017307 |
| H | -3.687917 | 3.418913 | -1.001417 |
| H | -2.558863 | 5.076517 | 0.467986 |
| C | -0.610469 | 4.448977 | 2.351908 |
| N | -1.541972 | 1.363336 | 0.549939 |
| N | 1.524179 | 1.371985 | -0.539212 |
| C | -0.041075 | 1.655094 | -2.287287 |
| O | -0.297429 | 0.378878 | -2.167328 |
| C | -0.650700 | 2.521563 | -3.189827 |
| C | 0.021915 | 1.649399 | 2.298741 |
| O | 0.284213 | 0.374689 | 2.175623 |
| C | 0.627525 | 2.516458 | 3.203417 |
| H | 2.545361 | -2.727586 | 3.240969 |
| H | 1.958976 | -1.035716 | 3.129575 |
| H | 4.173066 | -1.581891 | 1.934131 |
| H | 3.239602 | -2.662078 | 0.875091 |
| H | -2.543507 | -2.731473 | -3.240571 |
| H | -1.964900 | -1.037197 | -3.124832 |
| H | -3.238543 | -2.675154 | -0.874746 |
| H | -4.176779 | -1.596611 | -1.931228 |
| H | 0.279295 | -1.783650 | -3.516904 |
| H | -0.338401 | -3.463532 | -3.652516 |
| H | 2.018084 | -3.556112 | -2.969068 |
| H | 0.976396 | -4.119072 | -1.634156 |
| H | -0.282063 | -1.792578 | 3.519535 |
| H | 0.343456 | -3.469850 | 3.652063 |
| H | -0.968191 | -4.127673 | 1.632330 |
| H | -2.012505 | -3.572316 | 2.968373 |
| C | 0.311311 | 3.894274 | 3.213955 |
| C | -0.340878 | 3.900852 | -3.196964 |
| H | -0.851170 | 4.543916 | -3.918092 |
| H | 0.804076 | 5.525015 | -2.359750 |

| | | | |
|---|---|---|---|
| H | -1.377771 | 2.119736 | -3.899210 |
| H | 0.818625 | 4.537923 | 3.936661 |
| H | -0.841183 | 5.515161 | 2.380747 |
| H | 1.356458 | 2.116256 | 3.911808 |

MacropaLaΔ_δδδ_δδδ_opt.log

SCF (RwB97XD) = -1861.92003121
E(SCF)+ZPE(0 K)= -1861.310793
H(298 K)= -1861.275493
G(298 K)= -1861.373699
Lowest Frequency = 33.0679cm-1

73

| | | | |
|---|---|---|---|
| C | 3.618769 | -0.846323 | -0.543780 |
| N | 2.860157 | -0.613383 | 0.690140 |
| O | 1.737551 | -1.910699 | -1.576467 |
| C | 3.129970 | -2.038432 | -1.343370 |
| C | 3.175705 | -1.618601 | 1.713826 |
| C | 1.994774 | -1.991933 | 2.582269 |
| O | 0.993735 | -2.564668 | 1.735184 |
| C | -0.001735 | -3.333153 | 2.388807 |
| C | -1.201250 | -2.489642 | 2.764362 |
| O | -1.732042 | -1.915214 | 1.576842 |
| O | -0.986297 | -2.568057 | -1.734618 |
| C | 0.011360 | -3.333886 | -2.388028 |
| C | 1.208451 | -2.487045 | -2.763815 |
| N | -2.858346 | -0.621849 | -0.690113 |
| C | -3.171039 | -1.628322 | -1.713435 |
| C | -1.989100 | -1.998598 | -2.581815 |
| C | -3.124109 | -2.046950 | 1.343891 |
| C | -3.616301 | -0.856517 | 0.543888 |
| C | 3.092463 | 0.747542 | 1.196203 |
| C | -3.094523 | 0.738258 | -1.196613 |
| C | -2.543278 | 1.800297 | -0.272811 |
| C | 2.538027 | 1.807750 | 0.272206 |
| C | -1.013800 | 2.284761 | 1.409209 |
| C | -1.443544 | 3.602113 | 1.499112 |
| C | -2.467535 | 4.014340 | 0.646791 |
| C | -3.040627 | 3.102961 | -0.237101 |
| C | 3.031520 | 3.111874 | 0.236233 |
| C | 2.455705 | 4.021390 | -0.647806 |
| C | 1.432911 | 3.605976 | -1.500018 |
| C | 1.007069 | 2.287376 | -1.409861 |
| La | 0.000993 | -0.673180 | -0.000013 |
| H | 0.416520 | -3.823129 | 3.283405 |
| H | -0.310261 | -4.117314 | 1.680648 |
| H | -0.917474 | -1.671626 | 3.446052 |
| H | -1.953594 | -3.124205 | 3.258345 |
| H | -0.405492 | -3.825298 | -3.282492 |
| H | 0.322125 | -4.116966 | -1.679652 |
| H | 1.962639 | -3.119588 | -3.257577 |
| H | 0.922334 | -1.670077 | -3.445779 |
| H | 4.172917 | 0.930101 | 1.357351 |
| H | 2.588135 | 0.852825 | 2.169527 |
| H | -4.175476 | 0.917647 | -1.357963 |
| H | -2.590331 | 0.844753 | -2.169882 |

| | | | |
|---|---|---|---|
| H | -0.974690 | 4.263715 | 2.227866 |
| H | -2.832708 | 5.042765 | 0.681433 |
| H | -3.858989 | 3.396885 | -0.895903 |
| H | 3.849033 | 3.408336 | 0.894954 |
| H | 2.817830 | 5.050885 | -0.682642 |
| H | 0.962081 | 4.266058 | -2.228876 |
| N | 1.532943 | 1.437850 | -0.521141 |
| N | -1.537135 | 1.433517 | 0.520643 |
| C | 0.085867 | 1.710853 | 2.302196 |
| O | 0.270843 | 0.444747 | 2.139959 |
| O | 0.708308 | 2.449606 | 3.038842 |
| C | -0.090919 | 1.710065 | -2.302715 |
| O | -0.272140 | 0.443447 | -2.140272 |
| O | -0.715584 | 2.446864 | -3.039441 |
| H | 4.699212 | -0.979565 | -0.336689 |
| H | 3.674998 | -2.053239 | -2.300808 |
| H | 3.325335 | -2.997477 | -0.832603 |
| H | -4.696360 | -0.992902 | 0.336838 |
| H | -3.518315 | 0.033508 | 1.178774 |
| H | -3.669034 | -2.062927 | 2.301369 |
| H | -3.316813 | -3.006735 | 0.833504 |
| H | -1.578248 | -1.119020 | -3.101835 |
| H | -2.309993 | -2.752143 | -3.320027 |
| H | -3.514955 | -2.545661 | -1.215855 |
| H | -4.001018 | -1.291821 | -2.361982 |
| H | 2.317772 | -2.744233 | 3.320830 |
| H | 1.581283 | -1.113314 | 3.101850 |
| H | 4.004695 | -1.279500 | 2.362280 |
| H | 3.522257 | -2.535130 | 1.216582 |
| H | 3.518253 | 0.043198 | -1.178971 |

MacropaLaΔ_δδλ_δδλ_opt.log

SCF (RwB97XD) = -1861.91456304
E(SCF)+ZPE(0 K)= -1861.306347
H(298 K)= -1861.270664
G(298 K)= -1861.370448
Lowest Frequency = 25.5337cm-1

73

| | | | |
|---|---|---|---|
| C | 3.605428 | 0.943567 | -0.263370 |
| N | 2.669277 | 0.853712 | 0.861132 |
| O | 2.688360 | -1.093138 | -1.149536 |
| C | 3.887661 | -0.359452 | -0.990451 |
| C | 3.157732 | 0.101769 | 2.022735 |
| C | 3.084055 | -1.417957 | 1.924493 |
| O | 1.755715 | -1.830643 | 1.648568 |
| C | 1.176420 | -2.863442 | 2.426808 |
| C | -0.277535 | -2.504645 | 2.665325 |
| O | -0.940369 | -2.246139 | 1.440273 |
| O | 0.514131 | -2.697017 | -1.442659 |
| C | 1.807855 | -3.269434 | -1.505768 |
| C | 2.790699 | -2.227258 | -1.991079 |
| N | -2.111263 | -1.723434 | -1.108188 |
| C | -1.670488 | -2.347354 | -2.359090 |
| C | -0.511922 | -3.312953 | -2.209138 |
| C | -1.672067 | -3.286932 | 0.826936 |

| | | | |
|---|---|---|---|
| C | -2.678340 | -2.655584 | -0.125791 |
| C | 2.286450 | 2.216098 | 1.279540 |
| C | -3.070814 | -0.652123 | -1.425919 |
| C | -3.224409 | 0.353871 | -0.310588 |
| C | 1.440497 | 2.925566 | 0.248537 |
| C | -2.189678 | 1.476681 | 1.440019 |
| C | -3.325176 | 2.234040 | 1.700186 |
| C | -4.455556 | 2.009748 | 0.917159 |
| C | -4.414674 | 1.047727 | -0.089543 |
| C | 1.376950 | 4.314943 | 0.146088 |
| C | 0.532132 | 4.877521 | -0.808820 |
| C | -0.202030 | 4.050411 | -1.657822 |
| C | -0.061821 | 2.676587 | -1.508754 |
| La | 0.257128 | -0.414211 | -0.067020 |
| H | 2.521672 | 0.392482 | 2.869959 |
| H | 4.200954 | 0.380501 | 2.274851 |
| H | 3.407988 | -1.824182 | 2.896085 |
| H | 3.754672 | -1.836652 | 1.160126 |
| H | 1.688651 | -2.951575 | 3.398690 |
| H | 1.277490 | -3.829144 | 1.898864 |
| H | -0.329603 | -1.565547 | 3.234312 |
| H | -0.788374 | -3.304639 | 3.225273 |
| H | 1.821267 | -4.139410 | -2.180554 |
| H | 2.088630 | -3.610572 | -0.495581 |
| H | 3.809865 | -2.648218 | -1.948408 |
| H | 2.574603 | -1.934588 | -3.032785 |
| H | -1.348465 | -1.526040 | -3.014350 |
| H | -2.499464 | -2.887710 | -2.859073 |
| H | -0.808914 | -4.263100 | -1.731537 |
| H | -0.136170 | -3.552207 | -3.217621 |
| H | 3.179637 | 2.826538 | 1.513091 |
| H | 1.681590 | 2.137190 | 2.195651 |
| H | -4.060183 | -1.068767 | -1.693771 |
| H | -2.698666 | -0.105992 | -2.307212 |
| H | -3.298356 | 2.965526 | 2.508208 |
| H | -5.372717 | 2.574479 | 1.096482 |
| H | -5.291703 | 0.841551 | -0.705115 |
| H | 1.973111 | 4.943478 | 0.809317 |
| H | 0.456277 | 5.963229 | -0.896741 |
| H | -0.869264 | 4.435029 | -2.429540 |
| N | 0.720238 | 2.149161 | -0.560911 |
| N | -2.147421 | 0.586309 | 0.441996 |
| C | -0.918463 | 1.600353 | 2.280772 |
| O | -0.079119 | 0.641797 | 2.089338 |
| O | -0.777481 | 2.556464 | 3.017338 |
| C | -0.787668 | 1.664251 | -2.395642 |
| O | -0.368267 | 0.455452 | -2.239481 |
| O | -1.692365 | 2.037717 | -3.115830 |
| H | 3.169084 | 1.644180 | -0.987994 |
| H | 4.577034 | 1.372010 | 0.055088 |
| H | 4.321544 | -0.118040 | -1.975780 |
| H | 4.626249 | -0.981035 | -0.453523 |
| H | -3.257808 | -3.459472 | -0.620566 |
| H | -3.385437 | -2.090929 | 0.498513 |
| H | -2.230138 | -3.855652 | 1.590159 |
| H | -0.987353 | -3.989493 | 0.323785 |

MacropaLaΔ_δλδ_δλδ_opt.log

SCF (RwB97XD) = -1861.92551980
E(SCF)+ZPE(0 K)= -1861.316008
H(298 K)= -1861.280755
G(298 K)= -1861.378754
Lowest Frequency = 35.2014cm-1

73

| | | | |
|---|---|---|---|
| C | 3.577759 | -0.893184 | -0.734368 |
| N | 2.904186 | -0.614130 | 0.536160 |
| O | 1.690853 | -2.127951 | -1.535330 |
| C | 3.100289 | -2.166628 | -1.399206 |
| C | 3.291331 | -1.587274 | 1.562652 |
| C | 2.242776 | -1.794306 | 2.625541 |
| O | 1.067818 | -2.328971 | 2.022140 |
| C | 0.106779 | -2.669098 | 3.000925 |
| C | -1.142616 | -3.170435 | 2.331481 |
| O | -1.687195 | -2.129426 | 1.537159 |
| O | -1.063785 | -2.332663 | -2.020120 |
| C | -0.102176 | -2.672118 | -2.998577 |
| C | 1.148069 | -3.170667 | -2.328640 |
| N | -2.903115 | -0.619603 | -0.535727 |
| C | -3.288571 | -1.594316 | -1.561364 |
| C | -2.239631 | -1.800530 | -2.624034 |
| C | -3.096553 | -2.170699 | 1.401031 |
| C | -3.576233 | -0.898694 | 0.735033 |
| C | 3.188230 | 0.754336 | 0.988546 |
| C | -3.189492 | 0.747976 | -0.989312 |
| C | -2.586463 | 1.800029 | -0.090806 |
| C | 2.583390 | 1.804580 | 0.089141 |
| C | -0.936691 | 2.298122 | 1.463924 |
| C | -1.374346 | 3.608867 | 1.595286 |
| C | -2.471480 | 4.010671 | 0.834322 |
| C | -3.099287 | 3.094627 | -0.005461 |
| C | 3.094006 | 3.099980 | 0.002675 |
| C | 2.464631 | 4.014228 | -0.837891 |
| C | 1.368175 | 3.609896 | -1.598493 |
| C | 0.932758 | 2.298522 | -1.465996 |
| La | 0.000624 | -0.677211 | 0.000246 |
| H | 4.279946 | 0.923273 | 1.069310 |
| H | 2.755422 | 0.893275 | 1.991404 |
| H | -4.281494 | 0.914978 | -1.070248 |
| H | -2.756889 | 0.886776 | -1.992277 |
| H | -0.854070 | 4.273295 | 2.285551 |
| H | -2.847916 | 5.033114 | 0.905279 |
| H | -3.971937 | 3.378590 | -0.595366 |
| H | 3.966177 | 3.385936 | 0.592325 |
| H | 2.839325 | 5.037249 | -0.909735 |
| H | 0.846755 | 4.272840 | -2.289324 |
| N | 1.514744 | 1.440990 | -0.619486 |
| N | -1.517205 | 1.438873 | 0.618144 |
| C | 0.237797 | 1.737546 | 2.260304 |
| O | 0.410737 | 0.472509 | 2.087859 |
| O | 0.920958 | 2.480587 | 2.936957 |
| C | -0.240780 | 1.735248 | -2.261872 |
| O | -0.411516 | 0.470059 | -2.088366 |

| | | | |
|---|---|---|---|
| O | -0.925247 | 2.476542 | -2.939122 |
| H | 4.677012 | -0.958061 | -0.607275 |
| H | 3.567024 | -2.233497 | -2.396835 |
| H | 3.391029 | -3.073147 | -0.840161 |
| H | -4.675367 | -0.965607 | 0.607968 |
| H | -3.378601 | -0.062431 | 1.419069 |
| H | -3.563201 | -2.237485 | 2.398706 |
| H | -3.385688 | -3.078234 | 0.842802 |
| H | -1.978923 | -0.859530 | -3.133458 |
| H | -2.623025 | -2.521726 | -3.366644 |
| H | -3.469615 | -2.564969 | -1.081584 |
| H | -4.238320 | -1.302622 | -2.049051 |
| H | 2.627410 | -2.514182 | 3.368792 |
| H | 1.980485 | -0.853287 | 3.134117 |
| H | 4.240604 | -1.293536 | 2.050040 |
| H | 3.473997 | -2.558047 | 1.083732 |
| H | 0.943401 | -4.054839 | -1.698615 |
| H | 1.868081 | -3.465386 | -3.109453 |
| H | 0.131767 | -1.780544 | -3.606792 |
| H | -0.498104 | -3.462225 | -3.661302 |
| H | 3.378646 | -0.057883 | -1.419150 |
| H | -1.862114 | -3.465623 | 3.112590 |
| H | -0.936439 | -4.054872 | 1.702320 |
| H | 0.504046 | -3.457878 | 3.664426 |
| H | -0.128680 | -1.777326 | 3.608260 |

MacropaLaΔ_δλλ_δλλ_opt.log

SCF (RwB97XD) = -1861.92280131
E(SCF)+ZPE(0 K)= -1861.313203
H(298 K)= -1861.278087
G(298 K)= -1861.375577
Lowest Frequency = 27.3478cm-1

73

| | | | |
|---|---|---|---|
| C | -3.652466 | -0.849688 | 0.245176 |
| N | -2.766776 | -0.501591 | -0.872135 |
| O | -1.872535 | -1.943781 | 1.429093 |
| C | -3.240487 | -2.043827 | 1.086911 |
| C | -2.882485 | -1.373299 | -2.046536 |
| C | -2.188269 | -2.721581 | -1.919574 |
| O | -0.794581 | -2.605866 | -1.668712 |
| C | 0.043256 | -2.586866 | -2.818829 |
| C | 1.450984 | -2.901969 | -2.381585 |
| O | 1.873326 | -1.943213 | -1.428872 |
| O | 0.795633 | -2.605338 | 1.669021 |
| C | -0.042208 | -2.586513 | 2.819138 |
| C | -1.449810 | -2.902239 | 2.381937 |
| N | 2.766996 | -0.500388 | 0.872177 |
| C | 2.883063 | -1.371915 | 2.046677 |
| C | 2.189367 | -2.720481 | 1.919888 |
| C | 3.241324 | -2.042656 | -1.086695 |
| C | 3.652822 | -0.848254 | -0.245098 |
| C | -3.028319 | 0.893486 | -1.267404 |
| C | 3.027969 | 0.894842 | 1.267284 |
| C | 2.536186 | 1.887354 | 0.240843 |
| C | -2.536926 | 1.886322 | -0.241089 |

| | | | |
|---|---|---|---|
| C | 1.052245 | 2.291803 | -1.499617 |
| C | 1.512609 | 3.591222 | -1.666196 |
| C | 2.535246 | 4.035325 | -0.828882 |
| C | 3.068335 | 3.172139 | 0.124964 |
| C | -3.069585 | 3.170910 | -0.125369 |
| C | -2.536835 | 4.034425 | 0.828369 |
| C | -1.514021 | 3.590830 | 1.665737 |
| C | -1.053143 | 2.291574 | 1.499319 |
| La | 0.000131 | -0.616170 | 0.000026 |
| H | -4.106832 | 1.058368 | -1.454023 |
| H | -2.498695 | 1.099899 | -2.209660 |
| H | 4.106412 | 1.060185 | 1.453896 |
| H | 2.498246 | 1.101149 | 2.209508 |
| H | 1.067994 | 4.214202 | -2.442632 |
| H | 2.928142 | 5.049267 | -0.927829 |
| H | 3.882764 | 3.489100 | 0.778149 |
| H | -3.884140 | 3.487468 | -0.778591 |
| H | -2.930131 | 5.048224 | 0.927192 |
| H | -1.069653 | 4.214082 | 2.442096 |
| N | -1.540890 | 1.485315 | 0.549324 |
| N | 1.540310 | 1.485855 | -0.549523 |
| C | -0.046176 | 1.693745 | -2.376542 |
| O | -0.242941 | 0.436814 | -2.174047 |
| O | -0.663213 | 2.413768 | -3.137478 |
| C | 0.045511 | 1.694055 | 2.376318 |
| O | 0.242776 | 0.437180 | 2.173969 |
| O | 0.662263 | 2.414413 | 3.137169 |
| H | -4.690366 | -1.018241 | -0.104830 |
| H | -3.854142 | -2.036953 | 2.004797 |
| H | -3.429843 | -3.008923 | 0.587537 |
| H | 4.690793 | -1.016345 | 0.104922 |
| H | 3.679715 | 0.020799 | -0.916256 |
| H | 3.854965 | -2.035633 | -2.004589 |
| H | 3.431084 | -3.007618 | -0.587215 |
| H | -1.501323 | -3.912760 | 1.937598 |
| H | -2.111023 | -2.872953 | 3.265080 |
| H | 0.002824 | -1.593413 | 3.297103 |
| H | 0.284196 | -3.358847 | 3.535047 |
| H | -3.679714 | 0.019432 | 0.916234 |
| H | 2.112184 | -2.872538 | -3.264733 |
| H | 1.502902 | -3.912409 | -1.937108 |
| H | -0.282838 | -3.359428 | -3.534632 |
| H | -0.002172 | -1.593851 | -3.296932 |
| H | -3.943652 | -1.553741 | -2.312665 |
| H | -2.419835 | -0.835951 | -2.886044 |
| H | -2.351243 | -3.287218 | -2.851487 |
| H | -2.596099 | -3.331083 | -1.105369 |
| H | 2.597425 | -3.329924 | 1.105755 |
| H | 2.352567 | -3.285942 | 2.851868 |
| H | 2.420221 | -0.834650 | 2.886132 |
| H | 3.944304 | -1.551913 | 2.312811 |

MacropaLaΔ_δλλ_λδλ.log

SCF (RwB97XD) = -1861.92467977
E(SCF)+ZPE(0 K)= -1861.315000
H(298 K)= -1861.279854

G(298 K)= -1861.377327
Lowest Frequency = 32.4894cm-1

73

| | | | |
|---|---|---|---|
| La | 0.878267 | 3.986601 | 9.179094 |
| N | 3.144985 | 2.608587 | 9.218658 |
| C | 3.171543 | 1.278445 | 9.302768 |
| C | 4.313858 | 0.615239 | 9.750403 |
| H | 4.324290 | -0.473204 | 9.823255 |
| C | 5.429325 | 1.369733 | 10.106781 |
| H | 6.329740 | 0.871585 | 10.471879 |
| C | 5.397100 | 2.757798 | 9.976169 |
| C | 4.225213 | 3.340284 | 9.511735 |
| C | 4.073894 | 4.848366 | 9.313961 |
| O | 2.949866 | 5.189571 | 8.784216 |
| H | 6.246457 | 3.397042 | 10.218132 |
| N | 1.899722 | 4.421922 | 11.620405 |
| C | 2.453822 | 5.577230 | 11.987797 |
| C | 3.302583 | 5.647245 | 13.093277 |
| H | 3.751156 | 6.599659 | 13.379612 |
| C | 3.570473 | 4.483442 | 13.808468 |
| H | 4.242899 | 4.512961 | 14.668195 |
| C | 2.961363 | 3.287614 | 13.429996 |
| C | 2.114901 | 3.307604 | 12.330009 |
| C | 1.376588 | 2.065313 | 11.841028 |
| O | 0.537286 | 2.311059 | 10.894868 |
| H | 3.116481 | 2.347616 | 13.960046 |
| C | 1.908911 | 0.553293 | 8.896248 |
| H | 2.172446 | -0.431329 | 8.463191 |
| H | 1.321521 | 0.368547 | 9.808635 |
| N | 1.096329 | 1.337991 | 7.954572 |
| C | 1.792036 | 1.468143 | 6.669669 |
| H | 1.788148 | 0.511259 | 6.110998 |
| H | 2.840994 | 1.721766 | 6.871055 |
| C | 1.219723 | 2.548245 | 5.777845 |
| H | 1.864841 | 2.640917 | 4.887239 |
| H | 0.201448 | 2.312024 | 5.419645 |
| O | 1.196851 | 3.774537 | 6.482247 |
| C | 0.969764 | 4.899380 | 5.655491 |
| H | -0.039794 | 4.836164 | 5.210357 |
| H | 1.711832 | 4.925560 | 4.838865 |
| C | 1.108151 | 6.149911 | 6.482029 |
| H | 2.130617 | 6.235776 | 6.886384 |
| H | 0.882158 | 7.022709 | 5.847547 |
| O | 0.172260 | 6.071660 | 7.552019 |
| C | -0.134851 | 7.303404 | 8.185146 |
| H | -1.175941 | 7.224000 | 8.515178 |
| H | -0.097954 | 8.120066 | 7.445305 |
| C | 0.820096 | 7.637547 | 9.322249 |
| H | 1.832597 | 7.705550 | 8.900076 |
| H | 0.560904 | 8.642935 | 9.711673 |
| N | 0.874098 | 6.643632 | 10.398406 |
| C | 2.142383 | 6.781223 | 11.133212 |
| H | 2.140493 | 7.691212 | 11.763696 |
| H | 2.961248 | 6.885538 | 10.405735 |
| C | -0.238260 | 6.713944 | 11.351119 |
| H | -0.371677 | 7.744295 | 11.737577 |

| | | | |
|---|---|---|---|
| H | 0.031665 | 6.082827 | 12.208501 |
| C | -1.581723 | 6.227810 | 10.839866 |
| H | -2.248559 | 6.092138 | 11.708917 |
| H | -2.071821 | 6.956858 | 10.173203 |
| O | -1.418475 | 4.992138 | 10.169396 |
| C | -2.627486 | 4.371765 | 9.759751 |
| H | -2.911745 | 4.736305 | 8.756431 |
| H | -3.438241 | 4.624823 | 10.462332 |
| C | -2.444352 | 2.877360 | 9.768236 |
| H | -2.139721 | 2.544670 | 10.776173 |
| H | -3.403420 | 2.396243 | 9.506655 |
| O | -1.447328 | 2.507280 | 8.834508 |
| C | -1.223327 | 1.098730 | 8.849001 |
| H | -2.175186 | 0.580155 | 8.640779 |
| H | -0.864600 | 0.810065 | 9.849364 |
| C | -0.230723 | 0.740005 | 7.770166 |
| H | -0.163132 | -0.363011 | 7.705361 |
| H | -0.641580 | 1.085282 | 6.811708 |
| O | 1.638449 | 0.978674 | 12.317789 |
| O | 4.958060 | 5.593062 | 9.690072 |

MacropaLaΔ_λδδ_λδδ_opt.log

SCF (RwB97XD) = -1861.91734482
E(SCF)+ZPE(0 K)= -1861.308114
H(298 K)= -1861.272975
G(298 K)= -1861.370481
Lowest Frequency = 30.0201cm-1

73

| | | | |
|---|---|---|---|
| C | 3.730162 | 0.922840 | 0.416774 |
| N | 2.866524 | 0.699215 | -0.762196 |
| O | 1.880228 | 1.875812 | 1.602149 |
| C | 2.998016 | 1.012594 | 1.747009 |
| C | 3.080221 | 1.737302 | -1.783106 |
| C | 1.876858 | 2.019225 | -2.653840 |
| O | 0.848120 | 2.562902 | -1.821877 |
| C | -0.190384 | 3.252426 | -2.499929 |
| C | -1.345471 | 2.337421 | -2.831394 |
| O | -1.880928 | 1.875051 | -1.602165 |
| O | -0.849109 | 2.562550 | 1.821866 |
| C | 0.189115 | 3.252496 | 2.499918 |
| C | 1.344580 | 2.337965 | 2.831379 |
| N | -2.866766 | 0.698058 | 0.762187 |
| C | -3.080888 | 1.736084 | 1.783069 |
| C | -1.877649 | 2.018480 | 2.653821 |
| C | -2.998328 | 1.011327 | -1.747024 |
| C | -3.730470 | 0.921310 | -0.416806 |
| C | 3.048727 | -0.645631 | -1.328123 |
| C | -3.048444 | -0.646845 | 1.328143 |
| C | -2.557686 | -1.724079 | 0.391412 |
| C | 2.558425 | -1.723044 | -0.391357 |
| C | -1.080148 | -2.244852 | -1.325175 |
| C | -1.540045 | -3.552203 | -1.403503 |
| C | -2.556343 | -3.939447 | -0.529804 |
| C | -3.088920 | -3.013941 | 0.364439 |
| C | 3.090212 | -3.012678 | -0.364347 |

| | | | |
|---|---|---|---|
| C | 2.558025 | -3.938389 | 0.529917 |
| C | 1.541553 | -3.551558 | 1.403597 |
| C | 1.081098 | -2.244406 | 1.325233 |
| La | -0.000124 | 0.746483 | 0.000006 |
| H | 4.285339 | 1.860486 | 0.274141 |
| H | 4.489541 | 0.127618 | 0.503444 |
| H | 3.691414 | 1.426908 | 2.498825 |
| H | 2.649973 | 0.032308 | 2.106001 |
| H | 0.197887 | 3.723052 | -3.417088 |
| H | -0.534855 | 4.049955 | -1.824333 |
| H | -1.011311 | 1.476902 | -3.437091 |
| H | -2.114334 | 2.898404 | -3.390740 |
| H | -0.199344 | 3.722956 | 3.417082 |
| H | 0.533257 | 4.050170 | 1.824327 |
| H | 2.113213 | 2.899266 | 3.390722 |
| H | 1.010775 | 1.477310 | 3.437077 |
| H | -2.649829 | 0.031177 | -2.105950 |
| H | -3.691887 | 1.425296 | -2.498881 |
| H | -4.286027 | 1.858737 | -0.274208 |
| H | -4.489527 | 0.125782 | -0.503475 |
| H | 4.110535 | -0.828622 | -1.582215 |
| H | 2.467345 | -0.726363 | -2.259658 |
| H | -4.110186 | -0.830255 | 1.582212 |
| H | -2.467054 | -0.727315 | 2.259696 |
| H | -1.104290 | -4.224823 | -2.142702 |
| H | -2.947920 | -4.958295 | -0.557246 |
| H | -3.901787 | -3.287051 | 1.038763 |
| H | 3.903200 | -3.285458 | -1.038658 |
| H | 2.950037 | -4.957069 | 0.557388 |
| H | 1.106079 | -4.224347 | 2.142808 |
| N | 1.560944 | -1.381209 | 0.423374 |
| N | -1.560356 | -1.381837 | -0.423333 |
| C | -0.012840 | -1.680961 | -2.261268 |
| O | 0.150449 | -0.406325 | -2.136922 |
| O | 0.598803 | -2.425882 | -2.999529 |
| C | 0.013544 | -1.680947 | 2.261307 |
| O | -0.150278 | -0.406382 | 2.136934 |
| O | -0.597789 | -2.426111 | 2.999579 |
| H | -2.163313 | 2.769293 | 3.408947 |
| H | -1.503688 | 1.113884 | 3.158890 |
| H | -3.938594 | 1.485117 | 2.436322 |
| H | -3.328114 | 2.674320 | 1.268595 |
| H | 2.162223 | 2.770143 | -3.408975 |
| H | 1.503241 | 1.114483 | -3.158902 |
| H | 3.327096 | 2.675644 | -1.268656 |
| H | 3.938010 | 1.486652 | -2.436371 |

MacropaLaΔ_λδλ_λδλ_opt.log

SCF (RwB97XD) = -1861.92270640
E(SCF)+ZPE(0 K)= -1861.314567
H(298 K)= -1861.278952
G(298 K)= -1861.378549
Lowest Frequency = 27.5355cm-1

73

| | | | |
|---|---|---|---|
| C | 3.691777 | 1.092107 | -0.298546 |

| | | | |
|---|---|---|---|
| N | 2.661753 | 0.595763 | -1.228713 |
| O | 2.047652 | 1.967097 | 1.189706 |
| C | 3.243704 | 1.201945 | 1.148384 |
| C | 2.537390 | 1.443664 | -2.421858 |
| C | 1.904346 | 2.792832 | -2.136950 |
| O | 0.567386 | 2.611586 | -1.686089 |
| C | -0.474256 | 3.312800 | -2.343612 |
| C | -1.669070 | 2.400302 | -2.486341 |
| O | -2.048039 | 1.966597 | -1.189842 |
| O | -0.567896 | 2.611628 | 1.685831 |
| C | 0.473588 | 3.313027 | 2.343413 |
| C | 1.668572 | 2.400758 | 2.486189 |
| N | -2.661838 | 0.595281 | 1.228654 |
| C | -2.537651 | 1.443333 | 2.421709 |
| C | -1.904910 | 2.792612 | 2.136646 |
| C | -3.243912 | 1.201166 | -1.148490 |
| C | -3.691979 | 1.091294 | 0.298440 |
| C | 2.893083 | -0.800081 | -1.625874 |
| C | -2.892843 | -0.800578 | 1.625949 |
| C | -2.589017 | -1.783832 | 0.523140 |
| C | 2.589473 | -1.783305 | -0.522980 |
| C | -1.296836 | -2.239941 | -1.353111 |
| C | -1.856242 | -3.502020 | -1.503123 |
| C | -2.837084 | -3.894410 | -0.593163 |
| C | -3.223498 | -3.022610 | 0.421871 |
| C | 3.224243 | -3.021924 | -0.421592 |
| C | 2.838022 | -3.893723 | 0.593517 |
| C | 1.857081 | -3.501478 | 1.503431 |
| C | 1.297381 | -2.239544 | 1.353299 |
| La | -0.000045 | 0.651522 | -0.000036 |
| H | 4.018494 | 2.091025 | -0.622726 |
| H | 4.590222 | 0.451547 | -0.332379 |
| H | 4.031751 | 1.713680 | 1.727320 |
| H | 3.064781 | 0.216270 | 1.607367 |
| H | 1.889690 | 0.911416 | -3.132233 |
| H | 3.519651 | 1.607227 | -2.907983 |
| H | 1.908927 | 3.387646 | -3.063345 |
| H | 2.455931 | 3.362541 | -1.371759 |
| H | -0.151048 | 3.628029 | -3.348275 |
| H | -0.737837 | 4.212445 | -1.762101 |
| H | -1.409511 | 1.523942 | -3.105675 |
| H | -2.498466 | 2.954647 | -2.958548 |
| H | 0.150276 | 3.628182 | 3.348066 |
| H | 0.737023 | 4.212728 | 1.761924 |
| H | 2.497850 | 2.955275 | 2.958401 |
| H | 1.409168 | 1.524362 | 3.105536 |
| H | -1.889826 | 0.911303 | 3.132134 |
| H | -3.519943 | 1.606730 | 2.907828 |
| H | -2.456607 | 3.362088 | 1.371366 |
| H | -1.909652 | 3.387552 | 3.062959 |
| H | -3.064750 | 0.215510 | -1.607418 |
| H | -4.032072 | 1.712685 | -1.727463 |
| H | -4.018987 | 2.090137 | 0.622560 |
| H | -4.590246 | 0.450486 | 0.332288 |
| H | 3.929905 | -0.948292 | -1.982843 |
| H | 2.221342 | -1.040921 | -2.465344 |
| H | -3.929628 | -0.948995 | 1.982942 |

| | | | |
|---|---|---|---|
| H | -2.221036 | -1.041186 | 2.465433 |
| H | -1.522899 | -4.135258 | -2.325666 |
| H | -3.310870 | -4.874209 | -0.681275 |
| H | -4.001400 | -3.299787 | 1.134919 |
| H | 4.002218 | -3.298984 | -1.134606 |
| H | 3.312035 | -4.873403 | 0.681723 |
| H | 1.523878 | -4.134717 | 2.326031 |
| N | 1.642152 | -1.428253 | 0.346023 |
| N | -1.641790 | -1.428640 | -0.345907 |
| C | -0.261600 | -1.676640 | -2.325301 |
| O | -0.067363 | -0.409878 | -2.188858 |
| O | 0.306106 | -2.424284 | -3.097400 |
| C | 0.262015 | -1.676394 | 2.325437 |
| O | 0.067482 | -0.409690 | 2.188876 |
| O | -0.305513 | -2.424096 | 3.097611 |

MacropaLaΔ_λλδ_λλδ_opt.log

SCF (RwB97XD) = -1861.91647434
E(SCF)+ZPE(0 K)= -1861.306363
H(298 K)= -1861.271504
G(298 K)= -1861.367597
Lowest Frequency = 37.6005cm-1

73

| | | | |
|---|---|---|---|
| C | -3.603668 | -0.823026 | 0.713637 |
| N | -2.863605 | -0.622783 | -0.549524 |
| O | -1.798665 | -2.267153 | 1.335435 |
| C | -2.720114 | -1.312199 | 1.856019 |
| C | -3.169788 | -1.704101 | -1.496152 |
| C | -2.142351 | -1.898828 | -2.585953 |
| O | -0.927774 | -2.358807 | -2.003494 |
| C | 0.059350 | -2.659599 | -2.972638 |
| C | 1.268936 | -3.204340 | -2.250283 |
| O | 1.803683 | -2.263196 | -1.335020 |
| O | 0.932969 | -2.356369 | 2.003917 |
| C | -0.053444 | -2.659225 | 2.973139 |
| C | -1.261783 | -3.206880 | 2.250902 |
| N | 2.864755 | -0.616133 | 0.549661 |
| C | 3.173468 | -1.696680 | 1.496353 |
| C | 2.146579 | -1.893721 | 2.586256 |
| C | 2.722984 | -1.306280 | -1.855786 |
| C | 3.605326 | -0.814770 | -0.713463 |
| C | -3.115921 | 0.705587 | -1.122054 |
| C | 3.114034 | 0.712866 | 1.122080 |
| C | 2.538163 | 1.793155 | 0.237337 |
| C | -2.542416 | 1.787288 | -0.237503 |
| C | 0.972653 | 2.274070 | -1.411044 |
| C | 1.376847 | 3.599501 | -1.491065 |
| C | 2.408211 | 4.017728 | -0.649306 |
| C | 3.012326 | 3.104917 | 0.212206 |
| C | -3.019556 | 3.097975 | -0.212570 |
| C | -2.417498 | 4.012295 | 0.648778 |
| C | -1.385176 | 3.596534 | 1.490587 |
| C | -0.977976 | 2.272012 | 1.410764 |
| La | 0.000765 | -0.721427 | 0.000075 |
| H | -4.429656 | -1.533850 | 0.550217 |

| | | | |
|---|---|---|---|
| H | -4.068677 | 0.119487 | 1.036732 |
| H | -3.342806 | -1.778850 | 2.636546 |
| H | -2.162067 | -0.476561 | 2.311877 |
| H | 2.163075 | -0.472039 | -2.311917 |
| H | 3.346807 | -1.771714 | -2.636137 |
| H | 4.433055 | -1.523535 | -0.549911 |
| H | 4.068020 | 0.128830 | -1.036701 |
| H | -4.197736 | 0.881347 | -1.278012 |
| H | -2.623997 | 0.778982 | -2.104190 |
| H | 4.195434 | 0.891076 | 1.278124 |
| H | 2.621813 | 0.785264 | 2.104144 |
| H | 0.886458 | 4.262974 | -2.203808 |
| H | 2.755465 | 5.052572 | -0.676384 |
| H | 3.836920 | 3.403674 | 0.860910 |
| H | -3.844846 | 3.394750 | -0.861298 |
| H | -2.767098 | 5.046352 | 0.675699 |
| H | -0.896286 | 4.261223 | 2.203226 |
| N | -1.527624 | 1.417054 | 0.542382 |
| N | 1.524239 | 1.420506 | -0.542525 |
| C | -0.122700 | 1.678221 | -2.291625 |
| O | -0.258476 | 0.403134 | -2.140251 |
| O | -0.783350 | 2.400557 | -3.010182 |
| C | 0.118720 | 1.678794 | 2.291443 |
| O | 0.257409 | 0.404003 | 2.140236 |
| O | 0.777704 | 2.402729 | 3.009921 |
| H | 2.524082 | -2.657899 | 3.287468 |
| H | 1.941598 | -0.966793 | 3.145705 |
| H | 4.161716 | -1.536673 | 1.970378 |
| H | 3.231904 | -2.633612 | 0.925404 |
| H | -2.518068 | -2.663809 | -3.287248 |
| H | -1.939335 | -0.971402 | -3.145299 |
| H | -3.226116 | -2.641134 | -0.925157 |
| H | -4.158363 | -1.546390 | -1.970263 |
| H | 0.304755 | -1.744962 | -3.541931 |
| H | -0.318967 | -3.421066 | -3.677394 |
| H | 2.038556 | -3.515031 | -2.975456 |
| H | 0.982918 | -4.083337 | -1.654141 |
| H | -0.300926 | -1.745033 | 3.542249 |
| H | 0.326627 | -3.419683 | 3.678041 |
| H | -0.973760 | -4.085353 | 1.654953 |
| H | -2.030696 | -3.519166 | 2.976139 |

MacropaLaΔ_λλλ_λλλ_opt.log

SCF (RwB97XD) = -1861.91950610
E(SCF)+ZPE(0 K)= -1861.310570
H(298 K)= -1861.275173
G(298 K)= -1861.374090
Lowest Frequency = 23.0001cm-1

73

| | | | |
|---|---|---|---|
| C | -3.691993 | 1.140098 | 0.469405 |
| N | -2.632338 | 0.600349 | 1.337075 |
| O | -2.075063 | 1.863640 | -1.138178 |
| C | -3.330868 | 1.208132 | -1.005055 |
| C | -2.446610 | 1.415644 | 2.545758 |
| C | -1.787460 | 2.755092 | 2.258551 |

| | | | |
|---|---|---|---|
| O | -0.441987 | 2.623123 | 1.817634 |
| C | 0.530268 | 2.522296 | 2.851589 |
| C | 1.894067 | 2.742503 | 2.239859 |
| O | 2.074675 | 1.863937 | 1.138395 |
| O | 0.441438 | 2.623449 | -1.817346 |
| C | -0.530806 | 2.522543 | -2.851304 |
| C | -1.894644 | 2.742387 | -2.239530 |
| N | 2.632230 | 0.601101 | -1.337035 |
| C | 2.446322 | 1.416497 | -2.545623 |
| C | 1.786878 | 2.755766 | -2.258258 |
| C | 3.330632 | 1.208740 | 1.005172 |
| C | 3.691760 | 1.140989 | -0.469300 |
| C | -2.868772 | -0.802791 | 1.699089 |
| C | 2.868985 | -0.801942 | -1.699213 |
| C | 2.601783 | -1.765330 | -0.569437 |
| C | -2.601351 | -1.765984 | 0.569198 |
| C | 1.345025 | -2.231464 | 1.326357 |
| C | 1.934422 | -3.479364 | 1.481452 |
| C | 2.913691 | -3.858895 | 0.565207 |
| C | 3.265369 | -2.988613 | -0.463157 |
| C | -3.264640 | -2.989416 | 0.462788 |
| C | -2.912759 | -3.859496 | -0.565676 |
| C | -1.933590 | -3.479625 | -1.481888 |
| C | -1.344495 | -2.231601 | -1.326657 |
| La | -0.000060 | 0.661214 | 0.000024 |
| H | -3.937663 | 2.160456 | 0.796728 |
| H | -4.622555 | 0.553621 | 0.573650 |
| H | -4.112453 | 1.778727 | -1.531229 |
| H | -3.262757 | 0.210303 | -1.464732 |
| H | 3.262758 | 0.210834 | 1.464717 |
| H | 4.112089 | 1.779447 | 1.531415 |
| H | 3.937181 | 2.161449 | -0.796489 |
| H | 4.622464 | 0.554750 | -0.573632 |
| H | -3.898520 | -0.950281 | 2.076194 |
| H | -2.181073 | -1.073537 | 2.515702 |
| H | 3.898767 | -0.949152 | -2.076333 |
| H | 2.181352 | -1.072748 | -2.515862 |
| H | 1.622732 | -4.111539 | 2.313241 |
| H | 3.411032 | -4.826569 | 0.657148 |
| H | 4.039123 | -3.255484 | -1.184665 |
| H | -4.038322 | -3.256557 | 1.184274 |
| H | -3.409866 | -4.827282 | -0.657721 |
| H | -1.621752 | -4.111633 | -2.313749 |
| N | -1.659445 | -1.418738 | -0.310079 |
| N | 1.659785 | -1.418411 | 0.309872 |
| C | 0.307219 | -1.694224 | 2.308980 |
| O | 0.071130 | -0.435704 | 2.167579 |
| O | -0.223705 | -2.451005 | 3.097952 |
| C | -0.306818 | -1.694003 | -2.309222 |
| O | -0.071018 | -0.435444 | -2.167673 |
| O | 0.224279 | -2.450568 | -3.098284 |
| H | 0.474015 | 1.524502 | 3.316983 |
| H | 0.351207 | 3.299773 | 3.613403 |
| H | 2.655668 | 2.557145 | 3.012876 |
| H | 1.998508 | 3.780327 | 1.882727 |
| H | -0.474345 | 1.524819 | -3.316823 |
| H | -0.351919 | 3.300153 | -3.613023 |

| | | | |
|---|---|---|---|
| H | -1.999294 | 3.780142 | -1.882259 |
| H | -2.656216 | 2.556975 | -3.012562 |
| H | 2.308981 | 3.302899 | -1.460407 |
| H | 1.817740 | 3.384488 | -3.163100 |
| H | 3.408138 | 1.598632 | -3.064531 |
| H | 1.805062 | 0.844831 | -3.230482 |
| H | -1.818467 | 3.383705 | 3.163463 |
| H | -2.309677 | 3.302200 | 1.460756 |
| H | -3.408465 | 1.597507 | 3.064689 |
| H | -1.805225 | 0.844038 | 3.230549 |

MacropaLuΔ_δδδ_δδδ_opt.log

SCF (RwB97XD) = -1870.28497173
E(SCF)+ZPE(0 K)= -1869.674292
H(298 K)= -1869.639441
G(298 K)= -1869.736259
Lowest Frequency = 36.4536cm-1

73

| | | | |
|---|---|---|---|
| C | 3.627013 | -0.782489 | -0.401162 |
| N | 2.854984 | -0.504974 | 0.806623 |
| O | 1.711033 | -1.615643 | -1.530065 |
| C | 3.047441 | -1.935852 | -1.185747 |
| C | 3.107820 | -1.496244 | 1.853620 |
| C | 1.832360 | -2.045088 | 2.452083 |
| O | 0.990626 | -2.478435 | 1.371433 |
| C | 0.054351 | -3.478900 | 1.724014 |
| C | -1.154123 | -2.866694 | 2.388602 |
| O | -1.680886 | -1.904882 | 1.493283 |
| O | -0.933297 | -2.506155 | -1.464281 |
| C | 0.091328 | -3.256691 | -2.097034 |
| C | 1.163341 | -2.332691 | -2.625401 |
| N | -2.835003 | -0.556057 | -0.607922 |
| C | -3.158934 | -1.644339 | -1.530556 |
| C | -1.975147 | -2.090251 | -2.354884 |
| C | -3.094413 | -1.856564 | 1.484268 |
| C | -3.559716 | -0.678590 | 0.656984 |
| C | 3.038759 | 0.868477 | 1.266325 |
| C | -3.057653 | 0.763986 | -1.204041 |
| C | -2.441208 | 1.840092 | -0.341803 |
| C | 2.446757 | 1.853794 | 0.286177 |
| C | -0.933287 | 2.262081 | 1.371043 |
| C | -1.317737 | 3.591897 | 1.467133 |
| C | -2.294208 | 4.049579 | 0.582262 |
| C | -2.884404 | 3.161724 | -0.315368 |
| C | 2.923882 | 3.158768 | 0.156933 |
| C | 2.360027 | 3.993536 | -0.803785 |
| C | 1.367442 | 3.495371 | -1.646436 |
| C | 0.949478 | 2.186629 | -1.452798 |
| Lu | -0.015566 | -0.559379 | 0.017692 |
| H | 0.523143 | -4.235905 | 2.373509 |
| H | -0.255361 | -3.965252 | 0.787684 |
| H | -0.899275 | -2.379574 | 3.344661 |
| H | -1.895107 | -3.658022 | 2.588586 |
| H | -0.331010 | -3.880169 | -2.901524 |
| H | 0.518154 | -3.925870 | -1.334087 |

| | | | |
|---|---|---|---|
| H | 1.945243 | -2.906455 | -3.145736 |
| H | 0.739193 | -1.595330 | -3.322252 |
| H | 4.110003 | 1.109168 | 1.415334 |
| H | 2.536461 | 0.995139 | 2.237311 |
| H | -4.138445 | 0.971291 | -1.330268 |
| H | -2.593356 | 0.796235 | -2.200580 |
| H | -0.857713 | 4.226296 | 2.225096 |
| H | -2.620140 | 5.091266 | 0.611815 |
| H | -3.684638 | 3.484683 | -0.982794 |
| H | 3.731757 | 3.508433 | 0.801458 |
| H | 2.713873 | 5.020785 | -0.911961 |
| H | 0.915408 | 4.081707 | -2.446701 |
| N | 1.449183 | 1.411465 | -0.483081 |
| N | -1.451240 | 1.439525 | 0.455730 |
| C | 0.087002 | 1.605906 | 2.289551 |
| O | 0.261006 | 0.353611 | 2.026644 |
| O | 0.661584 | 2.257773 | 3.136574 |
| C | -0.103830 | 1.524312 | -2.328767 |
| O | -0.330177 | 0.295754 | -2.004465 |
| O | -0.656793 | 2.153373 | -3.206803 |
| H | 4.691336 | -0.991650 | -0.175412 |
| H | 3.642522 | -2.078042 | -2.102239 |
| H | 3.070736 | -2.880116 | -0.612989 |
| H | -4.653269 | -0.767070 | 0.501372 |
| H | -3.390801 | 0.235657 | 1.238495 |
| H | -3.478013 | -1.740206 | 2.512078 |
| H | -3.480407 | -2.816219 | 1.095043 |
| H | -1.605364 | -1.277873 | -2.997510 |
| H | -2.275481 | -2.948809 | -2.977574 |
| H | -3.508247 | -2.509760 | -0.951718 |
| H | -3.985604 | -1.368730 | -2.211310 |
| H | 2.067220 | -2.911342 | 3.091347 |
| H | 1.307199 | -1.276760 | 3.035707 |
| H | 3.731579 | -1.081177 | 2.664393 |
| H | 3.679081 | -2.333710 | 1.426853 |
| H | 3.603013 | 0.103589 | -1.049219 |

MacropaLuΔ_δδλ_δδλ_opt.log

SCF (RwB97XD) = -1870.28291903
E(SCF)+ZPE(0 K)= -1869.672440
H(298 K)= -1869.637477
G(298 K)= -1869.734813
Lowest Frequency = 30.6315cm-1

73

| | | | |
|---|---|---|---|
| C | 3.541809 | 1.028165 | -0.216662 |
| N | 2.624816 | 0.855470 | 0.909629 |
| O | 2.531224 | -0.915063 | -1.190393 |
| C | 3.751141 | -0.212642 | -1.062361 |
| C | 3.144615 | 0.058207 | 2.016859 |
| C | 3.081519 | -1.444786 | 1.795859 |
| O | 1.751794 | -1.846456 | 1.495926 |
| C | 1.175797 | -2.879744 | 2.281033 |
| C | -0.233099 | -2.460656 | 2.633192 |
| O | -0.945134 | -2.146962 | 1.448346 |
| O | 0.494116 | -2.722217 | -1.185194 |

| | | | |
|---|---|---|---|
| C | 1.828364 | -3.156238 | -1.394747 |
| C | 2.599204 | -2.038454 | -2.051597 |
| N | -2.090716 | -1.700722 | -1.082325 |
| C | -1.635596 | -2.404110 | -2.276421 |
| C | -0.476664 | -3.344036 | -2.023594 |
| C | -1.745422 | -3.179126 | 0.907740 |
| C | -2.719565 | -2.553450 | -0.072946 |
| C | 2.187406 | 2.179219 | 1.372285 |
| C | -2.987137 | -0.607934 | -1.468447 |
| C | -3.059407 | 0.455187 | -0.402711 |
| C | 1.266134 | 2.814577 | 0.359428 |
| C | -2.005652 | 1.461059 | 1.397133 |
| C | -3.068606 | 2.325212 | 1.616848 |
| C | -4.170364 | 2.235207 | 0.767965 |
| C | -4.177819 | 1.274468 | -0.238733 |
| C | 1.083039 | 4.192194 | 0.241040 |
| C | 0.252515 | 4.675096 | -0.767935 |
| C | -0.333295 | 3.782638 | -1.665782 |
| C | -0.093694 | 2.428711 | -1.479312 |
| Lu | 0.252705 | -0.453512 | 0.001550 |
| H | 2.518305 | 0.281265 | 2.890810 |
| H | 4.189330 | 0.331057 | 2.269664 |
| H | 3.418300 | -1.938019 | 2.720355 |
| H | 3.743796 | -1.789182 | 0.989227 |
| H | 1.756872 | -3.034165 | 3.203236 |
| H | 1.186521 | -3.822601 | 1.705986 |
| H | -0.206651 | -1.539140 | 3.228844 |
| H | -0.747874 | -3.253546 | 3.198233 |
| H | 1.851898 | -4.066851 | -2.011760 |
| H | 2.268944 | -3.391475 | -0.414922 |
| H | 3.646388 | -2.348217 | -2.201462 |
| H | 2.163130 | -1.774864 | -3.029346 |
| H | -1.300961 | -1.633117 | -2.980787 |
| H | -2.451449 | -2.985564 | -2.751572 |
| H | -0.791295 | -4.294232 | -1.558712 |
| H | -0.026819 | -3.590218 | -2.998506 |
| H | 3.049073 | 2.849670 | 1.555357 |
| H | 1.644408 | 2.072024 | 2.321795 |
| H | -4.005941 | -0.977978 | -1.692006 |
| H | -2.609395 | -0.143594 | -2.391991 |
| H | -3.012028 | 3.029621 | 2.447065 |
| H | -5.030511 | 2.893791 | 0.904206 |
| H | -5.040808 | 1.154327 | -0.895588 |
| H | 1.590293 | 4.872391 | 0.926862 |
| H | 0.087449 | 5.749494 | -0.871191 |
| H | -0.953889 | 4.102817 | -2.503046 |
| N | 0.643966 | 1.971313 | -0.463929 |
| N | -1.991716 | 0.582372 | 0.387004 |
| C | -0.786241 | 1.422640 | 2.305070 |
| O | 0.022982 | 0.460500 | 2.023709 |
| O | -0.643210 | 2.259890 | 3.173501 |
| C | -0.614870 | 1.343102 | -2.409498 |
| O | -0.190971 | 0.167463 | -2.091505 |
| O | -1.373866 | 1.624231 | -3.315440 |
| H | 3.117697 | 1.804808 | -0.865267 |
| H | 4.532900 | 1.394821 | 0.117403 |
| H | 4.119551 | 0.094986 | -2.055649 |

| | | | |
|---|---|---|---|
| H | 4.513639 | -0.883256 | -0.626981 |
| H | -3.333385 | -3.351108 | -0.535480 |
| H | -3.402487 | -1.927178 | 0.518421 |
| H | -2.324463 | -3.660001 | 1.713834 |
| H | -1.109136 | -3.952322 | 0.448530 |

MacropaLuΔ_δλδ_δλδ_opt.log

SCF (RwB97XD) = -1870.28136799
E(SCF)+ZPE(0 K)= -1869.670420
H(298 K)= -1869.635350
G(298 K)= -1869.733738
Lowest Frequency = 15.8167cm-1

73

| | | | |
|---|---|---|---|
| C | 3.516896 | -0.736272 | -0.691439 |
| N | 2.889332 | -0.476694 | 0.599706 |
| O | 1.599352 | -2.048881 | -1.306442 |
| C | 3.018969 | -2.022945 | -1.307490 |
| C | 3.302227 | -1.447888 | 1.613150 |
| C | 2.174918 | -1.845108 | 2.530222 |
| O | 1.102114 | -2.356713 | 1.743074 |
| C | 0.165840 | -3.034400 | 2.548542 |
| C | -1.052381 | -3.319340 | 1.720358 |
| O | -1.582553 | -2.083381 | 1.266141 |
| O | -1.105046 | -2.349679 | -1.753199 |
| C | -0.161732 | -2.933299 | -2.621623 |
| C | 1.077225 | -3.255135 | -1.840870 |
| N | -2.891619 | -0.486054 | -0.545427 |
| C | -3.316849 | -1.488050 | -1.521693 |
| C | -2.221563 | -1.855671 | -2.487529 |
| C | -2.994086 | -1.995239 | 1.393809 |
| C | -3.490317 | -0.710414 | 0.766456 |
| C | 3.118589 | 0.891503 | 1.056666 |
| C | -3.133033 | 0.868121 | -1.038407 |
| C | -2.449861 | 1.896657 | -0.174570 |
| C | 2.444693 | 1.896725 | 0.158656 |
| C | -0.838273 | 2.258116 | 1.448765 |
| C | -1.196345 | 3.589148 | 1.600630 |
| C | -2.219964 | 4.082281 | 0.791138 |
| C | -2.874332 | 3.222978 | -0.087359 |
| C | 2.872627 | 3.220641 | 0.050853 |
| C | 2.225975 | 4.067079 | -0.845148 |
| C | 1.203262 | 3.563426 | -1.648866 |
| C | 0.841103 | 2.235910 | -1.477442 |
| Lu | -0.003389 | -0.570104 | 0.000897 |
| H | 4.201184 | 1.123142 | 1.109872 |
| H | 2.710963 | 1.004595 | 2.073361 |
| H | -4.216938 | 1.094769 | -1.084207 |
| H | -2.737105 | 0.954347 | -2.062308 |
| H | -0.680105 | 4.196844 | 2.344129 |
| H | -2.529699 | 5.126570 | 0.866390 |
| H | -3.710122 | 3.569666 | -0.696846 |
| H | 3.705028 | 3.575426 | 0.660345 |
| H | 2.538907 | 5.109146 | -0.936256 |
| H | 0.688518 | 4.160450 | -2.401977 |
| N | 1.418745 | 1.447427 | -0.564054 |

| | | | |
|---|---|---|---|
| N | -1.419588 | 1.459204 | 0.548037 |
| C | 0.224324 | 1.571796 | 2.289367 |
| O | 0.356018 | 0.323008 | 1.994125 |
| O | 0.865074 | 2.196010 | 3.110063 |
| C | -0.231171 | 1.549565 | -2.306082 |
| O | -0.374950 | 0.306106 | -1.995764 |
| O | -0.869388 | 2.170750 | -3.130918 |
| H | 4.622206 | -0.788432 | -0.621261 |
| H | 3.385203 | -2.090852 | -2.345942 |
| H | 3.390517 | -2.909553 | -0.765989 |
| H | -4.597651 | -0.747498 | 0.726454 |
| H | -3.218465 | 0.126946 | 1.421338 |
| H | -3.270846 | -2.010429 | 2.461538 |
| H | -3.447974 | -2.882326 | 0.920837 |
| H | -1.894382 | -0.990420 | -3.084127 |
| H | -2.588972 | -2.647528 | -3.162964 |
| H | -3.623340 | -2.400983 | -0.995635 |
| H | -4.201418 | -1.148001 | -2.091794 |
| H | 2.530933 | -2.634575 | 3.214178 |
| H | 1.803957 | -0.991088 | 3.116546 |
| H | 4.142772 | -1.065111 | 2.220703 |
| H | 3.672151 | -2.354814 | 1.117845 |
| H | 0.870760 | -3.962218 | -1.017798 |
| H | 1.811114 | -3.722279 | -2.515816 |
| H | 0.088607 | -2.224906 | -3.430772 |
| H | -0.570942 | -3.855618 | -3.071980 |
| H | 3.275253 | 0.090490 | -1.371672 |
| H | -1.791801 | -3.853174 | 2.336208 |
| H | -0.813434 | -3.951192 | 0.847179 |
| H | 0.594867 | -3.978341 | 2.930515 |
| H | -0.116317 | -2.401569 | 3.408351 |

MacropaLuΔ_δλλ_δλλ_opt.log

SCF (RwB97XD) = -1870.28676273
E(SCF)+ZPE(0 K)= -1869.675879
H(298 K)= -1869.641074
G(298 K)= -1869.737583
Lowest Frequency = 37.4453cm-1

73

| | | | |
|---|---|---|---|
| C | -3.647318 | -0.672637 | 0.278217 |
| N | -2.761806 | -0.468459 | -0.867040 |
| O | -1.857133 | -1.829573 | 1.374330 |
| C | -3.257306 | -1.821763 | 1.183629 |
| C | -2.900079 | -1.463455 | -1.925173 |
| C | -2.146847 | -2.752232 | -1.635026 |
| O | -0.755635 | -2.528975 | -1.451547 |
| C | 0.016618 | -2.631155 | -2.642199 |
| C | 1.446000 | -2.864038 | -2.244110 |
| O | 1.857755 | -1.828598 | -1.374674 |
| O | 0.756500 | -2.529080 | 1.451020 |
| C | -0.015732 | -2.631880 | 2.641629 |
| C | -1.445036 | -2.865124 | 2.243470 |
| N | 2.761981 | -0.467740 | 0.867026 |
| C | 2.900564 | -1.462932 | 1.924933 |
| C | 2.147789 | -2.751901 | 1.634463 |

| | | | |
|---|---|---|---|
| C | 3.257923 | -1.820397 | -1.183956 |
| C | 3.647571 | -0.671365 | -0.278266 |
| C | -2.938827 | 0.896182 | -1.369715 |
| C | 2.938528 | 0.896848 | 1.370011 |
| C | 2.368500 | 1.892881 | 0.388109 |
| C | -2.369083 | 1.892192 | -0.387627 |
| C | 0.965444 | 2.189850 | -1.433407 |
| C | 1.338532 | 3.517933 | -1.583867 |
| C | 2.254217 | 4.043470 | -0.672814 |
| C | 2.796188 | 3.218190 | 0.309585 |
| C | -2.797161 | 3.217360 | -0.308841 |
| C | -2.255431 | 4.042604 | 0.673720 |
| C | -1.339600 | 3.517152 | 1.584676 |
| C | -0.966116 | 2.189212 | 1.433950 |
| Lu | 0.000109 | -0.584643 | -0.000030 |
| H | -4.005988 | 1.131340 | -1.547739 |
| H | -2.416762 | 1.003439 | -2.331123 |
| H | 4.005603 | 1.132321 | 1.548136 |
| H | 2.416376 | 1.003718 | 2.331417 |
| H | 0.914225 | 4.098007 | -2.403659 |
| H | 2.568955 | 5.086514 | -0.745029 |
| H | 3.548630 | 3.590700 | 1.006344 |
| H | -3.549712 | 3.589786 | -1.005527 |
| H | -2.570475 | 5.085541 | 0.746140 |
| H | -0.915474 | 4.097186 | 2.404590 |
| N | -1.437512 | 1.424199 | 0.444717 |
| N | 1.437073 | 1.424777 | -0.444333 |
| C | -0.003062 | 1.487657 | -2.372932 |
| O | -0.214461 | 0.256617 | -2.052044 |
| O | -0.517565 | 2.097898 | -3.288089 |
| C | 0.002581 | 1.487107 | 2.373348 |
| O | 0.214389 | 0.256213 | 2.052171 |
| O | 0.516851 | 2.097294 | 3.288671 |
| H | -4.698440 | -0.818571 | -0.041083 |
| H | -3.763092 | -1.692135 | 2.156227 |
| H | -3.584072 | -2.799039 | 0.786551 |
| H | 4.698736 | -0.817046 | 0.041009 |
| H | 3.623353 | 0.244709 | -0.881605 |
| H | 3.763677 | -1.690376 | -2.156518 |
| H | 3.584994 | -2.797667 | -0.787115 |
| H | -1.555312 | -3.839318 | 1.732101 |
| H | -2.072623 | -2.873971 | 3.150928 |
| H | 0.080847 | -1.703768 | 3.226154 |
| H | 0.330086 | -3.491282 | 3.238058 |
| H | -3.623384 | 0.243300 | 0.881773 |
| H | 2.073590 | -2.872417 | -3.151571 |
| H | 1.556600 | -3.838340 | -1.733020 |
| H | -0.328916 | -3.490496 | -3.238881 |
| H | -0.080274 | -1.702892 | -3.226436 |
| H | -3.961875 | -1.709900 | -2.128410 |
| H | -2.484487 | -1.027169 | -2.843883 |
| H | -2.298317 | -3.455395 | -2.469216 |
| H | -2.500056 | -3.254142 | -0.728892 |
| H | 2.501186 | -3.253475 | 0.728216 |
| H | 2.299489 | -3.455207 | 2.468490 |
| H | 2.484791 | -1.027002 | 2.843730 |
| H | 3.962438 | -1.709060 | 2.128145 |

MacropaLuΔ_δλλ_λδλ_opt.log

SCF (RwB97XD) = -1870.28346304
E(SCF)+ZPE(0 K)= -1869.672200
H(298 K)= -1869.637390
G(298 K)= -1869.733918
Lowest Frequency = 39.2156cm-1

73

| | | | |
|---|---|---|---|
| Lu | 0.921946 | 3.984196 | 9.233439 |
| N | 3.057879 | 2.678749 | 9.289645 |
| C | 3.137795 | 1.356545 | 9.449031 |
| C | 4.307069 | 0.763843 | 9.925505 |
| H | 4.352316 | -0.317126 | 10.065746 |
| C | 5.405791 | 1.572044 | 10.206392 |
| H | 6.327009 | 1.128134 | 10.589140 |
| C | 5.332440 | 2.942503 | 9.959047 |
| C | 4.130541 | 3.451545 | 9.488924 |
| C | 3.923738 | 4.921314 | 9.157196 |
| O | 2.731495 | 5.179875 | 8.740037 |
| H | 6.171719 | 3.621243 | 10.112156 |
| N | 1.952537 | 4.393440 | 11.523286 |
| C | 2.581549 | 5.507395 | 11.895755 |
| C | 3.483200 | 5.501085 | 12.960739 |
| H | 3.999361 | 6.419659 | 13.243906 |
| C | 3.701694 | 4.311403 | 13.650139 |
| H | 4.410995 | 4.282312 | 14.479734 |
| C | 2.980424 | 3.170869 | 13.297005 |
| C | 2.103115 | 3.267059 | 12.227041 |
| C | 1.226355 | 2.119930 | 11.757448 |
| O | 0.466322 | 2.450603 | 10.769345 |
| H | 3.070115 | 2.222010 | 13.826370 |
| C | 1.919139 | 0.564068 | 9.043792 |
| H | 2.241239 | -0.420534 | 8.650628 |
| H | 1.305238 | 0.373633 | 9.937069 |
| N | 1.129199 | 1.297443 | 8.055718 |
| C | 1.855290 | 1.424270 | 6.794876 |
| H | 1.877145 | 0.470030 | 6.231782 |
| H | 2.896506 | 1.693770 | 7.012612 |
| C | 1.269885 | 2.497863 | 5.911336 |
| H | 1.932842 | 2.643676 | 5.041233 |
| H | 0.273058 | 2.226501 | 5.519812 |
| O | 1.168814 | 3.707963 | 6.637503 |
| C | 0.813693 | 4.787096 | 5.797472 |
| H | -0.219528 | 4.644550 | 5.431231 |
| H | 1.489628 | 4.828953 | 4.926174 |
| C | 0.931040 | 6.067339 | 6.569177 |
| H | 1.973856 | 6.250485 | 6.871174 |
| H | 0.569607 | 6.895347 | 5.938341 |
| O | 0.113204 | 5.947036 | 7.726690 |
| C | -0.221050 | 7.190773 | 8.325302 |
| H | -1.244401 | 7.096253 | 8.700522 |
| H | -0.242943 | 7.974919 | 7.552083 |
| C | 0.769179 | 7.598425 | 9.403933 |
| H | 1.750875 | 7.723683 | 8.927175 |
| H | 0.468657 | 8.588890 | 9.802615 |

| | | | |
|---|---|---|---|
| N | 0.931091 | 6.608896 | 10.463730 |
| C | 2.233423 | 6.751282 | 11.118264 |
| H | 2.255595 | 7.625068 | 11.798287 |
| H | 3.007348 | 6.912820 | 10.353758 |
| C | -0.126781 | 6.606576 | 11.471247 |
| H | -0.281534 | 7.613587 | 11.908172 |
| H | 0.197284 | 5.950494 | 12.288720 |
| C | -1.462269 | 6.097692 | 10.964590 |
| H | -2.077508 | 5.775521 | 11.821653 |
| H | -2.025476 | 6.889042 | 10.442167 |
| O | -1.259813 | 5.004202 | 10.084939 |
| C | -2.439111 | 4.580883 | 9.417557 |
| H | -2.449085 | 5.004320 | 8.397454 |
| H | -3.332737 | 4.936261 | 9.953157 |
| C | -2.458410 | 3.081638 | 9.385163 |
| H | -2.441059 | 2.692152 | 10.418024 |
| H | -3.381170 | 2.731316 | 8.889296 |
| O | -1.322798 | 2.616871 | 8.687147 |
| C | -1.221787 | 1.198774 | 8.812523 |
| H | -2.195829 | 0.746039 | 8.560083 |
| H | -0.966872 | 0.957989 | 9.855659 |
| C | -0.183822 | 0.689870 | 7.848844 |
| H | -0.136409 | -0.413286 | 7.920144 |
| H | -0.526414 | 0.920500 | 6.831550 |
| O | 1.306152 | 1.025270 | 12.276476 |
| O | 4.822357 | 5.719953 | 9.328484 |

MacropaLuΔ_λδδ_λδδ_opt.log

SCF (RwB97XD) = -1870.28214670
E(SCF)+ZPE(0 K)= -1869.671664
H(298 K)= -1869.636835
G(298 K)= -1869.733106
Lowest Frequency = 38.1730cm-1

73

| | | | |
|---|---|---|---|
| C | 3.718138 | 0.695911 | 0.443107 |
| N | 2.876414 | 0.574705 | -0.757266 |
| O | 1.814861 | 1.774448 | 1.401363 |
| C | 2.921674 | 0.947890 | 1.719611 |
| C | 3.075603 | 1.686038 | -1.686852 |
| C | 1.820362 | 2.097131 | -2.418269 |
| O | 0.843674 | 2.492586 | -1.444958 |
| C | -0.176150 | 3.344772 | -1.940458 |
| C | -1.324271 | 2.534037 | -2.485893 |
| O | -1.815396 | 1.774074 | -1.401150 |
| O | -0.844424 | 2.492151 | 1.445248 |
| C | 0.175140 | 3.344584 | 1.940860 |
| C | 1.323505 | 2.534129 | 2.486199 |
| N | -2.876549 | 0.573695 | 0.757314 |
| C | -3.076102 | 1.684875 | 1.687006 |
| C | -1.821009 | 2.096288 | 2.418497 |
| C | -2.921932 | 0.947192 | -1.719516 |
| C | -3.718310 | 0.694752 | -0.443049 |
| C | 2.996070 | -0.739733 | -1.383958 |
| C | -2.995804 | -0.740847 | 1.383868 |
| C | -2.435991 | -1.797128 | 0.460088 |

| | | | |
|---|---|---|---|
| C | 2.436600 | -1.796291 | -0.460287 |
| C | -1.004784 | -2.200414 | -1.322757 |
| C | -1.403424 | -3.524743 | -1.426360 |
| C | -2.348509 | -3.991273 | -0.511902 |
| C | -2.890767 | -3.115838 | 0.425769 |
| C | 2.891818 | -3.114853 | -0.426104 |
| C | 2.349845 | -3.990569 | 0.511469 |
| C | 1.404596 | -3.524452 | 1.425967 |
| C | 1.005514 | -2.200245 | 1.322503 |
| Lu | -0.000088 | 0.614141 | 0.000037 |
| H | 4.436557 | 1.517135 | 0.302364 |
| H | 4.323680 | -0.212093 | 0.590180 |
| H | 3.564552 | 1.457385 | 2.455832 |
| H | 2.553684 | 0.017608 | 2.177314 |
| H | 0.228649 | 4.023480 | -2.707019 |
| H | -0.527471 | 3.948693 | -1.090494 |
| H | -0.992520 | 1.864435 | -3.298601 |
| H | -2.112233 | 3.203613 | -2.871968 |
| H | -0.229869 | 4.023075 | 2.707502 |
| H | 0.526282 | 3.948713 | 1.090970 |
| H | 2.111263 | 3.203899 | 2.872354 |
| H | 0.991957 | 1.864330 | 3.298827 |
| H | -2.553629 | 0.017103 | -2.177359 |
| H | -3.564983 | 1.456581 | -2.455660 |
| H | -4.437042 | 1.515685 | -0.302200 |
| H | -4.323508 | -0.213461 | -0.590247 |
| H | 4.047708 | -0.985172 | -1.629841 |
| H | 2.426774 | -0.754993 | -2.325294 |
| H | -4.047371 | -0.986640 | 1.629704 |
| H | -2.426524 | -0.756034 | 2.325213 |
| H | -0.978462 | -4.148540 | -2.213060 |
| H | -2.685413 | -5.029251 | -0.547953 |
| H | -3.663243 | -3.444881 | 1.122259 |
| H | 3.664411 | -3.443563 | -1.122621 |
| H | 2.687097 | -5.028438 | 0.547413 |
| H | 0.979837 | -4.148475 | 2.212598 |
| N | 1.479745 | -1.386293 | 0.373474 |
| N | -1.479280 | -1.386723 | -0.373638 |
| C | -0.019963 | -1.543510 | -2.280629 |
| O | 0.159347 | -0.287426 | -2.030669 |
| O | 0.520699 | -2.194510 | -3.149135 |
| C | 0.020474 | -1.543775 | 2.280447 |
| O | -0.159244 | -0.287720 | 2.030636 |
| O | -0.519979 | -2.195054 | 3.148873 |
| H | -2.052877 | 2.961953 | 3.059522 |
| H | -1.413414 | 1.280355 | 3.033311 |
| H | -3.864079 | 1.463531 | 2.432766 |
| H | -3.413152 | 2.555381 | 1.108942 |
| H | 2.051942 | 2.962935 | -3.059211 |
| H | 1.413011 | 1.281131 | -3.033157 |
| H | 3.412402 | 2.556591 | -1.108713 |
| H | 3.863626 | 1.465008 | -2.432656 |

MacropaLuΔ_λδλ_λδλ_opt.log

SCF (RwB97XD) = -1870.29245718
E(SCF)+ZPE(0 K)= -1869.681866

H(298 K)= -1869.647122
G(298 K)= -1869.743498
Lowest Frequency = 35.3387cm-1

73

| | | | |
|---|---|---|---|
| C | 3.690800 | 0.914394 | -0.247602 |
| N | 2.660432 | 0.532893 | -1.222311 |
| O | 1.952936 | 1.808312 | 1.098980 |
| C | 3.161426 | 1.068259 | 1.169590 |
| C | 2.543663 | 1.479541 | -2.327949 |
| C | 1.936823 | 2.794637 | -1.884546 |
| O | 0.613689 | 2.578448 | -1.403260 |
| C | -0.426184 | 3.373727 | -1.945597 |
| C | -1.588143 | 2.477949 | -2.290054 |
| O | -1.953549 | 1.807842 | -1.098555 |
| O | -0.614598 | 2.577804 | 1.403912 |
| C | 0.424958 | 3.373383 | 1.946415 |
| C | 1.587286 | 2.477995 | 2.290642 |
| N | -2.660555 | 0.531520 | 1.222400 |
| C | -2.544151 | 1.477913 | 2.328293 |
| C | -1.937818 | 2.793362 | 1.885243 |
| C | -3.161748 | 1.067335 | -1.169360 |
| C | -3.691064 | 0.912890 | 0.247789 |
| C | 2.811219 | -0.842780 | -1.687983 |
| C | -2.810809 | -0.844340 | 1.687688 |
| C | -2.397525 | -1.824763 | 0.618467 |
| C | 2.398326 | -1.823664 | -0.619036 |
| C | -1.155874 | -2.152022 | -1.311373 |
| C | -1.600243 | -3.458240 | -1.452374 |
| C | -2.477344 | -3.955358 | -0.488384 |
| C | -2.901321 | -3.124691 | 0.544720 |
| C | 2.902640 | -3.123412 | -0.545656 |
| C | 2.478989 | -3.954540 | 0.487211 |
| C | 1.601689 | -3.458042 | 1.451340 |
| C | 1.156803 | -2.151960 | 1.310709 |
| Lu | -0.000069 | 0.620203 | 0.000058 |
| H | 4.143615 | 1.870378 | -0.552212 |
| H | 4.513969 | 0.180247 | -0.236058 |
| H | 3.898196 | 1.620579 | 1.776063 |
| H | 2.971518 | 0.101060 | 1.658672 |
| H | 1.877334 | 1.031504 | -3.077294 |
| H | 3.520992 | 1.683047 | -2.810053 |
| H | 1.909550 | 3.485259 | -2.740507 |
| H | 2.522669 | 3.272428 | -1.083673 |
| H | -0.082830 | 3.892034 | -2.853477 |
| H | -0.729916 | 4.130636 | -1.202786 |
| H | -1.299393 | 1.741287 | -3.058349 |
| H | -2.431489 | 3.087637 | -2.656729 |
| H | 0.081408 | 3.891337 | 2.854423 |
| H | 0.728369 | 4.130589 | 1.203776 |
| H | 2.430394 | 3.087937 | 2.657440 |
| H | 1.298852 | 1.741041 | 3.058775 |
| H | -1.877646 | 1.029937 | 3.077517 |
| H | -3.521557 | 1.680913 | 2.810454 |
| H | -2.523842 | 3.271137 | 1.084491 |
| H | -1.910815 | 3.483766 | 2.741389 |
| H | -2.971464 | 0.100339 | -1.658696 |

| | | | |
|---|---|---|---|
| H | -3.898735 | 1.619527 | -1.775687 |
| H | -4.144242 | 1.868620 | 0.552654 |
| H | -4.513955 | 0.178434 | 0.236047 |
| H | 3.849943 | -1.059683 | -2.003025 |
| H | 2.169127 | -1.001769 | -2.567388 |
| H | -3.849451 | -1.061738 | 2.002657 |
| H | -2.168668 | -1.003324 | 2.567056 |
| H | -1.263744 | -4.042441 | -2.309184 |
| H | -2.851578 | -4.978840 | -0.556530 |
| H | -3.618263 | -3.473611 | 1.289504 |
| H | 3.619721 | -3.471837 | -1.290537 |
| H | 2.853628 | -4.977893 | 0.555069 |
| H | 1.265421 | -4.042620 | 2.307984 |
| N | 1.514230 | -1.381563 | 0.276180 |
| N | -1.513607 | -1.382059 | -0.276627 |
| C | -0.254069 | -1.471326 | -2.330851 |
| O | -0.045372 | -0.225827 | -2.068289 |
| O | 0.206998 | -2.107366 | -3.257082 |
| C | 0.254734 | -1.471899 | 2.330376 |
| O | 0.045569 | -0.226407 | 2.068161 |
| O | -0.206093 | -2.108375 | 3.256426 |

MacropaLuΔ_λλδ_λλδ_opt.log

SCF (RwB97XD) = -1870.27650515
E(SCF)+ZPE(0 K)= -1869.666676
H(298 K)= -1869.631563
G(298 K)= -1869.728969
Lowest Frequency = 39.4027cm-1

73

| | | | |
|---|---|---|---|
| C | -3.585944 | -0.671509 | 0.718957 |
| N | -2.890821 | -0.519569 | -0.566123 |
| O | -1.674718 | -2.047398 | 1.141688 |
| C | -2.661563 | -1.251323 | 1.794640 |
| C | -3.213243 | -1.622345 | -1.469203 |
| C | -2.124145 | -1.936975 | -2.461950 |
| O | -0.961414 | -2.364214 | -1.760023 |
| C | 0.015583 | -2.880732 | -2.638654 |
| C | 1.227334 | -3.205638 | -1.805992 |
| O | 1.680649 | -2.041031 | -1.142907 |
| O | 0.968431 | -2.362287 | 1.758758 |
| C | -0.006915 | -2.882826 | 2.636830 |
| C | -1.217508 | -3.211056 | 1.803782 |
| N | 2.892359 | -0.510957 | 0.566281 |
| C | 3.218000 | -1.613120 | 1.468915 |
| C | 2.129618 | -1.931677 | 2.461204 |
| C | 2.664910 | -1.241096 | -1.795086 |
| C | 3.587903 | -0.660141 | -0.718881 |
| C | -3.090810 | 0.797887 | -1.159910 |
| C | 3.088076 | 0.806840 | 1.160644 |
| C | 2.438371 | 1.853249 | 0.287184 |
| C | -2.444363 | 1.845942 | -0.286002 |
| C | 0.893932 | 2.219612 | -1.403679 |
| C | 1.245698 | 3.555669 | -1.524438 |
| C | 2.227350 | 4.047693 | -0.663193 |
| C | 2.849724 | 3.185541 | 0.236169 |

| | | | |
|---|---|---|---|
| C | -2.859862 | 3.176910 | -0.234200 |
| C | -2.240107 | 4.040476 | 0.665625 |
| C | -1.256890 | 3.551030 | 1.526552 |
| C | -0.900990 | 2.216136 | 1.405019 |
| Lu | 0.000601 | -0.615619 | -0.000107 |
| H | -4.475851 | -1.310293 | 0.594082 |
| H | -3.956459 | 0.300058 | 1.073623 |
| H | -3.229312 | -1.874736 | 2.501954 |
| H | -2.154780 | -0.455601 | 2.361548 |
| H | 2.155537 | -0.446014 | -2.360565 |
| H | 3.234239 | -1.861775 | -2.503532 |
| H | 4.479236 | -1.297030 | -0.594544 |
| H | 3.956252 | 0.312577 | -1.072689 |
| H | -4.163964 | 1.038329 | -1.293047 |
| H | -2.622923 | 0.829959 | -2.155766 |
| H | 4.160445 | 1.050674 | 1.293975 |
| H | 2.620039 | 0.837030 | 2.156487 |
| H | 0.756999 | 4.169174 | -2.281658 |
| H | 2.529597 | 5.095761 | -0.712435 |
| H | 3.651224 | 3.534729 | 0.888693 |
| H | -3.662497 | 3.523983 | -0.886458 |
| H | -2.545618 | 5.087568 | 0.715480 |
| H | -0.770090 | 4.165632 | 2.284105 |
| N | -1.451361 | 1.411722 | 0.490461 |
| N | 1.446772 | 1.416350 | -0.489578 |
| C | -0.132289 | 1.534305 | -2.292358 |
| O | -0.264643 | 0.277263 | -2.020147 |
| O | -0.745100 | 2.163157 | -3.129065 |
| C | 0.127281 | 1.533392 | 2.293298 |
| O | 0.263290 | 0.276879 | 2.020444 |
| O | 0.738292 | 2.163615 | 3.130293 |
| H | 2.477744 | -2.749895 | 3.114824 |
| H | 1.864351 | -1.063135 | 3.084463 |
| H | 4.158241 | -1.420061 | 2.022318 |
| H | 3.380148 | -2.512372 | 0.859641 |
| H | -2.469845 | -2.755978 | -3.115875 |
| H | -1.861821 | -1.067295 | -3.084869 |
| H | -3.372360 | -2.522383 | -0.860290 |
| H | -4.154258 | -1.431975 | -2.022214 |
| H | 0.260435 | -2.127542 | -3.409047 |
| H | -0.359283 | -3.789753 | -3.141695 |
| H | 2.029666 | -3.638742 | -2.423080 |
| H | 0.966400 | -3.929780 | -1.019142 |
| H | -0.254358 | -2.131165 | 3.407892 |
| H | 0.370917 | -3.791043 | 3.139109 |
| H | -0.954075 | -3.933604 | 1.016301 |
| H | -2.018424 | -3.647412 | 2.420418 |

MacropaLuΔ_λλλ_λλλ_opt.log

SCF (RwB97XD) = -1870.28844292
E(SCF)+ZPE(0 K)= -1869.677825
H(298 K)= -1869.643119
G(298 K)= -1869.739329
Lowest Frequency = 34.4722cm-1

73

| | | | |
|---|---|---|---|
| C | -3.707552 | 0.893215 | 0.403331 |
| N | -2.655667 | 0.495298 | 1.344155 |
| O | -1.957738 | 1.770502 | -0.974696 |
| C | -3.198906 | 1.075923 | -1.020897 |
| C | -2.496040 | 1.432609 | 2.450707 |
| C | -1.887789 | 2.741167 | 1.981510 |
| O | -0.551416 | 2.575170 | 1.517960 |
| C | 0.439139 | 2.751425 | 2.521734 |
| C | 1.767146 | 2.888579 | 1.824448 |
| O | 1.957387 | 1.770886 | 0.974759 |
| O | 0.550892 | 2.575356 | -1.517868 |
| C | -0.439704 | 2.751430 | -2.521634 |
| C | -1.767739 | 2.888270 | -1.824340 |
| N | 2.655590 | 0.495932 | -1.344141 |
| C | 2.495762 | 1.433249 | -2.450658 |
| C | 1.887227 | 2.741659 | -1.981418 |
| C | 3.198701 | 1.076566 | 1.020934 |
| C | 3.707389 | 0.894037 | -0.403301 |
| C | -2.775569 | -0.888806 | 1.782990 |
| C | 2.775794 | -0.888129 | -1.783032 |
| C | 2.381875 | -1.847123 | -0.685903 |
| C | -2.381437 | -1.847670 | 0.685822 |
| C | 1.195316 | -2.154571 | 1.280936 |
| C | 1.634985 | -3.462948 | 1.417112 |
| C | 2.479866 | -3.972721 | 0.431790 |
| C | 2.877662 | -3.150739 | -0.617981 |
| C | -2.876928 | -3.151396 | 0.617853 |
| C | -2.478950 | -3.973248 | -0.431950 |
| C | -1.634189 | -3.463247 | -1.417257 |
| C | -1.194813 | -2.154776 | -1.281032 |
| Lu | -0.000048 | 0.617363 | 0.000004 |
| H | -4.161439 | 1.838348 | 0.739784 |
| H | -4.527105 | 0.154494 | 0.390200 |
| H | -3.930355 | 1.653820 | -1.605414 |
| H | -3.037804 | 0.114529 | -1.527494 |
| H | 3.037798 | 0.115110 | 1.527478 |
| H | 3.930025 | 1.654588 | 1.605485 |
| H | 4.161069 | 1.839285 | -0.739710 |
| H | 4.527104 | 0.155495 | -0.390204 |
| H | -3.801414 | -1.129058 | 2.123229 |
| H | -2.106894 | -1.057539 | 2.640764 |
| H | 3.801692 | -1.128142 | -2.123284 |
| H | 2.107155 | -1.056971 | -2.640813 |
| H | 1.318424 | -4.038748 | 2.287080 |
| H | 2.848735 | -4.998449 | 0.495064 |
| H | 3.569228 | -3.509043 | -1.382131 |
| H | -3.568410 | -3.509886 | 1.381993 |
| H | -2.847586 | -4.999057 | -0.495261 |
| H | -1.317503 | -4.038941 | -2.287249 |
| N | -1.527762 | -1.390505 | -0.232644 |
| N | 1.528096 | -1.390185 | 0.232578 |
| C | 0.322002 | -1.473427 | 2.324645 |
| O | 0.088368 | -0.233791 | 2.058933 |
| O | -0.098539 | -2.105157 | 3.273004 |
| C | -0.321655 | -1.473398 | -2.324719 |
| O | -0.088283 | -0.233723 | -2.058959 |
| O | 0.099016 | -2.104999 | -3.273107 |

| | | | |
|---|---|---|---|
| H | 0.442792 | 1.880875 | 3.197552 |
| H | 0.233302 | 3.670154 | 3.095367 |
| H | 2.568462 | 2.948218 | 2.575797 |
| H | 1.786329 | 3.805799 | 1.212037 |
| H | -0.443171 | 1.880902 | -3.197482 |
| H | -0.234069 | 3.670223 | -3.095235 |
| H | -1.787121 | 3.805462 | -1.211894 |
| H | -2.569068 | 2.947764 | -2.575688 |
| H | 2.452584 | 3.167984 | -1.141520 |
| H | 1.899646 | 3.480352 | -2.797992 |
| H | 3.456398 | 1.649040 | -2.960396 |
| H | 1.823480 | 0.971131 | -3.186158 |
| H | -1.900375 | 3.479835 | 2.798103 |
| H | -2.453236 | 3.167389 | 1.141621 |
| H | -3.456723 | 1.648175 | 2.960452 |
| H | -1.823660 | 0.970611 | 3.186191 |

Macropa2-_ligand_opt.log

SCF (RwB97XD) = -1830.14717483
E(SCF)+ZPE(0 K)= -1829.546180
H(298 K)= -1829.510704
G(298 K)= -1829.612072
Lowest Frequency = 19.9838cm-1

72

| | | | |
|---|---|---|---|
| N | 3.841451 | 3.130788 | 8.905548 |
| C | 3.798678 | 1.905070 | 8.379525 |
| C | 4.819492 | 1.401542 | 7.573866 |
| H | 4.754430 | 0.384789 | 7.177028 |
| C | 5.896167 | 2.234142 | 7.263685 |
| H | 6.709847 | 1.874582 | 6.625622 |
| C | 5.891303 | 3.532702 | 7.745648 |
| C | 4.841371 | 3.947075 | 8.582607 |
| C | 4.800235 | 5.426253 | 9.053586 |
| O | 4.201347 | 5.663645 | 10.114790 |
| H | 6.657167 | 4.265813 | 7.489989 |
| N | -0.040372 | 4.917020 | 12.921994 |
| C | 1.048258 | 5.001695 | 12.150792 |
| C | 2.020104 | 3.996570 | 12.092458 |
| H | 2.856938 | 4.101191 | 11.393447 |
| C | 1.877334 | 2.912886 | 12.958173 |
| H | 2.627833 | 2.115508 | 12.965158 |
| C | 0.778017 | 2.860528 | 13.809243 |
| C | -0.201503 | 3.860859 | 13.724223 |
| C | -1.527077 | 3.689709 | 14.514668 |
| O | -2.552419 | 4.102262 | 13.937193 |
| H | 0.619062 | 2.055780 | 14.528921 |
| C | 2.555514 | 1.086547 | 8.667854 |
| H | 2.742662 | 0.022669 | 8.379363 |
| H | 2.406954 | 1.107201 | 9.756132 |
| N | 1.377598 | 1.638200 | 8.041081 |
| C | 1.386236 | 1.743520 | 6.597230 |
| H | 0.521832 | 1.194008 | 6.174731 |
| H | 2.289791 | 1.270981 | 6.172625 |
| C | 1.354636 | 3.181767 | 6.094809 |
| H | 0.938820 | 3.221124 | 5.061767 |

| | | | |
|---|---|---|---|
| H | 0.690962 | 3.776380 | 6.745699 |
| O | 2.664660 | 3.669594 | 6.102415 |
| C | 2.829313 | 5.059808 | 6.078896 |
| H | 2.403701 | 5.497002 | 5.148672 |
| H | 3.916899 | 5.222440 | 6.083737 |
| C | 2.276399 | 5.824844 | 7.267351 |
| H | 2.400096 | 5.236281 | 8.191840 |
| H | 2.900318 | 6.731053 | 7.373204 |
| O | 0.915964 | 6.175552 | 7.087664 |
| C | 0.532600 | 7.320964 | 7.791770 |
| H | -0.563768 | 7.390521 | 7.694560 |
| H | 0.956773 | 8.229364 | 7.306474 |
| C | 0.946258 | 7.386212 | 9.260873 |
| H | 2.045166 | 7.402775 | 9.320891 |
| H | 0.597172 | 8.387095 | 9.630093 |
| N | 0.481721 | 6.312119 | 10.098768 |
| C | 1.232638 | 6.268071 | 11.347261 |
| H | 0.961329 | 7.122261 | 12.010004 |
| H | 2.297518 | 6.348997 | 11.083614 |
| C | -0.962508 | 6.235469 | 10.229080 |
| H | -1.431567 | 7.220418 | 10.003653 |
| H | -1.222434 | 5.996404 | 11.273395 |
| C | -1.579751 | 5.171284 | 9.324101 |
| H | -2.665554 | 5.374389 | 9.196168 |
| H | -1.102014 | 5.212684 | 8.333122 |
| O | -1.387715 | 3.871768 | 9.803164 |
| C | -2.344229 | 3.426311 | 10.732239 |
| H | -3.314720 | 3.278482 | 10.208491 |
| H | -2.494459 | 4.126748 | 11.576654 |
| C | -1.875387 | 2.121307 | 11.349369 |
| H | -1.092414 | 2.331351 | 12.090785 |
| H | -2.718002 | 1.672984 | 11.897197 |
| O | -1.408880 | 1.185004 | 10.404396 |
| C | -0.049131 | 1.342549 | 10.071501 |
| H | 0.540883 | 0.562186 | 10.592788 |
| H | 0.340310 | 2.325308 | 10.379409 |
| C | 0.091406 | 1.235356 | 8.563078 |
| H | -0.190465 | 0.201512 | 8.239404 |
| H | -0.670966 | 1.918351 | 8.158441 |
| O | -1.414688 | 3.105325 | 15.608813 |
| O | 5.356531 | 6.215239 | 8.260487 |

MacropaTbΔ_δδδ_δδδ_opt.log

SCF (RwB97XD) = -1866.81747521
E(SCF)+ZPE(0 K)= -1866.207698
H(298 K)= -1866.172621
G(298 K)= -1866.270326
Lowest Frequency = 29.1368cm-1

73

| | | | |
|---|---|---|---|
| C | 3.564862 | -0.712824 | -0.639981 |
| N | 2.834997 | -0.558373 | 0.620351 |
| O | 1.692855 | -1.926114 | -1.516274 |
| C | 3.106771 | -1.906418 | -1.450076 |
| C | 3.167472 | -1.621592 | 1.573249 |
| C | 1.987754 | -2.061222 | 2.408174 |

| | | | |
|---|---|---|---|
| O | 0.965770 | -2.530469 | 1.521997 |
| C | -0.049470 | -3.295405 | 2.150492 |
| C | -1.157618 | -2.396800 | 2.651444 |
| O | -1.703653 | -1.699407 | 1.541966 |
| O | -0.996840 | -2.522393 | -1.449205 |
| C | -0.046902 | -3.489251 | -1.853368 |
| C | 1.167543 | -2.834765 | -2.468863 |
| N | -2.841987 | -0.532677 | -0.779802 |
| C | -3.118025 | -1.519547 | -1.830144 |
| C | -1.860775 | -2.040887 | -2.488839 |
| C | -3.051779 | -1.982197 | 1.211480 |
| C | -3.610198 | -0.811739 | 0.434107 |
| C | 3.064489 | 0.774578 | 1.190390 |
| C | -3.054142 | 0.840428 | -1.242339 |
| C | -2.479690 | 1.847938 | -0.274176 |
| C | 2.462776 | 1.850400 | 0.317115 |
| C | -0.971275 | 2.225595 | 1.449005 |
| C | -1.394673 | 3.536966 | 1.613883 |
| C | -2.398881 | 4.009001 | 0.769508 |
| C | -2.965133 | 3.151953 | -0.170403 |
| C | 2.911397 | 3.170599 | 0.301609 |
| C | 2.318576 | 4.069129 | -0.583224 |
| C | 1.330557 | 3.625698 | -1.462532 |
| C | 0.943342 | 2.295313 | -1.381991 |
| Tb | 0.011507 | -0.586622 | -0.021145 |
| H | 0.374619 | -3.895496 | 2.972114 |
| H | -0.444610 | -3.986677 | 1.390076 |
| H | -0.768626 | -1.645968 | 3.355547 |
| H | -1.932991 | -2.992089 | 3.157474 |
| H | -0.501367 | -4.209245 | -2.553698 |
| H | 0.253653 | -4.031249 | -0.944153 |
| H | 1.909018 | -3.612675 | -2.713450 |
| H | 0.917153 | -2.288633 | -3.393566 |
| H | 4.146575 | 0.971660 | 1.321183 |
| H | 2.593324 | 0.826965 | 2.183398 |
| H | -4.131085 | 1.053742 | -1.390172 |
| H | -2.555480 | 0.971920 | -2.214803 |
| H | -0.937231 | 4.146293 | 2.393662 |
| H | -2.757654 | 5.036445 | 0.858042 |
| H | -3.776599 | 3.485413 | -0.818933 |
| H | 3.713619 | 3.485465 | 0.970495 |
| H | 2.647423 | 5.110127 | -0.603754 |
| H | 0.861530 | 4.272218 | -2.204579 |
| N | 1.471696 | 1.459894 | -0.483472 |
| N | -1.479163 | 1.428363 | 0.502165 |
| C | 0.099050 | 1.591058 | 2.329665 |
| O | 0.313475 | 0.348796 | 2.054596 |
| O | 0.674239 | 2.258203 | 3.165006 |
| C | -0.099826 | 1.665844 | -2.297814 |
| O | -0.268969 | 0.405165 | -2.077700 |
| O | -0.696448 | 2.350712 | -3.103283 |
| H | 4.657090 | -0.800251 | -0.474674 |
| H | 3.533012 | -1.822151 | -2.463850 |
| H | 3.456967 | -2.862804 | -1.021652 |
| H | -4.678618 | -1.000550 | 0.210533 |
| H | -3.567218 | 0.069416 | 1.087647 |
| H | -3.643338 | -2.108590 | 2.132593 |

| | | | |
|---|---|---|---|
| H | -3.105017 | -2.925762 | 0.639717 |
| H | -1.349977 | -1.246468 | -3.051841 |
| H | -2.115868 | -2.876379 | -3.160921 |
| H | -3.653013 | -2.373358 | -1.389787 |
| H | -3.783881 | -1.105869 | -2.607540 |
| H | 2.301364 | -2.889402 | 3.064870 |
| H | 1.595797 | -1.232230 | 3.016401 |
| H | 3.990063 | -1.317583 | 2.246402 |
| H | 3.527311 | -2.498054 | 1.017753 |
| H | 3.401860 | 0.188826 | -1.242667 |

MacropaTbΔ_δδλ_δδλ_opt.log

SCF (RwB97XD) = -1866.81465787
E(SCF)+ZPE(0 K)= -1866.204456
H(298 K)= -1866.169495
G(298 K)= -1866.266498
Lowest Frequency = 31.4766cm-1

73

| | | | |
|---|---|---|---|
| C | 3.551405 | 0.997552 | -0.233346 |
| N | 2.627935 | 0.844066 | 0.893191 |
| O | 2.589395 | -0.984271 | -1.179386 |
| C | 3.797231 | -0.262510 | -1.040813 |
| C | 3.143934 | 0.059868 | 2.015932 |
| C | 3.081757 | -1.449736 | 1.833535 |
| O | 1.752591 | -1.857668 | 1.544879 |
| C | 1.172020 | -2.881410 | 2.337598 |
| C | -0.253931 | -2.476658 | 2.643105 |
| O | -0.942789 | -2.183004 | 1.440021 |
| O | 0.503482 | -2.720425 | -1.269304 |
| C | 1.826446 | -3.206555 | -1.420675 |
| C | 2.678867 | -2.117150 | -2.025397 |
| N | -2.084204 | -1.699226 | -1.099834 |
| C | -1.634261 | -2.374172 | -2.315983 |
| C | -0.480106 | -3.329867 | -2.098696 |
| C | -1.718960 | -3.216483 | 0.868838 |
| C | -2.698342 | -2.582118 | -0.103770 |
| C | 2.214351 | 2.180914 | 1.350365 |
| C | -3.004252 | -0.614337 | -1.464401 |
| C | -3.106729 | 0.431740 | -0.383349 |
| C | 1.312749 | 2.848712 | 0.340741 |
| C | -2.061118 | 1.473293 | 1.404505 |
| C | -3.145691 | 2.309734 | 1.629542 |
| C | -4.255565 | 2.180328 | 0.796452 |
| C | -4.247427 | 1.217066 | -0.208402 |
| C | 1.158236 | 4.231490 | 0.244615 |
| C | 0.317419 | 4.744808 | -0.740581 |
| C | -0.312165 | 3.877512 | -1.633395 |
| C | -0.094086 | 2.515953 | -1.475396 |
| Tb | 0.258385 | -0.448419 | -0.022560 |
| H | 2.515510 | 0.301927 | 2.883506 |
| H | 4.187670 | 0.338622 | 2.265730 |
| H | 3.418871 | -1.915414 | 2.772547 |
| H | 3.744482 | -1.816128 | 1.036821 |
| H | 1.727811 | -3.003562 | 3.280533 |
| H | 1.214343 | -3.838811 | 1.788096 |

| | | | |
|---|---|---|---|
| H | -0.254605 | -1.546367 | 3.226683 |
| H | -0.772842 | -3.268542 | 3.206253 |
| H | 1.844111 | -4.108612 | -2.050830 |
| H | 2.209298 | -3.472922 | -0.423750 |
| H | 3.722977 | -2.465061 | -2.095684 |
| H | 2.326470 | -1.849537 | -3.035632 |
| H | -1.298599 | -1.583941 | -2.999331 |
| H | -2.456643 | -2.935494 | -2.803510 |
| H | -0.795676 | -4.283640 | -1.641122 |
| H | -0.047001 | -3.565115 | -3.084276 |
| H | 3.091317 | 2.827033 | 1.547048 |
| H | 1.656615 | 2.080616 | 2.292522 |
| H | -4.012272 | -1.003986 | -1.702338 |
| H | -2.629537 | -0.121959 | -2.375032 |
| H | -3.098402 | 3.024938 | 2.451082 |
| H | -5.133038 | 2.813882 | 0.940855 |
| H | -5.113356 | 1.073023 | -0.856495 |
| H | 1.689998 | 4.891829 | 0.931246 |
| H | 0.172813 | 5.823736 | -0.825799 |
| H | -0.952422 | 4.222840 | -2.445483 |
| N | 0.663454 | 2.031337 | -0.487166 |
| N | -2.038581 | 0.588550 | 0.400092 |
| C | -0.826822 | 1.481866 | 2.297971 |
| O | -0.014641 | 0.512274 | 2.052985 |
| O | -0.679966 | 2.361894 | 3.122714 |
| C | -0.675473 | 1.456871 | -2.404388 |
| O | -0.237645 | 0.270728 | -2.151778 |
| O | -1.492732 | 1.769405 | -3.247572 |
| H | 3.116778 | 1.746124 | -0.907893 |
| H | 4.532111 | 1.393071 | 0.098412 |
| H | 4.185838 | 0.025858 | -2.032240 |
| H | 4.557120 | -0.911893 | -0.570171 |
| H | -3.301341 | -3.378073 | -0.582873 |
| H | -3.389743 | -1.976445 | 0.499098 |
| H | -2.295900 | -3.728798 | 1.657100 |
| H | -1.065421 | -3.965895 | 0.394185 |

MacropaTbΔ_δλδ_δλδ_opt.log

SCF (RwB97XD) = -1866.81684073
E(SCF)+ZPE(0 K)= -1866.206203
H(298 K)= -1866.171150
G(298 K)= -1866.269147
Lowest Frequency = 22.4195cm-1

73

| | | | |
|---|---|---|---|
| C | 3.521393 | -0.765689 | -0.732297 |
| N | 2.883710 | -0.515232 | 0.558916 |
| O | 1.629068 | -2.102359 | -1.351129 |
| C | 3.046541 | -2.055419 | -1.364636 |
| C | 3.296484 | -1.497469 | 1.563530 |
| C | 2.201814 | -1.829285 | 2.543275 |
| O | 1.083515 | -2.345423 | 1.825150 |
| C | 0.141805 | -2.915126 | 2.707029 |
| C | -1.090310 | -3.282279 | 1.932180 |
| O | -1.625957 | -2.102541 | 1.354194 |
| O | -1.079721 | -2.349691 | -1.822093 |

| | | | |
|---|---|---|---|
| C | -0.137263 | -2.920631 | -2.702361 |
| C | 1.095145 | -3.284264 | -1.926300 |
| N | -2.882788 | -0.520317 | -0.559174 |
| C | -3.293854 | -1.504128 | -1.562969 |
| C | -2.198191 | -1.835864 | -2.541640 |
| C | -3.043539 | -2.058610 | 1.366073 |
| C | -3.520489 | -0.770492 | 0.732078 |
| C | 3.136433 | 0.848187 | 1.029880 |
| C | -3.137414 | 0.842258 | -1.031553 |
| C | -2.486091 | 1.874986 | -0.147389 |
| C | 2.483247 | 1.879049 | 0.144912 |
| C | -0.867407 | 2.275721 | 1.462155 |
| C | -1.249247 | 3.601474 | 1.606013 |
| C | -2.293551 | 4.068216 | 0.807735 |
| C | -2.937111 | 3.192652 | -0.062494 |
| C | 2.932150 | 3.197350 | 0.058651 |
| C | 2.287089 | 4.070998 | -0.812396 |
| C | 1.243488 | 3.601760 | -1.610138 |
| C | 0.863786 | 2.275550 | -1.464890 |
| Tb | 0.000527 | -0.604018 | 0.000273 |
| H | 4.223347 | 1.054918 | 1.089844 |
| H | 2.724272 | 0.958740 | 2.045093 |
| H | -4.224619 | 1.047278 | -1.092107 |
| H | -2.725032 | 0.952416 | -2.046733 |
| H | -0.731876 | 4.226164 | 2.334509 |
| H | -2.623146 | 5.106590 | 0.880568 |
| H | -3.780649 | 3.521826 | -0.670978 |
| H | 3.775234 | 3.528494 | 0.666696 |
| H | 2.615001 | 5.109827 | -0.886315 |
| H | 0.725097 | 4.224865 | -2.339267 |
| N | 1.445709 | 1.460331 | -0.578499 |
| N | -1.447982 | 1.458637 | 0.576587 |
| C | 0.231882 | 1.629535 | 2.291711 |
| O | 0.375555 | 0.373823 | 2.037471 |
| O | 0.889231 | 2.294643 | 3.066669 |
| C | -0.234367 | 1.626575 | -2.293748 |
| O | -0.375888 | 0.370903 | -2.038081 |
| O | -0.892826 | 2.289671 | -3.069490 |
| H | 4.626400 | -0.803214 | -0.652096 |
| H | 3.407746 | -2.097068 | -2.406335 |
| H | 3.440270 | -2.944358 | -0.842706 |
| H | -4.625394 | -0.810117 | 0.651497 |
| H | -3.274397 | 0.059523 | 1.406950 |
| H | -3.405766 | -2.100033 | 2.407428 |
| H | -3.434800 | -2.948901 | 0.844586 |
| H | -1.873165 | -0.951539 | -3.110765 |
| H | -2.564434 | -2.607771 | -3.240217 |
| H | -3.585931 | -2.434257 | -1.059329 |
| H | -4.184340 | -1.160536 | -2.121646 |
| H | 2.569424 | -2.599813 | 3.242661 |
| H | 1.876084 | -0.944623 | 3.111473 |
| H | 4.186959 | -1.152295 | 2.121259 |
| H | 3.589222 | -2.427846 | 1.060744 |
| H | 0.872076 | -4.013876 | -1.127736 |
| H | 1.825953 | -3.740468 | -2.612195 |
| H | 0.125149 | -2.191932 | -3.489383 |
| H | -0.557027 | -3.823605 | -3.180987 |

| | | | |
|---|---|---|---|
| H | 3.273534 | 0.063137 | -1.407981 |
| H | -1.820346 | -3.737641 | 2.619468 |
| H | -0.866522 | -4.013686 | 1.135454 |
| H | 0.562671 | -3.816366 | 3.187948 |
| H | -0.121297 | -2.184643 | 3.492165 |

MacropaTbΔ_δλλ_δλλ_opt.log

SCF (RwB97XD) = -1866.81983104
E(SCF)+ZPE(0 K)= -1866.209387
H(298 K)= -1866.174486
G(298 K)= -1866.271211
Lowest Frequency = 32.2474cm-1

73

| | | | |
|---|---|---|---|
| C | -3.639276 | -0.724642 | 0.263311 |
| N | -2.748715 | -0.476546 | -0.872520 |
| O | -1.862031 | -1.880211 | 1.382063 |
| C | -3.254965 | -1.895728 | 1.144145 |
| C | -2.885432 | -1.431503 | -1.971588 |
| C | -2.159321 | -2.746647 | -1.734652 |
| O | -0.766162 | -2.564004 | -1.526036 |
| C | 0.031046 | -2.630766 | -2.702328 |
| C | 1.451107 | -2.891906 | -2.282142 |
| O | 1.861628 | -1.880917 | -1.381840 |
| O | 0.765569 | -2.564035 | 1.526373 |
| C | -0.031680 | -2.630418 | 2.702659 |
| C | -1.451810 | -2.891209 | 2.282487 |
| N | 2.748727 | -0.477240 | 0.872582 |
| C | 2.885143 | -1.432063 | 1.971803 |
| C | 2.158671 | -2.747041 | 1.735051 |
| C | 3.254552 | -1.896853 | -1.143892 |
| C | 3.639231 | -0.725780 | -0.263198 |
| C | -2.955363 | 0.899411 | -1.342104 |
| C | 2.955789 | 0.898727 | 1.341959 |
| C | 2.406873 | 1.897284 | 0.351057 |
| C | -2.406138 | 1.897949 | -0.351355 |
| C | 0.982094 | 2.225573 | -1.450160 |
| C | 1.368323 | 3.551102 | -1.591823 |
| C | 2.308222 | 4.055770 | -0.693481 |
| C | 2.852853 | 3.217053 | 0.275707 |
| C | -2.851693 | 3.217874 | -0.276217 |
| C | -2.306801 | 4.056567 | 0.692845 |
| C | -1.367072 | 3.551738 | 1.591275 |
| C | -0.981266 | 2.226064 | 1.449821 |
| Tb | -0.000002 | -0.605690 | 0.000031 |
| H | -4.028197 | 1.109729 | -1.516399 |
| H | -2.436910 | 1.038072 | -2.301745 |
| H | 4.028685 | 1.108745 | 1.516227 |
| H | 2.437377 | 1.037689 | 2.301579 |
| H | 0.934206 | 4.145996 | -2.395718 |
| H | 2.635636 | 5.095116 | -0.762781 |
| H | 3.616725 | 3.576221 | 0.966995 |
| H | -3.615442 | 3.577180 | -0.967570 |
| H | -2.633881 | 5.096030 | 0.761978 |
| H | -0.932771 | 4.146620 | 2.395079 |
| N | -1.463572 | 1.446468 | 0.476637 |

| | | | |
|---|---|---|---|
| N | 1.464156 | 1.445974 | -0.476856 |
| C | -0.017105 | 1.551523 | -2.381950 |
| O | -0.215395 | 0.308627 | -2.103673 |
| O | -0.565283 | 2.198033 | -3.252454 |
| C | 0.017721 | 1.551840 | 2.381713 |
| O | 0.215629 | 0.308843 | 2.103614 |
| O | 0.566096 | 2.198306 | 3.252124 |
| H | -4.686863 | -0.867069 | -0.068647 |
| H | -3.795883 | -1.802045 | 2.101950 |
| H | -3.548281 | -2.867487 | 0.710335 |
| H | 4.686763 | -0.868513 | 0.068803 |
| H | 3.624655 | 0.172924 | -0.892829 |
| H | 3.795518 | -1.803465 | -2.101700 |
| H | 3.547545 | -2.868653 | -0.709957 |
| H | -1.540023 | -3.878022 | 1.792246 |
| H | -2.096124 | -2.886230 | 3.178222 |
| H | 0.043416 | -1.680416 | 3.255614 |
| H | 0.307485 | -3.463858 | 3.338965 |
| H | -3.624383 | 0.174139 | 0.892828 |
| H | 2.095414 | -2.887216 | -3.177883 |
| H | 1.539047 | -3.878685 | -1.791782 |
| H | -0.308365 | -3.464193 | -3.338520 |
| H | -0.043789 | -1.680815 | -3.255405 |
| H | -3.948770 | -1.650171 | -2.196908 |
| H | -2.450580 | -0.962328 | -2.864860 |
| H | -2.318701 | -3.404924 | -2.603692 |
| H | -2.532774 | -3.284421 | -0.857023 |
| H | 2.531997 | -3.285059 | 0.857517 |
| H | 2.317845 | -3.405226 | 2.604197 |
| H | 2.450385 | -0.962626 | 2.864984 |
| H | 3.948411 | -1.650990 | 2.197195 |

MacropaTbΔ_δλλ_λδλ_opt.log

SCF (RwB97XD) = -1866.81840133
E(SCF)+ZPE(0 K)= -1866.207715
H(298 K)= -1866.172799
G(298 K)= -1866.269598
Lowest Frequency = 36.7686cm-1

73

| | | | |
|---|---|---|---|
| Tb | 0.902576 | 3.981891 | 9.212035 |
| N | 3.089828 | 2.658969 | 9.274685 |
| C | 3.150013 | 1.334654 | 9.418356 |
| C | 4.306441 | 0.720749 | 9.899234 |
| H | 4.338520 | -0.362303 | 10.026190 |
| C | 5.408989 | 1.513946 | 10.207536 |
| H | 6.320411 | 1.054292 | 10.595159 |
| C | 5.352142 | 2.890085 | 9.987819 |
| C | 4.163557 | 3.421006 | 9.506904 |
| C | 3.977707 | 4.903281 | 9.206807 |
| O | 2.808081 | 5.185307 | 8.744333 |
| H | 6.194548 | 3.557300 | 10.171478 |
| N | 1.937313 | 4.401641 | 11.552171 |
| C | 2.547005 | 5.529576 | 11.914774 |
| C | 3.444068 | 5.549559 | 12.983521 |
| H | 3.943468 | 6.479280 | 13.260183 |

| | | | |
|---|---|---|---|
| C | 3.684251 | 4.368999 | 13.681153 |
| H | 4.391181 | 4.359898 | 14.513237 |
| C | 2.992934 | 3.209776 | 13.329215 |
| C | 2.114075 | 3.279702 | 12.258050 |
| C | 1.275151 | 2.099780 | 11.792339 |
| O | 0.481742 | 2.402687 | 10.822449 |
| H | 3.107595 | 2.264169 | 13.859586 |
| C | 1.917901 | 0.564850 | 9.007111 |
| H | 2.221950 | -0.418325 | 8.597131 |
| H | 1.310345 | 0.372177 | 9.904173 |
| N | 1.122214 | 1.318077 | 8.033183 |
| C | 1.842963 | 1.440425 | 6.765364 |
| H | 1.860404 | 0.481345 | 6.210748 |
| H | 2.885115 | 1.708822 | 6.980321 |
| C | 1.262672 | 2.508901 | 5.869958 |
| H | 1.925593 | 2.636243 | 4.996910 |
| H | 0.262271 | 2.243080 | 5.483595 |
| O | 1.177584 | 3.726455 | 6.584516 |
| C | 0.867010 | 4.819961 | 5.744694 |
| H | -0.157149 | 4.700993 | 5.346370 |
| H | 1.570136 | 4.858241 | 4.894934 |
| C | 0.985291 | 6.090703 | 6.535734 |
| H | 2.022198 | 6.245917 | 6.873556 |
| H | 0.664252 | 6.934230 | 5.903346 |
| O | 0.126579 | 5.976518 | 7.664796 |
| C | -0.207831 | 7.213517 | 8.275091 |
| H | -1.234937 | 7.112797 | 8.638868 |
| H | -0.220075 | 8.008892 | 7.512615 |
| C | 0.772428 | 7.603835 | 9.370318 |
| H | 1.762690 | 7.717730 | 8.908266 |
| H | 0.479962 | 8.596973 | 9.767852 |
| N | 0.908749 | 6.611559 | 10.434931 |
| C | 2.202410 | 6.758832 | 11.111997 |
| H | 2.212632 | 7.645049 | 11.775782 |
| H | 2.989175 | 6.905659 | 10.357506 |
| C | -0.162788 | 6.633410 | 11.431055 |
| H | -0.301241 | 7.646612 | 11.858798 |
| H | 0.142632 | 5.976908 | 12.255812 |
| C | -1.505797 | 6.145675 | 10.924431 |
| H | -2.138304 | 5.880752 | 11.788810 |
| H | -2.042311 | 6.927565 | 10.360893 |
| O | -1.321210 | 5.006851 | 10.102171 |
| C | -2.515307 | 4.519710 | 9.509921 |
| H | -2.607951 | 4.925735 | 8.486791 |
| H | -3.391714 | 4.842891 | 10.093279 |
| C | -2.471485 | 3.018514 | 9.500847 |
| H | -2.371695 | 2.646347 | 10.535453 |
| H | -3.409269 | 2.624820 | 9.070140 |
| O | -1.365635 | 2.584086 | 8.736422 |
| C | -1.222742 | 1.167253 | 8.828158 |
| H | -2.189144 | 0.692157 | 8.587215 |
| H | -0.937161 | 0.910368 | 9.859642 |
| C | -0.193553 | 0.709680 | 7.828325 |
| H | -0.134585 | -0.394993 | 7.854235 |
| H | -0.556088 | 0.979187 | 6.827571 |
| O | 1.416778 | 1.005839 | 12.300733 |
| O | 4.874382 | 5.687724 | 9.444790 |

MacropaTbΔ_λδδ_λδδ_opt.log

SCF (RwB97XD) = -1866.81379310
E(SCF)+ZPE(0 K)= -1866.203916
H(298 K)= -1866.168891
G(298 K)= -1866.266077
Lowest Frequency = 34.8662cm-1

73

| | | | |
|---|---|---|---|
| C | 3.707509 | 0.743274 | 0.444431 |
| N | 2.856078 | 0.599468 | -0.751331 |
| O | 1.829797 | 1.828176 | 1.451450 |
| C | 2.929377 | 0.977729 | 1.733539 |
| C | 3.073556 | 1.691961 | -1.705147 |
| C | 1.838297 | 2.081799 | -2.481401 |
| O | 0.852066 | 2.529556 | -1.543504 |
| C | -0.168781 | 3.351122 | -2.085601 |
| C | -1.325063 | 2.516729 | -2.578443 |
| O | -1.830162 | 1.827889 | -1.451317 |
| O | -0.852580 | 2.529251 | 1.543700 |
| C | 0.168089 | 3.351001 | 2.085855 |
| C | 1.324554 | 2.516823 | 2.578629 |
| N | -2.856184 | 0.598802 | 0.751374 |
| C | -3.073894 | 1.691178 | 1.705273 |
| C | -1.838720 | 2.081216 | 2.481561 |
| C | -2.929561 | 0.977231 | -1.733470 |
| C | -3.707644 | 0.742516 | -0.444380 |
| C | 2.999498 | -0.722814 | -1.366274 |
| C | -2.999335 | -0.723557 | 1.366216 |
| C | -2.462509 | -1.790528 | 0.441697 |
| C | 2.462910 | -1.789968 | -0.441827 |
| C | -1.024859 | -2.226460 | -1.330297 |
| C | -1.432682 | -3.549693 | -1.416320 |
| C | -2.389915 | -3.995341 | -0.503859 |
| C | -2.930020 | -3.105029 | 0.421153 |
| C | 2.930715 | -3.104366 | -0.421374 |
| C | 2.390809 | -3.994863 | 0.503578 |
| C | 1.433477 | -3.549491 | 1.416069 |
| C | 1.025360 | -2.226342 | 1.330139 |
| Tb | -0.000060 | 0.650890 | 0.000031 |
| H | 4.402938 | 1.581808 | 0.293312 |
| H | 4.336996 | -0.149411 | 0.585753 |
| H | 3.591599 | 1.459953 | 2.471583 |
| H | 2.553095 | 0.042022 | 2.173577 |
| H | 0.233119 | 3.980106 | -2.895324 |
| H | -0.511365 | 4.009201 | -1.272901 |
| H | -0.998208 | 1.793741 | -3.346289 |
| H | -2.105412 | 3.168484 | -3.008294 |
| H | -0.233946 | 3.979835 | 2.895627 |
| H | 0.510526 | 4.009216 | 1.273203 |
| H | 2.104764 | 3.168716 | 3.008524 |
| H | 0.997861 | 1.793708 | 3.346425 |
| H | -2.553082 | 0.041636 | -2.173578 |
| H | -3.591886 | 1.459369 | -2.471478 |
| H | -4.403249 | 1.580893 | -0.293200 |
| H | -4.336942 | -0.150291 | -0.585768 |

| | | | |
|---|---|---|---|
| H | 4.055951 | -0.943917 | -1.613385 |
| H | 2.427962 | -0.755917 | -2.306112 |
| H | -4.055745 | -0.944900 | 1.613293 |
| H | -2.427810 | -0.756613 | 2.306061 |
| H | -1.004360 | -4.189495 | -2.188239 |
| H | -2.735092 | -5.030927 | -0.529174 |
| H | -3.706229 | -3.420865 | 1.119575 |
| H | 3.706995 | -3.419980 | -1.119817 |
| H | 2.736219 | -5.030373 | 0.528822 |
| H | 1.005297 | -4.189442 | 2.187943 |
| N | 1.503480 | -1.397614 | 0.396165 |
| N | -1.503164 | -1.397903 | -0.396265 |
| C | -0.021271 | -1.594245 | -2.288654 |
| O | 0.147093 | -0.329642 | -2.080015 |
| O | 0.543378 | -2.275410 | -3.118643 |
| C | 0.021633 | -1.594418 | 2.288542 |
| O | -0.146994 | -0.329833 | 2.080002 |
| O | -0.542877 | -2.275767 | 3.118473 |
| H | -2.094050 | 2.914614 | 3.156007 |
| H | -1.434098 | 1.242659 | 3.068324 |
| H | -3.883883 | 1.452975 | 2.421263 |
| H | -3.390203 | 2.577826 | 1.140253 |
| H | 2.093451 | 2.915302 | -3.155785 |
| H | 1.433850 | 1.243203 | -3.068227 |
| H | 3.389681 | 2.578633 | -1.140061 |
| H | 3.883592 | 1.453981 | -2.421159 |

MacropaTbΔ_λδλ_λδλ_opt.log

SCF (RwB97XD) = -1866.82308018
E(SCF)+ZPE(0 K)= -1866.213004
H(298 K)= -1866.178092
G(298 K)= -1866.275144
Lowest Frequency = 29.1414cm-1

73

| | | | |
|---|---|---|---|
| C | 3.682240 | 0.960109 | -0.268601 |
| N | 2.643764 | 0.551030 | -1.228042 |
| O | 1.983097 | 1.870554 | 1.121172 |
| C | 3.182176 | 1.111955 | 1.158352 |
| C | 2.532122 | 1.472120 | -2.360265 |
| C | 1.927201 | 2.803173 | -1.961853 |
| O | 0.598502 | 2.604783 | -1.491137 |
| C | -0.440525 | 3.370652 | -2.076307 |
| C | -1.614737 | 2.465481 | -2.352681 |
| O | -1.983136 | 1.870429 | -1.121285 |
| O | -0.598565 | 2.604843 | 1.491002 |
| C | 0.440444 | 3.370801 | 2.076087 |
| C | 1.614689 | 2.465692 | 2.352523 |
| N | -2.643765 | 0.551027 | 1.228015 |
| C | -2.532149 | 1.472175 | 2.360193 |
| C | -1.927263 | 2.803226 | 1.961722 |
| C | -3.182200 | 1.111804 | -1.158411 |
| C | -3.682251 | 0.960034 | 0.268555 |
| C | 2.818608 | -0.830074 | -1.681960 |
| C | -2.818577 | -0.830058 | 1.682004 |
| C | -2.445383 | -1.818521 | 0.605841 |

| | | | |
|---|---|---|---|
| C | 2.445441 | -1.818489 | -0.605742 |
| C | -1.200733 | -2.187406 | -1.317039 |
| C | -1.674093 | -3.484953 | -1.448679 |
| C | -2.573397 | -3.951401 | -0.489832 |
| C | -2.982809 | -3.105013 | 0.536763 |
| C | 2.982900 | -3.104963 | -0.536594 |
| C | 2.573512 | -3.951304 | 0.490049 |
| C | 1.674197 | -3.484826 | 1.448872 |
| C | 1.200804 | -2.187299 | 1.317161 |
| Tb | -0.000003 | 0.635377 | -0.000015 |
| H | 4.109431 | 1.923484 | -0.585526 |
| H | 4.520391 | 0.242917 | -0.268063 |
| H | 3.941384 | 1.647771 | 1.752594 |
| H | 2.987894 | 0.142654 | 1.642756 |
| H | 1.868613 | 1.004471 | -3.100306 |
| H | 3.513022 | 1.655454 | -2.842560 |
| H | 1.914960 | 3.468536 | -2.838284 |
| H | 2.504967 | 3.302289 | -1.168050 |
| H | -0.102179 | 3.820802 | -3.022153 |
| H | -0.729950 | 4.181054 | -1.386200 |
| H | -1.338175 | 1.679409 | -3.075657 |
| H | -2.453869 | 3.060003 | -2.752845 |
| H | 0.102097 | 3.821024 | 3.021897 |
| H | 0.729832 | 4.181151 | 1.385902 |
| H | 2.453805 | 3.060272 | 2.752635 |
| H | 1.338158 | 1.679668 | 3.075565 |
| H | -1.868628 | 1.004580 | 3.100258 |
| H | -3.513055 | 1.655504 | 2.842478 |
| H | -2.505049 | 3.302302 | 1.167908 |
| H | -1.915026 | 3.468620 | 2.838130 |
| H | -2.987904 | 0.142479 | -1.642761 |
| H | -3.941423 | 1.647571 | -1.752678 |
| H | -4.109451 | 1.923423 | 0.585425 |
| H | -4.520393 | 0.242832 | 0.268065 |
| H | 3.856323 | -1.020994 | -2.015816 |
| H | 2.162659 | -1.010094 | -2.547517 |
| H | -3.856288 | -1.020984 | 2.015867 |
| H | -2.162626 | -1.010016 | 2.547573 |
| H | -1.341423 | -4.086940 | -2.294620 |
| H | -2.972902 | -4.965528 | -0.554489 |
| H | -3.710217 | -3.433415 | 1.280689 |
| H | 3.710315 | -3.433388 | -1.280503 |
| H | 2.973043 | -4.965418 | 0.554762 |
| H | 1.341543 | -4.086776 | 2.294846 |
| N | 1.552075 | -1.401892 | 0.292006 |
| N | -1.552025 | -1.401951 | -0.291928 |
| C | -0.268827 | -1.540420 | -2.334631 |
| O | -0.071903 | -0.284698 | -2.116224 |
| O | 0.224494 | -2.213168 | -3.217767 |
| C | 0.268881 | -1.540281 | 2.334718 |
| O | 0.071927 | -0.284576 | 2.116244 |
| O | -0.224426 | -2.212995 | 3.217888 |

MacropaTbΔ_λλδ_λλδ_opt.log

SCF (RwB97XD) = -1866.81005689
E(SCF)+ZPE(0 K)= -1866.199750

H(298 K)= -1866.164921
G(298 K)= -1866.261019
Lowest Frequency = 39.6774cm-1

73

| | | | |
|---|---|---|---|
| C | -3.578725 | -0.723292 | 0.714373 |
| N | -2.859248 | -0.554053 | -0.559913 |
| O | -1.722645 | -2.153236 | 1.189690 |
| C | -2.679833 | -1.297299 | 1.809732 |
| C | -3.183505 | -1.647963 | -1.478753 |
| C | -2.120772 | -1.918092 | -2.512894 |
| O | -0.936049 | -2.355019 | -1.856163 |
| C | 0.043042 | -2.796560 | -2.774280 |
| C | 1.249426 | -3.216597 | -1.974525 |
| O | 1.735757 | -2.133578 | -1.203700 |
| O | 0.950434 | -2.361886 | 1.840775 |
| C | -0.025728 | -2.816593 | 2.755563 |
| C | -1.229311 | -3.238648 | 1.952659 |
| N | 2.861966 | -0.539415 | 0.557370 |
| C | 3.193240 | -1.637521 | 1.468678 |
| C | 2.132158 | -1.921709 | 2.500750 |
| C | 2.687332 | -1.266961 | -1.817524 |
| C | 3.582558 | -0.695197 | -0.718029 |
| C | -3.086279 | 0.767382 | -1.146287 |
| C | 3.080642 | 0.779346 | 1.152839 |
| C | 2.455271 | 1.843389 | 0.282968 |
| C | -2.467763 | 1.829379 | -0.269035 |
| C | 0.910262 | 2.249335 | -1.400776 |
| C | 1.270892 | 3.585489 | -1.495801 |
| C | 2.260608 | 4.053659 | -0.630221 |
| C | 2.878214 | 3.172652 | 0.254362 |
| C | -2.899691 | 3.155501 | -0.230755 |
| C | -2.287871 | 4.034282 | 0.660048 |
| C | -1.294860 | 3.566587 | 1.522098 |
| C | -0.925244 | 2.233610 | 1.417409 |
| Tb | 0.001735 | -0.653721 | -0.001723 |
| H | -4.458187 | -1.370799 | 0.564275 |
| H | -3.966767 | 0.242153 | 1.067164 |
| H | -3.276696 | -1.871451 | 2.535556 |
| H | -2.148645 | -0.497909 | 2.350720 |
| H | 2.150932 | -0.467057 | -2.352577 |
| H | 3.287827 | -1.831926 | -2.547549 |
| H | 4.465996 | -1.338298 | -0.572421 |
| H | 3.964627 | 0.275115 | -1.063929 |
| H | -4.164911 | 0.978171 | -1.282142 |
| H | -2.614976 | 0.816468 | -2.139745 |
| H | 4.157929 | 0.996048 | 1.290074 |
| H | 2.609157 | 0.818633 | 2.146654 |
| H | 0.781916 | 4.218136 | -2.236954 |
| H | 2.570368 | 5.100223 | -0.661990 |
| H | 3.680544 | 3.506387 | 0.913869 |
| H | -3.704467 | 3.488518 | -0.887638 |
| H | -2.604730 | 5.078457 | 0.699433 |
| H | -0.810092 | 4.197143 | 2.267785 |
| N | -1.473303 | 1.414563 | 0.514824 |
| N | 1.463805 | 1.427495 | -0.504088 |
| C | -0.129776 | 1.590614 | -2.296950 |

| | | | |
|---|---|---|---|
| O | -0.255746 | 0.324450 | -2.070331 |
| O | -0.759472 | 2.251420 | -3.096468 |
| C | 0.119065 | 1.575384 | 2.308964 |
| O | 0.252964 | 0.311616 | 2.073698 |
| O | 0.744669 | 2.234674 | 3.112936 |
| H | 2.494015 | -2.725470 | 3.164840 |
| H | 1.889944 | -1.036188 | 3.109525 |
| H | 4.153989 | -1.451868 | 1.987862 |
| H | 3.317888 | -2.547345 | 0.866501 |
| H | -2.477475 | -2.719623 | -3.182445 |
| H | -1.884511 | -1.026862 | -3.115648 |
| H | -3.302125 | -2.562714 | -0.882852 |
| H | -4.145531 | -1.464995 | -1.996527 |
| H | 0.293658 | -1.977456 | -3.472123 |
| H | -0.337655 | -3.652384 | -3.359428 |
| H | 2.040071 | -3.609069 | -2.633407 |
| H | 0.971882 | -4.005446 | -1.259392 |
| H | -0.281734 | -2.004325 | 3.459409 |
| H | 0.360584 | -3.674179 | 3.334417 |
| H | -0.946596 | -4.020455 | 1.231840 |
| H | -2.017412 | -3.641071 | 2.608578 |

MacropaTbΔ_λλλ_λλλ_opt.log

SCF (RwB97XD) = -1866.81904345
E(SCF)+ZPE(0 K)= -1866.208900
H(298 K)= -1866.174015
G(298 K)= -1866.270905
Lowest Frequency = 28.1350cm-1

73

| | | | |
|---|---|---|---|
| C | -3.693026 | 0.941849 | 0.399819 |
| N | -2.631774 | 0.517383 | 1.325043 |
| O | -1.988299 | 1.839936 | -1.013860 |
| C | -3.217451 | 1.123682 | -1.034323 |
| C | -2.483386 | 1.427754 | 2.461127 |
| C | -1.872399 | 2.753017 | 2.043474 |
| O | -0.528869 | 2.606361 | 1.597978 |
| C | 0.452083 | 2.708401 | 2.622014 |
| C | 1.790565 | 2.886975 | 1.950687 |
| O | 1.987891 | 1.840094 | 1.014397 |
| O | 0.528266 | 2.607038 | -1.597183 |
| C | -0.452708 | 2.709170 | -2.621187 |
| C | -1.791238 | 2.887174 | -1.949805 |
| N | 2.631676 | 0.518467 | -1.324931 |
| C | 2.483060 | 1.429174 | -2.460718 |
| C | 1.871761 | 2.754157 | -2.042626 |
| C | 3.217219 | 1.124134 | 1.034628 |
| C | 3.692832 | 0.942887 | -0.399575 |
| C | -2.786122 | -0.870909 | 1.756161 |
| C | 2.786367 | -0.869644 | -1.756511 |
| C | 2.429816 | -1.839206 | -0.656885 |
| C | -2.429325 | -1.840020 | 0.656216 |
| C | 1.231227 | -2.187690 | 1.297782 |
| C | 1.701277 | -3.486648 | 1.427719 |
| C | 2.574188 | -3.965656 | 0.451310 |
| C | 2.961309 | -3.128604 | -0.590967 |

| | | | |
|---|---|---|---|
| C | -2.960490 | -3.129532 | 0.589874 |
| C | -2.573156 | -3.966145 | -0.452677 |
| C | -1.700366 | -3.486594 | -1.428928 |
| C | -1.230646 | -2.187560 | -1.298564 |
| Tb | -0.000055 | 0.635934 | 0.000074 |
| H | -4.119183 | 1.895187 | 0.747825 |
| H | -4.527674 | 0.220148 | 0.400980 |
| H | -3.973573 | 1.688623 | -1.600667 |
| H | -3.054395 | 0.161442 | -1.540737 |
| H | 3.054398 | 0.161689 | 1.540727 |
| H | 3.973202 | 1.689077 | 1.601156 |
| H | 4.118754 | 1.896443 | -0.747271 |
| H | 4.527654 | 0.221389 | -0.400979 |
| H | -3.814643 | -1.078374 | 2.108548 |
| H | -2.111837 | -1.061898 | 2.605033 |
| H | 3.814937 | -1.076736 | -2.108972 |
| H | 2.112124 | -1.060516 | -2.605443 |
| H | 1.385307 | -4.079760 | 2.286226 |
| H | 2.969823 | -4.981436 | 0.513597 |
| H | 3.667820 | -3.466128 | -1.350856 |
| H | -3.666917 | -3.467485 | 1.349651 |
| H | -2.968532 | -4.982004 | -0.515297 |
| H | -1.384243 | -4.079342 | -2.287630 |
| N | -1.560974 | -1.409119 | -0.260261 |
| N | 1.561357 | -1.408825 | 0.259736 |
| C | 0.323193 | -1.538618 | 2.334948 |
| O | 0.104692 | -0.288368 | 2.109148 |
| O | -0.135631 | -2.205567 | 3.240910 |
| C | -0.322771 | -1.537925 | -2.335516 |
| O | -0.104566 | -0.287698 | -2.109302 |
| O | 0.136211 | -2.204465 | -3.241699 |
| H | 0.444623 | 1.793835 | 3.237113 |
| H | 0.244453 | 3.586872 | 3.255412 |
| H | 2.581952 | 2.880793 | 2.715064 |
| H | 1.825469 | 3.848670 | 1.412532 |
| H | -0.445009 | 1.794817 | -3.236600 |
| H | -0.245302 | 3.587910 | -3.254287 |
| H | -1.826394 | 3.848684 | -1.411336 |
| H | -2.582620 | 2.881039 | -2.714188 |
| H | 2.421298 | 3.204439 | -1.204444 |
| H | 1.903275 | 3.466413 | -2.882378 |
| H | 3.450601 | 1.624004 | -2.964871 |
| H | 1.819422 | 0.945052 | -3.190037 |
| H | -1.904075 | 3.464987 | 2.883462 |
| H | -2.422046 | 3.203450 | 1.205446 |
| H | -3.450979 | 1.622191 | 2.965334 |
| H | -1.819643 | 0.943554 | 3.190297 |
